# Association of Intensive Blood Pressure Control and Living Arrangement on Cardiovascular Outcomes by Race: Post Hoc Analysis of SPRINT Randomized Clinical Trial

**DOI:** 10.1001/jamanetworkopen.2022.2037

**Published:** 2022-03-14

**Authors:** Kosuke Inoue, Karol E. Watson, Naoki Kondo, Tamara Horwich, William Hsu, Alex A. T. Bui, O. Kenrik Duru

**Affiliations:** 1 Department of Social Epidemiology, Graduate School of Medicine, Kyoto University, Kyoto, Japan; 2 Department of Epidemiology, University of California Los Angeles Fielding School of Public Health, Los Angeles; 3 Department of Medicine, Division of Cardiology, University of California Los Angeles, Los Angeles; 4 Institute for Future Initiatives, The University of Tokyo, Tokyo, Japan; 5 Medical and Imaging Informatics Group, Department of Radiological Sciences, David Geffen School of Medicine, University of California Los Angeles, Los Angeles; 6 Department of Radiological Sciences, David Geffen School of Medicine, University of California Los Angeles, Los Angeles; 7 Department of Bioengineering, University of California Los Angeles Samueli School of Engineering, Los Angeles; 8 Division of General Internal Medicine and Health Services Research, David Geffen School of Medicine at University of California Los Angeles, Los Angeles

## Abstract

**Importance:**

Living alone, a key proxy of social isolation, is a risk factor for cardiovascular disease. In addition, Black race is associated with less optimal blood pressure (BP) control than in other racial or ethnic groups. However, it is not clear whether living arrangement status modifies the beneficial effects of intensive BP control on reduction in cardiovascular events among Black individuals.

**Objective:**

To examine whether the association of intensive BP control with cardiovascular events differs by living arrangement among Black individuals and non-Black individuals (eg, individuals who identified as Alaskan Native, American Indian, Asian, Native Hawaiian, Pacific Islander, White, or other) in the Systolic Blood Pressure Intervention Trial (SPRINT).

**Design, Setting, and Participants:**

This secondary analysis incorporated data from SPRINT, a multicenter study of individuals with increased risk for cardiovascular disease and free of diabetes, enrolled at 102 clinical sites in the United States between November 2010 and March 2013. Race and living arrangement (ie, living alone or living with others) were self-reported. Data were collected between November 2010 and March 2013 and analyzed from January 2021 to October 2021.

**Exposures:**

The SPRINT participants were randomized to a systolic BP target of either less than 120 mm Hg (intensive treatment group) or less than 140 mm Hg (standard treatment group). Antihypertensive medications were adjusted to achieve the targets in each group.

**Main Outcomes and Measures:**

Cox proportional hazards model was used to investigate the association of intensive treatment with the incident composite cardiovascular outcome (by August 20, 2015) according to living arrangement among Black individuals and other individuals. Transportability formula was applied to generalize the SPRINT findings to hypothetical external populations by varying the proportion of Black race and living arrangement status.

**Results:**

Among the 9342 total participants, the mean (SD) age was 67.9 (9.4) years; 2793 participants [30%] were Black, 2714 [29%] lived alone, and 3320 participants (35.5%) were female. Over a median (IQR) follow-up of 3.22 (2.74-3.76) years, the primary composite cardiovascular outcome was observed in 67 of 1001 Black individuals living alone (6.7%), 76 of 1792 Black individuals living with others (4.2%), 108 of 1713 non-Black individuals living alone (6.3%), and 311 of 4836 non-Black individuals living with others (6.4%). The intensive treatment group showed a significantly lower rate of the composite cardiovascular outcome than the standard treatment group among Black individuals living with others (hazard ratio [HR], 0.53 [95% CI, 0.33-0.85]) but not among those living alone (HR, 1.07 [95% CI, 0.66-1.73]; *P* for interaction = .04). The association was observed among individuals who were not Black regardless of living arrangement status. Using transportability, we found a smaller or null association between intensive control and cardiovascular outcomes among hypothetical populations of 60% Black individuals or more and 60% or more of individuals living alone.

**Conclusions and Relevance:**

Intensive BP control was associated with a lower rate of cardiovascular events among Black individuals living with others and individuals who were not Black but not among Black individuals living alone.

**Trial Registration:**

ClinicalTrials.gov Identifier: NCT01206062

## Key Points

**Question:**

Does the association of intensive blood pressure control with cardiovascular disease outcomes differ by Black race and living arrangement status (ie, living alone or not)?

**Findings:**

In this secondary analysis of a randomized clinical trial of 9342 individuals, intensive blood pressure control (ie, treating to a systolic blood pressure target of <120 mm Hg) was associated with lower rates of cardiovascular events compared with standard blood pressure control (ie, treating to a systolic blood pressure target of <140 mm Hg) among Black individuals living with others, but not among those living alone. The association was observed among non-Black individuals regardless of living arrangement.

**Meaning:**

These findings highlight that living arrangement status, a key component of social isolation, may be critical information when building a tailored approach to improve cardiovascular health among Black individuals.

## Teaser

This post hoc analysis of the Systolic Blood Pressure Intervention Trial examines whether the association of intensive blood pressure control with cardiovascular events differs by living arrangement among Black individuals and non-Black individuals (eg, individuals who identified as Alaskan Native, American Indian, Asian, Native Hawaiian, Pacific Islander, White, or other).

## Introduction

Hypertension is a leading cause of cardiovascular disease (CVD) and death, affecting nearly half of adults in the United States, and imposing substantial health and economic burden on individuals and society.^1,2^ Importantly, the prevalence and control rates of hypertension vary by race or ethnicity, with significantly higher prevalence and lower rates of optimal control among Black patients than White patients.^3,4^ Moreover, the mortality rate related to hypertension was nearly double among Black patients compared with White patients in 2018.^5,6^ However, underlying mechanisms of such racial disparities have not been well established because of the complex interaction across biological, environmental, behavioral, genetic, social factors, and systemic racism.^7^ This suggests that we need further evidence to build tailored approaches for hypertension prevention and control that consider individual sociodemographic characteristics.^8,9^ To consider and design effective policy or clinical interventions that improve the unfavorable health outcomes because of hypertension among Black individuals, greater insight into factors that modify (ie, increase or decrease) the treatment benefit among this racial group is needed.

Social isolation is a major public health issue worldwide and a devastating risk factor for CVD.^10,11^ A previous meta-analysis of 16 longitudinal studies showed that loneliness and social isolation were associated with increased risks of incident coronary heart disease (relative risk, 1.29) and stroke (relative risk, 1.32).^11^ Additionally, the number of individuals living alone is substantially increasing, particularly for older adults in the US.^12^ According to a recent report based on the US Census data, the percentage of Black adults who are 50 years or older without living close kin was projected to more than triple from 2015 to 2060, larger than what was projected for older White adults.^13^ Given the possible impact of living alone itself on developing and deteriorating CVD through both social and biological pathways, such as poor adherence to medications and vascular damage,^14,15,16,17^ it is important to evaluate whether the benefit of blood pressure (BP) control to reduce CVD risks differs by living arrangement status among Black individuals and other individuals.

In this study, we aimed to investigate whether the association of intensive BP control with cardiovascular events varies by living arrangement status among Black individuals and non-Black individuals (a prespecified racial subgroup in the Systolic Blood Pressure Intervention Trial [SPRINT]that included individuals who identified as Alaskan Native, American Indian, Asian, Native Hawaiian, Pacific Islander, White, or other) using data from SPRINT.^18,19^ SPRINT was a multicenter, large randomized clinical trial (RCT) demonstrating 25% relative reduction in cardiovascular morbidity and mortality with intensive BP treatment (goal systolic BP <120 mm Hg) over standard BP treatment (goal systolic BP <140 mm Hg). Because participants in SPRINT do not necessarily mirror the target population of interest, we applied a transportability formula to extrapolate the findings from SPRINT to hypothetical target populations, varying the distribution of Black race and living arrangement status. This approach allows us to quantify the potential benefits of intensive BP control in external populations with different distribution of baseline characteristics,^20,21,22,23^ and thus maximize the use of the trial findings by improving its generalizability, which is a longstanding challenge in RCTs.

## Methods

This post hoc analysis of a randomized clinical trial followed the Strengthening the Reporting of Observational Studies in Epidemiology (STROBE) reporting guideline. It was exempted by the institutional review board at the University of California, Los Angeles because this is a secondary data analysis using previously collected deidentified data.

### Study Cohort

This is a post hoc analysis of SPRINT, a multicenter, randomized trial conducted at 102 clinical sites in the United States between 2010 and 2013. SPRINT enrolled 9361 adults aged 50 years or older with hypertension at high cardiovascular risk, which included the presence of clinical or subclinical CVD, chronic kidney disease, the 10-year risk of CVD of 15% or more (per Framingham risk score), or age 75 years or older. Black race was a prespecified variable for the subgroup analyses, and 2802 Black individuals (including both Hispanic and non-Hispanic individuals who self-identified as Black^24^) were enrolled in the trial. Race was self-identified and was included in this study because it was a prespecified variable in SPRINT.

 Key exclusion criteria included a diagnosis of diabetes, a history of stroke, symptomatic heart failure within the past 6 months or left ventricular ejection fraction less than 35%, proteinuria (>1 g per day), end-stage kidney disease, or an estimated glomerular filtration rate (eGFR) of less than 20 mL/min/1.73 m^2^. The study was approved by institutional review boards at each clinical site, and all patients provided written informed consent.

SPRINT was stopped early (median follow-up of 3.26 years) because of an approximately 30% relative risk reduction in CVD events and 25% relative risk reduction in mortality among the intensive BP control group compared with the standard BP control group.^18^ The SPRINT protocol can be found in Supplement 1, and other details regarding the SPRINT study can be found elsewhere.^18,24^ Data for these analyses were obtained through National Heart, Lung, and Blood Institute BioLINCC data repository.

**Figure 1.**
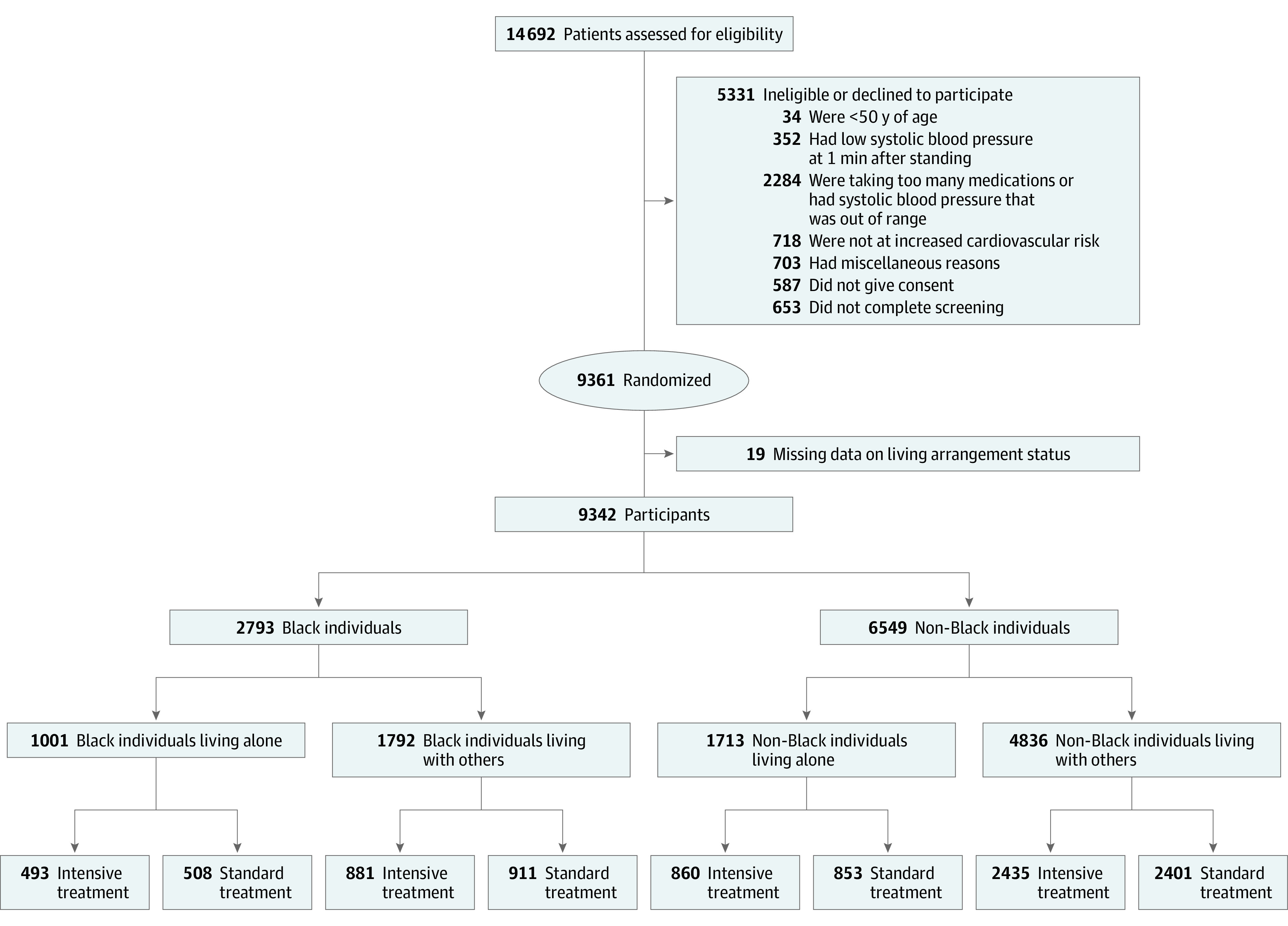
The Flow of the Study Sample Non-Black individuals indicate those who identified as Alaskan Native, American Indian, Asian, Native Hawaiian, Pacific Islander, White, or other during the Systolic Blood Pressure Intervention Trial.

We excluded 19 participants with missing information on living arrangement status resulting in a final analytical sample of 9342 participants. This study cohort was categorized into the following 4 groups based on Black race and their living arrangement status reported by participants (the questionnaires and responses are described in eTable 1 in Supplement 2): Black individuals living alone (n = 1001), Black individuals living with 1 or more other adults (n = 1792), non-Black individuals living alone (n = 1713), and non-Black individuals living with 1 or more other adults (n = 4836). A flow of sample selection in the present study is provided in Figure 1.

### Intervention and Other Measurements

In SPRINT, the participants were randomly assigned to either the intervention group (systolic BP target of less than 120 mm Hg) or the control group (systolic BP target of less than 140 mm Hg) at a 1:1 ratio.^18,24^ BP was measured using an automated measurement system during an office visit with the patient in a seated position after 5 minutes of quiet rest. Antihypertensive medications were adjusted to achieve the systolic BP targets in each group.

Sociodemographic and lifestyle characteristics including age (years), sex (male, female), education status (less than college, college or above), insurance status (private, public, uninsured), smoking status (never, former, current), and alcohol intake (yes, no) were collected by self-report at baseline. Clinical and laboratory information (ie, blood pressure, body mass index [BMI], eGFR, fasting glucose levels, total cholesterol levels, high-density lipoprotein cholesterol levels, triglyceride levels, previous history of CVD, statin use, number of antihypertensives use, and 10-year Framingham CVD risk score) were also collected by trained study personnel according to the standardized protocols.^24^

### Outcomes

The primary outcome of SPRINT was the incidence of a composite end point of myocardial infarction, acute coronary syndrome, stroke, acute decompensated heart failure, or CVD death by August 20, 2015. The secondary outcomes included: (1) all-cause mortality; and (2) composite of the primary outcome and all-cause mortality. Self-reported study outcomes were ascertained by trained staff at each clinical site using structured interviews. Medical records and other corroborating data were also collected. All study outcomes were reviewed and adjudicated by the SPRINT Clinical Outcomes Adjudicator Subcommittee, who were blinded to treatment assignment using a prespecified protocol.^24^

### Serious Adverse Events

The occurrence of serious adverse events (SAEs) was defined as fatal or life-threatening events resulting in death, persistent disability, or hospitalization or prolongation of hospitalization.^18,24^ They included hypotension, syncope, bradycardia, electrolyte abnormalities, injurious fall, and acute kidney injury or acute kidney failure.

### Statistical Analysis

After describing baseline characteristics and trends in systolic BP and the number of antihypertensive medications during the follow-up, we used Cox proportional hazard models to estimate the hazard ratio (HR) of the primary and secondary outcomes according to intensive vs standard BP control in each group (ie, Black individuals living alone, Black individuals living with others, non-Black individuals living alone, and non-Black individuals living with others). To account for the potential imbalance of baseline characteristics in each subgroup of this RCT, we reanalyzed the data adjusting for sociodemographic characteristics, lifestyle, biomarkers, comorbidities, and medications.

Then, we applied the transportability formula to estimate the HRs for these outcomes across the hypothesized target populations with different proportions (0, 20%, 40%, 60%, 80%, and 100%) of Black race and living arrangement status, which were derived from the entire SPRINT study samples. The proportion of other variables was considered consistent with the original trial. A transportability formula is a statistical approach that allows us to extrapolate the results from the SPRINT trial to a target population in which an intervention (intensive BP control) is considered, using the information on outcomes from the original study participants and difference in the distribution of baseline characteristics between the study sample and the target population.^20,21,22,23^ Briefly, we applied the weights created by the inverse of odds of being in the SPRINT as opposed to the target population so that we could emulate the hypothesized target population from the original SPRINT participants and estimate the intervention effects among the target population of interest. More detailed information on this approach including required assumptions appears in eAppendix in Supplement 2 and elsewhere.^20,21,22,23^ The 95% CIs were calculated by repeating the analyses on 5000 bootstrapped samples.

Lastly, given the possible increased risk of SAEs because of intensive BP control,^18^ we compared the occurrence of SAEs across each group by Black race and living arrangement status. All statistical tests were 2-sided and *P* < .05 denoted statistical significance. Analyses were performed with R version 4.0.2 and STATA version 16. Data were collected beween November 2010 and March 2013 and analyzed from January 2021 to October 2021.

## Results

Of 9342 participants, the mean (SD) age was 67.9 (9.4) years, 2793 participants [30%] were Black, 2714 [29%] lived alone, and 3320 (35.5%) were female. Baseline characteristics of the study population according to living arrangement status among Black and non-Black individuals are shown in the Table. Among both Black and non-Black individuals, individuals living alone were more likely to be female and have public insurance coverage compared with those living with others. Clinical background and medication use were similar between Black individuals living alone and those living with others. Baseline characteristics were generally balanced between the intensive and standard treatment groups. During the follow-up, no differences in trends were observed in systolic BP and the number of antihypertensive medications according to living arrangement status in both the standard and intensive treatment group among Black and non-Black individuals (eFigure 1 in Supplement 2).

**Table.** Baseline Characteristics of the Study Participants According to Living Arrangement Status (Living Alone vs Living With Others) and Treatment Assignment (Intensive vs Standard Blood Pressure Control) Among Black and Non-Black Individuals^**a**^

Variables	Treatment assignment of Black participants, No (%)				Treatment assignment of non-Black participants, No (%)^a^			
	Living alone (N = 1001)		Living with others (N = 1792)		Living alone (N = 1713)		Living with others (N = 4836)	
	Intensive (N = 493)	Standard (N = 508)	Intensive (N = 881)	Standard (N = 911)	Intensive (N = 860)	Standard (N = 853)	Intensive (N = 2435)	Standard (N = 2401)
Sex								
Female	255 (51.7)	257 (50.6)	372 (42.2)	382 (41.9)	414 (48.1)	389 (45.6)	636 (26.1)	615 (25.6)
Male	238 (48.3)	251 (49.4)	509 (57.8)	529 (58.1)	446 (51.9)	464 (54.4)	1799 (73.9)	1786 (74.4)
Age, mean (SD), y	65.3 (9.1)	65.4 (9.4)	63.5 (8.8)	63.7 (8.7)	71.7 (9.2)	71.9 (9.3)	68.7 (8.9)	68.5 (9.1)
Education status								
Less than college	384 (77.9)	386 (76.0)	683 (77.5)	678 (74.4)	469 (54.5)	470 (55.1)	1295 (53.2)	1244 (51.8)
College or above	109 (22.1)	109 (24.0)	198 (22.5)	233 (25.6)	391 (45.5)	383 (44.9)	1140 (46.8)	1157 (48.2)
Insurance								
Private	83 (16.9)	97 (19.2)	245 (27.8)	226 (24.8)	128 (14.9)	114 (13.4)	583 (24.0)	603 (25.1)
Public	319 (65.0)	323 (64.0)	463 (52.6)	514 (56.5)	673 (78.3)	678 (79.7)	1685 (69.2)	1624 (67.7)
Uninsured	89 (18.1)	85 (16.8)	172 (19.6)	170 (18.7)	59 (6.9)	59 (6.9)	166 (6.8)	173 (7.2)
Smoking status								
Never	205 (42.1)	216 (43.1)	396 (45.5)	402 (44.5)	379 (44.6)	394 (46.9)	1073 (44.5)	1056 (44.2)
Current	118 (24.2)	119 (23.8)	206 (23.7)	196 (21.7)	91 (10.7)	77 (9.2)	224 (9.3)	209 (8.8)
Former	164 (33.7)	166 (33.1)	268 (30.8)	305 (33.8)	379 (44.6)	370 (44.0)	1117 (46.3)	1123 (47.0)
Alcohol intake	271 (55.2)	297 (58.8)	475 (54.3)	500 (55.2)	592 (69.0)	573 (69.5)	1641 (67.6)	1645 (68.6)
Baseline blood pressure, mean (SD), mm Hg								
Systolic	140.0 (16.7)	139.4 (16.5)	139.2 (16.8)	140.4 (15.4)	139.9 (15.4)	141.5 (16.2)	139.7 (15.3)	138.8 (14.8)
Diastolic	80.6 (12.8)	80.4 (12.4)	81.5 (12.4)	81.8 (12.2)	75.6 (11.3)	76.2 (12.0)	77.5 (11.4)	76.8 (11.4)
Clinical cardiovascular disease	50 (10.1)	57 (11.2)	85 (9.7)	87 (9.6)	139 (16.2)	143 (16.8)	465 (19.1)	433 (18.0)
Subclinical cardiovascular disease	46 (9.3)	37 (7.3)	75 (8.5)	82 (9.0)	60 (7.0)	66 (7.7)	199 (8.2)	208 (8.7)
Statin use	162 (33.2)	181 (35.9)	268 (30.5)	332 (36.6)	377 (44.0)	380 (44.9)	1171 (48.4)	1182 (49.6)
Aspirin use	203 (41.2)	215 (42.4)	359 (40.9)	372 (40.9)	499 (58.1)	413 (48.5)	1345 (55.3)	1348 (56.3)
No. of antihypertensive medications								
0	39 (7.9)	30 (5.9)	69 (7.8)	68 (7.5)	77 (9.0)	116 (13.6)	238 (9.8)	229 (9.5)
1	131 (26.6)	157 (30.9)	236 (26.8)	258 (28.3)	289 (33.6)	259 (30.4)	810 (33.3)	799 (33.3)
2	163 (33.1)	188 (37.0)	323 (36.7)	301 (33.0)	291 (33.8)	292 (34.2)	832 (34.2)	800 (33.3)
≥3	160 (32.4)	133 (26.2)	253 (28.7)	284 (31.2)	203 (23.6)	186 (21.8)	555 (22.8)	573 (23.9)
10-y Framingham cardiovascular disease risk %, median (IQR)	14.8 (11.3)	14.7 (12.6)	14.9 (11.3)	15.2 (12.3)	18.0 (15.9)	19.1 (15.1)	19.3 (13.4)	19.1 (13.9)

^a^ Non-Black individuals indicate those who identified as Alaskan Native, American Indian, Asian, Native Hawaiian, Pacific Islander, White, or other during the Systolic Blood Pressure Intervention Trial.

### Cardiovascular Events and All-Cause Mortality

**Figure 2.**
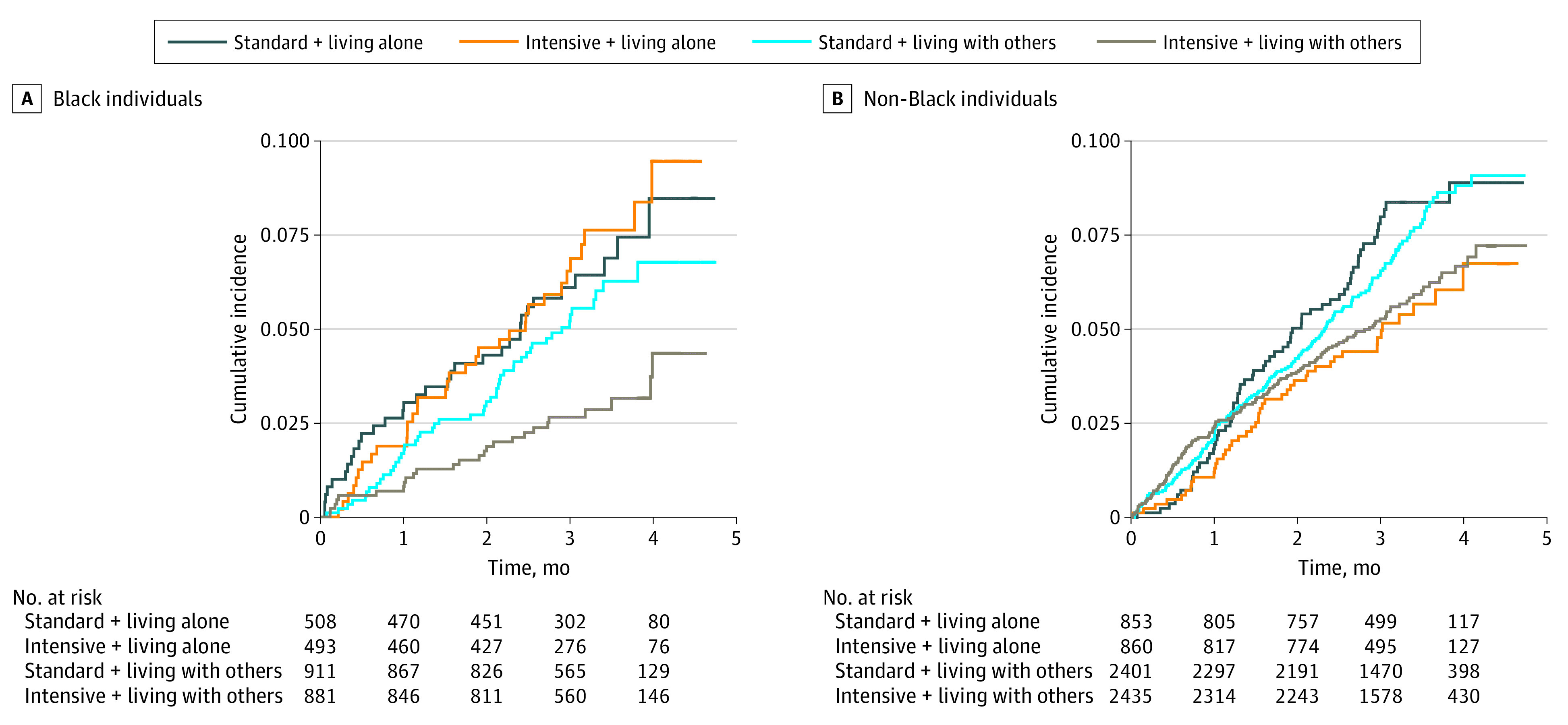
Cumulative Incidence Curves for Cardiovascular Outcomes According to Living Arrangement Status (Living Alone vs Living With Others) and Treatment Assignment (Intensive vs Standard Blood Pressure Control) Among Black Individuals and Non-Black Individuals Non-Black individuals indicate those who identified as Alaskan Native, American Indian, Asian, Native Hawaiian, Pacific Islander, White, or other during the Systolic Blood Pressure Intervention Trial.

**Figure 3.**
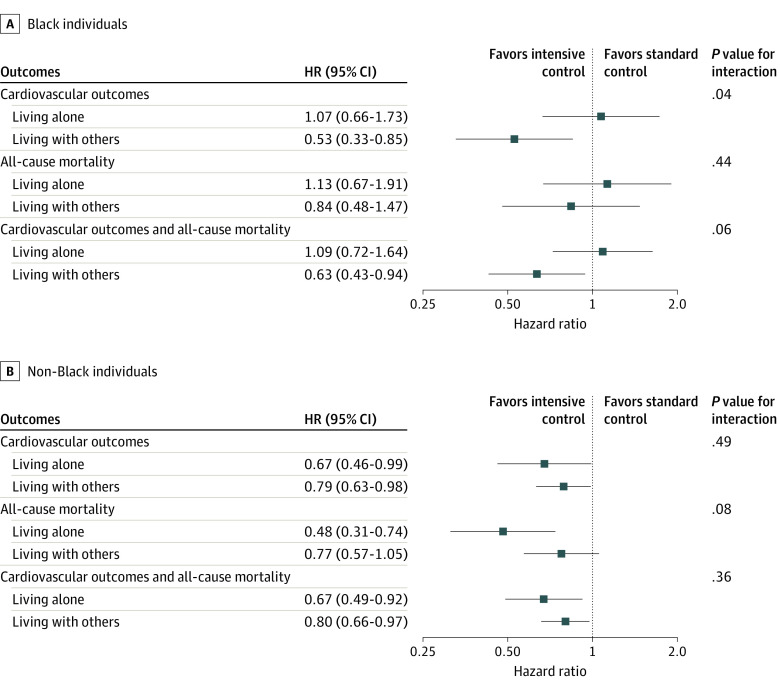
Effects of Intensive Blood Pressure Control on Cardiovascular Outcomes, All-Cause Mortality, and Composite of Cardiovascular Outcomes and All-Cause Mortality According to Living Arrangement Among Black and Non-Black Individuals Non-Black individuals indicate those who identified as Alaskan Native, American Indian, Asian, Native Hawaiian, Pacific Islander, White, or other during the Systolic Blood Pressure Intervention Trial.

Kaplan-Meier survival curves for the primary and secondary outcomes according to living arrangement status and treatment assignment are shown in Figure 2. Over a median (IQR) follow-up of 3.22 (2.74-3.76) years, the primary composite cardiovascular outcome was observed in 67 of 1001 Black individuals living alone (6.7%), 76 of 1792 Black individuals living with others (4.2%), 108 of 1713 non-Black individuals living alone (6.3%), and 311 of 4836 non-Black individuals living with others (6.4%). We found an association between intensive BP control and lower rates of the cardiovascular outcome among Black individuals living with others (HR, 0.53 [95% CI, 0.33-0.85]) but not among those living alone (HR, 1.07 [95% CI, 0.66-1.73]) (*P* for interaction = .04) (Figure 3). We did not find an association between intensive BP control and all-cause mortality among either Black individuals living alone or those living with others, although results for the secondary composite outcomes tended to be consistent with the results for the primary outcomes (Black individuals living alone: HR, 1.09 [95% CI, 0.72-1.64]; those living with others: HR, 0.63 [95% CI, 0.43-0.94]; living arrangement: *P* for interaction = .06). Among non-Black individuals, we found the association of intensive BP control with these outcomes regardless of living arrangement status (Figure 3). The results did not qualitatively change when we reanalyzed the data adjusting for baseline characteristics (Black individuals living alone: HR, 1.02 [95% CI, 0.62-1.66]; those living with others: HR, 0.55 [95% CI, 0.34-0.88]; living arrangement, *P* for interaction = .08; eFigure 2 in Supplement 2).

**Figure 4.**
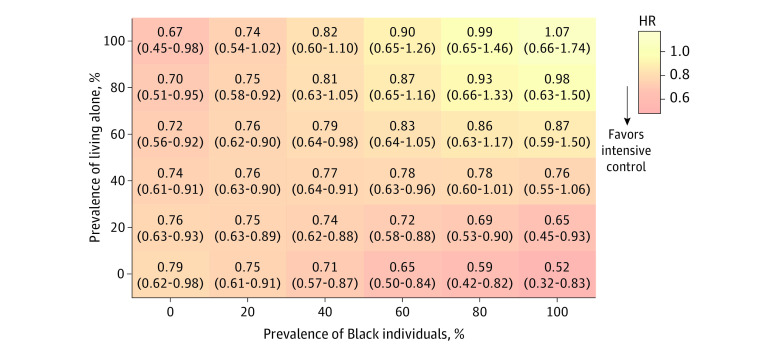
Effects of Intensive Blood Pressure Control (vs Standard Control) on Cardiovascular Outcomes Among the Hypothetical Population by Varying the Distribution of Black Individuals and People Living Alone The 95% CIs for each estimate were calculated by 5000 bootstrapped samples.

Varying the distribution of Black race and living arrangements simultaneously, we found the lower rates of both primary and secondary outcomes associated with intensive BP control across most of the hypothetical populations derived from the entire SPRINT data. However, we did not find the association of intensive BP control with these outcomes among the hypothetical population with both 60% or more of Black individuals and 60% or more of individuals living alone (Figure 4; eFigure 3 in Supplement 2). For example, among a population comprising 80% Black individuals and 60% individuals living alone, the HR for the primary outcomes was 0.86 (95% CI, 0.63-1.17).

### Serious Adverse Events

SAEs occurred in 369 of 1001 Black individuals living alone (37%), 620 of 1792 Black individuals living with others (35%), 697 of 1713 non-Black individuals living alone (41%), and 1841 of 4836 non-Black individuals living with others (38%) (eTable 2 in Supplement 2). We found an increased risk of SAEs by intensive BP control among non-Black individuals living with others but not among other subgroups.

## Discussion

In this secondary analysis of SPRINT, significantly lower rates of cardiovascular events and mortality associated with intensive BP control were found among cohabitating Black individuals as well as non-Black individuals but not among Black individuals living alone. When we extrapolated the SPRINT findings to hypothetical external populations, we found that the association between intensive BP control and these outcomes was decreased or diminished across hypothetical populations with a higher prevalence of Black individuals and a higher prevalence of people living alone. These findings highlight the possible role of living arrangement status in hypertension and CVD management as one of the social determinants of health for the Black population.

To the best of our knowledge, this is the first study showing the difference in the association of intensive BP control with cardiovascular health by living arrangement status (ie, living alone or living with others) among Black individuals and non-Black individuals. Racial disparities in cardiovascular health, particularly social and structural barriers for Black individuals, have been one of the major public health concerns in the United States for years.^25^ Ample evidence has indicated the challenges in CVD prevention and management due to the combination of these barriers.^25,26,27^ Over the last several decades, the prevalence of adults 50 years old or older living alone is rapidly increasing among Black individuals with a larger increase than White individuals.^13^ Given that living alone is a known strong risk for social isolation and the absence of instrumental, emotional, and informational supports at home, such a trend raises concern about its potential effect on their cardiovascular health and increasing the racial disparities. Previous studies have reported the increased risk of cardiovascular and all-cause mortality not only by social isolation, but living alone itself.^14,15,16^ The original SPRINT study and the post hoc analysis found no evidence of heterogeneity in the treatment effect by Black race for the primary outcome (Black: HR, 0.77 [95% CI, 0.55 to 1.06]; non-Black; HR, 0.74 [95% CI, 0.61 to 0.90]; *P* for interaction = .83).^18,28^ Our study substantially extends these results by revealing the underlying heterogeneity by living arrangement status specific to Black individuals. To achieve the American Heart Association 2030 Impact Goal (ie, equitably increase healthy life expectancy from 66 to at least 68 years in the United States),^29^ our findings, along with the previous evidence about the association between living alone and CVD risks,^11^ suggest that living arrangement status needs to be carefully considered when intensively controlling BP to improve cardiovascular health for Black individuals.

Although the underlying mechanisms of the observed heterogeneity by living arrangement among the Black population are unclear, the lack of opportunities to receive social support from their family members or other adults may (at least partially) explain the null findings for cardiovascular outcomes among Black individuals living alone. Social support is associated with CVD risks through both behavioral and physiological pathways.^30^ In addition, socially isolated individuals are known to have poor adherence to prescribed medication,^17^ and such associations may vary across race or ethnicity groups.^31^ A previous study has shown that family emotional involvement and family cohesion contributed to the benefit of a weight loss intervention among Black individuals but not among White individuals.^32^ Given that Black individuals may be more likely to rely on small networks with primary sources of support from family members compared with White individuals in the United States,^33^ they may have a higher chance of being socially isolated by living alone and may have failed to receive enough support including monitoring and emotional care during the trial. Physiologically, social isolation or loneliness may induce activation of the hypothalamic-pituitary-adrenal axis and the sympathetic nervous system, inflammation, and vascular oxidative stress,^34,35^ and these multifactorial mechanisms impairing vasodilation and exaggerating vasoconstriction play critical roles for the elevated CVD burden among Black individuals.^36^ Such allostatic load because of social isolation and cumulative stress of living in a race-conscious society over time^37^ might also contribute to the null findings among Black individuals living alone in our study.

Our application of transportability allowed us to evaluate the association of intensive BP control with cardiovascular outcomes across populations with different prevalence of Black individuals and people living alone. As it is not practical to repeat such large RCTs in a different population, there has been a longstanding debate about how to interpret the results of SPRINT in the real-world population. Although previous studies have tried to generalize their findings to US general populations through the simple comparison of baseline characteristics^38^ or to another cohort through creating prediction models (with c-statistics approximately equal to 0.7),^39^ robust strategies that clinicians, researchers, and policymakers can use to generalize the findings of this RCT have not been well-established so far. Given that we can easily apply the transportability formula through inverse-probability weighting—common statistical modeling—to create pseudo-population that mirror the target population,^21,22,23^ this approach would be a fundamental, flexible, and powerful tool to increase the generalizability of RCT results if the required causal assumptions hold (eAppendix in Supplement 2). For example, we did not find the association between intensive BP control and cardiovascular outcomes among a population comprising 80% Black individuals and 60% individuals living alone. Such information would facilitate the discussion about the importance of careful monitoring and potential intervention to prevent the rapid increase in single person household^12^ in a city with a high proportion of Black individuals such as Detroit, Michigan (78.3% Black individuals in 2021^40^).

### Limitations

This study had limitations. First, we cannot rule out the possibility of misclassification because living arrangement status was self-reported. Second, living arrangement status was measured at baseline, and our study did not take account of the status during the follow-up period. Third, the SPRINT data had the information on whether participants lived with 1 or more other adults or not, but not on who they were living with. We also did not have information on other factors related to social isolation, such as marital status (only available for 20% of the entire SPRINT cohort) and social participation (not available). Therefore, our findings do not represent the potential role of people with who they were living, residential instability, and overall social isolation in hypertension management among the Black population. Fourth, although SPRINT was designed to enhance recruitment of the prespecified subgroup of Black individuals, randomization was not predetermined by living arrangement, and thus our post hoc analyses were not sufficient to establish the causality. In our sensitivity analysis, however, the results did not change qualitatively even after the adjustment of baseline characteristics. Fifth, it is important to note that our transportability formula does not allow us to extrapolate the results to populations outside of the SPRINT eligibility criteria (eg, people with low risk of CVD or people with diabetes). Future investigations focusing on evaluating the possible influence of living alone on hypertension management among the Black population would be needed to validate our findings, identify the underlying mechanisms, and design effective policy or clinical interventions targeting such populations.

## Conclusions

The findings of this post hoc analysis of the SPRINT trial suggest that among Black individuals living alone, intensive BP control (ie, treating to a systolic BP target of <120 mm Hg) was not associated with lower rates of fatal and nonfatal major cardiovascular events and all-cause mortality compared with standard BP control (ie, treating to a systolic BP target of <140 mm Hg), while we found significantly lower rates of these outcomes among Black individuals living with others and non-Black individuals regardless of their living arrangement status. Our transportability analysis suggested that the benefit of intensive BP control might not be expected when we target the population with a very high prevalence of Black individuals and people living alone. These findings generate a hypothesis that the beneficial effect of intensive BP control may be modified by living arrangement status—a key component of social isolation—in the Black population through social differences in treatment and support systems by race or ethnicity groups in the US.

## Notes

Supplement 1.
Trial Protocol

Supplement 2. **eAppendix.** Transportability Approach to Extend SPRINT Findings to the External Target Populations **eFigure 1.** Trends in Systolic Blood Pressure and Number of Antihypertensive Medications According to the Living Arrangement Status and Treatment Assignment Among Black Individuals and Non-Black Individuals **eFigure 2.** Association of Intensive Blood Pressure Control With Cardiovascular Outcomes, All-Cause Mortality, and Composite Of Cardiovascular Outcomes And All-Cause Mortality According to Living Arrangement Among Black Individuals and Non-Black Individuals Adjusting For Baseline Characteristics **eFigure 3.** Association of Intensive Blood Pressure Control (vs Standard Control) With the Secondary Outcomes Among the Hypothetical Population by Varying the Distribution of Black Individuals and People Living Alone **eTable 1.** Questionnaires About Race and Living Arrangement in SPRINT **eTable 2.** Distribution of Incident Serious Adverse Events According to the Living Arrangement Status and Treatment Assignment Among Black Individuals and Non-Black Individuals

Supplement 3.
Data Sharing Statement

## References

[1] Facts About Hypertension. Centers for Disease Control and Prevention. Published September 8, 2020. Accessed April 20, 2021. https://www.cdc.gov/bloodpressure/facts.htm

[2] Forouzanfar MH, Liu P, Roth GA, . Global burden of hypertension and systolic blood pressure of at least 110 to 115 mm Hg, 1990-2015. JAMA. 2017;317(2):165-182. doi:10.1001/jama.2016.19043 28097354

[3] Muntner P, Carey RM, Gidding S, . Potential US population impact of the 2017 ACC/AHA high blood pressure guideline. Circulation. 2018;137(2):109-118. doi:10.1161/CIRCULATIONAHA.117.032582 29133599 PMC5873602

[4] Fryar CD, Ostchega Y, Hales CM, Zhang G, Kruszon-Moran D. Hypertension prevalence and control among adults: United States, 2015-2016. NCHS Data Brief. 2017;(289):1-8. 29155682

[5] Benjamin EJ, Muntner P, Alonso A, ; American Heart Association Council on Epidemiology and Prevention Statistics Committee and Stroke Statistics Subcommittee. Heart disease and stroke statistics-2019 update: a report from the American Heart Association. Circulation. 2019;139(10):e56-e528. doi:10.1161/CIR.0000000000000659 30700139

[6] Rethy L, Shah NS, Paparello JJ, Lloyd-Jones DM, Khan SS. Trends in hypertension-related cardiovascular mortality in the United States, 2000 to 2018. Hypertension. 2020;76(3):e23-e25. doi:10.1161/HYPERTENSIONAHA.120.15153 32654559 PMC9390965

[7] Kaufman JS, Dolman L, Rushani D, Cooper RS. The contribution of genomic research to explaining racial disparities in cardiovascular disease: a systematic review. Am J Epidemiol. 2015;181(7):464-472. doi:10.1093/aje/kwu319 25731887

[8] Mueller M, Purnell TS, Mensah GA, Cooper LA. Reducing racial and ethnic disparities in hypertension prevention and control: what will it take to translate research into practice and policy? Am J Hypertens. 2015;28(6):699-716. doi:10.1093/ajh/hpu233 25498998 PMC4447820

[9] Muntner P, Abdalla M, Correa A, . Hypertension in Blacks. Hypertension. 2017;69(5):761-769. doi:10.1161/HYPERTENSIONAHA.117.09061 28320850 PMC5472537

[10] Williams RB, Barefoot JC, Califf RM, . Prognostic importance of social and economic resources among medically treated patients with angiographically documented coronary artery disease. JAMA. 1992;267(4):520-524. doi:10.1001/jama.1992.03480040068032 1729574

[11] Valtorta NK, Kanaan M, Gilbody S, Ronzi S, Hanratty B. Loneliness and social isolation as risk factors for coronary heart disease and stroke: systematic review and meta-analysis of longitudinal observational studies. Heart. 2016;102(13):1009-1016. doi:10.1136/heartjnl-2015-308790 27091846 PMC4941172

[12] Bureau UC. Historical Census of Housing Tables: Living Alone. The United States Census Bureau. Accessed April 20, 2021. https://www.census.gov/data/tables/time-series/dec/coh-livealone.html

[13] Verdery AM, Margolis R. Projections of White and Black older adults without living kin in the United States, 2015 to 2060. Proc Natl Acad Sci U S A. 2017;114(42):11109-11114. doi:10.1073/pnas.1710341114 28973934 PMC5651770

[14] Case RB, Moss AJ, Case N, McDermott M, Eberly S. Living alone after myocardial infarction: impact on prognosis. JAMA. 1992;267(4):515-519. doi:10.1001/jama.1992.03480040063031 1729573

[15] Udell JA, Steg PG, Scirica BM, ; REduction of Atherothrombosis for Continued Health (REACH) Registry Investigators. Living alone and cardiovascular risk in outpatients at risk of or with atherothrombosis. Arch Intern Med. 2012;172(14):1086-1095. doi:10.1001/archinternmed.2012.2782 22711020

[16] Jensen MT, Marott JL, Holtermann A, Gyntelberg F. Living alone is associated with all-cause and cardiovascular mortality: 32 years of follow-up in the Copenhagen male study. Eur Heart J Qual Care Clin Outcomes. 2019;5(3):208-217. doi:10.1093/ehjqcco/qcz004 30689783

[17] Steptoe A, Shankar A, Demakakos P, Wardle J. Social isolation, loneliness, and all-cause mortality in older men and women. Proc Natl Acad Sci U S A. 2013;110(15):5797-5801. doi:10.1073/pnas.1219686110 23530191 PMC3625264

[18] Wright JT Jr, Williamson JD, Whelton PK, ; SPRINT Research Group. A randomized trial of intensive versus standard blood-pressure control. N Engl J Med. 2015;373(22):2103-2116. doi:10.1056/NEJMoa1511939 26551272 PMC4689591

[19] Lewis CE, Fine LJ, Beddhu S, ; SPRINT Research Group. Final report of a trial of intensive versus standard blood-pressure control. N Engl J Med. 2021;384(20):1921-1930. doi:10.1056/NEJMoa1901281 34010531 PMC9907774

[20] Bareinboim E, Pearl J. A general algorithm for deciding transportability of experimental results. J Causal Inference. 2013;1(1). doi:10.1515/jci-2012-0004

[21] Dahabreh IJ, Robertson SE, Steingrimsson JA, Stuart EA, Hernán MA. Extending inferences from a randomized trial to a new target population. Stat Med. 2020;39(14):1999-2014. doi:10.1002/sim.8426 32253789

[22] Westreich D, Edwards JK, Lesko CR, Stuart E, Cole SR. Transportability of trial results using inverse odds of sampling weights. Am J Epidemiol. 2017;186(8):1010-1014. doi:10.1093/aje/kwx164 28535275 PMC5860052

[23] Inoue K, Hsu W, Arah OA, Prosper AE, Aberle DR, Bui AAT. Generalizability and transportability of the national lung screening trial data: extending trial results to different populations. Cancer Epidemiol Biomarkers Prev. 2021;30(12):2227-2234. doi:10.1158/1055-9965.EPI-21-0585 34548326 PMC8643314

[24] Ambrosius WT, Sink KM, Foy CG, ; SPRINT Study Research Group. The design and rationale of a multicenter clinical trial comparing two strategies for control of systolic blood pressure: the Systolic Blood Pressure Intervention Trial (SPRINT). Clin Trials. 2014;11(5):532-546. doi:10.1177/1740774514537404 24902920 PMC4156910

[25] Carnethon MR, Pu J, Howard G, ; American Heart Association Council on Epidemiology and Prevention; Council on Cardiovascular Disease in the Young; Council on Cardiovascular and Stroke Nursing; Council on Clinical Cardiology; Council on Functional Genomics and Translational Biology; and Stroke Council. Cardiovascular health in African Americans: a scientific statement from the American Heart Association. Circulation. 2017;136(21):e393-e423. doi:10.1161/CIR.0000000000000534 29061565

[26] Harper S, Lynch J, Smith GD. Social determinants and the decline of cardiovascular diseases: understanding the links. Annu Rev Public Health. 2011;32:39-69. doi:10.1146/annurev-publhealth-031210-101234 21219168

[27] Kreatsoulas C, Anand SS. The impact of social determinants on cardiovascular disease. Can J Cardiol. 2010;26(suppl C):8C-13C. doi:10.1016/S0828-282X(10)71075-8 20847985 PMC2949987

[28] Still CH, Rodriguez CJ, Wright JT Jr, ; SPRINT Writing Group. Clinical outcomes by race and ethnicity in the Systolic Blood Pressure Intervention Trial (SPRINT): a randomized clinical trial. Am J Hypertens. 2017;31(1):97-107. doi:10.1093/ajh/hpx138 28985268 PMC5861531

[29] Angell SY, McConnell MV, Anderson CAM, . The American Heart Association 2030 impact goal: a presidential advisory from the American Heart Association. Circulation. 2020;141(9):e120-e138. doi:10.1161/CIR.0000000000000758 31992057 PMC8690536

[30] Li H, Xia N. The role of oxidative stress in cardiovascular disease caused by social isolation and loneliness. Redox Biol. 2020;37:101585. doi:10.1016/j.redox.2020.101585 32709420 PMC7767744

[31] Miyawaki CE. Association of social isolation and health across different racial and ethnic groups of older Americans. Ageing Soc. 2015;35(10):2201-2228. doi:10.1017/S0144686X14000890 26494934 PMC4610249

[32] Samuel-Hodge CD, Gizlice Z, Cai J, Brantley PJ, Ard JD, Svetkey LP. Family functioning and weight loss in a sample of African Americans and Whites. Ann Behav Med. 2010;40(3):294-301. doi:10.1007/s12160-010-9219-z 20721650 PMC2975766

[33] Ajrouch KJ, Antonucci TC, Janevic MR. Social Networks among Blacks and Whites: the interaction between race and age. J Gerontol B Psychol Sco Soc Sci. 2001;56(2):S112-S118. doi:10.1093/geronb/56.2.S112 11245365

[34] McCabe PM, Gonzales JA, Zaias J, . Social environment influences the progression of atherosclerosis in the watanabe heritable hyperlipidemic rabbit. Circulation. 2002;105(3):354-359. doi:10.1161/hc0302.102144 11804992

[35] Nation DA, Gonzales JA, Mendez AJ, . The effect of social environment on markers of vascular oxidative stress and inflammation in the Watanabe heritable hyperlipidemic rabbit. Psychosom Med. 2008;70(3):269-275. doi:10.1097/PSY.0b013e3181646753 18256340

[36] Brothers RM, Fadel PJ, Keller DM. Racial disparities in cardiovascular disease risk: mechanisms of vascular dysfunction. Am J Physiol Heart Circ Physiol. 2019;317(4):H777-H789. doi:10.1152/ajpheart.00126.2019 31397168 PMC6843015

[37] Geronimus AT, Hicken M, Keene D, Bound J. “Weathering” and age patterns of allostatic load scores among blacks and whites in the United States. Am J Public Health. 2006;96(5):826-833. doi:10.2105/AJPH.2004.060749 16380565 PMC1470581

[38] Bress AP, Tanner RM, Hess R, Colantonio LD, Shimbo D, Muntner P. Generalizability of SPRINT results to the U.S. adult population. J Am Coll Cardiol. 2016;67(5):463-472. doi:10.1016/j.jacc.2015.10.037 26562046 PMC5237387

[39] Patel KK, Arnold SV, Chan PS, . Personalizing the intensity of blood pressure control: modeling the heterogeneity of risks and benefits from SPRINT (Systolic Blood Pressure Intervention Trial). Circ Cardiovasc Qual Outcomes. 2017;10(4):e003624. doi:10.1161/CIRCOUTCOMES.117.003624 28373269 PMC5428922

[40] U.S. Census Bureau QuickFacts: United States. Accessed January 6, 2022. https://www.census.gov/quickfacts/fact/table/US/PST045219

