# Supplementary material for: Association of Intensive Blood Pressure Control and Living Arrangement on Cardiovascular Outcomes by Race: Post Hoc Analysis of SPRINT Randomized Clinical Trial
Source: JAMA Netw Open. 2022 Mar 14;5(3):e222037. doi: 10.1001/jamanetworkopen.2022.2037 (PMC9608340; doi:10.1001/jamanetworkopen.2022.2037)
Supplement: Supplement 1. — Trial Protocol [file jamanetwopen-e222037-s001.pdf]

Systolic Blood Pressure Intervention Trial (SPRINT)

Protocol Version 5.0

Table of Contents

Executive Summary...1

Chapter 1 – Introduction and Background...4

Chapter 2 – Overview of Trial Design..13

Chapter 3 – Participant Selection..16

Chapter 4 – Intervention 24

Chapter 5 – Measurements and Follow-up..32

Chapter 6 – SPRINT MIND 39

Chapter 7 – Health-Related Quality of Life and Economic Analyses.........................46

Chapter 8 – Safety Monitoring and Reporting..52

Chapter 9 – Clinical Outcome Measures 56

Chapter 10 – Statistical Considerations...60

Chapter 11 – Data Management 69

Chapter 12 – Quality Control...72

Chapter 13 – Study Organization 78

References – 87

Appendices:

Appendix 1: Abbreviations Used
Appendix 2: Computational Details and Sensitivity Analyses for the CVD outcomes
Appendix 3: Computational Details and Sensitivity Analyses for the MIND outcomes
Appendix 4: SPRINT Organizational Chart
Appendix 5: Membership and Charges of the Committees and Subcommittees
Appendix 6: Participating Sites

SPRINT Protocol
Executive Summary

SPECIAL UPDATE TO PROTOCOL VERSION 5.0:

On August 4, 2015, the SPRINT Data and Safety Monitoring Board (DSMB) recommended unmasking trial investigators and notifying participants of the lower rate of cardiovascular outcomes and total mortality in the intensive arm. The board also recommended developing a transition plan for collecting additional outcome data and for managing study participants' blood pressure. In addition, the DSMB recommended promptly modifying the protocol to reflect the changes required by this early finding of benefit in the intensive arm of the trial.

The NHLBI accepted the DSMB recommendations on August 20, 2015, and asked the SPRINT Steering Committee to rapidly implement these recommendations, including notifying SPRINT staff and study participants. These notifications occurred during the week of September 8-11, 2015, with the goal of informing staff of this news in advance of the participants receiving their letters. Participant letters were mailed on September 8, 2015.

This protocol modification incorporates changes required for discontinuing the blood pressure intervention (see Chapter 4) and outlines the measurements that will be taken at closeout visits (see Chapter 5). The goals are to continue ensuring participant safety while collecting additional outcome data and conducting an orderly trial closeout.

The Systolic Blood Pressure Intervention Trial (SPRINT) is a 2-arm, multicenter, randomized clinical trial designed to test whether a treatment program aimed at reducing systolic blood pressure (SBP) to a lower goal than currently recommended will reduce cardiovascular disease (CVD) risk. About 9250 participants with SBP ≥ 130 mm Hg and at least one additional CVD risk factor will be recruited at approximately 90 clinics within 5 clinical center networks (CCNs) over a 2-year period, and will be followed for 4-6 years. Approximately 4300 participants will have chronic kidney disease (CKD), and 3250 will be aged 75 or older. The primary outcome is the first occurrence of a myocardial infarction (MI), acute coronary syndrome (ACS), stroke, heart failure (HF), or CVD death. Secondary outcomes include all-cause mortality, decline in renal function or development of end stage renal disease (ESRD), dementia, decline in cognitive function, and small vessel cerebral ischemic disease.

Design

SPRINT will randomize about 9250 participants aged ≥ 50 years with SBP ≥130 mm Hg and at least one additional CVD risk factor. The trial will compare the effects of randomization to a treatment program of an intensive SBP goal with randomization to a treatment program of a standard goal. Target SBP goals are <120 vs <140 mm Hg,

respectively, to create a minimum mean difference of 10 mm Hg between the two randomized groups. The primary endpoint is incident CVD events identified over a follow-up period of up to six years. The primary hypothesis is that CVD event rates will be lower in the intensive arm. Both the number of randomizations and the length of follow-up may differ from these targets depending on how observed values of parameters differ from estimates used to design the study. Secondary hypotheses include whether the lower SBP goal reduces CVD event rates and progression of renal disease in people with CKD, whether the lower SBP goal reduces progression of CVD event rates in people aged 75 or older, the impact of treatment strategy on health-related quality of life (HRQL), and the relative cost-effectiveness of the two strategies. Investigation of relevant genetic pathways and other genetic analyses will also be conducted. The sample size of the trial will be enriched by including 4300 persons with CKD (estimated GFR 20-59 ml/min/1.73 m^2) to permit assessment of treatment effect on CVD in this subgroup, as well as on measures of progression of kidney disease. The trial will also include 3250 participants who are 75 years old or older. The SPRINT Memory and cognition IN Decreased hypertension (SPRINT MIND study) will test whether the lower SBP goal influences the rate of incident dementia and mild cognitive impairment, global and domain-specific cognitive function, and small vessel ischemic disease. The sample sizes for each of the three components of the MIND study are different. Incident dementia will be determined in all participants. The rate of non-dementia related cognitive decline in important domains of cognition will be measured in 2800 persons representative of all SPRINT participants and from these 2800 persons the magnetic resonance imaging (MRI) study will involve a sub-set of 640 participants.

Patient population

Although epidemiologic evidence strongly suggests that lowering SBP will reduce CVD risk in nearly all adults, for practical and public health reasons the hypothesis is most efficiently studied in persons with an elevated risk of CVD. Thus, the trial will recruit persons 50 years or older with SBP ≥130 mm Hg and at least one additional CVD risk factor. Three groups will be excluded – patients with diabetes, patients with polycystic kidney disease (PKD), and patients who have had a stroke – because they are the target groups of completed or ongoing trials that are testing a lower BP goal. SPRINT will focus on three high risk groups: patients with clinical CVD other than stroke, patients with chronic kidney disease (estimated glomerular filtration rate [eGFR] 20-59 mL/min/1.73 m^2), and patients who have a Framingham Risk Score (FRS) of ≥15%. A large subgroup will be participants who are 75 years old or older. This trial is expected to enroll 50% women and 40% who are members of minority groups (African Americans, Hispanics, Native Americans, and Asians)

Sample size and power

Based on the Antihypertensive and Lipid-Lowering Treatment to Prevent Heart Attack Trial (ALLHAT) event rates adjusted downward approximately 50% for temporal changes in CVD risk factors and improved therapy, a sample size of 9250 provides approximately 90% power to detect a 20% effect on the primary composite endpoint of CVD mortality and non-fatal MI, ACS, stroke, and heart failure. The annual event rate used in this calculation was 2.2%. Recruitment of a subgroup of 4300 participants with CKD provides 80% power to detect a 20% effect on the same CVD composite endpoint. The probable dementia component of the MIND study will provide 80% power to detect a 15% reduction in the incidence of dementia, 2800 SPRINT-MIND participants will

provide ample power to detect a 20% reduction in the rate of decline in cognitive function between the two arms (more intensive vs. less intensive blood pressure control). In addition, MRI testing to detect differences in small vessel ischemic disease and total brain volume will provide 80% and 90% power, respectively, between the two strategy groups in SPRINT.

Other secondary outcomes

Several additional secondary outcomes will be examined, such as markers of renal function in non-CKD participants, co-morbidities, quality of life, and cost-effectiveness. Adverse events (e.g., postural hypotension, including falls) and biochemical changes will be measured and analyzed by randomized arm.

Chapter 1 – Introduction and Background

1. Background

1.1 Hypertension, public health and the need for a clinical trial testing a lower SBP target.

Elevated blood pressure (BP) is an important public health concern. It is highly prevalent, the prevalence may be increasing, and it is a risk factor for several adverse health outcomes, especially coronary heart disease, stroke, heart failure, chronic kidney disease, and decline in cognitive function. Given the high prevalence and severity of adverse outcomes, even small improvements in the treatment of elevated BP would result in widespread benefit. The benefit of lowering SBP to around 140 mm Hg is well-accepted, but patients treated to this level of BP are still at increased risk of BP-related adverse outcomes. Observational studies document a progressive increase in risk as BP rises above 115/75 mm Hg. Such epidemiologic evidence suggests there may be substantial benefit to targeting treatment to a SBP <120 mm Hg instead of <140 mm Hg. In contrast, targeting to <120 mm Hg may be harmful or unnecessarily costly and burdensome with limited expectation of benefit. Apart from the Action to Control Cardiovascular Risk in Diabetes (ACCORD) trial, which was restricted to participants with diabetes mellitus, no clinical trial has been conducted to test the hypothesis that more intensive reduction in SBP to <120 mm Hg is beneficial compared to the current recommendation of a goal SBP <140 mm Hg. At present, the results from clinical trials that have addressed related hypotheses are ambiguous. A definitive clinical trial testing whether lowering SBP below 120 mm Hg is better than lowering SBP below 140 mm Hg in non-diabetic hypertensive patients is needed, and this has been designated by an NIH Expert Panel as the most important hypothesis to test regarding the prevention of hypertension-related complications (2007).

1.1.1 Prevalence of hypertension

Approximately 1 billion people worldwide have hypertension (HTN) (Kearney and others, 2005). HTN is highly prevalent in the adult population of the US, especially among those aged ≥60 years. Two-thirds of those over age 60 have HTN, and the prevalence has increased in recent decades (Chobanian and others, 2003;Cutler and others, 2008;Hajjar and Kotchen, 2003;Ong and others, 2007;World Health Organization, 2002). By age 50 years, isolated systolic hypertension (ISH) is the most common form of HTN, and is associated with greatest risk of target organ damage and adverse health outcomes (Franklin, 1999;Franklin and others, 2001).

1.1.2 Hypertension as a cardiovascular risk factor

The importance of BP, especially SBP, as an independent risk factor for coronary events, stroke, chronic heart failure (CHF), and ESRD is well documented (Vasan and others, 2001;Collins and others, 1990;Macmahon and others, 1990;Sacco and others, 2001;Jackson, 2000;Staessen and others, 1997;Hsu and others, 2005;Chobanian and others, 2003;Gillum, 1991;Prospective Studies Collaboration, 2002;Levy and others, 1996). There is also substantial epidemiologic and clinical trial evidence supporting a role for hypertension therapy in reducing risk for age-related dementia, including vascular dementia and Alzheimer's dementia (Forette and others, 1998;Luchsinger and

Mayeux, 2004;Reitz and others, 2007;Skoog and Gustafson, 2003;Skoog and others, 2005;Skoog and Gustafson, 2006;Tzourio and others, 2003). Clinical trial data have shown reductions in CVD outcomes, including incident stroke (35% to 40%), MI (15% to 25%), and CHF (up to 50%) (Chobanian and others, 2003;Psaty and others, 1997;Neal, Macmahon, and Chapman, 2000). However, optimal targets for BP lowering are not established.

1.1.3 Support for current target

In addition to the Seventh Report of the Joint National Committee on Prevention, Detection, Evaluation, and Treatment of High Blood Pressure (JNC-7) (Chobanian and others, 2003), most recent practice guidelines recommend a target SBP <140 mm Hg in persons with established uncomplicated hypertension (Campbell and others, 2009;Mancia and others, 2007;Mancia and others, 2009;National Collaborating Centre for Chronic Conditions, 2006;National Heart Foundation of Australia (National Blood Pressure and Vascular Disease Advisory Committee), 2009;Whitworth, 2003). The benefits of lowering high BP in reducing CV morbidity and mortality are well-established (Cutler, MacMahon, and Furberg, 1989;Psaty and others, 1997). A meta-analysis evaluating the treatment efficacy of hypertension therapy in adults over age 60, from three major trials from different countries (Liu and others, 1998;SHEP, 1991;Staessen and others, 1997) found that lowering SBP significantly reduced all-cause and CVD mortality by 17% and 25% respectively, and all CVD end-points by 32% (Staessen and others, 1999;Staessen, Wang, and Thijs, 2001), though both treatment goals and the achieved SBP were >140 mm Hg.

1.1.4 Risk of SBP above normal but below current target

The World Health Organization estimates that about two-thirds of the cerebrovascular disease burden and one-half of the coronary heart disease (CHD) burden on a worldwide basis is attributable to SBP >115 mm Hg (World Health Organization, 2002). Further, SBP > 115 mm Hg has been estimated to account for 7.6 million premature deaths (13.5% of the global total), 92 million disability-adjusted life years (6.0% of the global total), 54% of stroke, and 47% of ischemic heart disease. About half of this burden is in persons with a SBP<145 mm Hg (Lawes, Vander, and Rodgers, 2008). The JNC-7 defined pre-hypertension based on the evidence that SBP values between 120 and 139 mm Hg and diastolic blood pressure (DBP) values between 80 and 89 mm Hg are associated with increased cardiovascular (CV) risk. Although the risk of a BP between 120/80 and 139/89 mm Hg is not as pronounced as that associated with a BP above 140/90 mm Hg (Chobanian and others, 2003), 36% of the adult US population had a BP within this range in the 2007-2008 National Health and Nutrition Examination Survey (Wang and Wang, 2004).

Strong evidence from large population-based longitudinal observational studies indicates that, regardless of other cardiovascular risk factors, SBP levels of about 115 mm Hg in adults over the age of 40 years are associated with lower CVD event rates, including death and slower progression of subclinical CVD (Lewington and others, 2002;Sipahi and others, 2006) compared to higher SBPs. In the Framingham Heart Study (FHS), the risk of CVD following 10 years of follow-up among persons with SBP 130-139 mm Hg and/or DBP 85-89 mm Hg and SBP 120-129 mm Hg and/or diastolic blood pressure (DBP) 80-84 mm Hg was significantly higher when compared to their counterparts with SBP <120 mm Hg and DBP <80 mm Hg (Vasan and others, 2001). Experience in the

Atherosclerosis Risk in Communities (ARIC) and Women's Health Initiative (WHI) studies also showed that individuals with SBP of 120-139 mm Hg and/or DBP of 80-89 mm Hg had an increased risk of CV events, relative to persons with SBP <120 mm Hg (Hsia and others, 2007;Kshirsagar and others, 2006). A large meta-analysis of data from 61 population-based longitudinal epidemiological studies showed a strong continuous graded relationship between SBP and CVD death risk for all age deciles between 40-89 years, independent of other CVD risk factors, beginning at SBP levels of about 115 mm Hg (Lewington and others, 2002). For those aged 40-69 years, there was an approximate doubling in the rates of death from stroke, ischemic heart disease and other vascular causes with each increase of 20 mm Hg in usual (that is, long-term average) SBP.

1.1.5 Evidence for possible benefit of lower target on CV outcomes

Clinical trial evidence of benefit from achieving SBP levels that approach the current recommended goal of <140 mm Hg with pharmacologic treatment is strong, but a trial specifically designed to test lowering the SBP treatment goal below the 140 mm Hg level, the ACCORD trial, found no clear evidence of benefit. The ACCORD trial tested the research question of whether a therapeutic strategy aimed at reducing SBP to <120 mm Hg was more effective in reducing CVD events than a strategy aimed at SBP <140 mm Hg in participants who had diabetes and were at increased risk for CVD events. ACCORD found a non-significant reduction in CV events in the intensively treated group, though a lower than expected event rate contributed to an inability to exclude a clinically meaningful effect (The ACCORD Study Group, 2010). The lack of overall benefit was generally consistent across a variety of subgroups. This is in contrast to prior experience of improved outcomes with more compared to less intensive BP reduction in the diabetic participants in the United Kingdom Prospective Diabetes Study (UKPDS) and in the diabetic subgroups in the Hypertension Optimal Treatment trial (HOT), Systolic Hypertension in the Elderly Program (SHEP) and Systolic Hypertension in Europe trial (Syst-Eur). Importantly, none of these trials tested the same level of intensity of BP reduction or the low BP goal employed in ACCORD. Consistent with previous trials, ACCORD did find a large reduction in the incidence of stroke in the intensively treated group, and though the incidence of serious adverse effects was significantly greater in the intensive treatment group, adverse events occurred with relatively low frequency overall.

Results from overall or subgroup analyses of other CV outcome trials are mixed, with some providing support for the benefit of a lower BP goal but others not providing such evidence. In addition, supportive data from other trials have generally been based on analyses of achieved BP rather than pre-defined treatment goals. For example, the Hypertension Detection and Follow-up Program (HDFP) showed reductions in mortality (17%) and CVD mortality (19%) in participants randomized to Stepped Care treatment of hypertension compared with Referred Care. Participants in the Stepped Care arm averaged 159 mm Hg at baseline and achieved SBP levels of 130 mm Hg at 4 years and 140 mm Hg at 5 years of follow-up (Abernethy and others, 1986;HDFP, 1979b;HDFP, 1979a;HDFP, 1982). In the Heart Outcomes Prevention Evaluation (HOPE) study, the use of ramipril in high-risk patients lowered SBP by 3-4 mm Hg from a baseline mean of 139 mm Hg compared to placebo and reduced the composite CVD endpoint that included CVD death (26%), MI (20%), stroke (32%), revascularization (15%), and CHF (23%) (Yusuf and others, 2000). In the European Trial on Reduction of Cardiac Events with Perindopril in Stable Coronary Artery Disease (EUROPA), use of perindopril (vs.

placebo) resulted in a 5/2 mm Hg reduction in BP (from a mean baseline value of 137/82 mm Hg) and a 20% reduction in CVD events (Fox, 2003). The perindopril protection against recurrent stroke study (PROGRESS) showed a significant reduction in stroke and major vascular events associated with a 9/4 mm Hg reduction in BP from a baseline mean of 147/86 mm Hg (PROGRESS Collaborative Group, 2001). More importantly, in a pre-specified subgroup analysis, those receiving 2 drugs (perindopril plus indapamide) had greater reductions in BP (12/5 mm Hg) and risk (43%) compared with placebo versus those on perindopril alone compared with placebo (5/3 mm Hg and 5%), supporting the hypothesis that lower BP is better. There were similar reductions in the risk of stroke in hypertensive and non-hypertensive subgroups (all $p<0.01$). Finally, in the Comparison of Amlodipine vs. Enalapril to limit Occurrences of Thrombosis trial (CAMELOT), a placebo-controlled trial of patients with heart disease and DBP <100 mm Hg (mean 129/78 mm Hg), amlodipine decreased BP by 4.8/2.5 mm Hg and CVD events by 31% (hazard ratio [HR], 0.69; 95% CI, 0.54-0.88); whereas enalapril lowered BP by 4.9/2.4 mm Hg but did not decrease events (HR, 0.85; 95%CI 0.67-1.07) (Nissen and others, 2004).

Other trials have not supported the hypothesis of benefit from a lower SBP target. In the HOT study, there were no differences in CVD events between groups randomized to target DBPs of ≤90 mm Hg vs ≤85 mm Hg vs ≤80 mm Hg in the entire cohort of 18,790 hypertensive participants; the average on-treatment SBP levels were 140 mm Hg and 144 mm Hg, respectively, in the ≤80 and ≤90 mm Hg target groups (Hansson and others, 1998). Only a post hoc analysis of the diabetic subgroup (n=1,501) showed that major CVD events were reduced by 51% ($p=0.005$) in those randomized to the lower BP goal. The average on-treatment SBP levels were 140 mm Hg and 144 mm Hg in the ≤80 and ≤90 mm Hg target groups, respectively (Hansson and others, 1998). Likewise, there was no special benefit in those with an achieved SBP of 130 mm Hg vs. 134 mm Hg in the Prevention of Events with Angiotensin Converting Enzyme (PEACE) trial, which compared trandolapril treatment to placebo in persons with stable coronary artery disease (Braunwald and others, 2004). In the aggregate, these trials had only modest net reductions in SBP (4-6 mm Hg), though ACCORD and other trials have shown that a much larger reduction (14 mm Hg difference in SBP between the two arms) can be achieved.

The ACCORD BP results provide a strong rationale for testing the potential benefits of intensive BP lowering. (i) The confidence interval around ACCORD's non-significant effect does not exclude benefit in the range of 20% to 25% reduction in the rate of CV events. Effects of that magnitude would be of considerable importance to public health. (ii) Serious adverse effects were significantly more frequent in the intensive treatment group, but occurred with low frequency overall. (iii) People without diabetes, who are probably less prone to microvascular disease but were excluded from ACCORD, may benefit from more intensive BP lowering. (iv) ACCORD excluded people with serum creatinine levels >1.5 mg/dL, which are prevalent in the US population and associated with high CV risk. (v) The glycemia arm of the ACCORD trial was stopped early because of an excess in total mortality and the possibility of interaction between these two interventions is still under investigation. The safety and benefit of intensive BP reduction in patients > age 75 remain to be tested. Thus, it is imperative that the potential benefits and harms of intense SBP-lowering be examined definitively in this and other high-risk populations, e.g. those with chronic kidney disease (CKD) or underlying CVD.

1.1.6 Possible harm from treatment of SBP to <120 mm Hg

There are a number of reasons for requiring recommendations to lower SBP treatment goals be based on definitive trial evidence. Treating to lower BP levels with medications could be harmful. For example, one proposed mechanism that has some support in post hoc analyses of clinical trials (Cruickshank and others, 1987;Cruickshank, 2000;Somes, Shorr, and Pahor, 1999), known as the “J-curve” hypothesis, states that lowering DBP too much may decrease coronary artery perfusion and increase the risk of CVD events in patients with coronary artery disease (CAD). In post-hoc observational analyses of clinical trial experience, the level of DBP below which risk increased has varied by trial, sometimes being as high as <85 mm Hg (Cruickshank and others, 1987). In corresponding analyses of SHEP participants, the higher risk was reported with DBP <55-60 mm Hg during treatment (Somes and others, 1999).

Further, if treatment has little or no benefit, adding drugs is a waste of patients’ and payers’ resources and time. For example, in a cost-effectiveness analysis of the HOT trial, which overall did not show a significant benefit for lower DBP goals, the cost-effectiveness ratios, expressed as cost per year of life gained, were most favorable for the DBP ≤90 mm Hg target group ($4262) and for added aspirin treatment ($12,710) (HOT, 1998). In the moderately intensive treatment (DBP ≤85 mm Hg) group, the cost-effectiveness ratio escalated to $86,360; with intensive treatment (DBP ≤80 mm Hg), costs further increased to $658,370 per year of life gained. Only treatment to a DBP target of 90 mm Hg and co-administering aspirin were considered highly cost effective; intensive BP lowering down to 80 mm Hg was clearly very costly.

A third reason for not recommending lower SBP goals without definitive clinical trial evidence relates to the increased number of drugs required to achieve these goals. For example, in the African American Study of Kidney disease and hypertension (AASK) trial, the intensive BP goal (achieved SBP = 128 mm Hg) group required an average of 3.04 drug classes compared with 2.39 in the conventional BP goal group (Wright, Jr. and others, 2002a) and in the ACCORD BP trial experience >3 drug classes were required for the intensive SBP goal group to achieve a SBP average of 119 mm Hg, compared with 2 classes in the standard SBP goal group with a mean SBP achieved of 134 mm Hg (The ACCORD Study Group, 2010). In addition to being more costly and having greater potential for drug-related adverse events, even 1-2 more medications per day may contribute to reduced adherence to other evidence-based drug treatment (e.g., statins or aspirin). Patients may choose to not take medications without more evidence for safety and benefit. In addition to being more costly, burdensome, and potentially risky, a 20-mm Hg lower SBP goal (and/or a 10 mm Hg lower DBP goal) would likely mean that up to 70-80 million Americans now considered "prehypertensive" may require drug therapy for a condition that has not been proven to be benefited by treatment (Greenlund, Croft, and Mensah, 2004).

Finally, all medications carry an intrinsic risk of side effects which may adversely affect clinical outcomes and quality of life, and lead to drug interactions, especially in older persons who may need to take a variety of medications.

1.1.7 Conclusion

If the SPRINT results are positive and support a SBP goal <120 mm Hg, and this is fully applied in practice a large number of major CVD could be prevented each year, in the

U.S. alone. If the results are negative and SPRINT is sufficiently powered and well-conducted, then recommendations for SBP goal in the treatment of most hypertensive patients, including those with stage 3 CKD and pre-existing CVD, would 1) allow for a redoubled focus on achieving a SBP goal of <140 mm Hg, and 2) abrogate the need for the additional effort and cost of achieving a lower SBP goal than currently recommended for most patients with elevated BP. If none of the major outcomes show harm from lowering to <120, and if any of the outcomes are positive, SPRINT may make a substantial contribution to public health.

1.2 SPRINT's target patient population

Although epidemiologic evidence strongly suggests that lowering SBP will reduce CVD risk in nearly all adults, for practical and public health reasons the hypothesis is most efficiently studied in high-risk individuals. A high risk population stands to benefit most in the sense that a greater number of events may be prevented per treated individual. Furthermore, results in a diverse high risk population will likely generalize to lower risk populations, at least in terms of relative risk reduction. Thus, the SPRINT trial will recruit patients 50 years or older with SBP ≥130 mm Hg who either have or are at high risk for CVD. SPRINT will focus on three high risk groups: individuals with clinical CVD other than stroke, individuals with CKD (estimated glomerular filtration rate [eGFR] 20-59 ml/min/1.73 m^2), and individuals without clinical CVD who have high estimated CVD risk based on factors such as smoking, low levels of HDL, high levels of LDL or age. Three other groups will be excluded: patients with diabetes, patients with polycystic kidney disease (PKD), and patients who have had a stroke. Patients with diabetes have been studied in the ACCORD trial; patients with prior stroke and PKD are part of other ongoing trials.

1.2.1 Chronic Kidney Disease (CKD)

An important and under-studied high-risk group for CVD is the population with CKD (Coca and others, 2006). In the U.S., the number of persons with Stage 3 CKD (eGFR between 30 and 60 ml/min/1.73 m^2) has recently been estimated to be 7.7% of the adult population, or 15.5 million (Coresh and others, 2007). Patients with prevalent CVD have a high prevalence of CKD, with reported ranges of 30-60% (Keeley and others, 2003;Levey and others, 1998;Shlipak and others, 2002).

Individuals with CKD are at high risk for CVD events (Shlipak and others, 2009;Go and others, 2004;Rahman and others, 2006;Weiner and others, 2004;Foster and others, 2007;McCullough and others, 2007;Rashidi and others, 2008;Fried and others, 2009). A meta-analysis of reported data from prospective studies in Western populations demonstrated that people with an eGFR of <60 ml/min/1.73m^2 have a relative risk of 1.4 for CVD, compared to those with an eGFR of ≥60 ml/min/1.73m^2 (Di Angelantonio and others, 2007). The relative risk increases as eGFR declines (Go and others, 2004). Pooled data from the ARIC and CHS cohorts demonstrated that participants with CKD were also at increased risk for stroke (Weiner and others, 2007), and CKD was a risk factor for CVD and all-cause mortality independent of traditional CVD risk factors (Weiner and others, 2004). In ALLHAT, despite exclusion criteria designed to exclude participants with significant GFR impairment, about 18% of participants had an eGFR 30-60 ml/min/1.73m^2. In that CKD subgroup, CHD was 38% higher and combined CVD 35% higher than in those with an eGFR >90 ml/min/1.73m^2 (Rahman and others, 2006).

The effect of BP control on the development of CVD in the CKD population is far less clear (Berl and others, 2005).

A strategy of treating to a lower BP goal may reduce the progression of kidney disease. The risk of CKD increases progressively beginning with pre-HTN levels of BP through the various stages of HTN (Haroun and others, 2003). Several observational studies have suggested that achievement of lower BP is associated with lower risk of adverse kidney outcomes (Bakris and others, 2000;Klag and others, 1996;Schaeffner and others, 2008). However, two randomized clinical trials, AASK and the Modification of Diet in Renal Disease Study (MDRD) that examined lower-than-usual BP goals failed to show an overall significant beneficial long-term effect of lower BP on decline in kidney function (Klahr and others, 1994;Wright, Jr. and others, 2002b) Both studies enrolled participants with non-diabetic CKD and randomized them to a mean arterial pressure (MAP) goal of <92 mm Hg (corresponding to <125/75 mm Hg) or a MAP goal of <107 mm Hg (corresponding to <140/90 mm Hg). The AASK trial compared two BP goals based on MAP (102-107 vs. <92 mm Hg) in 1094 African Americans with hypertensive kidney disease; the achieved difference of 128/78 vs. 141/85 did not reduce the progression of CKD (Wright, Jr. and others, 2002b). However, subgroup analyses of long-term (up to 10 years) post trial follow-up suggested the possibility of benefit in participants with baseline urinary protein excretion equivalent to >300 mg/day who were randomized to the lower goal (Appel and others, 2008). Among 585 non-diabetic participants with Stage 3/4 CKD in MDRD, 24% had PKD and only 53 were African American (Klahr and others, 1994). Mean baseline proteinuria was 2.2 g/d, and a beneficial effect of the lower BP goal on GFR was observed in the subgroup with urinary protein > 1 g/d (Peterson and others, 1995;Sarnak and others, 2005). In addition to the inherent problems associated with subgroup analysis, major caveats of these results from the MDRD Study were that the number of patients in the heavy proteinuric subgroups was small and the results were confounded by the use of angiotensin converting enzyme (ACE) inhibitors. Together, these studies fail to show convincing renoprotective effects for the lower BP goal; however their results have led to clinical recommendations that patients with high levels of proteinuria should have blood pressure goals below 140/90 mm Hg. They were not adequately powered to consider CVD outcomes. Nonetheless, they successfully demonstrated the feasibility of achieving significant separation in BP in large cohorts with advanced CKD. Given the rapid increase in the prevalence of CKD, the effects of aggressively lowering BP on the risks of CVD and CKD progression need to be clarified in a sample that appropriately mirrors the U.S. population with CKD (Sarnak and others, 2003).

1.2.2 SENIOR participants and SPRINT-MIND

Including a large subgroup of participants aged 75+ will provide data on whether intensive BP treatment will reduce CVD and renal events in the elderly. Both the Treatment of Hypertension in Patients over 80 Years of Age (HYVET) (Beckett and others, 2008) and the SHEP (SHEP, 1991) trials found that a SBP delta of 15 and 11 mm Hg, respectively, between treated and placebo groups resulted in >30% reduction in stroke, HF, and overall CVD events in the treated groups. Unlike HYVET and SHEP, which had SBP levels of about 150 and 143 mm Hg at the end of the trials, SPRINT will have a substantially lower SBP target of <120 mm Hg in the intensive treatment group, a goal which has never been tested in the elderly. No previous large scale trial has examined the impact of treating SBP in the elderly to <120 mm Hg versus <140 mm Hg.

Importantly, the elderly pose an additional question as to the safety of intensive SBP lowering in a population with known wider pulse pressures and a risk of excessively low DBP with intensive SBP treatment. In addition to concerns about hypotension, syncope, and falls, there may be a point of maximal benefit beyond which lowering BP could be detrimental in the elderly. This is a specific concern related to very low DBP, which could compromise coronary blood flow. The SPRINT-Senior cohort will allow us to more precisely assess the safety of the lower SBP goal.

The SPRINT Senior cohort also provides a critically important the main body of participants for SPRINT-MIND. Dementia is a leading cause of placement into nursing homes and assisted living facilities (guero-Torres and others, 2001;Guralnik and others, 1997;Magsi and Malloy, 2005;National Institute on Aging, 2000). Dementia affects 24 million individuals globally and 4.5 million persons in the US, a number that is expected to double by 2040 (Ferri and others, 2005;Plassman and others, 2007). Both dementia and a precursor, mild cognitive impairment (MCI), are highly prevalent among adults over age 70, with estimates running between 15-20% and 40-50% respectively in persons over age 80. In addition, there is evidence that MCI is also highly prevalent in persons above age 60 with CKD. Notably, approximately 15% of persons with MCI progress to dementia each year (Petersen, 2000), accruing substantial negative societal impact, and threatening the quality of life of its victims, their families and other caregivers. Proven strategies for prevention and delay of cognitive decline and dementia are lacking, and there is a clear need for clinical trials testing promising preventive interventions. Even a moderately effective strategy could have tremendous benefits, with a 5-year delay in onset of dementia estimated to decrease the number of cases of incident dementia by about 50% after several decades (Brookmeyer and others, 2002).

Cognitive impairment can have multiple etiologies and vascular risk factors are implicated in a large proportion of dementias including neurodegenerative dementias like Alzheimer's type (Qiu, Winblad, and Fratiglioni, 2005c). With this strong link to CVD risk plus several observational studies suggesting that the ideal SBP to lower CVD risk may be below 120 mm Hg (Chobanian and others, 2003) it is possible that targeting intensive blood pressure control intensive blood pressure control may have substantial implications for preserving brain function.

Substantial epidemiologic evidence identifies hypertension as a risk factor for dementia. Longitudinal observational studies have yielded mixed results, depending on the age at which blood pressure is measured, the impact and duration of treatment, duration of hypertension, and level of BP control (Birns and others, 2006;Qiu, Winblad, and Fratiglioni, 2005). Midlife hypertension appears to increase the risk of all-cause dementia in large prospective cohort studies (Freitag and others, 2006;Kivipelto and others, 2001b). However, lower SBP in older adults has been associated with subsequent development of dementia (Nilsson and others, 2007). Clinical trials of antihypertensive treatment have also provided conflicting experience regarding the impact of treatment of hypertension on the risk of cognitive impairment and dementia in older people (Guo and others, 1999; Hajjar and others, 2005; Veld and others, 2001). Four large randomized, placebo-controlled studies have investigated the effects of antihypertensive agents on the incidence of dementia. The Syst-Eur (Staessen and others, 1997) and the Perindopril Protection Against Recurrent Stroke Study (PROGRESS) studies (Tzourio and others, 2003) found that more aggressive antihypertensive treatment reduced the rate of small vessel ischemic disease (also the

primary outcome of SPRINT MIND MRI), a risk factor for dementia (Dufouil and others, 2009), as well as reducing dementia incidence by 50% compared to placebo. In contrast, the Study on Cognition and Prognosis in the Elderly (SCOPE) and SHEP trials (SHEP, 1991) found no significant difference in incidence of dementia between the active treatment and placebo groups, although differential missing data for the placebo vs. treatment groups may explain the SHEP findings (Di Bari and others, 2001). More recently, the HYVET-COG, a BP lowering trial in people age ≥80, was powered to detect a 33% reduction in adjudicated incident dementia (Peters 2008). The trial was stopped prior to its planned date of completion due to significant reductions in stroke and all-cause mortality in the intervention group. It yielded a 14% non-significant reduction in incident dementia. One reason for the non-significant result was a loss of power due to the unexpectedly early conclusion of follow-up, resulting in a relatively short, two-year period of follow-up. One possible explanation for the ambiguous relationships described between hypertension, hypertension treatment and preservation of cognitive function is that the cognitive measures included in most of these trials have not been sensitive enough to detect early, but clinically important, cognitive changes in a cohort with intact general cognitive function at baseline. Studies using more sensitive neuropsychological tests, such as the testing proposed for SPRINT-MIND, have shown the strongest relationships (Elias and others, 1993;Kivipelto and others, 2001a;Kivipelto and others, 2001c;Knopman and others, 2001).

Hypertension is the primary risk factor for small vessel ischemic disease and cortical white matter abnormalities (Basile and others, 2006;Kuller and others, 2010;Liao and others, 1996;Longstreth, Jr. and others, 1996). Chronic kidney disease is also associated with white matter abnormalities (Ikram and others, 2008), thus the SPRINT population is at high risk for significant white matter changes. Longitudinal studies document that hypertension-associated white matter abnormalities are an independent risk factor for cognitive decline and dementia (Verdelho and others, 2007;Vermeer and others, 2003), lower extremity functional abnormalities (Rosano and others, 2005), and clinical stroke (Debette and others, 2010). However, there is limited evidence that better control of BP slows the progression of white matter lesions in the brain (Dufouil and others, 2005). Recently reported results from the Women’s Health Initiative Memory Study (WHIMS) indicate that white matter volume (detected by MRI) is associated with baseline BP, even after adjustment for treatment, other CVD risk factors, and age (Coker L.H. and others, 2008). Although the beneficial effects of treating hypertension on CVD, such as stroke have been shown (Collins and others, 1990), it is not known whether intensive lowering of SBP as proposed in SPRINT will provide reduction in the risk for developing white matter disease and brain volume loss.

SUMMARY

Higher than optimal BP is the leading cause of disability adjusted life-years lost on a global basis, and more intensive control of SBP than is currently recommended may contribute to reductions in stroke, heart failure, coronary heart disease, chronic kidney disease, and dementia. This potential benefit must be weighed against potential risks, including complications resulting from low coronary, cerebral, and renal perfusion pressure and the medications themselves. Definitive evidence from a well designed and conducted trial should form the foundation for pertinent recommendations and healthcare policies.

Chapter 2 – Overview of Trial Design

The SPRINT randomized controlled clinical trial will examine the effect of a high BP treatment strategy aimed at reducing SBP to a lower goal than is currently recommended. The primary objective is to determine whether randomization to this intensive strategy is more effective than a standard strategy in reducing the incidence of serious cardiovascular disease events. Other important study objectives are to assess the impact of more intensive SBP reduction on renal function, incidence of probable dementia, quality of life, cost-effectiveness, cognitive function and small vessel ischemic disease.

The study cohort will include approximately 9250 people aged ≥ 50 years with SBP ≥130 mm Hg. SPRINT will focus on three high risk groups: patients with clinical CVD other than stroke, patients with chronic kidney disease (estimated glomerular filtration rate (eGFR) 20 -59 mL/min/1.73 m^2), and patients who have a Framingham Risk Score (FRS) of ≥15%. Participants will be recruited over a 2-year period at approximately 80 to 100 clinics in 5 clinical center networks (CCNs) and will be followed for up to 6 years. Both the number of randomizations and the length of follow-up may differ from these targets depending on how observed values of parameters differ from estimates used to design the study. Approximately 4300 SPRINT participants will have CKD, and 3250 will be age 75 or older. Chapter 3 presents the eligibility criteria for the trial.

Participants will be stratified by clinic and randomly assigned to either the intensive or standard SBP lowering strategy. Chapter 4 and 5 provides a general description of the intervention.

The primary outcome will be a composite end-point consisting of the first occurrence of a myocardial infarction (MI, by electrocardiogram (ECG) or hospitalization), stroke, heart failure, non-MI acute coronary syndrome, or CVD death. Study outcomes are described in Chapters 6, 7 and 9.

The sample size for SPRINT is estimated to provide 90% power to detect a 20% relative decrease in the rate of the composite primary outcome in participants randomized to the more intensive SBP lowering strategy. Sample size estimation is described further in Chapter 10.

The major objectives of the SPRINT trial are as follows:

2.1 Primary Hypothesis

In people aged ≥ 50 years with SBP ≥130 mm Hg and either a history of CVD, eGFR between 20 and 59, or a Framingham Risk Score (FRS) indicating 10-year CVD risk of ≥15%, does a therapeutic strategy that targets a SBP of < 120 mm Hg reduce the rate of CVD events compared to a strategy that targets a SBP of < 140 mm Hg? This hypothesis will be tested using a composite outcome including

- cardiovascular death,
- myocardial infarction,
- stroke,
- heart failure, and
- non-MI acute coronary syndrome

ascertained over a follow-up period of up to 6 years. Interim monitoring for overall trial efficacy will be based on the accrued rate of this primary outcome. The anticipated event rate for this outcome is 2.2%/year.

2.2 Subgroup Hypotheses

SPRINT will examine intervention effects in a number of subgroups; these are presented in greater detail in Chapter 10. Two subgroups are of particular interest due their connection to possible biological mechanisms affecting the primary outcome:

1. participants with and without CKD (eGFR <60 ml/min/1.73m^2) at baseline,
2. participants < or ≥ 75 years at baseline.

Consistency of the effects for the intervention on the primary outcome will also be examined in subgroups defined by gender, race/ethnicity (black vs. non-black), presence of clinical CVD at baseline (i.e., primary and secondary prevention participants) and tertiles of baseline systolic BP.

Subgroup analyses for secondary outcomes are described in Chapter 10.

2.3 Secondary Hypotheses

SPRINT prespecifies two types of secondary hypotheses. The first type will address secondary outcomes in analyses designed to support and confirm the primary analysis. These will include components of the primary composite outcome, total mortality, and a composite of the primary composite with total mortality (CVD-free survival). The other type addresses two areas of non-cardiovascular clinical effects: renal and cognitive outcomes.

2.3.1 Objectives for renal outcomes and the CKD subgroup

1. For the CKD subgroup, we will determine whether the intensive intervention arm experiences a lower rate of a composite of renal outcomes composed of:
   - ESRD or
   - A 50% decline from baseline eGFR

2. For the non-CKD subgroup, we will determine whether the intensive intervention arm experiences a lower rate of progression to CKD, defined as
   - ESRD or
   - 30% decrease from baseline eGFR and an end value of <60 ml/min/1.73M^2

2.3.2 SPRINT MIND Hypotheses

1. All-cause Dementia. The incidence of all-cause dementia will be lower in SPRINT participants assigned to the intensive SBP treatment arm compared to their counterparts assigned to the standard SBP treatment arm.

2. Cognitive Decline. The combined rate of decline in all domains of cognition will be slower in the intensive SBP treatment arm compared to the standard SBP treatment

arm. This hypothesis will be tested in a randomly selected subset of 2800 participants enrolled in SPRINT.

3. MRI Brain Changes. The volume small vessel ischemic disease (SVID) will be lower in SPRINT participants assigned to the intensive SBP treatment arm compared to their counterparts assigned to the standard SBP treatment arm. A sub-hypothesis is that total brain volume will also be greater (thus less atrophy) in the intensively treated group. The MRI sub-study will be conducted in 640 participants chosen from the 2800 selected to receive regular extensive cognitive assessment.

Chapter 3 – Participant Selection

3.1 Eligibility Criteria

The objective of setting inclusion/exclusion criteria is to identify a trial population that will ensure adequate event rates for statistical power, provide maximum generalizability, and maximize safety. Inclusion/exclusion criteria were made as simple as possible to ensure standard implementation across all SPRINT study sites. Specifically, the SPRINT eligibility criteria were developed to facilitate the identification and inclusion of a trial population at high risk for the major trial endpoints, including CVD, CKD, cognitive decline, and dementia. Hence, the trial population is comprised of individuals in three major classes: those with existing CVD, existing CKD, or an elevated estimated risk for CVD disease based on age and other risk factors.

Implementation of these inclusion and exclusion criteria and related recruitment strategies will be accomplished to meet several goals with respect to composition of the study population. The overall goal for recruitment is 9,250 participants, although the final number of randomizations may be between 8,500 and 10,000. For the target of 9,250 participants, we will strive to include approximately 4300 (46%) with chronic kidney disease (eGFR 20 -59 ml/min/1.73m^2), expected to be divided approximately evenly below and above 45 ml/min/1.73m^2, and approximately 3250 (35%) who are at least 75 years old. In addition, we will strive to include 50% women, 40% minorities, and 40% with clinical or subclinical cardiovascular disease. Among these goals there is an implicit hierarchy based on study hypotheses and design considerations: first, attain the overall sample size, to preserve power for the main hypothesis of SPRINT; second, reach the required sample sizes for formal sub-group hypotheses among participants with CKD and among seniors; and third, ensure a sufficiently diverse study population so that results are broadly applicable to the affected U.S. population. We will monitor these goals on an ongoing basis and the Recruitment, Retention, and Adherence Subcommittee and the Steering Committee will evaluate recruitment strategies and implement corrective actions.

a) Inclusion Criteria

1. At least 50 years old

2. Systolic blood pressure

   SBP: 130 – 180 mm Hg on 0 or 1 medication
   SBP: 130 – 170 mm Hg on up to 2 medications
   SBP: 130 – 160 mm Hg on up to 3 medications
   SBP: 130 – 150 mm Hg on up to 4 medications

3. There are no diastolic blood pressure (DBP) inclusion criteria, since risk is more related to SBP than DBP in the age and risk population anticipated for SPRINT. If a screenee is otherwise eligible for SPRINT but presents with a treated BP and/or number of medications that fall outside the SPRINT inclusion criteria, BP-lowering medications may be adjusted prior to the randomization visit to determine whether, with such adjustments, the screenee will meet eligibility criteria for SPRINT. A screenee who presents on no BP medications should have documentation of SBP

≥130 mm Hg on 2 visits within 3 months prior to the randomization visit in order to be eligible for the trial.

4. Risk (one or more of the following):
    a) Presence of clinical[*] or subclinical[**] cardiovascular disease other than stroke
    b) CKD, defined as eGFR 20 – 59 ml/min/1.73m^2 based on the 4-variable Modification of Diet in Renal Disease (MDRD) equation and latest lab value, within the past 6 months. (If the serum creatinine is unstable within the last 6 months, enrollment into SPRINT could be delayed until the serum creatinine has been stabilized and the eGFR is still within the allowed range.)
    c) Framingham Risk Score for 10-year CVD risk ≥ 15% based on laboratory work done within the past 12 months for lipids
    d) Age ≥ 75 years.

5. Clinical CVD (other than stroke)
    a) Previous myocardial infarction (MI), percutaneous coronary intervention (PCI), coronary artery bypass grafting (CABG), carotid endarterectomy (CE), carotid stenting
    b) Peripheral artery disease (PAD) with revascularization
    c) Acute coronary syndrome with or without resting ECG change, ECG changes on a graded exercise test (GXT), or positive cardiac imaging study
    d) At least a 50% diameter stenosis of a coronary, carotid, or lower extremity artery
    e) Abdominal aortic aneurysm (AAA) ≥5 cm with or without repair

6. Subclinical CVD
    a) Coronary artery calcium score ≧ 400 Agatston units within the past 2 years.
    b) Ankle brachial index (ABI) ≤0.90 within the past 2 years.
    c) Left ventricular hypertrophy (LVH) by ECG (based on computer reading), echocardiogram report, or other cardiac imaging procedure report within the past 2 years.

b) Exclusion Criteria

1. An indication for a specific BP lowering medication (e.g., beta-blocker following acute myocardial infarction) that the person is not taking and the person has not been documented to be intolerant of the medication class. (If a screenee has a non-hypertension indication for a BP-lowering medication (e.g., beta-blocker post-MI, renin angiotensin system (RAS) blocker for CVD prevention, or alpha blocker for benign prostatic hypertrophy (BPH)), the screenee should be on the appropriate dose of such medication before assessing whether he/she meets the SPRINT inclusion criteria. If the investigator believes that a potential participant has such an indication but is not receiving appropriate treatment, he/she should encourage the potential participant's primary care provider to consider placing the patient on the appropriate therapy prior to proceeding with the screening process.)
2. Known secondary cause of hypertension that causes concern regarding safety of the protocol.
3. One minute standing SBP < 110 mm Hg. Not applicable if unable to stand due to wheelchair use.

4. Proteinuria in the following ranges (based on a measurement within the past 6 months)
    (a) 24 hour urinary protein excretion ≥1 g/day, or
    (b) If measurement (a) is not available, then 24 hour urinary albumin excretion ≥ 600 mg/day, or
    (c) If measurements (a) or (b) are not available, then spot urine protein/creatinine ratio ≥ 1 g/g creatinine, or
    (d) If measurements (a), (b), or (c) are not available, then spot urine albumin/creatinine ratio ≥ 600 mg/g creatinine, or
    (e) If measurements (a), (b), (c), or (d) are not available, then urine dipstick ≥ 2+ protein
5. Arm circumference too large or small to allow accurate blood pressure measurement with available devices
6. Diabetes mellitus. Participants taking medications for diabetes at any time in the last 12 months are excluded. Participants are also excluded if there is documentation of: FPG at or above 126 mg/dL, A1C ≥6.5 percent, a two-hour value in an OGTT (2-h PG) at or above 200 mg/dL or a random plasma glucose concentration ≥200 mg/dL. The diagnosis of diabetes must be confirmed on a subsequent day by repeat measurement, repeating the same test for confirmation. However, if two different tests (e.g., FPG and A1C) are available and are concordant for the diagnosis of diabetes, additional testing is not needed. If two different tests are discordant, the test that is diagnostic of diabetes should be repeated to confirm the diagnosis.
7. History of stroke (not CE or stenting)
8. Diagnosis of polycystic kidney disease
9. Glomerulonephritis treated with or likely to be treated with immunosuppressive therapy
10. eGFR < 20 ml/min /1.73m^2 or end-stage renal disease (ESRD)
11. Cardiovascular event or procedure (as defined above as clinical CVD for study entry) or hospitalization for unstable angina within last 3 months
12. Symptomatic heart failure within the past 6 months or left ventricular ejection fraction (by any method) < 35%
13. A medical condition likely to limit survival to less than 3 years, or a cancer diagnosed and treated within the past two years that, in the judgment of clinical study staff, would compromise a participant's ability to comply with the protocol and complete the trial. Exceptions to the exclusion for diagnosed cancer would include, for example, non-melanoma skin cancer, early-stage prostate cancer, localized breast cancer.
14. Any factors judged by the clinic team to be likely to limit adherence to interventions. For example,
    (a) Active alcohol or substance abuse within the last 12 months
    (b) Plans to move outside the clinic catchment area in the next 2 years without the ability to transfer to another SPRINT site, or plans to be out of the study area for more than 3 months in the year following enrollment.
    (c) Significant history of poor compliance with medications or attendance at clinic visits
    (d) Significant concerns about participation in the study from spouse, significant other, or family members
    (e) Lack of support from primary health care provider

(f) Residence too far from the study clinic site such that transportation is a barrier including persons who require transportation assistance provided by the SPRINT clinic funds for screening or randomization visits
(g) Residence in a nursing home. Persons residing in an assisted living or retirement community are eligible if they meet the other criteria.
(h) Clinical diagnosis of dementia, treatment with medications for dementia, or in the judgment of the clinician cognitively unable to follow the protocol
(i) Other medical, psychiatric, or behavioral factors that in the judgment of the Principal Investigator may interfere with study participation or the ability to follow the intervention protocol

15. Failure to obtain informed consent from participant
16. Currently participating in another clinical trial (intervention study). Note: Patient must wait until the completion of his/her activities or the completion of the other trial before being screened for SPRINT.
17. Living in the same household as an already randomized SPRINT participant
18. Any organ transplant
19. Unintentional weight loss > 10% in last 6 months
20. Pregnancy, currently trying to become pregnant, or of child-bearing potential and not using birth control

c) Additional Criteria
I. SENIOR

Whereas there are no eligibility criteria specific to the SENIOR subgroup other than age, the general eligibility criteria were influenced by consideration of factors of importance to the inclusion of older participants in SPRINT, including cognitive status, orthostasis, transportation, and site of residence (e.g., nursing home). The goal is to assemble a representative population of older patients for whom intensive BP lowering is reasonable to consider from a medical perspective. This goal is motivated by the perspective that there may be some older persons with advanced frailty and/or multiple comorbid conditions whose health is so poor that it would not be reasonable to attempt to treat SBP as intensively as needed to control SBP to less than 120 mm Hg.

II. Participants with CKD

For the purposes of SPRINT, qualifying CKD is defined by eGFR, determined during the 6 months prior to randomization, between 20 and 59 ml/min/1.73m^2, inclusive, based on the 4-variable MDRD equation. Patients with significant proteinuria, defined as a 24-hour urine protein excretion exceeding 1 gram, or rough equivalents thereof (see Exclusion Criterion 4 above), will be excluded from SPRINT based on evidence from previous trials suggesting that intensive BP lowering therapy may be beneficial with respect to slowing the progression of CKD. The vast majority of participants with CKD so defined will likely be at high risk for CVD. An estimated 82.3% of those who qualify with eGFR between 45 and 59 ml/min/1.73m^2 will have a Framingham Risk Score for CVD exceeding 15% over 10 years, and an estimated 71.2% have a Framingham Risk Score for CVD exceeding 20% over 10 years; hence, these participants will contribute substantially to the overall event rate and provide the basis for informative subgroup analyses.

III. MIND

Dementia Screening - All individuals will receive dementia screening at baseline and every 2 years following baseline. Individuals who have been previously diagnosed with dementia by their physicians are excluded from SPRINT and SPRINT MIND.

Comprehensive Cognitive Assessment substudy – A subset of 2800 participants enrolled in SPRINT will also be assigned to undergo more extensive cognitive assessment to evaluate the impact of the intervention on decline in overall and domain-specific cognitive function that does not meet criteria for dementia. With limited exceptions, all clinics will enroll participants into this 2800 subset, and this subgroup is expected to be representative of all randomized participants, including the important CKD and SENIOR participants.

IV. MIND MRI

Individuals who enroll in the Comprehensive Cognitive Assessment substudy at a clinic within sufficient proximity to a SPRINT MIND MRI center, generally defined as within a 2 to 3 hour driving radius, are eligible to enroll in the MIND MRI Study. The MIND MRI Study will have a recruitment goal of approximately 640 participants. Standard safety-related exclusions pertaining to the ability to have a magnetic resonance imaging procedure performed will be applied.

Recruitment and risk implications of inclusion and exclusion criteria

As shown in Table 3.1, according to analyses of the National Health and Nutrition Examination Survey (NHANES) data for 1999-2004, approximately 6% of the US population meets the basic eligibility criteria related to age and SBP, and are free of diabetes and previous stroke. Among that group, approximately 70% meet the risk criteria described above. The vast majority of these individuals have an estimated 10-year risk of CVD exceeding 20% and the population average 10-year risk for CVD is approximately 28%. (Note that the use of the FRS in this manner likely underestimates the risk of those individuals with existing CHD and stage 3 CKD.) This analysis provides evidence that the recruitment pool will be large enough to enable us to recruit successfully and to generalize our ultimate results to a reasonably large proportion of the US population.

Table 3.1. Distribution of 10-year risk of CVD in NHANES participants who met basic SPRINT eligibility criteria

Criteria	% of US Population meeting basic eligibility criteria (age, SBP, no DM or stroke)	% of those meeting basic eligibility requirements who meet risk criteria	10-year CVD Risk Distribution (%)				Mean 10-yr CVD risk (%)
			5-10%	10-15%	15-20%	20+%	
CHD or Stage 3 CKD or FR≥15%	6.7	70.3	1.3	3.2	24.3	71.1	28.6

In additional analyses of the NHANES potentially eligible pool, 16.3% had stage 3 CKD (3.6% had eGFR < 45ml/min/1.73m^2), 15.6% had a history of CVD, 34.6% were 75 years old or older, 8.1% were African Americans, and 49.8% were women. Stage 3a

CKD, defined as eGFR 45-59 ml/min/1.73m^2, but a urine albumin-to-creatinine (ACR) ≤ 10 mg/g, comprised 6.1% of the eligible pool. These analyses, shown in Table 3.2, provide evidence to support our recruitment targets for participants with CKD, in the SENIOR population, minorities and women.

Table 3.2. Characteristics of SPRINT eligible sample based on NHANES data. Eligibility requirements include age≥50, SBP≥130, eGFR> 20, ACR<600 mg/g and no history of stroke or diabetes.

Characteristic	Proportion (%)
% Prior CVD	15.6
% CKD	16.3
% Stage 3b CKD	3.6
% Stage 3a + ACR > 10 mg/g	6.6
% Stage 3a + ACR ≤ 10 mg/g	6.1
% Senior (age≥75)	34.6
% Female	49.8
% Black	8.1
% Hispanic	7.4
% SBP 130-139 on no BP lowering medications	15.2
% with FRS < 15% per 10 yrs	4.5

3.2 Recruitment: Informed Consent, Screening, Baseline

Recruitment

The SPRINT recruitment goals are described above. Specific community resources will be used to target women and minority/under-served populations to ensure adequate representation of these groups in SPRINT. Recruitment strategies that have worked well in other trials related to hypertension and CKD will be used. Centralized training for CCN and Clinical Site staffs regarding recruitment issues will be provided before recruitment begins.

The goal of participant recruitment is to create a trial population that will ensure adequate event rates for statistical power while maximizing participant safety and generalizability to the population for which the intervention is intended. A multifaceted approach to screening and enrollment is essential to achieve the recruitment goal. For this multicenter trial, recruitment strategies targeting both existing populations within the clinical practice of the research sites as well as individuals from outside these practice settings will be used to identify potentially eligible participants.

The Recruitment, Retention and Adherence Subcommittee will play a significant role in monitoring the progress of study-wide recruitment and provide a forum for advising the CCNs and clinical sites on problem identification, goal setting, strategy deployment and evaluation in their efforts to achieve site and study-wide recruitment goals. This may include guidance for enhancing the recruitment of ethnic groups, women and the elderly. The Subcommittee will also contribute to the development of the recruitment tools including culture-, gender- and age-specific materials to promote enrollment among these important subgroups.

3.2.1 Regulatory and Ethical Considerations, including the Informed Consent Process

The study will be conducted in accordance with Good Clinical Practice (GCP), all applicable subject privacy requirements, and the guiding principles of Helsinki, including but not limited to:

1. Local Institute Review Board (IRB)/Central IRB review and approval of study protocol and any subsequent amendments.
2. Subject informed consent for main trial, SPRINT MIND, genetic testing, and post trial contact, and any ancillary studies. The study consent will contain the six essential elements from GCP guidelines that include:
    - Research statement, reasonably foreseeable risks or discomforts, reasonably expected benefits to subjects or others, appropriate alternatives, extent of confidentiality, compensation or treatment for injury.
    - Additional elements where appropriate such as unforeseeable risks to subjects, embryos, or fetuses, investigator-initiated termination of participation, additional costs, significant new findings, authorization for release of protected health Information for research purposes.
3. Investigator reporting requirements.

Written informed consent and Health Insurance Portability and Accountability Act (HIPAA) authorization must be obtained from each person prior to enrollment into SPRINT. In collaboration with the CCNs, the SPRINT Coordinating Center will provide full details and template documents for the above procedures in the Manual of Procedures and provide training to the investigators and clinical staff on regulatory and ethical considerations. All study personnel will be responsible for completing and remaining current with all applicable human subjects' protection, good clinical practice and data security and privacy training requirements

3.2.2 Existing Populations in the Clinical Site Practices

Methods for identifying potentially eligible participants within the clinical practice of the research settings may include: a targeted review of medical records or databases for those meeting the trial's inclusion criteria, referrals from providers/employees within the practice and/or from practice participants themselves. Additional approaches may also include written materials such as direct mailing and/or advertisement on such items as appointment reminders.

3.3. Screening Visits/ Baseline Visits

Screening Activity Considerations

Each SPRINT clinical center should consult their local IRB regarding approval requirements to access internal medical record searches for potential SPRINT patients. Depending upon the institution, prior approvals for data transfer agreements may be needed to obtain de-identified patient information. Pursuant to such agreements investigators may be required to sign a privacy agreement to protect the patient's protected health information (PHI) as well as comply with other policies and procedures as defined by the institution's designated privacy, security and compliance services.

SPRINT clinical centers will work with the respective CCNs to complete Health Insurance Portability and Accountability Act (HIPAA) Privacy rule documents, preparatory to research waivers and training prior to patient medical record searches. Once local regulatory requirements have been approved, investigator plans to identify potential study patients may be implemented. Large scale data base searches, stratified by key specified inclusion criteria may also yield a global assessment of the potentially eligible study population. Other study parameters (e.g. age, race, gender CKD status, etc.) can be added to further specify the eligible population.

Prior to conducting prescreening and screening activities, it may also be necessary to request additional approvals beyond the IRB (e.g. physician approval or consultation for a screening referral to the SPRINT clinic). Participant informed consent must also be obtained prior to performing any procedures related to the trial.

Screening Visits/Baseline Visit
The following are key elements of the screening and baseline visits and are outlined in the study assessments and procedures below:

Screening Visit(s)
1. Verify participant's interest in study.
2. Obtain in person study consent and HIPAA authorization for main trial, and if applicable, SPRINT MIND, genetic testing and any ancillary studies
3. Continue collection of screening information, including such items as contact information, additional eligibility information including BP measurement, concomitant medications, and medical history.

Baseline visit (Randomization Visit)
1. Confirmation that all inclusion/exclusion criteria satisfied
2. Verification of participant consent and HIPAA authorization.
3. Verification of participant contact information
4. Obtain a Release of Information, as permitted by local policy, to collect event and serious adverse event (SAE) documentation
5. Completion of the study randomization procedure and baseline data collection, including obtaining BP, ECG, and blood and urine samples for analysis and storage at the central lab

Data obtained from the screening, and randomization visits must be supported in the patient’s source documentation. Visit data will be entered into the SPRINT database within a specified time frame determined by the SPRINT Coordinating Center.

Chapter 4 – Intervention

In Protocol Version 5.0, the below intervention is included for informational purposes only. The blood pressure intervention, which randomized participants to a blood pressure treatment goal of <120 mm Hg or <140 mm Hg, has been discontinued as a result of the Data and Safety Monitoring Board (DSMB) recommendation to unmask trial investigators and notify participants of the lower rate of cardiovascular outcomes and total mortality in the intensive arm.

Participants' blood pressure management is being transitioned from SPRINT to the participants' health care providers. Participants are instructed to continue taking their antihypertensive medications and to contact their primary health care provider. Participants' health care providers should resume responsibility for managing their patients' antihypertensive medication and setting their blood pressure goals.

Participants will continue on their current SPRINT medications until they see their personal physician or health care provider, or unless a change is required for safety purposes. Once a participant's provider has again assumed care of the participant, the study staff will no longer manage the participant's blood pressure, but we will ask participants to come to their regularly scheduled visits until closeout visits begin. However, for participant convenience during the transition and closeout periods, the trial will provide participants with trial medication, including a 3-month supply at the closeout visit. If the health care provider makes changes to the participant's blood pressure medications, the study will provide these medications if they are part of the SPRINT formulary.

Blood Pressure Goals

Participants eligible for the trial will be randomized to one of two goals: SBP <120 mm Hg for the more intensive goal (Intensive Group) and SBP <140 mm Hg for the less intensive goal (Standard Group). Figures 4.1 and 4.2 describe the treatment algorithms for the two treatment groups. Although there are no diastolic blood pressure (DBP) inclusion criteria, participants in both groups with DBP ≥90 mm Hg will be treated to a DBP goal of <90 mm Hg if needed after meeting the SBP goal, because of the many trials documenting the CVD benefits in treating to a DBP goal <90 mm Hg.

Antihypertensive Classes (Agents)

Use of once-daily preparations of antihypertensive agents will be encouraged unless alternative dosing frequency (e.g., BID) is indicated/necessary. One or more medications from the following classes of agents will be provided by the study and intended for use in managing participants in both randomization groups to achieve study goals:

- Angiotension converting enzyme (ACE)-inhibitors
- Angiotension receptor blockers (ARBs)
- Direct vasodilators
- Thiazide-type diuretics
- Loop diuretics
- Potassium-sparing diuretics

- Beta-blockers
- Sustained-release calcium channel blockers (CCBs)
- Alpha1-receptor blockers
- Sympatholytics

Combination products will be available, depending on cost, utility, or donations from pharmaceutical companies

Selection of Antihypertensive Medications

The SPRINT trial is testing a treatment strategy question regarding different SBP goals and not testing specific medications. The SPRINT BP treatment protocol is flexible in terms of the choice and doses of antihypertensive medications, but there should be preferences among the drug classes, based on CVD outcome trials results and current guidelines. NHLBI is updating various guidelines. The update of hypertension recommendations, JNC-8, should be available early in the recruitment phase of SPRINT. These updates, along with any new scientific developments, will be considered during and following SPRINT protocol development and throughout the trial.

The investigator may select among the available SPRINT antihypertensive medications for initiation of therapy. Other drugs not supplied by the trial may also be used as the investigator determines appropriate. However, all antihypertensive regimens should include one or more drug classes with strong CVD outcome data from large randomized controlled hypertension trials, i.e., a thiazide-type diuretic, calcium channel blocker, ACE inhibitor or ARB. Current evidence, the most recent JNC guidelines and over 40 years of clinical trial experience in hypertension support the inclusion of a thiazide-type diuretic as one of the agents for patients without compelling reasons for another medication, or contraindication or intolerance to a thiazide-type diuretic. (ALLHAT, 2002;Beckett and others, 2008;Chobanian and others, 2003;Psaty and others, 1997;SHEP, 1991) Other classes associated with substantial reductions in CVD outcomes in hypertension trials, e.g. ACE inhibitors, ARBs, and calcium channel blockers, combine effectively with thiazides for lowering BP (Julius and others, 2004). ACE inhibitors and ARBs also combine well with CCBs; if three drugs are needed, a thiazide-type diuretic, a RAS blocker (ACE inhibitor or ARB, but usually not both), and CCB make a very effective and usually well-tolerated regimen (Calhoun and others, 2009). The preference for the order in which these agents are selected is left to the investigator as long as the SBP goals are achieved. A loop diuretic may be needed in addition to or in place of a thiazide-type diuretic for participants with advanced CKD.

Beta-adrenergic blockers, which were recommended in JNC-7 among the 4 preferred classes after diuretics, are now considered to be less effective in preventing CVD events as primary treatment of hypertension compared with thiazide-type diuretics, CCBs, and RAS blockers (Lindholm, Carlberg, and Samuelsson, 2005) However, there are patients for whom beta-blockers should be part of the initial therapy, namely those with coronary artery disease, including chronic stable angina or previous MI (Rosendorff and others, 2007).

Finally, although renoprotective benefits have been demonstrated in CKD patients with proteinuria, ACE inhibitors (and likely other RAS blockers) are less effective than other classes in lowering BP and in preventing CVD events in African American and elderly

hypertensive patients unless combined with a diuretic or CCB (Julius and others, 2004;Mancia and others, 2007;National Collaborating Centre for Chronic Conditions, 2006;Wright and others, 2005;Wright and others, 2008).

Since more than three drugs will be necessary in many participants to reach the intensive SBP goal, other classes will also be available in SPRINT. These include the potassium-sparing diuretics, spironolactone and/or amiloride, which are very effective as add-on agents for BP-lowering in "resistant hypertension" (Calhoun and others, 2008). However, they should be used with careful monitoring in participants with CKD or any tendency to hyperkalemia. Alpha-blockers have been used effectively as add-on therapy in the AASK, ACCORD and Anglo-Scandinavian Cardiac Outcomes (ASCOT) trials. However, alpha-blockers should be used only in combination with one or more other agents proven to reduce CVD events in hypertensive patients (ALLHAT, 2003). Sympatholytics, direct vasodilators, and/or loop diuretics may also be added for BP control in combination with agents proven to reduce CVD events.

Among thiazide-type diuretics, the most consistent and robust CVD outcome data have been seen with chlorthalidone (ALLHAT, 2002;SHEP, 1991). Chlorthalidone 12.5-25 mg/d has been shown to be more effective in lowering BP over 24 hours than hydrochlorothiazide 25-50 mg/d (Ernst and others, 2006). Among CCBs, amlodipine has been used in far more hypertension CVD outcome trials than any other agent and has more robust CVD outcome data. Amlodipine should be considered first when a CCB is to be used. In the presence of significant proteinuria, amlodipine should probably be used in conjunction with a RAS blocker. If a non-dihydropyridine CCB (e.g., diltiazem) is to be used, it should not be combined with a beta-blocker.

The ACCORD experience (The ACCORD Study Group, 2010) has shown that a treatment strategy that includes a variety of classes, can produce a 14 mm Hg delta in SBP between the two randomized groups. The average number of antihypertensive drugs used to produce this difference was 3.4 and 2.1 in the Intensive and Standard Groups, respectively. It is anticipated that the study participants in the CKD subgroup of SPRINT will require a greater number of antihypertensive drugs to reach the lower BP goal (Cushman and others, 2008)

Visit Frequency

For both randomized groups, routine visit frequency will be monthly for the first three months after randomization, then every three months for the duration of the trial. "Monthly visits will continue in the Intensive Group until SBP <120 mm Hg (or no more titration planned) and in the Standard group whenever SBP $\geq$ 160 mm Hg." Additional visits will be scheduled as needed for management of adverse effects or for monitoring significant medication changes or other clinical issues.

Intensive BP Goal Group (Figure 4.1)

The SBP goal for the Intensive Group, <120 mm Hg, should be achievable in the majority of participants within 8-12 months of follow-up based on the ACCORD experience (The ACCORD Study Group, 2010). For most participants in the Intensive Group, a two- or three-drug regimen of a diuretic and either an ACE inhibitor or ARB and/or a CCB should be initiated at randomization. If a diuretic is contraindicated or not tolerated, an ACE inhibitor or ARB plus a CCB should be initiated. A beta-blocker

should be included in the initial regimen, usually in combination with a diuretic, if there is a compelling indication for a beta-blocker. Drug doses should be increased and/or additional antihypertensive medications should be added at each visit in the Intensive Group, usually at monthly intervals, until the participant's goal of <120 mm Hg has been reached or the investigator decides no further antihypertensive medications may be added.

SPRINT provides a unique opportunity to determine both the efficacy and safety of intensive BP control in elderly populations. However, based on limited data, there is a concern that this population may be less tolerant of aggressive BP lowering. Therefore, in participants ≥75 years of age randomized to the intensive BP goal who are on 0-1 antihypertensive medications and have baseline SBP <140 mm Hg, antihypertensive therapy may be initiated with a single agent at the discretion of the investigator with a return visit scheduled in one month. If the participant is asymptomatic at the first post-randomization visit and SBP ≥130 mm Hg, a second agent will be added and titration continued as indicated in above.

Milepost Visits

"Clinical inertia" in hypertension management, where clinicians fail to intensify therapy despite patients not being at goal BP, has been observed in both clinical practice (Berlowitz and others, 1998) and clinical trial settings (Cushman and others, 2002). For this reason, "Milepost Visits" were used in the intensive BP group in the ACCORD trial to assist in reaching goal SBP (Cushman and others, 2007). For SPRINT participants in the Intensive Group, Milepost Visits will be every 6 months throughout follow-up, beginning at the 6-month visit. If the SBP is not <120 mm Hg at a Milepost Visit, then an antihypertensive drug from a class different from what is being taken should be added, unless there are compelling reasons to wait. A "Milepost Exemption Form" will be completed whenever a new drug is not added at a Milepost Visit in which the participant's BP is not <120 mm Hg to document the reason for not adding a drug and to outline a plan for making progress toward goal in that participant. Milepost Visit procedures do not apply to the Standard Group. Once the Intensive Group participant has been prescribed 5 drugs at maximally tolerated doses, if the BP remains above goal at subsequent Milepost Visits, it will be permitted to substitute a different class into the regimen instead of adding another drug or increasing the dose of a drug. However, additional (more than 5) drugs may be needed to achieve goal SBP in some participants. Medication adherence will be assessed routinely in SPRINT and should be evaluated especially carefully for participants not at goal on 4 or more medications. Strategies to enhance adherence are described in brief in Chapter 5 and in detail in the Manual of Procedures and Adherence Binder.

Standard BP Goal Group (Figure 4.2)

The SBP goal for the Standard Group, <140 mm Hg, should be achievable in the majority of participants within 3-6 months, based on the ACCORD experience (The ACCORD Study Group, 2010). The standard BP protocol is designed to achieve a SBP of 135-139 mm Hg in as many participants as possible. Participants in this group may or may not be on treatment with one or more antihypertensive medications. If antihypertensive medication(s) is indicated per protocol, consideration should be given to including a thiazide-type diuretic as initial therapy or as part of the regimen, unless there is a compelling indication for another drug class or intolerance to a thiazide.

At the randomization visit, Standard Group participants on previous antihypertensive drug therapy should be converted to SPRINT medications or no medications, depending on what the investigator believes is most likely to achieve a SBP level between 135-139 mm Hg. Because we expect a decrease in average SBP within the Standard Group following randomization due to improved adherence, lifestyle counseling, and intra-individual variation, sometimes described as "regression to the mean", treatment should not be intensified at the randomization visit for Standard Group participants unless SBP ≥160 mm Hg or there is a compelling reason to add medication, e.g., management of fluid balance in participants with CKD. Following the randomization visit, medication dose titration or addition of another drug is indicated if SBP is ≥160 mm Hg at a single visit or is ≥140 mm Hg at two successive visits.

Because it is not known if lowering SBP to the more intensive SPRINT goal of <120 mm Hg, compared with the standard goal of <140 mm Hg, is beneficial, neutral, or harmful in patients such as those entered into the SPRINT trial, careful step-down (a reduction of the dose or number of antihypertensive drugs) is allowed for participants in the Standard Group. Down-titration was not permitted in the HOT Trial if DBP was well below the goal for a participant (Hansson and others, 1998) – this likely contributed to the small differences in achieved BP between the three randomized groups and limited the study's ability to detect differences in outcomes. Therefore, down-titration was included in the ACCORD and AASK standard BP protocols and was successful in generating the planned differences in BP between treatment arms. Down titration should be carried out if the SBP is <130 mm Hg at a single visit or <135 mm Hg at two consecutive visits (Figure 4.2).

Diastolic Blood Pressure Treatment

Once the SBP goal has been achieved in any participant, the antihypertensive regimen should be intensified if DBP remains ≥100 mm Hg at a single visit or ≥90 mm Hg at two successive visits to achieve DBP <90 mm Hg. The visit intervals and decisions for titration (other than the BP levels) will be similar to those used for the SBP goal. Since beta-blockers reduce DBP more than SBP relative to other antihypertensive medications, a beta-blocker could be considered for such participants (Cushman and others, 2001).

Use of Home BP Devices

Home BP devices will not be provided to all participants by the trial. Since virtually all BP outcome trials have used office BP determinations and home readings are subject to more bias and error, in SPRINT titration of medications to goal should be based on office readings rather than home BP determinations.

Assessment of Orthostatic Hypotension (OH), Measurement of Standing Blood Pressure

Standing BP will be measured at screening, baseline, 1 month, 6 months, 12 months, and annually thereafter, and the close-out visit, using the same BP device that is used to measure seated BP. After seated determinations, participants will be asked to stand. Beginning when their feet touch the floor, BP will be taken one minute later in the same arm used for the seated measurements, using the BP device. Participants will be asked after the standing determination if they had any symptoms of orthostatic hypotension

during the standing BP measurement. The Coordinating Center will calculate BP change using the standing measurements minus the mean of the seated measurements.

Participants with standing SBP <110 mm Hg will not be eligible for randomization (may be rescreened if corrected). However, the detection of asymptomatic orthostatic hypotension, i.e., orthostatic hypotension unaccompanied by orthostatic symptoms of dizziness, presyncope or syncope, will not influence the antihypertensive drug treatment algorithm. Symptomatic orthostatic hypotension will be managed as described in "Management of Symptomatic Orthostatic Hypotension" (see Manual of Procedures).

4.1 Lifestyle Recommendations and Background Therapy

The purpose of including lifestyle recommendations and background therapy in SPRINT is twofold. First, it fosters high quality general medical care in all SPRINT participants in accordance with current practice guidelines. Second, it is intended that background therapies will be utilized equally across both study arms in order to minimize the differences in the effects of non-study strategies on the SBP or CV outcomes between arms. The background therapy recommendations will be provided to the participants and their physicians. Background therapy is considered part of usual recommended care for patients at risk of CVD and, as such, is not covered by research study costs. The delivery of these background therapies will be left up to the participants' own clinicians.

The Lifestyle and Background Therapy Working Group will coordinate the provision of the most current and relevant participant educational materials to be made available for study-wide use. These will include the topics of medical nutrition therapy, weight management, physical activity, smoking cessation, and anti-thrombotic therapy, and will complement educational materials related to the BP interventions that are part of the trial. Unlike most educational materials for BP, the SPRINT materials will not include specific goals for BP as these will depend on the participants' randomized treatment assignment. Specific recommendations will include: a) weight loss in those who are overweight or obese; b) adoption of a diet rich in fruits, vegetables and low-fat dairy products (the DASH diet) with appropriate modifications for participants with CKD; c) reduction in sodium intake to recommended levels; d) reduction of alcohol consumption to recommended levels; and e) participation in regular aerobic exercise. SPRINT participants will be encouraged to stop smoking (if a current smoker) and to follow current guidelines for testing for and treatment of dyslipidemia and the use of antithrombotic therapy.

Figure 4.1 Treatment Algorithm for Intensive Group (Goal SBP < 120 mm Hg)

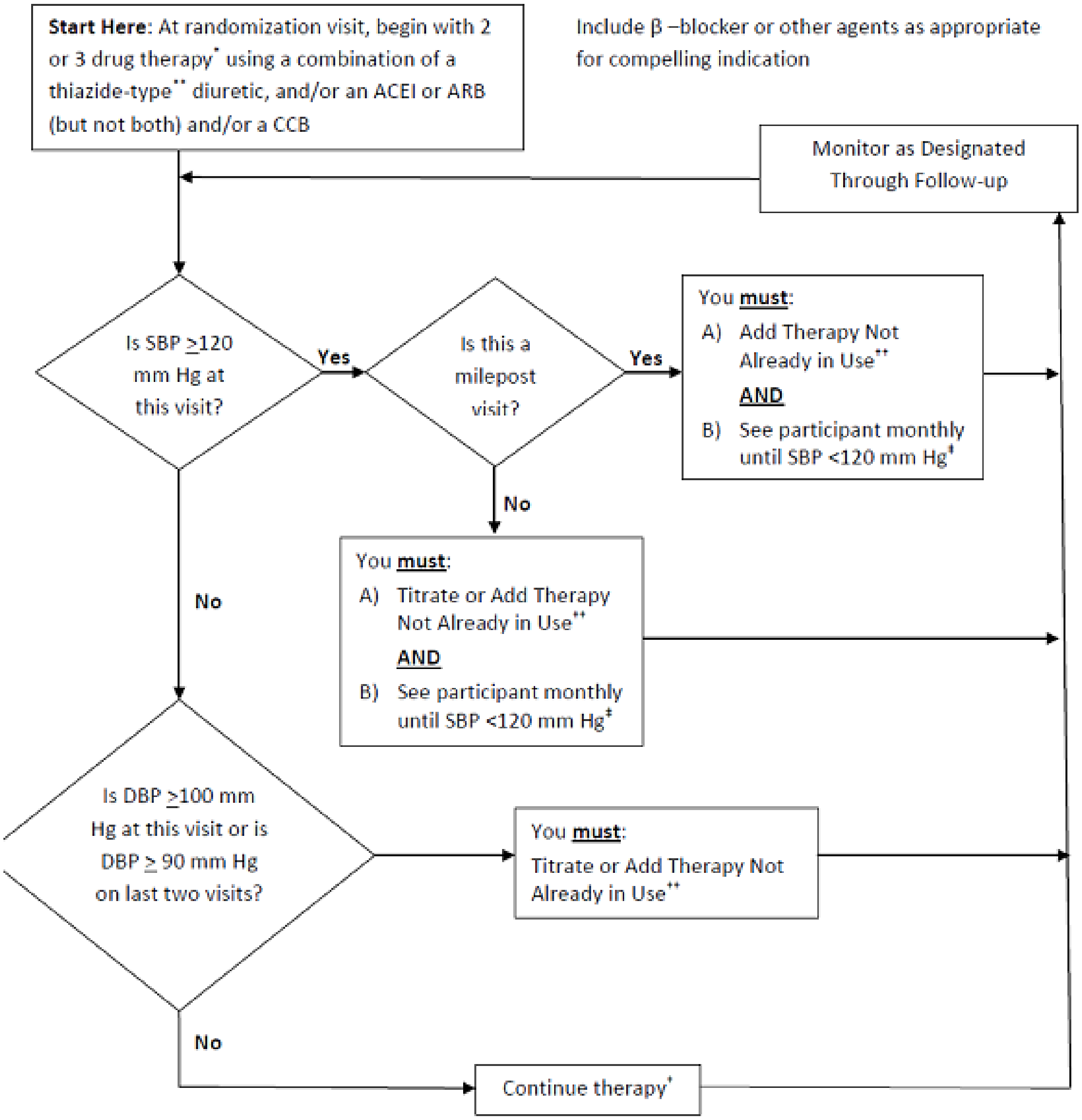

* May begin with a single agent for participants 75 years old or older with SBP < 140 on 0-1 meds at study entry. A second medication should be added at the 1 Month visit if participant is asymptomatic and SBP ≥ 130.

** May use loop diuretic for participants with advanced CKD

+ Unless side effects warrant change in therapy

++ Consider consulting with the Clinical Center Network before adding a fifth anti-hypertensive medication

‡ Or until clinical decision made that therapy should not be increased further

Figure 4.2 Treatment Algorithm for Standard Group (Goal SBP < 140 mm Hg)

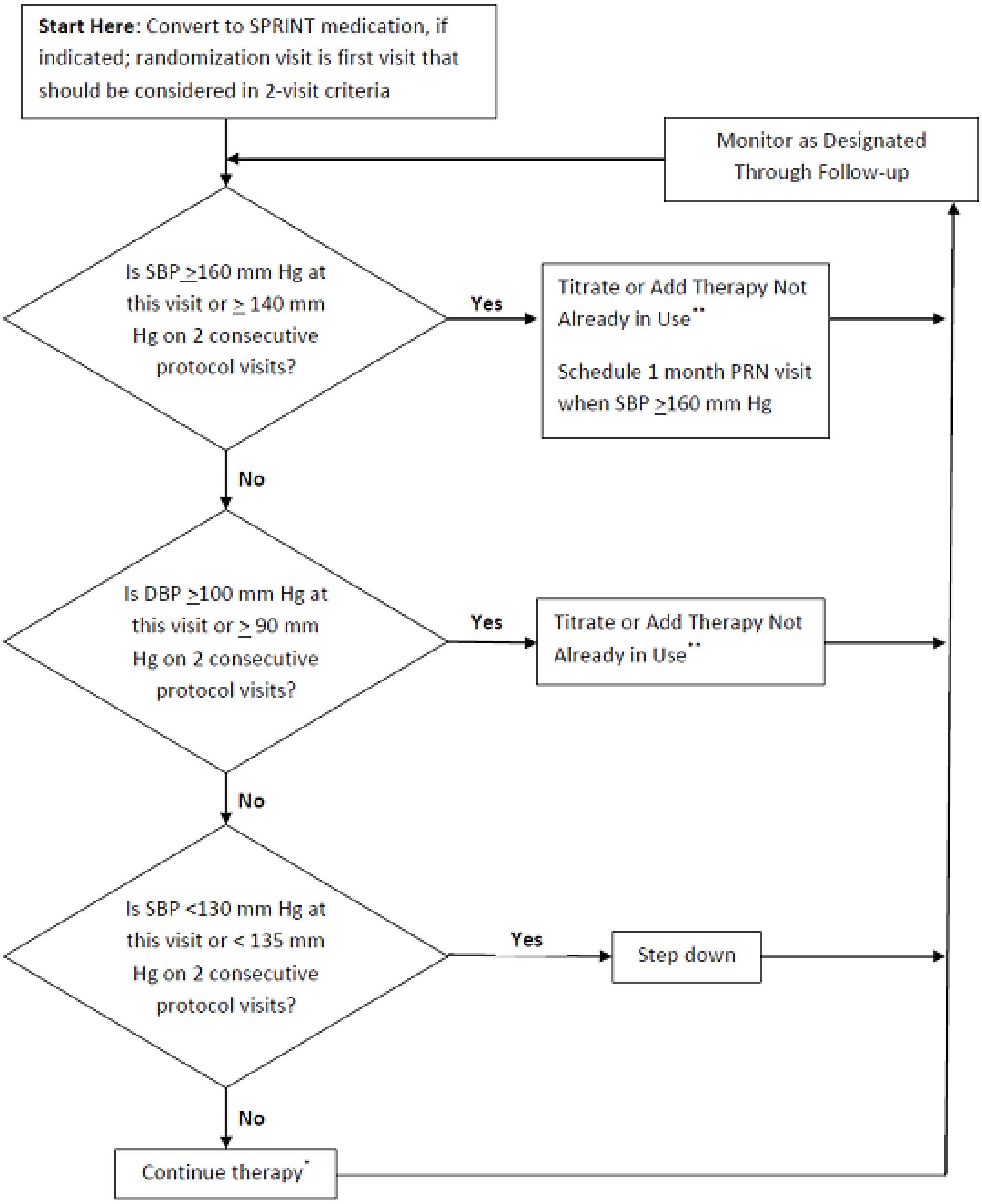

Include β –blocker or other agents as appropriate for compelling indications

* Unless side effects warrant change in therapy

** Consider consulting with the Clinical Center Network before adding a fifth anti-hypertensive medication

Chapter 5 – Measurements and Follow-up

5.1.1 Schedule of Follow-Up Visits

Post-randomization follow-up visit schedules for data collection do not differ by treatment group assignment. However, the visit schedule for treatment, that is achieving the BP goals, may differ by group while blood pressure goals are being met because of PRN visits not shown on Table 5.1. Additional information on treatment schedules is contained in Chapter 4 describing the SPRINT BP intervention. For data collection in both randomized groups, all participants will have post-randomization visits at Months 1, 2, 3, 6, and every 3 months thereafter. For the purpose of event ascertainment, all participants in both treatment groups will be queried regarding the occurrence of a possible event on the same schedule, specifically every 3 months.

5.1.2 Procedures by Visit

Scheduled examination components are shown by visit in Table 5.1. Assessments performed at the various visits include blood and urine collection, physical measures, and questionnaires. Assessments will be performed on the same schedule for both randomization groups. Baseline characteristics to define the patient population include sociodemographics, anthropometrics, BP, pulse, current and past medical history, concomitant medications, laboratory, dementia screening, cognitive function (subset), MRI (subset) and quality of life measurements. A physical examination is included for safety but is not standardized, and left to the discretion of the investigator.

5.2. Blood and urine collection and laboratory assays

Specific laboratory assessments (e.g. serum creatinine, fasting serum glucose, etc) are important for determining eligibility status. During follow-up, laboratory results will be used to monitor and adjust therapy in efforts to maintain blood pressure goals, assess safety (e.g. serum potassium concentrations), and to assess for study-related outcomes (e.g. deterioration of estimated glomerular filtration rate or increased protein excretion).

Serum, plasma, and urine samples will be stored for future measurements of other less traditional CV risk factors. White blood cells will be collected at baseline for DNA extraction for future genetic studies. It may prove possible to identify subgroups, defined by specific genes or genetic markers, which respond differentially to the various blood pressure treatment strategies.

5.3. Physical Examination Measures

5.3.1 Seated Blood Pressure and Pulse

Seated blood pressure and pulse are measured at each clinic visit after a rest period using an automated device or manual devises if necessary. The preferred method is the automated device as it offers reduced potential for observer biases and decreased demand on staff in terms of training and effort in data collection.

Table 5.1. Measures and Frequency

	Screening /RZ	1 mo	2 mo	3 mo	6 mo	9 mo	1 yr	Q 3 mo	Q 6 mo	2 yr	3 yr	4 yr	Close Out A*	Close Out B**
Blood collection														
Chemistry profile		X		X	X				X		X		X	
Fasting Chemistry profile	X						X			X		X		X
Fasting glucose	X									X		X		X
Fasting lipid profile	X						X			X		X		X
Fasting serum and plasma storage	X						X			X		X		X
Genomic material	X													
Complete Blood Count (CBC)***													X	X
Urine collection														
Albumin, creatinine	X				X		X			X	X	X	X	X
Fasting urine storage	X						X			X		X		X
Physical measures														
Seated blood pressure, pulse, & medication adjustment	X	X	X	X	X	X	X	X		X	X	X	X	X
Standing blood pressure	X	X			X		X			X	X	X	X	X
Weight	X						X			X	X	X	X	X
Height	X													
ECG	X									X		X		X
Physical examination	X						X			X	X	X	As required locally	As required locally
4 meter walk (≥ 75 ONLY)	X						X			X	X	X	X	X
Questionnaires														
Medical history	X													
Sociodemographics	X													
Alcohol use	X													
Smoking	X						X			X	X	X	X	X
Concomitant medications	X						X			X	X	X	X	X
Adherence & Adverse Events		X	X	X	X	X	X	X		X	X	X	X	X
Outcomes Ascertainment				X	X	X	X	X		X	X	X	X	X
**Health related quality of life**														
EQ-5D	X						X			X	X	X	X	X
Veterans Rand 12	X						X			X	X	X	X	X
PHQ-9 Depression	X						X			X	X	X	X	X
Patient satisfaction/Morisky	X						X					X		X
**Health related quality of life (subsets)**														
Falls Efficacy (FESI-I)	X				X		X			X	X	X		X
Sexual Function (FSFI/IEFF)	X				X		X			X	X	X		X

MIND Questionnaires/Tests	Screening or RZ	2 yr	4 yr	Close Out A*	Close Out B**
**Dementia Screening**					
MoCA	X	X	X		X
Digits Symbol Coding Test	X	X	X		X
Logical Memory Test Story A	X	X	X		X
**Cognitive Battery (subset)**					
Hopkins Verbal Learning Test	X	X	X		X
Trail Making Tests A and B	X	X	X		X
Digit Span	X	X	X		X
Boston Naming Test	X	X	X		X
Modified Rey-Osterrieth Figure	X	X	X		X
Verbal Fluency Animals	X	X	X		X

*****Close-out Visit A – Participants who have completed year 4 visit by the date of the site approval of this protocol amendment**
****Close-out Visit B – Participants who have NOT completed year 4 visit by the date of site approval of this protocol amendment**
*****Complete Blood Count (CBC) will only be performed on participants included in the MRI study**

5.3.2 Standing (Orthostatic) Blood Pressure

Standing BP will be measured at screening, baseline, 1 month, 6 months, 12 months, and annually thereafter, and the close-out visit, using the same BP device that is used to measure seated BP. After seated determinations, participants will be asked to stand. Beginning when their feet touch the floor, BP will be taken one minute later in the same arm used for the seated measurements, using the BP device. Participants will be asked after the standing determination if they had any symptoms of orthostatic hypotension during the standing BP measurement. The Coordinating Center will calculate BP change using the standing measurements minus the mean of the seated measurements.

5.3.3 Anthropometric Measurements (Weight and Height)

Body fat is a significant predictor for subclinical and clinically manifested cardiovascular disease. In addition, exercise and dietary modification with the goal of reducing total body fat may facilitate blood pressure control. Anthropometric measures gathered for SPRINT include height and weight for the calculation of body mass index.

5.3.4 Electrocardiography

A 12-lead ECG is obtained at baseline and at the 2 and 4 year follow-up visits and close-out visit, if the participant has not yet reached the 4 year follow-up visit, to ascertain the occurrence of silent (unrecognized) MI, primarily, as well as atrial fibrillation and left ventricular hypertrophy. The baseline ECG is used to identify previous (including silent) MIs, and to identify evidence of left ventricular hypertrophy.

5.3.5 Physical Examination

The physical examination includes components of a systems-based examination deemed necessary for safety by the SPRINT site investigator. Elements of the examination to be completed may vary depending upon the health status and any symptoms reported by the participant, the time and type of visit (initial, interval, annual, close-out). The physical examination will not be standardized or data entered, but will be available in the source documents for each participant.

5.3.6 Four meter walk

Participants who are 75 years old or older at baseline will be asked to complete a timed 4 meter walk to assess physical function. This will be done at baseline, annually, and at the close-out visit.

5.4. Questionnaires

5.4.1 Medical History

A detailed history of cardiovascular disease is collected at screening. The presence of CVD and CKD prior to entry into the study serves as an eligibility and stratification factor. Data regarding the duration of chronic kidney disease and the presence of complications are important for descriptive purposes, subgroup analyses, and prognostic analyses.

5.4.2 Sociodemographics

Information is collected during screening/baseline regarding age, race and ethnicity, gender, level of education, marital status, persons living with participants and United States (zip) postal code. These data will be used to identify eligible participants and to characterize the final study population.

5.4.3 Smoking/alcohol use

Consumption of alcohol and tobacco have important implications on cardiovascular risk, and adherence to medication regimens. Participants will be assessed at baseline for lifetime tobacco exposure, alcohol intake and binge drinking. At annual assessments and at the close-out visit, current smoking will be assessed.

5.4.4 Concomitant Medications

Information regarding the participants' concomitant non-study medication therapy is collected and documented at baseline and then reviewed and revised at annual follow-up visits as well as at the close-out visit. Appropriate sources for obtaining this

information include participant report, current pharmacy action profiles, and verification of medications documented in the medical record. Although data are collected on all current therapies, emphasis is placed on concurrent antihypertensive, cardiovascular, chronic kidney disease and dementia medications as well as background risk reduction therapy such as aspirin and lipid-lowering drugs.

5.4.5 Monitoring Adherence

Adherence to antihypertensive medications will be assessed as follows:

First, an adherence scale will be administered to all participants at the baseline, 12 month and 48 month visits, and the close-out visit if the participant has not reached their 48M visit at the time of close-out, in order to identify low adherence.

Secondly, at every medication management visit, participants will be administered a single item to screen for low adherence. If the participant's response to this item indicates a possible problem with adherence, or if the participant is not at the appropriate blood pressure target, study personnel will address the specific issues and barriers for each study participant that may be preventing optimal adherence. In such instances, administration of the Adherence Scale (to identify reasons for nonadherence) is recommended, as is use of the materials and procedures described in the adherence binder. Details regarding the adherence monitoring procedure, scoring algorithm for the Adherence Scale and the procedures to follow when low adherence is identified are provided in the MOP.

5.4.6 Adverse events

Adverse event ascertainment and reporting is described in chapter 8.

5.4.7 Study-related outcomes

Both randomized groups will be assessed for study related outcomes in the same way and on the same schedule. After randomization, participants will be assessed every 3 months for cardiovascular and renal outcomes. Medical records will be collected for adjudication of study outcomes as described in Chapter 9. Clinical center staff will use available resources and contact information to assess vital status annually on participants not attending study visits.

5.4.8 Health-Related Quality of Life

All participants will be assessed for the effect of interventions on health-related quality of life (see Chapter 7). HRQL data will be collected at Baseline, 12 months and annually thereafter, as well as at the close-out visit. Depression using the PHQ-9 scale will be assessed at baseline and annually thereafter, and at the close-out visit. A modified TSQM General Satisfaction subscale will be administered at baseline and at 1 and 4 years. A subset of participants will undergo additional data collection related to fall self-efficacy and sexual functioning at baseline, 6 months and annually thereafter. This same subset will receive the fall self-efficacy at the close-out visit, if the participant has not yet reached the 4 year follow-up visit.

5.4.9 MIND Battery: Dementia Screening

All participants will undergo a dementia screening at baseline, 24M, and 48M or close-out visit (only if the participant has not completed the 48M MIND tests). The tests will include the Montreal Cognitive Assessment (MoCA), Digit Symbol Coding test, and Logical Memory test. A subset of 2800 participants will undergo an additional comprehensive battery of neurocognitive tests conducted at baseline, Month 24, and Month 48 or close-out visit (only if the participant has not completed the 48M MIND tests). In addition, participants who trip the dementia screening battery also will receive this comprehensive battery of neurocognitive tests. In addition to the neurocognitive tests, a subsample of 640 MIND participants will have a Baseline and Month 48 MRI examination.

5.4.10 Consent for Future Contact

At the close-out visit, participants will be asked to sign an addendum to the informed consent for future contact.

5.5. Medications and Adherence

Adherence

As part of a central pretrial training session, all investigators and clinical coordinators will receive instruction on adherence issues. Additionally, study staff will periodically have refresher and retraining instruction in the overall adherence program throughout the trial. Also critical to maintaining good adherence is the routine discussion of participants who show problems with adherence and brain-storming about problem-solving strategies during clinic team meetings and Study Coordinator meetings and conference calls. Of particular importance is the involvement of all members of the clinic team, including clinic leadership, in adherence-related monitoring and problem-solving.

Drug Dispensing, Ordering, Storage, and Disposal

Drug Dispensing

The complexity created by the large number of medications and multiple treatment strategies employed by SPRINT requires substantial attention to the process of medication dispensing. All study medications dispensed to the participants will be labeled and identified with the study name, participant's name, medication name, strength and quantity, directions for use, and authorized prescriber's name. An emergency study-related phone number for study drug information will also appear on the label. All participants are to be verbally counseled on medication administration. Written instructions will also be provided.

Participants receive medication supplies at regularly scheduled visits in sufficient quantity to last until the next scheduled visit. Medication dispensing may occur in the intervening periods between visits in case of emergency, loss, or schedule changes. A tracking mechanism is maintained for all dispensing actions. It is recommended that authorized dispensing personnel be limited in number to assure proper adherence with established accountability and dispensing procedures.

Drug Supply Ordering
Each Clinical Site, upon completion of procedures for study initiation, will receive a standard initial shipment (determined by the Coordinating Center and prepared by the Drug Distribution Center (DDC)) of study drug supplies for the trial. It is expected that this initial shipment will suffice for a specified number of visits for a given number of randomized participants. Subsequent ordering of inventory will be managed by the site, primarily through the web-based inventory system. Sites are responsible for appropriately managing their inventory and are able to customize their medication quantities to suit the prescribing practices of their site.

The DDC in consultation with each Clinical Site sets inventory levels for each item. When an item reaches the reorder point, additional stock is automatically shipped from the DDC.

Drug Receipt and Storage
Drug shipments are sent to the Clinical Site in care of a designated staff member. The shipment is inspected for damage and its contents reconciled with the accompanying SPRINT Shipping Notice. The inventory is logged using the established tracking mechanism. Packing slips are filed in a secure location. Any damage or discrepancies in the shipment are to be reported promptly to the DDC for corrective action. Each Clinical Site is responsible for storing the study drug supplies in a locked, secure area with limited access. Manufacturer recommendations and local policies for drug storage are followed.

Drug Disposal

Clinical Sites are authorized to destroy SPRINT stock locally, complying with any local policies and procedures. Destruction will be documented via the web-based inventory system. All study drugs are labeled with an expiration date. Prior to expiration, the DDC will automatically ship replacement stock based on the current electronic inventory profile. Once replacement stock is received the clinical site will destroy expired stock and document destruction as described above.

Chapter 6 – SPRINT MIND

6.1 SPRINT-MIND Overview

SPRINT-MIND is an integral part of the overall SPRINT study and all SPRINT participants will participate in one or more components of SPRINT-MIND. There are three objectives of SPRINT-MIND. The primary objective is to determine whether a strategy of intensive blood pressure lowering to target systolic blood pressure (SBP) <120 mm Hg versus a standard treatment target of <140 mm Hg will produce a greater reduction in the incidence of all-cause dementia. The second objective is to determine whether global cognitive function measured in key specific domains of cognition will decline less in persons randomized to a SBP goal of <120 mm Hg versus a standard treatment goal of <140 mm Hg in a representative sub-sample of approximately 2800 SPRINT participants. The third objective is to assess whether MRI-derived changes in brain structure differ by treatment assignment in a subset (approximately 640) of the 2800 participants.

6.2 Study Hypotheses and Aims

6.2.1 All-cause Dementia

Primary hypothesis: Over an average of 60 months, the incidence of all-cause dementia will be lower in SPRINT participants assigned to the intensive SBP treatment arm compared to their counterparts assigned to the standard SBP treatment arm. This hypothesis will be tested in all SPRINT participants.

6.2.2 Cognitive Decline

Secondary hypothesis: Over an average of 48 months, the rate of global decline in cognition measured across key domains of cognition will be lower in the intensive SBP treatment arm compared to the standard SBP treatment arm. This hypothesis will be tested in a representative subset of approximately 2800 participants enrolled in SPRINT.

6.2.3 MRI Brain Changes

The Primary brain MRI hypothesis is that over an average of 48 months, the volume small vessel ischemic disease (SVID) will be lower in SPRINT participants assigned to the intensive SBP treatment arm compared to their counterparts assigned to the standard SBP treatment arm. An additional hypothesis is that total brain volume will also be greater (thus less atrophy) in the intensively treated group. The MRI sub-study will be conducted in approximately 640 participants chosen from the 2800 subset of participants selected in 6.2.2.

6.3 Study Design

6.3.1 Study Population

We will ascertain incident all-cause dementia in all participants enrolled in SPRINT. In addition, approximately 2800 participants will be selected to receive additional cognitive assessments at baseline, 24 months, and 48 months (or the close-out visit if the 48

month tests have not been administered)in order to examine changes in global and domain-specific cognition. Participants participating in the MRI substudy will, at baseline, generally be required to reside within 1.5 hours travel distance to a designated study MRI Scanner. The components of the two cognitive batteries selected to assess dementia incidence and decline in cognition are listed in Table 5.1 of Chapter 5.

6.4 Procedures for Identifying Incident All-Cause Dementia in SPRINT (see Figure 6.1).

6.4.1 Overview

A 3-step process will be used to ascertain incident cases of all-cause dementia. First, to identify possible cases of dementia a brief Cognition Screening Battery will be administered to all participants. Participants who score below the pre-designated screening cut-point for possible cognitive impairment during follow-up will be administered a more comprehensive and detailed neurocognitive test battery (the Extended Cognitive Assessment Battery) plus the Functional Assessment Questionnaire (FAQ) which assesses impairments in daily living skills as a result of cognitive impairments. Last, all the above available tests and questionnaire data will be submitted to a centralized, web-based system for adjudication by a panel of dementia experts who will assign final study classifications of probable dementia (PD), mild cognitive impairment (MCI) or no impairment (NI).

6.4.2 Cognition Screening Battery

A brief screening battery consisting of 3 well-validated neurocognitive tests will be administered to all participants at study randomization and repeated at years 2, 4 (or close-out if the Year 4 testing has not occurred. This battery requires 15 minutes or less to administer.

Tests included in the SPRINT-MIND Cognition Screening Battery were selected because they are sensitive to detecting dementia, easy to administer and brief. They are:

1. The Montreal Cognitive Assessment (MoCA) The MoCA (Nasreddine et al., 2005) is part of the NIH Toolbox and is a reliable and valid brief screening instrument for characterizing global cognitive functioning. It has been used previously to screen for dementia and MCI with sensitivity of >85%. The MoCA has several sub-scales that can be used to characterize more specific cognitive functions.

2. Digit Symbol Coding test (DSC) The DSC ((Wechsler, 1996b; Wechsler D., 1981) is a sub-test of the Wechsler Adult Intelligence Scale-IV. It measures psychomotor speed and working memory. The DSC and its predecessor the Digit Symbol Substitution test have been extensively used and normed.

3. Logical Memory test (LM): The LM test is a sub-test of the Wechsler Memory Scale-IV(Wechsler, 1996a; Wechsler, 1996a). It measures episodic verbal memory and has extensive normative data. Episodic verbal memory is an especially sensitive predictor of early Alzheimer’s dementia and amnestic MCI.

The sensitivity and specificity of the Cognition Screening Battery to detect participants with poorer cognitive function will be evaluated on an ongoing basis during the trial by using available baseline cognition data from SPRINT. We estimate 20-25% of participants will trip the battery and receive a brief assessment of the impact of their cognitive function on daily life (the 10 item FAQ). At the years 2 and 4 (or close-out visit if the Year 4 tests have not been administered, participants who trip the screening battery will also be administered the SPRINT Extended Cognitive Assessment Battery and the FAQ for adjudication of incident dementia. In order to achieve the 20-25% target, various cut-points for the Cognition Screening Battery will be compared and adjustments will be made to maximize study efficiency and economy during the trial.

6.4.3 SPRINT Extended Cognitive Assessment Battery

The Extended Cognitive Assessment Battery will provide a more comprehensive and detailed assessment of specific major cognitive functions (memory, language, visuospatial skills, executive function) that are necessary for classification of dementia and for detecting domain-specific changes. During follow-up years 2 and 4 (or the close-out visit if the Year 4 tests have not been administered), participants scoring in the impaired range on the Cognition Screening Battery will be administered the Extended Cognitive Assessment Battery at their next scheduled visit (typically a blood pressure assessment and medication distribution visit). This entire battery requires less than 40 minutes including scoring and data entry and less than 30 minutes in persons without significant memory impairment.

The neurocognitive tests comprising the Extended Cognitive Assessment Battery are:

1) The Hopkins Verbal Learning Test (HVLT) (Brandt and Benedict, 2001)**:** A measure of episodic verbal learning and memory, this test is a 12-item list learning and memory task with immediate recall, delayed recall and recognition components.

2) The Trail Making Test: Parts A and B (Reitan R.M., 1958)**:** The Trail Making Test (TMT) is a two-part test measuring speed of processing and executive function. The times to complete Part A and Part B are the primary measures of interest.

3) Digit Span test (Wechsler D., 1981): The Digit Span test (DST), a subtest of the Wechsler Adult Intelligence Scale-IV, requires the participant to recite gradually increasing series of digits forward and backward. The DST measures concentration and working memory.

4) The Boston Naming Test (Kaplan E et al., 1983) The Boston Naming Test (BNT) is used to assess language function. The participant is asked to name familiar objects from simple drawings. The number of correctly identified objects is the variable of interest. We will use a validated short form that includes 15 items.

5) The Modified Rey-Osterrieth Complex Figure (mRey-O). (Saxon, 2003) The mRey-O measures of visuospatial and visuomotor function and non-verbal memory by having participants copy and reproduce from memory a multi-component figure. For ease of use and scoring reliability, the mRey-O figure will be faxed to the CC and scored centrally.

6) Category Fluency-Animals. The animal fluency task requires the participant to spontaneously name as many animals as possible in 60 seconds. It provides an assessment of semantic fluency.

6.4.4 Additional measures

Functional Assessment Questionnaire (FAQ). Since impairment of daily functioning is required for a classification of dementia, we also will administer, either locally (by certified SPRINT clinic staff) or centrally (by certified SPRINT staff from the coordinating center), the FAQ, a 10-item, validated questionnaire assessing functional status (Pfeffer and others, 1982), to a person previously designated by the participant who is familiar with his/her current abilities. Administration of the FAQ will be limited to participants in the 2800 and those participants whose Cognition Screening Battery indicates possible impairment. Items assess functions like managing money and remembering names of familiar persons.

6.4.5 Alternative cognitive assessment.

If participants cannot come to the clinic for their follow-up exams or if they reside in nursing homes, study personnel will complete either a home or nursing home visit. Technicians conducting the home visit must be MIND certified. The Screening Battery and the Extended Battery can be administered during home visits.

Telephone assessment of general cognitive function is now standard practice in many large trials assessing for dementia outcomes. For SPRINT participants unable to receive a face-to-face cognitive assessment by certified SPRINT staff at their local clinic, a telephone assessment of cognition status to assess for incident dementia will be performed centrally by SPRINT certified staff. The components of the **phone interview** are:

Modified Telephone Interview for Cognitive Status (TICS-M), a validated instrument requiring <10 minutes (Welsh, 1993)
Category Fluency-Animals
Oral Trail Making Test (Ricker et al., 1996)
FAQ to a contact

For participants unable to be interviewed in-person or by phone, a previously identified contact will be administered:

The Dementia Questionnaire (DQ). The DQ (Ellis , 1998;Kawaset al, 1994) is a semi-structured interview designed for a knowledgeable proxy to provide information regarding the participant's cognitive and behavioral functioning and other health information needed to make a diagnosis of dementia and MCI and to identify causes of cognitive impairment. Again, it will only be administered in the absence of an in-person or phone assessment and may be performed either by local or central staff who are SPRINT certified. The DQ will also be obtained on all participants who died more than 1 year after their last MIND testing.

6.5 Adjudication of Dementia, MCI or No Impairment

A primary goal of SPRINT MIND will be to determine the incidence of all-cause dementia in SPRINT and its relation to the treatment assignment. Final classification (Dementia,

MCI or No Impairment) will be made by a panel of experts consisting of neurologists, geriatricians, psychiatrists and neuropsychologists with recognized expertise in dementia blinded to study assignment and blood pressure data. Data used in the adjudication will include all available cognitive test data (SPRINT Cognition Screening Battery, SPRINT Extended Cognitive Battery), functional status assessments (FAQ or DQ) and additional data including demographic information and medical history. Each suspected case identified by our scoring criteria (see 6.4) will be randomly assigned to two members of the Adjudication Committee for review. Adjudicators will independently review all the available data via a web-based system before recording their classification-Dementia, MCI or No Impairment. Each adjudicator will be masked to the other's classification and to the participant's treatment assignment. If the two adjudicators' classifications agree, then the classification will become final. Disagreements will be resolved at periodic face-to-face meetings or by phone conferences between adjudicators and additional members of the Adjudication Committee until consensus is achieved. These procedures have been successfully used by our team in other large clinical trials including the Gingko Evaluation of Memory Study (GEMS) (DeKosky et al, 2008) and the Women's Health Initiative Memory Study (WHIMS) (Shumaker et al, 2004).

Participants classified as having dementia will no longer be assessed for cognitive function. Those not classified as having dementia will continue to receive regularly scheduled cognitive assessments with the screening and extended cognitive batteries when indicated.

6.5.1 Diagnostic Criteria for Dementia

Criteria used for identifying dementia will be those described in the Diagnostic and Statistical Manual of the American Psychiatric Association-Fourth Edition (DSM-IV). These are:

- Significant decline in memory and at least one additional cognitive domain; and
- Significant functional impairment due to cognitive problems; and
- Cognitive deficits are not due to obvious reversible causes such as acute illness, metabolic disturbances, infections, mood disorders or substance-induced conditions; and cognitive deficits do not occur exclusively during the course of delirium.

No attempt to classify dementia subtype will be made.

6.5.2 Diagnostic Criteria for MCI

While not a primary or secondary outcome, MCI syndrome is important because of its relevance to dementia. MCI represents a transitional state between no cognitive impairment and dementia and specific subtypes of MCI are highly predictive of subsequent dementia. Thus, identifying MCI will provide valuable information about pre-dementia cognitive impairment related to the SPRINT intervention. Criteria to be used for identifying mild cognitive impairment syndrome are those described by Winblad et al., which are:

- Observation by participant or proxy of cognitive decline; and
- Deficit in performance in one or more cognitive domains; and
- Absence of significant functional impairment attributable to cognition; and
- No diagnosed dementia

MCI will be further sub-classified into 4 categories using criteria adapted from Winblad, et Al. (Winblad et al, 2004) as follows:

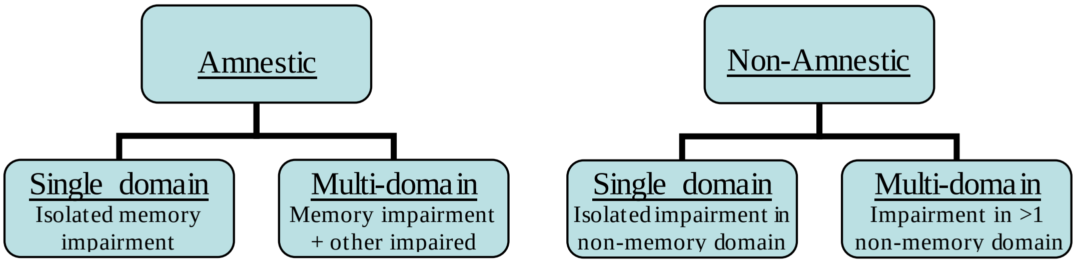

Specific cognitive tests in the Cognition Screening Battery and the Extensive Cognitive Assessment Battery will be used to subtype adjudicated cases of MCI.

6.6 Baseline classification of cognitive status:

Rare cases of dementia, where the participant or their personal physician are unaware of the diagnosis, may be identified during baseline cognitive testing. In participants scoring below the cut-point on the Screening Battery, we will administer the FAQ to a contact in order to determine the presence of impaired daily function related to cognition (see 6.4.2).

6.7 Definition of Cognitive Change Over Time Outcome (Extended Cognitive Assessment Battery Sub Sample).

Each test score from the Cognition Screening Battery and the Extended Cognitive Assessment Battery will be used to measure decline in cognitive function. The primary outcomes will be composite scores for two domains: 1) Memory, consisting of the Hopkins Verbal Learning Test, Logical Memory and the Modified Rey Osterrieth Figure, and 2) Processing Speed, consisting of Trails Making Tests and Digit Symbol Coding Test. Prior to analysis of this outcome, we will review the science related to summary scores for cognitive function and may make modifications which will be specified prior to initiation of the analysis.

6.8 Quality Control and Training

At each clinical site, at least one person will be identified to serve as the trained and certified cognitive technician. Technicians will be trained during a central, intensive training session held in conjunction with the overall SPRINT training. Training will include review of the MIND protocol and procedures for administration of the test batteries, demonstrations of each component of the SPRINT MIND test batteries, and opportunities to practice with feedback from trainers. When a level of competence is attained, technicians will receive certification and approval to administer the test batteries to SPRINT participants. During the course of the study as additional staff are needed, certified technicians will train new technicians and submit materials to the MIND Coordinating Center for review. Technicians will be recertified throughout the course of the trial byreview of audio taped administrations. Technicians will be encouraged to communicate questions or problems to the SPRINT MIND Coordinating Center.

Figure 6.1.

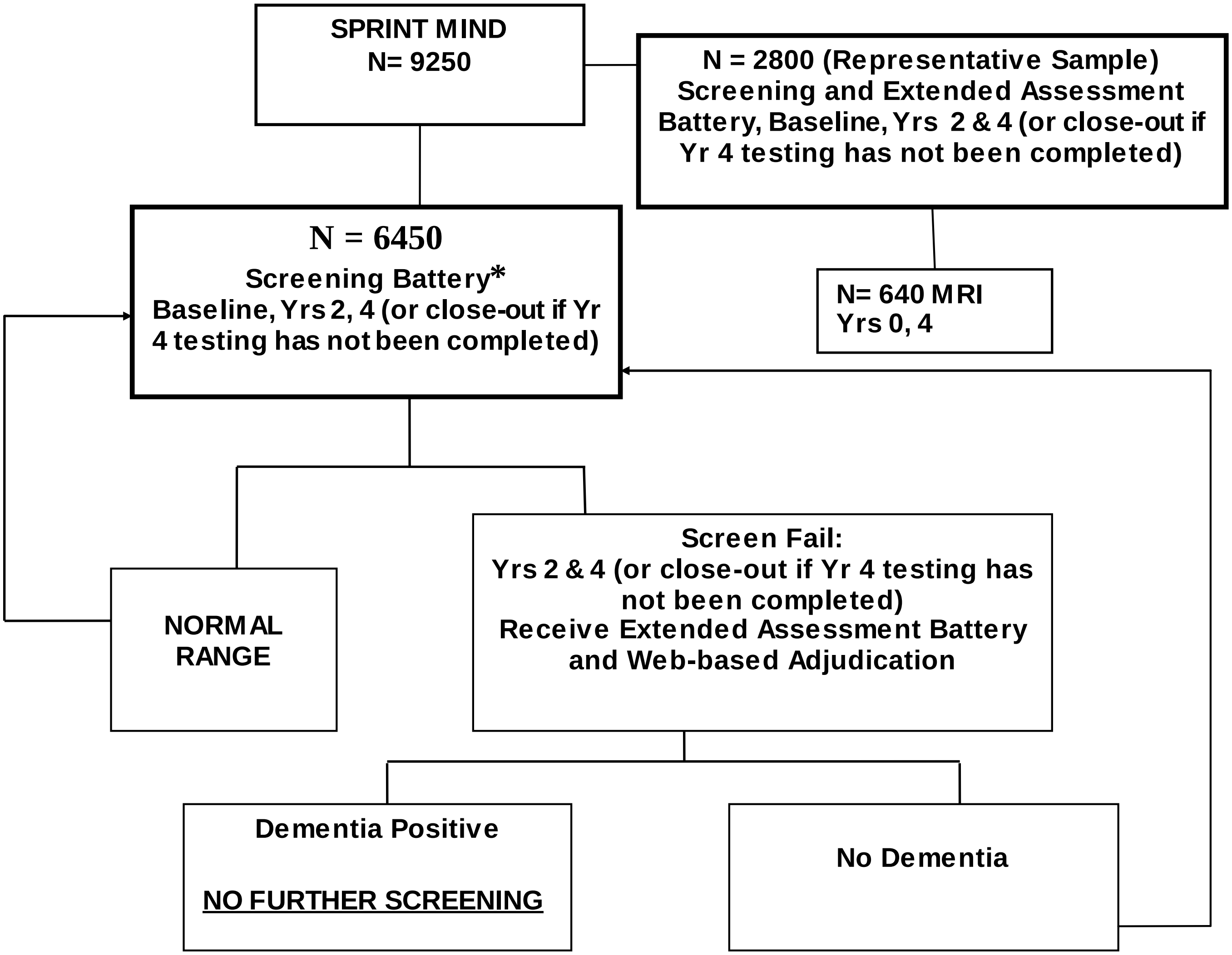

*At baseline, participants scoring below cutoffs specified during trial will also receive the FAQ.

Chapter 7 – Health-Related Quality of Life and Economic Analyses

7.1. Introduction

In addition to the cardiovascular, renal and cognitive outcomes, SPRINT is well poised to examine differences in health-related quality of life (HRQL) as a result of its blood pressure interventions. Differences in HRQL may affect adherence, and thus the effectiveness of the two interventions. It is also reasonable to anticipate that in some cases, the intensive arm may result in diminished HRQL relative to the standard arm due to a number of factors:

- side effects of specific medications or increased numbers and/or doses of medications required to achieve the <120 mm Hg goal,
- increased occurrence of hypotensive symptoms, which may not only result in higher rates of falls and fractures, but also an increased fear of falling which may limit the participant's perceived ability to engage in activities of daily living, and/or
- reduced perfusion pressures and medication side effects which may contribute to erectile dysfunction in men, and possible sexual dysfunctions in women.

On the other hand, the intensive arm may result in improved general HRQL versus the standard arm due to reduced number of medical events and more favorable physical and cognitive function. The effects of the two interventions upon HRQL are further nuanced by the possibility that some participants in either treatment arm may adjust to decrements in health status by changing their internal perception of favorable HRQL, known as "response shift".

There may also be potential health cost tradeoffs of the intensive versus standard treatment. While the intensive arm is anticipated to result in higher short-term costs due to more frequent office visits and greater medication use, this arm may also result in lower long-term costs from event-related hospitalizations and other medical costs if the treatment approach is efficacious in reducing these medical events. Assuming the primary outcomes are as hypothesized, examining the HRQL and cost-effectiveness of the intensive and standard treatment arms will be important determinants of the potential adoption of the intensive BP control in clinical practice, and will be informative in identifying subgroups of patients for whom intensive or standard BP control is most appropriate.

7.2. Hypotheses

7.2.1 HRQL Hypotheses

The hypotheses generated for the HRQL measures are:

- Overall HRQL (Entire sample, Veterans RAND-12) Intensive control of blood pressure compared to standard control will result in worse HRQL at the 1-year assessment, but better HRQL at the 5-year assessment. The effect will be greater in those with lower baseline HRQL and greater number of comorbid conditions at baseline.

- Falls Self-efficacy (Subsample, Falls Self Efficacy Scale) Intensive control of blood pressure compared to standard control will result in less favorable fall-related self-efficacy at the 1-year assessment. The effect will be the greater in older participants, those with lower baseline HRQL, and those with a greater number of baseline comorbid conditions. By Year 5, intensive control of blood pressure will result in more favorable fall-related self-efficacy compared to standard control.

- Sexual function (Subsample, Modified Female Sexual Function Assessment /International Index of Erectile Function) Intensive control of blood pressure compared to standard control will decrease sexual function among men and women participants at one year. By year 5, the intensive treatment participants will report more favorable sexual function compared to participants in the standard treatment.

7.2.2 Cost-Effectiveness Hypotheses

The primary hypotheses generated for the economic and cost-effectiveness analyses are:

- Intensive control of blood pressure compared to standard control will result in higher healthcare costs and utilization in the first year due to the greater number of office visits, medications, and lab tests likely required to achieve the intensive control targets.

- Intensive control of blood pressure compared to standard control will result in lower healthcare costs and utilization over the study period due to decreased events and related health costs among intensive control participants.

- The incremental cost-effectiveness ratio will be ≤$100,000/Quality Adjusted Life Years (QALY) gained when compared to the standard intervention.

7.3. Health-Related Quality of Life Measures

7.3.1 Rationale for Selection

The SPRINT HRQL instruments were selected based upon the following criteria: (1) inclusion of the major dimensions shown in the literature to be affected by hypertension and its treatment; (2) brevity; (3) responsiveness to treatment-related changes, and (4) appropriateness for the age range, racial/ethnic diversity, and anticipated medical conditions of the participants in SPRINT.

To reduce participant burden, some HRQL instruments will be administered to the entire SPRINT sample, while others will be administered only in a subsample of participants. All HRQL instruments will be self-administered unless participants require assistance due to sensory, motor, or cognitive deficits in which case the instruments will be administered by clinic staff or family/friends accompanying the participant to the clinic visit. For Spanish-speaking participants, Spanish versions of all HRQL instruments will be administered to participants at all assessment points who indicate at baseline that

they do not have sufficient written English fluency to complete the instruments in English.

7.3.2 Health-Related Quality of Life (HRQL) Measures

Veterans RAND 12-item (VR-12) questionnaire. The VR-12 is a shorter version of the VR-36 (which is derived from the SF-36). Changes of the VR-12 relative to the SF-12 have lowered the floor and ceiling, improved the distributional properties, increased reliability, and improved discriminant validity of the physical and mental health summary scores. Validated conversion formulas allow for direct comparisons to prior studies using the SF-36 or SF-12. The VR-12 will be administered to all SPRINT participants at baseline and at annual visits thereafter, as well as at the close-out visit.

Fall Self-Efficacy Scale International (FES-I) The FES-I, shortened version, consists of seven items which the respondent answers on a 1-4 scale, indicating level of concern for falling. The activities are getting dressed or undressed, taking a bath or shower, getting in or out of a chair, going up or down stairs, reaching for something above your head or on the ground, walking up or down a slope, and getting out to a social event. An evaluation of the Short FES-I found good internal and 4-week test-retest reliability. The correlation between the Short FES-I and the FES-I was 0.97. The Short FES-I will be administered among a subsample of SPRINT participants.

International Index of Erectile Function (IIEF) The IIEF-5 is the 5-item short form of the original 15-item IIEF, and was developed specifically for use in clinical settings to supplement physical examination and patient history. IIEF-5 scores can be classified into the following categories; severe erectile dysfunction (ED), moderate ED, mild to moderate ED, mild or no ED. Scores less than 21 have 98% sensitivity and 88% specificity for the presence of ED. The IIEF-5 will be administered in a male subsample of SPRINT participants.

Female Sexual Function Assessment (FSFI) The FSFI is a 19-item survey that assesses female sexual function over the past four weeks in 6 domains (desire, arousal, lubrication, orgasm, satisfaction, and pain). Utilizing recently proposed modifications to the FSFI, participants not sexually active over the past four weeks would complete only 4 items, substantially reducing respondent burden. The FSFI has high internal consistency (Cronbach alpha > 0.8). This assessment will be administered in a female subsample of SPRINT participants.

Patient Satisfaction (Bharmal and others, 2009) A modified Treatment Satisfaction Questionnaire for Medication (TSQM) General Satisfaction subscale will be administered at baseline (based on current blood pressure medications being taken, if any) and at 1 and 4 years (or close-out for those participants who have not reached the 48M visit at the time of close-out). This corresponds with the administration of the Morisky Adherence scale, which will allow for analyses of the relationship between satisfaction and adherence at these time points.

Patient Health Questionnaire-9 (PHQ-9) The PHQ-9 is a self-report measure of depression that has been recommended by the AHA Advisory Panel on Depression and Coronary Heart Disease, has a low response burden (9 items; 2-3 minutes to complete), excellent reliability, and good sensitivity and specificity with depression diagnoses. This

assessment will be done at baseline, annually, and at the close-out visit on all participants.

7.3.3 Health State Utility Measures

EQ-5D is a self-administered 5-item instrument including mobility, self-care, usual activities, pain/discomfort and depression. There are three responses to each question (no, moderate, or severe limitations). This commonly used measure of health utilities will be used to convert quality of life and health status into quality adjusted life-years (QALYs) for cost-effectiveness analysis. The EQ-5D will be administered to all participants at baseline, annually and at the close-out visit.

7.4. Cost-Effectiveness Assessment

7.4.1 Rationale

It is expected that the intensive therapy for hypertension will not only reduce cardiovascular events but will be more cost-effective over the long-term. The two primary measures of cost-effectiveness are the incremental cost per QALY and life-year gained. The primary cost-effectiveness hypothesis is that the intensive blood pressure treatment will be cost-effective as compared to the standard treatment. This question will be addressed by conducting incremental cost-effective analyses in which the net costs and net effectiveness of intensive therapy defined by the main trial to standard therapy will be calculated and expressed as a series of ratios.

For QALYs, the cost-effectiveness hypothesis is that the ratio of costs per QALY (as measured by the EQ-5D) will be significantly less (i.e., more favorable cost-effectiveness) for the intensive intervention than for the standard intervention. Costs will be discounted to weigh future costs less heavily than present ones.

7.4.2 Effectiveness

The primary endpoints defined by the main trial are considered as primary outcome measures for this economic evaluation. The primary effectiveness measures will be life-years gained and QALY gained. The measure of life-year gained is determined by the difference in number of life–years between intensive therapy and standard therapy. QALYs adjust life-years gained by the quality of the participant’s overall HRQL during these life-years gained.

7.4.3 Costs

All direct medical costs associated with treatment of hypertension and its complications and costs for treating adverse effects of the therapy will be considered. These costs will include costs of inpatient care, outpatient care, medications, medical equipment, supplies, laboratory tests, and professional services. The participant's costs such as waiting time, transportation, lodging, and informal care arising from the disease will not be included. Likewise, opportunity costs of premature death, productivity loss, and long-term disability will not be considered in this study.

7.4.3.1 Cost Data Collection

Hospitalizations are the primary cost drivers in most cost-effectiveness analyses, and SPRINT has proposed obtaining hospitalization events via multiple sources. Patient report of hospitalizations, along with emergency department (ED) visits, stays in rehabilitation facilities, and day-surgery admissions, are obtained every 3 months during scheduled SPRINT study visits. Discharge summaries and other pertinent records (including reason for hospitalization and length of stay) will be obtained from hospitalizations, Emergency Department visits, rehabilitation stays, and day-surgery admissions related to outcome events and potential adverse events (including cardiovascular, renal, and cerebrovascular disorders; dementia; falls) which will constitute many of the admissions that might be expected to differ by arm. Because of the large proportion of VA and Medicare patients in SPRINT, we also will be able to determine hospitalizations, dates of admission, length of stay, and reason for admission via Medicare and VA databases for those hospitalizations for which we do not have discharge summaries. For the limited number of remaining patient reports for which we have neither discharge summaries nor database information, we will perform regression analyses of reported vs. actual length of stay and costs for all those with such data to estimate the costs of the undocumented hospitalizations. Cost estimates for hospitalizations will be based on DRG-specific Medicare cost weights. For professional costs associated with hospitalizations we plan to obtain these costs from Medicare and VA databases as available in a subsample and use these data to estimate professional costs for the entire sample based on these subsample analyses. We will also explore whether these databases allow us to obtain costs associated with ED visits, stays in rehabilitation facilities, and day surgery admissions.

7.4.3.2 Intensive and Standard Therapy Non-Research Costs

For medications, we plan to use study medication logs to obtain the medications prescribed by the study. This log also includes blood pressure lowering medications prescribed by other healthcare providers. Medication costs will be estimated using median wholesale price. We will obtain information on non-study prescribed medications (concomitant medications) from participants annually and will estimate costs for these medications based on the most commonly used doses in clinical practice. We will not obtain cost data on non-study related labs, as this source of utilization is not expected to differ by group. To estimate non-research related costs for the SPRINT office visits, we plan to obtain estimated CPT codes (minus research-specific activities) from clinic staff for a random subset of these visits to estimate costs via Medicare payment rates. Non-study outpatient visits will not be obtained but will be estimated with non-study medication costs by age using national health care expenditure data.

7.4.3.3 Data Analysis for Cost-Effectiveness

Two methods of cost-effectiveness analysis (CEA) and cost-utility analysis (CUA) will be used in the economic evaluation. The ratios of cost to outcome derived from CEA/CUA are used to compare cost-effectiveness among treatment strategies. An incremental cost-effectiveness ratio (ICER) will be calculated, which provides a summary of the cost-effectiveness of one intervention relative to the other.

The basic formula to calculate incremental CEA ratio and CUA ratio of a specific treatment A relative to the reference treatment B is presented as following:

$$ICER_{CEA} = \frac{(\text{Mean Cost}_{\text{treatment A}} - \text{Mean Cost}_{\text{treatment B}})}{(\text{Mean Effect}_{\text{treatment A}} - \text{Mean Effect}_{\text{treatment B}})}$$

$$ICER_{CUA} = \frac{(\text{Mean Cost}_{\text{treatment A}} - \text{Mean Cost}_{\text{treatment B}})}{(\text{Mean QALY}_{\text{treatment A}} - \text{Mean QALY}_{\text{treatment B}})}$$

The ratio of incremental cost to incremental effectiveness represents cost-effectiveness of the intensive BP treatment. Bootstrap methods will be used to calculate confidence intervals for cost-effectiveness ratios. All costs will be adjusted to the baseline year using the medical component of the Consumer Price Index. Future costs and outcomes will be discounted by 3%. Estimates of utilization over time will be adjusted for the presence of censored data with variable follow-up. Sensitivity analysis will explore the effect of correlations between costs and outcomes, which will also be empirically examined in the cost and outcome data.

QALYs will be calculated by summing the area under each individual's QALY curve (constructed by plotting the EQ-5D scores for each interview during follow-up). The estimates of mean differences in costs and outcomes – which will be used to create net health benefits and the cost per QALY ratios -- will be derived from multivariable regression analyses. For the evaluation of the difference in costs, the dependent variable in the regression will either be costs or the natural log of costs (determination of the form of the dependent variable will be based on statistical tests of its distribution). If the dependent variable used in the analysis is the log of costs, a smearing retransformation will be used to estimate the absolute difference in costs between the treatment groups.

Chapter 8 – Safety Monitoring and Reporting

8.1 Introduction

The SPRINT trial is testing whether lowering SBP to a goal of <120 mm Hg results in better outcomes than a goal of <140 mm Hg in patients at risk for CVD events. SPRINT is not a study of specific anti-hypertensive agents. All antihypertensive agents provided by the trial or recommended by SPRINT have been approved by the Food and Drug Administration (FDA) and are routinely prescribed for lowering blood pressure.

Patient safety will be carefully monitored in SPRINT. Each participating investigator has primary responsibility for the safety of the individual participants under his/her care. In addition, an independent Data and Safety Monitoring Board (DSMB) will have primary responsibility for monitoring the accumulating study data for signs of adverse trends in morbidity/mortality and treatment-related serious adverse events.

8.2 Participant population

Participants enrolled in SPRINT have elevated risk for CVD outcomes. Inclusion and exclusion criteria for SPRINT were set in order to maximize safety while facilitating inclusion of a trial population at risk for the major trial outcomes. Exclusions are outlined in Section 3.1.

Potentially Vulnerable populations: The SPRINT population includes a significant proportion of older adults (>75 years), some of whom may become cognitively impaired during the course of the trial. Thus, participants are asked to identify a contact person at the time of enrollment that can provide information about the participant as it relates to the study. In addition, participants with CKD may need care coordination or referral to a nephrologist during the study. Various management issues in patients with eGFR values lower than 30 ml/min/1.73m^2 may arise including dietary issues and the effects of CKD on pharmacokinetics, pharmacodynamics and side-effects of various drugs. All participants, including those with CKD, will be managed according to current national guidelines. If patients with this level of renal impairment are not already followed by a nephrologist and the investigator feels it is needed, he/she will coordinate with the participant's primary care physician regarding the recommendation for renal follow-up.

8.3 Safety Monitoring

Several types of safety issues and serious adverse events may occur in SPRINT and participants will be monitored for these regularly throughout the study.

8.3.1 Expected Events:

The potential adverse effects of the blood pressure drugs used in SPRINT have been well documented. For example, electrolyte abnormalities (hyponatremia or hypokalemia are known to be associated with diuretics; hyperkalemia and short-term decline in GFR with RAAS blockers, hyperkalemia with potassium-sparing drugs; as well as bradycardia with beta blockers and calcium channel blockers). Participants will be monitored routinely with interviews, vital signs, targeted physical examination and laboratory tests to ensure safety (Chapter 5, Table 5.1). In addition, site clinicians may also obtain local

labs and ECG's if safety is a concern at non-scheduled intervals. Clinical alerts are generated when safety parameters are exceeded. (Table 8.1). Expected events are not considered serious adverse events (SAEs) unless they meet criteria for an SAE (see 8.3.2).

Table 8.1 Clinical Safety Alerts

**Measure**	**Alert Value**
Serum sodium	< =132 or >150 mEq/L
Serum potassium	<3.0 or >5.5 mEq/L
Serum creatinine	Increase by at least 50% to a value >=1.5 mg/dL since the last study lab (usually 6 months apart).
Heart rate	<40
ECG	acute MI, complete heart block, or bradycardia <40 bmp
PHQ-9 (depression screen)	Positive response to question on suicidal ideation
Dementia Assessment	Adjudicated dementia

8.3.2 Adverse Events and Serious Adverse Events

An adverse event (AE) is defined as any untoward or unfavorable medical occurrence in a human subject, including any clinically significant abnormal sign (for example, abnormal physical exam or laboratory finding), symptom, or disease, temporally associated with the subject's participation in the research, whether or not considered related to the subject's participation in the research. The burden of collecting and reporting data on every possible AE in SPRINT is excessive and side effects from the drugs to be used in SPRINT have been well defined in previous studies. Therefore, in SPRINT, sites will report all serious adverse events and selected AEs to the Coordinating Center.

Consistent with NHLBI guidelines and OHRP policy, SAEs are adverse events that meet any of the following criteria:

- fatal or life-threatening,
- result in significant or persistent disability,
- require or prolong hospitalization,
- result in a congenital anomaly/birth defect, or
- are important medical events that investigators judge to represent significant hazards or harm to research participants and may require medical or surgical intervention to prevent one of the other outcomes listed in this definition (e.g. hospitalization, death, persistent disability).

Any adverse event that meets any of these criteria will be documented and reported as a serious adverse event. In addition, a select list of other important events (see manual of procedures for details and definitions), regardless of whether they resulted in hospitalization, will also be considered SAEs in SPRINT, including:

- Injurious falls
- Syncope
- Unexpected events for which the investigator believes that the SPRINT intervention caused the event or contributed to the immediate cause of the event

Participants will be queried for SAEs and selected AEs at quarterly clinic visits.

8.3.3 Modification of treatment in response to safety concerns

SPRINT is testing two different SBP treatment goals. The study physician may add, increase or reduce the dose, stop, or change antihypertensive drugs in the interest of participant safety. Depending on the situation, the change may be temporary or permanent. Situations that may require temporary reduction or elimination of a study medication include: side effects, worsening congestive heart failure, acute kidney injury, symptomatic hypotensive episodes, and other illnesses. Orthostatic hypotension is usually related to specific drug classes and not BP level per se and thus should NOT usually alter target blood pressure goals. The MOP contains a section on management of symptomatic orthostatic hypotension.

8.4 Safety Reporting

8.4.1 Clinical Safety Alerts

Clinical Safety Alerts (section 8.3.1. and Table 8.1) are provided to the site clinician for his/her action. When any laboratory measurement attains a defined alert level, the Central Laboratory will immediately notify the clinical site and the CCN. Site clinicians may also obtain local labs if safety is a concern at non-scheduled intervals. Site clinicians are responsible for timely review of all labs drawn locally and when central lab results become available. ECGs will be done at specified visits and read by the ECG reading center. However, if a participant has one of a short list of abnormalities (reported on the ECG by the machine), such as acute MI, complete heart block, or bradycardia <40 beats/minute, the ECG will be reviewed by the site clinician immediately (see ECG section of the SPRINT MOP).

8.4.2 Serious Adverse Events

At each quarterly visit, SPRINT staff will specifically query participants for serious adverse events. In addition, information on serious adverse events may also be reported to study staff spontaneously by participants through telephone calls or emails between study visits. In addition to local reporting requirements, all serious adverse events will be recorded by clinic staff and forwarded to the CC Medical Safety Officer **within 72 hours** of knowledge of the event. SAEs will be collected and reported from screening to the end of the study follow-up period for an individual participant. SAEs will be followed until resolution, stabilization, or until it is determined that study participation is not the cause.

The Coordinating Center will be responsible for timely reporting to the NIH and the DSMB. The Coordinating Center will provide reports of serious adverse events for review by the DSMB at their meetings.

8.5 Data Safety Monitoring Board

A **Data Safety Monitoring Board (DSMB)** is established, with responsibility to monitor all aspects of the study. The **Medical Safety Officer** reports to the DSMB for issues related to participants' safety. This independent Data and Safety Monitoring Board will be established to monitor data and oversee participant safety. Members will be appointed by the NHLBI to provide oversight of the trial and its ancillary studies. The SPRINT DSMB may include experts in cardiovascular medicine (particularly hypertension), kidney disease, clinical trials, geriatrics, biostatistics, quality of life, cost effectiveness, cognitive function and other areas as needed. DSMB participants include the Steering Committee Chair and Vice-Chair, CC PI and senior staff, and representatives from the NHLBI and other NIH sponsors. The DSMB normally meets twice a year to monitor safety, to advise the NHLBI about study progress and performance, and to make recommendations to the NHLBI regarding study continuation and protocol changes. In addition, the CC may provide data to the DSMB Chair to ensure early identification of any major adverse outcomes of therapy. The DSMB has the responsibility to recommend to the NHLBI whether the trial should continue, whether the protocol should be modified, or whether there should be early termination. The DSMB will provide reports to the NHLBI through the Executive Secretary, who will be appointed by the NHLBI. Recommendations by the DSMB must be approved by the NHLBI prior to implementation.

Chapter 9 – Clinical Outcome Measures

9.0 Outcomes

This chapter describes the SPRINT primary and secondary clinical outcomes. Clinical events occurring during follow-up will be ascertained primarily through surveillance of self-reported events, laboratory, and ECG data collected by the study and classified by members of the Morbidity and Mortality subcommittee masked to treatment assignment. Additional sources, including searches of the National Death Index (NDI), will also be used to augment follow-up data.

9.1 Primary Outcome

The primary outcome measure for SPRINT will be major CVD events, defined as the composite endpoint comprised of the first occurrence of a

- fatal or non-fatal myocardial infarction (MI),
- non-MI acute coronary syndrome (non-MI ACS),
- fatal or non-fatal stroke,
- fatal or non-fatal heart failure (HF), or
- death attributable to cardiovascular disease (CVD).

MI and non-MI ACS are defined in Section 9.1.1; stroke is defined in Section 9.1.2; HF is defined in Section 9.1.3, and CVD death is defined in Section 9.1.4. The SPRINT Manual of Procedures contains the full details of these definitions.

9.1.1 MI and Non MI ACS

9.1.1.1 MI: Defined as the death of part of the myocardium due to an occlusion of a coronary artery from any cause, including spasm, embolus, thrombus or rupture of a plaque. SPRINT will use standard case definitions for both fatal and nonfatal MI based on the combination of symptoms, elevation in biomarkers, and/or ECG findings. The algorithm for classifying MI includes elements of the clinical presentation (signs and symptoms), results of cardiac biomarker determinations, and ECG readings, and is based on a 2003 Scientific Statement (Luepker and others, 2003). The definition includes MI that occurred during surgery/procedure and MI aborted by thrombolytic therapy or procedure. SPRINT adjudicators will be guided by specific, pre-specified definitions and operational rules. Adjudicators will use their clinical interpretation of the ECGs and other available evidence for the event to classify MI cases as definite, probable, or possible, with all included in the primary outcome (Luepker and others, 2003). MI will be ascertained both from adjudication of hospital records for clinical events and also from the finding of new significant Q waves from the standardized interpretation of the study visit-obtained ECG (silent or unrecognized MI). MIs that present clinically will include Q wave, ST elevation and non-ST elevation infarctions (segment elevation myocardial infarction (STEMI) and Non-ST Segment elevation myocardial infarction (NSTEMI), as well as aborted MI and post-intervention MI.

9.1.1.2 Non-MI ACS: Defined as hospitalization for evaluation and treatment of an accelerating or new symptom pattern consistent with coronary artery insufficiency without meeting the definition of MI, but requiring evaluation to rule-out MI on clinical presentation. Non-MI ACS in SPRINT will also require objective findings of coronary ischemia, including any of the following: history of previous catheterization with

significant obstruction or previous revascularization; significant obstructive lesion(s) on coronary catheterization during index hospitalization and/or intervention for revascularization; ischemic ECG changes or imaging findings on exercise or pharmacologic stress testing associated with the index hospitalization; or resting ECG findings consistent with ischemia occurring with symptoms during the index hospitalization.

9.1.2 Stroke

9.1.2.1 Stroke: SPRINT will use standard case definitions for both fatal and nonfatal stroke. Stroke will be defined based on all available data, including symptoms and signs, imaging of the brain and large vessels, and cardiac testing, e.g., echocardiography. Adjudicators will use their clinical judgment based on the available evidence to classify each case, and will be guided by pre-specified definitions and operational rules. Stroke is generally defined as neurological deficit of cerebrovascular cause that persists beyond 24 hours or is interrupted by death within 24 hours (World Health Organization, 1978 Cerebrovascular Disorders (Offset Publications). Geneva: World Health Organization. ISBN 9241700432. Exclusionary conditions for stroke include major brain trauma, intracranial neoplasm, coma due to metabolic disorders or disorders of fluid or electrolyte balance, peripheral neuropathy, or central nervous system infections. Stroke will be classified as brain infarction, subarachnoid hemorrhage, intraparenchymal hemorrhage, other hemorrhage, other type, or unknown type. In SPRINT, brain infarction (ischemic stroke) is defined as a new lesion detected by computed tomography or magnetic resonance imaging or, in the absence of a new lesion on available imaging, clinical findings consistent with the occurrence of stroke that lasted for more than 24 hours (N Engl J Med 2001;345:1444-51). Brain infarctions will be further sub-typed using the Causative Classification of Stroke system as evident, probable, or possible cases of large artery atherosclerosis, cardio-aortic embolism, small artery occlusion, other causes, and undetermined causes (Ay and others, 2007). Strokes following invasive cardiovascular interventions will also be classified as such.

9.1.3 HF

9.1.3.1 HF: Defined as hospitalization, or emergency department visit requiring treatment with infusion therapy, for a clinical syndrome that presents with multiple signs and symptoms consistent with cardiac decompensation/inadequate cardiac pump function. Adjudication will use the ARIC study adjudication system (Rosamond and others, 2009). The SPRINT HF outcome will include definite or possible acute decompensation, including HF with preserved left ventricular ejection fraction as well as HF with reduced ejection fraction. HF cases may also be adjudicated as chronic stable HF but this is not considered a SPRINT outcome. In SPRINT, HF will include a variety of clinical presentations, including acute or subacute HF as the primary reason for hospital admission or for emergency department visit where HF was diagnosed and intravenous treatment was given. The identification and classification of HF cases will rely on multiple pieces of key clinical data as well as adjudicators' clinical judgment, guided by specific, pre-specified definitions and operational rules. No identification of HF should rely on a single piece of data such as the presence of dyspnea or of edema, a low ejection fraction, or an increased brain naturetic peptide (BNP) value. Adjudicators will use both the data available and clinical judgment to distinguish between "definite" and "possible" decompensated HF. "Definite" decompensated HF will be assigned when

decompensation is clearly present based on available data (satisfies criteria for decompensation). “Possible” decompensation will be assigned when decompensation is possibly but not definitively present, typically where the presence of co-morbidity could account for the acute symptoms (chronic obstructive pulmonary disease (COPD) exacerbation, for example).

For participants with advanced CKD with or without chronic dialysis, the ascertainment of HF can be particularly difficult, since the fluid overload can be purely the consequence of fluid retention by the kidney or absence of kidneys. Under these circumstances, the adjudicators will again use their best judgment, utilizing all available information.

9.1.4 CVD Death

9.1.4.1 CVD Death: SPRINT will use standard case definitions for classification of CVD death. Definite CVD events will be defined based on temporal relationship to a documented event (e.g., hospitalization for MI or for stroke), or postmortem findings of an acute CVD event. Probable coronary heart disease (CHD) death (Luepker, 2003) will be defined based on autopsy findings consistent with chronic CHD, prior history of CHD or documented symptoms consistent with CHD prior to death, and the absence of another likely cause of death. Possible fatal CHD will be adjudicated based on death certificate information consistent with an underlying CHD cause and no evidence of a non-coronary cause. Stroke deaths will be categorized based on the temporal relationship between the stroke event and death, in cases where the underlying cause of death is attributed to stroke. Proximal stroke death is a death attributed to stroke and occurring within 30 days of stroke; remote stroke death is underlying cause attributed to stroke and more than 30 days from stroke to death. Other forms of CVD death will also be adjudicated and include ruptured abdominal aortic aneurysm, and documented arrhythmia.

9.2 Secondary Outcomes

In addition to the primary outcome, SPRINT will assess additional clinical outcomes in order to more fully evaluate the relative effects of treating to a SBP goal lower than the currently recommended goal. In order to do so, data will be collected on secondary and other trial outcomes. Main secondary outcomes are included in the analysis plan in Chapter 10.

9.2.1 Main secondary cardiovascular composite outcome: The main secondary composite outcome of SPRINT is comprised of the first occurrence of any of the components of the primary outcome and all cause mortality. A major and analogous secondary outcome of CVD-free survival, defined as survival without any of the primary or secondary CVD outcomes, will also be examined because of the significant proportion of elderly in the trial and the public health importance of the issue of CVD in that age group. All cause mortality and components of the primary outcome will also be examined.

9.2.2 Main secondary renal outcome: The main secondary renal outcome of SPRINT will be the composite of a 50% decrease in eGFR or development of ESRD requiring chronic dialysis or kidney transplantation. This outcome applies to the CKD subgroup only.

9.2.3 Main secondary cognitive outcomes: SPRINT MIND will evaluate the incidence of all-cause dementia as adjudicated by an expert panel as the most important outcome for the MIND study. The second most important outcome is cognitive impairment among the Extensive Cognitive Assessment Battery participants will be tested with the full assessment battery (6.4.1.3 and 6.6.2). Each test score from the full assessment battery will be classified as indicating "impairment (1)" or "no impairment (0)" based on norms. A sum of impairment scores will be calculated indicating the total number of impairments. Detailed definitions of these outcomes are contained in chapter 6.

9.2.4 Additional secondary outcomes: In addition to the secondary outcomes specified in Chapter 10, other outcomes will also be examined separately and combined with other outcomes in composites (e.g., CVD-free survival defined above):

- Peripheral arterial disease, including carotid and peripheral revascularization, abdominal aortic aneurysm repair, and other objectively defined PAD events
- Coronary revascularization
- Transient Ischemic Attack (TIA): TIA in SPRINT will be defined as one or more transient episodes of the sudden onset of a focal neurological deficit, no lesion on brain imaging consistent with the deficit, and no signs or symptoms consistent with seizures, migraine, or other non-vascular causes.
- ECG diagnosed Left Ventricular Hypertrophy (LVH): ECG-diagnosed LVH will be defined primarily using the sex-specific Cornell voltage criteria. Other ECG-LVH criteria mentioned in the American Heart Association (AHA)/American College of Cardiology (ACC) statement on ECG changes associated with cardiac chamber hypertrophy (Hancock and others, 2009) will be also considered.
- Atrial fibrillation or flutter: In SPRINT, atrial fibrillation/flutter will be primarily detected from the scheduled study ECGs using Minnesota ECG classification (Minnesota code 8.3). Other sources of detection include hospital discharge ICD code (ICD-10 code 148 or ICD-9 code 427.3) and self-report.
- Other renal outcomes
    - Incident CKD, defined as a >30% decrease in eGFR and an end value of <60 ml/min/1.73M^2. This outcome applies only to the non-CKD subgroup. This decrease in eGFR requires a confirmatory value in the next available official SPRINT lab check.
    - Incident albuminuria, defined as a doubling of urinary albumin-to-creatinine (ACR) ratio from a value <10 mg/g to a value of >10 mg/g. This outcome applies to CKD and non-CKD subjects. This increase in ACR requires a confirmatory value in the next available official SPRINT lab check.

Chapter 10 – Statistical Considerations

The SPRINT Trial has a single primary objective and several key secondary objectives, some of which will be addressed within a number of subgroups whose target size has been guided by power computations. The primary objective is to determine whether the intensive BP treatment strategy will, when compared to a standard BP treatment strategy, reduce the incidence of serious cardiovascular events, defined as MI, stroke, heart failure, non-MI acute coronary syndrome or other cardiovascular death. This will be tested in all SPRINT participants.

The key secondary objectives are to determine whether the intensive BP strategy reduces the incidence of:

1) total mortality,
2) progression of CKD,
3) probable dementia,
4) cognitive impairment, and
5) white matter lesions detected by MRI.

The primary analysis of each of these objectives will be in different groups of participants. The analysis plan to address the primary and each secondary objective is described below, followed by estimates of the required sample size for each.

10.1 Analysis Plan

This section describes some of the key pre-specified analyses directed at the study's primary and key secondary objectives. Many other outcomes and measurements, such as blood pressure, adverse event experiences, health related quality of life, cost, and results of assays performed on blood and urine specimens will also be analyzed.

10.1.1 Analysis of the Primary Outcome in all Randomized Participants

The primary analysis will apply Cox proportional hazards regression (Cox, 1972) to all randomized participants to compare the time from randomization to the first occurrence of the primary CVD composite endpoint between the randomized BP groups. The model will include an indicator for intervention arm as its sole predictor variable. Clinical site at randomization will be a stratifying factor. Follow-up time will be censored at the last date of event ascertainment. The p-value from the primary analysis will be based on the chi-square statistic from a likelihood ratio test obtained from proportional hazards models with and without the term for intervention arm. This likelihood ratio test will constitute the primary test of statistical significance for the primary analysis.

Primary comparisons of intervention groups will be performed according to the intention-to-treat principle. All randomized participants in these analyses will be grouped according to their intervention assignment at randomization, regardless of adherence.

10.1.2 Secondary analyses supporting the primary analysis

10.1.2.1 Secondary outcomes. A number of secondary outcomes will be analyzed to clarify the interpretation of the results of the primary analysis. These will include:

a) all myocardial infarction,

b) all stroke,
c) non-MI acute coronary syndrome,
d) all heart failure,
e) CVD mortality,
f) total mortality, and
g) a composite of total mortality and the primary composite outcome (i.e. major CVD event- free survival).

Each of these will be analyzed using a proportional hazards model as described for the primary analysis. These will be reported with 95% confidence intervals and nominal p-values without an adjustment for multiple comparisons, since the intent is to articulate a pattern of effects closely related to the primary outcome, rather than to provide additional tests of efficacy.

10.1.2.2 Subgroup analyses. In addition to the analysis of the secondary outcomes described above, a set of analyses will be reported to explore whether intervention effects on the primary and confirmatory secondary outcomes are consistent across subgroups of interest. These subgroups are:

a) CKD (defined as eGFR < 60 at randomization) vs. non-CKD,
b) senior vs. non-senior (aged $\geq$75 at randomization vs. aged <75),
c) male vs. female,
d) black vs. non-black,
e) with and without a history of CVD at randomization (as defined in Chapter 3), and
f) tertiles of systolic blood pressure at baseline.

The subgroups defined by CKD, age and race are motivated by biologically plausible hypotheses. For each subgroup analysis, a proportional hazards model will be used that is similar to the one described for the primary analysis above, but with additional terms identifying subgroup membership and the intervention by subgroup interaction. The nominal p-value for the interaction term using a likelihood ratio test will be reported along with within subgroup estimates of the intervention effect and associated nominal 95% confidence intervals. We will report the Hommel adjusted p-values for the interaction effects.

10.1.3 Non-cardiovascular clinical outcomes

10.1.3.1. Acute vs. chronic effects of intervention

It is possible that the intervention will have some acute adverse effects due to under-perfusion of various organs, notably the kidney and the brain, which are major targets of SPRINT. In the long term, however, lower SBP may protect these organs from hypertension-related damage. We will examine the possibility of acute effects as part of the data monitoring plan, particularly if differential adverse effects are observed early in the trial; we also will examine the possibility of acute effects as part of the data analysis at the end of the trial.

10.1.3.2 Renal outcomes

Renal outcomes are of particular importance in SPRINT, both to assess the incidence of new kidney disease among participants free of CKD at baseline and to assess the progression of kidney disease among those with CKD at baseline. Because some

outcomes are more interpretable in either people with CKD or without CKD at baseline, some analyses will be restricted to these subgroups.

The primary hypothesis for the renal outcomes is whether, in the subgroup with CKD at baseline, the rate of a composite of a 50% decrease in eGFR or ESRD undergoing chronic dialysis or kidney transplantation is lower in the intensive intervention arm. The decline in eGFR must be seen on two visits at least three months apart. This will be analyzed using a proportional hazards model as described for the primary CV analysis.

A number of additional analyses related to this hypothesis will also be performed. These will include:

a) incident CKD in the non-CKD subgroup, defined as a 30% decline from baseline eGFR to a value of <60 mL/min/1.73m^2 (observed on two visits at least 3 months apart. There must be a decrease of at least 30% AND the end value of this decrease must be <60 ml/min/1.73m^2 in order to satisfy this endpoint criterion) or ESRD
b) incident albuminuria, defined as a doubling of urinary albumin-to-creatinine (ACR) ratio from a value <10 mg/g to a value of >10 mg/g. This outcome applies to CKD and non-CKD subjects. This increase in ACR must be observed at two visits at least 3 months apart.

Subgroup analyses. Analyses of the renal outcomes will be by CKD and non-CKD strata. Within each strata, assessments of the renal composite endpoint will be by subgroups. The analytical approach will be the same as for the primary CV analysis as described in 10.1.2.2. The renal subgroups are:

a) urinary albumin/creatinine ratio (>300 mg/g and ≤ 300 mg/g),
b) black vs. non-black,
c) senior vs. non-senior (aged 75+ at randomization vs. aged <75),
d) male vs female,
e) eGFR (median split)

The subgroups defined by albumin/creatinine ratio, age and race are motivated by biologically plausible hypotheses. The main renal outcome composite is defined differently for the CKD and non-CKD strata, so that these will be separate analyses.

10.1.3.3 Dementia and cognitive outcomes.

The primary outcome for SPRINT MIND will be the first identification of adjudicated dementia. Cox proportional hazards models (as described above for the SPRINT primary outcome) will be used to compare the time from randomization to the first identification of dementia between the two treatment arms. All participants will be screened for dementia at baseline.

Secondary analyses. Secondary analyses in the areas of cognitive function, small vessel ischemic disease (SVID) lesion load, and mild cognitive impairment will also be performed to support the primary analysis.

Cognitive Function. A cognitive assessment battery will be administered at baseline and 2 and 4 years post-randomization and at the close-out visit (if the year 4 testing has not been completed) in a subsample of 2800. The primary outcomes will be composite scores for two domains: 1) Memory, consisting of the Hopkins Verbal Learning Test, Logical Memory and the Modified Rey Osterrieth Figure, and 2) Processing Speed,

consisting of Trails Making Tests and Digit Symbol Coding Test. Changes in impairment over time will be compared between the two treatment arms.

Supporting analyses will also be conducted on the effect of the interventions on individual domains of memory over 48 months. Follow-up test scores will be compared using mixed-effects analysis of covariance models (Laird, 1982). Mixed-effects models allow for departure from linearity in the relationship between the outcome and time. Estimates of the difference in mean levels of the outcome between control and intervention groups will be obtained using maximum likelihood techniques. Sensitivity of results to missing data will be investigated through the use of multiple imputation techniques (Rubin, 1987).

Magnetic Resonance Imaging (MRI). Other than age, hypertension is the strongest correlate of SVID. Total SVID lesion load including abnormal white matter, abnormal gray matter and abnormal basal ganglia will be the SPRINT measure of total SVID lesion load. Differences in total SVID lesion between treatment groups at 48 months will be the main outcomes of the MRI component. Furthermore, differences in total brain volume will also be compared after 48 months. These measures are continuous and will be analyzed using mixed effects analysis of covariance models as described above.

Mild Cognitive Impairment (MCI). This outcome is defined as the time to the first of two consecutive occurrences of MCI. Analytical methods used for dementia will be applied to the analyses of MCI, in those free of MCI at baseline. Furthermore, these same methods will be applied to the analyses of the first cognitive impairment defined as the first event classified either as MCI or dementia in those free of MCI at baseline.

Subgroups. Analyses of the cognitive outcomes will also explore the intervention effects within subgroups. The analytic approach will be the same as for the primary CV analysis as described in 10.1.2.2. The subgroups are:

a) CKD (defined as eGFR < 60 at randomization) vs. non-CKD,
b) senior vs. non-senior (aged 75+ at randomization vs. aged <75),
c) male vs. female,
d) black vs. non-black,
e) with and without a history of CVD at randomization (as defined in Chapter 3),
f) tertiles of systolic blood pressure at baseline,
g) MCI at baseline (yes vs. no),
h) orthostatic hypotension (yes vs. no).

The subgroups of CKD, age, and MCI are motivated by biologically plausible hypotheses.

10.1.4 Other analyses

We expect to explore fully the rich set of data that SPRINT will obtain. Exploratory analyses of biologically plausible subgroups are of particular interest. Some of these will be further articulation of supporting subgroup analyses described above, such as analysis of continuous baseline factors as continuous variables rather as pre-specified categorical variables. Other analyses will involve baseline variables that are not listed in the pre-specified subgroup but which may modify treatment effect, such as diastolic blood pressure or presence of the metabolic syndrome.

10.1.5 Missing data

Consistent with an intention-to-treat analysis, we will categorize all participants by their randomization group, regardless of compliance, in our primary analyses. For those participants lost to follow-up, we plan to use all available information until the time of death or loss to follow-up.

Our approach to handling missing outcomes in clinical trials is consistent with the opinion of Molenberghs and Kenward (2007, p9), who state that while ignorable, missing-at-random (MAR) analyses are reasonable for the primary analysis, exploration of the sensitivity of conclusion to the MAR assumption may include models which allow for missingness that is not random. If loss to follow-up is related to the level of the outcome being analyzed (e.g. as often occurs when analyzing health related outcomes), then results obtained under the assumption of independent loss to follow-up may be biased. The magnitude of this problem will be investigated by using measurements taken at previous visits to predict loss to follow-up. Variables determined to predict loss to follow-up will be included in our predictive models in order to satisfy the conditions described by Little and Rubin (1987) for the data to be considered MAR. Maximum likelihood techniques will be used to estimate parameters. If necessary, other approaches may be examined in consideration of how robust the results will be and whether they provide appropriately conservative estimates for the trial.

In order to explore the possibility of a relationship between ESRD and CV outcomes, we will conduct sensitivity analyses which treat ESRD as a censoring point for the primary outcome. This exploration may include an auxiliary composite outcome combining the events in the primary outcome and ESRD.

Robustness of inferences to missing outcome data will be further explored in sensitivity analyses. These analyses will include examination of several “worst-case” scenarios, including opposite and pooled imputation approaches (Wittes, Lakatos & Probstfield 1989; Proschan et al., 2001). These types of scenarios are members of a broad class that can be parameterized as pattern mixture models (Little 1993) and allow for examination of sensitivity of conclusions to missing-not-at-random (MNAR) mechanisms (Mohlenberg and Kenward, 2007).

The MRI substudy involves two assessments—one at baseline and one at 48 months—in 640 participants, thus limiting the range of analytic strategies. We recommend using maximum likelihood based general linear models for analyzing outcomes. Intracranial volume will be included as a covariate. The validity of the MAR assumption can be improved by including baseline covariates that predict missingness. If loss to follow-up is related to the unobserved cognitive outcome then our results may be biased. Again, some modeling and sensitivity analysis options may be considered if necessary.

10.2 Sample Size Estimation and Power Calculations

10.2.1 Primary Outcome

We have assumed a 2.2 %/yr event rate of the primary outcome in the standard group, a 20% effect size for the intervention (hazard ratio of 0.8), a two-year uniform recruitment period, a total study length of 5 years and 10 months, a 2 %/yr rate of loss to follow-up, and a two-sided test at the 5% level. With these assumptions, power for a variety of

sample sizes is presented in Table 1. Power is also presented for hazard ratios of 0.78 and 0.82 and for event rates of 2.0 and 2.4 %/yr. A sample size of 9250 provides high power for a hazard ratio of 0.8 (representing a 20% effect) and a 2.2 %/yr event rate. This sample size would also provide over 80% power for an effect of 18% (hazard ratio of 0.82) with an event rate of 2.2 %/yr and would have reasonable power of 77.3% even with a smaller than assumed event rate of 2.0 %/yr and an 18% effect. Depending on the observed event rate and treatment effect, the table below shows that sample sizes of 8500 to 10000 would be consistent with study goals.

Table 1: Power for the SPRINT primary outcome.

	Event Rate								
	2.0 %/yr			2.2 %/yr			2.4 %/yr		
N\Hazard Ratio	0.78	0.8	0.82	0.78	0.8	0.82	0.78	0.8	0.82
8500	89.4	82.7	73.7	91.9	85.9	77.6	93.9	88.6	80.9
8750	90.3	83.7	75.0	92.6	86.9	78.7	94.5	89.5	82.0
9000	91.0	84.7	76.1	93.3	87.8	79.8	95.0	90.3	83.0
**9250**	**91.7**	**85.7**	**77.3**	**93.9**	**88.7**	**80.9**	**95.5**	**91.0**	**84.0**
9500	92.4	86.6	78.3	94.4	89.4	81.9	95.9	91.7	85.0
9750	93.0	87.4	79.4	94.9	90.2	82.9	96.4	92.4	85.9
10000	93.6	88.2	80.4	95.4	90.9	83.8	96.7	93.0	86.7

If the event rate in the standard therapy arm is substantially less than 2.2%, we may ask that the DSMB consider recommending a two year extension of the trial.

10.2.2 Summary

For the primary outcome under the assumptions detailed below, with 9250 participants, the SPRINT study is designed to have

- 88.7% power to detect a treatment effect of 20% of intensive blood pressure control compared with standard blood pressure control,
- 81.9% power to detect a treatment effect of 20% of intensive blood pressure control compared with standard blood pressure control among participants with estimated glomerular filtration rates of <60 ml/min/1.73m^2 at baseline,
- 84.5% power to detect a treatment effect of 25% of intensive blood pressure control compared with standard blood pressure control among participants at least 75 years old at baseline,
- 96% power to detect a 20% effect and 80% power to detect a 15% effect for incident dementia, the primary outcome for SPRINT MIND.

These estimates of power are valid under the following assumptions:

- The primary outcome for SPRINT is a composite of fatal CVD, MI, stroke, heart failure, and non-MI acute coronary syndrome.
- The event rate for this composite outcome is
  - 2.2 %/yr in the standard BP arm,
  - 4 %/yr among participants with eGFR <60 ml/min/1.73m^2, and
  - 3.5 %/yr among participants ≥75 years old.
- The event rate for the SPRINT MIND primary outcome of incident dementia is 3.1%/yr.
- There are
  - 9250 participants in SPRINT,

- 4300 participants with eGFR < 60 ml/min/1.73m^2, and
  - 3250 participants ≥75 years old.
- Participants are recruited uniformly over 2 years.
- Minimum follow-up is 3 years, 10 months which assumes that closeout visits occur uniformly over a 4 month period.
- Two-sided tests at the 0.05 level are used.
- Annual loss to follow-up is 2 %/yr (3 %/yr for incident dementia).

Additional computational details and a justification for the assumed event rates are included in the appendix.

10.2.3 Power for the MIND primary outcome

Power for the MIND primary outcome is presented in Table 10.2 for a range of event rates with 9250 participants, 5 years and 10 months of follow-up, 2 years of recruitment, and 3 %/yr loss to follow-up. Details of the event rate estimation are given in Appendix 3.

Table 10.2. Power for the SPRINT MIND primary outcomes.

	Event Rate (%/yr)				
Hazard Ratio	3.1	3.2	3.3	3.4	3.5
0.80	96.3	96.7	97.1	97.4	97.7
0.85	79.0	80.2	81.3	82.4	83.4

10.3 Statistical Reports

10.3.1 Steering Committee Reports

Periodic reports will be generated for the Steering Committee, CCNs and Clinical Sites. These reports will include information on recruitment, loss to follow-up, adherence, baseline covariate information on the comparability of treatment groups, and adverse events. Information will be stratified by CCNs and Clinical Sites. Other reports will include information on quality control for central facilities and data entry.

10.3.2 Data and Safety Monitoring Board Reports

The role and composition of the Data and Safety Monitoring Board are described elsewhere (Chapter 13.6). Meetings of the DSMB will be held at least annually. Material for these meetings will be distributed two weeks in advance of the meetings. Up-to-date statistical analyses will be provided to the DSMB in preparation for their meetings. The analyses will include data on recruitment, outcome measures, any side-effects or safety concerns, adherence, and quality control, and will be designed in cooperation with the DSMB. Interim analyses of the intervention effectiveness will be performed at times coinciding with the meetings of the DSMB, and will be controlled to protect the overall Type I error of the trial. These results will be for the use of the DSMB and will not be revealed to the investigators. The purpose of these analyses will be for the DSMB to assess the trial progress with respect to intervention efficacy and safety, for possible recommendations regarding early termination of the trial.

We will work with the DSMB to finalize the monitoring plan. We include here a potential starting point for those discussions.

Interim analyses will be performed periodically for the DSMB. Monitored parameters will include the following:

1. SBP separation between groups
2. SBP distribution within groups
3. Primary outcome results
4. Adverse events
5. Laboratory alerts
6. Recruitment progress
7. Other event rates, and event rates by subgroups
8. Enrollment overall and by subgroups such as level of eGFR and CKD category

Sequential monitoring and early stopping. Incidence rates of outcomes will be monitored throughout the trial and used for interim analyses of efficacy and futility. Group sequential methods for event rates will be used to control the Type I error to be 0.05 across these repeated analyses. Critical values for interim testing will be defined based on an O'Brien-Fleming type bound and will use a spending function to allow flexibility in the number and timing of interim analyses. Information time will be defined based on the expected number of events under the null hypothesis. With this approach, interim tests early in the trial are conservative and the reduction in the overall power of the trial caused by interim testing is small. If needed, conditional power calculations will be used to assess the futility of continuation in the presence of a negative treatment effect.

The monitoring plan will include consideration of the hypothesis that early adverse effects may occur and then be followed by long-term beneficial effects. Because kidney function will be measured at baseline, 1, 3, and 6 months, we will be able to analyze the acute impact of our intervention on kidney function. Because of the study design, episodes of acute kidney injury (AKI) that are of more than a transient nature will be identified as changes in chronic kidney function, consistent with contemporary paradigms acknowledging the interrelationships between AKI and CKD. Episodes of AKI will be specifically sought in review of medical records in appropriate patients as adverse events. Regarding the possibility of acute cognitive decline, spontaneously reported SAEs would be the source of such information.

At each meeting, the DSMB will review data on adverse events and other safety issues to make an overall recommendation to the NIH concerning the safety of continuing SPRINT. Consistent with NIH policy, each SPRINT CCN Principal Investigator will receive a report summarizing the DSMB review of the adverse event data. Principal Investigators are responsible for providing this report to their sites and institutional IRB.

10.3.3 Website Reports

The Coordinating Center will prepare many reports and place them on the SPRINT website. These reports enable a user to click on a static link which starts a real-time report processed by SAS and returned as output in the user's web browser. These reports access live data and run within seconds. Examples of real-time reports on randomization and screening activities include: number of clinics actively recruiting, percent at target (overall, to date, and by demographic subgroups such as women and race/ethnic group). Clinical Sites will have access to live data showing exactly where their clinic stands in relation to their recruitment goals and those of the other Clinical Sites, as well as projections of activity needed to meet their goals. Committee members

will have expanded access to information across all Clinical Sites for the purpose of monitoring recruitment performance for the trial as a whole.

Chapter 11 – Data Management

11.1 Overview: Use of the World Wide Web

All Clinical Center Networks and Clinical Sites will use the World Wide Web (WWW) to enter SPRINT data collected on forms from participants seen within the Clinical Sites. Each Clinical Site will have a password protected area on the SPRINT home page through which data will be entered. Documentation of the data entry system will be maintained at the CC. In addition, training materials for measurement and data entry personnel will be available in downloadable format on the SPRINT web site. Site-specific reports relating to participant demographics, recruitment goals, etc., among other reports, will be available on the web site.

Data security in the web-based data system uses 128-bit encryption and Secure Socket Layer (SSL). Once data has been received at the CC, recovery from disasters such as natural phenomenon (water, fire, or electrical) is possible through the ability to reconstruct both the database management system and the data up to the last back-up through the use of nightly backups. This will ensure optimal recovery of data systems in the event of a disaster. Back-up tapes are kept in a locked, fire and waterproof storage cabinet away from the computer room. Additional back-up tapes will be stored at another location on the Wake Forest University Health Sciences campus. CCNs and clinical sites have local procedures for back-up and recovery of data following a disaster. As a supplement to those plans, the SPRINT CC will have all participant contact information to minimize the chance for disruption of communication with participants regarding study medications and test results.

11.2 Flow of Data from Trial Units to Databases

11.2.1 Data from Clinical Sites and Clinical Center Networks

Participant Randomization: SPRINT will use an internet-based, web browser randomization procedure. Clinical Sites access the randomization application through the study web site. Access to this application is password protected and its communications are encrypted. Once security requirements are satisfied, a series of questions identify and verify the eligibility of the participant prior to allowing randomization of the participant.

Participant Tracking: The Participant Tracking System (PTS) is a fully integrated tracking and notification system that advises clinic staff about participant follow-up windows, and projects clinic and laboratory workload for a week at a time (longer if necessary). Tracking a participant begins at screening and continues automatically throughout the project by integrating participant follow-up data with predetermined follow-up "windows". When a participant is enrolled into the study, a schedule of target dates for each of the visits is automatically generated. The report details the recommended "windows" that each visit should fall into and a case file is created for the participant.

Data Entry: The images on the data entry screens mirror the data collection forms for ease and accuracy of entry. Typically, as participant visits are completed, and hard copy forms are filled out, the clinic coordinator reviews each form for accuracy and completeness, including laboratory reports and any supporting documentation (hospital

records, etc.). Once any data problems have been resolved, data are entered by clinic staff into the computer via the web-based browser application. During data entry, a variety of programmed edit checks are performed for key variables. When the edit checks fail, data may be flagged for further review or prevented from becoming part of the study database. Also, a sample of key forms may be double-keyed for additional quality control.

11.2.2 Data from Central Laboratory and ECG Reading Center

Laboratory specimens and electrocardiographic data are sent to the Central Laboratory and ECG Reading Center from the Clinical Sites on a fixed schedule. The Central Laboratory and ECG Reading Center provide results to the CC on live internet feed. Depending on clinic needs, reports will be sent to assist in the clinical functions (e.g., providing timely feedback to the clinic on any measurement that exceeds a predefined alert level).

11.2.3 Central Database Edits

At regular intervals, data queries will be carried out on the computerized databases at the CC to perform consistency checks on key variables and forms. Although much of this will have been done at the data entry level in the clinic, this additional pass through the data serves as a quality control check.

11.3 Feedback to Clinical Sites and Clinical Center Networks

Data edit reports will be generated to help ensure that data are entered in timely and complete manner. These reports will include both the assessment for each Clinical Site of the time between data collection and entry, and the generation of reports by the CC of missing items. These reports will be provided to the Clinical Center Networks, Clinical Sites, and study committees on a regular basis so that data collection items that are troublesome can be identified and Clinical Sites not meeting study standards can be notified. CCN Coordinators will have access to all data reports for Clinical Sites within their network via the study website and will be asked to follow-up on any action that needs to be taken.

11.4 Confidentiality

The confidentiality of all participant information (including but not limited to any genetic analysis) must be protected at the Clinical Sites, the CCNs, and the CC. Paper records and computer files must be appropriately safeguarded from unauthorized access.

Paper and/or electronic records for study participants will be stored at the Clinical Sites. Copies of records identified by participant identification number pertaining to SAEs and study-defined clinical events, including necessary medical records, will be stored at the CC. These records will receive the same care as would ordinary medical records. They will be stored in locked filing cabinets and/or filing rooms within secure office space or, if uploaded through the study website, they are stored in a non-url accessible area that can be accessed only through the SPRINT website. Only study personnel who have completed SPRINT training in data handling will have access to study forms.

Similar care will be used in the handling of the computer records of study data stored at each Clinical Site. Access to the data in any local SPRINT database will be controlled by a system of user identification names and passwords. Each Clinical Site staff member must complete the SPRINT data handling training program before being given an ID and password to use the data system. The privileges allowed to each ID can be individually specified by the local CCN Coordinator. All passwords stored within the system will be encrypted using SSL encryption.

Confidentiality of information within the CC will be protected through a variety of procedures and facilities:

1. The confidential nature of the data collected, processed, and stored at the CC is explained to all new personnel.

2. All access to CC office space containing data is controlled through a single door, which is locked with a keypunch lock. This door remains locked at all times.

3. All participant data sent to the CC is encrypted as described above.

4. All participant data stored on the Wake Forest University's servers are likewise encrypted. In addition, all such databases are protected by passwords that must be supplied before the data can be accessed.

5. All study documents containing individually identifiable data are produced on printers within the CC's secure office space.

6. The CC will obtain a Certificate of Confidentiality for SPRINT, which prevents researchers from being forced to disclose identifying information by certain legal proceedings.

Chapter 12 – Quality Control

12.1 Introduction

Data integrity and quality are among the highest priorities in SPRINT. This feature is reflected in the details provided in the protocol regarding initial screening and recruitment of participants, data acquisition at baseline and follow-up visits, outcome definition and assessments, reading and/or interpretation of the results, and their analysis and publication. There are two primary purposes for quality control: to document the level of quality and to provide feedback to the clinical, reading and laboratory centers in order to maintain and improve the quality of the study data over the course of the trial. The Measurement Procedures and Quality Control Committee will establish guidelines for quality assurance and quality control, detailed in the Manual of Procedures.

Quality control monitoring in SPRINT will involve the CC, the CCN hubs, and various SPRINT committees and other groups, although the Measurement Procedures and Quality Control Subcommittee will monitor quality control and quality assurance activities for the study overall, integrating input from these other groups. For example, the Recruitment, Retention and Adherence Subcommittee will monitor progress toward achieving recruitment goals, and the SPRINT MIND subcommittee will monitor the quality of assessment with the cognitive battery. The CC will generate reports and supply them to the CCN hubs for their sites, to the Measurement Procedures and Quality Control Subcommittee for all sites and entities, and to other involved groups for the activities in their purview. The CCN hubs will be responsible for tracking the performance of sites within their Networks, and for following up with their sites on areas of concern. The Measurement Procedures and Quality Control Subcommittee will conduct monitoring for the trial overall, will raise issues on specific sites and communicate them to the CCN hub for follow-up, will monitor the central facilities (ECG reading center and central lab), and will report any areas of concern to the Steering Committee for consideration, as needed.

This chapter outlines the type of quality assurance activities that will be conducted in the SPRINT Trial. Two phrases are used. The first, quality assurance, is the collection of manuals and procedures that will be in place to assure the integrity of the data. A subset of these procedures is referred to as quality control, which describes the monitoring and analytic activities that assess performance during data collection and its processing.

12.2 Manual of Procedures

As with any multicenter study, standardization of study procedures is very important in the SPRINT Trial. The MOP includes the detailed descriptions of all trial procedures. This MOP is used for training purposes and as a reference for all study investigators and staff. The MOP is an important aspect of efforts to standardize study procedures across clinical sites in the SPRINT Trial.

Key study procedures will be standardized; these include the use of a central lab and ECG reading center, and standard forms, equipment, and procedures in the clinics for

BP measurement and other data collection procedures. Furthermore, standard event definitions and event validation procedures will be used.

12.3 Study Forms and Data Entry Procedures

Quality assurance concepts were employed during the development of forms. Forms can be printed with accompanying question-by-question instructions for easy reference. Web-based data entry screens will be developed from the forms, and enable the incorporation of range and logical checks at the time of data entry. These features will contribute to quality assurance.

12.4 Training

Training of staff and pilot testing of procedures will be crucial to standardize procedures and assure data quality. SPRINT uses two different training models: central training for study staff and the train-the-trainer approach. In the central training aspects of the SPRINT training effort, all relevant staff members from all clinical sites will be convened in a single, centrally administered face-to-face training session. This approach is cost-efficient and contributes to uniformity of the training experience and thereby to uniformity of data quality across sites. In the train-the-trainer aspect of the SPRINT training effort, CCN hub staff will provide training sessions to persons who were unable to attend the central training session and to newly hired staff as turnover occurs. In addition, the CCN hubs will organize training and refresher training sessions, as needed, including CCN remedial training in specific areas targeted by quality control monitoring for a specific site.

12.5 Data Queries

The Coordinating Center will be responsible for data editing, which will include checks for missing data, unrealistic values, and crosschecks for inconsistencies. Data will be checked on form submission and any data queries presented to the data entry staff for immediate resolution, if possible. The CC will also produce data query reports on the website that summarize the number and types of queries by clinic and network. Clinical center staff will be responsible for reviewing and resolving the data queries in a timely manner. Reports, including reports on timeliness of data entry and query resolution, will be shared with the Measurement Procedures and Quality Control Subcommittee and the corresponding CCN hub investigators and staff for quality control purposes.

12.6 Quality Control Reports

The Measurement Procedures and Quality Control Subcommittee will develop quality indicators, both to document data quality and to provide feedback to individual clinical sites, which will be tracked in routine quality control reports in the SPRINT Trial. All reports will be generated by the CC and distributed to the Subcommittee, to the corresponding CCN hub, and/or to other relevant groups (e.g., the SPRINT MIND subcommittee for those measures). Investigators and staff at the CCN hubs will be responsible for disseminating reports and feedback to the appropriate investigators and staff at the clinics in their networks. These reports will be used to inform discussions that will take place during regularly scheduled telephone contacts and site visits. Additional information about these processes is contained in the MOP.

Quality Control reports will focus on measures of process, impact, and outcomes. Examples of process measures that will be tracked for quality control purposes include:

1. Days between data collection and data entry
2. Percent of forms with late data entry
3. Number of participants with missed or late visits by contact, number of missed or late visits clinic-wide, and number of participants missing two or more consecutive visits
4. Number, name and dose of prescribed antihypertensive medications for individual participants

Examples of impact measures that will be tracked for quality control purposes include:

1. Number (and percent) of participants at goal according to the BP target assignment as assessed by in-clinic BP measurements.

Examples of outcome measures that will be tracked for quality control purposes include:

1. Submission of medical record documentation for reported study events by the clinical site (e.g., timeliness, completeness)
2. Proportion of participants with ECG submitted to central ECG Reading Center overall and by quality grade
3. Proportion of participants with urine samples submitted for albuminuria assessment
4. Proportion of participants with blood samples submitted to central lab
5. Percent agreement of individual study adjudicators with the final outcome assignments for cases adjudicated

Details of the various quality control procedures are contained in the Manual of Procedures. In general, the CC will generate reports and analyses on progress at the clinical sites on an agreed upon schedule appropriate to the study phase. Reports will most often be developed at the level of the clinical site but may also include patient-level reports by site, technician-level reports by site, and summary reports study-wide and within and across CCNs. The CC will supply these reports to the Measurement Procedures and Quality Control Subcommittee, to other relevant Subcommittees, and to the corresponding CCN hub investigators and staff.

12.6.1 Deviations from protocol

Adherence to the study protocol is crucial to collection of high quality data and to the internal validity of the trial. Thus, the Intervention Subcommittee will define important deviations from the intervention protocol for tracking purposes. A clinic-site-specific report describing important protocol deviations will be disseminated by the CC to the respective CCNs for quality control purposes. Copies of these reports and a summary report describing important protocol deviations and plan for corrective actions on a study-wide basis will be shared with the Measurement Procedures and Quality Control Subcommittee and the Steering Committee.

12.6.2 Monitoring the Clinical Centers in the Networks

Primary responsibility for clinical site monitoring in SPRINT will be assigned to the corresponding CCN hub. CCN hub investigators and staff will be responsible for

monitoring performance at each of their clinical sites. The CCN hub monitoring team will coordinate research activities of the study within their network and maintain effective communications with clinical sites, other clinical center networks, the coordinating center, project office and study central units (Central Lab, ECG Reading center, MRI Reading Center and Drug Distribution Center). One of the primary roles of CCN hubs is to monitor clinical sites in all aspects of trial operations and performance and to assist in problem solving related to all aspects of the main study and ancillary studies. Site monitoring can and will be performed using regular communications including email, conference calls, site visits and other means.

12.7 Site Initiation

Clinical site initiation to enroll and randomize participants is dependent upon completion of a series of preliminary tasks. These include completion of appropriate regulatory approvals (IRBs), and letters of agreement. Site staff training, certification, and receipt of all study supplies including medications will need to be completed as well as the development of a recruitment plan. CCNs will provide the appropriate assistance to their clinical sites toward these ends, which may include site visits to ensure that the study enrollment and randomization process follows proper study procedures.

12.8 Site Visits

12.8.1 CCNs to clinical sites

During the course of the trial, clinical center network personnel will site visit clinical sites in their network at specified intervals, and as needed. The scope of these visits is broad and can include but is not limited to regulatory requirements, study communications, site initiation, site staffing, and general site performance. A minimum standard for all site visits content and frequency is detailed in the MOP; however, areas of emphasis and/or additional monitoring may vary according to the circumstances of a specific site and site visit. Site visits may be conducted to evaluate performance deficits in one or more critical areas, such as consistent departures from the protocol or MOP. Site visits are also an opportunity for refresher training and/or training of new staff, as needed. Site visit frequency and visit procedures can be found in more detail within the appropriate section of the MOP.

Site visitors will include CCN hub and site staff and investigators as deemed appropriate. As needed, representatives from the coordinating center, project office, other CCNs, and study committees may attend these visits.

A summary of the site visit will be presented to the clinical site investigator and staff at the conclusion of the site visit. The CCN staff will prepare a written site visit report within a reasonable time-frame post visit. Copies of the site visit report will be sent to the clinical site investigator, the coordinating center, the project office, and the CCN. Additional copies of the site visit report may be requested by other SPRINT Study entities.

A sample of site visit reports may be reviewed by the Measurement Procedures and Quality Control Committee or other study committees with recommendations for follow-up actions and/or reporting changes as needed.

12.8.2 Coordinating Center to CCN hubs

The SPRINT Coordinating Center will periodically site visit each CCN hub in order to monitor and ensure high performance throughout the trial. Representatives from the NIH SPRINT project office (including NHLBI, NIA, NIDDK, and NINDS) and study leadership may also attend.

12.8.3 Project Office to Coordinating Center

Representatives from the NIH SPRINT project office and study leadership will visit the coordinating center in order to monitor and ensure high performance throughout the trial.

12.9 Laboratory and ECG Center Quality Control

The SPRINT Measurement Procedures and Quality Control Subcommittee will work with the Coordinating Center, the Central Laboratory and the ECG Reading Center to develop quality control procedures to ensure high quality data, including monitoring clinical site performance as well as performance of the Central Laboratory and ECG Reading Center. The results of quality control procedures performed at the Central Laboratory and the ECG Reading Center will be reported on a regular basis to the Measurement Procedures and Quality Control Subcommittee and by them to the Steering Committee.

<u>Core Laboratory for Blood and Urine Assays</u>
Clinical site performance in acquisition, handling, storage and shipping of specimens will be tracked by the Central Laboratory and the Measurement Procedures and Quality Control Subcommittee. The first step in quality assurance at the site level consists of the training and certification process for staff within the clinical sites. Other steps include maintaining logs of equipment checks at each clinical site according to the Manual of Operations; observation of technicians performing all steps of sample collection and processing during site visits; reviewing study forms; reviewing and tracking the condition of samples received at the Central Laboratory for problems in shipment; and periodic analysis of the study data for participant compliance with fasting, where required, and for signs of problems in drawing or processing, such as hemolysis. Reports on clinical center performance will be submitted regularly by the Central Laboratory to the CCN hubs and the SPRINT Measurement Procedures and Quality Control Subcommittee.

Performance of the Central Laboratory will be monitored regularly by the SPRINT Measurement Procedures and Quality Control Subcommittee. Quality Control procedures in the laboratory for assays include the use of the internal Laboratory Manual, training and certification of Laboratory staff, Laboratory participation in external standardization and certification quality control programs, and implementation of the SPRINT internal quality control program. Process measures, such as turn-around time for the Laboratory reporting back relevant analyte results to the clinical sites, will also be monitored. Particular attention will be paid to the feed-back of pre-specified laboratory alerts to the Clinical Sites by the Central Laboratories.

As part of the internal quality control program specified in the manual of operations, the Central Laboratory will regularly provide summaries of the internal quality control results to the Coordinating Center, including the following information for each assay: (1) monthly summary statistics (n, mean, and standard deviation) on all quality control pools, including

new pools being overlapped to replace established QC pools; (2) summaries of any unusual problems or conditions noted. The SPRINT Measurement Procedures and Quality Control Subcommittee will review these reports for evidence of trends with time in results on these pools.

ECG

Clinical site performance in acquisition and submission of ECG tracings will be tracked by the Reading Center and by the Measurement Procedures and Quality Control Subcommittee. The first step in quality assurance at the site level consists of the training and certification process. All SPRINT staff acquiring ECGs must be certified, consisting of the successful recording and transmission to EPICARE of three successive, adequate quality ECGs. The ECG Reading Center will continuously monitor ECG quality and will identify errors in acquisition. Each tracing submitted will be graded for quality and used to compile continuous quality trend analysis data for each clinical site. Quality control grade reports will be regularly submitted to the CCN hubs and to the SPRINT Measurement Procedures and Quality Control Subcommittee.

The ECG Reading Center has an internal quality control protocol that monitors performance of ECG coding and measurement. This includes regular monitoring of the repeatability and accuracy of editing ECG waveforms of the digital (electronic) ECGs, and procedures to safeguard against change in trends due to change in ECG reading software. The SPRINT Measurement Procedures and Quality Control Subcommittee will monitor performance of ECG coding and measurement within the ECG Reading Center by regularly reviewing the results of the center's quality control reports.

Chapter 13 – Study Organization

13.1 Overview

The SPRINT organizational structures and responsibilities are similar to those of other large multicenter clinical trials sponsored by government or industry. The National Heart, Lung, and Blood Institute (NHLBI) initiated this study, and the National Institute of Diabetes and Digestive and Kidney Diseases (NIDDK) is a co-sponsor of the main SPRINT trial. The National Institute of Neurological Disorders and Stroke (NINDS) and the National Institute on Aging (NIA) are jointly sponsoring the SPRINT MIND study. Five Clinical Center Networks and a Coordinating Center work together through the Steering Committee to successfully design and conduct the trial (see Figure 13.1). In addition, there is a Central Laboratory, an ECG Reading Center, an MRI Reading Center and a Drug Distribution Center. Scientific leadership is provided by the Steering Committee. External oversight is provided by Institutional Review Boards and a Data and Safety Monitoring Board.

13.2 Clinical Center Networks and Clinical Sites

SPRINT participants will be recruited, randomized, treated, and followed through a system of five CCNs. Each CCN consists of collaborating clinical sites, which are medical facilities and/or individual practices involved in the initial evaluation, enrollment, treatment and follow-up of participants in the trial. Each CCN and clinical site will be responsible for timely recruitment and protocol adherence in accordance with the SPRINT protocol and MOP. In addition, the CCNs will contribute to the study's scientific leadership and operational management, and each CCN Principal Investigator (PI) will participate in Steering Committee and other investigator meetings. The clinical sites will collect data at the local level in accordance with the study protocol and the manual of operations, and will manage each participant's hypertension treatment. For all participants recruited, the CCNs and clinical sites will be responsible for achieving the goals specified in the protocol for adherence to study treatment and retention of study participants. The CCN will have the primary responsibility for overseeing their clinical sites and timely evaluation and correction of recruitment, adherence, and retention problems, including development and implementation of alternative strategies to achieve the stipulated goals, and funding the related activities. It is anticipated that each CCN will conduct periodic site visits within its network of clinical sites to supervise recruitment, adherence, and retention activities and to ensure high quality performance. The CCN activities will be coordinated with the CC, and may include site visits conducted by the CC, along with other organizational components of the study. The CCNs will collaborate closely with and assist the CC in implementation and standardization of the protocol within its network.

13.3 The Coordinating Center

The CC, with input from the SPRINT Steering Committee, will be responsible for coordinating protocol writing activities, including protocol drafting and finalization; developing and distributing forms and the MOP; training trial personnel in standardized protocol implementation and data collection; generating and distributing numerous reports (including specific recruitment goals and projections); providing rapid feedback to the CCN and Central Units on the quality of data submitted and proposed corrections;

developing and maintaining trial databases and related internal and public websites; collecting, managing, and analyzing all trial data; developing and overseeing the web-based adjudication of clinical events and endpoints; preparing reports for the DSMB; ensuring that the provisions of the manual of operations are carried out by all investigating groups; and providing timely and high quality statistical analysis expertise as required to prepare presentations and manuscripts. The CC will conduct periodic visits to each CCN in order to monitor and ensure high performance throughout the trial.

The CC will oversee 4 Central Units: the Drug Distribution Center, the Central Laboratory, the ECG Reading Center, and the MRI Reading Center.

The Central Laboratory will serve as a repository for immediate and future analyses of urine and blood specimens. The Central Laboratory will be responsible for the development and distribution of specific measurement procedures, and laboratory analyses, and for participating in quality assurance activities related to laboratory measures. Periodic reports will be generated to address sample acquisition quality for each clinical site and assay performance, and these will be provided to the CCNs and the Measurements, Procedures and Quality Control (MPQC) Subcommittee for review.

The ECG Reading Center will provide central interpretation of ECGs. The ECG Reading Center will develop procedures for obtaining and transmitting ECG data from the clinical sites to ensure the highest quality data collection. Periodic reports will be generated to address ECG quality for each clinical site, and these will be provided to the CCNs and the MPQC for review.

In collaboration with each CCN participating in the MRI study, the MRI Reading Center will identify an MRI site which is located in geographic proximity to the CCN's clinical sites. The MRI Reading Center will develop a detailed protocol and manual of procedures to ensure that the MRIs taken over time are of the highest quality with the smallest variation due to changes in technique and to allow the most precise estimate of change over time. The MRI Reading Center will provide training and certification for MRI site staff in order to ensure uniformity of methods, and will monitor carefully the quality of their work. Working with the CC, the MRI Reading Center will develop an analytical plan to estimate as precisely as possible the change in brain MRI over time for each SPRINT-MIND-MRI participant. Periodic reports will be generated to address MRI quality for each scanning site, and these will be provided to the CCNs and the MPQC for review.

The Drug Distribution Center will be responsible for developing and implementing plans for cost-effective drug acquisition; packaging, labeling, and dispensing drugs according to the study protocol; and providing data to the CC for further analyses. The DDC will design the technical aspects of drug packaging and labeling to facilitate participants' ability to understand and adhere to the drug regimen. The DDC will work with the clinical sites and CCNs to develop cost-effective inventory management procedures.

13.4 NHLBI Project Office and Other Government Representatives

The NHLBI Project Office will be responsible for the scientific conduct and administration of SPRINT. Representatives from the Project Office participate in the scientific, general organizational and fiscal management of the trial. NHLBI staff includes scientific representation from the Project Office team and members of the Office of Acquisitions

and the Office of Biostatistics Research. In addition, the NIH SPRINT team includes scientific staff from the NIDDK, the NINDS and the NIA.

13.5 The SPRINT Steering Committee, Executive Committee, Conflict of Interest Committee and the Subcommittees of the Steering Committee

The SPRINT Steering Committee provides the overall leadership for the study and establishes scientific and administrative policy. It is composed of the Principal Investigators from the five Clinical Center Networks, the Principal Investigator from the Coordinating Center, the NHLBI Project Officer, representatives from NIDDK, NINDS, NIA, the Steering Committee Chair, and the Steering Committee Vice-Chair. This committee oversees the overall conduct of the trial throughout all phases, develops the trial design, prepares the final protocol, and approves the study forms and manual of operations. During the data collection phases of the trial, this committee oversees data collection practices and procedures to identify and correct deficiencies. The Steering Committee also will consider and adopt changes in the study protocol or procedures as necessary during the course of the trial.

The SPRINT Steering Committee is chaired by the Steering Committee Chair, who serves as the senior executive officer of the investigative group. A Vice-Chair assists the Chair with Steering Committee responsibilities. Voting Steering Committee members are the Principal Investigators from the five CCNs, the Principal Investigator from the Coordinating Center, and the NHLBI Project Officer. If a CCN PI or the CC PI cannot make a meeting at which a vote is taken, then the Co-Principal Investigator may vote (with the understanding that the Co-PI is fully informed about the issue). The Steering Committee Chair, or Vice-Chair in his/her absence, votes only to break a tie. CCN and Site Co-investigators and Coordinators, CC staff, NIH staff, consultants, and opinion leaders may also be invited to attend meetings.

The SPRINT Executive Committee will oversee the day-to-day operations of the trial as an extension of the Steering Committee to ensure efficient and quality performance. The members include the Steering Committee Chair, Steering Committee Vice-Chair, Coordinating Center personnel, Project Office personnel, and one CCN PI (rotated annually so that each PI has the opportunity to serve). Other key study personnel (e.g., Chair of the Operations/Project Coordinators Subcommittee, Director of the DDC) may be asked to participate as either ad hoc or regular members.

The SPRINT Conflict of Interest Committee reviews potential conflict of interest issues. The NIH Project Office, Steering Committee Chair, and CC PI comprise this committee, which has the overall responsibility for the trial's ethical oversight policy and procedures.

There are a number of standing subcommittees and working groups which report to the Steering Committee. These subcommittees and groups and their charges are detailed in Appendix 5.

13.6 The Data and Safety Monitoring Board

An independent Data and Safety Monitoring Board will be established to monitor data and oversee participant safety. Members will be appointed by the NHLBI to provide oversight of the trial and its ancillary studies. The SPRINT DSMB may include experts in cardiovascular medicine (particularly hypertension), kidney disease, clinical trials,

geriatrics, biostatistics, bioethics, quality of life, cost effectiveness, cognitive function and other areas as needed. DSMB participants include the Steering Committee Chair (who is unblinded) and Vice-Chair (who is blinded), CC PI and senior staff, and representatives from the NHLBI and other NIH sponsors. The DSMB normally meets twice a year to monitor safety, to advise the NHLBI about study progress, including contractor performance, and to make recommendations to the NHLBI regarding study continuation and protocol changes. In addition, the CC may provide data to the DSMB Chair to ensure early identification of any major adverse outcomes of therapy. The DSMB has the responsibility to recommend to the NHLBI whether the trial should continue, whether the protocol should be modified, or whether there should be early termination. The DSMB will provide reports to the NHLBI through the Executive Secretary, who will be appointed by the NHLBI. Recommendations by the DSMB must be approved by the NHLBI prior to implementation.

13.7 Role of Industry

Industry may contribute resources to the study and will be acknowledged appropriately. However, the scientific decisions and governance of the trial will be determined by the Steering Committee, as per NHLBI Policy.

13.8 Conflict of Interest Policy

The SPRINT investigators have established a policy regarding Conflict of Interest, which is presented in the MOP. This policy was developed to meet two goals. First, the investigators wished to maintain the confidence that advice was being given, and decisions made, in as unbiased and fully informed manner as possible. Second, the investigators wished that the processes and results of the trial would meet public standards of conduct.

13.9 Timeline

SPRINT will begin recruiting and randomizing during the fall of 2010. Recruitment will continue for approximately two years. The minimum length of participant planned follow-up will be four years, and maximum length of follow-up will be approximately six years, so the final study visits will occur in late 2016 or early 2017. If the event rate in the standard therapy arm is substantially less than 2.2%, we may ask that the DSMB consider recommending a two year extension of the trial.

13.10 Ancillary Studies

13.10.1 Introduction

In addition to the main SPRINT protocol, investigators may wish to perform Ancillary Studies using the SPRINT population, blood or urine samples, or other collected data. An ancillary study is an investigation not initiated by the SPRINT Steering Committee, with objectives that are not within the main SPRINT specific objectives and not part of the SPRINT protocol but uses SPRINT participants, samples, and/or data collected by SPRINT. In most cases, an ancillary study will involve acquisition of additional data that are not compiled as part of the SPRINT data set. An ancillary study may or may not use all randomized participants. Investigators are encouraged to propose and conduct ancillary studies. Such studies enhance the value and productivity of SPRINT and help

ensure the continued interest of the diverse group of investigators who are critical to the success of the trial as a whole. These studies provide an exceptional opportunity for investigators, either within or outside of SPRINT, to conduct additional projects at relatively low cost. In general, ancillary studies will require additional funding from the NIH or other sources.

13.10.2 Application Review Process

To protect the integrity of SPRINT, all ancillary studies must be reviewed and approved by the SPRINT Steering Committee before access to SPRINT data, samples, or participants is permitted. Investigators will not be allowed access to the SPRINT participants, samples, or database without approval. New ancillary study proposals will be submitted to the SPRINT Ancillary Science (AS) Subcommittee, which will review all ancillary study proposals and make a recommendation to the Steering Committee. In the event that investigators wish to modify an ancillary science protocol that has already been approved by the SPRINT SC, they will need to first obtain AS Subcommittee and SC approval. Ancillary study forms can be obtained by contacting the Coordinating Center or accessing the SPRINT website.

Studies submitted for approval less than four months prior to a funding application deadline may not receive timely approval. When the application is complete, the study proposal will be sent to the AS Subcommittee for review. The AS Subcommittee will have monthly calls to discuss proposals, which will be circulated at least one week prior to the calls. After review and approval by the AS Subcommittee, approval/disapproval will be made by the Steering Committee. Ancillary Science investigators must include one or more SPRINT investigators in their ancillary study proposals.

The Coordinating Center will usually be responsible for all data management and analysis for all ancillary studies. Specialized expertise external to the coordinating center (e.g., processing of images) may be needed at the coordinating center's discretion. Costs associated with ancillary study data management and analysis must be budgeted into each ancillary study, even if the applicants have the necessary expertise in data management and analysis.

Prior to grant submission (or study initiation if no external funding is required), the CCN PI must approve participation of sites in her/his network. This is required as the CCN PI is responsible for the conduct of all aspects of SPRINT within her/his network. Part of this is management and oversight of clinic and participant burden. As needed, the CCN will include funding for oversight (e.g., investigator, coordinator, and fiscal personnel time, travel). The SPRINT Steering Committee also reserves the right to review the burden of ancillary studies on an on-going basis and take appropriate actions as necessary. Investigators with approved ancillary studies will report the status of the studies annually to the Chair of the AS Subcommittee.

Additional detail on the review process and criteria for judging proposals can be found in the MOP.

13.10.3 Additional Requirements of Ancillary Science Investigators

All ancillary study investigators will be required to budget adequately for all necessary resources for their studies. This includes, but may not be limited to, costs for data

collection, sample collection, sample shipping, sample extraction, sample analysis, data entry, website development, data analysis, dataset preparation, data storage and publication of results. The final budget may be determined after AS and SC approval.

Each ancillary study will cause an increase in utilization of main SPRINT study resources, particularly by the SPRINT Presentations and Publications (P&P) Subcommittee. To help with study operations, each ancillary science proposal team should budget for and may be asked to contribute efforts to the main SPRINT study by, for example, assigning a person to serve as a reviewer for the P&P Subcommittee.

Investigators proposing the use of laboratory measurements are encouraged to use the SPRINT Central Laboratory if at all possible. This will facilitate sample processing and shipping and may reduce the amount of sample required.

All images (e.g., MRI) or tracings (e.g., ECG) must be available for other investigators to use in the spirit of the NIH policy available at http://grants.nih.gov/grants/sharing.htm. To achieve this goal, ancillary studies must budget for the costs associated with archiving these images and making them available to others. If there are legitimate reasons why this cannot be accomplished, this can be discussed on a case-by-case basis by the investigators, the funding agency, and the SPRINT SC.

13.11 Publication Policy

The purpose of the policy is to encourage and facilitate the presentation and publication of SPRINT Study background, rationale, design, and analyses; ensure appropriate use of the SPRINT data, timely completion of manuscripts and presentations, equitable access to authorship, and adherence to established principles of authorship; and coordinate the reporting of trial results. The policy applies to all investigators analyzing, presenting, and publishing data from main SPRINT, SPRINT-MIND, SPRINT-Senior (hereafter collectively called "SPRINT") and ancillary studies, except for those using the NHLBI Data Repository data (see *https://biolincc.nhlbi.nih.gov/home/*).

There are several principles underlying this policy:

1. Research questions and hypotheses to be addressed using SPRINT Study data should be formulated *a priori* and clearly stated in a manuscript proposal to reduce the likelihood that study results are attributable to type I error.

2. Publication of scientific findings from the SPRINT Study should proceed in a timely fashion once relevant analyses are complete.

3. The publications arising from the SPRINT Study should avoid overlap and conflicting representation of SPRINT Study findings. Overlaps are, however, acceptable for review articles.

4. Recognition through authorship will be distributed among the SPRINT investigators so that:
    i) all SPRINT investigators and team members have equitable opportunity to lead and co-author SPRINT publications and, if appropriate, publications from ancillary studies;

ii) all Ancillary Study investigators have the opportunity to lead and be co-authors on publications resulting from their ancillary studies.

5. The SPRINT Study should promote the career development of trainees and junior faculty by providing them the opportunity to lead and be recognized as co-authors of SPRINT publications, as appropriate.

6. Standards for authorship on SPRINT publications will adhere to the Uniform Requirements for Manuscripts Submitted to Biomedical Journals of the International Committee of Medical Journal Editors (NEJM 1997;336:309-315) and those established by the destination journals.

7. The concept, in the form of a proposal, for all manuscripts must be approved by the P&P Subcommittee prior to preparation.

There are three categories of manuscripts and anticipated authorship:

i) Main results developed based on core SPRINT data and study aims/hypotheses (which will bear the corporate authorship, "The SPRINT Research Group"). The design and main baseline papers will also be corporate authored.

ii) Manuscripts developed and authored by investigators using data that are not considered to be main SPRINT results.

iii) Ancillary study results led by investigators bringing external funding or resources into SPRINT for a specific project.

    (1) Unless specific justifications and alternative arrangements are made, all SPRINT analyses will be performed by the Coordinating Center (CC), with specialized expertise external to the Coordinating Center as needed at the Coordinating Center's discretion. Ancillary study budgets should include funds allocated to the CC for that purpose.

    (2) Ancillary study manuscripts are subject to similar review and tracking procedures as other SPRINT manuscripts.

During the operational phase of the trial, manuscripts proposing to use data other than baseline data will be reviewed closely to ensure that the SPRINT study objectives are not compromised. In general, the following will not be allowed:

(1) Publication of follow-up data according to randomized group

(2) Longitudinal analyses of outcomes pre-specified in the main protocol

All such proposals will be considered on a case-by-case basis.

The final responsibility for review and approval of manuscript proposals, including composition of writing committees, readiness for submission, and abstracts and material

for presentations at meetings and conferences, rests with the Steering Committee. The P&P Subcommittee will oversee and facilitate these processes, assisted by a Publications Coordinator based at the Coordinating Center.

Figure 13.1: SPRINT Organizational Chart

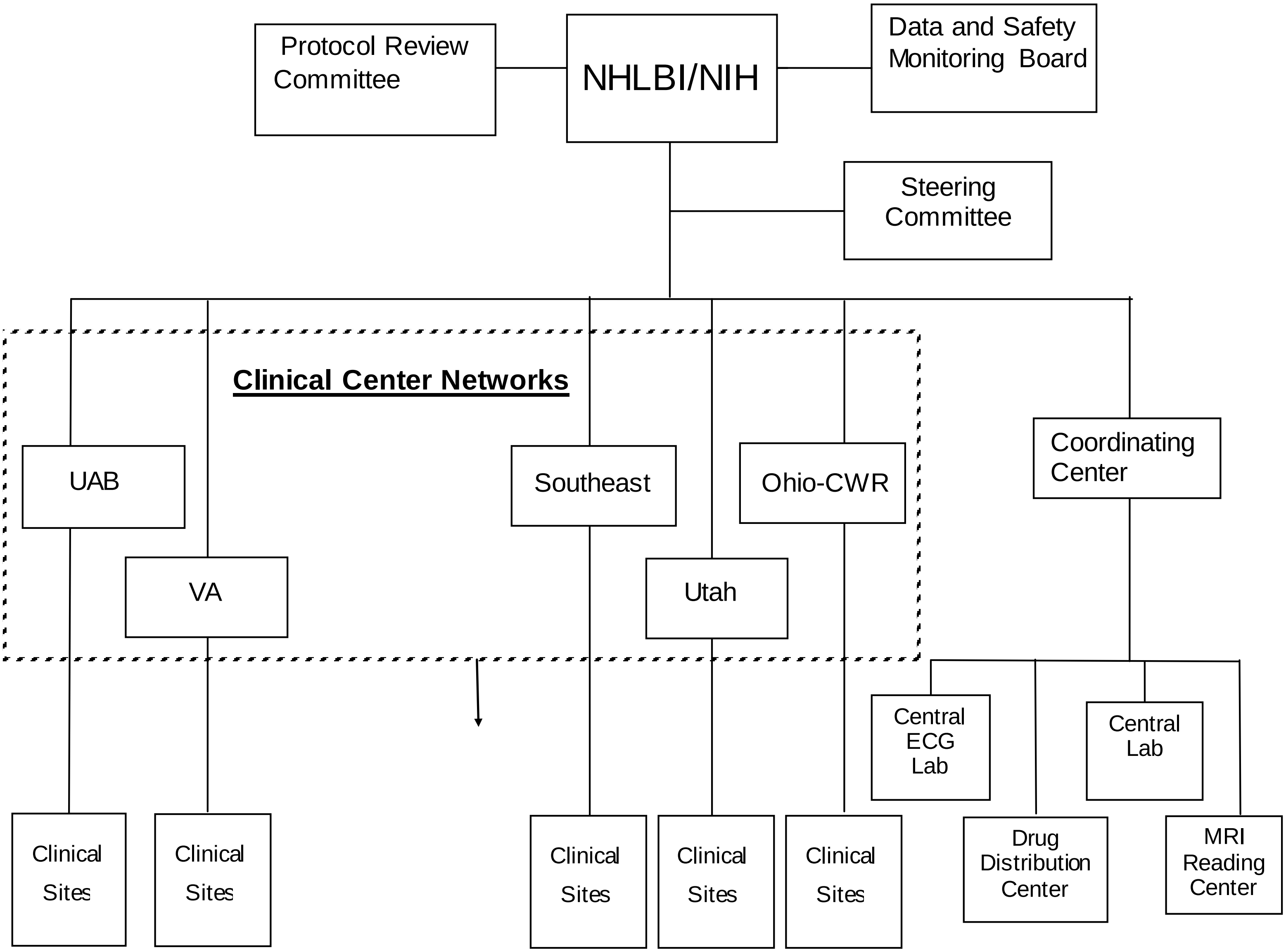

Reference List

1. Working Group Report: Expert Panel on a Hypertension Treatment Trial Initiative Meeting Summary. 2007.
   Ref Type: Internet Communication

2. Abernethy, J., Borhani, N. O., Hawkins, C. M., Crow, R., Entwisle, G., Jones, J. W., Maxwell, M. H., Langford, H., and Pressel, S., 1986, Systolic blood pressure as an independent predictor of mortality in the Hypertension Detection and Follow-up Program: Am.J Prev.Med, v. 2, p. 123-132.

3. ALLHAT, 2002, Major outcomes in high-risk hypertensive patients randomized to angiotensin-converting enzyme inhibitor or calcium channel blocker vs diuretic: The Antihypertensive and Lipid-Lowering Treatment to Prevent Heart Attack Trial (ALLHAT): JAMA, v. 288, p. 2981-2997.

4. ALLHAT, 2003, Diuretic versus alpha-blocker as first-step antihypertensive therapy: final results from the Antihypertensive and Lipid-Lowering Treatment to Prevent Heart Attack Trial (ALLHAT): Hypertension, v. 42, p. 239-246.

5. Appel, L. J., Wright, J. T., Jr., Greene, T., Kusek, J. W., Lewis, J. B., Wang, X., Lipkowitz, M. S., Norris, K. C., Bakris, G. L., Rahman, M., Contreras, G., Rostand, S. G., Kopple, J. D., Gabbai, F. B., Schulman, G. I., Gassman, J. J., Charleston, J., and Agodoa, L. Y., 2008, Long-term effects of renin-angiotensin system-blocking therapy and a low blood pressure goal on progression of hypertensive chronic kidney disease in African Americans: Archives of Internal Medicine, v. 168, p. 832-839.

6. Ay, H., Benner, T., Arsava, E. M., Furie, K. L., Singhal, A. B., Jensen, M. B., Ayata, C., Towfighi, A., Smith, E. E., Chong, J. Y., Koroshetz, W. J., and Sorensen, A. G., 2007, A computerized algorithm for etiologic classification of ischemic stroke - The causative classification of stroke system: Stroke, v. 38, p. 2979-2984.

7. Bakris, G. L., Williams, M., Dworkin, L., Elliott, W. J., Epstein, M., Toto, R., Tuttle, K., Douglas, J., Hsueh, W., and Sowers, J., 2000, Preserving renal function in adults with hypertension and diabetes: a consensus approach. National Kidney Foundation Hypertension and Diabetes Executive Committees Working Group: Am.J Kidney Dis., v. 36, p. 646-661.

8. Basile, A. M., Pantoni, L., Pracucci, G., Asplund, K., Chabriat, H., Erkinjuntti, T., Fazekas, F., Ferro, J. M., Hennerici, M., O'brien, J., Scheltens, P., Visser, M. C., Wahlund, L. O., Waldemar, G., Wallin, A., and Inzitari, D., 2006, Age, hypertension, and lacunar stroke are the major determinants of the severity of age-related white matter changes. The LADIS (Leukoaraiosis and Disability in the Elderly) Study: Cerebrovasc.Dis., v. 21, p. 315-322.

9. Beckett, N. S., Peters, R., Fletcher, A. E., Staessen, J. A., Liu, L., Dumitrascu, D., Stoyanovsky, V., Antikainen, R. L., Nikitin, Y., Anderson, C., Belhani, A., Forette, F., Rajkumar, C., Thijs, L., Banya, W., and Bulpitt, C. J., 2008, Treatment of hypertension in patients 80 years of age or older: N Engl J Med, v. 358, p. 1887-1898.

10. Berl, T., Hunsicker, L. G., Lewis, J. B., Pfeffer, M. A., Porush, J. G., Rouleau, J. L., Drury, P. L., Esmatjes, E., Hricik, D., Pohl, M., Raz, I., Vanhille, P., Wiegmann, T. B., Wolfe, B. M., Locatelli, F., Goldhaber, S. Z., Lewis, E. J., and for the Collaborative Study Group, 2005, Impact of Achieved Blood Pressure on Cardiovascular Outcomes in the Irbesartan Diabetic Nephropathy Trial: Journal of the American Society of Nephrology, v. 16, p. 2170-2179.

11. Berlowitz, D. R., Ash, A. S., Hickey, E. C., Friedman, R. H., Glickman, M., Kader, B., and Moskowitz, M. A., 1998, Inadequate management of blood pressure in a hypertensive population: New England Journal of Medicine, v. 339, p. 1957-1963.

12. Bharmal, M., Payne, K., Atkinson, M. J., Desrosiers, M. P., Morisky, D. E., and Gemmen, E., 2009, Validation of an abbreviated Treatment Satisfaction Questionnaire for Medication (TSQM-9) among patients on antihypertensive medications: Health Qual.Life Outcomes., v. 7, p. 36.

13. Birns, J., Morris, R., Donaldson, N., and Kalra, L., 2006, The effects of blood pressure reduction on cognitive function: a review of effects based on pooled data from clinical trials
BIRNS2006: J.Hypertens., v. 24, p. 1907-1914.

14. Brandt, J. and Benedict, R. H. B., 2001, Hopkins Verbal Learning Test-Revised. Professional manual: Lutz, FL, Psychological Assessment Resources, Inc.

15. Braunwald, E., Domanski, M. J., Fowler, S. E., Geller, N. L., Gersh, B. J., Hsia, J., Pfeffer, M. A., Rice, M. M., Rosenberg, Y. D., and Rouleau, J. L., 2004, Angiotensin-converting-enzyme inhibition in stable coronary artery disease: N Engl J Med., v. 351, p. 2058-2068.

16. Brookmeyer, R., Corrada, M. M., Curriero, F. C., and Kawas, C., 2002, Survival following a diagnosis of Alzheimer disease: Arch.Neurol., v. 59, p. 1764-1767.

17. Calhoun, D. A., Jones, D., Textor, S., Goff, D. C., Murphy, T. P., Toto, R. D., White, A., Cushman, W. C., White, W., Sica, D., Ferdinand, K., Giles, T. D., Falkner, B., and Carey, R. M., 2008, Resistant hypertension: Diagnosis, evaluation, and treatment - A Scientific Statement from the American Heart Association Professional Education Committee of the Council for High Blood Pressure Research: Hypertension, v. 51, p. 1403-1419.

18. Calhoun, D. A., Lacourciere, Y., Chiang, Y. T., and Glazer, R. D., 2009, Triple Antihypertensive Therapy With Amlodipine, Valsartan, and

Hydrochlorothiazide A Randomized Clinical Trial: Hypertension, v. 54, p. 32-39.

19. **Campbell, N. R., Khan, N. A., Hill, M. D., Tremblay, G., Lebel, M., Kaczorowski, J., McAlister, F. A., Lewanczuk, R. Z., and Tobe, S., 2009, 2009 Canadian Hypertension Education Program recommendations: the scientific summary--an annual update: Can.J Cardiol., v. 25, p. 271-277.**

20. **Chobanian, A. V., Bakris, G. L., Black, H. R., Cushman, W. C., Green, L. A., Izzo, J. L., Jr., Jones, D. W., Materson, B. J., Oparil, S., Wright, J. T., Jr., and Roccella, E. J., 2003, The Seventh Report of the Joint National Committee on Prevention, Detection, Evaluation, and Treatment of High Blood Pressure: the JNC 7 report: JAMA, v. 289, p. 2560-2572.**

21. **Coca, S. G., Krumholz, H. M., Garg, A. X., and Parikh, C. R., 2006, Underrepresentation of renal disease in randomized controlled trials of cardiovascular disease: JAMA, v. 296, p. 1377-1384.**

22. **Coker L.H., Hogan P.E., Bryan N.R., and et al. Effects of postmenopausal hormone therapy on volumetric sub-clinical cerebrovascular disease: The Women's Health Initiative Memory Study - Magnetic Resonance Imaging Study (WHIMS-MRI). 2008.**
    Ref Type: Unpublished Work

23. **Collins, R., Peto, R., Macmahon, S., Hebert, P., Fiebach, N. H., Eberlein, K. A., Godwin, J., Qizilbash, N., Taylor, J. O., and Hennekens, C. H., 1990, Blood pressure, stroke, and coronary heart disease. Part 2, Short-term reductions in blood pressure: overview of randomised drug trials in their epidemiological context: Lancet, v. 335, p. 827-838.**

24. **Coresh, J., Selvin, E., Stevens, L. A., Manzi, J., Kusek, J. W., Eggers, P., Van Lente, F., and Levey, A. S., 2007, Prevalence of chronic kidney disease in the United States: JAMA, v. 298, p. 2038-2047.**

25. **Cox, D. R., 1972, Regression models and life-tables: Journal of the Royal Statistical Society, Series B: Methodological, v. 34, p. 187-220.**

26. **Cruickshank, J. M., 2000, Antihypertensive treatment and the J-curve: Cardiovasc.Drugs Ther., v. 14, p. 373-379.**

27. **Cruickshank, J. M., Pennert, K., Sorman, A. E., Thorp, J. M., Zacharias, F. M., and Zacharias, F. J., 1987, Low mortality from all causes, including myocardial infarction, in well-controlled hypertensives treated with a beta-blocker plus other antihypertensives: J Hypertens., v. 5, p. 489-498.**

28. **Cushman, W. C., Ford, C. E., Cutler, J. A., Margolis, K. L., Davis, B. R., Grimm, R. H., Black, H. R., Hamilton, B. P., Holland, J., Nwachuku, C., Papademetriou, V., Probstfield, J., Wright, J. T., Jr., Alderman, M. H., Weiss, R. J., Piller, L., Bettencourt, J., and Walsh, S. M., 2002, Success and predictors of blood pressure control in diverse North American settings:**

the antihypertensive and lipid-lowering treatment to prevent heart attack trial (ALLHAT): J.Clin.Hypertens.(Greenwich.), v. 4, p. 393-404.

29. Cushman, W. C., Ford, C. E., Einhorn, P. T., Wright, J. T., Preston, R. A., Davis, B. R., Basile, J. N., Whelton, P. K., Weiss, R. J., Bastien, A., Courtney, D. L., Hamilton, B. P., Kirchner, K., Louis, G. T., Retta, T. M., and Vidt, D. G., 2008, Blood Pressure Control by Drug Group in the Antihypertensive and Lipid-Lowering Treatment to Prevent Heart Attack Trial (ALLHAT): Journal of Clinical Hypertension, v. 10, p. 751-760.

30. Cushman, W. C., Grimm, R. H., Cutler, J. A., Evans, G. W., Capes, S., Corson, M. A., Sadler, L. S., Alderman, M. H., Peterson, K., Bertoni, A., and Basile, J. N., 2007, Rationale and design for the blood pressure intervention of the Action to Control Cardiovascular Risk in Diabetes (ACCORD) trial: American Journal of Cardiology, v. 99, p. 44I-55I.

31. Cutler, J. A., MacMahon, S. W., and Furberg, C. D., 1989, Controlled clinical trials of drug treatment for hypertension. A review: Hypertension, v. 13, p. I36-I44.

32. Cutler, J. A., Sorlie, P. D., Wolz, M., Thom, T., Fields, L. E., and Roccella, E. J., 2008, Trends in Hypertension Prevalence, Awareness, Treatment, and Control Rates in United States Adults Between 1988-1994 and 1999-2004: Hypertension, v. 52, p. 818-827.

33. Debette, S., Beiser, A., DeCarli, C., Au, R., Himali, J. J., Kelly-Hayes, M., Romero, J. R., Kase, C. S., Wolf, P. A., and Seshadri, S., 2010, Association of MRI markers of vascular brain injury with incident stroke, mild cognitive impairment, dementia, and mortality: the Framingham Offspring Study: Stroke, v. 41, p. 600-606.

34. DeKosky, S. T., Williamson, J. D., Fitzpatrick, A. L., Kronmal, R. A., Ives, D. G., Saxton, J. A., Lopez, O. L., Burke, G., Carlson, M. C., Fried, L. P., Kuller, L. H., Robbins, J. A., Tracy, R. P., Woolard, N. F., Dunn, L., Snitz, B. E., Nahin, R. L., Furberg, C. D., and for the Ginkgo Evaluation of Memory (GEM) Study Investigators, 2008, Ginkgo biloba for Prevention of Dementia: A Randomized Controlled Trial: JAMA: The Journal of the American Medical Association, v. 300, p. 2253-2262.

35. Di Angelantonio, E., Danesh, J., Eiriksdottir, G., and Gudnason, V., 2007, Renal function and risk of coronary heart disease in general populations: new prospective study and systematic review: PLoS.Med, v. 4, p. e270.

36. Di Bari, M., Pahor, M., Franse, L. V., Shorr, R. I., Ferrucci, L., Wan, J. Y., Somes, G. W., and Applegate, W. B., 2001, Dementia and disability outcomes in large hypertension trials: lessons learned from the Systolic Hypertension in the Elderly Program (SHEP) Trial: Am.J.Epidemiol., v. 153, p. 72-78.

37. Dufouil, C., Chalmers, J., Coskun, O., Besancon, V., Bousser, M. G., Guillon, P., Macmahon, S., Mazoyer, B., Neal, B., Woodward, M., Tzourio-

Mazoyer, N., and Tzourio, C., 2005, Effects of blood pressure lowering on cerebral white matter hyperintensities in patients with stroke: the PROGRESS (Perindopril Protection Against Recurrent Stroke Study) Magnetic Resonance Imaging Substudy: Circulation, v. 112, p. 1644-1650.

38. Dufouil, C., Godin, O., Chalmers, J., Coskun, O., Macmahon, S., Tzourio-Mazoyer, N., Bousser, M. G., Anderson, C., Mazoyer, B., and Tzourio, C., 2009, Severe cerebral white matter hyperintensities predict severe cognitive decline in patients with cerebrovascular disease history: Stroke, v. 40, p. 2219-2221.

39. Elias, M. F., Wolf, P. A., D'Agostino, R. B., Cobb, J., and White, L. R., 1993, Untreated blood pressure level is inversely related to cognitive functioning: the Framingham Study: Am.J.Epidemiol., v. 138, p. 353-364.

40. Ellis, R. J., Jan, K., Kawas, C., Koller, W. C., Lyons, K. E., Jeste, D. V., Hansen, L. A., and Thal, L. J., 1998, Diagnostic validity of the dementia questionnaire for Alzheimer disease: Arch.Neurol., v. 55, p. 360-365.

41. Ernst, M. E., Carter, B. L., Goerdt, C. J., Steffensmeier, J. J. G., Phillips, B. B., Zimmerman, M. B., and Bergus, G. R., 2006, Comparative antihypertensive effects of hydrochlorothiazide and chlorthalidone on ambulatory and office blood pressure: Hypertension, v. 47, p. 352-358.

42. Ferri, C. P., Prince, M., Brayne, C., Brodaty, H., Fratiglioni, L., Ganguli, M., Hall, K., Hasegawa, K., Hendrie, H., Huang, Y., Jorm, A., Mathers, C., Menezes, P. R., Rimmer, E., and Scazufca, M., 2005, Global prevalence of dementia: a Delphi consensus study: Lancet, v. 366, p. 2112-2117.

43. Forette, F., Seux, M. L., Staessen, J. A., Thijs, L., Birkenhager, W. H., Babarskiene, M. R., Babeanu, S., Bossini, A., Gil-Extremera, B., Girerd, X., Laks, T., Lilov, E., Moisseyev, V., Tuomilehto, J., Vanhanen, H., Webster, J., Yodfat, Y., and Fagard, R., 1998, Prevention of dementia in randomised double-blind placebo-controlled Systolic Hypertension in Europe (Syst-Eur) trial: Lancet, v. 352, p. 1347-1351.

44. Foster, M. C., Hwang, S. J., Larson, M. G., Parikh, N. I., Meigs, J. B., Vasan, R. S., Wang, T. J., Levy, D., and Fox, C. S., 2007, Cross-classification of microalbuminuria and reduced glomerular filtration rate: associations between cardiovascular disease risk factors and clinical outcomes: Archives of Internal Medicine, v. 167, p. 1386-1392.

45. Fox, K. M., 2003, Efficacy of perindopril in reduction of cardiovascular events among patients with stable coronary artery disease: randomised, double-blind, placebo-controlled, multicentre trial (the EUROPA study): Lancet., v. 362, p. 782-788.

46. Franklin, S. S., 1999, Cardiovascular risks related to increased diastolic, systolic and pulse pressure. An epidemiologist's point of view: Pathol.Biol.(Paris), v. 47, p. 594-603.

47. Franklin, S. S., Jacobs, M. J., Wong, N. D., L'Italien, G. J., and Lapuerta, P., 2001, Predominance of isolated systolic hypertension among middle-aged and elderly US hypertensives: analysis based on National Health and Nutrition Examination Survey (NHANES) III: Hypertension, v. 37, p. 869-874.

48. Freitag, M. H., Peila, R., Masaki, K., Petrovitch, H., Ross, G. W., White, L. R., and Launer, L. J., 2006, Midlife pulse pressure and incidence of dementia: the Honolulu-Asia Aging Study: Stroke, v. 37, p. 33-37.

49. Fried, L. F., Katz, R., Cushman, M., Sarnak, M., Shlipak, M. G., Kuller, L., and Newman, A. B., 2009, Change in cardiovascular risk factors with progression of kidney disease: Am.J Nephrol., v. 29, p. 334-341.

50. Gillum, R. F., 1991, Cardiovascular disease in the United States: an epidemiologic overview: Cardiovasc.Clin., v. 21, p. 3-16.

51. Go, A. S., Chertow, G. M., Fan, D., McCulloch, C. E., and Hsu, C. Y., 2004, Chronic kidney disease and the risks of death, cardiovascular events, and hospitalization: N.Engl.J.Med., v. 351, p. 1296-1305.

52. Greenlund, K. J., Croft, J. B., and Mensah, G. A., 2004, Prevalence of heart disease and stroke risk factors in persons with prehypertension in the United States, 1999-2000: Archives of Internal Medicine, v. 164, p. 2113-2118.

53. guero-Torres, H., von, S. E., Viitanen, M., Winblad, B., and Fratiglioni, L., 2001, Institutionalization in the elderly: the role of chronic diseases and dementia. Cross-sectional and longitudinal data from a population-based study: J.Clin.Epidemiol., v. 54, p. 795-801.

54. Guralnik, J. M., Leveille, S. G., Hirsch, R., Ferrucci, L., and Fried, L. P., 1997, The impact of disability in older women: J.Am.Med.Womens Assoc., v. 52, p. 113-120.

55. Hajjar, I. and Kotchen, T. A., 2003, Trends in prevalence, awareness, treatment, and control of hypertension in the United States, 1988-2000: JAMA, v. 290, p. 199-206.

56. Hancock, E. W., Deal, B. J., Mirvis, D. M., Okin, P., Kligfield, P., and Gettes, L. S., 2009, AHA/ACCF/HRS Recommendations for the Standardization and Interpretation of the Electrocardiogram Part V: Electrocardiogram Changes Associated With Cardiac Chamber Hypertrophy A Scientific Statement From the American Heart Association Electrocardiography and Arrhythmias Committee, Council on Clinical Cardiology; the American College of Cardiology Foundation; and the Heart Rhythm Society Endorsed by the International Society for Computerized Electrocardiology: Journal of the American College of Cardiology, v. 53, p. 992-1002.

57. Hansson, L., Zanchetti, A., Carruthers, S. G., Dahlof, B., Elmfeldt, D., Julius, S., Menard, J., Rahn, K. H., Wedel, H., and Westerling, S., 1998, Effects of intensive blood-pressure lowering and low-dose aspirin in patients with

hypertension: principal results of the Hypertension Optimal Treatment (HOT) randomised trial. HOT Study Group: Lancet., v. 351, p. 1755-1762.

58. Haroun, M. K., Jaar, B. G., Hoffman, S. C., Comstock, G. W., Klag, M. J., and Coresh, J., 2003, Risk factors for chronic kidney disease: a prospective study of 23,534 men and women in Washington County, Maryland: J Am Soc Nephrol, v. 14, p. 2934-2941.

59. HDFP, 1979a, Five-year findings of the hypertension detection and follow-up program. I. Reduction in mortality of persons with high blood pressure, including mild hypertension. Hypertension Detection and Follow-up Program Cooperative Group: JAMA, v. 242, p. 2562-2571.

60. HDFP, 1979b, Five-year findings of the hypertension detection and follow-up program. II. Mortality by race-sex and age. Hypertension Detection and Follow-up Program Cooperative Group: JAMA, v. 242, p. 2572-2577.

61. HDFP, 1982, The effect of treatment on mortality in "mild" hypertension: results of the hypertension detection and follow-up program: The New England Journal of Medicine, v. 307, p. 976-980.

62. HOT, 1998, Cost effectiveness of intensive treatment of hypertension. Based on presentations by Donald S. Shepard, PhD; and Dominic Hodgkin, PhD: Am.J Manag.Care, v. 4, p. S765-S769.

63. Hsia, J., Margolis, K. L., Eaton, C. B., Wenger, N. K., Allison, M., Wu, L., LaCroix, A. Z., and Black, H. R., 2007, Prehypertension and cardiovascular disease risk in the Women's Health Initiative: Circulation, v. 115, p. 855-860.

64. Hsu, C. Y., McCulloch, C. E., Darbinian, J., Go, A. S., and Iribarren, C., 2005, Elevated blood pressure and risk of end-stage renal disease in subjects without baseline kidney disease: Archives of Internal Medicine, v. 165, p. 923-928.

65. Ikram, M. A., van, O. M., de Jong, F. J., Kors, J. A., Koudstaal, P. J., Hofman, A., Witteman, J. C., and Breteler, M. M., 2008, Unrecognized myocardial infarction in relation to risk of dementia and cerebral small vessel disease: Stroke, v. 39, p. 1421-1426.

66. Jackson, R., 2000, Guidelines on preventing cardiovascular disease in clinical practice: BMJ., v. 320, p. 659-661.

67. Julius, S., Alderman, M. H., Beevers, G., Dahlof, B., Devereux, R. B., Douglas, J. G., Edelman, J. M., Harris, K. E., Kjeldsen, S. E., Nesbitt, S., Randall, O. S., and Wright, J. T., 2004, Cardiovascular risk reduction in hypertensive black patients with left ventricular hypertrophy - The LIFE study: Journal of the American College of Cardiology, v. 43, p. 1047-1055.

68. Kaplan E, Goodglass F., and Weintraub S., 1983, Boston Naming Test: Philadelphia PA, Lea and Febiger.

69. Kawas, C., Segal, J., Stewart, W. F., Corrada, M., and Thal, L. J., 1994, A validation study of the Dementia Questionnaire: Arch.Neurol., v. 51, p. 901-906.

70. Kearney, P. M., Whelton, M., Reynolds, K., Muntner, P., Whelton, P. K., and He, J., 2005, Global burden of hypertension: analysis of worldwide data: The Lancet, v. 365, p. 217-223.

71. Keeley, E. C., Kadakia, R., Soman, S., Borzak, S., and McCullough, P. A., 2003, Analysis of long-term survival after revascularization in patients with chronic kidney disease presenting with acute coronary syndromes: Am.J Cardiol., v. 92, p. 509-514.

72. Kivipelto, M., Helkala, E. L., Hanninen, T., Laakso, M. P., Hallikainen, M., Alhainen, K., Soininen, H., Tuomilehto, J., and Nissinen, A., 2001a, Midlife vascular risk factors and late-life mild cognitive impairment: A population-based study: Neurology, v. 56, p. 1683-1689.

73. Kivipelto, M., Helkala, E. L., Hanninen, T., Laakso, M. P., Hallikainen, M., Alhainen, K., Soininen, H., Tuomilehto, J., and Nissinen, A., 2001b, Midlife vascular risk factors and late-life mild cognitive impairment: A population-based study: Neurology, v. 56, p. 1683-1689.

74. Kivipelto, M., Helkala, E. L., Laakso, M. P., Hanninen, T., Hallikainen, M., Alhainen, K., Soininen, H., Tuomilehto, J., and Nissinen, A., 2001c, Midlife vascular risk factors and Alzheimer's disease in later life: longitudinal, population based study: BMJ, v. 322, p. 1447-1451.

75. Klag, M. J., Whelton, P. K., Randall, B. L., Neaton, J. D., Brancati, F. L., Ford, C. E., Shulman, N. B., and Stamler, J., 1996, Blood pressure and end-stage renal disease in men: N.Engl.J.Med., v. 334, p. 13-18.

76. Klahr, S., Levey, A. S., Beck, G. J., Caggiula, A. W., Hunsicker, L., Kusek, J. W., and Striker, G., 1994, The effects of dietary protein restriction and blood-pressure control on the progression of chronic renal disease. Modification of Diet in Renal Disease Study Group: N.Engl.J.Med., v. 330, p. 877-884.

77. Knopman, D., Boland, L. L., Mosley, T., Howard, G., Liao, D., Szklo, M., McGovern, P., and Folsom, A. R., 2001, Cardiovascular risk factors and cognitive decline in middle-aged adults: Neurology, v. 56, p. 42-48.

78. Kshirsagar, A. V., Carpenter, M., Bang, H., Wyatt, S. B., and Colindres, R. E., 2006, Blood pressure usually considered normal is associated with an elevated risk of cardiovascular disease: Am.J Med, v. 119, p. 133-141.

79. Kuller, L. H., Margolis, K. L., Gaussoin, S. A., Bryan, N. R., Kerwin, D., Limacher, M., Wassertheil-Smoller, S., Williamson, J., and Robinson, J. G., 2010, Relationship of hypertension, blood pressure, and blood pressure control with white matter abnormalities in the Women's Health Initiative

Memory Study (WHIMS)-MRI trial: J Clin.Hypertens.(Greenwich.), v. 12, p. 203-212.

80. Lachin, J. M. and Foulkes, M. A., 1986, Evaluation of sample size and power for analyses of survival with allowance for nonuniform patient entry, losses to follow-up, noncompliance, and stratification: Biometrics, v. 42, p. 507-519.

81. Lakatos, E., 1988, Sample sizes based on the log-rank statistic in complex clinical trials: Biometrics, v. 44, p. 229-241.

82. Lawes, C. M., Vander, H. S., and Rodgers, A., 2008, Global burden of blood-pressure-related disease, 2001: Lancet, v. 371, p. 1513-1518.

83. Levey, A. S., Beto, J. A., Coronado, B. E., Eknoyan, G., Foley, R. N., Kasiske, B. L., Klag, M. J., Mailloux, L. U., Manske, C. L., Meyer, K. B., Parfrey, P. S., Pfeffer, M. A., Wenger, N. K., Wilson, P. W., and Wright, J. T., Jr., 1998, Controlling the epidemic of cardiovascular disease in chronic renal disease: what do we know? What do we need to learn? Where do we go from here? National Kidney Foundation Task Force on Cardiovascular Disease: Am.J Kidney Dis., v. 32, p. 853-906.

84. Levy, D., Larson, M. G., Vasan, R. S., Kannel, W. B., and Ho, K. K., 1996, The progression from hypertension to congestive heart failure: JAMA, v. 275, p. 1557-1562.

85. Lewington, S., Clarke, R., Qizilbash, N., Peto, R., and Collins, R., 2002, Age-specific relevance of usual blood pressure to vascular mortality: a meta-analysis of individual data for one million adults in 61 prospective studies: Lancet, v. 360, p. 1903-1913.

86. Liao, D., Cooper, L., Cai, J., Toole, J. F., Bryan, N. R., Hutchinson, R. G., and Tyroler, H. A., 1996, Presence and severity of cerebral white matter lesions and hypertension, its treatment, and its control. The ARIC Study. Atherosclerosis Risk in Communities Study: Stroke, v. 27, p. 2262-2270.

87. Lindholm, L. H., Carlberg, B., and Samuelsson, O., 2005, Should beta blockers remain first choice in the treatment of primary hypertension? A meta-analysis: Lancet, v. 366, p. 1545-1553.

88. Liu, L., Wang, J. G., Gong, L., Liu, G., and Staessen, J. A., 1998, Comparison of active treatment and placebo in older Chinese patients with isolated systolic hypertension. Systolic Hypertension in China (Syst-China) Collaborative Group: J Hypertens., v. 16, p. 1823-1829.

89. Longstreth, W. T., Jr., Manolio, T. A., Arnold, A., Burke, G. L., Bryan, N., Jungreis, C. A., Enright, P. L., O'Leary, D., and Fried, L., 1996, Clinical correlates of white matter findings on cranial magnetic resonance imaging of 3301 elderly people. The Cardiovascular Health Study: Stroke, v. 27, p. 1274-1282.

90. Luchsinger, J. A. and Mayeux, R., 2004, Cardiovascular risk factors and Alzheimer's disease: Curr.Atheroscler.Rep., v. 6, p. 261-266.

91. Luepker, R. V., Apple, F. S., Christenson, R. H., Crow, R. S., Fortmann, S. P., Goff, D., Goldberg, R. J., Hand, M. M., Jaffe, A. S., Julian, D. G., Levy, D., Manolio, T., Mendis, S., Mensah, G., Pajak, A., Prineas, R. J., Reddy, K. S., Roger, V. L., Rosamond, W. D., Shahar, E., Sharrett, A. R., Sorlie, P., and Tunstall-Pedoe, H., 2003, Case Definitions for Acute Coronary Heart Disease in Epidemiology and Clinical Research Studies: A Statement From the AHA Council on Epidemiology and Prevention; AHA Statistics Committee; World Heart Federation Council on Epidemiology and Prevention; the European Society of Cardiology Working Group on Epidemiology and Prevention; Centers for Disease Control and Prevention; and the National Heart, Lung, and Blood Institute: Circulation, v. 108, p. 2543-2549.

92. Macmahon, S., Peto, R., Cutler, J., Collins, R., Sorlie, P., Neaton, J., Abbott, R., Godwin, J., Dyer, A., and Stamler, J., 1990, Blood pressure, stroke, and coronary heart disease. Part 1, Prolonged differences in blood pressure: prospective observational studies corrected for the regression dilution bias: Lancet., v. 335, p. 765-774.

93. Magsi, H. and Malloy, T., 2005, Underrecognition of cognitive impairment in assisted living facilities: J.Am.Geriatr.Soc., v. 53, p. 295-298.

94. Mancia, G., De, B. G., Dominiczak, A., Cifkova, R., Fagard, R., Germano, G., Grassi, G., Heagerty, A. M., Kjeldsen, S. E., Laurent, S., Narkiewicz, K., Ruilope, L., Rynkiewicz, A., Schmieder, R. E., Struijker Boudier, H. A., Zanchetti, A., Vahanian, A., Camm, J., De, C. R., Dean, V., Dickstein, K., Filippatos, G., Funck-Brentano, C., Hellemans, I., Kristensen, S. D., McGregor, K., Sechtem, U., Silber, S., Tendera, M., Widimsky, P., Zamorano, J. L., Kjeldsen, S. E., Erdine, S., Narkiewicz, K., Kiowski, W., gabiti-Rosei, E., Ambrosioni, E., Cifkova, R., Dominiczak, A., Fagard, R., Heagerty, A. M., Laurent, S., Lindholm, L. H., Mancia, G., Manolis, A., Nilsson, P. M., Redon, J., Schmieder, R. E., Struijker-Boudier, H. A., Viigimaa, M., Filippatos, G., Adamopoulos, S., gabiti-Rosei, E., Ambrosioni, E., Bertomeu, V., Clement, D., Erdine, S., Farsang, C., Gaita, D., Kiowski, W., Lip, G., Mallion, J. M., Manolis, A. J., Nilsson, P. M., O'Brien, E., Ponikowski, P., Redon, J., Ruschitzka, F., Tamargo, J., van, Z. P., Viigimaa, M., Waeber, B., Williams, B., Zamorano, J. L., The task force for the management of arterial hypertension of the European Society of Hypertension, and The task force for the management of arterial hypertension of the European Society of Cardiology, 2007, 2007 Guidelines for the management of arterial hypertension: The Task Force for the Management of Arterial Hypertension of the European Society of Hypertension (ESH) and of the European Society of Cardiology (ESC): Eur.Heart J, v. 28, p. 1462-1536.

95. Mancia, G., Laurent, S., gabiti-Rosei, E., Ambrosioni, E., Burnier, M., Caulfield, M. J., Cifkova, R., Clement, D., Coca, A., Dominiczak, A., Erdine, S., Fagard, R., Farsang, C., Grassi, G., Haller, H., Heagerty, A., Kjeldsen, S. E., Kiowski, W., Mallion, J. M., Manolis, A., Narkiewicz, K., Nilsson, P.,

Olsen, M. H., Rahn, K. H., Redon, J., Rodicio, J., Ruilope, L., Schmieder, R. E., Struijker-Boudier, H. A., Van Zwieten, P. A., Viigimaa, M., and Zanchetti, A., 2009, Reappraisal of European guidelines on hypertension management: a European Society of Hypertension Task Force document: J Hypertens..

96. **McCullough, P. A., Jurkovitz, C. T., Pergola, P. E., McGill, J. B., Brown, W. W., Collins, A. J., Chen, S. C., Li, S., Singh, A., Norris, K. C., Klag, M. J., and Bakris, G. L., 2007, Independent components of chronic kidney disease as a cardiovascular risk state: results from the Kidney Early Evaluation Program (KEEP): Archives of Internal Medicine, v. 167, p. 1122-1129.**

97. **Nasreddine, Z. S., Phillips, N. A., Bedirian, V., Charbonneau, S., Whitehead, V., Collin, I., Cummings, J. L., and Chertkow, H., 2005, The montreal cognitive assessment, MoCA: A brief screening tool for mild cognitive impairment: Journal of the American Geriatrics Society, v. 53, p. 695-699.**

98. **National Collaborating Centre for Chronic Conditions. Hypertension: management in adults in primary care: pharmacological update. 2006. London, Royal College of Physicians.**
Ref Type: Report

99. **National Heart Foundation of Australia (National Blood Pressure and Vascular Disease Advisory Committee). Guide to management of hypertension 2008. 2009. *National Heart Foundation of Australia*.**
Ref Type: Report

100. **National Institute on Aging. Progress Report on Alzheimer's Disease. NIH Publication no. 00-4859. 2000. Department of Health and Human Services, Public Health Service.**
Ref Type: Report

101. **Neal, B., Macmahon, S., and Chapman, N., 2000, Effects of ACE inhibitors, calcium antagonists, and other blood-pressure-lowering drugs: results of prospectively designed overviews of randomised trials. Blood Pressure Lowering Treatment Trialists' Collaboration: Lancet, v. 356, p. 1955-1964.**

102. **Nilsson, S. E., Read, S., Berg, S., Johansson, B., Melander, A., and Lindblad, U., 2007, Low systolic blood pressure is associated with impaired cognitive function in the oldest old: longitudinal observations in a population-based sample 80 years and older: Aging Clin.Exp.Res., v. 19, p. 41-47.**

103. **Nissen, S. E., Tuzcu, E. M., Libby, P., Thompson, P. D., Ghali, M., Garza, D., Berman, L., Shi, H., Buebendorf, E., and Topol, E. J., 2004, Effect of antihypertensive agents on cardiovascular events in patients with coronary disease and normal blood pressure: the CAMELOT study: a randomized controlled trial: JAMA., v. 292, p. 2217-2225.**

104. Ong, K. L., Cheung, B. M., Man, Y. B., Lau, C. P., and Lam, K. S., 2007, Prevalence, awareness, treatment, and control of hypertension among United States adults 1999-2004: Hypertension, v. 49, p. 69-75.

105. Petersen, R. C., 2000, Aging, mild cognitive impairment, and Alzheimer's disease: Neurol.Clin., v. 18, p. 789-806.

106. Peterson, J. C., Adler, S., Burkart, J. M., Greene, T., Hebert, L. A., Hunsicker, L. G., King, A. J., Klahr, S., Massry, S. G., and Seifter, J. L., 1995, Blood pressure control, proteinuria, and the progression of renal disease. The Modification of Diet in Renal Disease Study: Ann.Intern Med, v. 123, p. 754-762.

107. Pfeffer, R. I., Kurosaki, T. T., Harrah, C. H., Jr., Chance, J. M., and Filos, S., 1982, Measurement of functional activities in older adults in the community: J Gerontol., v. 37, p. 323-329.

108. Plassman, B. L., Langa, K. M., Fisher, G. G., Heeringa, S. G., Weir, D. R., Ofstedal, M. B., Burke, J. R., Hurd, M. D., Potter, G. G., Rodgers, W. L., Steffens, D. C., Willis, R. J., and Wallace, R. B., 2007, Prevalence of dementia in the United States: the aging, demographics, and memory study: Neuroepidemiology, v. 29, p. 125-132.

109. PROGRESS Collaborative Group, 2001, Randomised trial of a perindopril-based blood-pressure-lowering regimen among 6105 individuals with previous stroke or transient ischaemic attack: The Lancet, v. 358, p. 1033-1041.

110. Prospective Studies Collaboration, 2002, Age-specific relevance of usual blood pressure to vascular mortality: a meta-analysis of individual data for one million adults in 61 prospective studies: The Lancet, v. 360, p. 1903-1913.

111. Psaty, B. M., Smith, N. L., Siscovick, D. S., Koepsell, T. D., Weiss, N. S., Heckbert, S. R., Lemaitre, R. N., Wagner, E. H., and Furberg, C. D., 1997, Health outcomes associated with antihypertensive therapies used as first-line agents. A systematic review and meta-analysis: JAMA, v. 277, p. 739-745.

112. Qiu, C., Winblad, B., and Fratiglioni, L., 2005, The age-dependent relation of blood pressure to cognitive function and dementia: Lancet Neurol., v. 4, p. 487-499.

113. Rahman, M., Pressel, S., Davis, B. R., Nwachuku, C., Wright, J. T., Jr., Whelton, P. K., Barzilay, J., Batuman, V., Eckfeldt, J. H., Farber, M. A., Franklin, S., Henriquez, M., Kopyt, N., Louis, G. T., Saklayen, M., Stanford, C., Walworth, C., Ward, H., and Wiegmann, T., 2006, Cardiovascular outcomes in high-risk hypertensive patients stratified by baseline glomerular filtration rate: Ann.Intern.Med., v. 144, p. 172-180.

114. Rashidi, A., Sehgal, A. R., Rahman, M., and O'Connor, A. S., 2008, The case for chronic kidney disease, diabetes mellitus, and myocardial infarction being equivalent risk factors for cardiovascular mortality in patients older than 65 years: Am.J Cardiol., v. 102, p. 1668-1673.

115. Reitan R.M. Validity of the trail making test as an indicator of organic brain damage. [8], 271-276. 1958. Percept Mot Skills.
Ref Type: Serial (Book,Monograph)

116. Reitz, C., Tang, M. X., Manly, J., Mayeux, R., and Luchsinger, J. A., 2007, Hypertension and the risk of mild cognitive impairment: Arch.Neurol., v. 64, p. 1734-1740.

117. Rosamond, W. D., Chang, P., Baggett, C., Bertoni, A., Shahar, E., Deswal, A., Heiss, G., and Chambless, L., 2009, Classification of Heart Failure in the Atherosclerosis Risk in Communities (ARIC) Study: A Comparison With Other Diagnostic Criteria: Circulation, v. 120, p. S506.

118. Rosano, C., Kuller, L. H., Chung, H., Arnold, A. M., Longstreth, W. T., Jr., and Newman, A. B., 2005, Subclinical brain magnetic resonance imaging abnormalities predict physical functional decline in high-functioning older adults: J.Am.Geriatr.Soc., v. 53, p. 649-654.

119. Rosendorff, C., Black, H. R., Cannon, C. P., Gersh, B. J., Gore, J., Izzo, J. L., Kaplan, N. M., O'Connor, C. M., O'Gara, P. T., and Oparil, S., 2007, Treatment of hypertension in the prevention and management of ischemic heart disease - A scientific statement from the American Heart Association council for high blood pressure research and the councils on clinical cardiology and epidemiology and prevention: Hypertension, v. 50, p. E28-E55.

120. Sacco, R. L., Boden-Albala, B., Abel, G., Lin, I. F., Elkind, M., Hauser, W. A., Paik, M. C., and Shea, S., 2001, Race-ethnic disparities in the impact of stroke risk factors: the northern Manhattan stroke study: Stroke., v. 32, p. 1725-1731.

121. Sarnak, M. J., Greene, T., Wang, X., Beck, G., Kusek, J. W., Collins, A. J., and Levey, A. S., 2005, The effect of a lower target blood pressure on the progression of kidney disease: long-term follow-up of the modification of diet in renal disease study: Ann.Intern Med, v. 142, p. 342-351.

122. Sarnak, M. J., Levey, A. S., Schoolwerth, A. C., Coresh, J., Culleton, B., Hamm, L. L., McCullough, P. A., Kasiske, B. L., Kelepouris, E., Klag, M. J., Parfrey, P., Pfeffer, M., Raij, L., Spinosa, D. J., and Wilson, P. W., 2003, Kidney Disease as a Risk Factor for Development of Cardiovascular Disease: A Statement From the American Heart Association Councils on Kidney in Cardiovascular Disease, High Blood Pressure Research, Clinical Cardiology, and Epidemiology and Prevention: Circulation, v. 108, p. 2154-2169.

123. Schaeffner, E. S., Kurth, T., Bowman, T. S., Gelber, R. P., and Gaziano, J. M., 2008, Blood pressure measures and risk of chronic kidney disease in men: Nephrol.Dial.Transplant, v. 23, p. 1246-1251.

124. SHEP, 1991, Prevention of stroke by antihypertensive drug treatment in older persons with isolated systolic hypertension. Final results of the Systolic Hypertension in the Elderly Program (SHEP). SHEP Cooperative Research Group: JAMA, v. 265, p. 3255-3264.

125. Shlipak, M. G., Heidenreich, P. A., Noguchi, H., Chertow, G. M., Browner, W. S., and McClellan, M. B., 2002, Association of renal insufficiency with treatment and outcomes after myocardial infarction in elderly patients: Ann.Intern Med, v. 137, p. 555-562.

126. Shlipak, M. G., Katz, R., Kestenbaum, B., Siscovick, D., Fried, L., Newman, A., Rifkin, D., and Sarnak, M. J., 2009, Rapid decline of kidney function increases cardiovascular risk in the elderly: J Am.Soc.Nephrol., v. 20, p. 2625-2630.

127. Shumaker, S. A., Legault, C., Kuller, L., Rapp, S. R., Thal, L., Lane, D. S., Fillit, H., Stefanick, M. L., Hendrix, S. L., Lewis, C. E., Masaki, K., and Coker, L. H., 2004, Conjugated equine estrogens and incidence of probable dementia and mild cognitive impairment in postmenopausal women: Women's Health Initiative Memory Study: JAMA, v. 291, p. 2947-2958.

128. Sipahi, I., Tuzcu, E. M., Schoenhagen, P., Wolski, K. E., Nicholls, S. J., Balog, C., Crowe, T. D., and Nissen, S. E., 2006, Effects of normal, pre-hypertensive, and hypertensive blood pressure levels on progression of coronary atherosclerosis: J Am.Coll.Cardiol., v. 48, p. 833-838.

129. Skoog, I. and Gustafson, D., 2006, Update on hypertension and Alzheimer's disease: Neurol.Res., v. 28, p. 605-611.

130. Skoog, I. and Gustafson, D., 2003, Hypertension, hypertension-clustering factors and Alzheimer's disease: Neurol.Res., v. 25, p. 675-680.

131. Skoog, I., Lithell, H., Hansson, L., Elmfeldt, D., Hofman, A., Olofsson, B., Trenkwalder, P., and Zanchetti, A., 2005, Effect of baseline cognitive function and antihypertensive treatment on cognitive and cardiovascular outcomes: Study on COgnition and Prognosis in the Elderly (SCOPE): Am.J Hypertens., v. 18, p. 1052-1059.

132. Somes, G. W., Pahor, M., Shorr, R. I., Cushman, W. C., and Applegate, W. B., 1999, The role of diastolic blood pressure when treating isolated systolic hypertension: Archives of Internal Medicine, v. 159, p. 2004-2009.

133. Somes, G. W., Shorr, R. I., and Pahor, M., 1999, A new twist in the J-shape curve?: J Am.Geriatr.Soc., v. 47, p. 1477-1478.

134. Staessen, J. A., Fagard, R., Thijs, L., Celis, H., Arabidze, G. G., Birkenhager, W. H., Bulpitt, C. J., de Leeuw, P. W., Dollery, C. T., Fletcher, A. E., Forette,

F., Leonetti, G., Nachev, C., O'Brien, E. T., Rosenfeld, J., Rodicio, J. L., Tuomilehto, J., and Zanchetti, A., 1997, Randomised double-blind comparison of placebo and active treatment for older patients with isolated systolic hypertension. The Systolic Hypertension in Europe (Syst-Eur) Trial Investigators: Lancet, v. 350, p. 757-764.

135. Staessen, J. A., Wang, J. G., and Thijs, L., 2001, Cardiovascular protection and blood pressure reduction: a meta-analysis: Lancet, v. 358, p. 1305-1315.

136. Staessen, J. A., Wang, J. G., Thijs, L., and Fagard, R., 1999, Overview of the outcome trials in older patients with isolated systolic hypertension: J Hum.Hypertens., v. 13, p. 859-863.

137. The ACCORD Study Group, 2010, Effects of Intensive Blood-Pressure Control in Type 2 Diabetes Mellitus: The New England Journal of Medicine, p. NEJMoa1001286.

138. Tzourio, C., Anderson, C., Chapman, N., Woodward, M., Neal, B., Macmahon, S., and Chalmers, J., 2003, Effects of blood pressure lowering with perindopril and indapamide therapy on dementia and cognitive decline in patients with cerebrovascular disease: Arch.Intern Med, v. 163, p. 1069-1075.

139. Vasan, R. S., Larson, M. G., Leip, E. P., Evans, J. C., O'Donnell, C. J., Kannel, W. B., and Levy, D., 2001, Impact of high-normal blood pressure on the risk of cardiovascular disease: N Engl J Med, v. 345, p. 1291-1297.

140. Verdelho, A., Madureira, S., Ferro, J. M., Basile, A. M., Chabriat, H., Erkinjuntti, T., Fazekas, F., Hennerici, M., O'brien, J., Pantoni, L., Salvadori, E., Scheltens, P., Visser, M. C., Wahlund, L. O., Waldemar, G., Wallin, A., and Inzitari, D., 2007, Differential impact of cerebral white matter changes, diabetes, hypertension and stroke on cognitive performance among non-disabled elderly. The LADIS study: J Neurol.Neurosurg.Psychiatry, v. 78, p. 1325-1330.

141. Vermeer, S. E., Prins, N. D., den, H. T., Hofman, A., Koudstaal, P. J., and Breteler, M. M., 2003, Silent brain infarcts and the risk of dementia and cognitive decline: N Engl J Med, v. 348, p. 1215-1222.

142. Wang, Y. and Wang, Q. J., 2004, The prevalence of prehypertension and hypertension among US adults according to the new joint national committee guidelines: new challenges of the old problem: Arch.Intern Med, v. 164, p. 2126-2134.

143. Wechsler D., 1981, Manual for the Wechsler Adult Intelligence Scale: The Psychological Corporation, Harcourt Brace Janvanovich, Inc..

144. Weiner, D. E., Tighiouart, H., Amin, M. G., Stark, P. C., MacLeod, B., Griffith, J. L., Salem, D. N., Levey, A. S., and Sarnak, M. J., 2004, Chronic kidney disease as a risk factor for cardiovascular disease and all-cause mortality:

a pooled analysis of community-based studies: J.Am.Soc.Nephrol., v. 15, p. 1307-1315.

145. Weiner, D. E., Tighiouart, H., Levey, A. S., Elsayed, E., Griffith, J. L., Salem, D. N., and Sarnak, M. J., 2007, Lowest systolic blood pressure is associated with stroke in stages 3 to 4 chronic kidney disease: J.Am.Soc.Nephrol., v. 18, p. 960-966.

146. Welsh, K. A., Breitner, J. C. S., and Magruderhabib, K. M., 1993, Detection of Dementia in the Elderly Using Telephone Screening of Cognitive Status: Neuropsychiatry Neuropsychology and Behavioral Neurology, v. 6, p. 103-110.

147. Whitworth, J. A., 2003, 2003 World Health Organization (WHO)/International Society of Hypertension (ISH) statement on management of hypertension: J Hypertens., v. 21, p. 1983-1992.

148. Winblad, B., Palmer, K., Kivipelto, M., Jelic, V., and Fratiglioni, L., 2004, Introduction: Mild cognitive impairment: beyond controversies, towards a consensus: Journal of Internal Medicine, v. 256, p. 181-182.

149. World Health Organization. World Health Report 2002: Reducing Risk, promoting healthy life. 2002. World Health Organization.
Ref Type: Report

150. Wright, J. T., Jr., Agodoa, L., Contreras, G., Greene, T., Douglas, J. G., Lash, J., Randall, O., Rogers, N., Smith, M. C., and Massry, S., 2002a, Successful blood pressure control in the African American Study of Kidney Disease and Hypertension: Arch.Intern.Med., v. 162, p. 1636-1643.

151. Wright, J. T., Jr., Bakris, G., Greene, T., Agodoa, L. Y., Appel, L. J., Charleston, J., Cheek, D., Douglas-Baltimore, J. G., Gassman, J., Glassock, R., Hebert, L., Jamerson, K., Lewis, J., Phillips, R. A., Toto, R. D., Middleton, J. P., and Rostand, S. G., 2002b, Effect of blood pressure lowering and antihypertensive drug class on progression of hypertensive kidney disease: results from the AASK trial: JAMA, v. 288, p. 2421-2431.

152. Wright, J. T., Dunn, J. K., Cutler, J. A., Davis, B. R., Cushman, W. C., Ford, C. E., Haywood, L. J., Leenen, F. H. H., Margolis, K. L., Papademetriou, V., Probstfield, J. L., Whelton, P. K., and Habib, G. B., 2005, Outcomes in hypertensive black and nonblack patients treated with chlorthalidone, amlodipine, and lisinopril: Jama-Journal of the American Medical Association, v. 293, p. 1595-1607.

153. Wright, J. T., Harris-Haywood, S., Pressel, S., Barzilay, J., Bairnbridge, C., Bareis, C. J., Basile, J. N., Black, H. R., Dart, R., Gupta, A. K., Hamilton, B. P., Einhorn, P. T., Haywood, L. J., Jafri, S. Z. A., Louis, G. T., Whelton, P. K., Scott, C. L., Sinnnons, D. L., Stanford, C., and Davis, B. R., 2008, Clinical outcomes by race in hypertensive patients with and without the metabolic syndrome - Antihypertensive and Lipid-Lowering Treatment to Prevent

Heart Attack Trial (ALLHAT): Archives of Internal Medicine, v. 168, p. 207-217.

154. Yusuf, S., Sleight, P., Pogue, J., Bosch, J., Davies, R., and Dagenais, G., 2000, Effects of an angiotensin-converting-enzyme inhibitor, ramipril, on cardiovascular events in high-risk patients. The Heart Outcomes Prevention Evaluation Study Investigators [published errata appear in N Engl J Med 2000 Mar 9;342(10):748 and 2000 May 4;342(18):1376]: N Engl J Med, v. 342, p. 145-153.

APPENDIX 1: Abbreviations Used

AAA:	Abdominal Aortic Aneurysm
AASK:	African American Study of Kidney Disease and Hypertension
ABI:	Ankle Brachial Index
ACC:	American College of Cardiology
ACCORD:	Action to Control Cardiovascular Risk in Diabetes
ACE:	Angiotensin Converting Enzyme
ACR:	Albumin to Creatinine Ratio
ACS:	Acute Coronary Syndrome
AD:	Alzheimer's Disease
AE:	Adverse Event
AHA:	American Heart Association
ALLHAT:	Antihypertensive and Lipid-Lowering Treatment to Prevent Heart Attack Trial
ARB:	Angiotensis Receptor Blocker
ARIC:	Atherosclerosis Risk in Communities
AS:	Ancillary Science
ASCOT:	Anglo-Scandinavian Cardiac Outcomes Trial
BID:	Twice Daily
BNT:	Boston Naming Test
BP:	Blood Pressure
BPH:	Benign Prostatic Hyperplasia
CABG:	Coronary Artery Bypass Grafting
CAD:	Coronary Artery Disease
CAMELOT:	Comparision of Amlodipine vs Enalapril to Limit Occurrences of Thrombosis Trial
CC:	Coordinating Center
CCB:	Calcium Channel Blockers
CCN:	Clinical Center Network
CE:	Carotid Endarterectomy
CEA:	Cost-Effectiveness Analysis
CHD:	Coronary Heart Disease
CHF:	Chronic Heart Failure
CHS:	Cardiovascular Health Study
CKD:	Chronic Kidney Disease
Co-PI:	Co-Principal Investigator
CPT:	Current Procedural Terminology
CUA:	Cost-Utility Analysis
CV:	Cardiovascular
CVD:	Cardiovascular Disease
DASH:	Dietary Approaches to Stop Hypertension
DBP:	Diastolic Blood Pressure
DDC:	Drug Distribution Center
DHP:	Dihydropyridine
DQ:	Dementia Questionnaire
DSC:	Digit Symbol Coding test
DSMB:	Data Safety Monitoring Board
DSM-IV:	Diagnostic and Statistical Manual of Mental Disorders - Fourth Edition
DSST:	Digit Symbol Substitution Test
DST:	Digit Span Test
ECG:	Electrocardiogram
ED:	Erectile Dysfunction
eGFR:	Estimated Glomerular Filtration Rate
EnaC Inhibitor:	Epithelial Sodium Channel Inhibitor
EPICARE:	Epidemiological Cardiology Research Center
EQ-5D:	EuroQol 5 Dimensional Descriptive System
ESRD:	End Stage Renal Disease
EUROPA:	European Trial on Reduction of Cardiac Events with Perindopril in Stable Coronary Artery Disease
FAQ:	Functional Activities Questionnaire
FDA:	Food and Drug Administration
FES-I:	Falls Self-Efficacy Scale International
FRS:	Framingham Risk Score
FSFI:	Female Sexual Function Assessment
GCP:	Good Clinical Practice
GEMS:	Gingko Evaluation of Memory Study
GFR:	Glomerular Filtration Rate
GXT:	Graded Exercise Test
HDFP:	Hypertension Detection and Follow-up Program
HF:	Heart Failure
HIPAA:	Health Information Portability and Accountability Act
HOPE:	Hospital Outcomes Project for the Elderly

HOT: Hypertension Optimal Treatment trial
HRQL: Health Related Quality of Life
HTN: Hypertension
HVLT: Hopkins Verbal Learning Test
HYVET: Hypertension in the Very Elderly Trial
HYVET COG: Hypertension in the Very Elderly Trial – cognitive function assessment
ICER: Incremental Cost-Effectiveness Ratio
ID: Identification
IIEF: International Index of Erectile Function
IRB: Institutional Review Board
ISH: Isolated Systolic Hypertension
JNC: Joint National Committee
JNC-7: The Seventh Report of the Joint National Committee on Prevention, Detection, Evaluation, and Treatment of High Blood Pressure
LMT: Logical Memory Test
LVH: Left Ventricular Hypertrophy
MAP: Mean Arterial Pressure
MAR: Missing-at-Random Analyses
MCI: Mild Cognitive Impairment
MDRD: Modification of Diet in Renal Disease Study
MI: Myocardial Infarction
MIND: Memory and Cognition In Decreased Hypertension
MoCA: Montreal Cognitive Assessment
MOP: Manual of Procedures
MPQC: Measurement Procedures and Quality Control
mRey-O: Modified Rey-Osterrieth Complex Figure
MRI: Magnetic Resonance Imaging
NEJM: New England Journal of Medicine
NKF: National Kidney Foundation
NHANES: National Health and Nutrition Examination Survey
NHLBI: National Heart, Lung, and Blood Institute
NIA: National Institute on Aging
NIDDK: National Institute of Diabetes and Digestive and Kidney Diseases
NIH: National Institutes of Health
NINDS: National Institute of Neurological Disorders and Stroke
OH: Orthostatic Hypotension
P&P: Publications and Presentations
PAD: Peripheral Artery Disease
PCI: Percutaneous Coronary Intervention
PEACE: Prevention of Events with Angiotensin Coverting Enzyme
PHI: Private Health Information
PHQ: Patient Health Questionnaire
PI: Principal Investigator
PKD: Polycystic Kidney Disease
PROGRESS: Perindopril Protection Against Recurrent Stroke Study
PTS: Participant Tracking System
QALY: Quality Adjusted Life Years
QC: Quality Control
RAAS: Renin-angiotensin-aldosteribe system
RAS: Renin Angiotensin System
SAE: Serious Adverse Event
SBP: Systolic Blood Pressure
SCOPE: Study on Cognition and Prognosis in the Elderly
SHEP: Systolic Hypertension in the Elderly Program
SPRINT: Systolic Blood Pressure Intervention Trial
SPRINT MIND: SPRINT Memory and Cognition In Decreased Hypertension
SSL: Secure Socket Layer
SVID: Small Vessel Ischemic Disease
Syst-Eur: Systolic Hypertension in Europe Trial
TICS-M: Modified Telephone Interview for Cognitive Status
TMT: Trail Making Test
UKPDS: United Kingdom Prospective Diabetes Study
WHI: Women's Health Initiative
WHIMS: Women's Health Initiative Memory Study
WWW: World Wide Web

APPENDIX 2: Computational Details and Sensitivity Analyses for the CVD outcome

Power computations were developed using event rates observed in ALLHAT. The ALLHAT Coordinating Center provided us with summary data across all three arms allowing us to calculate event rates using different combinations of baseline characteristics. Event rates were calculated using a composite outcome including fatal CVD, non-MI acute coronary syndrome, and nonfatal MI, stroke, and heart failure. For ALLHAT participants without diabetes, the annual event rate was 4.39 %/yr. (Note: ALLHAT used hospitalized angina rather than non-MI acute coronary syndrome.)

This rate of 4.39 %/yr provides a starting point for the estimation of event rates we will expect in SPRINT. Several factors can be considered which suggest that these rates should be either increased or decreased. Factors arguing for an increased event rate include (1) SPRINT will have an older cohort of participants than did ALLHAT, (2) SPRINT will use the Framingham risk score of ≥15% 10-year CVD risk as an inclusion criterion, and (3) inclusion of a substantial group of participants with Stage 3 or Stage 4 CKD. Factors that are expected to reduce the event rate include (1) the temporal trend towards a reduction in CVD event rates in the U.S. and (2) a more rigorous definition of non-MI acute coronary syndrome that will be used in SPRINT. It is difficult to precisely estimate the impact that these five factors will have on the SPRINT event rate.

In ALLHAT, event rates increased substantially with age. The event rate for participants 70 to <75 years old was 5.19 %/yr; for participants ≥75 years old, the event rate was 6.99 %/yr. In ALLHAT 17.7% of the participants were 70 to <75 years old, while 18.5% were ≥75 years old. We expect that participants in these age categories will represent a greater fraction of the SPRINT cohort. Approximately 50% (4625 participants) are expected to be at least 70 years old, while 35.1% (3250 participants) are expected to be ≥75 years old. This will likely yield a higher event rate in SPRINT, compared to ALLHAT.

The event rate in ALLHAT among participants with 10-year Framingham risk ≥15% at baseline was 4.67 %/yr. Our including people with ≥15% 10-year risk will help to ensure a higher event rate.

We expect that 4300 SPRINT participants will have eGFR 20 to <60 mL/min/1.73m^2 with equal numbers above and below 45 mL/min/1.73m^2. In ALLHAT, the event rate was 5.89 %/yr for those with eGFR 45 to <60 mL/min/1.73m^2. Among those <45, the event rate was 8.24 %/yr. In ALLHAT, 18.6% had eGFR <60 mL/min/1.73m^2 as compared with the expected 46.7% in SPRINT. Increasing the numbers of participants with CKD in SPRINT will help increase the event rates.

We compared ALLHAT participants with diabetes to participants in the ACCORD BP trial (all of whom have diabetes) using outcome variables that are as similar as possible. In ALLHAT the event rate was 5.90 %/yr. The corresponding event rate in ACCORD was 3.43 %/yr. The reduction in event rates between ALLHAT and ACCORD could be due to a temporal trend (ALLHAT was 1994—1999, ACCORD was 2001—2009), because ALLHAT participants were older (mean 67 years) than ACCORD (mean 62.2 years), or for other reasons.

Exactly how we should use the ALLHAT data to estimate the event rates for SPRINT is unclear. Since the rates in ACCORD were approximately half of those in ALLHAT, *for the purposes of*

power we will assume that the SPRINT rates will also be half of the ALLHAT rates. This assumption balances the possibility of a further temporal trend in event rate reduction with the fact that participants recruited for SPRINT will be older, have lower kidney function, and have greater Framingham CVD risk scores than those recruited in either ALLHAT or ACCORD. We expect that this may be slightly conservative. Thus, we assume that the event rate in SPRINT will be approximately 2.2 %/yr for the composite outcome including non-fatal MI, non-fatal stroke, cardiovascular death, hospitalized heart failure, and non-MI acute coronary syndrome.

We have assumed a 2-year uniform accrual period, 3 years 10 months minimum follow-up (assumes that closeout visits occur uniformly over a 4-month period), and a 2 sided significance level of 0.05. The effect size for the primary outcome is assumed to be 20% in the entire sample and the CKD subsample, and 25% in the Senior subsample. Loss to follow-up and events are assumed to follow an exponential model. We expect that the annual rate of loss to follow-up will be approximately 2% but have included rates up to 3% to be conservative. Calculations made using two methods (Lachin and Foulkes, 1986;Lakatos, 1988) were similar. Power for the primary outcome for a range of event rates and annual loss rates is presented in Table 1 for the assumed effect size of 20%.

Table 1. Power for the primary outcome in entire sample of 9250 participants for a 20% effect (Hazard Ratio of 0.8).

Annual Loss Rate (%/yr)	Annual Standard Arm Event Rate (%/yr)				
	1.8	2.0	2.2	2.4	2.6
1	82.9	86.5	89.4	91.7	93.5
2	82.0	85.7	88.7	91.0	93.0
3	81.1	84.8	87.9	90.4	92.4

In ALLHAT the event rates were 5.89 %/yr and 8.24 %/yr for people whose eGFR was 45 to <60 or <45 mL/min/1.73m^2. We will assume that the event rate for the primary outcome in SPRINT will be 4 %/yr among participants with eGFR <60 mL/min/1.73m^2. Power for the primary outcome among SPRINT participants with CKD for a range of event rates and annual loss rates is presented in Table 2 for the assumed effect size of 20%.

Table 2. Power for the primary outcome in CKD subsample (eGFR < 60 mL/min/1.73m^2) of 4300 participants for a 20% effect (Hazard Ratio of 0.8).

Annual Loss Rate (%/yr)	Annual Standard Arm Event Rate (%/yr)				
	3.5	3.75	4.0	4.25	4.5
1	77.9	80.5	82.7	84.8	86.6
2	76.9	79.5	81.9	83.9	85.8
3	75.9	78.6	80.9	83.1	85.0

In ALLHAT, the event rate was 6.99 %/yr among participants at least 75 years old. Applying the same halving as was done above for the entire sample, we will assume that the event rate in SPRINT will be 3.5 %/year among participants ≥75 years old. Power for the primary outcome among SPRINT Senior for a range of event rates and annual loss rates is presented in Table 3 for the assumed effect size of 25%.

Table 3. Power for the primary outcome in Senior subsample (≥75 years old) of 3250 participants for a 25% effect (Hazard Ratio of 0.75).					
Annual Loss Rate (%/yr)	Annual Standard Arm Event Rate (%/yr)				
	3.0	3.25	3.5	3.75	4.0
1	79.9	82.8	85.3	87.5	89.4
2	79.0	81.9	84.5	86.7	88.6
3	78.0	81.0	83.6	85.9	87.9

APPENDIX 3: Computational Details and Sensitivity Analyses for the MIND outcomes

Dementia. The primary outcome for SPRINT MIND is all-cause dementia. Table 1 summarizes dementia rates from HYVET-COG (Peters, 2008), the Ginkgo Evaluation of Memory Study (GEMS) (DeKosky, 2008), the Cardiovascular Health Study (CHS) (Fitzpatrick, 2004) and the Women's Health Initiative Memory Study (WHIMS) (Shumaker, 2004). In HYVET-COG, there was a 14% non-significant decline in dementia. Overall annual dementia rate varied from 0.13% to 3.86%. The Women's Health Initiative Memory Study (WHIMS) (Shumaker, 2004) recruited women 65 and older with a mean age of 69 in two hormone replacement therapy interventions. Both trials were stopped early because of unexpected increased health risks in women receiving the hormone therapy. Of the studies reported here, WHIMS may be the least similar to SPRINT.

Table 1. Annual rates of dementia from previous studies.

Age	eGFR	HYVET-COG	GEMS	CHS	WHIMS
<75				1.29	0.08
75+			3.09 (3.86)[1]	4.55	0.81
	<45		4.87 (6.39)		
	45-59.9		3.02 (3.20)		
	60-89.9		2.87 (3.70)		
	90+		3.86 (4.51)		
80+		3.50			
ALL		3.50	3.09 (3.86)	2.62	0.13

1 With prior CVD

Based on these data and the expected number of SPRINT participants 75 or older, and with CKD or MCI at baseline, we expect the annual event rate in SPRINT to be 3.1%-3.5%. In meta-analyses performed by the HYVET investigators, three of the four trials had hazard ratios ranging from 0.84 to 0.90. A reasonable goal for SPRINT MIND is to detect a relative difference between arms expressed by a hazard ratio of 0.5 to 0.8 for dementia. Using a 2-sided proportional hazards regression test of time until first incidence of dementia, we can expect at least 79% power for annual dementia rates of 3.1%-3.5% and an effect size of 0.15 and 96% power for annual dementia rates of 3.2%-3.5% and an effect size of 0.20.

Cognitive Function. SPRINT will include 2,800 participants receiving the extended cognitive battery at baseline, and years 2 and 4 post randomization. We obtained the standard deviations for several of the tests included in the SPRINT battery to determine detectable differences. The standard deviation for the Digit Symbol Substitution Test is from actual ACCORD MIND data 40 months post randomization adjusted for baseline and stratifying factors. Actual means were not available so we used the ACCORD MIND assumptions in their sample size calculations based on CHS data. GEMS provided us with standard deviations and means for Trails A & B, Digit Span and the Boston Naming Test. Table 2 shows that we can detect mean differences for

each test of 5.1% or less between the two SPRINT treatment groups at year 4, with 90% statistical power, assuming 3%/year loss to follow-up. The statistical power will even be increased when combining the scores for these tests in each domain.

Table 2. Means, standard deviations and power for cognitive tests.

**Cognitive Test**	**Mean (STD)**	**Power**	
		80%	90%
Effect Size		0.114	0.132
Digit Symbol Substitution Test	39.5[1] (7.9)[2]	0.90 (2.4%)	1.05 (2.7%)
Trails A[3]	47.5 (18.1)	2.07 (4.4%)	2.40 (5.1%)
Trails B[3]	124.4 (40.6)	4.65 (3.7%)	5.38 (4.3%)
Digit Span[3]	13.9 (2.6)	0.30 (2.2%)	0.34 (2.4%)
Boston Naming Test[3]	26.2 (2.6)	0.30 (1.1%)	0.34 (1.3%)

[1] From ACCORD MIND assumptions in sample size calculations based on CHS data
[2] From actual ACCORD MIND data at 40 months post randomization
[3] From GEMS at 48 months post randomization

MRI. We will perform MRI in 640 of SPRINT MIND participants. The standard deviations for total abnormal tissue volume and total brain volume from the ACCORDMIND study 40 months post randomization adjusted for baseline and cranial size are 2.77 cm^3 and 16.45 cm^3. The final analysis of the MRI data collected in SPRINT MIND will compare the mean total abnormal tissue and mean total brain volumes between the groups, controlling for the baseline MRI value and cranial side. With 640 participants (320 participants in each treatment group), after accounting for a 3%/yr loss to-follow-up, and assuming a 0.05 two-sided significance level, we will be able to detect group differences in total abnormal vascular lesion volumes of 0.65 cm^3 and 0.76 cm^3, and in total brain volumes of 3.9 cm^3 and 4.5 cm^3 over 4 years, with 80% and 90% power, respectively.

APPENDIX 4: SPRINT Organizational Chart
Committees and Subcommittees

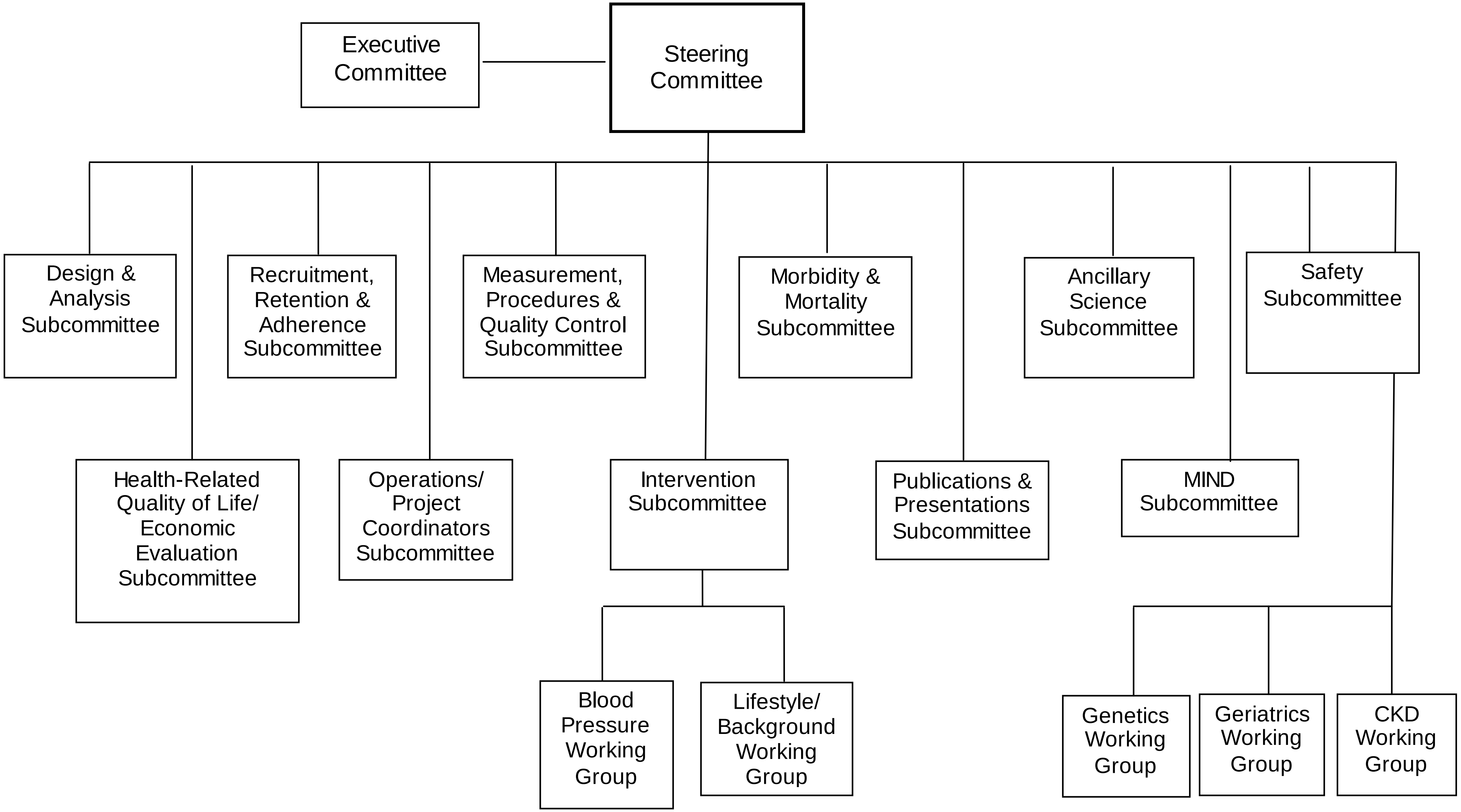

APPENDIX 5

SPRINT Charges & Membership of Committees & Subcommittees

Below are the charges of the committees and subcommittees to the Steering Committee. Each subcommittee will assume additional responsibilities as deemed necessary by the SPRINT Steering or Executive Committee.

SPRINT Steering Committee (SC) provides the overall leadership for the trial and establishes the scientific and administrative policies. It will be led by the independent Study Chair, who is also the Chair of the Steering Committee. The Vice Chair of the Steering Committee, who may be a CCN or a clinical site PI, will be a permanent SC member and also will be the Vice Chair of the Executive Committee. Other members of the Steering Committee include the Principal Investigators (PIs) from the Clinical Center Networks (CCNs), NIH representatives (from the NHLBI, NIDDK, NIA and NINDS), Coordinating Center (CC) staff, and other subcommittee chairs as needed. This committee oversees the overall conduct of the trial throughout all phases. The SC provides the leadership for the trial design, the protocol, Manual of Procedures (MOP), and study forms, all of which require final SC approval. This committee oversees recruitment, intervention, follow-up, and data collection practices and procedures to identify and correct deficiencies. They will consider adopting changes in the study protocol or procedures as necessary during the course of the SPRINT trial. Voting members will include the CCN PIs, the CC PI, and the NIH Project Office (which includes the joint interests of the four NIH funding institutions – NHLBI, NIDDK, NIA, and NINDS). The Steering Committee Chair will vote in the case of a tie.

SPRINT Executive Committee (EC) is the operational arm of the Steering Committee and makes decisions on behalf of the Steering Committee (SC) on day-to-day operational issues that require immediate action. This committee will consist of the Study Chair, SC Vice Chair, CC PI, NIH Project Office staff, Drug Distribution Center director, Project Coordinators/ Operations Subcommittee Chair, one rotating CCN PI, CC Program Coordinator, CC staff, and other subcommittee chairs as needed. This committee will meet by conference call every other week or as needed. The Executive Committee will develop the SC meeting agenda and timeline for completion of tasks. Important study issues, protocol changes, and other items will be discussed by the EC prior to presentation to the full SC for review and approval.

SPRINT Conflict of Interest Committee: This committee reviews potential conflict of interest issues. The NIH Project Office, Steering Committee Chair, and CC Chair comprise this committee, which has the overall responsibility for the trial's ethical oversight policy and procedures.

Subcommittees:

In general, each subcommittee will have representative(s) from the Coordinating Center, from each CCN, and from the NIH Project Office. Together the Steering Committee and each subcommittee should determine the expertise required for the given subcommittee. For example, the Intervention Subcommittee should include experts in hypertension, nephrology, neurology, and geriatrics. In addition, the various subcommittees may form working groups to address major issues within their charge (e.g., Genetics Working Group, CKD Working Group). The subcommittee and the CC will decide what periodic reports the subcommittee needs to perform its charge.

Ancillary Science Subcommittee (AS): This subcommittee is charged with developing procedures for review and approval by the SC for ancillary studies and substudies. The AS will review proposals for feasibility and compatibility with the main study protocol and aims. Specific evaluation criteria include participant and study burden. There will be substantial statistical support to the development of ancillary studies through this committee. It is suggested to have all 5 CCNs represented on this committee.

Design and Analysis Subcommittee (D&A): This subcommittee will review the currently proposed and alternative designs for the trial, including the analysis plan, the impact on sample size, statistical power and patient recruitment, as well as sequential monitoring, subgroup monitoring, and adjustments for multiple comparisons. This subcommittee will work closely with the Intervention Subcommittee and the Recruitment, Retention and Adherence Subcommittee on the development of analysis plans for recruitment and adherence monitoring.

Economic Evaluation/Health Related Quality of Life Subcommittee: This subcommittee will develop the protocol for the economic evaluation of the SPRINT interventions and the protocol for assessing the impact of these interventions on health-related quality of life. This will allow the study to estimate overall costs, cost effectiveness and cost utility for the SPRINT interventions. This subcommittee also will train the CCNs regarding collection of human resource costs, quality of life data and plans for analyses of these data, and provide interim reports to the SC.

Intervention Subcommittee: This subcommittee is charged with generating all of the blood pressure (BP) intervention plans for the trial, including materials, medications, titration algorithms and schedules, visit schedules, adherence strategies to the medications protocol and all BP monitoring including reports. This committee will consider issues concerning the SPRINT intervention on high-risk groups such as the elderly, CKD patients, and groups at highest risk for heart failure. The Intervention Subcommittee will provide guidelines on the standard of care for both treatment arms, as well as lifestyle choices, such as exercise, limiting salt, smoking cessation and medical management strategies. An additional charge for this subcommittee is to monitor the safety of the interventions and to make recommendations regarding any possible changes to the protocol and MOP for patient safety reasons. This subcommittee will likely have working groups such as a Medications Working Group and Lifestyle/Background Working Group to provide plans for standard of care.

Measurements, Procedures and Quality Control Subcommittee (MPQC): This subcommittee is charged with developing and implementing the quality assurance and control mechanisms for the study. The MPQC Subcommittee will work with the Central Lab in developing procedures for biological sample collection, processing, shipping, storage, and analysis – as well as a blood drawing and aliquoting scheme to reflect the storage of specimens for future use. This subcommittee will work with the ECG Reading Center to develop quality control procedures to ensure high quality data. Initially, this subcommittee will establish criteria under which the study will be expected to perform. This subcommittee will require communication with the CC in overseeing the quality assurance procedures, such as the standardized collection of data at all CCNs and clinical sites. They will monitor all quality control as well, and will work closely with the CC in producing quality control reports. The CC will provide the necessary information to the subcommittee, such as data entry quality control and missing data reports. If quality control is an issue based on site visits reports, the MPQC Subcommittee will be alerted and requested to provide recommendations to the Steering Committee, as all site visit reports are reviewed by this subcommittee to determine if any action is warranted. This subcommittee will develop site visit protocols and CCN "report cards." Clear definitions of the boundaries for the CC and CCN monitoring responsibilities will be drafted.

Mortality and Morbidity Subcommittee (M&M): This subcommittee will initially be responsible for developing event definitions and classifications and coding guidelines, then subsequent adjudication procedures. The M&M Subcommittee will be responsible for establishing the guidelines for cause of death; diagnosis of MI, stroke, and heart failure; and evaluating other cardiac events and the trial endpoints. They will jointly monitor all classifications of events, oversee the data collection of events, including forms design, and will serve as the liaison between the CCNs, clinical sites and the CC for the events ascertainment data collection. This subcommittee will require expertise in neurology, nephrology, and cardiology. The M&M subcommittee will function as an adjudication subcommittee once the trial gets underway.

Presentations and Publications Subcommittee (P&P): This subcommittee is charged with developing procedures for review and approval by the SC, and will review all publications, presentations, abstracts, and slides of the SPRINT trial and substudy results. The CC and this subcommittee will develop procedures to track the development of publications and presentations (P&P), as well as strategies for stimulating P&P productivity. Additionally, the CC will provide analyses for publications and presentations, and the study web site will provide P&P tracking reports and study presentations and publications.

Project Coordinators/Operations Subcommittee: This subcommittee facilitates communication and collaboration among clinical sites, the CCNs, and the Coordinating Center. It focuses on recruitment, retention, adherence, and implementation issues, identifying problems early to promptly implement solutions. In addition, the Operations subcommittee addresses specific CCN and clinic requests for tracking and scheduling reports, missed appointment reports, data entry updates or issues requiring attention, and coordinates certification updates and numerous data management issues. This subcommittee will include representatives from the CC (e.g., project managers) and from the MRI and ECG Reading Centers, Central Laboratory and Drug Distribution Center. The CCN Coordinator Chair of this committee can be rotated annually as needed and will serve as a member of the Executive Committee.

Recruitment, Retention and Adherence Subcommittee: This subcommittee will be charged with developing the eligibility criteria, recruitment, retention and adherence to the protocol and procedural strategies. Generation of the SPRINT template informed consent and HIPAA authorizations will be done in conjunction with other subcommittees, such as PC/Operations, MPQC, and Intervention subcommittees. Recruitment and retention strategies will be developed with special emphasis on issues pertinent to recruitment of ethnic groups, women, those with CKD and the elderly. The subcommittee will develop educational and recruitment materials and will provide the culture-specific central training in recruitment strategies. During the follow-up phase, this subcommittee will monitor all aspects of retention, including visit and procedure adherence, and will provide input on necessary retention tracking reports. This subcommittee will collaborate with the Intervention subcommittee to develop strategies and tactics to enhance and monitor intervention adherence. This subcommittee also will assist the Coordinating Center in monitoring recruitment at the CCNs and clinical sites in order to identify recruitment difficulties.

Safety Subcommittee: This subcommittee is charged with responding to concerns about the safety of study participants that may arise during the course of the SPRINT study. Concerns related to safety of study intervention, study medication or study procedures will be reviewed by the committee and either by addressed directly or referred to another subcommittee/ working group as appropriate. Additionally, this committee will help triage issues raised by clinic IRBs that are related to safety and review any clinical practice issues that may arise. They may also review summaries of study data related to the overall safety of study participation, but not

reported by treatment assignment, and develop related reports for or respond to concerns from the Data and Safety Monitoring Board. The Safety Committee will include the Safety Officer, representatives from the Intervention Committee, the CKD working group, the MIND Committee, the Geriatrics working group, and may be joined by other experts for specific issues as needed.

SPRINT-MIND Subcommittee: This subcommittee will provide the scientific leadership for SPRINT-MIND and will include cognitive functioning, dementia and MRI representatives from the CC, CCNs, the NIH (NINDS, NHLBI, NIDDK, and NIA) and the site PI of the MRI Reading Center. This subcommittee will monitor all 3 areas of MIND: dementia, cognitive functioning and MRI scans, as well as selection of the data collection instruments and training of clinical staff. The SPRINT-MIND Subcommittee will serve as the adjudicators for cognition outcomes as members of the M&M subcommittee. This subcommittee may utilize working groups as needed, such as MIND Operations or MIND Geriatrics Working Group.

APPENDIX 6
Participating Sites

SPRINT CLINICAL CENTER NETWORKS

Ohio/Case Western Reserve CCN
Network Hub: Case Western Reserve (PI: Jackson Wright, MD)
Bolwell Suite 2200
11100 Euclid Ave
Cleveland, OH 44106-6053

Southeast CCN
Network Hub: Wake Forest University Health Sciences (PI: Michael Rocco, MD)
Wake Forest University Health Sciences
Section on Nephrology
Medical Center Blvd
Winston-Salem, NC 27157-1063

University of Alabama – Birmingham CCN
Network Hub: University of Alabama, Birmingham (PI: Suzanne Oparil, MD)
703 19th St South
ZRB 1034
Birmingham, AL 35294

Utah CCN
Network Hub: University of Utah (PI: Alfred Cheung, MD)
Dialysis Program/University of Utah
Ezekiel R & Edna Dunke Bldg
84 N Medical Dr East, Room 201
Salt Lake City, UT 84108

Veteran's Administration (VA) CCN
Network Hub: Memphis, TN (PI: Bill Cushman, MD)
Hypertension and Lipids Research
111Q/1030 Jefferson Ave
Memphis, TN 38104-2193

SPRINT COORDINATING CENTER
(PI: David M Reboussin, PhD)
Wake Forest University Health Sciences
Division of Public Health Sciences
Department of Biostatistical Sciences
Medical Center Blvd, Wells Fargo-21
Winston-Salem, NC 27157

SPRINT CENTRAL RESOURCE CENTERS
Drug Distribution Center (PI: Mike Sather, Rob Ringer)

VA Cooperative Studies Program
Clinical Research Pharmacy Coordinating Center
2401 Centre Ave SE
Albuquerque, NM 87106

ECG Reading Center (PI: Elsayed Soliman)
EPICARE
Wake Forest University Health Sciences
Medical Center Blvd, Wells Fargo-13
Winston-Salem, NC 27157

MRI Reading Center (PI: R. Nick Bryan)
Brain Magnetic Resonance Imaging Reading Center
University of Pennsylvania
Section of Biomedical Image Analysis
3400 Spruce St
Philadelphia, PA 19104

Central Lab (PI: Tony Killeen)
University of Minnesota Collaborative Studies Clinical Lab
420 Delaware St SE
Minneapolis, MN 55455

FEDERAL SPONSORS

National Institutes of Health (NIH)
- National Heart, Lung, and Blood Institute (NHLBI)
- National Institute of Neurological Disorders and Stroke (NINDS)
- National Institute on Aging (NIA)
- National Institute of Diabetes and Digestive and Kidney Diseases (NIDDK)

Chapter 2. Recruitment and Informed Consent

2.1 Best Practices for Recruitment

The most successful clinics from a recruitment perspective adopt a very organized approach to recruitment that includes planning, developing and implementing multiple strategies in an organized manner. It is typically necessary to pursue 2 or 3 strategies simultaneously in order to be highly successful with recruitment. Success in simultaneous implementation of multiple strategies requires dedicated effort, organization, coordination and persistence.
Recruitment strategies include:

1. Many clinics will have access to a patient database that can be searched via a computerized search. Develop this resource early, including any relationships with information services personnel that will be required. Assure that you will have the ability to screen for basic eligibility criteria and to obtain contact information for mailings and telephone contacts. Clinics that do not have electronic medical records may have electronic billing databases that can be used to identify patients who have had encounters for hypertension (but not diabetes).

2. Mailings can be useful for contacting patients identified through the electronic database queries. A recruitment brochure or postcard with a toll free number and/or website can help engage potential participants. Similar mailings can be sent to residents in your clinic's catchment area by purchasing mailing lists or contracting with local organizations that provide this service. For example, in Winston-Salem, NC, the local newspaper provides this service, and we have used their services with good results in previous studies. Development of the brochure can be a central task, but if a local or regional phone number is needed, local adaption is required. This can be done using adhesive labels, or via local printing of centrally developed and locally modified brochures.

3. Brochures can also be useful for placement in waiting rooms of potential referring physicians' practices and in pharmacies as possible prescription bag stuffers for patients picking up prescriptions for blood pressure medications. Recruitment information can also be added to appt. reminder cards/letters. These relationships should be developed early.

4. Similarly, wall posters can be useful for display in physicians' offices and other places. In comparison with brochures, posters have the relative disadvantage of not being placed in a potential participant's hands. Since the potential participant cannot take the poster home, they must write the contact information down. This step is a potential barrier. Posters can be developed centrally, but must be adapted locally; if a local phone number is to be used (tear-off labels w/local contact information may be affixed to posters, where possible).

5. Advertisements can be useful, especially in newspapers, given the age range of our target population. In some communities, radio ads may be particularly useful. In our experience, billboards, bus ads and television have been less helpful than newspapers. Newspaper ad copy can be developed centrally and modified locally to include appropriate phone numbers. A phone service can reduce the need for local adaptation. (Advertisements in local church or senior center bulletins/newsletters are other potential

areas to pursue). A telephone script has been developed for the clinics to use as a brief screening tool to identify potential participants.

6. A central website that can be linked to other sites that people visit to find clinical research opportunities can be very helpful. The site can include simple screening questions and a look-up function for a nearby clinic, if any. The website can include a “contact us” function for requesting more information and submitting potential screenees contact and screening information. The site should also include telephone numbers for information, telephone screening, and/or scheduling of a screening visit.

7. A central phone bank can reduce the burden on busy clinic coordinators who may otherwise need to play telephone tag with potential participants between other pressing activities. In previous studies, we have used a central phone bank to receive calls and promptly return calls to conduct telephone screening. After initial telephone screening, tentatively eligible persons can be referred to local clinics for in-persons visits, so local coordinator time can be devoted to in-clinic screening of a higher yield population. The typical salary of a telephone data collector is lower than that of a study coordinator, so the budget is usually favorably affected. The telephone screening instrument can be developed centrally.

8. Public presentations can be useful. High blood pressure is quite common; hence, presentations to community groups can be quite useful. In addition, presentations to health professional groups can help develop referral sources. Slides were developed centrally to support lay and health professional presentations. For lay group presentations, simple screenings can be conducted when possible and allowable. Brochures are excellent supporting materials in these venues.

Organizational approaches
It is important for the recruitment team to develop a schedule of activities such that the team members know what events will be conducted each day during the recruitment period. It is advised that each clinic have at least 3 active strategies ongoing in any given month. Clinics that become overly reliant on a single method can see recruitment slow rapidly if that approach begins to falter. Development and implementation of multiple strategies leads to a greater likelihood of success. Some successful clinics have found it useful to have a recruitment calendar on which they write the recruitment oriented activities planned for each day, week, and month. By doing so, it is easy to see the planned and previously conducted activities and avoid overloading a specific day, week, or month with activities representing a single strategy.

Oversight
The clinic PI and coordinator should meet on an every week (or two) basis during recruitment to review the past week’s and month’s (and recruitment period to date) recruitment productivity (e.g., telephone screenings conducted, clinic screenings conducted, number of potential eligible, number randomized), the next week’s schedule for screening and randomization visits, and the next month’s schedule of recruitment activities (days on which newspapers ads are planned, presentations are planned, practices or pharmacies will be visited, mailings will be disseminated, etc.) We encourage each CCN to support clinic recruitment activities and to assess CCN and clinic recruitment performance during monthly CCN calls or meetings. Clinics should share successes and challenges.

2.2 General Recruitment and Regulatory Guidelines

Participant Screening within the Clinical Practice

Each SPRINT site should consult their local IRB regarding prior approvals to access internal medical record searches for potential SPRINT patients. SPRINT sites should work with the respective networks to complete HIPAA Privacy rule documents, preparatory to research waivers and training prior to patient medical record searches. Once the local regulatory requirements have been approved, investigator-based clinics can be reviewed using the SPRINT inclusion/exclusion criteria to identify potential study patients in their own practice. The investigator can contact the potential patient and ask about their interest by providing study information such as approved recruitment materials to them in person or by other means such as mail or email access.

Depending on the institution, it may also be necessary to request approval from other physicians in the clinical practice to request a consultation for a screening referral to the SPRINT clinic. The patient can then be contacted via an approved IRB screening letter or from the referring physician to discuss their interest in SPRINT. Informed consent procedures must be initiated first and obtained prior to performing any procedures related to the trial (see section below).

Large scale data base searches may also yield an accurate assessment of the study population using the basic inclusion criteria of the ICD-9 code for a hypertension diagnosis, no diagnosis of hypertension but have clinic readings of SBP $\geq$ 130 mm Hg, no diabetes mellitus, stroke or polycystic kidney disease. Other study parameters should include a breakdown by qualifying age, race, gender and the sub-set of potential patients with Stage 3 CKD. Depending on the institution, prior approvals may be needed to obtain de-identified patient information and are known commonly as data transfer agreements. Investigators usually must sign a privacy agreement to protect the patient's private health information (PHI) as part of this process with the local IRB in coordination with the institution's designated privacy, security and compliance services. Some institutions may also require a HIPAA waiver to review patient records for recruitment and possibly, consent waivers. Review data base searches with your respective institutional review boards and sometimes, it is best to complete these requests during the initial submission.

Individuals Recruited Outside Existing Clinical Site Practices

Individuals identified by any media strategy or who are otherwise identified outside of the practice of the SPRINT clinical site will have to be appropriately screened. Approved study general questionnaires could be completed utilizing the general inclusion/exclusion criteria to self identify potential patients with hypertension, no diabetes mellitus, stroke or PKD. A study brochure and a return post card can be used by potential study participants by mailing back their responses.

Telephone screenings can be useful to clinics for participants who call in or respond by post card. Once basic inclusion and exclusion criteria are ascertained, the participant can be invited to the clinic to review and sign the informed consent and HIPAA document to complete an in-depth screening using the SPRINT Inclusion and Exclusion Form.

Recruitment of Study Participants and Study Recruitment Reports
Patient recruitment is of prime importance to the success of this study. Each SPRINT site will be responsible for identifying and recruiting participants into the study. Once potential participants are identified, the site will collect information to determine eligibility. Potential participants who are eligible for and are interested in the study will be asked to sign an informed consent form and HIPAA document will subsequently be enrolled into the study. The site will track each potential participant from the time s/he is identified until s/he is enrolled or not enrolled. Each site will document and report a summary of recruitment and enrollment progress as provided by the CC with the Recruitment, Retention and Adherence Sub-Committee (RR&A). The CC will provide numerous recruitment reports on the SPRINT web site for RR&A and study leadership to closely monitor recruitment targets.

2.3 SPRINT Recruitment Materials
The SPRINT Recruitment, Retention and Adherence Sub-Committee and other committees have developed recruitment tools to assist sites in their local recruitment efforts. As previously stated, all patient and recruitment materials must receive local regulatory approval prior to their usage. Any recruitment materials that are posted to the SPRINT web site require Coordinating Center (CC) IRB approval prior to posting. The CC also provides most recruitment material in Spanish translations. Below is a chart to help your site organize a multi-strategy recruitment plan. From previous clinical trials, emphasis must be reiterated about the active support of the local investigator, site team with the doctors within your institution. The strategies can help a site organize their recruitment plans that include the doctors, mid-level providers and staff to identify and promote SPRINT.

The purpose of these materials is to provide potential participants with enough information to allow them to determine if they are eligible and interested in the study.

Note: All CC IRB approved recruitment materials, including Spanish translations, are posted on the SPRINT website, located under Documents > SPRINT Study Recruitment Materials. Please refer to the web site for the listing of centrally generated materials that are available.

Recruitment Strategies

**Suggested Sources:**	**Suggested Strategies:**
**1. Clinics**<br><br>• Cardiology<br><br>• Vascular<br><br>• Lipid<br><br>• Blood Pressure<br><br>• Cardiac risk<br><br>• Obesity<br><br>• Geriatrics	**1. Direct contact**<br><br>Approach patients or doctors in the wards and clinics. Display posters and pamphlets in the clinics and wards.<br><br>**2. Clinic chart reviews**<br><br>Performing a thorough clinic chart review can help to identify potential participants. Once potential participants are identified, a letter can be sent describing the study to the participant.<br><br>If the participant agrees to come in for a screening visit, send a letter describing what the visit will entail, as well as what they can expect to happen once randomized into the study.

• Peripheral Arterial Disease	□ Once a participant is randomized, a letter describing the participant's participation in the study should be sent to their family physician and/or specialist.
• Renal disease	**3. Physician referrals**
**2. General and family medicine clinics**	□ Approach the various doctors. Request that any patients with the inclusion criteria be referred to the SPRINT Study for a screening visit. Also, sites may provide the doctor with a pocket eligibility card to assist with identifying potential participants from his/her practice.
	□ An invitation letter requesting physician referrals can also be sent to doctors.
	**4. Advertisements**
	□ Printed media – press release, news stories, advertisements in your local paper or community magazine, bus posters that advertise non-profit groups; mail inserts through the local community newspapers.
	□ Religious facilities in their church bulletins, health clinics, senior centers, doctors' offices
**3. General Public**	□ Electronic media – advertise on radio or your hospital's website, public service announcements
	□ "Tag lines" on medical institutions' phones lines, e.g., "Wake Forest has a blood pressure study called SPRINT. Call XXXXXXXX for more information."
	**5. Educational Events**<br>□ Public forums – slide presentations for these events are available on the website. Also display posters and provide pamphlets.<br>Medical rounds –<br>Both professional and lay audience slide presentations for these events are available on the website.

To provide potential participants with a better understanding of the study, the following topics will be explained in one or more of the introductory materials (letters, fact sheets, etc):

- Purpose of the study;
- Voluntary nature of any response;
- Randomization;
- Extent of confidentiality of information;
- Time period for maintenance of records;
- Disposal of records; and
- Assurances regarding continued care for non-responders.

The recruitment materials should be used as they are presented. Any modifications to recruitment materials, as well as additional recruitment materials that the site may develop,

must be approved by their local regulatory institutions in advance of their use in the study.

2.4 Informed consent.

Obtaining Consent from Participants

Informed consent is not just a form. Rather, it is a process that involves the following steps:

- Giving a participant adequate information about the study;
- Providing adequate opportunity for the participant to consider all options;
- Responding to the participant's questions;
- Ensuring the participant has comprehended the information;
- Obtaining the participant's voluntary agreement to enter the study;
- Continuing to provide information as the participant or situation requires.

In order to be effective, the process should provide ample opportunity for the investigator and the participant to exchange information and ask questions. Participants should be fully informed about the study and have adequate time to evaluate the pros and cons of participation. Participants should be encouraged to discuss the study with anyone they wish, particularly family and friends who might be affected (for example, persons who might be needed to provide transportation).

Close associates of the participant may raise questions and considerations that the participant has overlooked, and questions that concern the family are better answered sooner than later. Furthermore, there is evidence to suggest that family support for studies of this kind increase the probability of participant cooperation and compliance during the course of the research.

The setting in which consent is obtained should be as private as possible so participants can freely ask questions without embarrassment.

Below are some frequently asked questions about the consent process.

- **Who can obtain consent from potential participants?**

FDA does not specify who can obtain consent from a potential participant. Some sponsors and IRBs require the clinical investigator to conduct the consent interview. Regardless, the person who conducts the consent interview should be knowledgeable about the study and able to answer questions. If someone other than the investigator obtains consent, the clinical investigator should formally delegate this responsibility and the person so delegated should have the appropriate training to perform this activity.

- **21 Code of Federal Regulations (CFR) 50.27(a) requires that a copy of the consent document be given to the person signing the form. Does this have to be a photocopy of the form with the participant's signature affixed?**

No. The regulation does not require the copy of the form given to the participant to be a copy of the document with the participant's signature, although this is <u>strongly</u> encouraged. It must, however, be a copy of the IRB approved document that was given to the participant to obtain their consent.

- **Do you have to have a witness to the consent process?**

An impartial witness is only required if the participant cannot read, if the participant is incapable of understanding the consent document, or if the participant does not speak English. Otherwise, a witness is not required.

- **When a witness is required, must they observe the entire consent interview or only the signature of the participant?**

When a witness is required, they must be present throughout the entire consent interview. The intended purpose is to have the witness attest to the presentation and the apparent understanding of the participant, not just the validity of the participant's signature.

- **How do you obtain informed consent from someone who speaks and understands English but cannot read?**

Illiterate persons who understand English may have the consent read to them and "make their mark," if appropriate under applicable state law. Federal regulations do permit the use of a short form for patients that cannot read. A short form is a document that states that the elements of informed consent as required by the Code of Federal Regulations have been presented orally to the participant. When this method is used, there must be an impartial witness to the oral presentation. Also, the IRB should approve a written summary of what is to be said to the participant. The participant must sign the short form. However, the witness must sign both the short form and a copy of the IRB approved summary. A copy of the summary and short form must be given to the participant. If you encounter an illiterate participant, consult with your IRB Chair to discuss your local guidelines.

- **How do you obtain consent from a person that does not speak English?**

The SPRINT Informed Consent documents are translated into Spanish for Spanish speaking participants. Clinical sites that recruit and enroll Spanish speaking participants should have Spanish speaking staff or interpreter available to answer questions. The Office for Human Research Protections (OHRP) strongly encourages the use of this procedure whenever possible. Department of Health and Human Services regulations for the protection of human research participants require that informed consent information be presented in language understandable to the participant and, in most situations, that informed consent be documented in writing.

Where informed consent is documented in accordance with federal regulations, the written consent document should embody, in language understandable to the participant, all the elements necessary for legally effective informed consent. Participants who do not speak English should be presented with a consent document written in a language understandable to them.

Human research subjects are protected through informed consent procedures and the HIPAA document. The signing of these documents is a criterion for eligibility to participate in SPRINT. Each site investigator will ensure that the participant understands the purpose of the study, risks and rights, study procedures and visit schedules. The PI is ultimately responsible for the proper oversight of these procedures prior to enrolling anyone in SPRINT.

The informed consent addresses the following important points:

- Each participant must be fully informed of all study procedures and requirements in order to be considered a "knowing" participant; and
- Participation is voluntary and all information provided by participants will be kept confidential.
- The site investigator or his/her designee will be fully responsible for obtaining written consent from each participant.

- Sites recruiting individuals who have very little or no knowledge of the English language must submit a translated consent in the respective language for IRB review and approval.
- In the development of the informed consent forms, each site will use the templates provided by the CC as a guide. The prototype consent forms and accompanying cover letters are found in the following Appendices: All information in the essential elements for the informed consent template must be included in the site consent forms. Additional information may be added based on individual IRB requirements, but information in the prototypes may not be excluded from the forms. Any changes made by the IRB will be submitted to the network and the CC for review.
- The participant will be given a copy of the consent form after it is signed. The original will be kept in the participant's file at the site in a secure and locked specified location.
- Administration of the consent form involves providing the participant with background information about the study and its requirements.
- The implications of randomization and the necessity for completing the required procedures should be emphasized to each potential participant.
- When the consent form is provided to the potential participant, s/he must be offered sufficient time to carefully read the document and must be given sufficient opportunity to have all questions regarding the study answered before s/he is asked to make a decision regarding participation. If requested, s/he can also take the consent home to review with family members or significant others prior to signing.

Other Tips

- Use of the first person (e.g., "I understand that ...") can be interpreted as suggestive, may be relied upon as a substitute for sufficient factual information, and can constitute coercive influence over a participant. Use of scientific jargon and legalese is not appropriate. Think of consent as a teaching tool not as a legal instrument.
- Anyone who signs a consent form should personally date it.
- If consent is obtained the same day that the participant's involvement in the study begins, the participant's medical records should document that consent was obtained prior to participation in the research. The following statement could be included in the records: "All the required elements of informed consent were presented to the patient. Voluntary consent was obtained and the patient's questions were answered prior to initiation of any research procedures."
- A copy of the consent document must be provided to the participant and the original signed consent should be retained in the study records.

Chapter 3. Measurements and Administering Questionnaires

This chapter provides instructions for the accurate and reproducible measurement of height, weight, seated blood pressure, standing blood pressure, pulse, and four meter walk. Additionally, instructions for administering SPRINT study questionnaires are also included.

3.1 Height, Weight, and Physical Exam

3.1.1 Background and Rationale

Accurate height and weight measures will be used to calculate body mass index (BMI) as an estimate of total body fat independent of height. Guidelines are available for the determination of overweight and obesity based on BMI values. BMI correlates well with adipose tissue composition measured by more burdensome procedures such as CT scan, underwater weighing and bioelectrical impedance. The purpose of the physical exam is to allow ascertainment of physical findings needed for appropriate and safe management of hypertension in SPRINT participants.

3.1.2 Height

<u>Frequency</u>

Height is obtained only at the baseline examination.

<u>Equipment</u>

1. Steel tape measure, marked in inches, hung vertically on the wall with the tape at a right angle to the floor and installed accurately to zero at the baseboard. A floor and back board unit attached together is recommended. Commercial stadiometers are also acceptable.
2. Headboard – a right triangle with an angle brace.

<u>Procedure</u>

Figure 1 provides an illustration of the correct procedure for assessing height, as discussed below in further detail.

1. Bare feet are preferred. Nylons or thin socks are acceptable. Thick socks must be removed.
2. The study participant should back up to the wall until their heels, buttocks, and/or shoulder blades touch the board (tape), with their eyes straight ahead. The subject's head should be in the Frankfort (horizontal) plane. Feet should be together with ankles touching or as close as possible.
3. Place the headboard over the crown of the head with the headboard forming a right angle with the tape measure. The headboard should touch the scalp lightly. Have the subject take a full inspiration.
4. Ask the subject to step out from under the headboard.
5. Read the height to the nearest 0.5 inch.

Special circumstances

1. For frail or unsteady participants, height should be taken by allowing them to be lightly steadied.
2. Participants who are unable to stand for measurement (e.g., wheelchair-bound subjects) should be coded on the form as indicated.
3. Height may be assessed in participants with conditions such as kyphosis. However, these conditions should be coded on the form as indicated.

Figure 1. Procedure for Assessing Height.

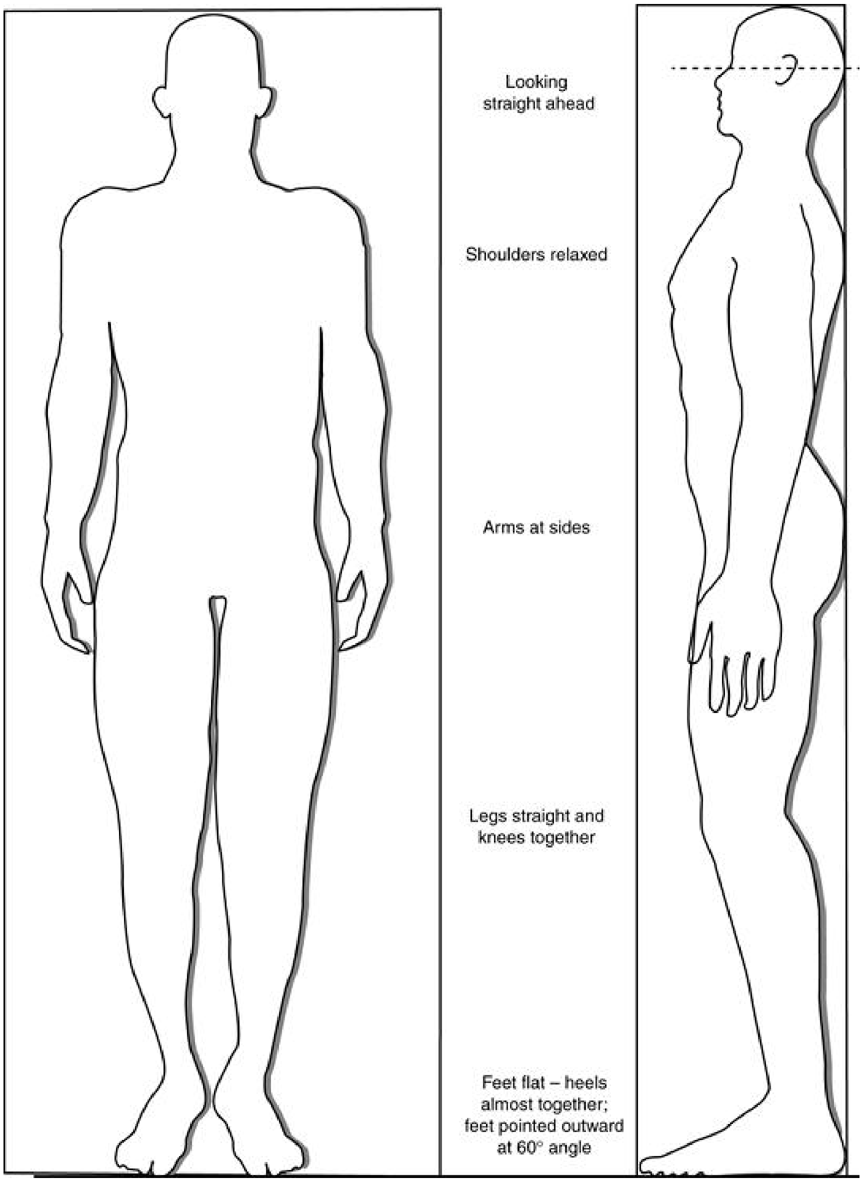

3.1.3 Weight

Frequency

Weight is obtained at the baseline examination and annually.

Equipment

High-quality scales that are currently used in clinical practice (clinical staff should ensure that the scales used for this study are in good working order).

Procedure

1. Scales should be placed on a firm, flat surface.
2. Perform necessary calibration based on the specifications of the scale being used.
3. Confirm that the scale is balanced (i.e., set on zero without anything on the scales). Balance scales if necessary.
4. Subjects should wear as little clothing as possible, removing shoes, outerwear, items in pockets, etc.
5. Have subject stand on scales with weight distributed equally on both feet.
6. Record weight to nearest 0.5 pound

Special circumstances

1. Subjects with prosthetic limbs and breast prosthesis should be weighed with prosthesis in place.
2. For frail or unsteady subjects, weight should be taken by allowing subjects to be lightly steadied.
3. Subjects who are unable to stand for measurement (e.g., wheelchair-bound subjects) should be coded on the form as indicated.
4. For subjects weighing over the maximum of the scale, an attempt should be made to obtain a weight on a different scale that has a higher upper limit. If a true weight measurement is not obtainable, record the upper limit of the scale.

3.1.4 Physical Exam

The purpose of the physical exam is to allow ascertainment of physical findings needed for appropriate and safe management of hypertension in SPRINT participants. The exam can be performed by the clinical site PI or a designee. The study protocol does not mandate specific data elements related to physical exam findings that need to be assessed; based on their clinical judgment, clinicians at the site can decide which components of a physical exam are most relevant for a given participant. Physical exam findings should be documented in the source documents at the site. However, physical exam findings do not need to be entered into the study data management system.

3.2 Blood Pressure/Pulse

3.2.1 Background and Rationale

Standardized and accurate measurement of blood pressure is critical to the conduct of SPRINT. A standard automated blood pressure measurement device (the OMRON

HEM-907 XL Professional Digital Blood Pressure Monitor) and a specific protocol for the measurement of blood pressure and pulse will be utilized.

3.2.2 Frequency and Timing

Seated blood pressure and pulse are measured three times at each clinic visit. The average of these three measurements will constitute the visit blood pressure and pulse.

A single standing blood pressure and pulse measurement will be made after the seated blood pressure measurements at the following visits: screening, baseline, 1 month, 6 months, 12 months, and annually thereafter.

Blood pressure measurement must be conducted early in the visit and not following potentially stressful exam components such as the blood drawing or self-administered questionnaires.

3.2.3 Methods

This protocol is written for use with the OMRON HEM-907 XL Professional Digital Blood Pressure Monitor. Special attention must be placed on assessment and maintenance of the instrument's accuracy as per the manual that accompanies the instrument.

The design and operation of the OMRON HEM-907 XL Professional Digital Blood Pressure Monitor are based upon the combined principles of compression of the brachial artery under an elastic, inflatable cuff and estimation of the systolic and diastolic blood pressure levels by oscillometric methods. The observer places the correct size cuff on the participant's arm, pushes the button on the device and waits for the output.

All readings will be recorded to the nearest digit.

Required Equipment

- OMRON HEM-907 XL Professional Digital Blood Pressure Monitor.
- BP cuff/bladder sets in four sizes:
    - Small: < 22 cm (7 to 9")
    - Medium: ≥22 to <32 cm (9 to 13")
    - Large: ≥32 to <42 cm (13 to 17")
    - Extra Large: ≥42 to ≤50 cm (17 to 20”)
- Metric tape
- Black pen
- Preferably, chair with arm support for blood pressure measurement, or chair and table. Table must provide for a comfortable resting posture of the arm with mid-cuff at heart level. Chair must have a back for participant's back to be supported during rest and BP determinations.

- Data collection form

<u>Cuff Size Determination</u>

Proper cuff size must be used to avoid under- or over-estimation of blood pressure. Cuff size refers to the cuff's bladder, not the cloth.

- BP measurements should usually be taken in the right arm. The left arm may be used if the BP is known to be higher in that arm or in the presence of an anomaly or other circumstance prohibiting use of the right arm.
- Have the participant remove his/her upper garment (bare arm).
- Have the participant stand, holding forearm horizontal (parallel) to the floor.
- Measure arm length from the acromion (bony protuberance at the shoulder) to the olecranon (tip of the elbow), using a metric tape.
- Mark the midpoint on the dorsal surface of the arm.
- Have participant relax arm along the side of the body.
- Draw the tape snugly around the arm at the midpoint mark. NOTE: Keep the tape horizontal. Tape should not indent the skin.
- Use the criteria in the table below for determining cuff size. A copy of the table should be attached to the sphygmomanometer for easy reference.
- Cuff size as determined at screening will be pre-printed on all subsequent blood pressure forms. Unless significant weight loss or gain has occurred between visits, it is preferable to use the same cuff size throughout the study.

Cuff Size Indicated by Measured Arm Circumference	
Arm Circumference	Cuff Size
< 22 cm (7 to 9")	Small
≥22 to <32 cm (9 to 13")	Medium
≥32 to <42 cm (13 to 17")	Large
≥42 to ≤50 cm (17 to 20”)	Extra Large

If the participant's arm circumference is > 50 cm, the OMRON will not be used for BP and pulse measurements. In these participants, a manual (preferably mercury) manometer will need to be used with an extra large or thigh sized cuff.

<u>Programming the OMRON HEM-907 XL Professional Digital Blood Pressure Monitor</u>

The BP monitor should be programmed to allow a 5 minute rest before the first BP measurement, to take 3 readings at 1 minute intervals, and to display the average. See specific instructions provided by OMRON at the end of this chapter.

Wrapping the Blood Pressure Cuff Around the Arm

The participant should be seated with back supported, legs uncrossed, in a quiet room, with the elbow and forearm of the right arm resting comfortably on the armrest of the blood pressure measurement chair (or the table) with the palm of the hand turned upward. If unable to take the BP using the right arm, please note in the source documentation. The area to which the cuff is to be applied must be bare.

Locate the brachial artery by palpation and mark the skin with a little dot. (The brachial artery is usually found at the crease of the arm, under the muscle and slightly towards the body).

Place the appropriate cuff around the upper right arm so that:

a) The midpoint of the length of the bladder lies over the brachial artery, and
b) The mid-height of the cuff is at heart level.

NOTE: Confirm for yourself where the midpoint of the length of the bladder is by folding the bladder in two. Do not trust the marking on the cuff.

Place the lower edge of the cuff, with its tubing connections, ½ to 1 inch above the natural crease across the inner aspect of the elbow.

Wrap the cuff snugly about the arm, with the palm of the participant's hand turned upward. Make sure that the long edges of the cuff lie on top of each other as you wrap the cuff around.

Secure the wrapped cuff firmly by applying pressure to the locking fabric fastener over the area where it is applied to the cuff.

Do not wrap the cuff too tightly around the arm, but so that you can insert only one finger between the cuff and arm.

Taking the Seated Blood Pressure and Pulse Measurements

The participant should sit quietly for a period of 5 minutes before the first blood pressure is taken. He or she should be seated comfortably, feet flat on the floor with their back supported. Ideally, he or she should not have smoked or had any caffeine within the last 30 minutes prior to the BP determinations.

During the 5 minute rest period, participants should be resting and should not be completing questionnaires or speaking with study staff. The staff member should leave the room during this 5 minute rest period. The following script can be used at this time.

SCRIPT: "I would like you to rest for 5 minutes before I begin taking your blood pressure. I will leave the room. When I return, I will not speak to you but will immediately begin to take your blood pressures. Do you understand?" With their agreement, leave the room and return in 5 minutes.

Push the button on the machine and wait for the output. Record the systolic and diastolic blood pressure and pulse readings obtained at each of the three readings and the

average of the three readings in the spaces provided on the appropriate form. [Note: only the averages will be data entered].

Standing Blood Pressure and Pulse Measurements

After the seated BP and pulse measurements have been obtained and recorded, ask the participant to stand. Time for one minute with a watch or stopwatch; you may also use the Omron itself (it can be used as a timer) to take the standing BP. If using the Omron, once his (her) feet touch the ground, press the start button on the Omron device to initiate a new BP and pulse measurement.

After 60 seconds, turn the mode selector to "single" and press the start button on the Omron again to take one standing BP and pulse measurement. Immediately after the standing BP and pulse measurement have been obtained and recorded, turn the power off to the machine.

For standing BP measurement, the arm should be bent slightly and the hand of the cuffed arm supported at heart level (a Mayo stand is acceptable for support).

After the BP and pulse reading, ask the participant "Did you experience dizziness or light headed feelings when standing for this assessment?"

Record the standing systolic and diastolic blood pressure and pulse readings and the answer to the question regarding symptoms on the case report form.

As a note, the Omron device will "clear" itself of all previous readings after a 5-min interval. Moreover, the Omron does not permit downloading readings to another electronic device. Thus, recording the blood pressure and pulse readings in a timely manner is essential.

3.2.3.1 Repeating Automated Measurements

For participants experiencing muscle tremors, abnormal heart rhythms, weak pulse or very low blood pressure, automated blood pressure devices may sometimes give unreliable results or fail to obtain a reading and indicate an error code. Errors or unreliable results may also occur due to problems with connectors, tubing or cuffs (see section 3.3.3 on troubleshooting). Functionally, the OMRON IntelliSense unit is designed to take up to three measurements and average them automatically (in AVE Mode). If the OMRON IntelliSense unit errors and restarts the measurement, the three readings should be taken in the SINGLE Mode and manually averaged. If there is still a problem in obtaining the readings, they should be taken manually with mercury or other properly calibrated manometer.

Occasionally, you may observe large differences between the 3 measurements even in the absence of the circumstances described above. For SPRINT, sets of measurements may be repeated once if the spread between the largest and smallest values of either systolic or diastolic pressures exceed 10 mm Hg. You should NOT repeat measurements when the difference is less than 10 mmHg.

If repeating the measures is needed, the full measurement protocol should be repeated, including the 5 minute rest period and a new set of 3 measurements should be collected.

If a difference between measurements of more than 10 mm Hg is again observed in the second set of measurements, the second set of measurements should still be used for data collection and a note should be recorded in the participant chart so that repeated measurements can be avoided in the future.

3.2.3.2 Proper technique for obtaining seated blood pressure and pulse in participants with arms too large for the Omron (> 50 cm circumference) or in other situations where the Omron cannot be used

In situations where the Omron device cannot be used, the techniques for obtaining seated blood pressure and pulse are described below. However, standing blood pressure should not be collected in situations where the Omron device cannot be used.

Participants with an arm circumference over 50 cm at screening will not be eligible for participation in SPRINT. However, it is possible that participants may gain weight over the course of the study, such that their arm circumference becomes more than 50 cm. An alternative to the Omron device will be necessary in the situation where the arm circumference is >50 cm, since the Omron does not have that size cuff available. In such situations, the steps described below are in the usual order performed when approaching a participant for blood pressure and pulse measurements when the Omron device is not used. In these cases a mercury sphygmomanometer is preferred. If a mercury manometer is unable to be used, another properly calibrated sphygmomanometer may be used.

<u>Cuff Size Determination</u>

See instructions above for determination of proper cuff size.

<u>Applying the BP Cuff</u>

- Place the midpoint of the length of the bladder over the brachial artery and the midheight of the cuff at heart level.
- The lower edge of the cuff should be about 1 inch above the natural crease of the inner aspect of the elbow.
- Wrap the cuff snugly and secure firmly.
- The participant should rest with their palm turned upward.

<u>Preparing to Take the Seated Blood Pressure Measurements</u>

- The participant should be allowed to sit quietly for 5 minutes.
- They should be seated comfortably, feet flat on the floor with their back supported.
- Ideally they should not have smoked or had any caffeine within the last 30 minutes prior to the BP determinations.

<u>Taking the Seated Pulse Measurements</u>

- Pulse measurements are obtained after the participant has rested 5 minutes and before the blood pressure is measured. Palpate the radial pulse for 30 seconds and multiply by 2. The product is recorded as the heart rate.

Determination of Peak Inflation Level

The peak inflation level (pressure) should be determined to assure accurate measurement of the systolic blood pressure. This pressure is determined by:

1) Inflating the BP cuff while palpating the radial pulse and watching the mercury column.
2) When sufficient pressure has been applied, the pulse is no longer felt. When the pulse disappearance is detected, note the level and continue to inflate the cuff another 20 mm Hg.
3) Slowly deflate the cuff while watching the mercury column. Note the level where the pulse reappears, then quickly and completely deflate the cuff.
4) Peak Inflation Level (PIL) = Pulse Obliteration Pressure (POP) + 20 mm Hg.
5) All readings are made at the top of the meniscus. Readings are made to the nearest even digit. Readings that fall exactly between markings should be read to the next marking immediately above.

Taking the Seated Blood Pressure Measurements

Blood Pressure Sounds

- Systolic blood pressure (SBP) is the first of at least two regular tapping sounds heard when deflating the cuff.
- Diastolic blood pressure (DBP) is the level at which the last of the rhythmic sounds are heard.
- A single sound heard in isolation either before the SBP or after the DBP does not meet the BP criteria.

Obtaining the BP Readings

1. Following determination of the peak inflation level or any other BP measurement, wait 60 seconds after complete deflation of the cuff before re-inflating for the next reading.
2. Place the diaphragm of the stethoscope over the brachial artery.
3. Inflate the cuff at a rapid, smooth, continuous rate to the peak inflation level.
4. At a slow and constant rate of 2 mm Hg/second deflate the cuff listening throughout the entire range of deflation to 10 mm Hg below the DBP (last regular sound heard).
5. Quickly and completely deflate the cuff.
6. Record the reading.
7. Wait at least 60 seconds between readings and repeat steps 2-6 two more times.
8. Record the 3 readings in the source note and the average of the 3 on the case report form.

Proficiency

All study personnel responsible for obtaining blood pressure readings must review and be familiar with the blood pressure measurement protocol. Blood pressure techniques will be reviewed periodically by the network project coordinators during site visits.

3.3 Guidelines for Proper Use and Maintenance of Equipment

3.3.1 Omron Calibration

The Omron unit has been validated to remain in calibration for up to 100,000 measurements. The units do not have to be calibrated before their first use.

3.3.2 Error caused by Atrial Fibrillation

Atrial fibrillation (AF) is not necessarily problematic with oscillometric devices. However, the presence of AF suggests that multiple measurements (three) be taken and averaged to provide a more accurate reading. Functionally, the OMRON IntelliSense unit is designed to take up to three measurements and average them automatically (in AVE Mode). An atrial fibrillation, however, could cause the OMRON IntelliSense unit to error and restart the measurement. If this is the case, the three readings should be taken in the SINGLE Mode and manually averaged. If there is still a problem in obtaining the readings, they should be taken manually with mercury or other properly calibrated manometer.

3.3.3 Troubleshooting

Refer to the OMRON HEM-907 IntelliSense Digital Blood Pressure Monitor Manual, given out at the SPRINT Study Training Program for a list of error codes and how to correct them.

If you believe your OMRON device is not operating correctly, you should first review the troubleshooting guide in the instruction manual included at the end of MOP Chapter 3. The most common problems with the device involve leaky connectors, tubes and cuff bladders, and replacements can be ordered directly from OMRON. Note that the approximate life of the battery is two years, and if the interval between chargings becomes short, you may need to replace it.

If you still believe your device is not working properly after reviewing the troubleshooting guide, the instruction manual also describes a process for checking the OMRON against a calibrated mercury manometer. However, due to safety concerns, your site may not have a mercury manometer on hand. In this case, comparisons can also be performed between the two OMRON devices located at each clinical site if mercury manometers are not available. If these checks confirm a problem with the OMRON device, it can be returned to the Coordinating Center for service and repairs. The Coordinating Center has a small number of replacement devices that can be provided to clinics so that there is no interruption in routine clinic flow.

4.3 Four Meter Walk (aka, Gait Speed Test)

Participants who are 75 years old or older at baseline will be asked to complete a timed 4 meter walk, also termed as a “gait speed test” to assess physical function. This will be done at baseline and annually.

<u>Equipment</u>

This component requires a stopwatch and a marked walking course.

Marking the Course

The walking course should be unobstructed and include at least an extra one-half meter on each end. The course should preferably be laid out on a hard surface. A carpet is acceptable if this is the only surface available. Avoid laying the course out over the edge of a rug, a throw rug, or any irregular surface that could cause the participant to trip. Try to find a space that is at least 5 meters long for laying out the 4 meter course. To mark the course, place a 2-3 foot piece of masking tape on the floor. Use a measuring tape to measure 4 meters (13 ft 1.5 inches) from the edge of the tape. Place another piece of masking tape at that mark (Figure). Once a proper course has been marked in your clinic, leave the tape on the floor if possible, to prevent having to repeat the measurement for each study visit.

If this test is being done in a new location (such as in a home visit), then a course must be laid out on the floor. If you are in a home, you can move small furniture with the permission of the owner.

Figure: Correct measurement of the 4 meter course

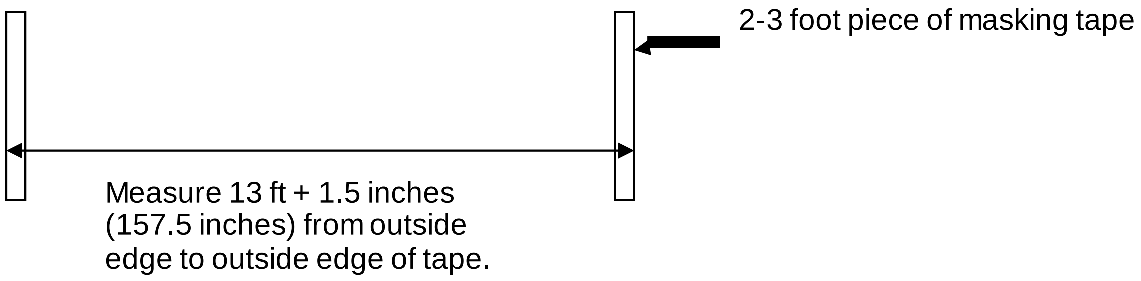

Administering the Test

Staff may refer to the DVD entitled "Assessing Physical Performance in the Older Patient" from the National Institutes on Aging, for complete instructions, as well as a demonstration of the 4-meter walk, which is termed "gait speed test" on the CD.

If possible, women wearing high heels should change into another pair of shoes before performing the gait speed test. Participants are instructed to walk at their usual speed, and timing is stopped when the first foot completely crosses the 4 meter mark. The walk is conducted twice and both times are recorded on the form.

Press the start/stop button to start the stopwatch when the participant steps over the starting line. Wait until the participant actually begins to move before starting the watch. Do not start the watch when you say "begin."

The position of the examiner is critical for the walk. It is recommended that you walk behind and to the side of the participant. However, if you are too close you will set the pace. If you are too far behind you will not be in a good position if the participant falls. You also need to be in a good position to observe the foot crossing the finish line. The best position to maintain during the walk is to the side and slightly behind, outside of the participant's visual field.

Record the time when the participant's first foot crosses the 4 meter line. If the foot lands on the line but doesn't cross it, this is not the end of the test. You need to anticipate when a foot will fully cross the line and be ready to stop the watch as it crosses the line. This is the time to stop the watch. You should envision the foot crossing through a vertical plane extending up from the line. Once the foot has crossed completely through this plane, the watch is stopped.

Record the time to the nearest hundredth of a second (i.e., two decimal places).

The walk is performed two times. After the first time is recorded, the participant is asked to turn around and repeat the test. The second time is then recorded on the form.

<u>Special circumstances</u>

A cane or walker may be used during the walk, but if people with such devices can walk short distances without them, they should be encouraged to do so. Many people with assistive devices use them only when they walk outdoors or for long distances indoors. Doing the test without the device provides a much more accurate assessment of the functional limitations of the participant. Ask the participant if he/she ever walks at home without the device. Then ask the participant if he/she thinks that he/she can walk a short distance for the test. Participants who normally use assistive devices should be watched particularly closely during the test to prevent falling.

<u>Script and demonstration for the 4 meter walk</u>

The following script should be used to introduce and demonstrate the 4 meter walk. As the 4-meter walk is administered using a standardized protocol, adherence to the script is essential.

"Now I am going to observe how you normally walk. If you use a cane or other walking aid and you feel you need it to walk a short distance, then you may use it. This is our walking course. I want you to walk to the other end of the course at your usual speed, just as if you were walking down the street to go to the store".

Demonstrate the walk for the participant.

"Walk all the way past the other end of the tape before you stop. I will walk with you. Do you feel this would be safe?"

Have the participant stand with both feet touching the starting line.

"When I want you to start, I will say: "Ready, begin." *When the participant acknowledges this instruction say:* **"Ready, begin."**

Press the start/stop button to start the stopwatch when the participant begins to move.

Walk behind and to the side of the participant.

Stop timing when one of the participant's feet is completely across the end line.

“Now I want you to turn around and repeat the test. When I want you to start, I will say: "Ready, begin." *When the participant acknowledges this instruction say:* **"Ready, begin."**

Complete the walk test as before.

4.4 General Guidelines for Administering Questionnaires

Below, several established general guidelines for administering the SPRINT questionnaires are discussed. Many of these guidelines have been successfully used in the Action to Control Cardiovascular Disease in Diabetes (ACCORD) study.

Be Familiar with the Study Protocol

It is extremely useful for the study staff to review all forms and the study protocol to refresh the memory regarding the forms and correct procedures before participants come to the assessment area. Indeed, if the clinic will recruit a small number of participants for the instruments that will be administered to subgroups, or if multiple staff members will administer the instruments, this guideline will be particularly important.

Data Collection

Although many questionnaires will be self-administered, the individual who will give the forms packet to participants will play an important role in the process of data collection. A data collector with a friendly, relaxed, and open manner will put the participant at ease, and conveys the message that the collector feels the forms are important to the study. In addition, the data collector should dress in a neat, clean and professional manner, which also conveys that the data collector is an appropriate representative of the research team. Also, should the participant have any questions, the data collector should be available to answer questions. In addition, the data collector or interviewer should be gracious to all participants irrespective of their dress, appearance, style of speech, or preferences that may be different than those of the interviewer.

In addition, along with a positive, professional demeanor, the data collector should refrain from any statements or body language that may suggest an answer for the participant. In general, most of the participant’s questions may be answered by reminding him or her of the directions, or by rereading the statement to the participant. The interviewer should read the statement exactly as it is written, without inserting emphasis on any word or phrase.

The Setting

The clinic staff should be available to greet participants as they arrive. In addition, if the survey area is in an office that is not immediately visible upon entry into the clinic, or if a participant has physical limitations, it will be useful to meet the participant and escort him or her to the area where the surveys will be administered. Ideally, this setting will be comfortable and private, with minimal opportunities for interruptions. Also, this area will ideally be located near a rest room or a break area.

Assessing Ability to Complete Questionnaires

It is possible that some participants will have low literacy skills, which may reduce the quality of the data and cause embarrassment to participants, which may also

affect retention to the study. Cues to poor literacy may include asking many questions or asking the same questions repeatedly, completing the measures very slowly, checking off items quickly, and glancing around and not appearing to focus on the questionnaires. In these cases, the data collector may ask the participant a non-threatening question such as “Many individuals prefer to have the questionnaire read to them. Would you like for me to read these questions to you?” If the answer is yes, the data collector can read the questions. Otherwise, if the participant can complete the questionnaire on his or her own, the data collector will allow the participant to proceed. Below, some guidelines for administering SPRINT instruments via interview are discussed.

Nonpartiality and Confidentiality of the Interviewer. The interviewer should be thoroughly familiar with the questionnaire before interviewing participants. Inexperienced interviewers should practice completing an interview with a mock participant. This will help the interviewer develop a relaxed, non-mechanical style. It is also very important that the interviewer conveys a sense of impartiality. Indeed, as there are no right or wrong answers to the HRQL measures, it may be helpful to remind participants of this fact, which will help put the participant at ease and provide candid answers. In addition, as the questionnaires are introduced, the interviewer should remind participants that their responses will remain confidential, and that the interviewer will not discuss their responses with anyone who is not directly related to the study.

Keep explanations to a minimum. The interviewer should not interpret questions. The interviewer, for example, may define a word, but should not make statements such as “I think they mean…”, which may alter the interpretation of the participant.

Correct Inappropriate Responses. Although the instruments all have fixed response categories, it may be the case that some participants will reply that none of the choices fit their desired response. In this event, the interviewer should suggest that the choice that comes the closest should be selected. If the participant still refuses, the interviewer should note this on the questionnaire.

Comfortable “Pace of Interview”. The interviewer should maintain a comfortable pace for himself or herself and the participant during the interview. For example, reading questions very quickly may convey the unintended message that the process of interviewing is undesirable, while reading questions extremely slowly may be inferred as being condescending. A good practice would be to ask the participant if questions are being read at the correct tempo. Also, after 10 minutes of interviewing, participants should be asked if they desire a brief (5-minute) break.

<u>Examining Completed Questionnaires</u>

Another important role of the data collector or interviewer entails examining the completed surveys. This should be done immediately after the participant has completed the forms and before the participant leaves the clinic setting. If the participant has skipped any questions, and/or filled out the questionnaire

incorrectly, the staff person will ask the participant to complete the questionnaire as the directions indicate. If an item is missing or incomplete, the data collector should ask the participant if he or she would like to answer the question, using a statement as "I've noticed that this item is blank. Would you like to answer this item?" If the participant states that he or she intended to leave the item blank, the data collector should accept the participant's decision without comment. Furthermore, the data collector should note in the margin of the form that the participant intended to leave the item blank. This will allow the item to be coded as 'refused/permanently missing' in the data query edit system.

Expressing Appreciation

Thanking the participant for his or her time, interest and focus in completing the questionnaires is extremely important. In addition, after all questionnaires have been completed, the data collector should escort the participant back to his or her family or to the waiting room.

Storing the Questionnaires

Once a questionnaire has been completed by a participant and edited by the survey administrator, the questionnaire should be stored in a secure place within the clinic. The questionnaire should not be left unattended by staff. Information collected for research purposes can only be shared with other members of the research team and the participants' privacy must be protected at all times. The data collector should never discuss any of the responses with anyone who is not directly involved in the study.

Mailing Self-Administered Forms to the Participant

The self-administered forms can be mailed to the participant's home prior to the visit for completion at home. Thus, the forms can be completed in advance of the clinic visit, but no more than one week prior to the visit. Forms should not be completed AFTER the visit. Forms completed at home should be brought into the clinic at the time of the clinic visit. As with forms completed in the clinic setting, a careful review should be made of each form prior to the participant leaving the clinic visit to assess completeness. In all situations, the participant should be encouraged to complete as much of the form as they are able to, by himself or herself.

	SC	RZ1	RZ2	1M	2M	3M	6M	9M	12M	15M	18M	21	24M	27M	30M	33M	36M	39M	42M	4SM	48M	51	54M	57M	60M	63M	66M	69M	72M	PRN
Annual Medications and Physical Exam History Form									x				x				x				x				x				x	
Baseline Medications and Physical Exam		x																												
BP Management Baseline		x																												
BP Medication Management Log			x	x	x	x	x	x	x	x	x	x	x	x	x	x	x	x	x	x	x	x	x	x	x	x	x	x	x	x
Day Surgery																														x
Dialysis																														x
Drug Dispensing Form			x	x	x	x	x	x	x	x	x	x	x	x	x	x	x	x	x	x	x	x	x	x	x	x	x	x	x	x
ECG		x											x								x									
Encounter and Disposition		x		x	x	x	x	x	x	x	x	x	x	x	x	x	x	x	x	x	x	x	x	x	x	x	x	x	x	x
Event Ascertainment						x	x	x	x	x	x	x	x	x	x	x	x	x	x	x	x	x	x	x	x	x	x	x	x	
Falls Self Efficacy							x		x				x				x				x				x				x	
Inclusion/Exclusion	x																													
Intensive BP Management 1M visit				x																										
Intensive BP Management 2M, 3M, 9M ... and PRN visits					x	x		x		x		x		x		x		x		x		x		x		x		x		x
Intensive BP Management 6M, 12M, 24M ... visits							x		x				x				x				x				x				x	
Intensive BP Management 18M, 30M, 42M ... visits											x				x				x				x				x			
Lab Form Month 1, 3, 18, 30, 42, 54				x		x					x				x				x				x				x			
Lab Form Month 12, 24, 48 (fasting is required)									x				x								x									
Lab Form Month 6, 36, 60							x										x								x				x	
Lab Form Randomization (fasting is required)		X																												
Lab Form Redraw or Missed Visit																														x
Milepost Exemption*							x		x		x		x		x		x		X		x		x		x		x		x	
MIND Extended Battery**			x										x								x									
MIND Functional Assessment Questionnaire**																														x
MIND Screening Battery			x										x								x									
Participant Contact Information		x				x	x	x	x	x	x	x	x	x	x	x	x	x	x	x	x	x	x	x	x	x	x	x	x	
Participant Status Log																														x
Self-Administered Baseline History		x																												
Self-Administered Mens/Womens Health**			x				X		x				x				x				x				x				x	
Self-Administered My Health			x						x				x				x				x				x				x	
Self-Administered Morisky Medication Adherence Scale			x						x												x									
Serious Adverse Events																														x
Standard BP Management 1M, 6M, 12M, 24M ...				x			x		x				x				x				x				x				x	
Standard BP Management 2M, 3M, 9M ... and PRN visits					x	x		x		x	x	x		x	x	x		x	x	x		x	x	x		x	x	x		x

* required only for intensive participants not at goal were no med was added.
** subsets of participants

OMRON®

INSTRUCTION MANUAL

IntelliSense® Blood Pressure Monitor

Intelli sense®

Model HEM-907XL

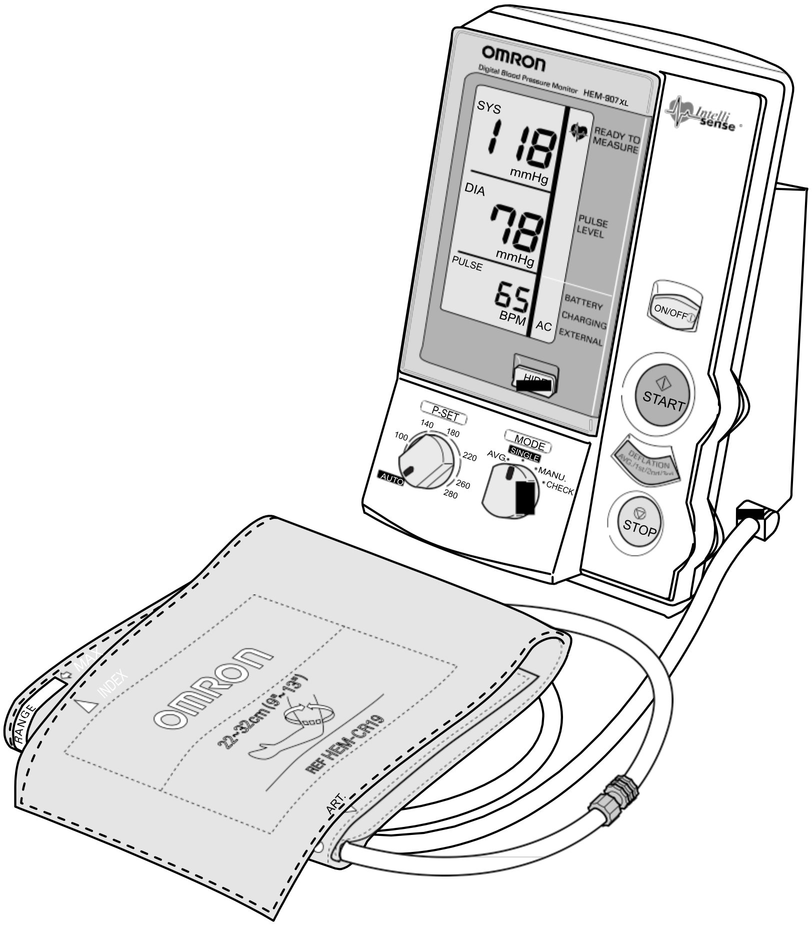

Thank you for purchasing the OMRON IntelliSense® Blood Pressure Monitor.

Please thoroughly read this Instruction Manual before using this monitor to ensure safe and accurate use.

Please keep this manual near the monitor all the time for future reference.

Table of Contents

Be Sure to Read This Section
- Exemptions..2
- Notes on Safety...................................3

Know Your Unit
- Features of the Product 7
- Components of the Product 8
- Features and Functions 9
  - Names of the Parts 9
  - Functions and setting while the unit is in use 11

Preparations before Measurement
- How to Apply the Cuff.......................13
- How to Use the Power Source 15
  - How to Use the AC Adapter 16
  - Installation and Replacement of Battery Pack 17

How to Measure Blood Pressure
- List of Measurement Modes 18
  - SINGLE Mode 19
  - AVG Mode 21
  - MANU Mode 23
  - How to Check Pressure Accuracy..25
- How to Clean the Unit after Use........26
- List of Error Codes 27
- Troubleshooting 28

Specifications
- Caution 29
- Five Year Limited Warranty...............29
- Specifications....................................30

EXEMPTIONS

OMRON does not accept liability and warranty becomes void under the following circumstances:

1. When persons, not authorized by OMRON, perform repairs or modifications of this product.
2. When use and/or operation of this device is adversely effected by a device not manufactured by OMRON.
3. When use and/or operation of this device is adversely effected by use of parts, not authorized by OMRON, to repair or modify this product.
4. When Notes on Safety or Instructions for Use contained in this manual are not followed.
5. When use and/or operation of this device is effected by an act of God, such as fire, earthquake, flood or other natural disasters.

1. The product and contents of this Instruction Manual may be changed without prior notice.
2. We have prepared the contents of this Instruction Manual thoroughly. However, if an inadequate description or error is found, please let us know.
3. Reproducing or copying any or all of this Instruction Manual without OMRON's written consent is prohibited.

NOTES ON SAFETY

- The warning signs and the sample icons shown here are listed to insure safe and accurate use.
- The icons and meanings are as follows.

Warning sign	Contents
⚠Warning	Indicates matters in which death or severe bodily damage may arise as a result of incorrect handling.
⚠Caution	Indicates matters in which bodily harm or material damage* may arise as a result of incorrect handling.

* Material damage refers to a wide range of damage involving your house, household goods, domestic animals, and pets.

Examples of signs	
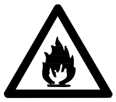	The △ icon indicates caution (including warning and danger).<br>Matters involving actual caution are indicated by text or pictures in or near △.<br>The pictured icon refers to “caution for flammability”.
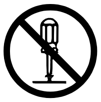	The ⃠ icon indicates prohibitions (what you cannot do).<br>Matters involving actual prohibitions are indicated by text or pictures in or near ⃠.<br>The pictured icon refers to “prohibition to disassemble”.
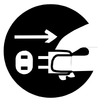	The ● icon indicates something that is compulsory (always follow).<br>Matters involving actual compulsory actions are indicated by text or pictures in or near ●.<br>The pictured icon refers to “unplugging the power source plug”.

Self diagnosis of measured results or treatment is dangerous. Please follow the instruction of the doctor or healthcare provider.<br><br>If cuff inflation does not stop, remove the cuff or pull out the air tube from the main unit.<br><br>If battery fluid gets into your eye or comes in contact with skin, wash the effected area with water repeatedly. Immediately consult a doctor for treatment.	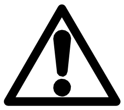
Do not wrap the cuff over an arm to which intravenous injection or transfusion is being conducted, or when otherwise contraindicated.<br><br>Do not connect the air tube or the cuff to other equipment which is connected to an intracorporeal organ. Air embolisms may result.	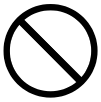
Do not use this unit in the presence of flammable gas or anesthetics or in a high pressure oxygen room or oxygen tent.<br><br>Do not use the battery pack for devices other than for this unit.<br><br>Do not disassemble the battery pack.	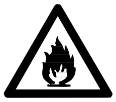
Do not touch the AC adapter with wet hands.	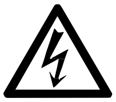

NOTES ON SAFETY

Unplug the AC adapter from the electric outlet if this unit is unused for an extended period of time.<br><br>Unplug the AC adapter from the electric outlet when installing, removing, or cleaning the unit.	
Confirm readings with a stethoscope when an irregular pulse wave is displayed or when the measured value is questionable or erratic.<br><br>Use an AC adapter indicated for use with a power supply of 110 VAC.<br><br>Do not share an electric outlet with other unit or electric appliance.<br><br>After cleaning this unit, dry it well before plugging the AC adapter in the electric outlet.<br><br>If this unit fails to perform as indicated, discontinue use, turn off the unit, unplug the AC adapter from the electric outlet, and contact OMRON's repair department.	
Do not disassemble or modify this unit.	
Do not use any cuff other than the models exclusive for this unit.<br><br>Do not use this unit on infants.<br><br>Do not use this unit on patients using a pump oxygenator.<br><br>Do not use an AC adapter or battery pack not specified for this unit.	

NOTES ON SAFETY

Do not use a cellular phone near this unit.

Do not use this unit in a vehicle.

Do not install the parts and/or instruments not specified for this unit.

Do not use a broken power cord or AC adapter.

Do not install or store this unit where it may come in contact with water or liquid medication.

This is a Class Ⅱ device with double insulation.

General advice

Do not place or put anything on this unit.

Do not drop this unit.

Turn off power to the unit and unplug the AC adapter from the electric outlet before moving the unit.

Read the instruction manual of the other devices to be used at the same time with this unit, to understand and be aware of the interaction between the devices.

When using the unit
- Do not inflate the cuff without being wrapped over the arm.
- Do not use a damaged cuff.
- Be sure that patients do not touch the Buttons of this unit.

After using the unit
- Do not disinfect this unit by autoclave or gas sterilization (EtO, gluteraldehyde, or high concentration ozone).

Do not install or store this unit in the following places.
- Under the direct sunlight,
- Dusty or salty environment,
- Places having slope, vibration, and/or shock,
- Storage of chemicals or where combustable gas may be generated,
- Under high temperature and high humidity.

NOTES ON SAFETY

Maintenance and inspection

1. Check the unit operation on regular basis.
2. If this unit has not been used for more than three months, be sure to check that this unit operates normally and safely before use.

Troubleshooting

If device error 9 (Er9) occurs, take the following procedure promptly:

(1) Remove the cuff from the patient's arm.

(2) Turn off the power of the unit and unplug the AC adapter from the electric outlet.

(3) Display "Out of use" on this unit so that it cannot be used.

(4) Contact Omron Healthcare's Customer Service at 1-877-216-1336.

FEATURES OF THE PRODUCT

OMRON IntelliSense® Blood Pressure unit, Model HEM-907XL is developed to measure blood pressure and pulse rate accurately and simply in a doctor's office, examination room, or patient bedside.

• One-button operation

Simply wrap the cuff and push the START Button. Blood pressure and pulse rate are automatically measured by the oscillometric method.

• Automatic pressure setting

When the P-SET (Pressure Setting) Knob is set to "AUTO", the unit will automatically inflate the cuff to the optimal pressure according to each patient's blood pressure. Pre-setting inflation level is not necessary.

• Noiseless operation

This unit operates so quietly that it can be used in the patient room at night.

• Average Mode (AVG Mode)

In the AVG Mode, this unit will automatically measure for two or three times. The average of systolic and diastolic blood pressures and pulse rate are displayed. Each measurement can also be shown individually. The number of measurements, waiting time before first measurement, and the interval can be changed.

• Auscultation Mode (MANU Mode)

You can measure auscultatory blood pressure by using a stethoscope, with automatic cuff inflation and deflation by this unit. Because the cuff pressures during deflation are displayed digitally and synchronized with the heart beat, they can be read with accuracy. After taking systolic reading you can accelerate cuff deflation to shorten measurement time.

• Large and easy to read display

Large and easy to read figures are displayed on the LCD display.

The IntelliSense® Monitor inflates the cuff to the ideal level with each use. No adjustments are required by the user to select an inflation level. This is especially convenient for hypertensive users and for people with certain arrhythmia or heart disorders, because their blood pressure is likely to fluctuate. The advantage is Personalized Inflation for maximum comfort.

COMPONENTS OF THE PRODUCT

Main unit

IntelliSense® Blood Pressure Monitor, Model HEM-907XL

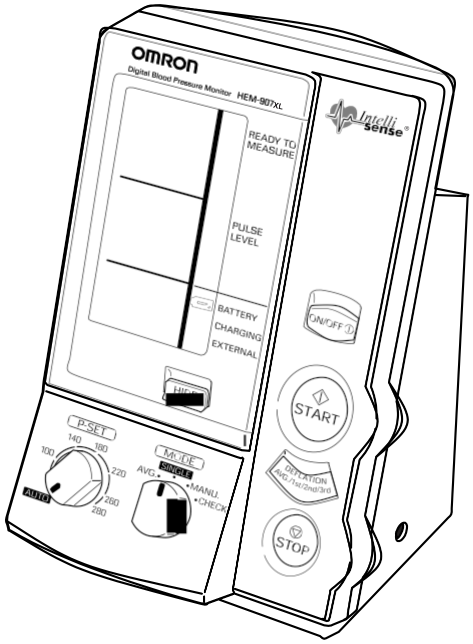

Accessories

(Included and also available separately)

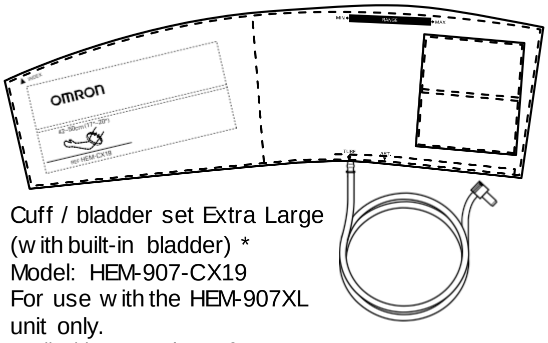

Cuff / bladder set Extra Large (with built-in bladder) *
Model: HEM-907-CX19
For use with the HEM-907XL unit only.
Applicable arm circumference:
17" to 20" (42 to 50 cm)

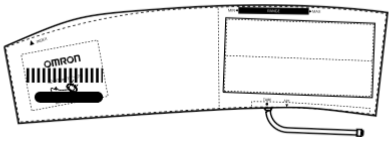

Cuff / bladder set Large (with built-in bladder) *
Model: HEM-907-CL19
Applicable arm circumference:
13" to 17" (32 to 42 cm)

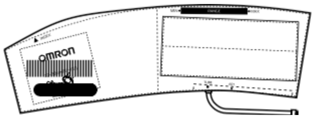

Cuff / bladder set Medium (with built-in bladder) *
Model: HEM-907-CR19
Applicable arm circumference:
9" to 13" (22 to 32 cm)

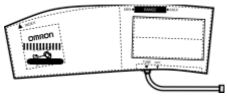

Cuff / bladder set Small (with built-in bladder) *
Model: HEM-907-CS19
Applicable arm circumference:
7" to 9" (17 to 22 cm)

AC adapter

Model: HEM-ADPT907

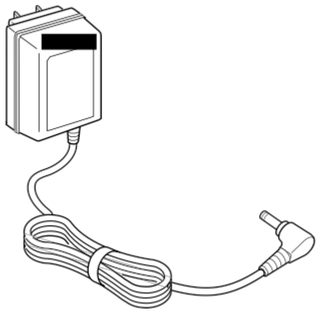

Air tube 1.3m (51 3/16") *

Model: HEM-TUBE-130

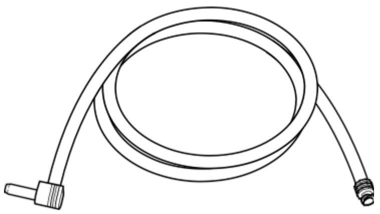

The air tube is available for the large, medium or small cuff size. It is NOT for use with the extra-large cuff size.

Battery pack (48H907N)

Model: HEM-907-PBAT

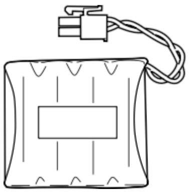

Instruction Manual

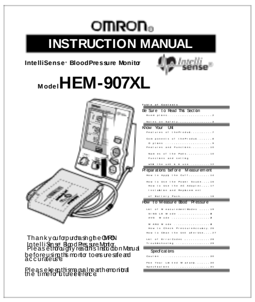

Items identified with an asterisk (*) are consumables and not covered by the guarantee.

FEATURES AND FUNCTIONS

Names of the Parts

Main unit

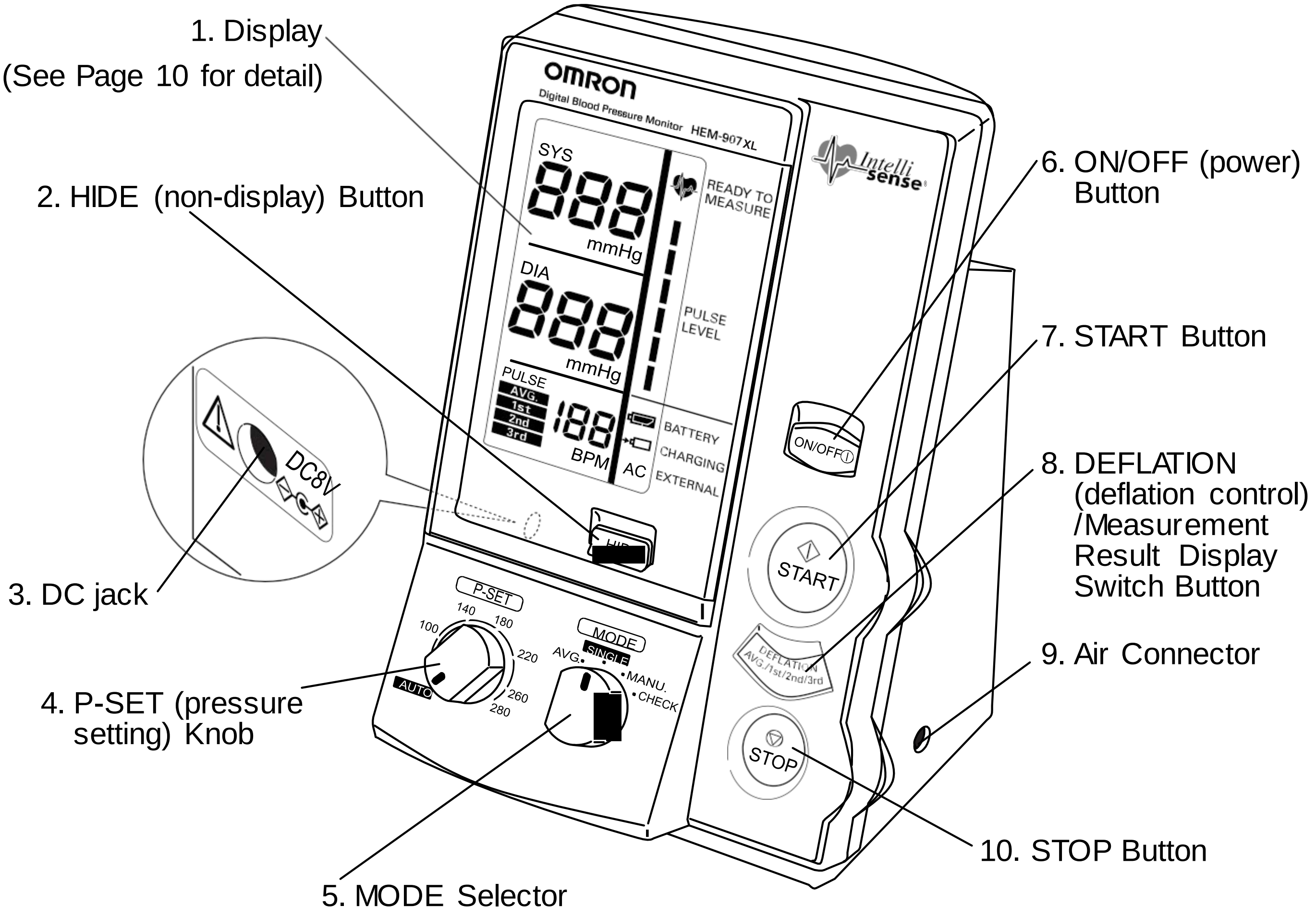

1. **Display:** Displays blood pressure and, pulse rate readings, and oscillation pulse level.
2. **HIDE Button:** Switches display and non-display of measured results.
3. **DC jack:** Connects the AC adapter.
4. **P-SET (pressure setting) Knob:** In the AUTO position, inflation level is automatically set. Otherwise, inflation level can optionally be set manually between 100 and 280 mmHg.
5. **MODE Selector:** Selects the operation mode.
   - One-time Measurement Mode (SINGLE Mode): Measurement with automatic inflation.
   - Average Mode (AVG Mode): Automatically measures two (or three) times consecutively.
   - Auscultation Mode (MANU Mode): Automatic inflation, automatic deflation, and pressure display for auscultation (does not measure blood pressure).
   - Check Mode (CHECK Mode): Checks the accuracy of pressure display. Displays only pressure.
6. **ON/OFF (power) Button:** Turns on or off the unit.
7. **START Button:** Starts the measurement.
8. **DEFLATION (deflation control) /Measurement Result Display Switch Button:**
   - In the MANU Mode, deflates the cuff by approximately 5 to 10 mmHg with each push during deflation.
   - In the AVG Mode, switches the display of average values and the measurement results with each push.
9. **Air Connector:** Connects the air tube.
10. **STOP Button:** Stops the measurement and deflates air rapidly.

FEATURES AND FUNCTIONS

Display

Systolic blood pressure

Ready to Measure /
Pulse synchronization

Diastolic blood pressure

Pulse level /
Number of irregular pulses

Number of irregular pulse waves: The pulse wave having a difference of more than 25% from the average interval of pulse waves is determined irregular. Number of irregular pulse waves is displayed up to three times.

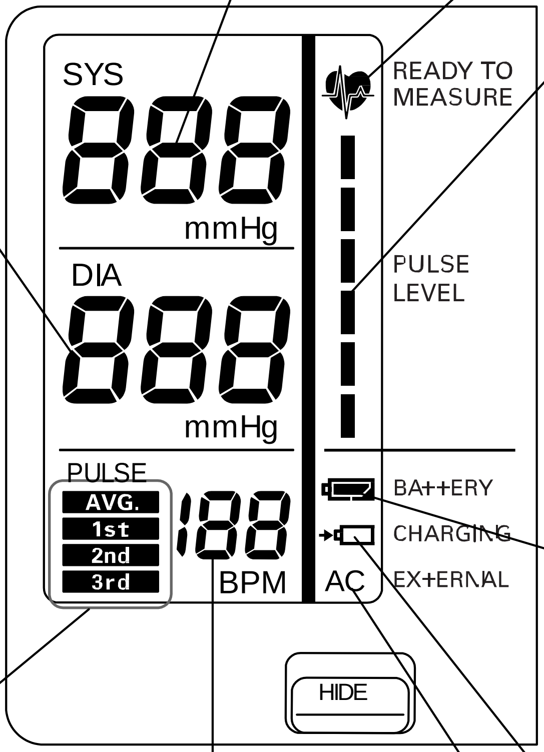

Example of display

No. of irregular pulses

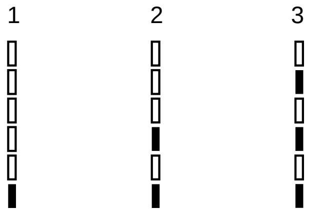

Battery level

Displays [battery icon] for the operable level.

Blinks [battery icon] for a low charged level.

Displays [battery icon] for the inoperable level.

Contents when the AVG Mode is selected

- AVG. : Mean value
- 1st : First reading
- 2nd : Second reading
- 3rd : Third reading

Pulse rate

Charging

Displays [charging icon] when the battery pack is being charged.

External power source

Displays **AC** when the unit is connected to the external power source via the AC adapter.

FEATURES AND FUNCTIONS

Functions setting

(1) Inflation level setting [P-SET]

AUTO (Automatic setting): Can be used when the SINGLE, AVG, or MANU Mode is selected. The unit estimates the systolic blood pressure during inflation and inflates to a proper cuff pressure (approximately 30-40 mmHg above the patient's systolic pressure).

Manual level setting: Inflation level can be set manually between 100 and 280 mmHg. Set the level to 30 to 40 mmHg higher than the expected systolic pressure.

- To set the P-SET to "AUTO", turn the P-SET Knob counterclockwise as far as it goes until you can hear a click.
- In the "AUTO" setting, inflation level may not be set automatically when the systolic blood pressure is more than 220 mmHg. Use the unit in the manual setting.
- If the cuff has not been inflated to the necessary level, it may be re-inflated automatically.

(2) Non-display function [HIDE]

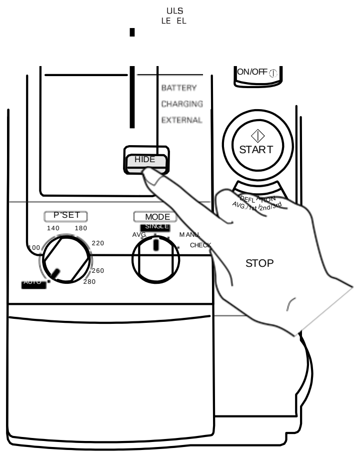

Use to prohibit the display of measurement results. However, the cuff pressure during measurement is displayed.
This function can be used in the SINGLE and AVG Modes.

By pushing the [HIDE] Button, display or non-display of status is switched alternatingly.

Display

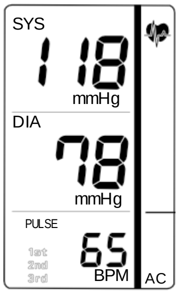

Non-display

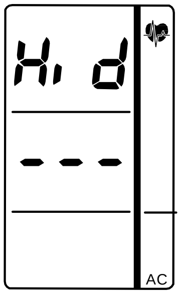

(3) Manual deflation control [DEFLATION AVG./1st/2nd/3rd]

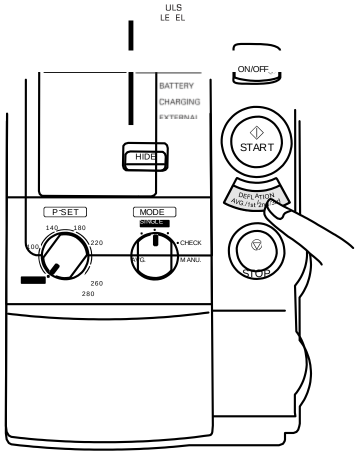

Accelerate deflation by pushing the DEFLATION (deflation control) / Measurement Result Display Switch Button during deflation in the measurement by the MANU Mode.

With each push of the [DEFLATION AVG./1st/2nd/3rd] Button, cuff is deflated rapidly in increments of 5 to 10 mmHg.

List of Functions for each Mode

Measurement Mode / Function	Single (SINGLE) Mode	Average (AVG) Mode	Auscultation (MANU) Mode	Check (CHECK) Mode
Inflation level setting	●	●	●	—
Non-display function	●	●	—	—
Manual deflation control	—	—	●	—

(4) AVG Function setting

You can set the number of measurements, the waiting time until the 1st measurement, and the measurement interval for the AVG Mode.

Function #	Items to set	Set value
F1	Number of measurements	**2 times** or 3 times
F2	Waiting time until the start of 1st measurement	**0 sec**, 3 min, 5 min, or 10 min.
F3	Measurement interval	5 sec, 30 sec, **1 min**, or 2 min.

Note: The bold letters represent the factory-set values.

Procedure to change the set values

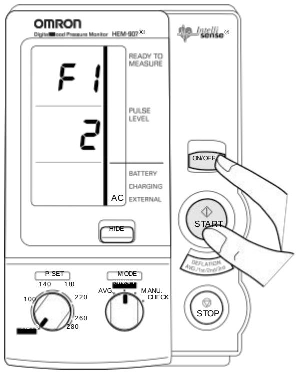

1) When the power is OFF, push the ON/OFF (power) Button for more than three seconds while holding the START Button; F1 is displayed.

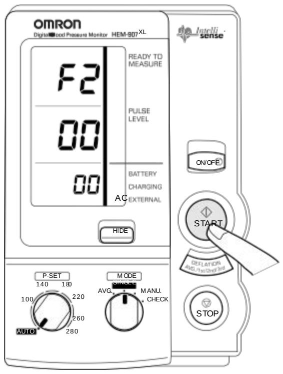

2) Push the START Button and select the function to set from F1 to F3. Each time you push the START Button, the functions change in the order of F1→F2→F3→ .

3) Push the DEFLATION (deflation control)/Measurement Result Display Switch Button and change the set values.

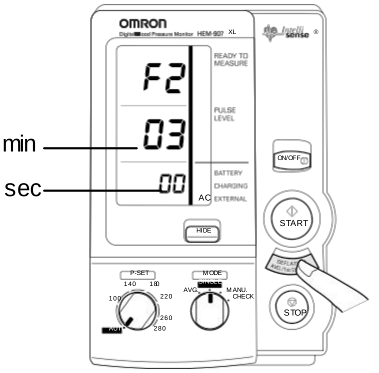

4) When the setting is finished, push the ON/OFF (power) Button to turn off the power. The setting is changed.

HOW TO APPLY THE CUFF

The cuff of OMRON IntelliSense® Blood Pressure Monitor HEM-907XL plays an important role of collecting the information on blood vessels. Please wrap the cuff according to the procedure, below.

Do not wrap the cuff over an arm to which intravenous injection or transfusion is being conducted, or when otherwise contraindicated.<br><br>Do not connect the air tube or the cuff to other equipment which is connected to intracorporeal organ. Air embolisms may result.	⃠

General advice

- Do not inflate the cuff without being wrapped over the arm.
- Do not use a damaged cuff.

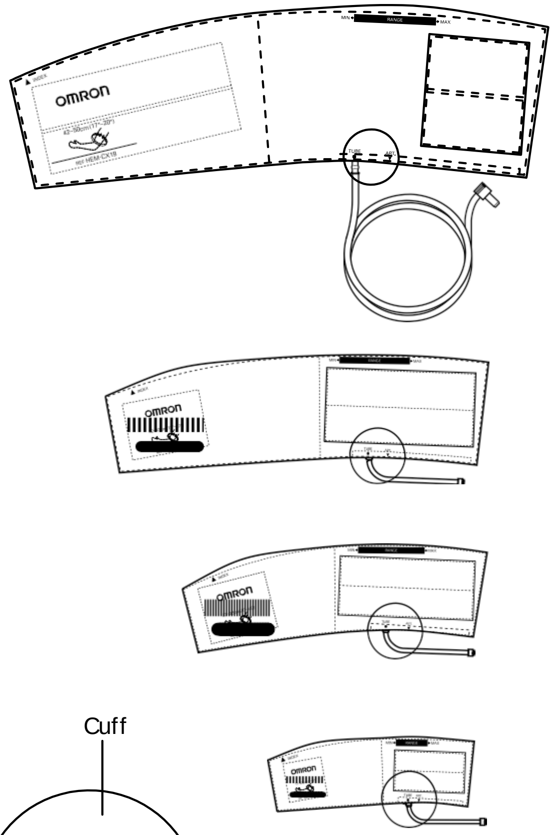

1. Select the cuff according to the arm size

Arm circumference		Name of the cuff
(7" - 9")	17-22 cm	HEM-907-CS19 (Small)
(9" - 13")	22-32 cm	HEM-907-CR19 (Medium)
(13" - 17")	32-42 cm	HEM-907-CL19 (Large)
(17" - 20")	42-50 cm	HEM-907-CX19 (Extra Large)

- Check the following before applying the cuff:

1) The bladder is correctly installed in the cuff.
2) The bladder is not twisted inside the cuff.
3) The bladder tube is protruding from the cuff as shown in the Figure on the right.

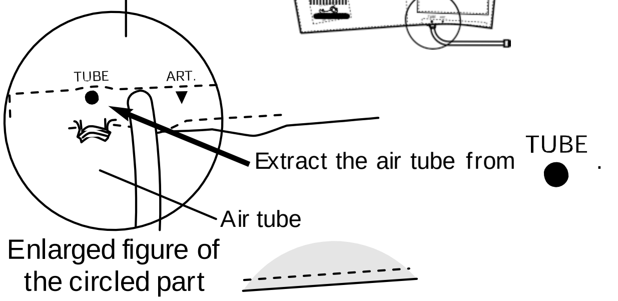

2. Connect the air tube securely.

To use the cuff in small, medium or large size

- Connect the air tube to the main unit by securing the air plug to the base of the air connector.
- Securely connect the air tube and the cuff/bladder set by rotating the luer connection as shown in the Figure on the right.

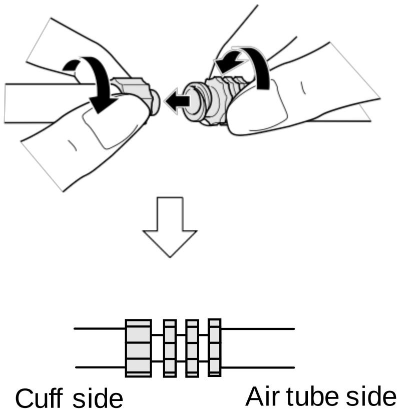

To use the cuff in extra-large size only

The extra-large cuff comes with an air tube with an integrated air plug. Connect the air plug of the cuff to the air connector securely when connecting to the main unit.

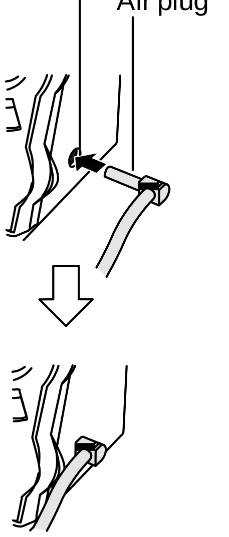

HOW TO APPLY THE CUFF

3. Place the right or left hand of the patient with the palm of hand facing upward.

4. Align the Artery Position Mark ART. ▼ with the brachial artery.

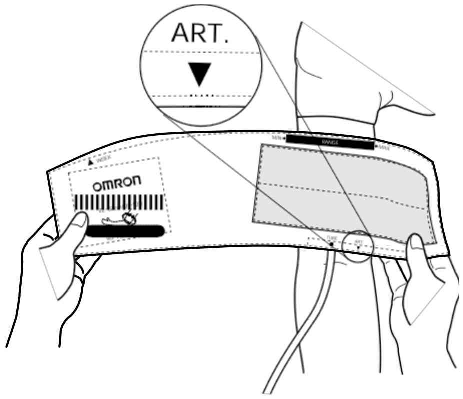

5. Wrap the cuff snugly using both hands and securely fasten it with the Velcro™ tape. At this time, the lower edge of the cuff must be placed 1/2" to 1" above the inner side of elbow joint.
   - If the INDEX is positioned outside the RANGE, select the cuff suitable for the patient’s arm circumference and wrap it again.
   - Wrap the cuff so that you can insert only one finger between the cuff and arm.

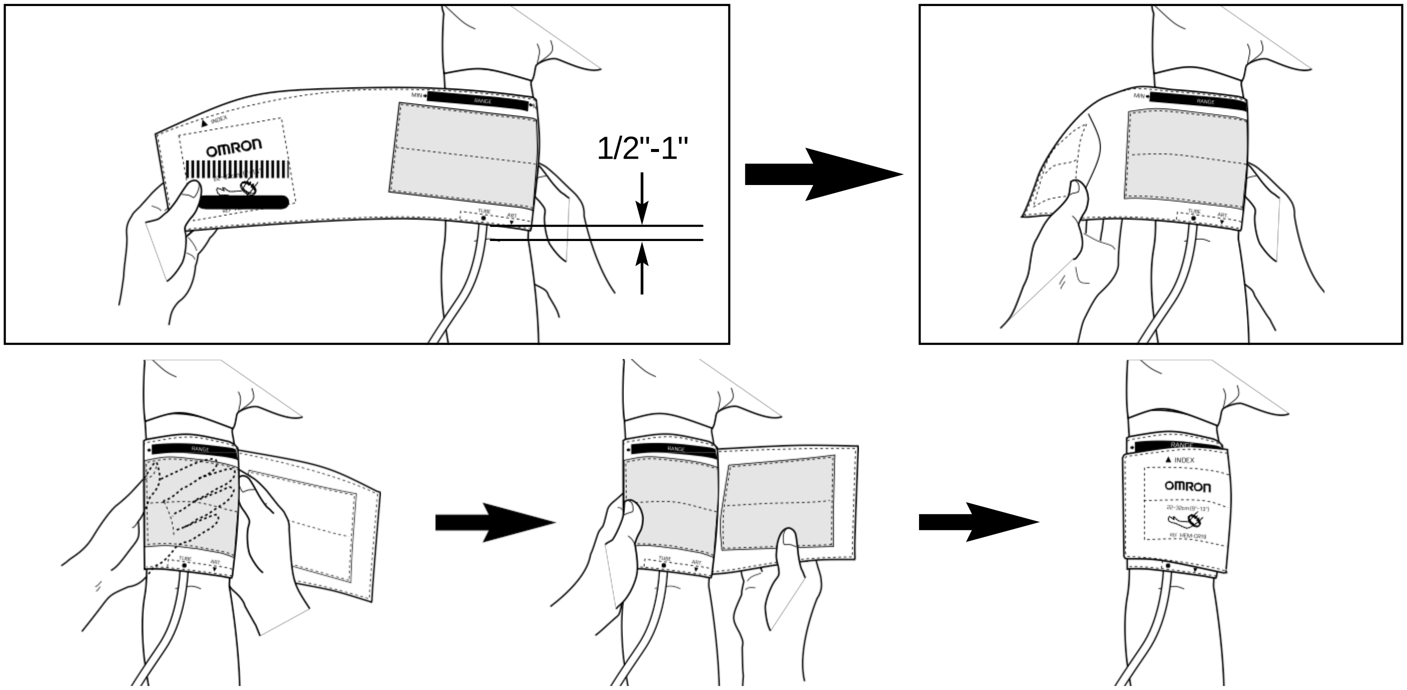

6. Keep the level of the cuff at the same level as the heart during the measurement.

HOW TO USE THE POWER SOURCE

- DANGER -
TO REDUCE THE RISK OF
FIRE OR ELECTRIC SHOCK,
CAREFULLY FOLLOW THESE INSTRUCTIONS.

⚠ For connection to a power supply not in the U.S.A., use an attachment plug adapter of the proper configuration for the power outlet.

⚠ The power unit (AC Adapter) is intended to be correctly oriented in a vertical or floor mount position.

NOTE: Use only the authorized AC Adapter that came with this monitor.

⚠ **CAUTION**

Use only authorized parts and accessories. Parts and accessories not approved for use with the device may damage the unit.

SAVE THESE INSTRUCTIONS

HOW TO USE THE POWER SOURCE

How to use the AC adapter

Do not use this unit in the presence of flammable gas, or anesthetics, or in a high pressure oxygen room or oxygen tent.	
Do not touch the AC adapter with wet hands.	

Be sure to use the AC adapter from the power supply of 110 VAC.	
Do not install or store this unit where it may come in contact with water or liquid medication.<br>This is a Class II device with double isolation. Earth pin is not for protective purposes.	

General advice
• Read the instruction manual of the other devices to be used at the same time with this unit to understand and be aware of the interaction between the devices.

Connect the AC adapter to the DC jack of the main unit [1] and the electric outlet [2].

NOTE: When the AC adapter is connected and the unit is turned off, the AC adapter charges the installed rechargeable battery.

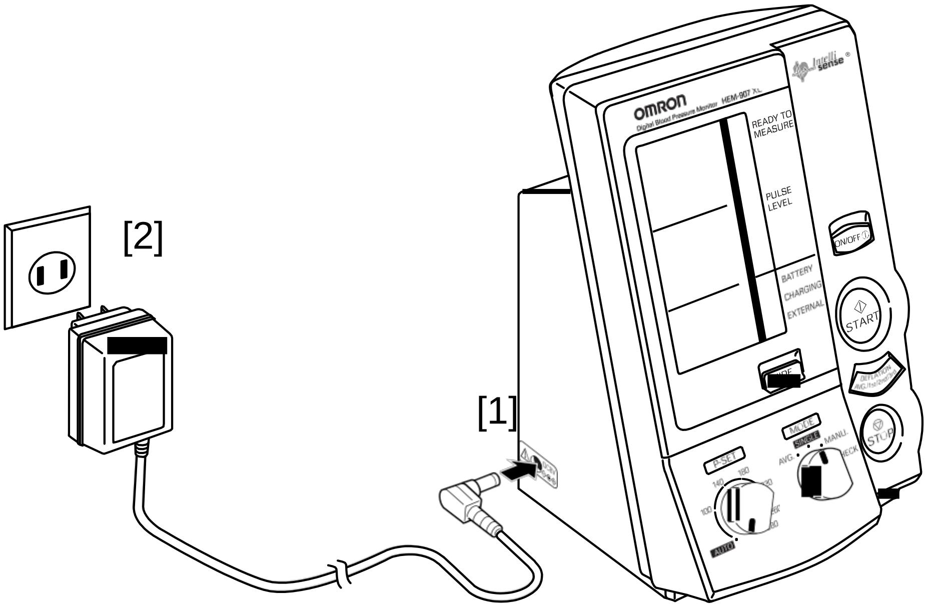

HOW TO USE THE POWER SOURCE

Installation and Replacement of Battery Pack

If battery fluid gets into your eye or comes in contact with skin, wash the affected area with water repeatedly. Immediately consult a doctor for treatment.	
Do not use the battery pack for devices other than for this unit.<br><br>Do not disassemble the battery pack.	

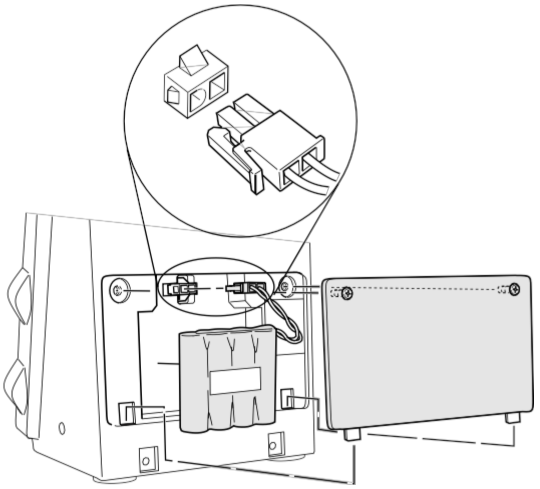

1. Remove both screws on the upper portion of the battery cover of this unit, and remove the cover.
2. Disconnect the old battery pack from the connector and replace with a new one.
3. Install the battery cover and fasten it with both screws. Be careful not to pinch the lead wire.
4. Connect the main unit to the AC adapter to charge the new battery. The battery is not charged when you purchase the monitor. When you use the battery for the first time, charge it for more than twelve hours before use.

Battery life

- You can use the unit for approximately three hundred measurements with one charge.
- Approximate life of battery is two years. However the battery life from each charging may be shortened depending on the state of using. If the interval between charging becomes short and the icon appears frequently, replace it.

Charging time

- At approximately five seconds after connecting the AC adapter, the unit will start battery charging automatically.
- While the battery is being charged, the icon turns on.
- The battery can be completely charged in approximately twelve hours.

Battery low

- When the icon starts to blink, twenty to thirty measurements remain on the battery. However if the Battery Low Mark starts to blink, charge it soon.
- If the icon is displayed, the battery is low and the unit cannot operate. Please charge the battery.

Automatic Power Off

- When using the unit with the battery, the unit will turn off automatically after five minutes of inactivity.
- While the AC adapter is connected, the Auto Power Off function does not work.

If cuff inflation does not stop, remove the cuff or pull out the air tube from the main unit.	!

General advice
• Patients should not touch the unit.

Confirm readings with the stethoscope when an irregular pulse wave is displayed or when the measured value is questionable or erratic.	!
Do not use a cellular phone near this unit.<br>Do not use this unit in a vehicle.	⊘

List of Measurement Modes

To measure only once	→ SINGLE Mode →	Refer to Page 19.
To measure two times (or three times) automatically and consecutively	→ AVG Mode →	Refer to Page 21.
To measure by using a stetho-scope	→ MANU Mode →	Refer to Page 23.

HOW TO MEASURE BLOOD PRESSURE (IN SINGLE MODE)

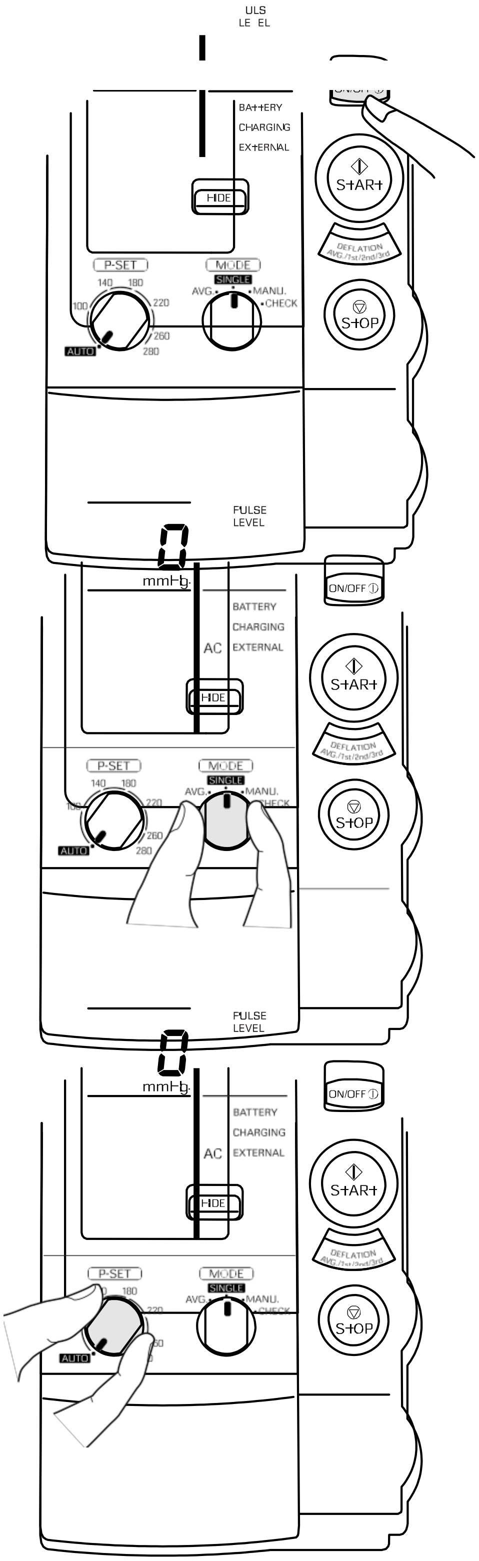

1. Push the ON/OFF (power) Button to turn on the power.

2. Set the MODE Selector to “SINGLE”.

3. Set the P-SET (inflation level) Knob to “AUTO” or to the target pressure value.

4. Measure the patient’s arm size, and wrap appropriate cuff over the patient’s arm. (Refer to Pages 13 and 14.)

HOW TO MEASURE BLOOD PRESSURE (IN SINGLE MODE)

5. Push the START Button to start the measurement.
   - Do not push the START Button without wrapping the cuff.
   - If you want to stop measurement, push the STOP Button. The cuff will rapidly deflate.

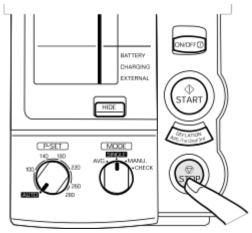

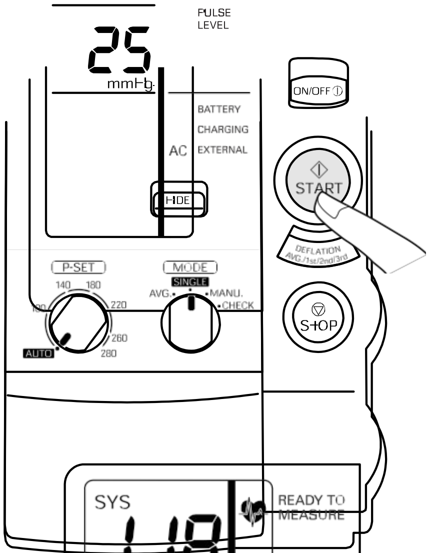

6. The measurement results are displayed.
   - While the battery pack is in use, the monitor will turn off automatically after five minutes of inactivity and the display (measurement results) will disappear. (Automatic Power Off)

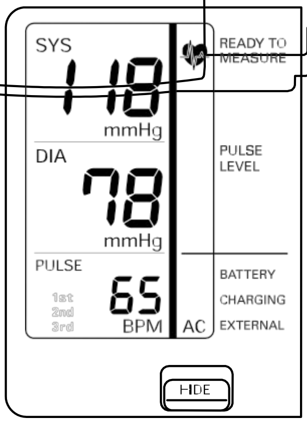

7. Push the ON/OFF (power) Button to turn off the power.

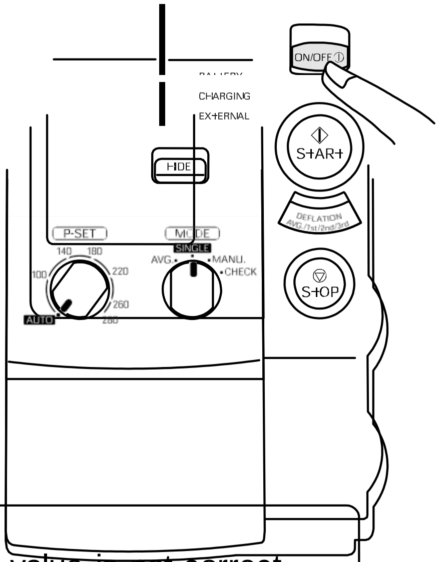

If the monitor determines that the pressure value is not correct, an error display appears (Er1 to 9). In this case, refer to Page 27 and start the measurement again.

HOW TO MEASURE BLOOD PRESSURE (IN AVERAGE MODE)

1. Push the ON/OFF (power) Button to turn on the power.

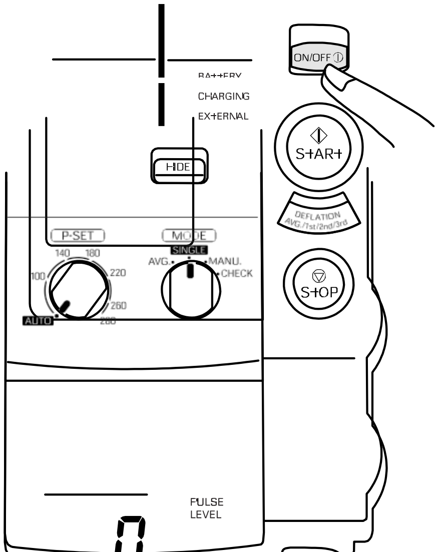

2. Set the MODE Selector to "AVG".

   The factory-set values are set as follows:

   - Number of measurements: 2
   - Waiting time until the 1st measurement: 0 sec.
   - Interval: 1 min.

   To change these factory-set values, refer to Page 12.

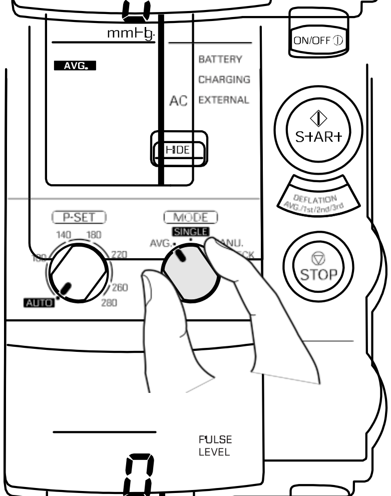

3. Set the P-SET (inflation level setting) Knob to "AUTO" or the target pressure value.

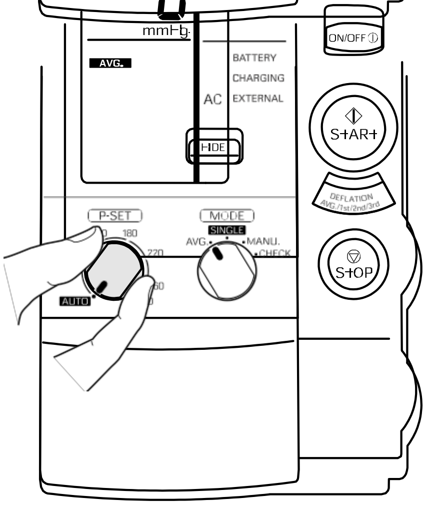

4. Measure the patient's arm size and wrap appropriate cuff over the patient's arm. (Refer to Pages 13 and 14.)

HOW TO MEASURE BLOOD PRESSURE (IN AVERAGE MODE)

5. Push the START Button to start the measurement.

   After the pre-select waiting time, the unit takes the 1st measurement.

   After displaying the results of 1st measurement, subsequent measures occur automatically at the specified intervals.

   - For setting the number of measurements, the waiting time until the 1st measurement, and the interval, refer to Page 12.
   - If you want to stop measurement, push the STOP Button. The unit will rapidly deflate.
   - If an error occurs during measurement, the monitor will automatically start measurement again. If a second error occurs, measurement will automatically stop.
   - Do not push the START Button without wrapping the cuff.

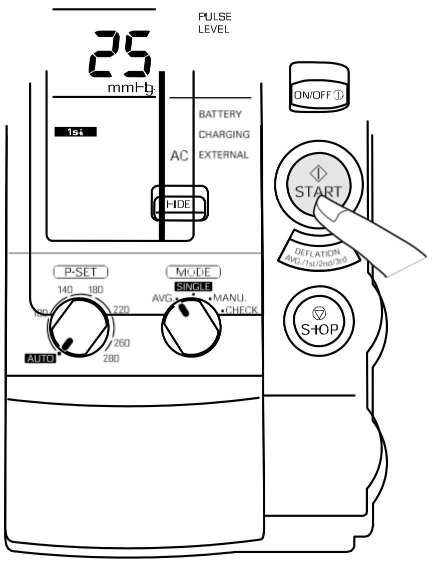

6. The measurement results are displayed.

   After all the measurements are finished, average values will be displayed.

   Each time the DEFLATION (deflation control) / Measurement Result Display Switch Button is pushed, the measurement results for each reading and the average value will be displayed.

   - While the battery is in use, the monitor will turn off after five minutes of inactivity and the display (measurement results) will disappear. (Automatic Power Off)

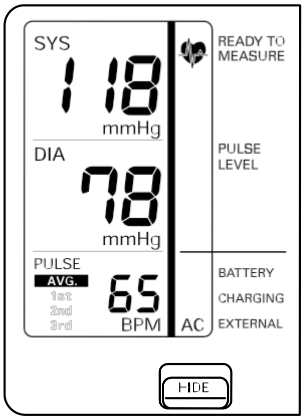

7. Push the ON/OFF (power) Button to turn off the power.

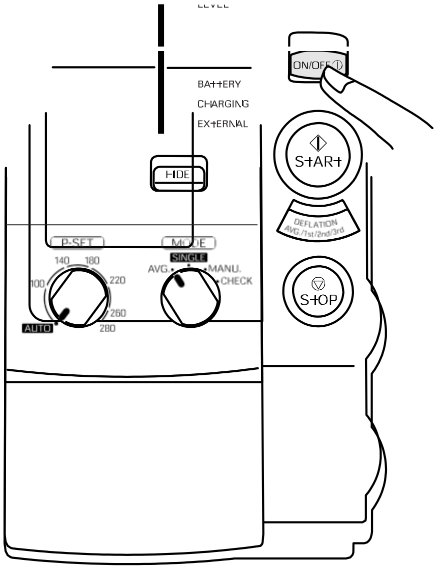

If the monitor determines that the pressure value is not correct, an error display appears (Er1 to 9). In this case, refer to Page 27 and start the measurement again.

HOW TO MEASURE BLOOD PRESSURE (IN MANUAL MODE)

1. Push the ON/OFF (power) Button to turn on the power.

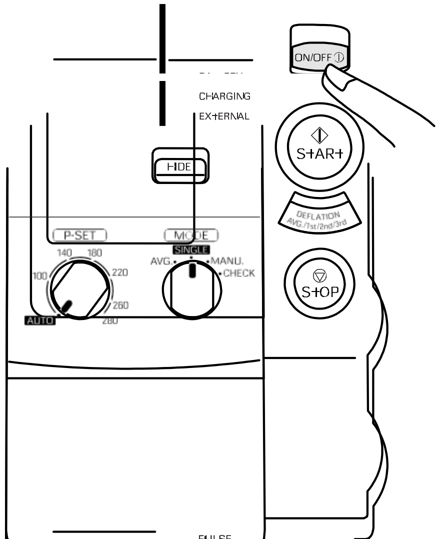

2. Set the MODE Selector to “MANU”.

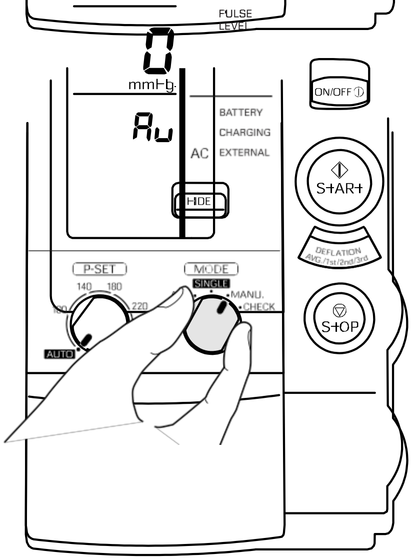

3. Set the P-SET (inflation level setting) Knob to “AUTO” or the target pressure value.

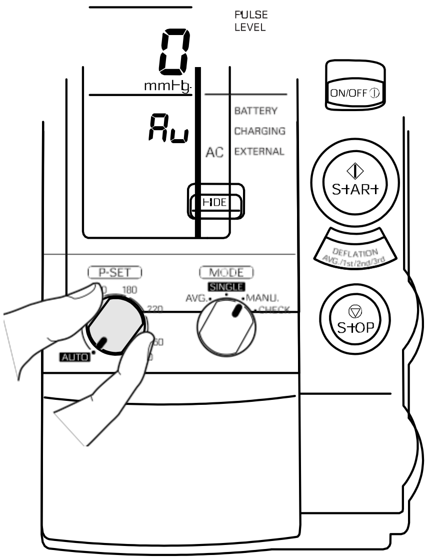

4. Measure the patient’s arm size and wrap appropriate cuff over the patient’s arm. (Refer to pages 13 and 14.)

HOW TO MEASURE BLOOD PRESSURE (IN MANUAL MODE)

5. Place the stethoscope on the patient's arm.

6. Push the START Button to start the measurement.
   - Do not push the START Button without wrapping the cuff.
   - Do not squeeze or press the cuff during the measurement.
   - If you want to inflate again after the start of deflation, push the START Button.
   - If you want to accelerate deflation after the start of deflation, push the DEFLATION (deflation control) / Measurement Results Display Switch Button. Each time the Button is pushed, cuff is deflated rapidly in increments of 5 to 10 mmHg.

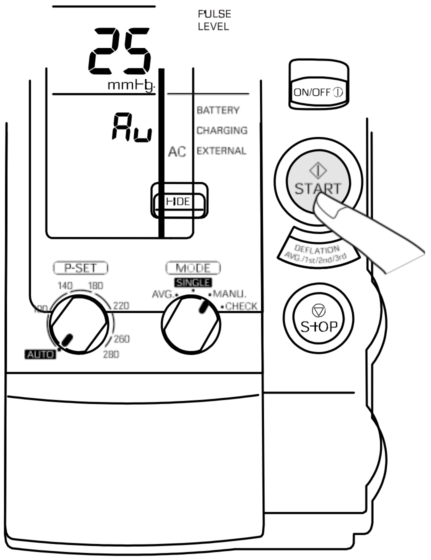

7. Take the readings.

8. Push the STOP Button to remove air inside the cuff.
   - The unit does not automatically deflate in the MANU Mode.

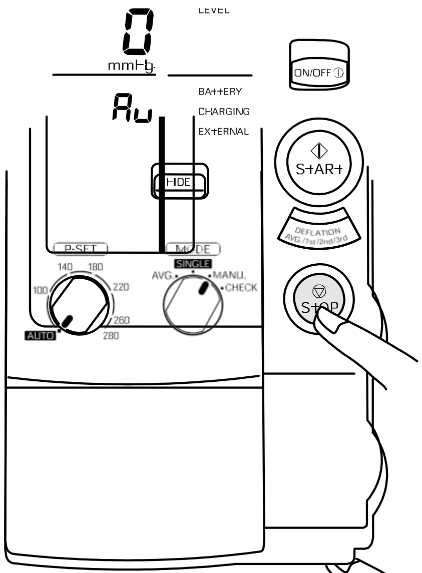

9. Push the ON/OFF (power) Button to turn off the power.

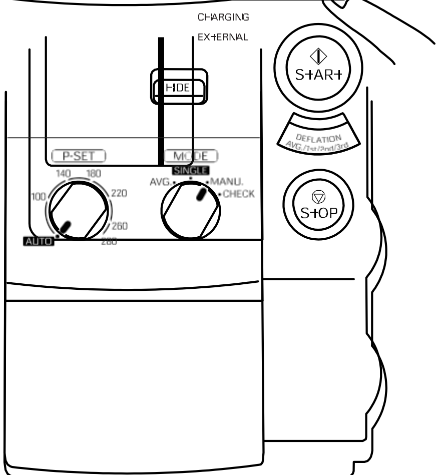

If the monitor determines that the pressure value is not correct, an error display appears (Er1 to 9). In this case, refer to Page 27 and start the measurement again.

HOW TO CHECK PRESSURE ACCURACY (IN CHECK MODE)

Accuracy of pressure display can be checked in the CHECK Mode.

What you need to prepare

(1) Calibrated mercury manometer (including inflation bulb), (2) T-tube, (3) two air tubes, and (4) a sturdy cylindrical shaped object on which the cuff is wrapped.

How to check

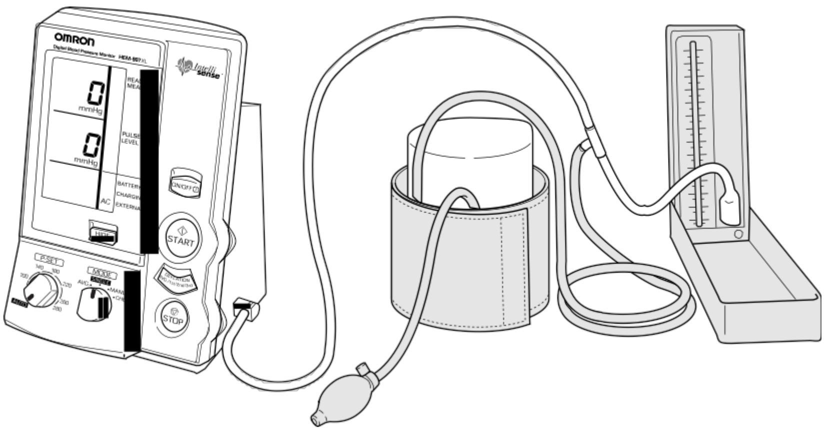

1. Connect the manometer, inflation bulb, cuff, and the monitor with the T-tube as shown in the figure on the right.
2. Tightly wrap the cuff over a sturdy cylinder.
3. Release the valve of inflation bulb to remove the air inside the cuff completely.
4. Push the ON/OFF (power) Button to turn on the monitor.
5. Set the MODE Selector to "CHECK".
6. Close the valve of inflation bulb and inflate the cuff to the pressure to be checked, based on the manometer read.
7. Compare the pressure values displayed on the monitor to the one on the manometer.

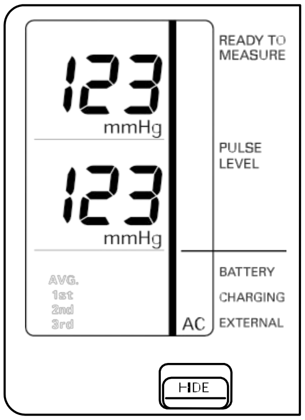

Check result

Accuracy of the monitor is validated to be ±3 mmHg or 2% of standard manometer reading. If your result shows a difference exceeding the tolerance, contact Omron Healthcare's Cusomer Service at 1-877-216-1336.

HOW TO CLEAN THE UNIT AFTER USE

When cleaning this unit, unplug the AC adapter from the electric outlet.	
After cleaning this unit, dry it well, before plugging the AC adapter in the electric outlet.	

**General advice**
• Do not clean this unit with gasoline, paint thinner, or high concentration alcohol.<br>• Do not disinfect this unit by autoclave or gas steril-ization (EtO, gluteraldehyde, or high concentration ozone.)

1. Wipe the monitor with a soft, damp cloth diluted with disinfectant alcohol, or diluted detergent.
2. Complete cleaning by wiping the monitor with a soft, dry cloth.

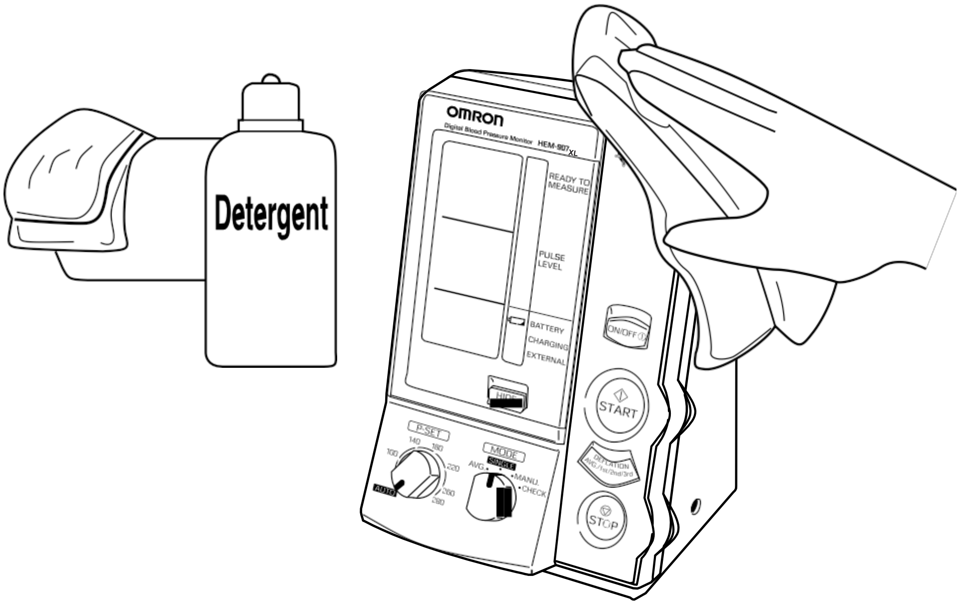

LIST OF ERROR CODES

<table>
<tr><th>Error code</th><th>Explanation</th><th>How to correct</th></tr>
<tr><td>Er1</td><td>Inflation error
• When the pressure does not exceed 12 mmHg within the set time after the start of inflation
• When the inflation does not reach the set cuff pressure within the specified time after the start of inflation</td><td rowspan="2">• Confirm that the air tube connecting the cuff and the main unit is connected securely.

• Confirm that the air flow in the air tube connecting the cuff and the main unit isn’t being restricted.

• Confirm that the cuff is wrapped correctly (refer to pages 13 and 14).

• Check bladder for leaks and, if necessary, replace the bladder with new one (option).</td></tr>
<tr><td>Er2</td><td>Deflation error
• When the deflation speed is too fast during the measurement
• When the deflation speed is too slow during the measurement
• When the measurement does not finish within the specified time after starting the measurement</td></tr>
<tr><td>Er3</td><td>Overpressure error
• The cuff pressure exceeded 299 mmHg.</td><td>• Confirm that air flow in the air tube connecting the cuff and the main unit isn’t being restricted.</td></tr>
<tr><td>Er4</td><td>Insufficient inflation error
• Blood pressure could not be measured due to insufficient inflation level.</td><td>• If the measurement is made by setting the P-SET to “AUTO”, ask the patient not to move during the inflation.
• Confirm that the P-SET is securely set to “AUTO”. Turn the Knob counterclockwise as far as it goes until you can hear a click sound.
• If the measurement is made by manual inflation level setting, set the value to 30 to 40 mmHg higher.</td></tr>
<tr><td>Er5</td><td>Indeterminable blood pressure error
• Blood pressure could not be measured even when the cuff pressure reached the specified pressure.</td><td>• Confirm that the cuff is wrapped correctly (refer to pages 13 and 14).</td></tr>
<tr><td>Er6</td><td>Low pulse level error
• Pulse wave was too small.</td><td>• Confirm that the cuff is wrapped correctly (refer to pages 13 and 14).</td></tr>
<tr><td>Er7</td><td>Blood pressure error
• Relationship between systolic and dias-tolic pressures was abnormal.</td><td rowspan="2">• Ask the patient not to move during the measure-ment.

• Check the patient for arrhythmia.</td></tr>
<tr><td>Er8</td><td>Pulse rate error
• Pulse rate did not stay within the range of 30 to 199 beats/min.</td></tr>
<tr><td>Er9</td><td>Device error
• Main unit malfunction.</td><td>• Contact Omron Healthcare’s Cusomer Service toll-free at 1-877-216-1336.</td></tr>
</table>

TROUBLESHOOTING

If the unit malfunctions during use, please check the following:

Trouble	What to inspect	How to correct
The unit inflates to abnormally high (low) pressure.	Is the cuff wrapped correctly?	Wrap the cuff correctly, and measure again. (Refer to Page 13 and 14.)
	Is the patient moving during inflation?	Ask the patient not to move during measurement, and measure again.
	Does this patient have arrhythmia?	Set the P-SET to 30 to 40 mmHg higher than estimated systolic pressure of the patient, then measure.
The monitor cannot measure blood pressure.<br><br>Measured values are abnormally high (low).	Check the patient's condition.	After checking the patient with the stethoscope refer to the "list of error codes". (Refer to Page 27.)
	Is the patient moving during the measurement?	Ask the patient not to move during measurement, and measure again.
	Does the patient have an arrhythmia?	Set the P-SET to 30 to 40 mmHg higher than estimated systolic pressure of the patient, then measure.
	Is the size of the cuff correct and is it wrapped correctly?	Select the cuff according to the patient's arm circumference, wrap it correctly, then measure again. (Refer to Pages 13 and 14.)
	Is the level of the brachium to which the cuff is wrapped at the same level as the heart?	Keep the level of the brachium to which the cuff is wrapped at the same level as the heart, then measure again.
	Are the patient's clothes restricting normal blood flow to the arm?	Remove the clothing and measure again.

CAUTION

CAUTION:

Changes or modifications not expressly approved by Omron Healthcare, Inc. could void the user's authority to operate this product.

NOTE:

POTENTIAL FOR RADIO/TELEVISION INTERFERENCE (for U.S.A. only)

This product has been tested and found to comply with the limits for a Class B digital device, pursuant to part 15 of the FCC rules.

These limits are designed to provide reasonable protection against harmful interference in a residential installation. The product generates, uses, and can radiate radio frequency energy and, if not installed and used in accordance with the instructions, may cause harmful interference to radio communications. However, there is no guarantee that interference will not occur in a particular installation. If the product does cause harmful interference to radio or television reception, which can be determined by turning the product on and off, the user is encouraged to try to correct the interference by one or more of the following measures:

- Reorient or relocate the receiving antenna.
- Increase the separation between the product and receiver.
- Connect the product into an outlet on a circuit different from that to which the receiver is connected.
- Consult the dealer or an experienced radio/TV technician for help.

POTENTIAL FOR RADIO/TELEVISION INTERFERENCE (for Canada only)

This digital apparatus does not exceed the Class B limits for radio noise emissions from digital apparatus as set out in the interference-causing equipment standard entitled "Digital Apparatus", ICES-003 of the Canadian Department of Communications.

Cet appareil numérique respecte les limites de bruits radioeléctriques applicables aux appareils numériques de Classe B prescrites dans la norme sur le matériel brouilleur: "Appareils Numériques", NMB-003 èdictée par le ministre des communications.

FIVE YEAR LIMITED WARRANTY

Your HEM-907XL IntelliSense® Automatic Blood Pressure Monitor is warranted to be free from manufacturing defects for a period of five years under normal use. The five year warranty excludes the monitor cuff. The cuff is warranted for a one year period.

This warranty extends only to the original retail purchaser.

To obtain warranty service contact Omron Healthcare's Customer Service by calling 1-877-216-1336 for the address of the repair location and the return shipping and handling fee. Information for warranty service is avalable on our website at www.omronhealthcare.com

We will either repair or replace (at our option) free of charge any parts necessary to correct defects in the materials or workmanship.

The above warranty is complete and exclusive. The warrantor expressly disclaims liability for incidental, special, or consequential damages of any nature. (Some states do not allow the exclusion or limitation of incidental or consequential damages, so the above warranty may not apply to you.)

Any implied warranties arising by the operation of law shall be limited in duration to the term of this warranty. (Some states do not allow limitations on how long an implied warranty lasts, so the above limitation may not apply to you.)

This warranty gives you specific legal rights, and you may have other rights which vary from state to state.

FOR CUSTOMER SERVICE CALL TOLL FREE: 1-877-216-1336

SPECIFICATIONS

Name:	OMRON Digital Automatic Blood Pressure Monitor
Model:	**HEM-907XL (HEM-907-Z2)**
Display:	Digital display
Measurement:	Oscillometric method
Measurement Range:	Pressure; 0 to 299 mmHg<br>Pulse rate; 30 to 199 beats/min
Accuracy:	Pressure; Within ±3 mmHg or 2%<br>Pulse rate; Within ±5% of reading
Inflation:	Automatic inflation with pumping
Deflation:	Automatic deflation by electromagnetic control valve
Air Release:	Automatic rapid air release by electromagnetic control valve
Pressure Detection:	Electrostatic capacity semi-conductor pressure sensor
Power supply:	AC adapter (120 VAC, 60 Hz, 13 VA) or (120VAC, 50/60Hz, 0.2A)<br>Battery pack (4.8 VDC, 6W)
Electic Shock Protection Method:	Class II B type
Operating Temperature and Humidity:	50°F to 104°F (10 to 40°C), 30 to 85% RH, IPX 0 Rating
Weight of Main Unit:	Approx. 32 oz (910 g)
External Dimensions:	Approx.5 1/2" (W) x 8"(H) x 5 1/6"(D)<br>139 (W) x 203 (H) x 131 (D) mm
Accessories:	Cuff / bladder set Extra Large, Cuff / bladder set Large, Cuff / bladder set Medium, Cuff / bladder set Small,<br>AC adapter, Battery pack, air tube 1.3 m (51 3/16"), Instruction Manual
Options:	Air tube 1.3 m (51 3/16"), Stand exclusive for this unit (Item: HEM-907-STAND), Wall-hanging kit (Item: HEM-907-WKIT), Pole-mounting kit (Item: HEM-907-PKIT)

Complies with:	IEC 60601-1:1998+A1:1991+A2:1995	General requirements for safety
	IEC 60601-1-2:1993	Electromagnetic compatibility - Requirements and tests
	AAMI SP10:1992	Manual, Electronic or Automated Sphygmomanometers

Please note that specifications may be changed without prior notice.

C UL US CLASSIFIED 58JN

Medical Equipment with respect to electric shock, fire and mechanical hazards only in accordance with UL 2601-1 and CAN/CSA C22.2 No. 601.1

For Customer Service
Call Toll Free

1-877-216-1336

Made in Japan

Manufactured by OMRON HEALTHCARE

Distributed by:
OMRON HEALTHCARE, INC.
1200 Lakeside Drive
Bannockburn, Illinois 60015
www.omronhealthcare.com

Copyright © 2009 OMRON HEALTHCARE, INC.

1628519-0E

Chapter 3.a Screening Visit Procedures

3.a.1 Determining Eligibility

The participant's history must be thoroughly investigated and **applicable inclusion & exclusion clearly documented in the participant's source documentation**. The criteria have been developed to define a particular participant population as well as to protect ineligible participants from unnecessary risks. Therefore, careful consideration should be given to determining eligibility in the SPRINT trial. Most of the tests needed to document eligibility are considered to be part of routine care for potential participants under the care of a SPRINT investigator and are not charged to the study; however, potential participants should not be charged for the cost of tests performed exclusively for study screening purposes. The following discussion provides an overview of the criteria contained on the **Inclusion/Exclusion Summary Form**. Applicable inclusion & exclusion criteria should be carefully evaluated and clearly documented in the source documentation of the participant's study chart. To provide the most complete assessment of a participant's eligibility, all applicable inclusion criteria should be documented.

PART A. DEMOGRAPHICS

Complete Part A items 1-6 on the **Inclusion/Exclusion Summary Form** for every participant who comes in to the clinic for screening. Items 1 – 6 include 1) name, 2) how the participant heard about the study, 3) gender, 4) race/ethnicity, 5) Spanish/Hispanic/Latino origin, and 6) birth date. Participants are ineligible if they are not $\geq$50 years of age at the time of screening, although they may be rescreened after their 50th birthday.

PART B. BLOOD PRESSURE AND ANTIHYPERTENSIVE MEDICATION USE

7. Current BP Medications

7a). Record all of the BP medications taken at screening. If no BP medications are taken, check the box indicating this.

Table 3a3 lists the FDA-approved antihypertensive drugs and the dosage ranges that are considered "antihypertensive." For the purpose of eligibility, BP-lowering medications, even if given for a purpose other than BP-lowering, are to be considered antihypertensive medications if total daily dose falls within the range indicated in the table. Doses or frequencies below the lowest ranges in this table do not count as antihypertensive drugs/doses for the purposes of SPRINT. Several short-acting drugs (e.g., furosemide) do not count as antihypertensive drugs if given once daily, regardless of the dose. However, after randomization, any dose/frequency of a BP-lowering medication the participant is taking should be reported on the SPRINT Blood Pressure Medications Management Log form.

Below are some specific considerations for counting the number of medications:

1. Each component of a combination antihypertensive medication should be counted as a separate drug, except that two diuretics in combination (e.g., HCTZ + triamterene) are counted as one drug.
2. Loop diuretics are considered antihypertensive medications if used at doses/frequencies known to reduce blood pressure, specifically:
    - Furosemide greater than or equal to 20 mg BID (once-a-day regimens at any dose do not qualify)
    - Bumetanide greater than or equal to 0.5 mg BID (once-a-day regimens at any dose do not qualify)

- Ethacrinic acid greater than or equal to 25 mg BID (once-a-day regimens do not qualify)
- Torsemide: any dose

3. Nitroglycerine is not considered an antihypertensive medication.

8a) Does the participant have an arm circumference too large (> 50 cm) to allow accurate blood pressure measurement with available devices?
If yes, then participant is ineligible. The Extra Large cuff for the Omron device is only able to accommodate arm circumferences up to 50 cm.

8b) Per the Omron instructions, obtain 3 BP readings and record all 3 and the average.

8c) Does the participant have one-minute standing SBP < 110 mm Hg?
If yes, then the participant is ineligible but may be rescreened at a future visit (e.g., after considering down-titration of any current BP-lowering medications). In a participant with a history of standing BP < 110 mm Hg, orthostasis may pose a potential safety risk. This exclusion is not applicable if the participant is unable to stand due to wheelchair use or amputation without prosthesis. Standing SBP must be measured in the clinic during the screening process.

8d) Sitting systolic blood pressure at screening:

a) SBP: 130 – 180 mm Hg on 0 or 1 medication
b) SBP: 130 – 170 mm Hg on up to 2 medications
c) SBP: 130 – 160 mm Hg on up to 3 medications
d) SBP: 130 – 150 mm Hg on up to 4 medications

If a screenee is otherwise eligible for SPRINT but presents with a treated blood pressure/medication number that falls outside the SPRINT eligibility criteria, BP-lowering medications may be adjusted prior to the randomization visit to determine whether the SBP will rise or fall to meet the SBP and number of medications criteria.

When adjusting antihypertensive medications to see if a screenee can meet the SBP/number of drugs eligibility criteria, clonidine and beta-blockers should be tapered to a low dose for 3-7 days before discontinuing, if the screenee is not already on a low dose (e.g., clonidine 0.1 mg BID). Other drugs may be stopped completely and abruptly, regardless of dosage, but the investigator may choose to reduce the dosage before discontinuing in some situations where a screenee is on a high dosage and there is concern about how high the BP will rise.

If a screenee has a non-hypertension indication for a BP-lowering medication (e.g., beta-blocker post-MI, RAS blocker for proteinuria, or alpha blocker for benign prostatic hypertrophy), the screenee should be on the appropriate dose of such medication before assessing whether he/she meets the SPRINT inclusion criteria. If the investigator believes that a potential participant has such an indication but is not receiving appropriate treatment, he/she should encourage the potential participant's primary care provider to consider placing the participant on the appropriate therapy prior to initiating or resuming the screening process.

There are no diastolic blood pressure (DBP) inclusion criteria because risk in the anticipated SPRINT population is more related to SBP than DBP

9). A screenee who presents on no BP medications should have documentation of SBP ≥130 mmHg on 2 visits within 3 months prior to the randomization visit in order to be eligible for the trial.

Part C. CARDIOVASCULAR DISEASE HISTORY & RISK FACTORS

The purpose of Part C of the screening form is to determine eligibility for SPRINT based upon a history of known cardiovascular disease or risk factors for the future development of cardiovascular disease. Specifically, Items 10a-e identify participants with a CVD history. Items 11a-e identify participants with indications of clinical or subclinical CVD. Items 12, 13, 14, and 15 assess risk factors that indicate a high likelihood of a cardiovascular event in the future.

Applicable inclusion & exclusion criteria should be carefully evaluated and clearly documented in the source documentation of the participant's study chart. Please note that a participant could answer no to Items 10a-e but still be eligible based on Items #11a-e, 14c, 15a or age > 75 years. Participants age 75 and older will automatically qualify regardless of their FRS. The lab items total cholesterol and HDL cholesterol will not be required for this senior population but other items on the form including number of BP medications, SBP, CVD history and eGFR must still be collected and recorded.

10a-e obtain CVD History:

10 a. Has the participant had a documented myocardial infarction > 3 months ago?
Documentation of an old or age-indeterminate myocardial infarction (MI) may be determined by one of the following: diagnostic EKG Q-waves; akinesis or dyskinesis on echocardiogram, MUGA, or ventriculogram; prior hospital discharge diagnosis, or elevations in cardiac enzyme tests indicative of MI. If enzyme tests for this particular MI were performed on more than one date, please enter the date of the total CPK, CK-MB, or Troponin-I (or T) that first documents the MI, or verification from the primary or consulting physician that an MI has occurred (ideally a discharge summary). The date and location of the most recent MI may be listed on the source document. If none of the above is available, the clinician may document after taking a careful history that in their judgment the participant's self-report of the event is consistent with that of a prior MI. If the most recent MI has occurred within the past 3 months, the screenee is currently ineligible based on exclusion criteria 29, but may be rescreened after the 3-month window has passed.

10b. Has the participant had an acute coronary syndrome with or without resting ECG changes, ECG changes on a graded exercise test, or a positive cardiac imaging study?
This can be identified with noninvasive cardiac diagnostic procedures such as:

- Exercise testing - ST depression ≥ 1mm for ≥ 1 minute in 2 or more contiguous leads
- Stress echocardiography- fixed regional or reversible wall motion abnormality
- Stress nuclear perfusion study - fixed or reversible defect consistent with prior infarct or ischemia

Note: When combined with graded exercise treadmill (GXT) or pharmacologic stress, perfusion imaging can be performed with several different nuclear imaging agents, including Thallium, Myoview, and others. SPECT refers to a type of image display, with computer generation to show the areas that do not take up the imaging agent. There is also a technique called planar image display that some diagnostic laboratories may use in some participants. If a participant has had multiple tests of the same or different kind (e.g., a GXT and a thallium), use the most

recent test result to answer this question. A copy of the report should be kept in the participant's chart.

10c. Has the participant had a documented coronary revascularization procedure > 3 months ago?
Identification of the specific type of coronary revascularization should be listed in the source documents with supportive evidence filed in the participant's chart. Some examples would be coronary artery bypass graft (CABG) surgery, stent placement, percutaneous coronary intervention (PCI), rotoblation, or laser (LEAD) atherectomy. If the most recent procedure has occurred within the past 3 months, the person is currently ineligible based on exclusion criteria 29, but may be rescreened after the 3-month window has passed.

10d. Has the participant had a documented peripheral, carotid or abdominal aortic revascularization procedure > 3 months ago?
Some examples would include carotid or peripheral endarterectomy, angioplasty, stent placement, atherectomy, bypass, or abdominal aortic aneurysm repair. Revascularization procedures not specifically listed under this criterion may be included under "other." The specific procedure, date, and location should be listed in the source documentation and copies of the hospital discharge summary or procedure report kept in the participant's chart. If the most recent procedure has occurred within the past 3 months, the person is currently ineligible based on exclusion criteria 29, but may be rescreened after the 3-month window has passed.

11a-e Obtain clinical or subclinical CVD:
11a. Does the participant have ≥ 50% stenosis of a coronary, carotid, or lower extremity artery (within the past 2 years)?
This would be documented by evidence from Doppler ultrasound or angiography, with the date and procedure listed in the source documents. A copy of the procedure report should be retained as source documentation.

11b. Does the participant have an abdominal aortic aneurysm (AAA) ≥ 5 cm with or without repair (within the past 2 years)?
AAA size may be assessed via ultrasound or AAA surgical repair with an operative report. The date and applicable procedure should be listed in the source documents. A copy of the procedure report should be retained as source documentation. If the most recent repair procedure has occurred within the past 3 months, the person is currently ineligible based on exclusion criteria 29, but may be rescreened after the 3-month window has passed.

11c. Does the participant have a coronary artery calcium score ≥ 400 Agatston units (within the past 2 years)?
The purpose of a calcium score is to determine if a participant is at high risk for coronary artery disease. In general, a high calcium score is associated with a higher risk of cardiovascular events. A calcium score estimates the burden of calcified plaque in the coronary arteries and is calculated from either Electron Beam or multi-detector Computed Tomography images (EBCT or MDCT). A copy of the cardiac CT report documenting the calcium score should be retained as source documentation.
11d. Does the participant have a low ankle-brachial index (ABI) of ≤ 0.9 (within the past 2 years)?
This ratio is indicative of advanced arterial obstruction and is obtained using Doppler ultrasound and a specific protocol. The measurements and date obtained should be listed in the source documents.

11e. Does the participant have left ventricular hypertrophy (LVH) by ECG or echocardiogram criteria (within the past 2 years)?An ECG or echocardiogram used for this criterion must have been done within the last 2 years prior to randomization and a copy of the qualifying tracing or report kept in the participant's chart for verification.
LVH by ECG includes any one of the following:

- R amplitude in V5 or V6 > 26 mm
- R amplitude in V5 or V6 plus S amplitude in V1 > 35mm
- R amplitude in aVL > 12 mm
- R amplitude in Lead I > 15 mm
- R amplitude in Leads II or III, or aVF > 20 mm
- R amplitude in Lead I plus plus S amplitude in Lead II1 > 25mm
- R amplitude in aVL plus S amplitude in V3 > 28 mm (men) or > 22 mm (women)
- Computerized ECG machine report documenting LVH

For visual LVH reading, QRS amplitudes are measured in the *second to last complete normal beat* of the lead. A computerized reading indicating "borderline" or possible" LVH should be measured for the above listed voltage criteria and documented as such on the tracing. LVH by echocardiogram includes a combined wall thickness of 25 mm or more, which refers to the posterior wall plus the interventricular septum.

If an ECG is not available within the prior 24 months, consider obtaining locally if it will assist in determining a participant's eligibility.

12. What is the participant's most recent serum creatinine (within past 6 months)?
List the most recent serum creatinine result and date performed. Retain a copy of the report in the chart. If there are 2 or more values available in the medical records, the most recent value takes precedence. If you do not have a serum creatinine value from the past 6 months, you will need to draw blood for local lab tests to determine eligibility.

What is the participant's most recent total cholesterol (within the past 12 months)?
List the most recent total cholesterol result and date performed. Retain a copy of the report in the chart. If the participant is currently receiving lipid-lowering medication, be sure to document the therapy in the source documents. If there are 2 or more values available in the medical records, the most recent value takes precedence. If a value < 12 months old is not available, consider obtaining locally if it will assist in determining a participant's eligibility.

What is the participant's most recent HDL cholesterol (within the past 12 months)?
List the most recent HDL result and date performed. File a copy of the qualifying result in the chart. If a value < 12 months old is not available, consider obtaining locally if it will assist in determining a participant's eligibility.

13. Does the participant currently smoke?
If the participant is currently smoking cigarettes or has smoked in the past 30 days, the participant would meet this criterion. No other tobacco use qualifies. Document the participant's cigarette smoking history in the source documents.

14a-c. Does the participant have an estimated GFR $\geq$ 20 $\leq$ 59 ml/min/1.73m^2 within the last 6 months?
An estimated eGFR 20 – 59 mL/min/1.73m^2 is based on the 4-variable Modification of Diet in Renal Disease (MDRD) equation and most recent serum creatinine value within the past 6 months. If you do not have a serum creatinine value from the past 6 months, you will need to

draw blood for local lab tests to determine eligibility. If you have a serum creatinine value from the past 6 months without an eGFR, you can go to the website www.MDRD.com to calculate eGFR based on the participant's age, gender, ethnicity, and serum creatinine.

15a-b. Does the participant have a Framingham Risk Score ≥ 13 (men); ≥ 16 (women)? Note that if the participant is 75 years of age or older, they are automatically eligible regardless of the FRS. Framingham risk scores are based upon total and HDL cholesterol values obtained within the prior 12 months, gender, race, age, smoking status, systolic blood pressure and number of BP lowering medications (see the Framingham Risk Score Look-up Tables). Each table is organized based on values of age, SBP, HDL and total cholesterol, and the number in each cell is the points obtained from the combination of those four variables (plus gender). Each cell is then color coded to indicate the participant is eligible for SPRINT (green) or may be eligible based on additional points for being on BP meds and/or a smoker (yellow, blue and red). Clear cells are not eligible.

Using the Framingham Risk Score Look-up Tables, begin by finding the row that corresponds to the total cholesterol, then the sub-row that corresponds to the HDL-C, then the column that corresponds to age and finally the sub-column that corresponds to SBP. Note the point total (adjusted for race/ethnicity where indicated) and color of the appropriate cell and cross-reference with the table key to determine eligibility related to this criterion.

16. Are any of the responses from #10a-e, 11a-e, 14c or 15a positive or is the participant ≥ 75 years of age?
If the answer is no, the participant does not meet the SPRINT risk criteria and is ineligible.

SUMMARY OF ELIGIBILITY CRITERIA

1. The participant must be at least 50 years of age (Part A, item 6) to be eligible for trial. If participant is currently ineligible but will turn 50 before the end of recruitment, they may be rescreened at a later date.
2. The participant must satisfy all of the criteria in Part B to be eligible for the trial. If the participant is currently ineligible based on the combination BP medication number and sitting systolic BP, the participant may be rescreened after adjusting BP medications as described in section 8c above.
3. The participant must have elevated CVD risk based on age > 75 years, a positive history of clinical or subclinical CVD (indicated by a yes response to at least one item in question 10a-e or 11a-e), chronic kidney disease (indicated by a yes response to question 14c) or a Framingham risk score that exceeds gender specific cutpoints (indicated by a yes response to question 15b) to be eligible for the trial. Any one of these criteria is sufficient to establish eligibility based on elevated CVD risk.

PART D. GENERAL EXCLUSION CRITERIA
The participant's history and physical must be verified to ensure that none of the following exclusion criteria are present. A pertinent negative history for the criteria must be documented to confirm exclusion. If any of the following are present, the participant is not eligible for SPRINT. **Applicable inclusion & exclusion criteria should be carefully evaluated and clearly documented in the source documentation of the participant's study chart**.

17. Does the participant have a known secondary cause of hypertension that causes concern regarding the safety of the protocol?

If yes, then participant is ineligible. Secondary causes of hypertension include obstructive sleep apnea, primary hyperaldosteronism, renal artery stenosis, renal parenchymal disease , excess catecholamines, coarctation of the aorta, Cushing's syndrome, pheochromocytoma, some drugs (e.g. sympathomimetic agents), diet (excess sodium intake), excess erythropoietin and some endocrine disorders (e.g. hyperthyroidism). Participants with uncontrolled or untreated conditions listed should be excluded. If hyperparathyroidism, which is known to be associated with hypertension, is treated or controlled, then there may be no concern and it is an issue of investigator judgment as to exclusion of the participant.

18. Does the participant have an unacceptable level of proteinuria within the last 6 months?
If yes then participant is ineligible. If no information is available within the last 6 months a measure of protein excretion must be obtained during the screening process to rule out the presence of proteinuria. Every SPRINT participant MUST be documented during screening to have urinary protein excretion less than 1 gram per day or the equivalent as specified in the protocol.

Proteinuria in the following ranges (based on a measurement within the past 6 months) excludes the participant:

a. 24 hour urinary protein excretion > 1 g/day, or
b. If measurement (a) is not available, then 24 hour urinary albumin excretion > 600 mg/day, or
c. If measurements (a) and (b) are not available, then spot urine protein/creatinine ratio > 1 g/g creatinine, or
d. If measurements (a), (b), and (c) are not available, then spot urine albumin/creatinine ratio > 600 mg/g creatinine, or
e. If measurements (a), (b), (c), and (d) are not available, then urine dipstick ≥ 2+ protein

When two or more results (of the same level in the proteinuria hierarchy listed above) within the past 6 months are available to the investigator, then the eligibility status is based on the majority result, that is, if the majority of results within the past 6 months for that measurement support inclusion, then include; otherwise exclude. Alternatively, investigators may choose to obtain a higher ranked measure in the hierarchy if in doubt regarding eligibility.

The protein dipstick grading table below can assist you with interpreting dipstick values to help make eligibility assessments in the absence of the hierarchical urine protein measures outlined above. Designations of 2+ or higher or concentrations greater than 100 mg/dL make the participant ineligible.

**Designation**	**Approximate Concentration**
Trace	15 mg/dL
1+	30 mg/dL
2+	100 mg/dL
3+	300 mg/dL
4+	> 2000 mg/dL

19. Does the participant have diabetes mellitus? In SPRINT diabetes mellitus is determined as follows: Participants taking medications for the treatment of diabetes at any time in the last 12 months are excluded. Participants are also excluded if there is documentation of: FPG at or above 126 mg/dL, A1C ≥6.5 percent, a two-hour value in an OGTT (2-h PG) at or above 200 mg/dL, or a random plasma glucose concentration ≥200 mg/dL. The diagnosis of diabetes must be confirmed on a subsequent day by repeat measurement, repeating the same test for confirmation. However, if two different tests (e.g., FPG and HbA1C) are available and are concordant for the diagnosis of diabetes, additional testing is not needed. If two different tests are discordant, the test that is diagnostic of diabetes should be repeated to confirm the diagnosis.

This means that people with a history of diabetes (based either on a prior diagnosis or use of diabetes medications within the past 12 months) are ineligible for the trial. In the absence of a history of diabetes, one or more HbA1c, FPG, two-hour OGTT, or random plasma glucose test within the past 12 months can be used to establish the presence or absence of diabetes. If all qualifying test results are negative, the participant is potentially eligible for the trial. If any of the qualifying tests are positive, the diagnosis should be confirmed by having the same test repeated on a different day. If the positive result is confirmed, the participant is ineligible for the trial. If sufficient evidence is not available from the medical record to establish the presence or absence of diabetes, an HbA1c, FPG, two-hour OGTT, or random plasma glucose test should be obtained during screening.

20. Does the participant have a history of stroke?
If yes, then participant is ineligible.

Determining eligibility when vague neurological symptoms potentially attributable to stroke are reported but there are non-definitive imaging results and/or a non-definitive diagnosis:

- Clinical strokes are excluded. TIAs are not excluded. If there is no definitive diagnosis of stroke, it is up to the clinic PI to make a decision regarding eligibility. If the clinic PI is unable to make the decision, the next step is to seek advice at the CCN level. If a decision cannot be made at the CCN level, the RR&A Committee will review the case and make a determination of eligibility.

Determining eligibility when there is an old or age-indeterminate brain lesion consistent with a possible brain infarct on a brain imaging examination that was performed for a reason other than evaluation of stroke and there is no history of a stroke like syndrome:

- Persons with a history of stroke are excluded. According to the protocol, "stroke is GENERALLY defined as neurological deficit of cerebrovascular cause that persists beyond 24 hours or is interrupted by death within 24 hours (World Health Organization, 1978 Cerebrovascular Disorders (Offset Publications). Geneva: World Health Organization. ISBN 9241700432." Hence, an imaging test with a lesion consistent with a possible brain infarct in a person who does not have a history of a clinical scenario consistent with stroke does not meet the exclusion criterion, and such person can be included in SPRINT, if otherwise eligible.

21. Does the participant have a diagnosis of polycystic kidney disease or end-stage renal disease (ESRD)?
If yes then participant is ineligible

22. Does the participant have glomerulonephritis treated with immunosuppressive therapy?

If yes then participant is ineligible.

There is not a general exclusion related to the use of immunosuppressives. Rheumatic disorders treated with immunosuppressive therapy are not excluded.

23. Does the participant currently have symptomatic congestive heart failure (CHF), or a history of New York Heart Association (NYHA) Class III or IV CHF, or ejection fraction < 35% (by any method)?
If yes then participant is ineligible. Question the participant and review the records to ensure that there are no symptoms or evidence of New York HA Class III or IV CHF, as listed below, at the time of enrollment.

New York Heart Association CHF Classes:
Class I – No limitations of physical activity, ordinary activity does not cause symptoms.
Class II – Slight limitation of physical activity, ordinary physical activity results in symptoms
Class III – Marked limitation of physical activity, comfortable at rest but less than ordinary physical activity causes symptoms
Class IV – Symptoms at rest

24 and 25. Does the participant have any medical condition likely to limit survival to less than 3 years, or a cancer diagnosed and treated within the past two years that, in the judgment of clinical study staff, would compromise a participant's ability to comply with the protocol and complete the trial?
If yes, then the participant is ineligible. Participants with life-threatening diseases would likely lead to non-cardiovascular deaths early in the study and therefore affect the power of the study to answer the study questions. Non-melanoma skin cancer, early-stage prostate cancer, or localized breast cancer are examples of possible exceptions to this exclusion criteria based upon the clinician's assessment.

26. Is the participant living in the same household as an already randomized SPRINT participant?
If yes, the participant is ineligible. Due to concerns regarding possible intervention contamination and lack of statistical independence only one individual in a household can be randomized into SPRINT.

27. Has the participant had an organ transplant?
If yes, the participant is ineligible. Participants with organ transplants may be placed on multiple medications to suppress organ rejection, some of which may pose adverse interactions with the intervention.

28. Does the participant have an indication for any specific BP medication not currently taking without documented intolerance?
If yes, then participant is ineligible. If the participant has a compelling indication for an evidence-based therapy with blood pressure lowering effects without any documented contraindication or intolerance to the therapy, the participant should be considered for initiation of that therapy and may be rescreened for eligibility following stabilization on the specified therapy.

29. Has the participant had any cardiovascular event or interventional procedure, or been hospitalized for unstable angina within the last 3 months?

If yes then participant is ineligible. This would include MI and revascularization. A 3-month waiting period ensures clinical stability before initiating more aggressive treatment. If the answer is yes, participant can be rescreened after 3 months past the procedure or event if they meet all other criteria. (NOTE: An angiogram, is a diagnostic test rather than an interventional procedure, and therefore, by itself, does not render a participant ineligible).

30. Are there any factors likely to limit adherence to the trial interventions?
For example,

- Active alcohol or substance abuse within the last 12 months
- Plans to move outside the clinic catchment area in the next 2 years without the ability to transfer to another SPRINT site, or plans to be out of the study area for more than 3 months in the next year.
- Significant history of poor compliance with medications or attendance at clinic visits
- Significant concerns about participation in the study from spouse, significant other, or family members
- Lack of support from primary health care provider
- Residence too far from the study clinic site such that transportation is a barrier including persons who require transportation assistance provided by the SPRINT clinic funds for screening or randomization visits
- Residence in a nursing home. Persons residing in an assisted living or retirement community are eligible if they meet the other criteria.
- Clinical diagnosis of dementia, treatment with medications for dementia, or in the judgment of the clinician cognitively unable to follow the protocol
- Other medical, psychiatric, or behavioral factors that in the judgment of the Principal Investigator may interfere with study participation or the ability to follow the intervention protocol

If the participant has any of these issues, please check (yes) and exclude the participant. The importance of adherence to the protocol is crucial to the success of SPRINT. A participant with any of the factors listed above may be eligible by all other criteria and will sign a consent form but may also be likely to subsequently refuse to take the prescribed medications or attend clinic visits. It is in the participants' best interest as well as the study's that you evaluate their long-term commitment to carrying out the protocol.

Support from the Primary Care Physician (PCP) can also be very important. Lack of support can be quite adverse to long-term retention and adherence. Encourage potential participants to have discussions with their PCPs prior to obtaining consent. Give participants adequate time to have these discussions, provide informational materials to assist in this process, and document the process in your source notes. Communicate with the PCP early and often during the trial, in accordance with permissions provided by the participants. Primarily out of respect for participant autonomy, but also due to logistical concerns, we are not adopting a requirement for PCP explicit and documented approval prior to randomization; however, evidence of lack of support from the PCP should be considered by clinic staff and investigators when deciding whether to enroll a potentially eligible person during the screening process.

31. Is the participant currently participating in another clinical trial?
If yes, the participant may or may not be eligible. Participants cannot be enrolled into SPRINT if already participating in another interventional clinical trial. Observational studies are acceptable. The screenee must wait until the completion of his or her activities in the other interventional clinical trial prior to the SPRINT randomization visit to participate in SPRINT.

32. Has the participant had > 10% unexplained weight loss in the last 6 months?
If yes, the participant is ineligible. This participant could conceivably be rescreened once participant's weight has stabilized if they meet all other criteria.

33. Is the participant pregnant, trying to become pregnant or of child bearing potential and not practicing birth control?
If yes the participant is ineligible due to concerns regarding the effectiveness and safety of the intervention in pregnancy.

PART E. INFORMED CONSENT
34. Has informed consent been obtained from the participant?
If no then participant is ineligible. The full trial informed consent **MUST** be signed prior to randomization into SPRINT.

PART F. CONTACT INFORMATION
Collect the participant's name and all contact information. This is critical information to obtain for future contact with the participant.

3.a.2 Tracking of Participants: Assignment of IDs

Participant IDs are eight digit numbers ranging from 10000001 – 99999999. Participant IDs are generated by the CC and pre-printed on the inclusion/exclusion form. When the inclusion / exclusion form is data entered, this ID is scanned and will follow the participant throughout the screening, randomization and follow-up visits and will be pre-printed on the rest of the study visits and forms. The IDs do not contain any encoded information about the clinical site or network and will not change for participants that transfer from one clinical site to another during the study. The study will not use acrostics, but participant names will be pre-printed on Case Report forms to assist clinics in organizing and tracking forms from visit to visit. Study forms that are sent outside the clinical sites (ie, Lab) <u>will not</u> have the participant name pre-printed on the form.

3.a.3 Existing populations in the clinical center practices
Medical record searches or reviews of existing data bases can be done initially by setting up the searches using the variables that match the final list of the inclusion/exclusion criteria. Additional "hand searches" may be necessary using the remaining inclusion/exclusion criteria not already part of the data base but which is part of the participant's existing clinical record. It is expected that all or most all of the inclusion/exclusion criteria will be available in the medical record. Medical record review will be used to begin the process of completing the Inclusion/Exclusion Summary Form.

3.a.4 The Screening Visit

The following are key elements of the screening visit and are outlined in the study assessments and procedures below:

Screening Visit(s)
1. Print out a copy of the Inclusion/Exclusion Form.
2. Verify participant's interest in study. Participants will be scheduled to attend the initial screening clinic visit. The participant will be instructed to fast for the visit, if applicable, and should bring current medications, and support person or significant other to the visit.

3. Review the study protocol with the potential participant. Obtain their written consent (including the Main Trial, MIND, MRI, genetics and applicable ancillary studies) and HIPAA authorization.
4. Review all data collected during the prescreening process with the participant.
5. Continue collection of screening information, including such items as contact information, additional eligibility information including BP measurement, height, weight, demographics, concomitant medications, social and medical history.
6. Determine eligibility status for SPRINT.
    a. Do they meet the general inclusion criteria: age, ≥ 130 SBP ≤ 180 mmHg on an appropriate number of antihypertensive agents:
        SBP: 130 – 180 mmHg on 0 or 1 medication
        SBP: 130 – 170 mmHg on up to 2 medications
        SBP: 130 – 160 mmHg on up to 3 medications
        SBP: 130 – 150 mm Hg on up to 4 medications
    b. Is their eGFR in the eligible range: ≥20 ml/min/1.73m^2 eGFR ≤59?
    c. Is their Framingham Risk Score point total ≥ 13 (men); ≥ 16 (women) or are they 75 years of age or older?
    d. Does the participant have a positive history of clinical or subclinical cardiovascular disease (CVD) within the past 2 years?
7. If the answer to #6a and at least one of 6b-d are positive the participant is provisionally eligible for enrollment. If the answer to any of the #6a-d are "no", the participant may be provisionally or permanently ineligible.
8. If provisionally eligible, explain to the potential participant the additional screening process and procedures that may be needed in the future to re-assess eligibility. If ineligible, thank the participant for his/her time. Refer to Tracking Ineligible Screenees (See MOP Section 3.a.6 below).
9. Perform phlebotomy to obtain blood samples, process and ship to the Local Lab for measurement of glucose, chemistry and lipid profiles, to determine eligibility criteria. Measurements of these values (glucose, total cholesterol and HDL) recorded in the clinical record during the previous year may be used for eligibility purposes. Measures of creatinine levels must be within 6 months of the anticipated date of randomization in order to determine eligibility. If no glucose or creatinine level are available, they may be drawn at the screening visit..
10. A spot urine sample dipstick test for proteinuria can be performed first to detect gross proteinuria. If negative and needed for eligibility a urine sample will be obtained for measurement of proteinuria. SPRINT does not reimburse for local tests done to determine eligibility
11. Results from an ECG done during the previous 2 years may be used for eligibility purposes. An ECG may be obtained and reviewed locally as needed to determine eligibility criteria.
12. Make an appointment for the next visit. Instruct participant that the baseline visit will take 2-3 hours or longer. Remind participants to bring all their medications to each visit.
13. Complete the **Inclusion/Exclusion Summary Form** and enter data as required.
14. It is required that this form be completed and data entered prior to the participant returning for the baseline visit.
15. Document all applicable data within source documentation.
16. If eligibility criteria are not yet complete (awaiting lab values or medical records), schedule the baseline visit only when adequate documentation of criteria is available. **NOTE:** For all potential participants who have an in person clinic visit for screening (regardless of eligibility), the minimum data collection and data entry for screening data are items in Part A and until ineligibility is established on the **Inclusion/Exclusion Summary Form.** If the screenee is eligible at that point, continue to use the screening forms until the screenee is found to be eligible or ineligible and enter all data collected.

3.a.5 Re-Screening Previously Screened Participants

If a participant has been screened previously and found to be ineligible, re-screening may be performed if in the judgment of the clinical site, the participant may meet the eligibility criteria at a later date. The participant will retain the same ID number that was initially assigned. No new ID number should be assigned unless the Inclusion/Exclusion Summary Form is damaged or lost. In instances where a participant is either re-screened or the screening process has been prolonged (i.e., more than 1 month), all eligibility criteria should be reviewed and updated if necessary. During this review, particular care should be taken to discern if BP therapy has changed or if any new CVD events or procedures occurred since the time of initial screening. Remember that no new CVD events or procedures should have occurred within 3 months of randomization.

It is acceptable and appropriate for sites to manage blood pressure during the screening process to bring it within range for the study. Clinics should do what is reasonable with consideration of participant and staff burden. It should be a rare event to screen anyone more than 3 times. Clinics should not screen an individual more than 4 times in a two month period. If the participant is not eligible within the two month period, they should not be contacted for 90 days.

3.a.6 Tracking Ineligible Screenees

The **Inclusion/Exclusion Summary Form** is to be completed and data entered in its entirety for participants who are enrolled in SPRINT. It has been designed to serve as a worksheet to keep track of where potential participants are in the screening process. It will also serve to collect limited data on individuals who are screened for the study, but are not enrolled. These data will provide some characteristics of the people who were NOT eligible for the trial, a requirement for describing the trial to other researchers and for journal publications. For the **Inclusion/Exclusion Summary Form** for ineligible screenees, sites will be instructed to:

- Complete Part A items on the **Inclusion/Exclusion Summary Form** for every participant who comes in to the clinic for screening.
- If the participant is eligible through Part A, continue until form is either complete or until ineligibility is found.
- All Inclusion/Exclusion Summary Forms (partially or fully) completed should be data entered at the clinical sites.

Men (require 13 points for 10 year CVD risk > 15%; D'Agostino et al, Circulation 2008). Add 1 point for African American men and subtract 1 point for Asian men. Also add 4 points for smokers and add 2 points for use of 1-4 antihypertensive medications.

Total Cholesterol mg/dl	HDL mg/dl	Age:	50-54				55-59				60-64				65-69				70-74				75+			
		SBP:	130-139	140-149	150-159	160+	130-139	140-149	150-159	160+	130-139	140-149	150-159	160+	130-139	140-149	150-159	160+	130-139	140-149	150-159	160+	130-139	140-149	150-159	160+
<160	60+		7	8	8	9	9	10	10	11	10	11	11	12	11	12	12	13	13	14	14	15	14	15	15	16
	50-59		8	9	9	10	10	11	11	12	11	12	12	13	12	13	13	14	14	15	15	16	15	16	16	17
	45-49		9	10	10	11	11	12	12	13	12	13	13	14	13	14	14	15	15	16	16	17	16	17	17	18
	35-44		10	11	11	12	12	13	13	14	13	14	14	15	14	15	15	16	16	17	17	18	17	18	18	19
	<35		11	12	12	13	13	14	14	15	14	15	15	16	15	16	16	17	17	18	18	19	18	19	19	20
160-199	60+		8	9	9	10	10	11	11	12	11	12	12	13	12	13	13	14	14	15	15	16	15	16	16	17
	50-59		9	10	10	11	11	12	12	13	12	13	13	14	13	14	14	15	15	16	16	17	16	17	17	18
	45-49		10	11	11	12	12	13	13	14	13	14	14	15	14	15	15	16	16	17	17	18	17	18	18	19
	35-44		11	12	12	13	13	14	14	15	14	15	15	16	15	16	16	17	17	18	18	19	18	19	19	20
	<35		12	13	13	14	14	15	15	16	15	16	16	17	16	17	17	18	18	19	19	20	19	20	20	21
200-239	60+		9	10	10	11	11	12	12	13	12	13	13	14	13	14	14	15	15	16	16	17	16	17	17	18
	50-59		10	11	11	12	12	13	13	14	13	14	14	15	14	15	15	16	16	17	17	18	17	18	18	19
	45-49		11	12	12	13	13	14	14	15	14	15	15	16	15	16	16	17	17	18	18	19	18	19	19	20
	35-44		12	13	13	14	14	15	15	16	15	16	16	17	16	17	17	18	18	19	19	20	19	20	20	21
	<35		13	14	14	15	15	16	16	17	16	17	17	18	17	18	18	19	19	20	20	21	20	21	21	22
240-279	60+		10	11	11	12	12	13	13	14	13	14	14	15	14	15	15	16	16	17	17	18	17	18	18	19
	50-59		11	12	12	13	13	14	14	15	14	15	15	16	15	16	16	17	17	18	18	19	18	19	19	20
	45-49		12	13	13	14	14	15	15	16	15	16	16	17	16	17	17	18	18	19	19	20	19	20	20	21
	35-44		13	14	14	15	15	16	16	17	16	17	17	18	17	18	18	19	19	20	20	21	20	21	21	22
	<35		14	15	15	16	16	17	17	18	17	18	18	19	18	19	19	20	20	21	21	22	21	22	22	23
280+	60+		11	12	12	13	13	14	14	15	14	15	15	16	15	16	16	17	17	18	18	19	18	19	19	20
	50-59		12	13	13	14	14	15	15	16	15	16	16	17	16	17	17	18	18	19	19	20	19	20	20	21
	45-49		13	14	14	15	15	16	16	17	16	17	17	18	17	18	18	19	19	20	20	21	20	21	21	22
	35-44		14	15	15	16	16	17	17	18	17	18	18	19	18	19	19	20	20	21	21	22	21	22	22	23
	<35		15	16	16	17	17	18	18	19	18	19	19	20	19	20	20	21	21	22	22	23	22	23	23	24

Qualified	Qualified if on 1-4 meds or a smoker	Qualified if a smoker	Qualified if both on 1-3 meds AND a smoker

Women (require 16 points for 10 year CVD risk > 15%; D'Agostino et al, Circulation 2008). Add 1 point for African American women and subtract 1 point for Asian women. Also add 3 points for smokers and add 2 points for use of 1-4 antihypertensive medications (add 1 additional point for hypertensive treatment if SBP is between 140 and 149 mm Hg, inclusive).

Total Cholesterol mg/dl	HDL mg/dl	Age:	50-54				55-59				60-64				65-69				70-74				75+			
		SBP:	130-139	140-149	150-159	160+	130-139	140-149	150-159	160+	130-139	140-149	150-159	160+	130-139	140-149	150-159	160+	130-139	140-149	150-159	160+	130-139	140-149	150-159	160+
<160	60+		6	7	9	10	7	8	10	11	8	9	11	12	9	10	12	13	10	11	13	14	11	12	14	15
	50-59		7	8	10	11	8	9	11	12	9	10	12	13	10	11	13	14	11	12	14	15	12	13	15	16
	45-49		8	9	11	12	9	10	12	13	10	11	13	14	11	12	14	15	12	13	15	16	13	14	16	17
	35-44		9	10	12	13	10	11	13	14	11	12	14	15	12	13	15	16	13	14	16	17	14	15	17	18
	<35		10	11	13	14	11	12	14	15	12	13	15	16	13	14	16	17	14	15	17	18	15	16	18	19
160-199	60+		7	8	10	11	8	9	11	12	9	10	12	13	10	11	13	14	11	12	14	15	12	13	15	16
	50-59		8	9	11	12	9	10	12	13	10	11	13	14	11	12	14	15	12	13	15	16	13	14	16	17
	45-49		9	10	12	13	10	11	13	14	11	12	14	15	12	13	15	16	13	14	16	17	14	15	17	18
	35-44		10	11	13	14	11	12	14	15	12	13	15	16	13	14	16	17	14	15	17	18	15	16	18	19
	<35		11	12	14	15	12	13	15	16	13	14	16	17	14	15	17	18	15	16	18	19	16	17	19	20
200-239	60+		9	10	12	13	10	11	13	14	11	12	14	15	12	13	15	16	13	14	16	17	14	15	17	18
	50-59		10	11	13	14	11	12	14	15	12	13	15	16	13	14	16	17	14	15	17	18	15	16	18	19
	45-49		11	12	14	15	12	13	15	16	13	14	16	17	14	15	17	18	15	16	18	19	16	17	19	20
	35-44		12	13	15	16	13	14	16	17	14	15	17	18	15	16	18	19	16	17	19	20	17	18	20	21
	<35		13	14	16	17	14	15	17	18	15	16	18	19	16	17	19	20	17	18	20	21	18	19	21	22
240-279	60+		10	11	13	14	11	12	14	15	12	13	15	16	13	14	16	17	14	15	17	18	15	16	18	19
	50-59		11	12	14	15	12	13	15	16	13	14	16	17	14	15	17	18	15	16	18	19	16	17	19	20
	45-49		12	13	15	16	13	14	16	17	14	15	17	18	15	16	18	19	16	17	19	20	17	18	20	21
	35-44		13	14	16	17	14	15	17	18	15	16	18	19	16	17	19	20	17	18	20	21	18	19	21	22
	<35		14	15	17	18	15	16	18	19	16	17	19	20	17	18	20	21	18	19	21	22	19	20	22	23
280+	60+		11	12	14	15	12	13	15	16	13	14	16	17	14	15	17	18	15	16	18	19	16	17	19	20
	50-59		12	13	15	16	13	14	16	17	14	15	17	18	15	16	18	19	16	17	19	20	17	18	20	21
	45-49		13	14	16	17	14	15	17	18	15	16	18	19	16	17	19	20	17	18	20	21	18	19	21	22
	35-44		14	15	17	18	15	16	18	19	16	17	19	20	17	18	20	21	18	19	21	22	19	20	22	23
	<35		15	16	18	19	16	17	19	20	17	18	20	21	18	19	21	22	19	20	22	23	20	21	23	24

Not Qualified	Qualified	Qualified if on 1-4 meds or a smoker	Qualified if a smoker	Qualified if both on 1-3 meds AND a smoker

Table 3a3. List of FDA approved hypertension medications and dosages (doses less than the below do not count as antihypertensive medications)

Class(s)	Agent(s)	Usual Dose Range in mg/day
ACE Inhibitors	benazepril (Lotensin)	10 - 40
ACE Inhibitors	captopril (Capoten)	25 - 100
ACE Inhibitors	enalapril (Vasotec)	5 - 40
ACE Inhibitors	fosinopril (Monopril)	10 - 80
ACE Inhibitors	lisinopril (Prinivil; Zestril)	10 - 40
ACE Inhibitors	moexipril (Univasc)	7.5 - 30
ACE Inhibitors	perindopril (Aceon)	4 - 8
ACE Inhibitors	quinapril (Accupril)	10 - 80
ACE Inhibitors	ramipril (Altace)	2.5 - 20
ACE Inhibitors	trandolapril (Mavik)	1 - 8
ACEI + CCB	amlodipine/benzapril hydrochloride (Lotrel)	2.5-10/10-40
ACEI + CCB	enalapril maleate/felodipine (Lexxel)	5-40/2.5-20
ACEI + CCB	trandolapril/verapamil (Tarka)	1 - 8 / 120 - 480
ACEI + Diuretic	benazepril/hctz (Lotensin HCT)	10 - 40 / 12.5 - 50
ACEI + Diuretic	captopril/hctz (Capozide)	25 - 100 / 12.5 - 50
ACEI + Diuretic	enalapril/hctz (Vaseretic)	5 - 40 / 12.5 - 50
ACEI + Diuretic	fosinopril/hctz (Monopril HCT)	10 - 80 / 12.5 - 50
ACEI + Diuretic	lisinopril/hctz (Prinzide; Zestoretic)	10 - 40 / 12.5 - 50
ACEI + Diuretic	moexipril HCl/hctz (Uniretic)	7.5 - 30 / 12.5 - 50
ACEI + Diuretic	quinapril/hctz (Accuretic)	10 - 80 / 12.5 - 50
Aldosterone receptor blockers	eplerenone (Inspra)	50 - 100
Aldosterone receptor blockers	spironolactone (Aldactone)	25 - 50
Alpha 1-blockers	doxazosin (Cardura)	1 - 16
Alpha 1-blockers	phenoxybenzamine (Dibenzyline)	40 - 120
Alpha 1-blockers	prazosin (Minipress)	2 - 20
Alpha 1-blockers	terazosin (Hytrin)	1 - 20
Angiotensin II Antagonists	Azilsartan (Edarbi)	40-80

Angiotensin II Antagonists	candesartan (Atacand)	8 - 32
Angiotensin II Antagonists	eprosartan (Treveten)	400 - 800
Angiotensin II Antagonists	irbesartan (Avapro)	150 - 300
Angiotensin II Antagonists	losartan (Cozaar)	25 - 100
Angiotensin II Antagonists	olmesartan (Benicar)	20 - 40
Angiotensin II Antagonists	telmisartan (Micardis)	20 - 80
Angiotensin II Antagonists	valsartan (Diovan)	80 - 320
ARB + Diuretic	candesartan/hctz (Atacand HCT)	8 - 32 / 12.5 - 50
ARB + Diuretic	eprosartan mesylate/hctz (Teveten HCT)	400 - 800 / 12.5 - 50
ARB + Diuretic	irbesartan/hctz (Avalide)	150 - 300 / 12.5 - 50
ARB + Diuretic	losartan potassim/hydrochlorothiazide (Hyzaar)	25 - 100 / 12.5 - 50
ARB + Diuretic	olmesartan/hctz (Benicar HCT)	20 - 40 / 12.5 - 50
ARB + Diuretic	telmisartan/hctz (Micardis HCT)	20 - 80 / 12.5 - 50
ARB + Diuretic	valsartan/hydrochlorothiazide (Diovan HCT)	80 - 320 / 12.5 - 50
Beta Blockers with intrinsic sympathomimetic (ISA) activity	acebutolol (Secrtral)	200 - 800
Beta Blockers with intrinsic sympathomimetic (ISA) activity	penbutolol (Levatol)	10 - 40
Beta Blockers with intrinsic sympathomimetic (ISA) activity	pindolol	10 - 40
Beta-blocker + Diuretic	atenolol/chlorthalidone (Tenoretic)	25-100 / 12.5 - 25
Beta-blocker + Diuretic	bisoprolol fumarate/hctz (Ziac)	2.5 - 10 / 12.5 - 50
Beta-blocker + Diuretic	metoprolol tartrate/hctz (Lopressor HCT)	50 - 200 / 12.5- 50
Beta-blocker + Diuretic	nadolol/bendroflumethiazide (Corzide)	40 - 120 / 5
Beta-blocker + Diuretic	propranolol LA/hctz (Inderide)	40 - 160 / 12.5 - 50
Beta-blocker + Diuretic	timolol maleate/hctz (Timolide)	20 - 40 / 12.5 - 50
Central Alpha2-agonists and other centrally acting	clonidine (Catapres)	0.1 - 0.8
Central Alpha2-agonists and other centrally acting	clonidine patch (Catapres-TTS)	0.1 - 0.6
Central Alpha2-agonists and other centrally acting	guanfacine (Tenex)	0.5 - 2
Central Alpha2-agonists and other centrally acting	methyldopa (Aldomet)	250 - 1000

Central Alpha2-agonists and other centrally acting	reserpine	0.05 - 0.25
Central Alpha2-agonists and other centrally acting	guanabenz	8 - 64
Centrally acting drug + Diuretic	clonidine/chlorthalidone (Clorpres)	0.1 - 0.8 / 12.5 - 25
Centrally acting drug + Diuretic	methyldopa/hctz (Aldoril)	250 - 1000 / 12.5 - 50
Combined alpha- and beta-blockers	carvedilol (Coreg)	12.5 - 50
Combined alpha- and beta-blockers	labetalol (Normodyne; Trandate)	200 - 800
DHP CCB + Angiontensin II agonist	amlodipine/olmesartan (Azor)	2.5 - 10 / 20 - 40
DHP CCB + Angiontensin II agonist	amlodipine/telmisartan (Twynsta)	2.5 - 10 / 20 - 80
DHP CCB + Angiontensin II agonist	amlodipine/valsartan (Exforge)	2.5 - 10 / 80 - 320
DHP CCB + Angiontensin II agonist + Diuretic	amlodipine/olmesartan/hydrochlorothiazide (Tribenzor)	2.5 - 10 / 20 - 40/ 12.5 - 50
DHP CCB + Angiontensin II agonist + Diuretic	amlodipine/valsartan/hydrochlorothiazide (Exforge HCT)	2.5 - 10 / 80 - 320 / 12.5 - 50
Dihydropyridine Calcium Channel Blockers	amlodipine (Norvasc)	2.5 - 10
Dihydropyridine Calcium Channel Blockers	felodipine (Plendil)	2.5 - 20
Dihydropyridine Calcium Channel Blockers	isradipine (Dynacirc)	2.5 - 10
Dihydropyridine Calcium Channel Blockers	isradipine SR (Dynacirc CR)	5 - 20
Dihydropyridine Calcium Channel Blockers	nicardipine	60 - 120
Dihydropyridine Calcium Channel Blockers	nicardipine sustained release (Cardene SR)	60 - 120
Dihydropyridine Calcium Channel Blockers	nifedipine (Adalat; Procardia)	30 - 60
Dihydropyridine Calcium Channel Blockers	nifedipine CR/ER (Adalat CC; Procardia XL)	30 - 60
Dihydropyridine Calcium Channel Blockers	nisoldipine (Sular)	10 - 40
Direct Vasodilators	hydralazine (Apresoline)	25 - 200

Direct Vasodilators	minoxidil (Loniten)	2.5 - 100
Direct Vasodilators + Diuretic	hydralazine/hctz (Hydra-zide)	25 - 200 / 12.5 - 50
Diuretic + Diuretic	amiloride hcl/hctz (Moduretic)	5 - 10 / 12.5 - 50
Diuretic + Diuretic	spironolactone/hctz (Aldactone)	25 - 50 / 12.5 - 50
Diuretic + Diuretic	triamterene/hctz (Dyazide; Maxzide)	50 - 100 / 12.5 - 50
k-sparing	amiloride (Midamor)	5 - 10
k-sparing	triamterene (Dyrenium)	50 - 100
loop	bumetanide	0.5 - 2
loop	furosemide (Lasix)	40 - 320
loop	torsemide (Demadex)	2.5 - 10
Non-dihydropyridine Calcim Channel Blockers	diltiazem extended release (Cardizem CD; Cartia XT; Dilacor XR; Dilt-CD; Diltia XT; Diltzac; Taztia XT; Tiazac; Cardizem LA)	120 - 540
Non-dihydropyridine Calcim Channel Blockers	verapamil (Coer; Covar HS; Verelan PM)	120 - 360
Non-dihydropyridine Calcim Channel Blockers	verapamil immediate release (Calan; Isoptin)	80 - 320
Non-dihydropyridine Calcim Channel Blockers	verapamil long acting (Calan SR; Isoptin SR)	120 - 480
Non-ISA Beta Blockers	atenolol (Tenormin)	25 - 100
Non-ISA Beta Blockers	bisoprolol (Zebeta)	2.5 - 10
Non-ISA Beta Blockers	metoprolol (Lopressor)	50 - 200
Non-ISA Beta Blockers	metoprolol extended release (Toprol XL)	50 - 200
Non-ISA Beta Blockers	nadolol (Corgard)	40 - 120
Non-ISA Beta Blockers	nebivolol (Bystolic)	5 - 40
Non-ISA Beta Blockers	propranolol (Inderal)	40 - 160
Non-ISA Beta Blockers	propranolol long-acting (Inderal LA)	60 - 240
Non-ISA Beta Blockers	timolol (Blocadren)	20 - 40
Non-ISA Beta Blockers + diuretic	nadol/bendroflumethiazide	40 - 120 / 5
Renin Inhibitor	Aliskiren (Tekturna)	150 - 300
Renin Inhibitor + Angiontensin II agonists	aliskiren/valsartan (Valturna)	150 - 300 / 80 - 320
Renin Inhibitor + DHP CCB	aliskiren/amlodipine (Tekamlo)	150 - 300 / 2.5 - 10
Renin Inhibitor + DHP CCB + diuretic	aliskiren/amlodipine/hydroclorothiazide (Amturnide)	150 - 300 / 2.5 - 10 / 12.5 - 50

Renin Inhibitor + diuretic	aliskiren/hydrochlorothiazide (Tekturna HCT)	150 - 300 / 12.5 - 50
Thiazide Diuretic	chlorothiazide (Diuril)	125 - 500
Thiazide Diuretic	chlorthalidone	12.5 - 25 mg
Thiazide Diuretic	hydrochlorothiazide (HydroDIURIL; Microzide)	12.5 - 50
Thiazide Diuretic	indapamide (Lozol)	1.25 - 5
Thiazide Diuretic	methyclothiazide (Enduron)	2.5 - 5
Thiazide Diuretic	metolazone (Mykrox)	0.5 - 1
Thiazide Diuretic	metolazone (Zaroxolyn)	2.5 - 5

Chapter 3.b Baseline/Randomization Visit Procedures

The purpose of this section of the MOP is to lay out the required sequence of the baseline (randomization) visit. To facilitate planning for both the participant and staff, inform participant that the anticipated length of the baseline (RZ) visit is about two to three hours. One short rest period is suggested following the blood draw. Additional rest periods may be needed between other exam components, especially if the participant is selected for the MIND Extended Battery.

The screening visit and the baseline/randomization visit should be conducted on different days. Part of the reason for having separate screening and baseline/randomization visits is to assess the participant's willingness to return to the clinic for study visits (i.e., an additional adherence/retention screen). SPRINT is a long-term study with many visits over the years. Good adherence begins with randomizing good adherers. Recognizing that there may be extenuating circumstances when the visits need to occur on the same day, please keep in mind the following:

- Some IRBs discourage or prohibit this practice. Sites should contact their local IRB prior to proceeding.
- Be sure to plan sufficient time between the two visits for a thorough consent process with the participant.
- You must get a second blood pressure measurement at baseline (i.e., you cannot copy the screening blood pressure onto the baseline forms.

There may also be situations when the baseline visit needs to split into two separate visits. This should be a rare occurrence, as it imposes burden to have a participant return for another visit. Sites can, however, be flexible and decide locally to conduct the second visit if necessary. Sites should do this only when absolutely necessary and if it is allowed by their local IRB. In these situations, the two visits should be as close together as possible, and it is best to hold randomization until the beginning of the second visit. The Coordinating Center does not need to be notified when the baseline visit is divided into two separate visits.

Prior to beginning the randomization visit, you should review the participant's consent and eligibility. The extent of the eligibility review will depend on the interval between the last screening visit and the randomization visit, as described below.

Table 3b.1. Eligibility checks required based on interval between last screening visit and randomization.

Item	Interval		
	0 – 6 Days	7 – 30 Days	30 Days
Intervening hospitalization?	✓	✓	
Intervening ED visit?	✓	✓	
Change in BP Medications?		✓	
Rescreen			✓

If it has been more than 30 days since the last screening visit, review the **Inclusion/Exclusion form** with the participant, checking carefully for any changes that may have occurred in the interval since the last screening visit. Confirm that <u>all</u> inclusion/exclusion criteria are satisfied. If circumstances have changed making the participant permanently ineligible, thank them for their time and dismiss them from the clinic. If an event has occurred to make the participant currently ineligible but potentially eligible at a later date (e.g., MI), talk with them about possible

rescreening in the future. If the participant is still eligible, continue with baseline/RZ visit procedures.

If it has been 30 days or less since the last screening visit, it is not necessary to review the full inclusion/exclusion form prior to randomization. However, you should always check to see if the participant has been hospitalized or treated in the emergency department (ED) since the last screening visit, and if so, determine whether that hospitalization or ED visit would change their eligibility (for example, hospitalization for stroke would make them permanently ineligible). Also, if it has been 7-30 days since the last screening visit, you should check to see if the participant's prescribed blood pressure medications have been changed during the interval. If therapy changes have been made, you should check their blood pressure and update the screening form (including visit date) to confirm that they are still eligible based on the combination of number of medications and current blood pressure. If their blood pressure therapy has not changed, it is not necessary to check their blood pressure to confirm eligibility. However, blood pressure should be measured and recorded for baseline data if the participant is otherwise eligible to be randomized.

The following items should be conducted PRIOR to the baseline/randomization (RZ) visit:

- Verify that all data collected during the screening visit have been reviewed by SPRINT investigator and the data have been entered.
- Provide written instructions about overnight fast (8 hours) and a reminder to take current BP medication(s) morning of visit.
- Call participant the evening before the visit to review questions or concerns and discuss the visit procedures. Remind the participant to bring all their medications and a support person or significant other with them to the visit. If you are not providing a snack, remind the participant to bring a snack. If unable to contact by telephone, leave a short voice mail message about visit procedures.

Recommended Sequence of Baseline (Randomization) Visit
Greeting
Height, weight
Review eligibility
Blood pressure
Blood draw, urine collection
ECG
4-meter walk *
HIPAA/medical release
Snack, rest, self-administered forms
**Randomization**
MIND Screening Battery
MIND Extended Battery *
MRI Consent *
Rest, My Health Form
Women's Health or Men's Health *
Record medications and physical exam
Assess blood pressure therapy
Adherence scales
Initiate/prescribe study medications
Schedule 1-month visit
Exit

* Shaded rows are subsets only.

The suggested sequencing of the RZ visit follows:

1. When the participant arrives for the visit, greet him/her, and confirm that they are fasting and have taken their current BP medications as prescribed. Take the participant to an examining room.
2. Obtain height and weight measurements. Record the height and weight on the **Baseline Medications and Physical Exam Form.**
3. Review the **Inclusion/Exclusion form** with the participant, checking carefully for any eligibility changes that may have occurred since the screening visit. Confirm that all inclusion/exclusion criteria are satisfied. If circumstances have changed making the participant permanently ineligible, thank them for their time and dismiss them from the clinic. If an event has occurred to make the participant currently ineligible but potentially eligible at a later date (e.g., MI), talk with them about possible rescreening in the future. If the participant is still eligible, continue with baseline/RZ visit procedures;
4. Collect all blood pressure related information:
   Confirm prior to taking the BP measurements that participant has taken BP medication as prescribed prior to coming to the visit. Obtain and record both sitting and standing blood pressure and pulse on the **Baseline BP Management Form.** If it was necessary to check the participant's blood pressure measurements during review of eligibility, a new set of measurements should be obtained for the randomization visit. It is not necessary for the randomization visit measurements to fall within eligibility criteria for the participant to be randomized.

5. Obtain fasting blood, urine, and genomic material for analysis and storage at the SPRINT central Laboratory.
6. Obtain fasting ECG. Three ECG tracings will be obtained and transmitted to the SPRINT ECG Reading Center;
7. For those participants age ≥75 yrs, conduct and record the time required to complete a 4 meter walk test on the **Baseline Medications and Physical Exam Form**;
8. If not already done, review HIPAA authorization and have the participant sign a Medical Release Form;
9. The participant should be provided with a snack and a short rest period during which they are asked to complete the **Self-Administered Baseline History Form** and the **Participant Contact Information** Form;
10. While the participant is eating their snack and filling out forms, take the participant's **Inclusion/Exclusion Form** and signed consent to a computer with internet access and log into the SPRINT website. Go to the clinical data entry section of the website and scan the barcode from the **Inclusion/Exclusion Form**. You will see a prompt asking whether you want to edit the **Inclusion/Exclusion Form**. If the participant's information has changed since the screening visit, select yes to update and save the form; otherwise you can select no and proceed directly to the participant's main data entry page. This page will display the participant's eligibility status and, for eligible participants, a randomization link;
11. **Click on the randomization link to begin the randomization process. DO NOT RANDOMIZE the participant until they are in the clinic with you!** Before the participant can be randomized, you must indicate that the baseline **BP Management Form** has been completed and that the participant has signed a medical release. You will also be prompted to double data enter the participant's responses to the layered consent questions. Once these questions have been answered, you will see a pop up message that indicates the participant has been successfully randomized and returned to the participant's main data entry page. This page will now indicate the treatment group assignment for the participant and whether they have been selected any sub-studies. You will also find links to print the participant's randomization assignment with guide on initializing therapy, all applicable sub-study assignments, target visit dates and the remainder of the baseline visit forms. It is recommended that the participant's treatment assignment and visit schedule be printed out and filed in their research record;
12. **Begin the MIND Measurements.** All participants will receive the Screening Battery (**Logical Memory Test, MoCA, and Digit Symbol Coding Test)**. A subset will be identified for the MIND Extended Battery so be sure to carefully review the information sheet that was provided at the time of randomization. If the participant has been selected for this sub-study, these forms will print automatically as part of the second baseline visit form set (RZ2). This subset will receive the **Hopkins Verbal Learning Test (HVLT), Trial Making Tests Parts A and B, Boston Naming Test, Modified Rey-Osterrieth Complex Figure, Category Fluency Animals, and Digit Span Forward and Backward)**;
13. Participants from selected sites that receive the MIND Extended Battery may also be asked to participate in the MRI substudy. If the clinic is within ~1.5 hours of a designated SPRINT MRI Scanning Facility, participants will be asked to consent on the **MRI Consent Form** (separate consent form) and screened for eligibility (see MRI chapter). The clinic will schedule the MRI (to be completed within 30 days of the baseline visit) and provide directions and instructions to the participant;
14. Participants may be given a 5 minute rest following the MIND exam, followed by having them fill out the **My Health Form**, which is self-administered and includes three health related quality of life instruments. All responses for self-administered forms should be

reviewed for completeness. ****Review the participant's response to question 9, page 5 (Thoughts that you would be better off dead or hurting yourself in some way). Instructions for dealing with a participant at risk of harming themselves are provided in the Safety chapter).

15. If participant was selected for the Health Related Quality of Life (HRQL) sub-study at the time of randomization, they should also complete the **Female Health** or **Male Health** questionnaires as appropriate at this time;
16. While the participant is completing the HRQL self-administered forms, review the **Participant Contact Form** and the **Self-Administered Baseline History Form** for completeness;
17. .Record current prescription medications (excluding BP medications) on the **Baseline Medications and Physical Exam Form**;
18. A limited physical exam for safety assessment (components selected at the discretion of the investigator) is expected but not data entered and will be kept in the participant chart. Document completion of physical exam on the **Baseline Medications and Physical Exam Form;**
19. Assessment of BP and adjustment of therapy:
    Evaluate the participant's baseline BP in terms of their assigned treatment goal (intensive goal SBP < 120 mm Hg) or (standard goal SBP < 140 mm Hg) and determine if adjustments in therapy are required based on the therapy algorithm.
20. Ask the participant to fill out (circle) the one item adherence scale on page 2 of the **Blood Pressure Medication Management Log** and the **Morisky Medication Adherence Scale** to evaluate the participant's adherence and satisfaction;
21. Initiate or convert all blood pressure-lowering medications to study drugs (if necessary). Record name of drug and dose on the **Blood Pressure Medication Management Log**. Enter BP medications that participant was taking prior to randomization as well as any new medications you will be prescribing during this visit. SPRINT investigator and or designated approved study mid-level provider should discuss continuing new or revised current medication regimen based on SPRINT assignment. The participant should be asked about their understanding of the study, questions about medication side effects, safety laboratory checks, and visit schedule and study procedures. Instruct participants on the dosing and frequency of administration for each prescribed medication;
21. Remove small part of labels from prescribed study medications at perforation and place on **Drug Dispensing Form;** then dispense study medication.
22. Instruct participants on diet and exercise and the relationship of medications with nutrition and exercise. Remind participants to take blood pressure medications the morning of next visit and to bring all their medications to each visit;
23. Schedule the follow-up visit in one month. The visit date must fall in the period from 15-45 days after the randomization visit, and should be scheduled as close to 30 days after randomization as possible. Instruct participant that the one-month visit will take ~45 minutes.
24. Thank the participant for their time and escort them to the door.

Items to be completed AFTER the participant has exited the clinic:

1. Proper processing and shipment of laboratory specimens: SPRINT Central Laboratory and if applicable, shipment of genomic specimens.
2. Transmission of ECG tracings to SPRINT ECG Laboratory.
3. Completion of independent source documentation per local IRB regulations, following Good Clinical Practice Guidelines.

4. Scan medication label bar codes into computer as soon as possible after the visit so that adequate inventory can be maintained at the clinical site;
5. Complete **Encounter and Disposition Form**;
6. Contact primary care provider as necessary;
7. Schedule MRI (If applicable);
8. Complete and/or assure (participant) completion of the following forms and enter data as required (within 7 days for all forms; within 2 days for the My Health Form):
   a) **Participant Contact Information Form**
   b) **Inclusion/Exclusion Summary Form**
   c) **Baseline Medications and Physical Exam Form**
   d) **Baseline Blood Pressure Management Form**
   e) **Blood Pressure Medication Management Log**
   f) **Morisky Medication Adherence Scale**
   g) MIND Assessments: **MoCA, Digit Symbol Coding Test and Logical Memory Test** and the Extended Battery, if applicable: the **Hopkins Verbal (HVLT), Trial Making Tests Parts A and B, Boston Naming, Modified Rey-Osterrieth Complex Figure and Category Fluency Animals, and Digit Span Forward and Backward**
   h) **My Health**
   i) Subset: **Female Health** or **Male Health**
   j) **ECG Form**
   k) **Randomization Lab Shipment Form *NOTE**: This form is NOT data entered. It is placed in the lab shipping box and sent to the Central Lab; the lab will do the data entry for the lab forms. Keep a copy for your records in the participant chart.
   l) **Drug Dispensing Form (as necessary)**
   m) **Release of Information (local not study form)**
   n) **Encounter Disposition Form**
   o) **MRI Informed Consent Form (if applicable)**

Chapter 3.c Post Randomization Visits Procedures

A.1. Overview

The SPRINT blood pressure (BP) trial component is designed to test whether a therapeutic strategy that targets a systolic blood pressure (SBP) of < 120 mm Hg reduces the rate of cardiovascular events in a middle-aged and older hypertensive population at high risk for cardiovascular events compared to a strategy that targets a SBP of < 140 mm Hg. Although there are no diastolic blood pressure (DBP) inclusion criteria, participants in both groups with DBP ≥90 mm Hg will be treated to a DBP goal of <90 mm Hg, if needed, after meeting the SBP goal, because of the many trials documenting the CVD benefits in treating to a DBP goal <90 mm Hg.

Although the treatment goals are different, the protocol visit schedule and procedures are the same for both intervention groups. Details regarding the implementation of the post randomization visits procedures are described below. Please refer to the Intervention chapter for specific information regarding medication use.

A.2. Visit Windows

Visit windows for each visit are calculated on each Participant Status Page; this is located on the right side called VISIT LIST. This has a printer version icon that sites can click and print off this visit window list for use in each participant's study chart. This Visit List contains each visit, and a Target Date and a Begin and End Window date already filled out for each visit. Ideally, visits would occur as close as possible to the TARGET Dates (which are Month 1, 2, 3, 6, 9, 12 (and quarterly thereafter) from the date of randomization). Each visit should be completed within this window. If a visit is missed (i.e., the date of the END WINDOW passes without the visit having occurred and at least some data collected), an Encounter and Disposition Form should be completed and data entered noting that the visit was missed.

A.3. Use of the Encounter and Disposition Form

The Encounter and Disposition Form is used to document every protocol-specified visit (RZ, 1M, 2M, 3M, 6M, etc.) and for PRN visits if study data was collected. Study data consists of data received from the participant. For example, if a participant comes in for medication dispensing only and no other study measures are collected and only the Drug Dispensing Form is completed, a PRN Encounter and Disposition Form should not be completed. However, if a participant comes in and has their blood pressure measured and subsequently medications are dispensed, a PRN Encounter and Disposition Form is completed.

A.4. Recommended Sequence of Post-Randomization Clinic Visit

The suggested sequencing of post-randomization clinic visit follows. Note that shaded rows indicate that the procedure or form is not completed at every visit. See Section A.4.a through A.4.i for specific directions for each visit. Some procedures/forms are collected in a subset of participants only. Additionally, if a participant is in an ancillary study, additional forms may be needed. The data entry screen for the visit for each participant will populate the expected forms. Additional forms completed can be printed and data entered via the PRN visit list. If a procedure is missed at a quarterly visit, it may be able to be collected at the next quarterly visit. See MOP Chapter 11, Procedures for BP Inactive/Lost to Follow-Up/Withdrawn Participants and Missed or Incomplete Visits, for further details.

Recommended Sequence of Post-Randomization Clinic Visits
Review Cover Page
Greeting
Review and Update Informed Consent
Blood pressure (BP Management Form)
Blood draw, urine collection
ECG (ECG Form)
Review Events (Events Ascertainment Form)<br>As needed: SAE, Day Surgery, Dialysis
Update medical release/HIPAA
Review contact information (Participant Contact Information Form)
Snack, rest, self-administered forms (My Health, Men's/Women's Health, Falls Self Efficacy)
MIND Testing (Screening and Extended, if needed)
Height, weight, physical exam and 4-meter walk (if needed) (Annual History and Physical Exam Form)
Adherence scale (Morisky Medication Adherence Scale)
Assess blood pressure therapy
Record medications (Blood Pressure Medication Management Log)
Dispense medications, as needed (Drug Dispensing Form)
Update and provide Medication Reconciliation Form to participant
Schedule next visit
Exit
Study Management Forms:<br>Encounter and Disposition Form<br>As needed: Milepost Exemption Form, Participant Status Log

A.4.a. BASELINE/RANDOMIZATION VISIT (see MOP Chapter 3b, Baseline/Randomization Visit Procedures)

A.4.b. MONTH 1 VISIT

This visit should occur within the visit window and as close to the Target Date as possible. The participants will report to the clinic and the following procedures will be conducted:

1. Prior to the participant coming in for the visit, review **Cover Page** for information specific to the participant and the visit (i.e., missing or incorrect contact information, PHQ-9 alerts, unsafe medication combinations, MIND extended battery tests needed).
2. When the participant arrives for the visit, greet him/her, and confirm that they have taken their current BP medications as prescribed. Review the participant's informed consent document and update as needed before beginning any SPRINT activities. Take the participant to an examining room.

3. Using the appropriate technique for the Omron device, obtain and evaluate both sitting and standing blood pressure and pulse values (See MOP Chapter 3, Measurements and Administering Questionnaires). Record the information on **BP Management Form.**
4. A blood sample will be obtained, processed and shipped to the SPRINT Central Lab for measurement of a chemistry profile (See Chapter 7, Central Lab). Complete the **Month 1 Lab Shipment Form**.
5. Confirm an active medical release/HIPAA is in place for the participant.
6. Assessment of BP and Adjustment of Therapy – Evaluate the participant's BP in terms of their assigned treatment goal (intensive goal SBP < 120 mm Hg) or standard goal SBP < 140 mm Hg) and determine if adjustments in therapy are required (See MOP Chapter 5, Intervention). Record current medications, dose and self-report of adherence and any adverse experiences since initiating study therapy in source documents and on the **Blood Pressure Medication Management Log.**
7. Instruct participants on administration instructions and actions to limit symptomatic orthostasis.
8. Remove small part of labels from prescribed study medications at perforation and place on **Drug Dispensing Form;** then dispense study medication. Scan label bar codes into computer as soon as possible after the visit so that adequate inventory can be maintained at the clinical site.
9. Instruct participants on diet and exercise and the relationship of medications with nutrition and exercise.
10. Schedule a clinic appointment in 1 month.
11. Remind participants to take blood pressure medications the morning of next visit and to bring all their medications to each visit. Provide updated **Medication Reconciliation Form** to participant to take home with them.
12. Contact primary care provider as necessary.
13. Complete the study management forms:
    a. Encounter and Disposition Form
    b. SAE Form (as necessary)
    c. Participant Status Log (as necessary)
14. Data enter as required:
    a. BP Management Form
    b. Blood Pressure Medication Management Log
    c. Drug Dispensing Form
    d. Encounter and Disposition Form
    e. SAE Form (as necessary – IF investigator feels it is an SAE, it must be data entered within 72 hours)
    f. Participant Status Log (as necessary)

A.4.c. MONTH 2 VISIT

This visit should occur within the visit window and as close to the Target Date as possible. The participants will report to the clinic and the following procedures will be conducted:

1. Prior to the participant coming in for the visit, review **Cover Page** for information specific to the participant and the visit (i.e., missing or incorrect contact information, PHQ-9 alerts, unsafe medication combinations, MIND extended battery tests needed).
2. When the participant arrives for the visit, greet him/her, and confirm that they have taken their current BP medications as prescribed. Review the participant's informed consent document and update as needed before beginning any SPRINT activities. Take the participant to an examining room.
3. Using the appropriate technique for the Omron device, obtain and evaluate both sitting and standing blood pressure and pulse values (See MOP Chapter 3,

Measurements and Administering Questionnaires). Record the information on **BP Management Form.**

4. Confirm an active medical release/HIPAA is in place for the participant.
5. Assessment of BP and Adjustment of Therapy – Evaluate the participant's BP in terms of their assigned treatment goal (intensive goal SBP < 120 mm Hg) or standard goal SBP < 140 mm Hg) and determine if adjustments in therapy are required (See MOP Chapter 5, Intervention). Record current medications, dose and self-report of adherence and any adverse experiences since initiating study therapy in source documents and on the **Blood Pressure Medication Management Log.**
6. Instruct participants on administration instructions and actions to limit symptomatic orthostasis.
7. Remove small part of labels from prescribed study medications at perforation and place on **Drug Dispensing Form;** then dispense study medication. Scan label bar codes into computer as soon as possible after the visit so that adequate inventory can be maintained at the clinical site.
8. Instruct participants on diet and exercise and the relationship of medications with nutrition and exercise.
9. Schedule a clinic appointment in 1 month.
10. Remind participants to take blood pressure medications the morning of next visit and to bring all their medications to each visit. Provide updated **Medication Reconciliation Form** to participant to take home with them.
11. Contact primary care provider as necessary.
12. Complete the study management forms:
    a. Encounter and Disposition Form
    b. SAE Form (as necessary)
    c. Participant Status Log (as necessary)
13. Data enter as required:
    a. BP Management Form
    b. Blood Pressure Medication Management Log
    c. Drug Dispensing Form
    d. Encounter and Disposition Form
    e. SAE Form (as necessary – IF investigator feels it is an SAE, it must be data entered within 72 hours)
    f. Participant Status Log (as necessary)

A.4.d. MONTH 3 VISIT

This visit should occur within the visit window and as close to the Target Date as possible. The participants will report to the clinic and the following procedures will be conducted:

1. Prior to the participant coming in for the visit, review **Cover Page** for information specific to the participant and the visit (i.e., missing or incorrect contact information, PHQ-9 alerts, unsafe medication combinations, MIND extended battery tests needed).
2. When the participant arrives for the visit, greet him/her, and confirm that they have taken their current BP medications as prescribed. Review the participant's informed consent document and update as needed before beginning any SPRINT activities. Take the participant to an examining room.
3. Using the appropriate technique for the Omron device, obtain and evaluate both sitting and standing blood pressure and pulse values (See MOP Chapter 3, Measurements and Administering Questionnaires). Record the information on **BP Management Form.**
4. A blood sample will be obtained, processed and shipped to the SPRINT Central Lab for measurement of a chemistry profile (See Chapter 7, Central Lab). Complete the **Month 3 Lab Shipment Form**.

5. Review any events that have occurred since the last ascertainment visit (RZ2) and complete **Events Ascertainment Form.** Complete additional study forms as directed, including: **Serious Adverse Event, Day Surgery** and/or **Dialysis**.
6. Confirm an active medical release/HIPAA is in place for the participant.
7. Provide participant pre-printed **Participant Contact Information Form** and ask them to confirm all information is up to date. Be sure to cross reference th**e Cover Page** to obtain or correct any missing or incorrect information.
8. Assessment of BP and Adjustment of Therapy – Evaluate the participant's BP in terms of their assigned treatment goal (intensive goal SBP < 120 mm Hg) or standard goal SBP < 140 mm Hg) and determine if adjustments in therapy are required (See MOP Chapter 5, Intervention). Record current medications, dose and self-report of adherence and any adverse experiences since initiating study therapy in source documents and on the **Blood Pressure Medication Management Log.**
9. Instruct participants on administration instructions and actions to limit symptomatic orthostasis.
10. Remove small part of labels from prescribed study medications at perforation and place on **Drug Dispensing Form;** then dispense study medication. Scan label bar codes into computer as soon as possible after the visit so that adequate inventory can be maintained at the clinical site.
11. Instruct participants on diet and exercise and the relationship of medications with nutrition and exercise.
12. Schedule a clinic appointment in 1 month for intensive BP (IBP) participants whose SBP is ≥ 120 mm Hg or standard BP (SBP) participants whose SBP is ≥ 160 mm Hg or there is some other clinical or safety indication requiring more frequent monitoring. All other participants should be scheduled for a clinic appointment in 3 months.
13. Remind participants to take blood pressure medications the morning of next visit and to bring all their medications to each visit. Provide updated **Medication Reconciliation Form** to participant to take home with them.
14. Contact primary care provider as necessary.
15. Complete the study management forms:
    a. Encounter and Disposition Form
    b. SAE Form (as necessary)
    c. Participant Status Log (as necessary)
16. Data enter as required:
    a. BP Management Form
    b. Event Ascertainment Form
    c. Participant Contact Information Form
    d. Blood Pressure Medication Management Log
    e. Drug Dispensing Form
    f. Encounter and Disposition Form
    g. Day Surgery Form (as necessary)
    h. Dialysis Form (as necessary)
    i. SAE Form (as necessary – IF investigator feels it is an SAE, it must be data entered within 72 hours)
    j. Participant Status Log (as necessary)

A.4.e. MILEPOST AND FOLLOW-UP VISITS (EVERY SIX MONTHS; MONTH 6, 18, 30, 42, 54, 66 VISITS)

This visit should occur within the visit window and as close to the Target Date as possible. The participants will report to the clinic and the following procedures will be conducted:

1. Prior to the participant coming in for the visit, review **Cover Page** for information specific to the participant and the visit (i.e., missing or incorrect contact

information, PHQ-9 alerts, unsafe medication combinations, MIND extended battery tests needed).

2. When the participant arrives for the visit, greet him/her, and confirm that they have taken their current BP medications as prescribed. Review the participant's informed consent document and update as needed before beginning any SPRINT activities. Take the participant to an examining room.
3. Using the appropriate technique for the Omron device, obtain and evaluate both sitting and standing blood pressure and pulse values (See MOP Chapter 3, Measurements and Administering Questionnaires). Record the information on **BP Management Form.** For intensive participants only, if directed, complete the **Milepost Exemption Form.**
4. Lab for measurement of a chemistry profile and urine albumin/creatinine will be obtained, processed and shipped to the SPRINT Central Lab (See Chapter 7, Central Lab). Complete the **Lab Shipment Form**.
   *****NOTE: Lab for measurement of chemistry profile is obtained every 6 months.***
5. Review any events that have occurred since the last ascertainment visit and complete **Events Ascertainment Form.** Complete additional study forms as directed, including: **Serious Adverse Event, Day Surgery** and/or **Dialysis**.
6. Confirm an active medical release/HIPAA is in place for the participant.
7. Provide participant pre-printed **Participant Contact Information Form** and ask them to confirm all information is up to date. Be sure to cross reference the **Cover Page** to obtain or correct any missing or incorrect information.
8. *****AT THE MONTH 6 VISIT ONLY***** Provide a snack and a place for the participant to rest and complete the self-administered forms, if necessary. A subset of participants will be selected to complete the **Falls Self Efficacy** and a **Women's Health** or **Men's Health** assessment.
9. Assessment of BP and Adjustment of Therapy – Evaluate the participant's BP in terms of their assigned treatment goal (intensive goal SBP < 120 mm Hg) or standard goal SBP < 140 mm Hg) and determine if adjustments in therapy are required (See MOP Chapter 5, Intervention). Record current medications, dose and self-report of adherence and any adverse experiences since initiating study therapy in source documents and on the **Blood Pressure Medication Management Log.**
10. Instruct participants on administration instructions and actions to limit symptomatic orthostasis.
11. Remove small part of labels from prescribed study medications at perforation and place on **Drug Dispensing Form;** then dispense study medication. Scan label bar codes into computer as soon as possible after the visit so that adequate inventory can be maintained at the clinical site.
12. Instruct participants on diet and exercise and the relationship of medications with nutrition and exercise.
13. Schedule a clinic appointment in 1 month for intensive BP (IBP) participants whose SBP is ≥ 120 mm Hg or standard BP (SBP) participants whose SBP is ≥ 160 mm Hg or there is some other clinical or safety indication requiring more frequent monitoring. All other participants should be scheduled for a clinic appointment in 3 months.
14. Remind participants to take blood pressure medications the morning of next visit and to bring all their medications to each visit. Provide updated **Medication Reconciliation Form** to participant to take home with them.
15. Contact primary care provider as necessary.
16. Complete the study management forms:
    a. Encounter and Disposition Form
    b. SAE Form (as necessary)
    c. Participant Status Log (as necessary)

17. Data enter as required:
    a. BP Management Form
    b. Events Ascertainment Form
    c. Participant Contact Information Form
    d. Blood Pressure Medication Management Log
    e. Drug Dispensing Form
    f. Encounter and Disposition Form
    g. Milepost Exemption Form (as necessary for intensive participants ONLY)
    h. Day Surgery Form (as necessary)
    i. Dialysis Form (as necessary)
    j. SAE Form (as necessary – IF investigator feels it is an SAE, it must be data entered within 72 hours)
    k. Falls Self Efficacy (if participant is in subset; month 6 visit only)
    l. Men's Health/Women's Health (if participant is in HRQL subset; month 6 visit only)
    m. Participant Status Log (as necessary)

A.4.e. MONTH 9, 15, 21, 27, 33, 39, 45, 51, 57, 63, 69 VISITS

This visit should occur within the visit window and as close to the Target Date as possible. The participants will report to the clinic and the following procedures will be conducted:

1. Prior to the participant coming in for the visit, review **Cover Page** for information specific to the participant and the visit (i.e., missing or incorrect contact information, PHQ-9 alerts, unsafe medication combinations, MIND extended battery tests needed).
2. When the participant arrives for the visit, greet him/her, and confirm that they have taken their current BP medications as prescribed. Review the participant's informed consent document and update as needed before beginning any SPRINT activities. Take the participant to an examining room.
3. Using the appropriate technique for the Omron device, obtain and evaluate both sitting and standing blood pressure and pulse values (See MOP Chapter 3, Measurements and Administering Questionnaires). Record the information on **BP Management Form.**
4. Review any events that have occurred since the last ascertainment visit and complete **Events Ascertainment Form.** Complete additional study forms as directed, including: **Serious Adverse Event, Day Surgery** and/or **Dialysis**.
5. Confirm an active medical release/HIPAA is in place for the participant.
6. Provide participant pre-printed **Participant Contact Information Form** and ask them to confirm all information is up to date. Be sure to cross reference the **Cover Page** to obtain or correct any missing or incorrect information.
7. Assessment of BP and Adjustment of Therapy – Evaluate the participant's BP in terms of their assigned treatment goal (intensive goal SBP < 120 mm Hg) or standard goal SBP < 140 mm Hg) and determine if adjustments in therapy are required (See MOP Chapter 5, Intervention). Record current medications, dose and self-report of adherence and any adverse experiences since initiating study therapy in source documents and on the **Blood Pressure Medication Management Log.**
8. Instruct participants on administration instructions and actions to limit symptomatic orthostasis.
9. Remove small part of labels from prescribed study medications at perforation and place on **Drug Dispensing Form;** then dispense study medication. Scan label bar codes into computer as soon as possible after the visit so that adequate inventory can be maintained at the clinical site.
10. Instruct participants on diet and exercise and the relationship of medications with nutrition and exercise.

11. Schedule a clinic appointment in 1 month for intensive BP (IBP) participants whose SBP is ≥ 120 mm Hg or standard BP (SBP) participants whose SBP is ≥ 160 mm Hg or there is some other clinical or safety indication requiring more frequent monitoring. All other participants should be scheduled for a clinic appointment in 3 months.
12. Remind participants to take blood pressure medications the morning of next visit and to bring all their medications to each visit. Provide updated **Medication Reconciliation Form** to participant to take home with them.
13. Contact primary care provider as necessary.
14. Complete the study management forms:
    a. Encounter and Disposition Form
    b. SAE Form (as necessary)
    c. Participant Status Log (as necessary)
15. Data enter as required:
    a. BP Management Form
    b. Events Ascertainment Form
    c. Participant Contact Information Form
    d. Blood Pressure Medication Management Log
    e. Drug Dispensing Form
    f. Encounter and Disposition Form
    g. Day Surgery Form (as necessary)
    h. Dialysis Form (as necessary)
    i. SAE Form (as necessary – IF investigator feels it is an SAE, it must be data entered within 72 hours)
    j. Participant Status Log (as necessary)

A.4.g. ANNUAL (MONTH 12, 36, AND 60) VISITS

This visit should occur within the visit window and as close to the Target Date as possible. The participants will be instructed to attend the clinic following an at least 8 hour fast. They should, however, take their blood pressure medication (with water) prior to coming to clinic. During the visit, the following procedures will be conducted:

1. Prior to the participant coming in for the visit, review **Cover Page** for information specific to the participant and the visit (i.e., missing or incorrect contact information, PHQ-9 alerts, unsafe medication combinations, MIND extended battery tests needed).
2. When the participant arrives for the visit, greet him/her, and confirm that they have taken their current BP medications as prescribed. Review the participant's informed consent document and update as needed before beginning any SPRINT activities. Take the participant to an examining room.
3. Using the appropriate technique for the Omron device, obtain and evaluate both sitting and standing blood pressure and pulse values (See MOP Chapter 3, Measurements and Administering Questionnaires). Record the information on **BP Management Form.** For intensive participants only, if directed, complete the **Milepost Exemption Form.**
4. Blood and urine samples will be obtained, processed and shipped to the SPRINT Central Lab for measurement of chemistry and lipid profiles and urine albumin/creatinine as well as serum, plasma and urine for storage (See Chapter 7, Central Lab). Complete the **Lab Shipment Form**.
5. Review any events that have occurred since the last ascertainment visit and complete **Events Ascertainment Form.** Complete additional study forms as directed, including: **Serious Adverse Event, Day Surgery** and/or **Dialysis**.
6. Confirm an active medical release/HIPAA is in place for the participant.

7. Provide participant pre-printed **Participant Contact Information Form** and ask them to confirm all information is up to date. Be sure to cross reference the **Cover Page** to obtain or correct any missing or incorrect information.
8. Provide a snack and a place for the participant to rest and complete the self-administered forms.
9. Participants will be given the **My Health Form** and instructed on how to complete it. Verify that the participant completed all items on the assessments before leaving at the end of visit. Review the answer to the PHQ-9, Question 9 specifically to determine if the response is "more than half the days" or "nearly every day." If the response is one of these options, the SPRINT PI or designated site clinician should assess the participant for risk of suicide before the participant leaves the clinic.
10. A subset of participants will be selected to complete the **Falls Self Efficacy** and a **Women's Health** or **Men's Health** assessment.
11. Obtain and perform **Annual History and Physical Exam Form**, including updates for concomitant medications, weight, smoking use, diabetes history, and marital status. A limited physical exam for safety assessment (components selected at the discretion of the investigator) will include at a minimum, weight and blood pressure measurement. For those participants age ≥75 yrs (at the baseline visit), conduct and record the time required to complete a 4 meter walk test.
12. **<u>***AT THE MONTH 12 VISIT ONLY</u>** Review and evaluate the participant's adherence and satisfaction using the **Morisky Medication Adherence Scale**
13. Assessment of BP and Adjustment of Therapy – Evaluate the participant's BP in terms of their assigned treatment goal (intensive goal SBP < 120 mm Hg) or standard goal SBP < 140 mm Hg) and determine if adjustments in therapy are required (See MOP Chapter 5, Intervention). Record current medications, dose and self-report of adherence and any adverse experiences since initiating study therapy in source documents and on the **Blood Pressure Medication Management Log.**
14. Instruct participants on administration instructions and actions to limit symptomatic orthostasis.
15. Remove small part of labels from prescribed study medications at perforation and place on **Drug Dispensing Form;** then dispense study medication. Scan label bar codes into computer as soon as possible after the visit so that adequate inventory can be maintained at the clinical site.
16. Instruct participants on diet and exercise and the relationship of medications with nutrition and exercise.
17. Schedule a clinic appointment in 1 month for intensive BP (IBP) participants whose SBP is ≥ 120 mm Hg or standard BP (SBP) participants whose SBP is ≥ 160 mm Hg or there is some other clinical or safety indication requiring more frequent monitoring. All other participants should be scheduled for a clinic appointment in 3 months.
18. Remind participants to take blood pressure medications the morning of next visit and to bring all their medications to each visit. Provide updated **Medication Reconciliation Form** to participant to take home with them.
19. Contact primary care provider as necessary.
20. Complete the study management forms:
    a. Encounter and Disposition Form
    b. SAE Form (as necessary)
    c. Participant Status Log (as necessary)
21. Data enter as required:
    a. BP Management Form
    b. Event Ascertainment Form
    c. Participant Contact Information Form

d. My Health Form
e. Annual History and Physical Exam Form
f. Morisky Medication Adherence Scale (month 12 visit only)
g. Blood Pressure Medication Management Log
h. Drug Dispensing Form
i. Encounter and Disposition Form
j. Milepost Exemption Form (as necessary for intensive participants ONLY)
k. Day Surgery Form (as necessary)
l. Dialysis Form (as necessary)
m. SAE Form (as necessary – IF investigator feels it is an SAE, it must be data entered within 72 hours)
n. Falls Self Efficacy (if participant is in subset)
o. Men's Health/Women's Health (if participant is in HRQL subset)
p. Participant Status Log (as necessary)

A.4.h. MONTH 18, 30, 42, 54, 66 VISITS

This visit should occur within the visit window and as close to the Target Date as possible. The participants will report to the clinic and the following procedures will be conducted:

1. Prior to the participant coming in for the visit, review **Cover Page** for information specific to the participant and the visit (i.e., missing or incorrect contact information, PHQ-9 alerts, unsafe medication combinations, MIND extended battery tests needed).
2. When the participant arrives for the visit, greet him/her, and confirm that they have taken their current BP medications as prescribed. Review the participant's informed consent document and update as needed before beginning any SPRINT activities. Take the participant to an examining room.
3. Using the appropriate technique for the Omron device, obtain and evaluate both sitting and standing blood pressure and pulse values (See MOP Chapter 3, Measurements and Administering Questionnaires). Record the information on **BP Management Form.** For intensive participants only, if directed, complete the **Milepost Exemption Form.**
4. Blood will be obtained, processed and shipped to the SPRINT Central Lab for measurement of a chemistry profile (See Chapter 7, Central Lab). Complete the **Lab Shipment Form**.
5. Review any events that have occurred since the last ascertainment visit and complete **Events Ascertainment Form.** Complete additional study forms as directed, including: **Serious Adverse Event, Day Surgery** and/or **Dialysis**.
6. Confirm an active medical release/HIPAA is in place for the participant.
7. Provide participant pre-printed **Participant Contact Information Form** and ask them to confirm all information is up to date. Be sure to cross reference the **Cover Page** to obtain or correct any missing or incorrect information.
8. Assessment of BP and Adjustment of Therapy – Evaluate the participant's BP in terms of their assigned treatment goal (intensive goal SBP < 120 mm Hg) or standard goal SBP < 140 mm Hg) and determine if adjustments in therapy are required (See MOP Chapter 5, Intervention). Record current medications, dose and self-report of adherence and any adverse experiences since initiating study therapy in source documents and on the **Blood Pressure Medication Management Log.**
9. Instruct participants on administration instructions and actions to limit symptomatic orthostasis.
10. Remove small part of labels from prescribed study medications at perforation and place on **Drug Dispensing Form;** then dispense study medication. Scan label bar codes into computer as soon as possible after the visit so that adequate inventory can be maintained at the clinical site.

11. Instruct participants on diet and exercise and the relationship of medications with nutrition and exercise.
12. Schedule a clinic appointment in 1 month for intensive BP (IBP) participants whose SBP is ≥ 120 mm Hg or standard BP (SBP) participants whose SBP is ≥ 160 mm Hg or there is some other clinical or safety indication requiring more frequent monitoring. All other participants should be scheduled for a clinic appointment in 3 months.
13. Remind participants to take blood pressure medications the morning of next visit and to bring all their medications to each visit. Provide updated **Medication Reconciliation Form** to participant to take home with them.
14. Contact primary care provider as necessary.
15. Complete the study management forms:
    a. Encounter and Disposition Form
    b. SAE Form (as necessary)
    c. Participant Status Log (as necessary)
16. Data enter as required:
    a. BP Management Form
    b. Event Ascertainment Form
    c. Participant Contact Information Form
    d. BP Medication Management Log
    e. Drug Dispensing Form
    f. Encounter and Disposition Form
    g. Milepost Exemption Form (as necessary for intensive participants ONLY)
    h. Day Surgery Form (as necessary)
    i. Dialysis Form (as necessary)
    j. SAE Form (as necessary – IF investigator feels it is an SAE, it must be data entered within 72 hours)
    k. Participant Status Log (as necessary)

A.4.i. ANNUAL (MONTH 24, 48 AND 72) VISITS

This visit should occur within the visit window and as close to the Target Date as possible. The participants will be instructed to attend the clinic following an at least 8 hour fast. They should, however, take their blood pressure medication (with water) prior to coming to clinic. During the visit, the following procedures will be conducted:

1. Prior to the participant coming in for the visit, review **Cover Page** for information specific to the participant and the visit (i.e., missing or incorrect contact information, PHQ-9 alerts, unsafe medication combinations, MIND extended battery tests needed).
2. When the participant arrives for the visit, greet him/her, and confirm that they have taken their current BP medications as prescribed. Review the participant's informed consent document and update as needed before beginning any SPRINT activities. Take the participant to an examining room.
3. Using the appropriate technique for the Omron device, obtain and evaluate both sitting and standing blood pressure and pulse values (See MOP Chapter 3, Measurements and Administering Questionnaires). Record the information on **BP Management Form.** For intensive participants only, if directed, complete the **Milepost Exemption Form.**
4. Blood and urine samples will be obtained, processed and shipped to the SPRINT Central Lab for measurement of fasting glucose, chemistry and lipid profiles and urine albumin/creatinine as well as serum, plasma and urine for storage (See Chapter 7, Central Lab). Complete the **Lab Shipment Form**.
5. An ECG will be obtained and transmitted to the SPRINT ECG Reading Center. Retain a copy for participant's research records. Complete the **ECG Form.**

6. Review any events that have occurred since the last ascertainment visit and complete **Events Ascertainment Form.** Complete additional study forms as directed, including: **Serious Adverse Event, Day Surgery** and/or **Dialysis**.
7. Confirm an active medical release/HIPAA is in place for the participant.
8. Provide participant pre-printed **Participant Contact Information Form** and ask them to confirm all information is up to date. Be sure to cross reference the Cover Page to obtain or correct any missing or incorrect information.
9. Provide a snack and a place for the participant to rest and complete the self-administered forms.
10. Participants will be given the **My Health Form** and instructed on how to complete it. Verify that the participant completed all items on the assessments before leaving at the end of visit. Review the answer to the PHQ-9, Question 9 specifically to determine if the response is "more than half the days" or "nearly every day." If the response is one of these options, the SPRINT PI or designated site clinician should assess the participant for risk of suicide before the participant leaves the clinic.
11. A subset of participants will be selected to complete the **Falls Self Efficacy** and a **Women's Health** or **Men's Health** assessment.
12. **MIND Study Procedures**: Complete the Screening Battery (**Logical Memory Test, MoCA, Digit Symbol Coding Test)** on all participants and the Extended Battery **(Hopkins Verbal Learning Test (HVLT), Trial Making Tests Parts A and B, Boston Naming Test, Modified Rey-Osterrieth Complex Figure, Category Fluency Animals, and Digit Span Forward and Backward)** on a subset of participants. If the Extended Battery is necessary, these forms will print automatically.
13. *****AT THE MONTH 48 VISIT ONLY***** Those participants that completed an MRI scan at Baseline will need to be scheduled for the follow-up MRI scan. Participants should be re-screened to ensure that no contraindications for an MRI are present.
14. Obtain and perform **Annual History and Physical Exam Form**, including updates for concomitant medications, weight, smoking use, diabetes history, and marital status. A limited physical exam for safety assessment (components selected at the discretion of the investigator) will include at a minimum, weight and blood pressure measurement. For those participants age ≥75 yrs (at the baseline visit), conduct and record the time required to complete a 4 meter walk test.
15. *****AT THE MONTH 48 VISIT ONLY** Review and evaluate the participant's adherence and satisfaction using the **Morisky Medication Adherence Scale**
16. Assessment of BP and Adjustment of Therapy – Evaluate the participant's BP in terms of their assigned treatment goal (intensive goal SBP < 120 mm Hg) or standard goal SBP < 140 mm Hg) and determine if adjustments in therapy are required (See MOP Chapter 05 Intervention). Record current medications, dose and self-report of adherence and any adverse experiences since initiating study therapy in source documents and on the **Blood Pressure Medication Management Log.**
17. Instruct participants on administration instructions and actions to limit symptomatic orthostasis.
18. Remove small part of labels from prescribed study medications at perforation and place on **Drug Dispensing Form;** then dispense study medication. Scan label bar codes into computer as soon as possible after the visit so that adequate inventory can be maintained at the clinical site.
19. Instruct participants on diet and exercise and the relationship of medications with nutrition and exercise.
20. Schedule a clinic appointment in 1 month for intensive BP (IBP) participants whose SBP is ≥ 120 mm Hg or standard BP (SBP) participants whose SBP is ≥

160 mm Hg or there is some other clinical or safety indication requiring more frequent monitoring. All other participants should be scheduled for a clinic appointment in 3 months.

21. Remind participants to take blood pressure medications the morning of next visit and to bring all their medications to each visit. Provide updated **Medication Reconciliation Form** to participant to take home with them.
22. Contact primary care provider as necessary.
23. Complete the study management forms:
    a. Encounter and Disposition Form
    b. SAE Form (as necessary)
    c. Participant Status Log (as necessary)
24. Data enter as required:
    a. BP Management Form
    b. ECG Form
    c. Event Ascertainment Form
    d. Participant Contact Information Form
    e. My Health Form
    f. MIND Screening Battery
    g. MIND Extended Battery (if necessary)
    h. Annual History and Physical Exam Form
    i. Morisky Medication Adherence Scale (month 48 visit only)
    j. Blood Pressure Medication Management Log
    k. Drug Dispensing Form
    l. Encounter and Disposition Form
    m. Milepost Exemption Form (as necessary for intensive participants ONLY)
    n. Day Surgery Form (as necessary)
    o. Dialysis Form (as necessary)
    p. SAE Form (as necessary – IF investigator feels it is an SAE, it must be data entered within 72 hours)
    q. Falls Self Efficacy (if participant is in subset)
    r. Men's Health/Women's Health (if participant is in HRQL subset)
    s. MRI Form (if participant is in subset)
    t. Participant Status Log (as necessary)

A.5. Supplemental (PRN) Visits

Although these visits may be required more often in the intensive blood pressure group in follow-up to milepost visits, they may apply to either group. These visits may be needed for the purposes of blood pressure checks and/or treatment/medication adjustments to comply with the protocol algorithms and/or for safety monitoring. Other reasons for a PRN visit may be possible; the reason for the visit is recorded in Question 5 on the Encounter and Disposition Form. Note that unless study data is collection, a PRN Encounter and Disposition Form is not required.

The following procedures may apply to these visits:

1. Collect all blood pressure related information.
    a. Verify prior to taking the BP measurements, that the participant has taken his/her BP medication that morning prior to coming to the visit.
    b. Record current medications, dose and self-report of adherence in source documents.
    c. Using the appropriate technique for the Omron device, obtain and evaluate both sitting and standing blood pressure and pulse values (See MOP Chapter 3, Measurements and Administering Questionnaires). Record the information on **BP Management Form.**

Intensive Blood Pressure Group

- If the SBP is at the desired goal of < 120 mm Hg, maintain current therapy.
- If the SBP ≥ 120 mm Hg, an upward dose titration or an additional drug (not already in use) should be added. Participants should be seen at monthly intervals until at goal.

Standard Blood Pressure Group

- If the SBP is ≥130 –139 mm Hg at this visit and was ≥ 135 mm Hg at previous visit, maintain current therapy.
- If the SBP < 130 mm Hg at this visit or < 135 mm Hg on 2 consecutive visits, step down therapy is indicated.
- If SBP is ≥ 160 mm Hg at this visit or ≥140 mm Hg on 2 consecutive visits, an upward dose titration or an additional drug (not already in use) must be added. It is reasonable to see the participant at monthly intervals for adjustment of therapy until the SBP is < 140 mm Hg.

2. Record current medications, dose and self-report of adherence and any adverse experiences since initiating study therapy in source documents and on the **Blood Pressure Medication Management Log.**
3. Remove small part of labels from prescribed study medications at perforation and place on **Drug Dispensing Form;** then dispense study medication. Scan label bar codes into computer as soon as possible after the visit so that adequate inventory can be maintained at the clinical site.
4. Remind participants to take blood pressure medications the morning of next visit and to bring all their medications to each visit. Provide updated **Medication Reconciliation Form** to participant to take home with them.
5. Complete the following forms and enter data as required:
   a. BP Management Form
   b. Blood Pressure Medication Management Log
   c. Drug Dispensing Form
   d. Encounter and Disposition Form

A.6. SPRINT Procedures for Non-Clinic Post-Randomization Visits

SPRINT visits should always be completed in-clinic if at all possible. If a situation arises where the participant is unwilling or unable to attend an in-clinic visit, a home visit (annual visits only) or phone visit (any visit) can be substituted for an in-clinic visit.

In special circumstances, data collection can occur by interviewing a proxy. A proxy is an individual named by the participant who is best able to provide health and contact information about the participant. Other terms that are used interchangeable with proxy are 'contact'. A Legally Authorized Representative (LAR) is a specific type of proxy.

Study visits should not be conducted solely via electronic medical record (EMR). EMR data may be useful for some retention based information (e.g., participant contact information or status). There are some situations where EMR systems can be used. For example, some clinics receive automatic notification when a research participant is hospitalized. Since hospitalizations represent a possible Serious Adverse Event attributable to the trial, they cannot be ignored, and a SAE should be completed (if applicable). However, an Event Ascertainment Form and other study information cannot be collected using EMR only.

A.6.a. GENERAL CONSIDERATIONS

The first priority for any in-clinic, home, phone or proxy visit is to complete the Event Ascertainment Form, and the SAE form, if necessary.

As budget allows, self-administered forms (My Health, Falls Self Efficacy, Men's/Women's Health, and Morisky Medication Adherence scale) can be mailed to participants with a self-addressed/postage paid envelope for return.

Sometimes home or phone visits are done because a participant is unwilling to come into the clinic. Depending on the circumstances (e.g., if the participant is a retention risk) only seek data that would not affect willingness to continue in the trial.

Appendix 3c.1 outlines which forms and procedures can be completed at home and phone visits. Please note that the appendix table includes specifics for all visits (e.g., Month 3, Month 6), although home visits should only be conducted for annual visits, unless the site's budget allows for additional visits. With the exception of Annual History and Physical Exam Form, any form that is completed in a home or phone visit should be completed in its entirety. For the Annual History and Physical Exam Form (or Annual History and Physical Exam Form with 4 Meter Walk) form, question 2 (participant weight) should not be completed at a home visit. For annual visits administered via phone contact, questions 2 and 3 of the Annual History and Physical Exam Form, and questions 2, 3, 8, 9, 10 and 11 of the Annual History and Physical Exam Form with 4 Meter Walk should be omitted.

If a re-consent is required, staff should obtain the re-consent prior to beginning the visit. For phone visits, the consent form may be mailed to the participant, along with an addressed postmarked return envelope. Sites should check with their local IRB for more information about allowed methods to collect re-consents.

Prior to conducting any home, phone, or proxy visits, each site should contact their local IRB to ensure that all local regulations are followed.

A.6.b. HOME VISITS (ANNUAL VISITS ONLY)

A.6.b.1. Preparing for the Home Visit

In scheduling the visit with the participant SPRINT staff should consider the participant's routines, meal times and rest periods. Staff should remind the participant to take his or her current blood pressure medication(s) the morning of the visit. If the visit is for a Month 12, Month 24, or Month 48 visit, staff should provide instructions about overnight fast (8 hours). When scheduling, staff should attempt to schedule the visit in the morning (for fasting visits) or after a usual rest period since fatigue can impair a participant's test performance and result in misleading data. In scheduling a home visit the participant is reminded of the approximate time needed to complete the testing, and the requirement for a quiet and private testing area. SPRINT staff should carefully explain the need to avoid distractions during the examination period. This applies to interruptions by family members, children, pets, noise from TV, radio, stereo, or phone calls. If there is a pet in the home, the participant is asked whether the pet can be kept in a separate room during the testing, with the exception of service animals used by the participant. At this time it is also determined whether the participant's proxy will be available during the home visit.

The scheduled visit, date, and time are confirmed by letter. The letter includes reference to the physical requirements for home testing. SPRINT staff telephones the participant on the day before, or the morning of the scheduled visit to confirm the appointment day and time. If needed, the examination time is adjusted. The participant is reminded of the need for a quiet space during testing.

The home examination materials are assembled on the day before the home visit; all forms are printed; biospecimen collection supplies, small container with ice (for EDTA tubes), the ECG machine, the OMRON blood pressure monitor and cuffs, 4 meter walk supplies (tape measure, masking tape, stopwatch), and MIND testing materials (for Month 24 and Month 48

visits) are gathered. Before departing for the home visit, staff verifies that equipment and testing materials are complete, that the directions to the home are clear, and that adequate travel time has been allowed.

The recommended sequence of the visit is the same as for an in-clinic visit.

A.6.b.2. Staff Safety Considerations
It is highly recommended that staff conduct home visits in 2 person teams. In the field, staff must remain aware of the surroundings and use common sense for personal safety. A written record of the participant visit and travel arrangements, as well as the examiners' cell phone numbers are left with the staff at the clinic. Purses are locked inside the trunk of the car before leaving (rather than doing this at the participant's home). Staff are encouraged to be cautious of pets, either the participant's or others, and to have car keys in hand when leaving the participant's home (do not stand by the car to search for the keys). When directions to the home are obtained, ask whether there are safety concerns or pets (the participant's or others) to be aware of.

A.6.b.3. Liability Issues
At each clinic, staff should seek counsel on the requirements of the liability insurance policy that covers this work. If paperwork is applicable for purposes of insurance, this is completed before leaving for the home visit.

A.6.b.4. Participant Safety Considerations
Alerts identified at home visits should be handled the same as if the alert was discovered during an in clinic visit. Refer to MOP Chapter 14, Safety Monitoring and Reporting, for instructions about alerts. If there is any question about symptoms the participant may be having, such as chest pain or shortness of breath, staff should contact their PI or clinician designee by phone, just as they would if the participant were in the clinic. If a situation arises where the staff believe that the participant or another member of the household is in immediate medical danger, staff should call 911.

A.6.b.5. Special considerations for Home Visits
If a Month 24 month or Month 48 visit is being conducted, a MIND certified staff member must be one member of the team.

A 4-meter walk can be done at home if a safe and acceptable course can be set up in a hallway or another area of the home. You will need a way to measure distance and a stopwatch.

For safety reasons, staff should not transport drugs to the participant's home. After assessing blood pressure at the home visit, refills and new prescriptions may be mailed to the participant. In some states, medications cannot be mailed to the participant. It is important that staff is familiar with state and local regulations. If mailing medications is not permitted, the site should use their judgment to determine the best way to get medications to the participant (i.e., take two trips to the participant's house).

A.6.b.6. Biospecimen Collection During the Home Visit
1. Biospecimens can be collected at the home. If fasting is required, but the participant is not fasting, collect according the blood as specified in the protocol and indicate on the form that the participant is not fasting. The blood collection tubes need to be centrifuged within 1 hour of the draw. Longer delays in centrifugation will result in falsely elevated or decreased lab values (example: falsely elevated potassium values and falsely decreased glucose values).
2. Bring the lab collection form, visit specific lab processing charts (found in the MOP Chapter 7, Biospecimen Collection and Processing Manual), visit specific kit,

phlebotomy supplies, specimen rack, a shipping container with a frozen gel pack, and an extra frozen gel pack inside a zip lock plastic bag for urine samples. Place all of these materials inside of a small hand held cooler.

3. Collect the blood tubes at the end of the visit to ensure that the samples can be centrifuged within 1 hour of collection.
4. Follow the visit specific instructions in the MOP Chapter 7, Biospecimen Collection and Processing Manual.
5. After the blood collection tubes have been collected, place any 7.5 mL red-top w/gel separator (SST) tubes in the specimen rack and place inside the hand held cooler. These tubes will clot while being transported back to the clinic. These tubes are kept at room temperature while being transported back to the clinic. Any centrifugation, processing, and shipping procedures will be performed back at the clinic.
6. After blood collection tubes have been collected, wrap any 8.0 mL purple-top (EDTA) w/gel separator tubes and any 10.0 mL purple top (EDTA) tubes in paper towels, place in plastic biohazard bag, and place on frozen gel pack inside the Styrofoam shipping container. These tubes need to be kept at refrigerated temperature while being transported back to the clinic. The shipping container with samples can be placed inside the hand held cooler. Any centrifugation, processing, and shipping procedures will be performed back at the clinic.
7. If a urine sample is needed for the visit, place a frozen gel back at the bottom of a plastic zip lock bag and then place the urine cup on top of the frozen gel pack inside the bag. NOTE: Make sure the cover on the urine container is tightly sealed so the urine does not leak during transport. The urine sample can be placed into the hand held cooler for transport back to the clinic. The urine sample can be aliquoted into the transport tube and prepared for shipment back at the clinic.
8. Upon return to the clinic, follow the visit specific instructions in MOP Chapter 7, Biospecimen Collection and Processing Manual, for centrifuging, processing, and shipping the samples.
9. Small portable centrifuges are an option for sites to purchase if centrifuging the blood samples within1 hour of collection is not possible. The portable centrifuge can be used with a portable power system or can also be plugged directly into a conventional grounded outlet. (See Appendix 3c.2, Centrifugation of SPRINT Blood Collection Tubes using a Portable Centrifuge for details).

A.6.b.7. ECG Recording During the Home Visit
The procedures for recording SPRINT ECGs during the home visits are the same as those during the clinic visits.

1. Electrocardiograph (ECG Machine):
   - Similar to the SPRINT clinic visits, the ECG machine to be used during the home visits is the GE MAC 1200 electrocardiograph.
   - The MAC1200 is a portable device and can easily be moved from the clinic to the participants' homes.
   - Make sure that the ECG machine battery is fully charged. You may keep it charging the night before the home visit.
   - Although in most cases the ECG recording will be conducted using the machine battery, make sure to take the power cable in case of troubles with the battery.

2. Supplies
   - Make sure to take enough supplies, especially electrodes.
   - Always take extra electrodes during the home visits
   - Table 1 in MOP Chapter 8, ECG, summarizes the supplies needed for recording ECGs.
   -

3. Preparation for ECG recording and location of ECG electrodes:
   - Follow the same procedures of preparation for ECG recording and location of ECG electrodes you usually do during the SPRINT clinic visits as detailed in MOP Chapter 8, ECG.

4. Local ECG reading (Alert ECGs)
   - If you find any ECG abnormality that fall under the "Alert ECGs" listed in the MOP Chapter 8, ECG, do not alarm the participant by telling him/her about it. Instead, contact the Principal Investigator or their designated investigator for ECG alerts to discuss the concern and identify and take the appropriate steps (i.e., contact 911).

5. Data management procedures
   - Since the same ECG machine in the SPRINT clinic visits will be used during the home visits, there is no need to change the machine set-up.
   - Managing the ECG machine directory that includes the list of the recorded ECGs should be done at the clinic after returning from the home visit.
   - More information on the data management procedures and machine set-up is located in MOP Chapter 8, ECG.

6. ECG Transmission
   - The ECG machine can safely store up to 50 ECGs. Therefore, transmitting the ECGs to the ECG Reading should be done after returning to the clinic not from the participants' homes
   - For details on the ECG transmission, see page 8-13 of MOP Chapter 8, ECG.

7. Quality Control
   - ECGs conducted during home visits will be handled the same way as the SPRINT clinic visits ECGs in terms of quality control. Therefore, the technicians should try as much as possible to follow MOP Chapter 8, ECG, in recording these ECGs
   - Always keep the contact information of the ECG Reading Center staff during the home visits. In case of urgent questions, the ECG Reading staff may be able to troubleshoot any recording errors you may face during the home visits. Appendix A in MOP Chapter 8, ECG, lists all the ECG Reading Center staff you can contact.
   - ECG-related forms could be filled after returning from the home visit.
   - Make sure to return to the clinic with the ECG printouts and do not leave them with the participant. These printouts could be needed in case of unsuccessful transmission. If the participant requests a copy of his or her ECG, inform the participant that you will make a copy and mail it to them after you return from the home visit.

A.6.b.8. MIND Testing During the Home Visit
If conducting a Month 24 or Month 48 visit, staff should administer the complete MIND Screening Battery during home visits, and if the participant is in the Extended MIND substudy (the 2800), the complete Extended Battery should be administered as well. If the participant triggers the Screening Battering, the Extended MIND battery should be administered while at the home. If the home visit team is made up of more than one individual, one technician can score the screening battery to determine whether the participant has triggered further testing, while the other technician continues with the visit. Please refer to MOP Chapter 4, Cognition Assessment Procedures-SPRINT MIND, for additional information.

A.6.c. PHONE VISITS (ANY VISIT)
In scheduling the phone visit with the participant, SPRINT staff should consider the participant's routines, meal times and rest periods. Staff should attempt to schedule the visit after a usual rest period since fatigue can impair a participant's test performance and result

in misleading data. In scheduling a phone visit the participant is reminded of the approximate time needed to complete the testing, and the need for a quiet area for them to take the call free of interruptions.

A.6.c.1. Recommended Sequence of Post-Randomization Phone Visit
The suggested sequencing of post-randomization phone visit follows. Note that shaded rows indicate that the procedure or form is not completed at every visit. Some protocol-specified procedures/forms are unable to be collected via phone. Since blood pressure is unable to be measured if a visit is completely solely by phone, a protocol-specified visit can never be completed as specified in the protocol (i.e., Question 7 on the Encounter and Disposition Form should always be answered as “No”).

Some procedures/forms are collected in a subset of participants only. Additionally, if a participant is in an ancillary study, additional forms may be needed. The data entry screen for the visit for each participant will populate the expected forms. Additional forms completed can be printed and data entered via the PRN visit list. If a procedure is missed at a quarterly visit, it may be able to be collected at the next quarterly visit. See MOP Chapter 11, Procedures for BP Inactive/Lost to Follow-Up/Withdrawn Participants and Missed or Incomplete Visits, for further details.

Recommended Sequence of Post-Randomization Phone Visits
Review Cover Page
Forms to be mailed to participant:<br>Informed Consent, My Health,<br>Men’s/Women’s Health, Falls Self Efficacy,<br>Morisky Medication Adherence Scale
Greeting
Review Events (Events Ascertainment Form)<br>As needed: SAE, Day Surgery, Dialysis
Assess blood pressure therapy
Record medications (Blood Pressure Medication Management Log)
Review contact information (Participant Contact Information Form)
Partial Annual History and Physical Exam Form
Schedule next visit
Exit
Mail medications, as needed and allowed by local regulations (Drug Dispensing Form)
Update and mail Medication Reconciliation Form to participant, if needed
Study Management Forms:<br>Encounter and Disposition Form<br>As needed: Milepost Exemption Form,<br>Participant Status Log

A.6.c.2. Special Considerations for Phone Visits
For phone visits, drug dispensing can be done for refills only. Medication and/or dose changes generally should not be made over phone. If a participant is continuing on their current medication, but is scheduled to run out before their next in-clinic visit, a new bottle may be mailed to a participant’s home. In some states, medications cannot be mailed to the participant. It is important that staff is familiar with state and local regulations. If mailing

medications is not permitted, the site should use their judgment to determine the best way to get medications to the participant. Generally, if a participant has not had an in-clinic or home visit in over a 9 month period (i.e., participant has been solely followed by the phone for 9 months), the clinic will need to assess the implications for continuing to supply refills of medications without actively managing the participant's blood pressure.

A.6.c.3. Participant Safety Considerations
Alerts identified at phone visits should be handled the same as if the alert was discovered during an in clinic visit. Refer to MOP Chapter 14, Safety Monitoring and Reporting, for instructions about alerts. In the rare case that a staff member encounters a person who is under significant emotional distress or has suicidal ideation, the staff may need to take additional steps. Typically, staff should contact the site PI or clinical supervisor to discuss the situation and make a plan for safety and referral to a mental health professional (examples are in the MOP Chapter 14). If serious suicidal ideation is detected (i.e., in the last month, the participant has had a suicide plan or attempted suicide), immediate action by the interviewer is required. The interviewer should enlist the help of 911 and the study clinical supervisor. On a different phone, a staff member should contact 911 and apprise them of the situation. Someone should keep the participant on the phone line until the emergency responders arrive at the participant's location.

A.6.c.4. MIND Testing During the Phone Visit
For telephone visits, the MIND Screening Battery will be administered centrally by the Coordinating Center. The MIND Screening Battery will be completed using the Modified Telephone Interview for Cognitive Status (TICS-M), the Category Fluency Animals Test, the Oral Trail Making Tests A & B and the Functional Assessment Questionnaire (FAQ), administered to a contact. These tests will be administered at month 24 and month 48 visits. The MIND Extended Battery cannot be completed over the phone.

<u>A.6.d. DATA COLLECTION VIA PROXY</u>
As stated in MOP Chapter 13, Ascertainment and Documentation of Study Outcomes, "If it is not possible to complete the Event Ascertainment Form or the SAE form with the participant (e.g. in a participant with severe speech problems due to a stroke or cognitive impairment), these forms can be completed by interviewing a proxy informant designated by the participant. When a participant is incapacitated and unable to furnish this information, interview a family member or friend instead, if possible, and indicate this on the Event Ascertainment form and/or Serious Adverse Event form in the space provided."

Each site must follow their state and local regulations as to order of distinction about who to contact for any release of medical information. At every visit, clinic staff should review with the participant who the appropriate contact would be and confirm that the contact information for that person is correct. A note can be placed in the Participant Notes section on the website to indicate who the participant has selected on the source document copy of the Participant Contact Information Form.

A letter for participants to give to the proxy informants (or anyone listed on the Participant Contact Information Form) is located on the SPRINT website and in the SPRINT Survival Kit. This letter provides the proxy informants information about the SPRINT study. The letter has been approved by the Wake Forest IRB and can be used as is or as a template for sites. Sites are welcome to create their own documents to introduce the SPRINT study to contacts/proxies/LARs/etc. and/or modify the template according to their local requirements. Each CCN should work closely with their sites to assess their local issues and develop appropriate procedures.

In some circumstances (e.g., a participant with cognitive impairment), the study staff may be able to obtain blood pressure, blood collection, and other study data from the participant; however the Event Ascertainment Form is administered to the proxy informant.

The only study forms that can be collected via proxy informants are the Event Ascertainment Form, the Serious Adverse Event Form, the Day Surgery Form and the Dialysis Form. The type of contact made with the proxy should be indicated on the Encounter and Disposition Form in Question 2 (e.g., if proxy contact is made by phone, "phone" should be selected). The individual from whom information was collected should be recorded on the Event Ascertainment Form and the SAE Form.

A.7. Central Phone Assessment for Study Outcomes and MIND Administration

The SPRINT phone assessment for study outcomes and MIND administration will be done centrally by the SPRINT Coordinating Center (CC). If a participant misses two consecutive visits OR if they do not complete the MIND battery or the Events Ascertainment Form at their visits, these questionnaires will be administered centrally. A participant can be called to obtain outcomes data only, MIND data only, or both outcomes and MIND data. Appendix 3C.3 provides the Central Phone Assessment Flow Chart.

Beginning at the 30M visit, participants will appear on the Central Phone Assessment (CPA) list if the Event Ascertainment Form has not been completed for the participant's two most recent visits. For example, if a participant does not have the Event Ascertainment Form completed at their 24M and their 27M visit, the participant will be on the list for a central phone assessment. The participant will stay on the CPA list until an Event Ascertainment Form is completed or the maximum number of calls have been made. Participants, who were removed from the CPA list because the Event Ascertainment Form was completed OR because the maximum number of calls was made, will be placed back on the CPA list if they again miss two consecutive visits where the Event Ascertainment Form is not collected.

If a participant is in their 30M window and the Year 2 MIND tests have not been completed in the clinic (or 54M window following the Year 4 MIND tests), the participant will appear on the list for CPA. The participant will remain on the list until the MIND tests are completed, the maximum number of calls has been made, OR the 39M visit window opens (63M window for Year 4 MIND test).

SPRINT Coordinating Center staff administering the SPRINT study questionnaires will review the SPRINT website to determine which participants to call and which form(s) to administer – Event Ascertainment, MIND, or both. To see which participants will be called, CCN Coordinators and Clinic Staff should review the report found at: Reports > Clinical Operations > Telephone Battery Participants – Site Level **OR** Reports > CCN Coordinators > Telephone Battery Participants – CCN Level. (See Figure below.) Clinic Staff should confirm that the Participant Contact Information Form is updated and that all relevant information is placed in the Participant Notes section (on the Participant Status Page under Data Entry/Management).

SPRINT Telephone Batteries
See Participant Notes For Additional Information

Clinic	PID	Instance on List	Phone Battery Call Attempt	Phone Battery Call Outcome	Date of Phone Battery Call Attempt	Participant Notes?	Date Participant Notes Last Updated	Most Recent Note Written by CoC?
[redacted]	[redacted]	2	.	.	[redacted]	Yes	[redacted]	Yes
[redacted]	[redacted]	1	.	.	[redacted]	Yes	[redacted]	
[redacted]	[redacted]	2	3	Left Message	[redacted]	Yes	[redacted]	
[redacted]	[redacted]	1	5	Max Calls Made	[redacted]	Yes	[redacted]	Yes
[redacted]	[redacted]	1	7	Max Calls Made	[redacted]	.	[redacted]	
[redacted]	[redacted]	1	6	Completed	[redacted]	Yes	[redacted]	Yes
[redacted]	[redacted]	1	8	Contact Incorrect	[redacted]	No	[redacted]	Yes
[redacted]	[redacted]	1	3	Contact Refused	[redacted]	Yes	[redacted]	Yes
[redacted]	[redacted]	1	3	Max Calls Made	[redacted]	.	[redacted]	
[redacted]	[redacted]	1	5	Left Message	[redacted]	Yes	[redacted]	
[redacted]	[redacted]	1	3	Max Calls Made	[redacted]	Yes	[redacted]	Yes
[redacted]	[redacted]	1	4	Not Completed	[redacted]	.	[redacted]	
[redacted]	[redacted]	1	2	Scheduled	[redacted]	.	[redacted]	
[redacted]	[redacted]	1	3	Left Message	[redacted]	.	[redacted]	
[redacted]	[redacted]	1	4	Scheduled	[redacted]	.	[redacted]	
[redacted]	[redacted]	1	2	Left Message	[redacted]	.	[redacted]	
[redacted]	[redacted]	1	1	Contact Refused	[redacted]	Yes	[redacted]	Yes
[redacted]	[redacted]	1	2	Left Message	[redacted]	.	[redacted]	
[redacted]	[redacted]	1	2	Left Message	[redacted]	.	[redacted]	
[redacted]	[redacted]	1	.	.	[redacted]	.	[redacted]	
[redacted]	[redacted]	1	.	.	[redacted]	.	[redacted]	
[redacted]	[redacted]	1	.	.	[redacted]	.	[redacted]	

Figure. Example Telephone Battery Participants Report – Site Level. (Note that information in the clinic, PID and all dates have been removed; but this information is displayed on the report.)

CC phone assessors will make at least four attempts to reach the participant. Calls will be placed at varying times of day with at least one attempt in the evening. The outcome of the call (completed, left message, scheduled, refused, max calls made, contact information incorrect, contact information missing) will be logged in the Participant Notes section and listed on the report. **It is the site's responsibility to monitor this report and update contact information as needed and/or print off source documents for completed phone calls.**

The CC phone assessors will complete and data enter the following questionnaires:

Event Ascertainment Form
MIND Cover Page
Telephone Interview for Cognition (TICS)
Oral Trails

Category Fluency

The CC phone assessors will data enter all forms completed, scan all completed forms, and upload them to the SPRINT website. The scanned forms will serve as the source documents and will be available on the SPRINT website (Data Management >> Download Central PDFs). The completed scanned forms should be printed and placed in the participant's source documents.

Should the clinic staff need to review the data or make edits to any of the forms completed centrally, these forms will be displayed under PRN.

If an outcome or event is reported on the Events Ascertainment Form, the CC phone assessor will notify Debbie Felton, Loretta Cloud, and Marjorie Howard at the CC for follow-up with the local clinic. Clinic Staff will need to complete any additional forms, obtain any needed medical records, and will be responsible for any additional requests made through the Outcomes Tracking System. The site will be responsible for making any needed edits to the Events Ascertainment Form.

If the person who answers the phone indicates that the participant is unavailable via phone because he/she is hospitalized, in a nursing home/skilled nursing facility, or has passed away, CC phone assessors will notify Debbie Felton, Loretta Cloud, and Marjorie Howard at the CC immediately via email and no forms will be completed. The CC will notify by email the clinic staff responsible for completion of the Serious Adverse Event (SAE) Form, if needed. The phone assessors will update the Participant Notes section with the information they received during the phone call.

Completion of the SAE Form by Clinic Staff:

- The SAE form should be completed with as much information as possible (the Participant Notes section of the Participant Status page will be updated by the CC so the site has sufficient information to begin completing the SAE Form) and entered on the SPRINT website within three days of receiving the email from the CC. For additional information, sites can also view the centrally administered Events Ascertainment Form, if it was completed, on the website under PRN forms.

- When completing the SAE form, Q2 "Type of Report" should be answered as "other" and specified as "centrally ascertained SAE". For Q3, select 'other' and also specify as "centrally ascertained SAE" and any others that apply.

- Clinic staff is responsible for any additional requests regarding the centrally ascertained SAE(s) through the SAE Tracking system including completion of missing items or edits on the SAE form and obtaining medical records as requested.

Any questions regarding the central data collection process should be addressed to Nancy Woolard, Debbie Felton, Loretta Cloud, or Marjorie Howard.

Appendix 3C.1.
SPRINT Post Randomization Study Forms/Procedures and Visit Types.

An 'X' indicates that the form or procedure can be collected based upon the visit type

Visit	Form or Procedure	Home Visit	Phone Visit
1M	Blood Pressure Medication Management Log	X	X
	Drug Dispensing Form	X[a]	refills only[a]
	Encounter and Disposition Form	X	X
	Intensive BP Management Form 1M Visit	X[b]	
	Lab Form Month 1M, 3M, 18M, 30M, 42M, 54M and PRN Visits	see special considerations	
	Standard BP Management Form 1M, 6M, 12M, 24M, ... and 72M Visits	X[b]	
2M	Blood Pressure Medication Management Log	X	X
	Drug Dispensing Form	X[a]	refills only[a]
	Encounter and Disposition Form	X	X
	Intensive BP Management Form 2M, 3M, 9M, ... PRN Visits	X[b]	
	Standard BP Management Form 2M, 3M, 9M, PRN and non-annual Visits	X[b]	
3M	Blood Pressure Medication Management Log	X	X
	Drug Dispensing Form	X[a]	refills only[a]
	Encounter and Disposition Form	X	X
	Event Ascertainment Form	X[c]	X[c]
	Intensive BP Management Form 2M, 3M, 9M, ... PRN Visits	X[b]	
	Lab Form Month 1M, 3M, 18M, 30M, 42M, 54M and PRN Visits	see special considerations	
	Participant Contact Information Form	X	X
	Standard BP Management Form 2M, 3M, 9M, PRN and non-annual Visits	X[b]	
6M	Blood Pressure Medication Management Log	X	X
	Drug Dispensing Form	X[a]	refills only[a]
	Encounter and Disposition Form	X	X
	Event Ascertainment Form	X[c]	X[c]
	Falls Self Efficacy	mailed	mailed
	Intensive BP Management Form 6m, ... Milepost Visits	X[b]	
	Lab Form Month 6, 36, 60	see special considerations	
	Men's Health	mailed	mailed
	Participant Contact Information Form	X	X
	Standard BP Management Form 1M, 6M, 12M, 24M, ... and 72M Visits	X[b]	
	Women's Health	mailed	mailed

Visit	Form or Procedure	Home Visit	Phone Visit
9M	Blood Pressure Medication Management Log	X	X
	Drug Dispensing Form	X[a]	refills only[a]
	Encounter and Disposition Form	X	X
	Event Ascertainment Form	X[c]	X[c]
	Intensive BP Management Form 2M, 3M, 9M, ... PRN Visits	X[b]	
	Participant Contact Information Form	X	X
	Standard BP Management Form 2M, 3M, 9M, PRN and non-annual Visits	X[b]	
12M	Annual History and Physical Exam Form	partial[d]	partial[e]
	Annual History and Physical Exam Form with 4 Meter Walk	partial[d]	partial[e]
	Blood Pressure Medication Management Log	X	X
	Drug Dispensing Form	X[a]	refills only[a]
	Encounter and Disposition Form	X	X
	Event Ascertainment Form	X[c]	X[c]
	Falls Self Efficacy	mailed	mailed
	Intensive BP Management Form 6m, ... Milepost Visits	X[b]	
	Lab Form Month 12, 24, 48	see special considerations	
	Men's Health	mailed	mailed
	My Health Form	mailed	mailed
	Participant Contact Information Form	X	X
	Standard BP Management Form 1M, 6M, 12M, 24M, ... and 72M Visits	X[b]	
	The Morisky Medication Adherence Scale	mailed	Mailed
	Women's Health	mailed	mailed
15M	Blood Pressure Medication Management Log	X	X
	Drug Dispensing Form	X[a]	refills only[a]
	Encounter and Disposition Form	X	X
	Event Ascertainment Form	X[c]	X[c]
	Intensive BP Management Form 2M, 3M, 9M, ... PRN Visits	X[b]	
	Participant Contact Information Form	X	X
	Standard BP Management Form 2M, 3M, 9M, PRN and non-annual Visits	X[b]	

Visit	Form or Procedure	Home Visit	Phone Visit
18M	Blood Pressure Medication Management Log	X	X
	Drug Dispensing Form	X[a]	refills only[a]
	Encounter and Disposition Form	X	X
	Event Ascertainment Form	X[c]	X[c]
	Intensive BP Management Form 18M, ... Milepost Visits	X[b]	
	Lab Form Month 1M, 3M, 18M, 30M, 42M, 54M and PRN Visits	see special considerations	
	Participant Contact Information Form	X	X
	Standard BP Management Form 2M, 3M, 9M, PRN and non-annual Visits	X[b]	
21M	Blood Pressure Medication Management Log	X	X
	Drug Dispensing Form	X[a]	refills only[a]
	Encounter and Disposition Form	X	X
	Event Ascertainment Form	X[c]	X[c]
	Intensive BP Management Form 2M, 3M, 9M, ... PRN Visits	X[b]	
	Participant Contact Information Form	X	X
	Standard BP Management Form 2M, 3M, 9M, PRN and non-annual Visits	X[b]	
24M	Annual History and Physical Exam Form	partial[d]	partial[e]
	Annual History and Physical Exam Form with 4 Meter Walk	partial[d]	partial[e]
	Blood Pressure Medication Management Log	X	X
	Drug Dispensing Form	X[a]	refills only[a]
	ECG Form	X[b]	
	Encounter and Disposition Form	X	X
	Event Ascertainment Form	X[c]	X[c]
	Falls Self Efficacy	mailed	mailed
	Intensive BP Management Form 6m, ... Milepost Visits	X[b]	
	Lab Form Month 12, 24, 48	see special considerations	
	Men's Health	mailed	mailed
	MIND Screening Battery	X	completed centrally
	MIND Extended Battery (if participant is part of the 2800 or if Screening Battery is triggered)	X	
	My Health Form	mailed	mailed
	Participant Contact Information Form	X	X
	Standard BP Management Form 1M, 6M, 12M, 24M, ... and 72M Visits	X[b]	
	Women's Health	mailed	mailed

Visit	Form or Procedure	Home Visit	Phone Visit
27M	Blood Pressure Medication Management Log	X	X
	Drug Dispensing Form	X[a]	refills only[a]
	Encounter and Disposition Form	X	X
	Event Ascertainment Form	X[c]	X[c]
	Intensive BP Management Form 2M, 3M, 9M, ... PRN Visits	X[b]	
	Participant Contact Information Form	X	X
	Standard BP Management Form 2M, 3M, 9M, PRN and non-annual Visits	X[b]	
30M	Blood Pressure Medication Management Log	X	X
	Drug Dispensing Form	X[a]	refills only[a]
	Encounter and Disposition Form	X	X
	Event Ascertainment Form	X[c]	X[c]
	Intensive BP Management Form 18M, ... Milepost Visits	X[b]	
	Lab Form Month 1M, 3M, 18M, 30M, 42M, 54M and PRN Visits	see special considerations	
	Participant Contact Information Form	X	X
	Standard BP Management Form 2M, 3M, 9M, PRN and non-annual Visits	X[b]	
33M	Blood Pressure Medication Management Log	X	X
	Drug Dispensing Form	X[a]	refills only[a]
	Encounter and Disposition Form	X	X
	Event Ascertainment Form	X[c]	X[c]
	Intensive BP Management Form 2M, 3M, 9M, ... PRN Visits	X[b]	
	Participant Contact Information Form	X	X
	Standard BP Management Form 2M, 3M, 9M, PRN and non-annual Visits	X[b]	

Visit	Form or Procedure	Home Visit	Phone Visit
36M	Annual History and Physical Exam Form	partial[d]	partial[e]
	Annual History and Physical Exam Form with 4 Meter Walk	partial[d]	partial[e]
	Blood Pressure Medication Management Log	X	X
	Drug Dispensing Form	X[a]	refills only[a]
	Encounter and Disposition Form	X	X
	Event Ascertainment Form	X[c]	X[c]
	Falls Self Efficacy	mailed	mailed
	Intensive BP Management Form 6m, ... Milepost Visits	X[b]	
	Lab Form Month 6, 36, 60	see special considerations	
	Men's Health	mailed	mailed
	My Health Form	mailed	mailed
	Participant Contact Information Form	X	X
	Standard BP Management Form 1M, 6M, 12M, 24M, ... and 72M Visits	X[b]	
	Women's Health	mailed	mailed
39M	Blood Pressure Medication Management Log	X	X
	Drug Dispensing Form	X[a]	refills only[a]
	Encounter and Disposition Form	X	X
	Event Ascertainment Form	X[c]	X[c]
	Intensive BP Management Form 2M, 3M, 9M, ... PRN Visits	X[b]	
	Participant Contact Information Form	X	X
	Standard BP Management Form 2M, 3M, 9M, PRN and non-annual Visits	X[b]	
42M	Blood Pressure Medication Management Log	X	X
	Drug Dispensing Form	X[a]	refills only[a]
	Encounter and Disposition Form	X	X
	Event Ascertainment Form	X[c]	X[c]
	Intensive BP Management Form 18M, ... Milepost Visits	X[b]	
	Lab Form Month 1M, 3M, 18M, 30M, 42M, 54M and PRN Visits	see special considerations	
	Participant Contact Information Form	X	X
	Standard BP Management Form 2M, 3M, 9M, PRN and non-annual Visits	X[b]	

Visit	Form or Procedure	Home Visit	Phone Visit
45M	Blood Pressure Medication Management Log	X	X
	Drug Dispensing Form	X[a]	refills only[a]
	Encounter and Disposition Form	X	X
	Event Ascertainment Form	X[c]	X[c]
	Intensive BP Management Form 2M, 3M, 9M, ... PRN Visits	X[b]	
	Participant Contact Information Form	X	X
	Standard BP Management Form 2M, 3M, 9M, PRN and non-annual Visits	X[b]	
48M	Annual History and Physical Exam Form	partial[d]	partial[e]
	Annual History and Physical Exam Form with 4 Meter Walk	partial[d]	partial[e]
	Blood Pressure Medication Management Log	X	X
	Drug Dispensing Form	X[a]	refills only[a]
	ECG Form	X[b]	
	Encounter and Disposition Form	X	X
	Event Ascertainment Form	X[c]	X[c]
	Falls Self Efficacy	mailed	mailed
	Intensive BP Management Form 6m, ... Milepost Visits	X[b]	
	Lab Form Month 12, 24, 48	see special considerations	
	Men's Health	mailed	mailed
	MIND Screening Battery	X	completed centrally
	MIND Extended Battery (if participant is part of the 2800 or if Screening Battery is triggered)	X	
	MRI Initial Contact Screening Form	X	
	MRI Appointment Confirmation Form	X	
	My Health Form	mailed	mailed
	Participant Contact Information Form	X	X
	Standard BP Management Form 1M, 6M, 12M, 24M, ... and 72M Visits	X[b]	
	The Morisky Medication Adherence Scale	mailed	mailed
	Women's Health	mailed	mailed
51M	Blood Pressure Medication Management Log	X	X
	Drug Dispensing Form	X[a]	refills only[a]
	Encounter and Disposition Form	X	X
	Event Ascertainment Form	X[c]	X[c]
	Intensive BP Management Form 2M, 3M, 9M, ... PRN Visits	X[b]	
	Participant Contact Information Form	X	X
	Standard BP Management Form 2M, 3M, 9M, PRN and non-annual Visits	X[b]	

Visit	Form or Procedure	Home Visit	Phone Visit
54M	Blood Pressure Medication Management Log	X	X
	Drug Dispensing Form	X[a]	refills only[a]
	Encounter and Disposition Form	X	X
	Event Ascertainment Form	X[c]	X[c]
	Intensive BP Management Form 18M, ... Milepost Visits	X[b]	
	Lab Form Month 1M, 3M, 18M, 30M, 42M, 54M and PRN Visits	see special considerations	
	Participant Contact Information Form	X	X
	Standard BP Management Form 2M, 3M, 9M, PRN and non-annual Visits	X[b]	
57M	Blood Pressure Medication Management Log	X	X
	Drug Dispensing Form	X[a]	refills only[a]
	Encounter and Disposition Form	X	X
	Event Ascertainment Form	X[c]	X[c]
	Intensive BP Management Form 2M, 3M, 9M, ... PRN Visits	X[b]	
	Participant Contact Information Form	X	X
	Standard BP Management Form 2M, 3M, 9M, PRN and non-annual Visits	X[b]	
60M	Annual History and Physical Exam Form	partial[d]	partial[e]
	Annual History and Physical Exam Form with 4 Meter Walk	partial[d]	partial[e]
	Blood Pressure Medication Management Log	X	X
	Drug Dispensing Form	X[a]	refills only[a]
	Encounter and Disposition Form	X	X
	Event Ascertainment Form	X[c]	X[c]
	Falls Self Efficacy	mailed	mailed
	Intensive BP Management Form 6m, ... Milepost Visits	X[b]	
	Lab Form Month 6, 36, 60	see special considerations	
	Men's Health	mailed	mailed
	My Health Form	mailed	mailed
	Participant Contact Information Form	X	X
	Standard BP Management Form 1M, 6M, 12M, 24M, ... and 72M Visits	X[b]	
	Women's Health	mailed	mailed

Visit	Form or Procedure	Home Visit	Phone Visit
63M	Blood Pressure Medication Management Log	X	X
	Drug Dispensing Form	X[a]	refills only[a]
	Encounter and Disposition Form	X	X
	Event Ascertainment Form	X[c]	X[c]
	Intensive BP Management Form 2M, 3M, 9M, ... PRN Visits	X[b]	
	Participant Contact Information Form	X	X
	Standard BP Management Form 2M, 3M, 9M, PRN and non-annual Visits	X[b]	
66M	Blood Pressure Medication Management Log	X	X
	Drug Dispensing Form	X[a]	refills only[a]
	Encounter and Disposition Form	X	X
	Event Ascertainment Form	X[c]	X[c]
	Intensive BP Management Form 18M, ... Milepost Visits	X[b]	
	Lab Form Month 1M, 3M, 18M, 30M, 42M, 54M and PRN Visits	see special considerations	
	Participant Contact Information Form	X	X
	Standard BP Management Form 2M, 3M, 9M, PRN and non-annual Visits	X[b]	
69M	Blood Pressure Medication Management Log	X	X
	Drug Dispensing Form	X[a]	refills only[a]
	Encounter and Disposition Form	X	X
	Event Ascertainment Form	X[c]	X[c]
	Intensive BP Management Form 2M, 3M, 9M, ... PRN Visits	X[b]	
	Participant Contact Information Form	X	X
	Standard BP Management Form 2M, 3M, 9M, PRN and non-annual Visits	X[b]	

Visit	Form or Procedure	Home Visit	Phone Visit
72M	Annual History and Physical Exam Form	partial[d]	partial[e]
	Annual History and Physical Exam Form with 4 Meter Walk	partial[d]	partial[e]
	Blood Pressure Medication Management Log	X	X
	Drug Dispensing Form	X[a]	refills only[a]
	Encounter and Disposition Form	X	X
	Event Ascertainment Form	X[c]	X[c]
	Falls Self Efficacy	mailed	mailed
	Intensive BP Management Form 6m, ... Milepost Visits	X[b]	
	Lab Form Month 6, 36, 60	see special considerations	
	Men's Health	mailed	mailed
	My Health Form	mailed	mailed
	Participant Contact Information Form	X	X
	Standard BP Management Form 1M, 6M, 12M, 24M, ... and 72M Visits	X[b]	
	Women's Health	mailed	mailed

[a]Based upon local and state regulations, some medications may be able to be mailed to participant
[b]If equipment (OMRON and/or ECG) is available
[c]Completion of the Event Ascertainment Form is the highest priority
[d]Complete all items on form except question 2
[e]Complete all items on form except questions 2 and 3 (Annual History and Physical Exam Form) or question 2, 3, and 8 – 11 (Annual History and Physical Exam Form with 4 Meter Walk)

Appendix 3C.2
Centrifugation of SPRINT Blood Collection Tubes using a Portable Centrifuge

Biospecimens can be collected at the participant's home. The blood collection tubes need to be centrifuged within 1 hour of the draw. Longer delays in centrifugation will result in falsely elevated and falsely decreased lab values (example: falsely elevated potassium values and falsely decreased glucose values).

Small portable centrifuges are an option for sites to purchase if centrifuging the blood samples within 1 hour of collection is not possible. The portable centrifuge can be used with a portable power system or can be plugged directly into a conventional grounded outlet.

Portable centrifuge option: Fischer Scientific. Fischer Centrific Model 228, 115 V. Catalog ID 29104. Product ID 618075. Price $755.
http://www.fishersci.com/ecomm/servlet/fsproductdetail?catalogId=29104&productId=618075&langId=-1&storeId=10652&distype=2&isChemical=false&fromSearch=0
Field personnel should have a portable centrifuge for their use in the general area where the blood collection is to be performed. The recommended centrifuge is the Fisher Centrific Model 228. This unit operates at one, fixed speed. Other manufacturers and models are acceptable, but the specimens must be centrifuged at a minimum of 1,200 rcf (*g*-force) for 15 minutes. The centrifuge requires an external power source. This source may be the standard electrical wall receptacles within the participant's residence that supply the usual AC voltage and Hertz (in the US: 120 volts AC at 60 cps; in Europe: 230 volts AC at 50 cps). If the participant is unwilling to allow blood centrifugation in their residence, the electrical power can be provided from a rechargeable battery pack with a DC to AC power inverter appropriate for the centrifuge.

OPTION 1: Using electricity within the participant's residence
Before beginning the venipuncture procedure, ask the participant if he or she will allow the centrifuge to be plugged in and operated within their residence. The power requirements of the centrifuge are very minimal, and pose no threat to their electrical system. If the participant is uncomfortable with having the centrifuge in their kitchen or living area, offer to locate it in a bathroom, storage area or basement. Bring a square piece of plywood or large cutting board to serve as a base for the centrifuge. Bring a grounded (3-prong) extension cord in case the electrical receptacle is some distance from a convenient centrifugation site.

Procedure:

1. Place the centrifuge on a firm surface, plywood, or cutting board.
2. Plug the centrifuge into a wall outlet. Use the grounded extension cord if necessary.
3. Load the blood collection tubes to be centrifuged into the rotor per the instructions in the SPRINT MOP Chapter 7, Central Lab, Section 4, Blood and Urine Processing, pages 16-26. The tubes must be balanced for safe centrifuge operation. Close the lid on the centrifuge, and lock the latch. For safety reasons, the centrifuge will not operate unless the lid is closed and locked shut.
4. Turn the timer to 15 minutes. Centrifugation will begin and will stop automatically after 15 minutes.
5. After the centrifuge automatically shuts off, remove the tubes, wrap in paper towels, place in biohazard bag, and place on frozen gel pack inside shipping container. Transport samples back to the clinic and continue blood processing for shipment as described in the SPRINT MOP Chapter 7, Central Lab protocol.

OPTION 2: Using a Portable Power Supply
If the participant will not allow the blood specimens to be centrifuged in their residence, it will be necessary to plug the centrifuge into a portable power pack. The power pack should typically be stored in the trunk of the phlebotomy technician's vehicle with the portable centrifuge, where the centrifugation can be performed if necessary. The recommended power supply is the Powerpack 300 Plus or the 400 Plus, manufactured by Xantrex International, Inc. It may be purchased through the manufacturer's website http://110220volts.com. The Powerpack 300 or 400 Plus contains a rechargeable (from an AC wall receptacle or a vehicle's DC source) 12-volt DC lead-acid battery, which is similar to a typical car battery, and a DC to AC power inverter. US and European centrifuges require different AC voltages and frequencies (Hertz or cps), so the US power pack cannot be used with the European centrifuges or vice versa. After the battery is fully charged, the centrifuge may be plugged directly into the grounded outlet on the Powerpack.

Read the complete Powerpack 300 or 400 Plus Owner's Guide before use. The Powerpack will automatically shut off if excessive surge power is drawn or if the DC battery's voltage falls below 10 volts. The Powerpack 300 or 400 Plus will produce an audible alarm when the battery charge reaches 10.7 volts, indicating that it is getting very low on power and needs recharging as soon as possible.

Procedure:

1. Fully charge the battery. Upon receipt, the initial charge may require up to 40 hours while plugged into a standard electrical outlet. The Powerpack 300 or 400 Plus has a display feature that indicates the status of battery charge. A fully charged battery is capable of completing at least five 15-minute centrifugation sequences. The Powerpack 300 or 400 Plus should be recharged each night to ensure adequate power for the following day's work.
2. After collecting the specimens bring the blood collection tubes to the vehicle for centrifugation.
3. Make sure the centrifuge is in a stable position.
4. Turn the Powerpack 300 or 400 Plus' AC outlet switch ON.
5. Plug the centrifuge into the grounded outlet on the Powerpack 300 or 400 Plus below the switch.
6. Load the tubes to be centrifuged into the rotor per the instructions in the SPRINT MOP Chapter 7, Central Lab, Section 4 Blood and Urine Processing, pages 16-26. The tubes must be balanced for safe centrifuge operation as usual. Close the lid on the centrifuge and lock the latch. For safety reasons, the centrifuge will not operate unless the lid is latched is not closed and locked shut.
7. Turn the timer to 15 minutes. Centrifugation will begin automatically.
8. After the centrifuge shuts off, turn the AC outlet switch OFF.
9. Remove the tubes, wrap in paper towels, place in biohazard bag, and place on frozen gel pack inside shipping container. Transport samples back to the clinic and continue blood/urine processing for shipment as described in the SPRINT MOP Chapter 7, Central Lab protocol.
10. Recharge the battery overnight.

Appendix 3C.3

Central Phone Assessment Flow Chart

Participant has not completed MIND battery (past 27M or 51M window)

→ On Central Call list for MIND

Participant does not complete Event Ascertainment Form for two consecutive visits (past 27M window)

→ On Central Call list for Events

→ Participant will appear on Report >> Clinical Operations >> Telephone Battery Participants – Site Level

→ Clinic staff should ensure Participant Contact Information is up to date and any relevant information is placed in the participant notes

→ Central callers review participant notes and make up to 4 attempts to contact the participant

Successful call: MIND tests

Central callers will:

1. Make a note in the participant notes section
2. Update status to: call completed
3. Data enter completed forms
4. Scan forms and upload to SPRINT website

Clinic staff should:

1. Review participant notes section
2. Print source notes (Data Management >> Download Central PDFs)
3. **Complete MIND testing at next face to face clinic visit**

Successful call: event ascertainment

Central callers will:

1. Make a note in the participant notes section
2. Update status to: call completed
3. Data enter completed forms
4. Scan forms and upload to SPRINT website
5. See Central Event Collection Flow Chart

Clinic staff should:

1. Review participant notes section
2. Print source notes (Data Management >> Download Central PDFs)
3. See Central Event Collection Flow Chart

Discover participant is unavailable because of hospitalization, nursing home/skilled nursing facility or passed away

Central callers will:

1. Make a note in the participant notes section
2. Update status to: call completed
3. See Central Event Collection Flow Chart

Clinic staff should:

1. Review participant notes section
2. See Central Event Collection Flow Chart

Unsuccessful call

Central callers will:

1. Make a note in the participant notes section
2. Update status to: participant refused; max calls made; do not call; contact information missing; contact information incorrect

Clinic staff should:

1. Review participant notes section
2. Update contact information if necessary

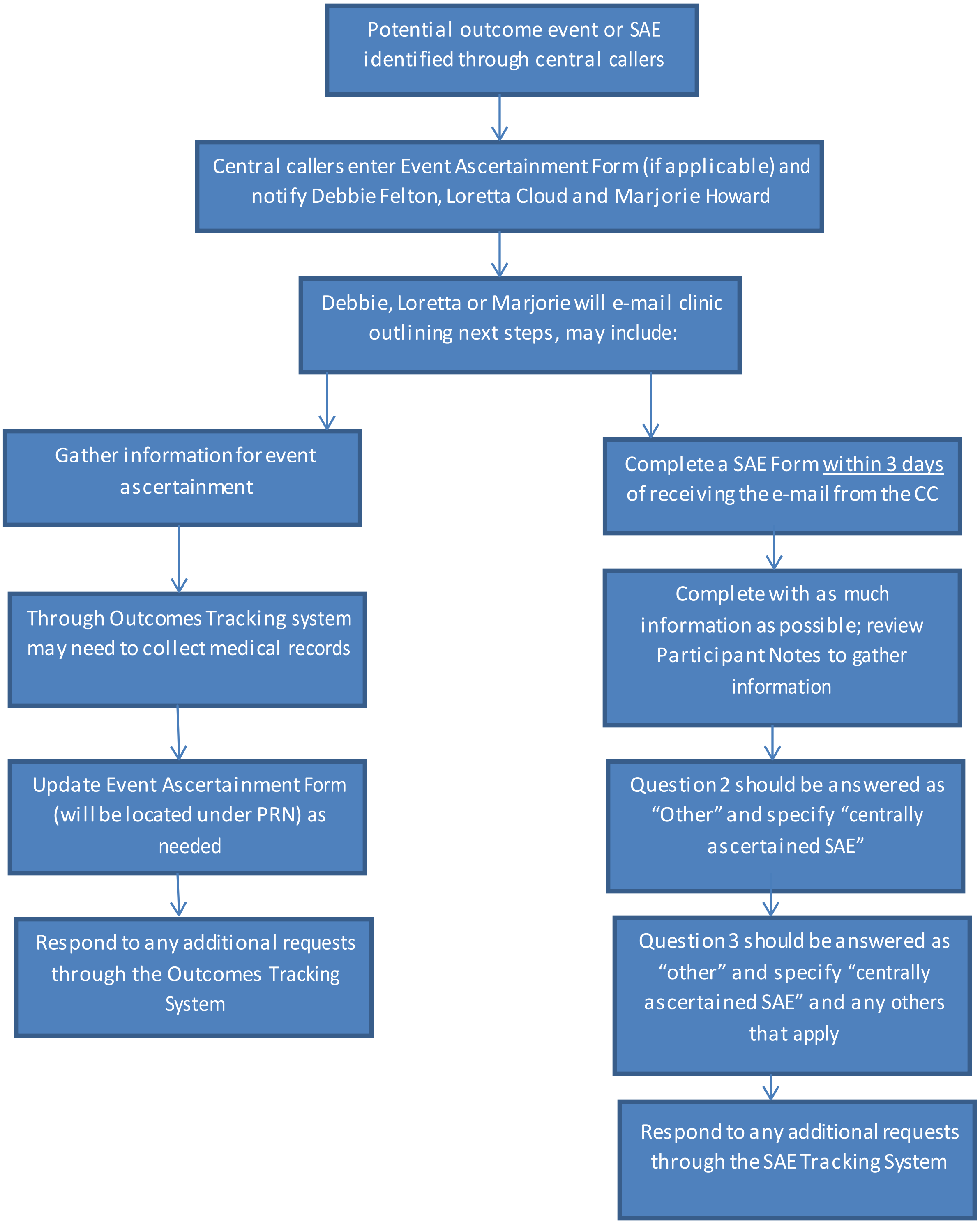

Potential outcome event or SAE identified through central callers
Central callers enter Event Ascertainment Form (if applicable) and notify Debbie Felton, Loretta Cloud and Marjorie Howard
Debbie, Loretta or Marjorie will e-mail clinic outlining next steps, may include:
Gather information for event ascertainment
Through Outcomes Tracking system may need to collect medical records
Update Event Ascertainment Form (will be located under PRN) as needed
Respond to any additional requests through the Outcomes Tracking System
Complete a SAE Form within 3 days of receiving the e-mail from the CC
Complete with as much information as possible; review Participant Notes to gather information
Question 2 should be answered as "Other" and specify "centrally ascertained SAE"
Question 3 should be answered as "other" and specify "centrally ascertained SAE" and any others that apply
Respond to any additional requests through the SAE Tracking System

Chapter 3.d Health Related Quality of Life Procedures

A. Introduction

This chapter of the Manual of Procedures (MOP) describes the procedures for the assessment of health-related quality of life (HRQL) in SPRINT. The overall goal of these assessments is to examine the short-term and long-term impact of the standard and intensive interventions upon HRQL among the total sample, as well as in specific subsamples.

B. Rationale

The SPRINT HRQL instruments were selected based upon the following criteria:

(1) inclusion of the major dimensions shown in the literature to be affected by hypertension and its treatment;
(2) brevity;
(3) responsiveness to treatment-related changes; and
(4) appropriateness for the age range and racial/ethnic diversity in SPRINT.

The following HRQL dimensions have been selected for study in SPRINT: (1) general HRQL; (2) event-related HRQL; (3) depressive symptoms; (4) falls efficacy; (5) sexual function; and (6) participant satisfaction.

C. General Mode of Administration

All assessments will be self-administered in private areas with appropriate oversight provided by trained clinic staff. It is suggested that the HRQL forms be completed before the medical, cognitive function, and physical performance exams, because these exams may potentially influence participants' perceptions of HRQL. Also, because some of the instruments query participants regarding sensitive information (e.g. sexual function), it is suggested that the questionnaires be completed in a private area. After the forms have been completed, clinic staff will review the forms for completeness and data quality before the participant leaves the location.

The core assessments are designed to take approximately 10 minutes to complete. In addition, all HRQL instruments will be self-administered, which is designed to minimize staff burden. Also, the instruments are closed-ended, which should minimize burden on participants and staff during data entry. However, it is reasonable to assume that some participants may take longer to complete the assessments due to factors such as low literacy, poor vision, or discomfort with answering certain questions. Thus, it is recommended that a 10-minute break be offered if a participant is still completing the assessments after 30 minutes. For a small percentage of participants, it may be necessary to administer some forms via interview if poor vision or poor comprehension impairs the participant's ability to complete the assessment.

Next, the health-related quality of life measures used in SPRINT will be introduced and discussed, in their recommended order of administration.

D. Health-Related Quality of Life Instruments

D.i. Summary of Questionnaires and Frequency of Administration

The HRQL instruments are packaged into four forms packets for SPRINT. The groupings follow a similar frequency of administration and participant subset (where relevant).

(1) My Health
   a. Veterans RAND 12 (VR-12)
   b. EuroQol-5D (EQ-5D)
   c. Patient Health Questionnaire (PHQ-9)

(2) Women's Health
   a. Female Sexual Function Index (FSFI)

(3) Men's Health
   a. International Index of Erectile Function (IIEF)

(4) Fall Self-Efficacy Scale International (FES-I)

The My Health packet is administered to the entire SPRINT cohort at baseline and annually thereafter. The FSFI will be administered to a subset of at least 500 women and the IIEF will be administered to a subset of at least 500 men. These two forms will be administered at the 6 month visit and annually thereafter. The FES-I will be administered to the same subset of at least 500 women and at least 500 men and to all participants 75 years or older.

D.ii. HRQL Dimensions and Instruments

General HRQL

The **Veterans RAND 12 (VR-12)** is a shorter version of the VR-36, which was derived from the SF-36. Changes of the VR-12 relative to the SF-12 have lowered the floor and ceiling, improved the distributional properties, increased reliability, and improved discriminant validity of the physical and mental health summary scores. Validated conversion formulas allow for direct comparisons to prior studies using the SF-36 or SF-12.

Health Utility

The **EuroQol-5D (EQ-5D)**[1] includes five items that assess mobility, self-care, usual activities, pain/discomfort and depression. There are three responses to each question (no, moderate, or severe limitations).

Depressive symptoms

The **Patient Health Questionnaire (PHQ-9)**[2] is a self-report measure of depressive symptoms over the previous 2 weeks that has been recommended by the AHA Advisory Panel on Depression and Coronary Heart Disease. It has a low response burden (9 items; 2-3 minutes to complete), excellent reliability, and good sensitivity and specificity with depression diagnoses. To assess depression in the SPRINT participants, the PHQ-9 will be administered annually in all patients. Despite the strong psychometric properties of the PHQ-9, it must be noted that the

scale has significant somatic item overlap, including items assessing trouble sleeping, feeling tired, poor appetite, trouble concentrating, and moving or speaking slowly which could be the result of treatment side effects and/or various medical events in the SPRINT trial.

> *<u>Assessment of suicidal ideation</u>. If the response to question 9 ("Thoughts that you would be better off dead or of hurting yourself in some way") is scored as 2 or 3 ("More than half the days" or "Nearly every day", respectively), immediate action is required.* ***Refer to the Safety MOP Chapter 14 for details on actions that must be taken.***

<u>Falls Self-Efficacy, Occurrence and Fall-Related Injury</u>

The **Fall Self-Efficacy Scale International (FES-I)**[3]**,** shortened version, consists of seven activities which the respondent answers on a 1-4 scale, indicating level of concern for falling. The activities are getting dressed or undressed, taking a bath or shower, getting in or out of a chair, going up or down stairs, reaching for something above your head or on the ground, walking up or down a slope, and getting out to a social event. An evaluation of the Short FES-I found good internal and 4-week test-retest reliability. The correlation between the Short FES-I and the FES-I was 0.97. These results suggest that the short instrument has good psychometric properties that can be completed in 1-2 minutes.

Reports of actual falls will be collected from all participants every three months using the Event Ascertainment Form. The form queries for falls in which the participant landed on the floor or ground or hit an object like a table or stair, and how many of these falls resulted in an injury.

<u>Sexual function (women)</u>

A modified version of the **Female Sexual Function Index (FSFI)**[4] will be administered to assess sexual function among a subset of at least 500 women. The FSFI is a 19-item survey that assesses female sexual function over the past 4 weeks in 6 domains, including desire, arousal, lubrication, orgasm, satisfaction, and pain, as well as a full scale score. Individual domain scores are obtained by adding the scores of the individual items in each domain and multiplying the sum by its associated domain factor. The full scale score is computed as the sum of the six domain scores. A domain score of 0 indicates that no sexual activity was reported during the past month, and higher scores indicate better sexual function. A score of <26.55 is commonly used as a cutoff score to indicate sexual dysfunction[5]. The FSFI domains have high internal consistency (Cronbach alpha > 0.8). The FSFI takes approximately 5 minutes to complete.

<u>Sexual function (men)</u>

The **International Index of Erectile Function (IIEF)**[6] will be administered to assess sexual function among a subset of at least 500 men. The IIEF is the 5-item short form of the original 15-item IIEF, and was developed specifically for use in clinical settings to supplement physical examination and patient history. Each item on the IIEF-5 has a possible range of 1 to 5. The scoring for the IIEF-5 is straightforward, and is the sum of the 5 questions, with a possible total score ranging from 5 to 25. Higher scores indicate better function. IIEF-5 scores can be classified into the following categories; severe ED (5 to 7), moderate ED (8 to 11), mild to moderate ED (12 to 16), mild (17-21) or no ED (22 to 25). Scores less than 21 have 98%

sensitivity and 88% specificity for the presence of ED. The IIEF-5 takes approximately 2 minutes to complete.

Participant Satisfaction

Participant satisfaction will be assessed using three items as found in the Morisky Medication Adherence Scale. This instrument is administered at the RZ2, 12 month and 48 month visits (or as needed) to all participants. The items refer to (1) satisfaction with the medications you have received for your blood pressure, and (2) satisfaction with the care you have received for your blood pressure. Each item is scored on a 5-point Likert scale, ranging from 1 ("Very Satisfied") to 5 ("Very Dissatisfied").

Reference List

(1) Rabin R, de Charro F. EQ-5D: a measure of health status from the EuroQol Group. *Annals of Medicine* 2001;33(5):337-343.

(2) Kroenke K, Spitzer RL, Williams JB. The PHQ-9: validity of a brief depression severity measure. *J Gen Intern Med* 2001;16(9):606-613.

(3) Yardley L, Beyer N, Hauer K, Kempen G, Pilot-Ziegler C, Todd C. Development and initial validation of the Falls Efficacy Scale-International (FES-I). *Age and Aging* 2005;34(6):614-619.

(4) Rosen R, Brown C, Heiman J et al. The female sexual function index (FSFI): a multidimensional self-report instrument for the assessment of female sexual function. *Journal of Sex & Marital Therapy* 2000;26(2):191-208.

(5) Wiegel M, Meston C, Rosen R. The female sexual function index (FSFI): cross-validation and development of clinical cutoff scores. *Journal of Sex and Marital Therapy* 2005;31(1):1-20.

(6) Rosen RC, Cappelleri JC, Smith MD, Lipsky J, Pena BM. Development and evaluation of an abridged, 5-item version of the International Index of Erectile Function (IIEF-5) as a diagnostic tool for erectile dysfunction. *International Journal of Impotence Research* 1999;11(6):319-326.

COGNITION ASSESSMENT PROCEDURES: SPRINT-MIND

INTRODUCTION

SPRINT-MIND is an integral part of the overall SPRINT study and all SPRINT participants will participate in one or more components of SPRINT-MIND. There are three objectives of SPRINT-MIND:

1. To determine whether a strategy of intensive blood pressure lowering to target systolic blood pressure (SBP) <120 mm Hg versus a standard treatment target of 140 mm Hg will result in a lower incidence of ***all-cause dementia***.
2. To determine whether ***global cognitive function,*** measured in key specific domains of cognition, will decline less in persons randomized to a SBP goal of <120 mm Hg versus a standard treatment goal of 140 mm Hg in a representative sub-sample of approximately 2800 SPRINT participants.
3. To assess whether MRI-derived ***changes in brain structure*** differ by treatment assignment in a subset (approximately 640) of the 2800 participants.

We will ascertain incident all-cause dementia in all participants enrolled in SPRINT at 24 months, 48 months and closeout if testing has not occurred in the past 12 months. In addition, approximately 2800 participants will be selected to receive additional cognitive assessments at baseline, 24 months, and 48 months in order to examine changes in global and domain-specific cognition. Participants that reside within approximately 1.5 hours of a designated study MRI Scanner will be screened for eligibility, enrolled in the MRI substudy, and receive a scan at baseline and 48 months.

GUIDELINES FOR COGNITIVE TEST ADMINISTRATION

In order for SPRINT to capture cognitive data that are valid and useable, it is imperative that examiners follow a standardized procedure when administering and scoring the test battery. The way in which the testing is administered to a participant can affect the validity of the responses. Therefore, it is critical that the examiner adhere strictly to the written guidelines, read the instructions verbatim and the following general tips for test administration.

Standardizing Administration

Variability in administration introduces bias in data collection. Testing guidelines are established so that each examiner administers the instruments in the same way. The examiner’s actions should not bias the participant’s perception or response to a question, and different examiners should be able to obtain the same responses from the same participant. Examiners should think of themselves as part of the instrumentation.

To enhance standardization:

a) Do not provide either explicit or implicit verbal or non-verbal responses that could influence the participant’s responses beyond what is permitted. For example, do not convey surprise, pleasure or disapproval to any answer. The examiner’s role is to obtain honest, uninfluenced responses to the questions.
b) Be thoroughly familiar with the instruments before administering them. This ensures that participant questions or concerns can be easily addressed. Periodic review of the test protocol and occasional practice test administration helps to

reduce ‘rustiness’ or the unsmooth style that sometimes results from being unfamiliar with the material.

c) Convey a sense of impartiality. Be gracious and supportive of all participants.
d) Read all directions to the participant verbatim, both sample and actual test directions.
e) Administer all tests in the order in which they are presented. ***<u>The sequence of the tests is important.</u>***
f) Write the participant’s responses on the test forms as they are given. Recording each test administration is required. This provides back-up for scoring should you fall behind during the session. Never depend on your memory to record the participant’s choices after the test session is over. <u>Retain these recordings at the site for at least 30 days.</u> The SPRINT MIND CC will notify the site during that time if the administration has been selected for QC review.
g) Keep explanations to a minimum. Avoid interpreting or paraphrasing instructions or interview questions--it is easy to unintentionally alter the meaning in this way.
h) Offer generic phrases of encouragement during and between tests (e.g., “You are doing fine”) but do not offer any information on the correctness of a particular response (including nonverbal communication like nodding or shaking your head). If participants ask how they are doing, explain that you are not permitted to give feedback.
i) Do not offer any assistance in completing tasks that are not specifically permitted in the instructions.
j) Do not place score sheets where the participant can see them. Use a clipboard or other device to shield the results.
k) Never suggest an answer or disagree with a response. The examiner’s role is to obtain and record the ***participant’s*** responses.
l) If the participant is having trouble hearing you, adjust your volume, speak more slowly and ask him/her to listen carefully to your instructions. You can repeat this reminder in between tests if you think the participant is not hearing adequately or isn’t paying close attention. Be sure to make a comment on the cover page of the screening battery forms that indicates that the participant had difficulty hearing.
m) Once you begin reading the instructions for a specific task, you may pause and ask the participant if s/he has any questions. If they did not hear you or asks for clarification, you may repeat the instructions verbatim.
n) If the participant loses track of the instructions **<u>during the task</u>** and asks for help, you should say “I’m sorry, but I am not allowed to repeat the instructions.” Do <u>not</u> repeat stories, word lists or digits.
o) The participant must be administered the MIND tests in the same language each time, i.e., if MIND was administered in Spanish at the Baseline visit, it must be administered in Spanish at 24 and 48 month visits.
p) Anytime the MIND cover page prints, it should be completed and data entered, regardless of whether or not the MIND tests were completed.
    1) If the participant successfully completes a test, the cover page should be marked as YES the test was completed.
    2) If the participant does not attempt a test (i.e., vision, refuses all MIND testing, doesn’t have time at visit, etc), the cover page should be marked as NO the test was not completed and a reason should be noted.

3) If the participant cannot successfully complete the sample items (Digit Symbol, Trails) and the scored portions are not administered, then the cover page should be marked as NO the test was not completed and a note provided that the participant could not complete the sample items correctly.
4) If the participant attempts the scored/timed portion of a test, but refuses to complete it, the cover page should be marked as YES the test was completed and a score should be entered. A note should also be included that the participant refused to complete the test.

General Instructions

a) Prior to each administration, review the test materials to refresh your memory as to the correct procedures.
b) Ask if the participant uses glasses, hearing aids or other assistive devices. If s/he does, request that s/he use them.
c) Choose a quiet and private area in the clinic to conduct the cognitive testing.
d) Put a sign on the door such as **"Quiet Please: Testing in Process"** to minimize interruptions.
e) If there is a phone in the room, turn it off or unplug it. Also ask the participant to turn off any cell phone or pager.
f) Assemble testing materials and equipment before beginning the testing. Check your equipment (recorder and stopwatch) to make sure the batteries are good. Keep extra batteries in the testing area.
g) You may highlight portions of the test forms to remind you to do things such as check time or remember to give a prompt.
h) Have a relaxed and friendly manner. This will help put the participant at ease and convey the importance of the testing. Spend a few minutes putting the participant at ease with light conversation.
i) Be patient and polite. Convey a sense that the participant's answers are important. Do not rush.
j) Observe the participant for signs of strain or stress (e.g., becomes nervous, puts less effort into tasks, and starts making excuses). Try to help the participant relax with generic reassurances and encouragement (e.g., "You're doing fine" or "These tasks are designed to be challenging, so don't worry about making mistakes, just do the best you can."). If the participant needs a break, it can be taken in between batteries (after the screening battery is complete), but not during or in between tests.
    - If you continue to notice signs of stress or anything unusual during the testing, note this information on the cover page so that if the participant goes to adjudication, the adjudicators will be aware.
k) Reassure the participant that their answers will be kept confidential, if they ask.
l) Family members or friends may accompany the participant to the clinic. They should <u>not</u> be present during test administration. Explain to the friend or family member the necessity of providing privacy and confidentiality, and be prepared to suggest a place where they can wait comfortably during testing.

Additional Suggestions for Preserving Standardization

Use these specific words to offer as encouragement for ALL participants:

a) ***"Just do the best that you can. As long as you do the best that you can, you are doing what we want you to do."***
b) ***"These tasks are designed so that no one gets all the answers correct."***

COGNITIVE ASSESSMENT TESTING ORDER

SPRINT-MIND Screening Battery
Administered to all SPRINT participants at Baseline, 24 months, 48 months and closeout visit *:
1. Logical Memory - Immediate Recall
2. Montreal Cognitive Assessment (MoCA)
3. Digit Symbol Coding (DSC)
4. Logical Memory – Delayed Recall
   * closeout administration unless cognitive testing has been administered in the past 12 months

SPRINT-MIND Extended Battery
Administered to the 2800 participants in the general cognitive function assessment substudy at Baseline, 24 months and 48 months. Also administered during follow-up (24 months and 48 months) to all who "trip" the Screening Battery at that time (score below specified cut points on the MoCA):
1. Hopkins Verbal Learning Test (HVLT) – Learning Trials 1-3
2. Trail Making Test: Parts A and B
3. Boston Naming Test
4. Modified Rey-Osterrieth (Rey-O)
5. Category Fluency-Animals
6. Digit Span Forward and Backward
7. Hopkins Verbal Learning Test – Delayed Recall and Delayed Recognition

MATERIALS NEEDED FOR TESTING
Screening Battery
Have in place and arrange all of the following prior to each test session:
- Test forms printed from the SPRINT Website
- Test administration script book (provided by the SPRINT-MIND CC)
- Participant worksheets for MoCA and DSC (mailed from the SPRINT-MIND CC)
- Digital recorder
- Sharpened pencils without erasers
- Clipboard
- Stopwatch
- Laminated testing sheets MoCA
- Digit Symbol Coding scoring template

Extended Battery
Have in place and arrange all of the following prior to each test session:
- Test forms printed from the SPRINT Website
- Test administration script book (provided by the SPRINT-MIND CC)
- Participant worksheets for Trails A & B (mailed from the SPRINT-MIND CC)
- HVLT form (mailed from the SPRINT-MIND CC)

- Digital recorder
- Clipboard
- Stopwatch
- Boston Naming Stimulus book
- Laminated figure for Rey-O drawing
- Black Pen
- Sharpened pencils without erasers

INSTRUCTIONS FOR SPECIFIC SPRINT-MIND ASSESSMENTS

The SPRINT-MIND Screening Battery

Introducing the Test Battery
Examiner: ***"During this part of the visit I am going to have you do a series of tasks that may challenge your memory and other thinking abilities. You will not get every item correct, so don't worry about how you are doing. So that everyone has the same amount of time to work, you will notice you are being timed on some of the tests. Again, just relax and do the best you can on each and every one."***

LOGICAL MEMORY (LM) Test

Description
The LM test is a sub-test of the Wechsler Memory Scale-IV. It measures episodic verbal memory. Participants are read a short story that consists of 14 bits of information and immediately asked to recall as many bits as possible. The story is repeated and recall is tested. After a delay of approximately 15 minutes, participants are asked to recall as much information from the story as possible. (Do the best you can to retest as close to 15 minutes as possible but always do the recall.) The total score is the total bits of information recalled according to a standard checklist. Scores can range from 0 to 14 on each recall.

Materials Needed
- Test forms printed from SPRINT Website
- A watch or clock for noting the time at the end of the second recall
- Digital recorder

Administration
Prior to reading the Story, say "***I am going to read a short story to you. Listen carefully and try to remember it just the way I say it, as close to the same words as you can remember. When I am through, I want you to tell me everything I read to you. You should tell me all you can remember even if you are not sure. Ready?"***

The SPRINT examiner should read the story at a normal pace, being careful to enunciate clearly. After reading the story, say ***"Tell me everything you can remember about this story. Start at the beginning."*** Write out the participant's

response exactly as s/he says it or afterwards from the tape recording to ensure accurate scoring. Space is provided on the test form.

After the participant has repeated as much of the story as they can remember, proceed to Second Recall. Say, ***"I am going to read the same story I just read to you one more time. Try to remember it just the way I read it. Ready?"***

Read the story a second time and say, ***"Tell me everything you can remember about this story. Start at the beginning."*** Write out the participant's response exactly as s/he says it or afterwards from the tape recording to ensure accurate scoring. Space is provided on the test form.

When the participant has finished with the second recall, note the time. There is to be an approximate 15 minute delay between the second recall and the delayed recall at the end of the Screening Battery (after the MoCA and the DSC).

For the Delayed Recall DO NOT read the story to the participant. Say, ***"Do you remember that story I read to you a little while ago? I want you to tell me the story again. Tell me everything that you can remember. Start at the beginning."*** Write out the participant's response exactly as s/he says it or afterwards from the tape recording to ensure accurate scoring. Space is provided on the test form.

If the participant does not recall any of the story details, say, ***"The story was about a man and a woman."*** Place a checkmark (√) in the *Story cue given* box on the form. Do not give any further help other than general encouragement.

Scoring

Score 1 point for each correctly recalled story detail. Score 0 points for each incorrectly recalled or omitted story detail. Circle either the 1 or 0 for accurate scoring.

If the participant cannot recall any part of the story or does not give a response, a score of 0 should be entered. You will get a query for the 0 but you should just confirm that it is correct.

The scoring criteria for each detail are listed on the form and examples of 1-point and 0-point responses are listed below. To receive a score of 1, the participant must give a response that meets the scoring criteria. The scoring criteria for each detail may or may not allow variations from the verbatim story detail.

Examples of responses and possible scores:

Item	Story Detail	1-Point Criteria	Example of 1-Point Response	Example of 0-Point Response
1.	Ruth	*Ruth* or Ruthie or variant	Ruthie	Ruby, Rudy, any other name
2.	and Paul	*Paul* or Paulie or variant	Paulie	Feminine versions such as Paula or Pauline, any other name

3.	have been friends	*friends* (in any context)	who were friends, they are friends	they were married, they worked together, any other relationship
4.	for thirty	*thirty* (in any context) is required, reference to years is not required	30 days	any other numeric response
5.	years.	*year(s)* is required, reference to specific number of years is not required	knew each other for years, friends for years, friends for a few years	knew each other for days/months, knew each other for a long time.
6.	They meet	Any indication of intentionally getting together	get together, hang out, see each other	run into each other, accidentally met, they go, or any indication the two characters do not intentionally meet
7.	every	*every* (**<u>in reference to Tuesday only</u>**) or *on Tuesdays*	every Tuesday, Tuesday's, each Tuesday, every week on Tuesday	every week, meet often, every day, every Thursday
8.	Tuesday	*Tuesday(s)* (in any context) is required	Tuesday	any other day of the week
9.	at Alma's	*Alma's* (in any context) is required	Alma's Diner, Alma's Restaurant, Alma's Place	any other name
10.	Diner	*Diner* is required	Diner	Restaurant, Place, Café
11.	for breakfast	*breakfast* (in any context) is required	eat breakfast, have breakfast, break the fast	lunch, dinner, any other meal, to each with no reference to a type of meal
12.	and then they go for a walk	indication that they walk	take a stroll, walk around	go for a jog/run, any other activity
13.	in Mason	*Mason* (in any context) is required	Mason Diner, Mason Zoo, Mason Lake	any other name
14.	Park.	*Park* (in any context) is required	Mason Park, Alma's park, the park	any other place

The points for each recall are totaled and that number is entered on the form and into the SPRINT database.

<u>The MONTREAL COGNITIVE ASSESSMENT (MoCA) Test</u>

Description

The Montreal Cognitive Assessment (MoCA) was designed as a rapid screening instrument of global cognitive function. It assesses different cognitive domains: attention and concentration, executive functions, naming, memory, language, visuoconstructional skills, conceptual thinking, calculations, and orientation. Time to administer the MoCA is approximately 10 minutes. The total possible score is 30 points.

Materials Needed

- Laminated Animals Picture
- Participant worksheet (mailed from the SPRINT-MIND CC)
- Test forms printed from SPRINT Website
- Pencil without an eraser

Administration and Scoring

1. **Visuospatial/Executive**

For the first three tasks, place the Participant MoCA Worksheet and a pencil without an eraser on a table in front of the participant.

A. **Alternating Trail Making**

Administration

The examiner says: ***"Please draw a line, going from a number to a letter in ascending order. Begin here*** [point to (1)] ***and draw a line from 1 then to A then to 2 and so on. End here*** [point to (E)]**."** You may repeat these instructions only twice for clarification purposes.

Scoring

Allocate one point if the participant successfully draws the following pattern, without drawing any lines that cross:

1 -A- 2- B- 3- C- 4- D- 5- E

The participant must begin at 1 and continue correctly to receive a score of 1. Any error that is not immediately self-corrected earns a score of 0.

B. **Copying a Cube**

Administration

The examiner gives the following instructions, pointing to the cube: ***"Copy this drawing as accurately as you can, in the space below."***

Scoring

One point is allocated for a correctly executed drawing.

- Drawing must be three-dimensional
- All lines are drawn
- No line is added
- Lines are relatively parallel and their length is similar (rectangular prisms are accepted)
- Minor overshoots are permissible.

A point is not assigned if any of the above criteria are not met.

C. **Drawing a Clock**

Administration

Indicate the right third of the space and give the following instructions:
"Draw a clock. Put in all the numbers and set the time to 10 after 11."

Scoring

One point is allocated for each of the following three criteria:

- Contour (1 pt.): the clock face must be a circle with only minor distortion acceptable (e.g., slight imperfection on closing the circle);
- Numbers (1 pt.): all clock numbers must be present with no additional numbers; numbers must be in the correct order and placed in the

approximate quadrants on the clock face; Roman numerals are acceptable; numbers can be placed outside the circle contour;

- Hands (1 pt.): there must be two hands jointly indicating the correct time; the hour hand must be clearly shorter than the minute hand; hands must be centered within the clock face with their junction close to the clock center.

A point is not assigned for a given element if any of the above criteria are not met.

The total score for these three tests are added and entered on the test form.

2. **Naming**

Administration

Place the laminated animals picture in front of the participant and beginning on the left, point to each figure and say: ***"Tell me the name of this animal."***

Scoring

One point each is given for the following responses: (1) lion, (2) rhinoceros or rhino (3) camel or dromedary.

The scores are added and entered on the test form.

3. **Memory**

Administration

The examiner reads a list of 5 words at a rate of one per second, after giving the following instructions: ***"This is a memory test. I am going to read a list of words that you will have to remember now and later on. Listen carefully. When I am through, tell me as many words as you can remember. It doesn't matter in what order you say them."*** Place a check in the allocated space for each word the participant produces on this first trial. When the participant indicates that they have finished (has recalled all words), or can recall no more words, read the list a second time with the following instructions: ***"I am going to read the same list for a second time. Try to remember and tell me as many words as you can, including words you said the first time."*** Place a check in the allocated space for each word the participant recalls after the second trial.

At the end of the second trial, inform the participant that they will be asked to recall these words again by saying, ***"I will ask you to recall those words again at the end of the test."***

Scoring

No points are given for Trials 1 and 2. The memory score is only given for the delayed recall.

4. **Attention**

A. **Forward Digit Span**

Administration

Give the following instruction: ***"I am going to say some numbers and when I am through, repeat them to me exactly as I said them."*** Read the five number sequence at a rate of one digit per second.

B. **Backward Digit Span**
Administration
Give the following instruction**:** ***"Now I am going to say some more numbers, but when I am through you must repeat them to me in the backwards order."*** Read the three number sequence at a rate of one digit per second.

Scoring
Give one point for each sequence correctly repeated, (Note: the correct response for the backwards trial is 2-4-7).

C. **Vigilance**
Administration
The examiner reads the list of letters at a steady pace of one letter per second, after giving the following instruction: ***"I am going to read a sequence of letters. Every time I say the letter 'A', tap your hand once. If I say a different letter, do not tap your hand."***

Scoring
Give one point if there is zero to one error (an error is a tap on a wrong letter or a failure to tap on letter 'A'). No point is given for two or more errors.

D. **Serial 7s**
Administration
The examiner gives the following instruction: ***"Now, I will ask you to count by subtracting seven from 100, and then, keep subtracting 7 from your answer until I tell you to stop."*** Give this instruction twice if necessary.

Scoring
This item is scored from 0 to 3 points. Give no (0) points for no correct subtractions, 1 point for one correct subtraction, 2 points for two-to-three correct subtractions, and 3 points if the participant successfully makes four or five correct subtractions.

Count each correct subtraction of 7 beginning at 100. Each subtraction is evaluated independently; that is, if the participant responds with an incorrect number but continues to correctly subtract 7 from it, give a point for each correct subtraction. For example, a participant may respond "92 – 85 – 78 – 71 – 64" where the "92" is incorrect, but all subsequent numbers are subtracted correctly. This is one error and the item would be given a score of 3.

It is important to write out the participant's response on the form for scoring after the test session is over.

5. **Sentence Repetition**

Administration

The examiner gives the following instructions: ***"I am going to read you a sentence. Repeat it after me, exactly as I say it*** [pause]: '***I only know that John is the one to help today.'"*** Following the participant's response, say: ***"Now I am going to read you another sentence. Repeat it after me, exactly as I say it*** [pause]: '***The cat always hid under the couch when dogs were in the room.'"***

Scoring

Give 1 point for each sentence correctly repeated. Repetition must be exact. Be alert for errors that are omissions (e.g., omitting "only", "always") and substitutions/ additions (e.g., "John is the one who helped today;" substituting "hides" for "hid", altering plurals, etc.). If you misread the sentence, you should not stop and repeat it. The participant should repeat the sentence exactly as you say it. If you are unsure if the participant repeated the sentence as you said it, review the recording to verify their response.

6. **Verbal Fluency**

Administration

The examiner gives the following instruction: ***"Tell me as many words as you can think of that begin with a certain letter of the alphabet that I will tell you in a moment. You can say any kind of word you want, except for proper nouns (like Bob or Boston), numbers, or words that begin with the same sound but have a different suffix or ending, for example, love, lover, loving. I will tell you to stop after one minute.*** [Pause] ***Are you ready?*** [Pause] ***Now, tell me as many words as you can think of that begin with the letter F.*** (Time for 60 seconds (STOPWATCH WILL READ 1:00). ***Stop."***

NOTE: For Spanish administrations, use the letter "P".

Scoring

Give one point if the participant generates 11 or more unique and valid words in 1 minute. Write the participant's response at the bottom or side margins. Examples of words that should not be counted towards the total include:

- Proper Nouns: France, French, Fahrenheit
- Numbers: four, five, first, fourth
- Words with different suffixes or endings.
    - EXCEPTION: If the addition of the suffix changes the meaning of the word, both words may be counted. Examples include friend, friendship; fellow, fellowship.

If the participant gives a word that has multiple meanings and may/may not be allowed (i.e., four/for, frank/Frank), you should ask the participant after the time is up which meaning of the word they intended. You should not tell the participant that the word may or may not be allowed.

7. **Abstraction**

Administration

The examiner asks the participant to explain what each pair of words has in common, starting with the example: ***"Tell me how an orange and a banana are alike."*** If the participant responds **"Fruit,"** move on to the next trial, you do not need to ask for another way those items are alike. If the participant answers in a concrete manner but it is not the correct response, then say only one additional time: ***"Tell me another way in which those items are alike."*** If the participant does not give the appropriate response (fruit), say, ***"Yes, and they are also both fruit."*** Do not give any additional instructions or clarification.

After the practice trial, say: ***"Now, tell me how a train and a bicycle are alike."*** Following the response, administer the second trial, saying: ***"Now tell me how a ruler and a watch are alike."*** Do not give any additional instructions or prompts after the practice trial.

Scoring

Only the last two item pairs are scored. Give 1 point to each item pair correctly answered. The following responses are acceptable:

Train-bicycle = means of transportation, means of travelling, you take trips in both;
Ruler-watch = measuring instruments, used to measure.

The following responses are not acceptable:

Train-bicycle = they have wheels, you can ride them, they are a way of moving, they carry people, vehicles
Ruler-watch = they have numbers.

8. **Delayed Recall**

Administration

The examiner gives the following instruction: ***"I read some words to you earlier, which I asked you to remember. Tell me as many of those words as you can remember."*** Place a check mark (√) for each of the words correctly recalled spontaneously without any cues, in the allocated space.

Scoring

Give 1 point for each word recalled freely without any cues.

Following the delayed free recall trial for any word not recalled, prompt the participant with the semantic category cue provided below. Place a check mark (√) in the allocated space if the participant remembered the word with the help of a category or multiple-choice cue. Prompt all non-recalled words in this manner. If the participant does not recall the word after the category cue, give them a multiple choice cue, using the following example instruction, ***"Which of the following words do you think it was, NOSE, FACE, or HAND?"***

Use the following category and/or multiple-choice cues for each word, when appropriate:

FACE:
category: part of the body **multiple choice:** nose, face, hand

VELVET:
category: type of fabric **multiple choice:** denim, cotton, velvet

CHURCH:
category: type of building **multiple choice:** church, school, hospital

DAISY:
category: type of flower **multiple choice:** rose, daisy, tulip

RED:
category: a color **multiple choice:** red, blue, green

Scoring
No points are allocated for words recalled with a cue. A cue is used for clinical information purposes only and can give the test interpreter additional information about the type of memory disorder.

9. **Orientation**
Administration
The examiner gives the following instructions: ***"Tell me the date today."*** If the participant does not give a complete answer, then prompt accordingly by asking specifically for each component: ***"Tell me the year…..Tell me the month….Tell me the exact date….Tell me the day of the week."*** Then say: ***"Now, tell me the name of this place, and which city it is in."***
Scoring
Give one point for each item correctly answered. The participant must tell the exact date (including the name of the month) and the exact place (name of hospital, clinic, office).

It is important to write out the participant's response on the form for scoring after the test session is over.

MoCA Scores:
Enter all MoCA scores listed on the right-hand side of the form into the SPRINT database.

<u>DIGIT SYMBOL CODING (DSC) Test</u>
Description
The DSC assesses sustained attention, concentration, visuo-motor coordination and processing speed. The test consists of 8 rows containing a total of 144 small blank squares, each of which is paired with a randomly assigned number from 1 to 9. Above these rows is a printed key that pairs each number with a different symbol. The participant is asked to fill in the blank spaces with the symbol that is paired to the number as quickly as possible for **2 minutes**.

Materials Needed

- Participant worksheet (mailed from the SPRINT-MIND CC)
- Stopwatch
- Pencil without eraser
- Scoring Template

Specific Instructions for Administering and Scoring the DSC

- Read instructions provided verbatim and demonstrate as indicated.
- Do not proceed with the test until the participant understands and correctly completes the sample items. If the participant makes a mistake on the sample items, correct the error immediately. Instructions can be repeated up to 2 times.
- Begin timing once the sample items are completed and you say ***"Go."***.
- Testing time = 2 minutes.
- Boxes must be done in sequence (left to right). It is important that you position yourself so that you can observe the participant closely. Do not sit on the participant's dominant side (if they are right handed, sit on their left).
- If the participant begins working out of sequence (e.g., doing all of the 1's), redirect them **only** on the first omitted item. Give no further assistance except (if necessary) to remind the participant to continue until instructed to stop.

Administration

1. **Demonstration Items**

   Place the DSC Participant Worksheet in front of the participant. Point to the key at the top of the page and say, ***"Look at these boxes. Each box has a number in the top part*** (point across the numbers from 1 to 9) ***and a special mark in the bottom part*** (point across the symbols). ***Each number has its own mark*** (point to 1 and its symbol, then to 2 and its symbol).**"**

   Point to the demonstration items and say, ***"Down here, the boxes have numbers in the top parts but are empty in the bottom parts. You are to draw the marks that belong in the empty boxes, like this."***

   Point to the first demonstration item (6) and say, ***"Here is a 6. The 6 has this mark*** (point to the key to show its corresponding symbol), ***so I draw that mark in the box, like this*** (write the symbol).**"**

   Point to the second demonstration item (8) and say, ***"Here is an 8. The 8 has this mark*** (point to the key to show its corresponding symbol), ***so I draw that mark in the box*** (write the symbol).**"**

   Point to the third demonstration item (3) and say, ***"Here is a 3. The 3 has this mark*** (point to the key to show its corresponding symbol), ***so I draw that mark in the box*** (write the symbol).**"**

   Proceed to Sample Items.

2. **Sample Items**

   Hand the participant a pencil without an eraser and say, ***"Now you do these*** (point to the sample items). ***Stop when you get to this line*** (point to the heavy line that separates the sample items from the test items).**"**

   Allow the participant to work alone on the remaining sample items. If a left-handed participant partially blocks the key with their left arm while completing the sample items, stop the administration. Place an extra Coding subtest participant worksheet to the right of the participant. Position it so the extra key is aligned with the key the participant's arm is blocking. Have the participant complete the remaining sample items using the extra key so they will be accustomed to the arrangement when completing the test items.

   If the participant completes the sample items correctly, offer praise such as ***"Yes"*** or ***"Right"*** and, finally, ***"Now you know how to do them."***

   If the participant makes a mistake on a sample item, correct the error immediately. Use the item to review the use of the key. Continue to help the participant, if necessary, until the participant correctly completes the sample items. Use explanations such as ***"You see, this is a 9. The 9 has this mark, so I draw that mark in the box*** (write the symbol)**."**

   Do not proceed with the test items until the participant understands the task. If it is clear that the participant will not be able to understand the task with further instruction, discontinue the test and note reason for discontinuation.

   When the participant has successfully completed the sample items, proceed to Test Items.

3. **Test Items**

   Say, ***"When I say 'Go,' do these the same way. Start here*** (point to the first test item), ***go in order, and don't skip any. Work as fast as you can without making mistakes until I tell you to stop. Are you ready?"***

   Explain further if necessary, then say ***"Go."*** Begin timing and allow <u>2:00 minutes</u> (your stopwatch should read 2:00).

   If necessary, remind the participant to go in order and continue working. Give no further assistance.

   If the participant is still working at 2:00 minutes, stop timing and say, ***"Stop."*** Remove the participant worksheet and pencil from the participant's view.

Scoring

1. You should be seated where you can observe the participant closely. If the participant begins to work out of sequence <u>after</u> being redirected the first time do not stop or redirect again. Make a note of the box and once the test is completed and the participant hands the worksheet to you, place an "X" in the box after the

last correctly completed symbol (the symbol to the right of the second skipped box or start of working out of sequence again). Score only to that point.

2. Do not give credit for items that are drawn incorrectly or completed out of sequence (in a clustering or grouping fashion). Any boxes completed after the second "out of sequence" are not counted towards the total.
3. Use the Digit Symbol Coding Scoring Template to check the participant's responses. Line up the template with the boxes on the participant worksheet. Note incorrect symbols by place an X through them. Subtract the number of incorrect symbols from the total number of symbols drawn. Determining the total number is made easy by reading the small number on the template **above** the last symbol drawn to determine the total score.
4. Responses to the sample items are not included in the score.
5. A response is scored as correct if it is clearly identifiable as the keyed symbol, even if it is drawn imperfectly or if it is a spontaneous correction of an incorrect symbol.
6. Record the score on the DSC form and enter this score into the SPRINT database.
7. **Maximum score = 135 points**

SPRINT-MIND Extended Battery

HOPKINS VERBAL LEARNING TEST - REVISED (HVLT-R)

Description

The HVLT-R measures immediate memory span, provides a learning curve, reveals learning strategies or their absence, and short-term and longer-term retention. It consists of three presentations with free recall of a 12-word list, a delayed recall trial (20 minutes after Trial 3), and a delayed recognition.

Materials Needed

- Test Forms (mailed from the SPRINT-MIND CC)
- Pen/pencil
- Watch or clock to note the time at the end of Trial 3

Administration

1. **Free Recall: Trial 1**

Examiner: ***"I am going to read a list of words to you. Listen carefully, because when I am through, I'd like you to tell me as many of the words as you can remember. You can tell them to me in any order. Are you ready?"***

Read the words at the rate of one word every 2 seconds. If the participant does not understand a word, repeat it quickly and try to maintain the 2 second per word pace. Examiner: ***"OK. Now tell me as many of those words as you can remember."***

- Write the words the participant recalls on the form.
- If a word is said that is not in the list (*referred to as an "intrusion"*), write that word on the form, however say nothing to the participant about the word not being on the list.

- **There is no time limit for each recall trial.** However, if the participant does not produce any words for 10-15 seconds, ask the participant if he/she can remember any more words, e.g., "***You still have some time. Can you remember any more words?"***
- If not, move on to Trial 2. Later, you can total the number of words that were correctly repeated.

2. **Free Recall: Trial 2**

   Examiner: ***"Now we are going to try it again. I am going to read the same list of words to you. Listen carefully, and tell me as many of the words as you can remember, in any order, including all the words you told me the first time."***
   - <u>Write the words the participant recalls on the form</u>.
   - If a word is said that is not in the list (*referred to as an "intrusion"*), write that word on the form, however say nothing to the participant about the word not being on the list.
   - **There is no time limit for each recall trial.** However, if the participant does not produce any words for 10-15 seconds, ask if he/she can remember any more words.
   - If not, move on to Trial 3. Later, you can total the number of words that were correctly repeated.

3. **Free Recall: Trial 3**

   Examiner: ***"I am going to read the list one more time. As before, I'd like you to tell me as many of the words as you can remember, in any order, including all the words you've already told me."***
   - <u>Write the words the participant recalls on the form</u>.
   - If a word is said that is not in the list (*referred to as an "intrusion"*), write that word on the form, however say nothing to the participant about the word not being on the list.
   - **There is no time limit for each recall trial.** However, if the participant does not produce any words for 10-15 seconds, ask if he/she can remember any more words.

   <u>IMPORTANT</u>: ***<u>Do not</u>*** tell the participant that recall of the words will be tested later.

4. **Delayed Recall** (Administered approximately 20 minutes <u>after</u> Free Recall Trial 3)
   - ***<u>DO NOT READ THE WORD LIST AGAIN</u>*****.**
   - If the participant is quick and you reach this section before the 18-20 minute delay has been reached, use the time to make sure that all forms/questions have been answered. You may fill this time with other questionnaires needed for the study. If necessary, you may let the participant know that you need to wait a few minutes before you can administer the last part of the test.
   - If the participant is slower and you reach the 20-25 minute delay and you still have several tests to administer before the delayed recall, you may administer this part of the test before Digit Span Forward. When this happens be sure to make a note on the cover page.

Examiner: ***"Do you remember that list of words you tried to learn before? Tell me as many of those words as you can remember."***

- **There is no time limit for each recall trial.** However, if the participant does not produce any words for 10-15 seconds, ask if he/she can remember any more words.
- If the participant does not remember which list or starts to recite a different list (i.e., FACE, VELVET, CHURCH, DAISY, RED) you may prompt the participant one time using the script provided on the form ***"It was the list that I read to you three times and you were asked to recall the words each time."*** You may include "longer" in your prompt because the HVLT list is longer than the MoCA list.
- If the participant does not recall any words a score of 0 should be entered, then move on to the Delayed Recognition Trial. You will get a query for the 0, but you should confirm that it is correct.

5. **Delayed Recognition Trial**

Examiner: ***"Now I'm going to read a longer list of words to you. Some of them are words from the original list, and some are not. After I read each word, I'd like you to say "Yes" if it was on the original list or "No" if it was not. Was*** [word] ***on the list?"***

- Read the words from the top of the columns down.
- Circle either the **"Y"** (Yes) or **"N"** (No) next to each word to indicate the participant's response.
- The participant must give a response for every word. If they are unsure, ask for a best guess.
- If the test is discontinued or omitted, please mark this on the bottom of the test form and indicate the reason.

Scoring

For Trials 1-3 and Delayed Recall: total correct responses are recorded on the form. Correct minor errors in pronunciation or pluralization (e.g., "rubies" for "ruby") as they occur; count these responses as correct. Do not give credit for words not on the list (intrusions). Duplicate words should only be counted once towards the total.

For Delayed Recognition:

1. Total number of true-positives responses (words that were on the original list [*printed in caps and not shaded*]),
2. Number of semantically-related false positive errors (*participant responded "Yes;" lower case, light shaded*),
3. Number of semantically-unrelated false-positive errors (*participant responded "Yes;" lower case, dark shaded*)
4. Total number of false positive errors: The number of false positive errors is the sum of the semantically-related (light shaded boxes) and the semantically-unrelated (dark shaded boxes) false positive errors. Add light and dark shaded boxes.

Enter all scores on the form and in the SPRINT database. (DO NOT complete the Raw Score and T Score on the bottom of page 4.)

TRAIL MAKING TESTS A & B

Description

The Trail Making Tests assess processing speed, attention, sequencing, mental flexibility and visual-motor skills.

Materials Needed

- Test Administration Form (printed from the SPRINT Website)
- Trails A & B Participant Sample Pages and Worksheets (mailed from the SPRINT-MIND CC)
- Stopwatch
- Pencil without eraser

Specific Instructions for Administering Trail Making Test

- If a participant has been randomized to the MIND Extended Battery and s/he cannot complete the MoCA Trail, it does not mean that s/he cannot do the Trail Making Test Part A or Part B. You should still administer the Trail Making Test Parts A and B regardless of his/her performance on any other test.
- Read instructions verbatim.
- If the participant is unable to complete the sample item, you should repeat the instructions up to three times. If the participant still does not understand or cannot do the sample correctly, guide the participant's hand (using the opposite end of your pen) and verbalize the instructions as you move the participant's hand through the practice items. Then administer the test. If the participant makes 5 errors or reaches 5 minutes (5:00), politely discontinue the test and record the time as 5:00 with 5 errors. **It is important to make a note** on the cover page that the participant appeared unable to understand the instructions.
- After successful completion of the sample trial, give the participant the test page.
- Repeat the test instructions, reading verbatim, as you did for the sample trial.
- Testing time = maximum of 5 minutes (300 seconds) for each test – A & B.
- Observe the participant closely in order to catch errors as soon as they are made**.** As unobtrusively and quickly as possible make a mark on the incorrect line and provide immediate feedback. (***"You were correct up to here*** [point to last correct circle]. ***Remember to go in the correct order.")***
- Record time to complete the test as **MINUTES:SECONDS** – rounding to the nearest second (e.g., 1:20 is one minute and twenty seconds).
- Record the number of errors. Remember, after 5 errors discontinue the test and record the time as 5:00 minutes.
- Discontinuation Rule for A & B: If the participant makes 5 errors or reaches 5 minutes (300 seconds), discontinue the test. Total time should be scored as 5 minutes (300 seconds) and 5 errors. If the test is discontinued because the participant reached the maximum 5 minutes or 5 errors, or the test is discontinued for any reason, make a note on the cover page of the reason why the test was discontinued.
- For specific "troubleshooting" questions, please refer to the table below.

1. **Part A**
   Administration
   Place the Part A Sample form in front of the participant.

   Examiner: ***"There are numbers in circles on this page. Please take the pencil and draw a line from one number to the next, in order. Start at 1***(point to the number), ***then go to 2*** (point to the number), ***then go to 3*** (point to the number) ***and so on. Please try not to lift the pencil as you move from one circle to the next. Work as quickly as you can. Ready?*** [pause for a response] ***Begin."***

   If the participant makes an error, mark through the line and go back to the point at which the error was made and say, for example, ***"You were at number 2. What is the next number?"*** Wait for the participant's response and say, ***"Please start here and continue."***

   If the participant completes the sample correctly, go to Test A. **Repeat the instructions given for the sample.** Start timing as soon as the instruction is given to begin.

   Stop timing when Trail A is completed or when the maximum time (5 minutes) is reached.
   Allow a maximum of 5 minutes (300 seconds) for the test. YOUR STOPWATCH SHOULD READ 5:00. Be careful not to confuse 300 on the stopwatch (3 minutes) with 300 seconds (5 minutes).

   Scoring
   Record the time as MINUTES:SECONDS (rounding to the nearest second) and number of errors on the Trails form and enter in SPRINT database.

2. **Part B**
   Administration
   Place the Part B sample form in front of the participant.

   Examiner: ***"There are numbers and letters in circles on this page. Please take the pencil and draw a line, alternating in order between the numbers and letters. Start at number 1*** (point to the number), ***then go to the first letter, A*** (point to the letter)***, then go to the next number, 2*** (point to the number) ***and then to the next letter, B*** (point to the letter), ***and so on. Please try to not lift the pencil as you move from one circle to the next. Work as quickly as you can. Ready?*** [pause for a response] ***Begin."***

   If the participant makes an error, mark through the line and go back to the point at which the error was made and say, for example, ***"You were at number 2. What is the next letter?"*** Wait for the participant's response and say, ***"Please start here and continue."*** If the participant completes the sample correctly, go to Test B. **Repeat the instructions given for the sample.** Start timing as soon as the instruction is given to begin.

Stop timing when Trail is completed or stop participant when maximum time is reached or when 5 errors have been made.

Allow a maximum of 5 minutes (300 seconds) for the test. YOUR STOPWATCH SHOULD READ 5:00. Be careful not to confuse 300 on the stopwatch (3 minutes) with 300 seconds (5 minutes).

Scoring
Record the time as MINUTES:SECONDS (round to the nearest second) and number of errors on the Trails form and enter in SPRINT database.

Troubleshooting Table

Problem	Solution
Participant lifts pencil	Instruct the participant to keep the pencil in contact with the paper and move on to the next circle
Participant asks for feedback	Instruct the participant to finish the test; encourage to continue but do not provide qualitative feedback
Participant does not understand directions	Repeat the instructions up to three times. If the participant still does not understand or cannot do the sample correctly, take the participant's hand with the opposite end of your pen and verbalize the instructions as you move the participant's hand through the practice items. Then administer the test. If the participant makes 5 errors or reaches 5 minutes (5:00), politely discontinue the test and record the time as 5:00 with 5 errors. Make a note on the cover page that the participant appeared unable to understand the instructions.
Participant lifts the paper	Instruct the participant to keep the paper oriented vertically
Participant attempts self-correct	Instruct participant to put a dash through their line and continue from the last number. Do not count self-corrections as errors.
Participant becomes inattentive or tries to leave	Ask the participant to finish the test first
Participant starts with the wrong circle	Tell the participant that they started with the wrong circle and point to circle 1
Participant skips a circle or connects the wrong circle	Return the participant to the last correct circle and say "You were at number/letter XX. What is the next number/letter?" Wait for the participant's response and say, "Please start here and continue."

Adapted from "**Administration and interpretation of the Trail Making Test"** Christopher R Bowie & Philip D Harvey, ***Nature Protocols* 1; 2277 - 2281** (2006).

THE BOSTON NAMING TEST – 15 ITEM
Materials Needed
- Boston Naming Test Stimulus book with specific pictures tabbed
- Test form downloaded from SPRINT website

Administration
Say to the participant: ***"Now I am going to show you some pictures and I want you to say the name of each picture."***

Present the 15 line drawings in the Boston Naming Test Stimulus book to the participant in order, beginning with the picture tabbed #1. For each picture, ask the following until the participant begins to name the pictures on his/her own:

"What is the name of this object?" or ***"Can you name this?"***

Allow a maximum of 20 seconds for each picture. Responses are scored as follows:

1. If the participant spontaneously gives the correct response, it is given 1 point.
2. If the response is clearly wrong, even if it is in the correct stimulus category (e.g., "hippopotamus" for "rhinoceros"), the participant would receive a score of 0.
3. If the participant cannot name the object spontaneously (says "I don't know"), s/he can be given the stimulus cue printed on the BNT form (but only once per item). If the participant correctly names the picture in response to the stimulus cue, s/he receives 1 point. However, if s/he still cannot name the picture ("I don't know" a second time), or gives a response that is incorrect, no point is given and the examiner moves to the next item.
4. A stimulus cue can also be given when the participant's first response is a *misperception* of the picture (e.g., the participant says "snake" for the "pretzel" for picture #1). In these cases, the examiner should give the stimulus cue which is printed in parentheses under the response line for each item. For example, if the response to picture #1 was "snake" the examiner should say, "It is actually something that you eat." If the participant correctly names the picture in response to the stimulus cue, 1 point is given. If the participant gives an incorrect response, even if it is another misperception, s/he receives a score of 0.
5. Allow up to 20 seconds after giving the cue, then proceed to the next picture.

The cues are as follows:

**Picture**	**Stimulus Cue**
Pretzel	something to eat
Seahorse (or Horsefish)	an ocean animal
Rhinoceros	an animal
Acorn	it comes from a tree
Dominoes	a game
Pelican	a bird
Stethoscope	used by doctors and nurses
Muzzle	used on dogs
Unicorn	mythical animal
Asparagus	something you eat
Scroll	a document
Tongs	a utensil
Sphinx	it's found in Egypt
Trellis	used in a garden
Palette	artists use it

Do not give a stimulus cue for an incorrect response. For example, "dice" is not a correct response for "dominoes" and it does not warrant a cue. Score "dice" as incorrect.

Other examples of incorrect responses where stimulus cues should not be given are:

Rhinoceros: Hippopotamus
Pelican: Seagull
Muzzle: Harness, Bridle
Scroll: Declaration of Independence
Tongs: Thongs, Tweezers
Sphinx: Pharaoh, Pyramid
Trellis: Lattice, Fence

Use a prompt for responses that indicate only a part of the picture. For example, for picture #8 if the participant says, "dog" you should point to the picture with your pencil or pen, outline the item and say "It's used on dogs." Score as correct only if the participant names the item ('muzzle').

A non-specific prompt can be used if the response is correct but too general. For example, if the response to the "dominoes" item is "game", say ***"Is there another name for that?"*** You may NOT ask "Isn't that a special kind of game?" If no response is given, score as incorrect. Other examples of when to use a non-specific prompt are:

Acorn: Nut
Pelican: Bird
Sphinx: Statue

If the participant makes an error but corrects it immediately, score the response as correct. If the participant makes an error but corrects it after you have moved on to the next picture, no point is given.

Scoring

Give 1 point for each correct response given spontaneously. Give 1 point for a correct response following the stimulus cue. Give 0 points for incorrect responses whether or not a cue is given. The participant's total BNT score is the sum of responses to the 15 items.

Regional terms and synonyms, if verified, are counted as correct. Acceptable alternate responses are provided in parentheses next to the item on the score sheet. If the participant gives a response in a different language (other than the language the test is administered in), it should be counted as incorrect.

MODIFIED REY-OSTERREITH COMPLEX FIGURE

Description

The Rey-O figure assesses non-verbal learning and recall by having the participant first copy then reproduce from memory a figure. It uses visuospatial abilities, memory, attention, planning, and working memory.

Materials Needed

- Black Pen
- Laminated Rey-O figure
- Participant worksheets (printed from SPRINT website)

Specific Instructions for Administering the Rey-O

This test has two parts:

Part A – Copy**:** Participant copies the figure

Part B – Immediate Recall: Participant draws the figure from memory

- Use a black pen.
- Laminated card with figure should be placed directly in front of the participant in correct orientation (landscape orientation, 'kite' on right side and 'cross' on left side) with the blank piece of paper directly beneath it in the same orientation.
- Participant may not move or turn the drawing but they may move or turn the response sheet.
- Do not allow participant to draw on the laminated figure.

Administration

1. **Part A – Copy**
   - Give participant black pen.
   - Place the sheet entitled "Rey-O Copy" oriented landscape in front of the participant with the words "Rey-O Copy" printed at the top of the form.

   Examiner: ***"I would like you to make a copy of this drawing. Make sure that you look at the original carefully as you go along and try not to leave out any parts."***

   There is no time set limit for this test, however to keep the session efficient stop after 3 minutes. If the drawing is careless, the participant should be encouraged to make the copy as accurate as possible.

2. **Part B – Immediate Recall**
   - Remove the drawing and the laminated figure from view
   - Place the sheet entitled "Rey-O Immediate Recall" oriented landscape in front of the participant with the words "Rey-O Immediate Recall" printed at the top of the form.

   Examiner: ***"That was good. Next, I'd like to see how much of that drawing you can reproduce from memory. Even if you think you won't be able to remember very much, go ahead and try as best you can, even if it is just one single line that you remember…….Ready? Begin."***

   Again, there is no time limit for this test; encourage the participant to do his/her best. After 3 minutes thank the participant and remove the sheet.

Scoring

The Rey-O drawings will be scored centrally at the SPRINT-MIND CC and entered into the SPRINT database. Participant drawings should be faxed to the SPRINT-MIND CC (ATTN: Nancy Woolard, 336-713-8800) or copied and mailed to the CC:

Nancy Woolard, Clinical Research Manager
SPRINT MIND CC
Wake Forest Health Sciences
Medical Center Boulevard
Winston-Salem, NC 27157-1207

<u>CATEGORY FLUENCY-ANIMALS</u>

Description

This task requires the participant to name as many animals as possible in 60 seconds. (STOPWATCH WILL READ 1:00). It assesses verbal fluency.

Materials Needed

- Test form downloaded from website
- Stopwatch

Administration

Examiner: ***"I am going to give you a category and I want you to name, as fast as you can, all of the things that belong in that category. For example, if I say "articles of clothing," you could say 'shirt,' 'tie,' or 'hat'. Can you think of other articles of clothing?"***

Allow the participant to produce 2 or 3 responses. If the responses are not articles of clothing, you can say**, *"No, X is (are) not an article(s) of clothing. You could have said 'shoes' or 'coat' since they are articles of clothing."***

Then, read the following instructions: ***"Now, I want you to name things that belong to another category: Animals. You will have one minute. I want you to tell me all the animals you can think of in one minute. Ready? Begin."*** Start timer**.**

Record the participant's responses, using abbreviations if necessary. Review the audio recording afterwards if the participant names animals faster than you are able write.

One prompt is permitted ***("Tell me all the animals you can think of.")*** if the participant makes no response for 15 seconds or expresses incapacity (e.g., "I can't think of any more"). Stop the participant at the end of one minute (STOPWATCH SHOULD READ 1:00).

Scoring

Give one point for each animal named within one minute (1:00). Any animal, that is anything that is not mineral or vegetable, is acceptable (e.g., insects, viruses, bacteria, paramecium, anemone, humans, etc). Imaginary, extinct or magic animals are permissible as are names of different aged animals (puppy-dog). Proper names, repetitions, characteristics of animals (e.g. carnivore, herbivore), or variations (e.g.

‘baby dog-puppy’) are not allowed. Different breeds within the same family are acceptable, e.g., poodle, beagle. However, proper names (e.g., Butch) or different names for the same animal (e.g., little dog and puppy) are considered the repetitions and would receive only 1 point.

Enter total score on the form and into the SPRINT database.

<u>DIGIT SPAN TEST</u>

Description

For Digit Span Forward, the participant is read a sequence of numbers and asked to repeat them in the same order. For Digit Span Backward, the participant is read a sequence of numbers and asked to repeat them in reverse order.

Materials Needed

- Test forms downloaded from website

Special Instructions for Administering the Digit Span Test

- Digit Span includes two tasks: Digit Span Forward and Digit Span Backward. Administer both tasks to the participant.
- Each test is composed of two trials for a given span length. Administer both trials of each item.
- Read each trial verbatim at the rate of one digit per second, dropping your voice slightly on the last digit in the sequence. Pause to allow the participant to respond.
- If the participant begins to respond before you have finished reading the trial, present the remainder of the trial and allow the participant to respond. Award appropriate credit for the response and then say, ***“Remember to wait until I’m finished before you start.”***
- Do <u>not</u> repeat any trial. If the participant asks you to repeat a trial, say, ***“I cannot repeat the sequence. Just take your best guess.”***
- If a participant provides multiple responses to a trial or self-corrects after their initial response, score only the intended response. If it is not clear which one is the intended response, say, ***“You said*** [*insert participant’s response]* ***and you said*** [*insert participant’s response*]. ***Which one did you mean?”*** Score the intended response.
- Provide assistance on the sample items of Backward only. Proceed with Item 1 even if the participant is unable to respond correctly to any trial of the sample items.

Discontinuation Rule

- **Forward:** Discontinue after scores of 0 on *both trials* of an item.
- **Backward:** Discontinue after scores of 0 on *both trials* of an item.

Administration

1. **Forward**

   Items 1-8

Examiner: "***Now I'm going to say some numbers. Listen carefully; I can only say them one time. When I am through, I want you to say them back to me in the same order. Just say what I say.***" Proceed to Trial 1 of Item 1.

Administer Trial 1 and Trial 2 of each item. Discontinue when the participant misses both trials of the same span length. Proceed to the next item if the discontinue criterion has not been met. So, if the participant misses one trial and is correct on the other, you are to continue to the next item (span length).

Remember to administer Backward regardless of the participant's performance on Forward.

2. **Backward**

Sample Item

Sample Trial 1: Say: ***"Now I am going to say some more numbers, but this time when I stop, I want you to say the numbers backward. If I say 7 – 1, what would you say?"***

<u>If the response is correct</u> [1 – 7] then say, ***"That's right."*** Proceed to Trial 2.

<u>If the response is incorrect then</u> say, ***"That's not quite right. I said 7 – 1, so to say them backward, you should say 1 – 7."*** Proceed to Trial 2.

Sample Trial 2: Say, "***Let's try another one. Remember to say them backwards. 3 – 4.***"

<u>If response is correct</u> [4 – 3] then say, ***"That's right. Let's try some more."*** Proceed to Trial 1 of Item 1.

<u>If response is incorrect</u> then say, ***"That's not quite right. I said 3 – 4, so to say them backward, you should say 4 – 3. Let's try some more."*** Proceed to Trial 1 of Item 1.

Administer Trial 1 and Trial 2 of each item. <u>You do not need to repeat the sample items because they are included in the script.</u> Discontinue when the participant misses both trials of the same span length. Proceed to the next item if the discontinue criterion has not been met. So, if the participant misses one trial and is correct on the other, you are to continue to the next item (span length).

Scoring

- Correct responses are listed on the Record Form.
- Record the participant responses verbatim on the Record Form so you can verify afterwards.
- For each trial, score 1 point if the participant gives a correct response.
- For each trial, score 0 points if the participant gives an incorrect response, says that they do not know the answer, or does not respond within approximately 30 seconds.

- The item score is the sum of the correct responses for each part.
  Maximum Digit Span Forward Total Score: 16 points
- The Digit Span Backward total score is obtained by summing the Backward item scores. Do not include the sample item in the score.
  Maximum Digit Span Backward Total Score: 16 points

Participant Notification of Results

The SPRINT MIND CC does not send test results to participants because the results are designed for research and are not diagnostic for treatment purposes. Since participants often wonder how they did on the MIND tests, a letter has been developed that lets participants know that no news is good news and if they have concerns about their memory they should discuss them with their doctor. This letter can be found on the SPRINT website (Documents > MIND > Letter to give to participants following MIND testing) and may be given to participants after their MIND testing.

THE FUNCTIONAL ASSESSMENT QUESTIONNAIRE (FAQ)

Description

The FAQ is a brief, 10-item questionnaire that has been validated for the purposes of ascertaining the impact of cognition on important daily functions. The FAQ is essential to SPRINT MIND adjudicators' ability to determine the study's cognitive outcome. Results from the FAQ in combination with the cognitive tests will be used to classify the cognitive status of participants at Years 2 and 4.

The MIND Coordinating Center will administer the FAQ centrally for all SPRINT sites that have obtained IRB approval. Sites that opt not to participate in central FAQ administration must maintain a 90% completion rate (90% of required FAQs completed within the 30 day window).

The following reports and tools are provided on the SPRINT website to assist CCN Coordinators and clinic site personnel with the timely acquisition of the FAQ:

Reports > MIND > FAQ > Listing of CENTRAL FAQs Needed

> This report allows the clinics to know which participants need an FAQ administered centrally. It includes the progress made towards the FAQ completion.

Reports > Clinical Operations > Listing of LOCAL FAQs Needed

> This report is for those sites administering FAQs locally.

Reports > Clinical Operations > Missing or Incorrect Proxy Information - CCN Level or Site Level

> Clinic personnel should check this report on a weekly basis and update the contact information form for those participants listed.

Documents > MIND > Letter for Participant to Provide their Contacts

> Staff should confirm that this letter is provided to all participants and encourage participants to give it to their contacts.

The Participant Note section is the means to communicate with the CC. Clinic staff should note any information that will be helpful for the CC callers to know before making the FAQ call. CC callers will also add information in the Participant Note regarding success or challenges in obtaining the FAQ.

<u>Talking points with participants when questions arise about the FAQ or MIND testing:</u>
At the 24 and 48 Month visits, tell each participant (at the conclusion of the visit – not immediately following the MIND testing):

Many of our SPRINT participants are selected to have an additional questionnaire completed by a family member or friend. Calls will be made to the person listed as their contact (confirm that this person knows they are in SPRINT, confirm name and phone number) and ask participants to let their contact know to expect a call. Ask participants if the person listed is familiar enough with the participant's daily activities to respond to a few short questions.

Just as SPRINT is looking at how high blood pressure affects the heart, kidneys, and other organs, it is also looking at how high blood pressure might affect memory and thinking. By answering these few short questions, SPRINT participants and their contacts are making a difference for future generations.

At any time during SPRINT, if a problem is detected the participant will be notified through the physician at their SPRINT clinic.

Materials Needed for LOCAL FAQ Administration

- FAQ form printed from the SPRINT Website
- Name, phone number, and relationship of person to contact

Specific Instructions for Administering the FAQ
The FAQ will be obtained on any participant scoring below the specified cutpoint. Additionally, an FAQ will be obtained on all participants in the 2800 at 24 and 48 months.

Once the Screening Battery is entered in the database, a computer algorithm will evaluate if the FAQ needs to be administered. A site specific report on the SPRINT website will indicate participants who tripped and when the FAQ will need to be administered. This report will need to be checked regularly at the clinic and can be found on the SPRINT website under REPORTS→Clinical Operations→Listing of LOCAL FAQs Needed. The FAQ must be administered **within one month** of the baseline visit and at follow-up within one month of the Screening Battery.

It is not necessary for you to ask the participant again who you should contact. The participant has indicated who should be contacted on the Participant Contact Information Form in box 12.The FAQ is to be administered to a person listed as a contact of the participant – someone who is in close contact with the participant and has knowledge about the participant's daily functioning. The contact should be instructed to

respond to each question based on the participant's ability during <u>the past four (4) weeks</u>.

It is imperative that accurate contact information is provided on the Participant Contact Form. Review this form with the participant at each visit to confirm the information.

At least four attempts to contact the pre-designated person should be made. These four attempts should be made over a period of two weeks and should be attempted at different times and days. If, after four attempts, you are unable to reach that contact, you should choose another contact listed on the form and follow the same process. If you are still unable to reach the participant's contact, you should contact your CCN MIND PI.

Administration
The FAQ is typically administered over the phone.

Say, ***"Hello. I'm ____________from _______University/Clinic. I'm calling today on behalf of _______(Ppt name) who has volunteered for the SPRINT Study. He/She gave us permission to contact you if, during the study, we needed to collect some additional information about how their health might be affecting their ability to do certain things. This won't take more than 5 minutes and we really appreciate your willingness to do this.***

I would like to ask you some questions about how Mr./Mrs. __________handles some common daily functions. Using the following responses, please choose the most accurate representation of his/her level of ability to perform each activity in the past four weeks:

Normal
Has difficulty, but does by self
Requires assistance
Dependent
Not Applicable (e.g., never did)

In the past four weeks, did Mr./Ms. have any difficulty or need help with:"

The FAQ is a semi-structured interview where the examiner has some flexibility to probe for additional information or clarification in order to rate the person's responses correctly, unlike other cognitive tests where the script must be strictly followed.

1. The questions should be asked as noted on the form: "In the past 4 weeks, has Mr. X had any difficulty with or needed help in writing checks, paying bills, or balancing a checkbook?" ***However*,** the examiner may ask follow-up questions and provide clarification to the contact when necessary in order to assign a score based on the contact's response or narrative. This process allows for some judgment on the examiner's part.
2. If necessary, the examiner should continue to probe to make sure that his/her knowledge of the participant's status is accurate. For example, if the contact reports "dependent" and the examiner believes s/he misunderstands the term,

the examiner should seek clarification such as, “So you’re saying, Mr. X cannot perform this activity on his own.”

3. If after probing, the response N/A is selected, provide a rationale for that response in the comment box on the form in order to aid the adjudicators. This should be used very rarely.
4. Any information that might be helpful to adjudicators in their deliberation should be included in the comments box on the form. This could include information such as the participant was in the room, therefore the contact was not able to speak freely, the participant recently had a medical event which limits activities, etc. When in doubt err on the side of placing a note on in the comments box.

Record the responses on the FAQ form and enter into the SPRINT database.

<u>FOLLOW-UP SPRINT MIND TESTING AT 24 AND 48 MONTH VISITS</u>

Change in memory is important in SPRINT MIND. To identify people who have clinically significant changes in cognitive functioning we administer the MIND tests every 2 years. If a SPRINT participant has received a diagnosis from a local physician of dementia and/or started a memory enhancing medication, the SPRINT MIND tests should still be administered at the appropriate visits.

All SPRINT participants will complete the MIND Screening Battery at the 24 and 48 month visits. MIND forms will be printed in the visit packet from the SPRINT website*.

All participants who were randomized to the 2800 MIND Extended Battery at baseline will also complete the MIND Extended Battery at the 24 and 48 month visits <u>and</u> have the FAQ administered to a contact. These MIND forms will also be printed in the visit packet from the SPRINT website*.

For Non-2800 participants who score below the established cutpoint on the Screening Battery, the FAQ must be obtained within 30 days. If the FAQ score is >0 or the MoCA Delayed Recall is 0 or 1, the participant will be asked to complete the MIND Extended Battery at the 27 month or 51 month visit. The MIND forms will be printed in that (27 or 51 month) visit packet from the SPRINT website*. If an FAQ is not obtained within 60 days of the Screening Battery, the participant will be asked to complete the MIND Extended Battery at the 27 month or 51 month visit.

A report is available which provides clinics a list of Non-2800 participants who have triggered the MIND Screening Battery and FAQ and need to complete the Extended Battery at the 27 month or 51 month visit. The report can be found under Reports→Clinical Operations→Non-2800 Needing an Extended Battery. It is also included in the Top Reports folder.

If a participant misses the 24 or 48 month visits, MIND testing should be completed at the next clinic visit (27 or 51 month visits). If the participant misses 2 consecutive visits (24 and 27 month; 48 and 51 month), the SPRINT MIND Telephone Battery will be administered centrally by the MIND CC. If the participant should return to the clinic after the Phone Battery has been obtained, the full MIND test battery (Screening and/or Extended as appropriate for participant) should be completed.

If there is not sufficient cognitive data collected at the visit (i.e., several missing tests), as deemed by the MIND CC, you may be instructed to re-administer the entire battery at the next visit. Forms will automatically print with the visit packet.

Rule of thumb: If the participant has missed his/her MIND testing or has insufficient data and returns to the clinic for a visit, always obtain the Screening Battery. If participant is in the 2800 cohort or if the participant triggered, then the Extended Battery should be administered. The forms will print as part of the visit packet.

If there has been no contact at all with the participant between the 24 and 36 month visits, the Dementia Questionnaire will be administered to a contact by the MIND CC.

A portion of the MIND test forms are mailed to the site from the MIND CC.

CERTIFICATION PROCESS FOR FIELD SITE COGNITIVE ASSESSORS

In order for the cognitive data to be reliable, certification of all cognitive examiners is essential prior to any data collection. A 3-step process for training will be implemented:

Prior to the central training, all examiners will be required to review the MOP and the test forms carefully. Additionally each examiner will be asked to watch a video of a complete administration of both batteries by an experienced examiner. This material will be posted to the SPRINT website. In addition, each examiner will be asked to administer a practice administration of the complete test battery to a volunteer (colleague, friend or family member). Because this is prior to the training, the goal of these practices is to become familiar with the batteries. It is recommended that several practice sessions take place prior to training.

At centralized training, each examiner will attend a 2-hour session on each cognitive battery. During each session a brief overview of the tests in the battery will be given, a video demonstration will be viewed, live practice will occur and questions will be answered by the trainers.

After centralized training, examiners will be required to practice the test batteries, and when confident in their skills, record a test session with a volunteer. The recorded interview will be saved to a CD and the CD and corresponding answer sheets will be mailed to the SPRINT-MIND CC for review. When an acceptable level of proficiency with both batteries is acheived, certification will be awarded and the examiner will be approved to conduct the test batteries with SPRINT participants.

If at training, an examiner demonstrates competency with all or parts of the batteries, certification will be awarded at that time.

During the course of the SPRINT study, if the field sites determine that additional cognitive examiners are needed due to staff turnover or clinic volume, a certified examiner at the site will help to train the new staff member. The new staff member will be required to review the MOP along with the videos posted to the website, and then

practice the test batteries on volunteers who are not familiar with the test battery. The previously certified examiner should discuss questions and problems with the new examiner. Once confident in their skills, they should record a test session on a different volunteer who is not familiar with the test battery. The recorded session and corresponding scored answer sheets will be mailed to the SPRINT-MIND CC for review. Certification materials submitted using a co-worker or someone who is familiar with the MIND batteries will not be accepted or reviewed. Our experience with this and other trials has shown that administering cognitive tests less than once a month significantly increases the number of administration and scoring errors. If staff is asked to resubmit certain parts of the battery before certification can be awarded, these materials must be received by the MIND CC within 30 days of the most recent feedback. Any materials received after the 30 day window will not be reviewed and staff will be asked to resubmit the entire MIND battery.

For the purpose of ongoing quality assurance, the SPRINT-MIND CC will periodically request forms and recordings from certified examiners to review for ongoing QA purposes. The Coordinating Center will select these administrations randomly and notify the site by email. Once reviewed, the results are posted to the SPRINT website and an email will be sent to the examiners and clinic PI directing them to the location on the website to read the review. Each review will include feedback on the examiner's administration, scoring, and adherence to protocol.

After initial certification, the SPRINT-MIND CC will re-certify all cognitive examiners on an annual basis (+/- 1 month). The Coordinating Center will request an administration from a SPRINT study visit be submitted for review for recertification. If a QA review is done within the recertification timeframe, that recording may be used for recertification. The Coordinating Center will select these administrations randomly. Once reviewed, the examiners will be grouped into three categories:

- Acceptable: Recertification awarded
- Needs Improvement: Provisional Recertification awarded, however a second administration will be requested (randomly selected by the CC) within the next 3 months to confirm that the examiner read, understood, and has adjusted his/her administration or scoring. If adjustments are still necessary you will be notified that you are now in the Unsatisfactory category.
- Unsatisfactory: Examiner needs immediate consultation with the CCN MIND PI for additional training.

If your administration is deemed Unsatisfactory, we will expect you to contact your CCN MIND PI and set up a time to review the feedback the CC has provided and the MIND MOP chapter. The CCN MIND PI will work with you to re-train you for MIND test administration. When he/she is confident that you are now performing up to standard, he/she will notify Nancy Woolard and we will set up a time for you to administer the MIND tests to a staff member in the MIND CC over the phone. You will score the tests and fax them to the CC for review. Immediate feedback will be provided and if acceptable, recertification awarded. If there continue to be unresolved issues, further plans for retraining will be discussed and you will be advised to refrain from conducting the MIND testing until further notice.

Quality control reviews from this and other trials have clearly shown that administering the cognitive tests less than once a month significantly increases the number of administration and scoring errors. All SPRINT MIND certified examiners are encouraged to administer the MIND battery to participants at least once per month in order to keep their skills fresh. The MIND CC will review each examiner's administration status monthly and an email will be sent to staff that have not administered the battery in 30, 60, or 90+ days. If an examiner has not administered the test battery in 90 or more days, they will be required to become re-certified by conducting an administration (screening and extended battery) on a volunteer that is not familiar with the test battery and sending the recording and forms to the MIND CC for review and recertification.

If there is a period between the end of randomization, 24, and 48 month visits when the MIND battery is not administered to any participants in 90 or more days because of the visit schedule at the site, there will be a lapse in certification for SPRINT MIND. Each examiner at the site will be required to become recertified prior to the start of the next round of MIND visits, using the process listed above.

PROCESS FOR DEMENTIA ADJUDICATION AT FOLLOW-UP VISITS

Overview

A primary goal of SPRINT MIND is to ascertain incident cases of all-cause dementia in SPRINT and determine whether the incidence is related to the treatment assignment. Since the SPRINT intervention could impact the incidence of sub-clinical or pre-dementia syndromes as well, Mild Cognitive Impairment (MCI) syndrome will also be ascertained. Participants not classified as having dementia or MCI will be considered to have no cognitive impairment (NCI). Ascertainment will be based on information collected in SPRINT and SPRINT MIND including the Screening and Extended cognitive batteries, the Functional Assessment Questionnaire completed by a knowledgeable proxy and the demographic information, medical history, medication inventory (other than study drugs), depressive symptom severity, the results from a baseline physical examination and the report of any serious adverse events including the date, type of event and diagnoses. In rare instances when a participant is not able to be tested face-to-face, alternate measures of cognitive and functional status will be used (Telephone Inventory for Cognitive Status, Dementia Questionnaire). To evaluate the severity of cognitive deficits and changes over time, adjudicators will consult appropriate norms for each test in the Screening and Extended batteries (provided by SPRINT MIND). Absolute scores and intra-individual change in test performance over time will also be considered in adjudication.

Process

Classification of Dementia, MCI or No Impairment in SPRINT MIND is made by an Adjudication Panel consisting of experts in the diagnosis of dementia and MCI who are blinded to study assignment and blood pressure data. The steps in the adjudication process are as follows:

1. The initial pool of potential cases will include all participants where the MoCA test scores triggered the administration of the Extended Cognitive Battery and FAQ.
2. An algorithm will be applied to the initial pool to remove participants who are highly likely to be classified as 'No Impairment'.

3. The remaining potential cases will be adjudicated as follows:
   a) Two adjudicators will be selected by the adjudication coordinator to review each case with the stipulation that an adjudicator cannot review cases from his/her clinical site.
   b) Both adjudicators will independently log-in to the SPRINT website and the SPRINT MIND Adjudication sub-site, review all the available data and enter their final classification (Dementia, MCI, No Impairment, Cannot Classify). For MCI classifications, each adjudicator will then make an additional sub-classification of MCI type (amnestic-single domain, amnestic-multi-domain, non-amnestic single domain, non-amnestic-multi-domain).
   c) In instances where both adjudicators independently agree, their classification will be recorded as the final adjudication classification.
   d) In instances where adjudicators disagree:
      i) If the two adjudicators disagree between sub-classifications (i.e., MCI-AMD vs MCI-ASD), the two adjudicators will resolve the case via email. If they still cannot come to consensus, then the case will be assigned to the entire Adjudication Panel for review.
      ii) If the two adjudicators disagree on the primary classification (i.e., PD vs MCI), the case will be assigned to the entire Adjudication Panel to be reviewed and discussed on a regularly scheduled Consensus Conference call.
   e) On the Consensus Conference call, the case will be presented to the entire Adjudication Panel by the 2 primary reviewers and then discussed by the entire committee until consensus is reached. Once consensus is reached it will be recorded as the final adjudication classification.

Adjudicators will not be provided with incidence rates or other information that might bias their evaluations.

These procedures have been used by members of the cognitive adjudication team in other large, multi-site clinical trials including the Gingko Evaluation of Memory Study (GEMS), the Women's Health Initiative Memory Study (WHIMS), and the Lifestyle Interventions and Independence for Elders (LIFE).

Post Adjudication Assessment
All participants adjudicated as having MCI or No Impairment will continue to receive regularly scheduled cognitive assessments with the Screening and Extended Cognitive Batteries and will undergo subsequent adjudication when indicated.

Participants classified as having dementia will no longer be assessed for cognitive function but will be encouraged to continue their participation in SPRINT. Sites will be notified via email by the MIND Coordinating Center of participants who have been classified as having dementia. Sites will be responsible for notifying these participants and their primary care providers (if permission is allowed) of this classification. Sites should be careful not to use the word “dementia” when discussing this with participants since these tests are used for research purposes and are not diagnostic. Only their physician can determine a diagnosis. . A model letter has been developed for sites to use that includes language that will be helpful when discussing this outcome with the participant. The letter uses generic language that lets the participant know that some of

the scores on their recent memory tests were lower than expected for someone their age. The letter will be attached to the email from the MIND CC. The letter should not be mailed to the participant until the Principal Investigator (or their designee) discusses this information with the participant.

MRI SCREENING AND SCANNING PROTOCOL (See Chapter 9)

5. Intervention: Medications and Treatment Algorithms

5.1 Overview

The Systolic Blood Pressure Intervention Trial (SPRINT) is a 2-arm, multicenter, randomized clinical trial designed to test whether a treatment program aimed at reducing systolic blood pressure (SBP) to a lower goal than currently recommended will reduce cardiovascular disease (CVD) risk. About 9250 participants with SBP ≥130 mm Hg and at least one additional CVD risk factor will be recruited at approximately 90 clinics within 5 clinical center networks (CCNs) over a 2-year period, and will be followed for 4-6 years. Approximately 4300 participants will have chronic kidney disease (CKD), and 3250 will be aged 75 or older. The primary outcome is the first occurrence of a myocardial infarction (MI), acute coronary syndrome (ACS), stroke, heart failure (HF), or CVD death. Secondary outcomes include all-cause mortality, decline in renal function or development of end stage renal disease (ESRD), dementia, decline in cognitive function, and small vessel cerebral ischemic disease.

5.2 Research Design and Blood Pressure Goals

This is an unmasked, open label, randomized clinical trial. Participants eligible for the trial will be randomized to one of two goals: SBP <120 mm Hg for the more intensive goal (Intensive Group) and SBP <140 mm Hg for the less intensive goal (Standard Group). Figures 7.1 and 7.2 describe the treatment algorithms for the two treatment groups. Although there are no diastolic blood pressure (DBP) inclusion criteria, participants in both groups with DBP ≥90 mm Hg will be treated to a DBP goal of <90 mm Hg if needed after meeting the SBP goal, because of the many trials documenting the CVD benefits in treating to a DBP goal <90 mm Hg

5.3 Antihypertensive Classes (Agents)

Use of once-daily preparations of antihypertensive agents is encouraged unless alternative dosing frequency (e.g., BID) is indicated/necessary. One or more medications from the following classes of agents will be provided by the study and intended for use in managing participants in both randomization groups to achieve study goals:

- Angiotension converting enzyme (ACE)-inhibitors
- Angiotension receptor blockers (ARBs)
- Direct vasodilators
- Thiazide-type diuretics
- Loop diuretics
- Potassium-sparing diuretics
- Beta-blockers
- Sustained-release calcium channel blockers (CCBs)
- Alpha1-receptor blockers
- Sympatholytics

Combination products will be available, depending on cost, utility, or donations from pharmaceutical companies. The study formulary is shown in Table5.1, along with information about usual dose and frequency ranges.

Table 5.1 SPRINT Formulary

Class	Drug	Available Strengths	Usual Dose Range / day	Usual Daily Frequency
Diuretic	Chlorthalidone	25mg	12.5-25 mg	1
	Furosemide	20mg, 40mg, 80mg	20-80 mg	2
	Spironolactone	25mg, 50mg	25-50 mg	1
	Triamterene/HCTZ	75/50mg	37.5/25 mg – 75/50 mg	1
	Amiloride	5 mg	5-10 mg	1 - 2
Ace Inhibitor	Lisinopril	5mg, 10mg 20mg, 40mg	5-40 mg	1
Angiotensin Receptor Blocker	Losartan	25mg, 50mg, 100mg	25 – 100 mg	1 - 2
	Azilsartan	40mg, 80mg	40-80 mg	1
	Azilsartan/chlorthalidone	40/12.5mg, 40/25mg	40/12.5 – 40/25 mg	1
Calcium Channel Blockers	Diltiazem	120mg, 180mg, 240mg, 300mg	120-540 mg	1
	Amlodipine	2.5mg, 5mg, 10mg	2.5-10 mg	1
Beta Blockers	Metoprolol Tartate	25mg, 50mg, 100mg	50-200 mg	2
	Atenolol	25mg, 50mg, 100mg	25-100 mg	1
	Atenolol/Chlorthalidone	50/25mg, 100/25mg	50/25 mg – 100/25mg	1
Vasodilators	Hydralazine	25mg, 50mg, 100mg	50-200 mg	2
	Minoxidil	2.5mg, 10mg	2.5-40 mg	1-2
Alpha 2 Agonist	Guanfacine	1mg, 2mg	0.5-3 mg	1
Alpha Blockers	Doxazosin	1mg, 2mg, 4mg, 8mg	1-16 mg	1
Potassium Supplements	KCL tablets	20mEq	20-80 mEq	1-2
	KCL oral solution (10%)	20mEq/15ml	20-80 mEq	1-2

Additional medications are included in a restricted use formulary (Table 5.2). These medications will be provided by the study and but may be prescribed for individual participants only after consultation with and approval from a designated representative for each CCN.

Table 5.2 SPRINT Restricted Use Formulary

Class	Drug	Available Strengths	Usual Dose Range in mg/day	Usual Daily Frequency
Ace Inhibitor	Lisinopril/HCTZ	20/12.5 mg, 20/25mg	10-40 / 12.5-50	1
Adrenergic inhibitor	*Reserpine	0.1mg, 0.25mg	0.1-0.25	1
Alpha 2 Agonist	Clonidine patch	0.1mg, 0.2mg, 0.3mg	0.1-0.3	1 wkly
Beta blocker	Metoprolol ER	25mg, 50mg, 100 mg, 200 mg	50-200	1
Diuretic	Amiloride/HCTZ	5/50mg	5/50	1
Thiazide diuretic	HCTZ	12.5, 25 mg	12.5 – 50	1
Angiotensin Receptor Blocker	Valsartan	80mg, 160mg, 320mg	80-320	1-2

* temporarily unavailable

5.4 Selection of Antihypertensive Medications

The SPRINT trial is testing a treatment strategy question regarding different SBP goals and not testing specific medications. The SPRINT BP treatment protocol is flexible in terms of the choice and doses of antihypertensive medications, but there should be preferences among the drug classes, based on CVD outcome trials results and current guidelines. NHLBI is updating various guidelines. The update of hypertension recommendations, JNC-8, should be available early in the recruitment phase of SPRINT. These updates, along with any new scientific developments, will be considered during and following SPRINT protocol development and throughout the trial.

The investigator may select among the available SPRINT antihypertensive medications for initiation of therapy. Other drugs not supplied by the trial may also be used as the investigator determines appropriate. However, all antihypertensive regimens should include one or more drug classes with strong CVD outcome data from large randomized controlled hypertension trials, i.e., a thiazide-type diuretic, calcium channel blocker, ACE inhibitor or ARB. Current evidence, the most recent JNC guidelines and over 40 years of clinical trial experience in hypertension support the inclusion of a thiazide-type diuretic as one of the agents for patients without compelling reasons for another medication, or contraindication or intolerance to a thiazide-type diuretic. (ALLHAT, 2002; Beckett and others, 2008; Chobanian and others, 2003; Psaty and others, 1997; SHEP, 1991) Other classes associated with substantial reductions in CVD outcomes in hypertension trials, e.g. ACE inhibitors, ARBs, and calcium channel blockers, combine effectively with thiazides for lowering BP (Julius and others, 2004). ACE inhibitors and ARBs also combine well with CCBs; if three drugs are needed, a thiazide-type diuretic, a RAS blocker (ACE inhibitor or ARB, but not both), and CCB make a very effective and usually well-tolerated regimen (Calhoun and others, 2009). The preference for the order in which these agents are selected is left to the investigator as long as the SBP goals are achieved. A loop diuretic may be needed in addition to or in place of a thiazide-type diuretic for participants with advanced CKD. Combinations of RAS blockers (ACE inhibitor, ARB, and/or renin inhibitor) are strongly discouraged, based on lack of benefit and adverse outcomes seen in several large randomized controlled trials.

Beta-adrenergic blockers, which were recommended in JNC-7 among the 4 preferred classes after diuretics, are now considered to be less effective in preventing CVD events as primary treatment of hypertension compared with thiazide-type diuretics, CCBs, and RAS blockers (Lindholm, Carlberg, and Samuelsson, 2005). However, there are patients for whom beta-blockers should be part of the initial therapy, namely those with coronary artery disease, including chronic stable angina or previous MI (Rosendorff and others, 2007).

Finally, although renoprotective benefits have been demonstrated in CKD patients with proteinuria, ACE inhibitors (and likely other RAS blockers) are less effective than other classes in lowering BP and in preventing CVD events in African American and elderly hypertensive patients unless combined with a diuretic or CCB (Julius and others, 2004; Mancia and others, 2007; National Collaborating Centre for Chronic Conditions, 2006; Wright and others, 2005; Wright and others, 2008).

Since more than three drugs will be necessary in many participants to reach the intensive SBP goal, other classes will also be available in SPRINT. These include the potassium-sparing diuretics, spironolactone and/or amiloride, which are very effective as add-on agents for BP-

lowering in “resistant hypertension” (Calhoun and others, 2008). However, they should be used with careful monitoring in participants with CKD or any tendency to hyperkalemia. Alpha-blockers have been used effectively as add-on therapy in the AASK, ACCORD and Anglo-Scandinavian Cardiac Outcomes (ASCOT) trials. However, alpha-blockers should be used only in combination with one or more other agents proven to reduce CVD events in hypertensive patients (ALLHAT, 2003). Sympatholytics, direct vasodilators, and/or loop diuretics may also be added for BP control in combination with agents proven to reduce CVD events.

Among thiazide-type diuretics, the most consistent and robust CVD outcome data have been seen with chlorthalidone (ALLHAT, 2002; SHEP, 1991). Chlorthalidone 12.5-25 mg/d has been shown to be more effective in lowering BP over 24 hours than hydrochlorothiazide 25-50 mg/d (Ernst and others, 2006). Among CCBs, amlodipine has been used in far more hypertension CVD outcome trials than any other agent and has more robust CVD outcome data. Amlodipine should be considered first when a CCB is to be used. In the presence of significant proteinuria, amlodipine should probably be used in conjunction with a RAS blocker. If a non-dihydropyridine CCB (e.g., diltiazem) is to be used, it should not be combined with a beta-blocker.

The ACCORD experience (The ACCORD Study Group, 2010) has shown that a treatment strategy that includes a variety of classes can produce a 14 mm Hg delta in SBP between the two randomized groups. The average number of antihypertensive drugs used to produce this difference was 3.4 and 2.1 in the Intensive and Standard Groups, respectively. It is anticipated that the study participants in the CKD subgroup of SPRINT will require a greater number of antihypertensive drugs to reach the lower BP goal (Cushman and others, 2008).

5.5 Visit Frequency

For both randomized groups, routine visit frequency will be monthly for the first three months after randomization, then every three months for the duration of the trial. Monthly visits will continue in the Intensive Group until SBP <120 mm Hg (or no more titration planned) and in the Standard group whenever SBP $\geq$ 160 mm Hg. Additional visits will be scheduled as needed for management of adverse effects or for monitoring significant medication changes or other clinical issues.

5.6.1 Intensive BP Goal Group (Figure 5.1)

The SBP goal for the Intensive Group, <120 mm Hg, should be achievable in the majority of participants within 8-12 months of follow-up based on the ACCORD experience (The ACCORD Study Group, 2010). For most participants in the Intensive Group, a two- or three-drug regimen of a diuretic and either an ACE inhibitor or ARB and/or a CCB should be initiated at randomization. If a diuretic is contraindicated or not tolerated, an ACE inhibitor or ARB plus a CCB should be initiated. A beta-blocker should be included in the initial regimen, usually in combination with a diuretic, if there is a compelling indication for a beta-blocker. Drug doses should be increased and/or additional antihypertensive medications should be added at each visit in the Intensive Group, usually at monthly intervals, until the participant's goal of <120 mm Hg has been reached or the investigator decides no further antihypertensive medications may be added, which should be a rare exception.

SPRINT provides a unique opportunity to determine both the efficacy and safety of intensive BP control in elderly populations. However, based on limited data, there is a concern that this population may be less tolerant of aggressive BP lowering. Therefore, in participants ≥75 years of age randomized to the intensive BP goal who are on 0-1 antihypertensive medications and have baseline SBP <140 mm Hg, antihypertensive therapy may be initiated with a single agent at the discretion of the investigator with a return visit scheduled in one month. If the participant is asymptomatic at the first post-randomization visit and SBP ≥130 mm Hg, a second agent will be added and titration continued as indicated in above.

Once a participant has achieved SBP <120 mm Hg, the regimen should be reviewed and reinforced with the participant. Compliance with their medications, diet, exercise and other risk reduction therapy should be encouraged. If the participant's BP increases above goal, drug regimens may be adjusted (doses increased or drugs added) as needed until the goal BP is re-established. If BP above goal appears to be secondary to non-adherence (e.g., not taking medications the day of the visit), adherence should be reinforced and participant should be brought back within a month to confirm that BP is at goal.

Figure 5.1 Treatment Algorithm for Intensive Group (Goal SBP < 120 mm Hg)*

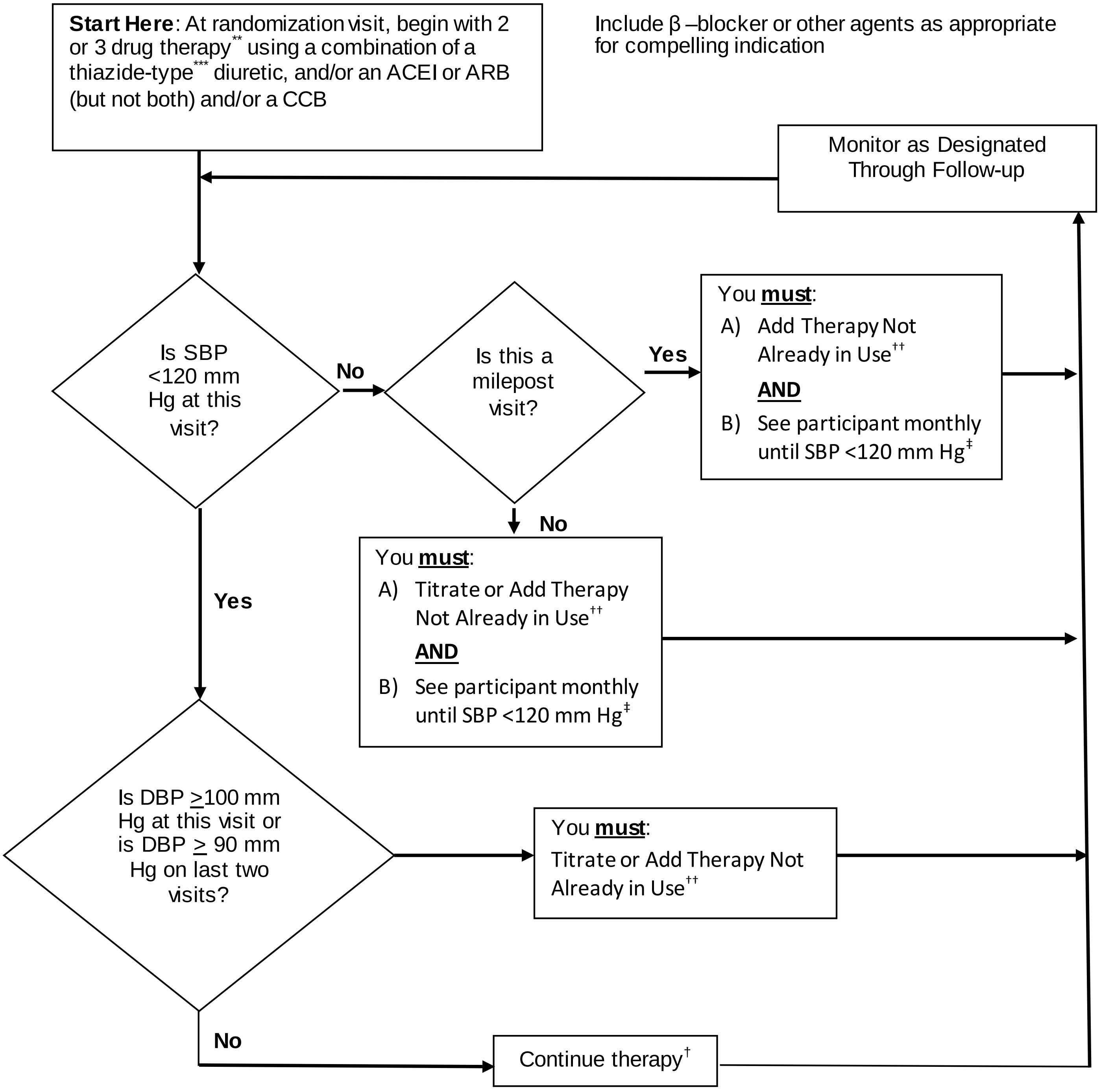

[*] Slightly modified from version found in the SPRINT Protocol

[**] May begin with a single agent for participants 75 years old or older with SBP < 140 on 0-1 meds at study entry. A second medication should be added at the 1 Month visit if participant is asymptomatic and SBP ≥ 130.

[***] May use loop diuretic for participants with advanced CKD

[†] Unless side effects warrant change in therapy

[††] Consider consulting with the Clinical Center Network before adding a fifth anti-hypertensive medication

[‡] Or until clinical decision made that therapy should not be increased further

5.6.2 Milepost Visits (Intensive Group Only)

"Clinical inertia" in hypertension management, where clinicians fail to intensify therapy despite patients not being at goal BP, has been observed in both clinical practice (Berlowitz and others, 1998) and clinical trial settings (Cushman and others, 2002). For this reason, "Milepost Visits" were used in the intensive BP group in the ACCORD trial to assist in reaching goal SBP (Cushman and others, 2007). For all SPRINT participants in the Intensive Group, a series of Milepost Visits dates will be assigned every 6 months throughout follow-up, beginning at the 6-month visit.

Between these dates, antihypertensive medications should be adjusted and/or additional antihypertensive medications should be added within the recommended dose range to achieve the target BP. However, **once a milepost date has been reached and if the participant's BP is not <120 mm Hg, the investigator is required to add an additional antihypertensive drug from a drug class different from what is being taken** to the existing regimen, unless there are compelling reasons to wait, **and see the participant monthly until the SBP goes below 120 mm Hg**.

In situations where the investigator believes that the ***addition of another drug may potentially be harmful to the participant, the requirement may be waived***. This decision must be justified with a Milepost Blood Pressure Drug Exception Form that is data entered at the clinical site. The number of Milepost exception forms will be closely monitored in each SPRINT clinic and regular feedback provided to the clinic for the degree of adherence to the drug protocol.

Milepost Visit procedures do not apply to the Standard Group. Once the Intensive Group participant has been prescribed 5 drugs at maximally tolerated doses, if the BP remains above goal at subsequent Milepost Visits, it will be permitted to substitute a different class into the regimen instead of adding another drug or increasing the dose of a drug. However, additional (more than 5) drugs may be needed to achieve goal SBP in some participants. Medication adherence will be assessed routinely in SPRINT and should be evaluated especially carefully for participants not at goal on 4 or more medications. Strategies to enhance adherence are described in detail in the Manual of Procedures and Adherence Binder.

Action is required at each milepost visit throughout the duration of the study for those intensive group participants who remain above their initial goal pressure of <120 mm Hg.

5.7 Standard BP Goal Group (Figure 5.2)

The SBP goal for the Standard Group, <140 mm Hg, should be achievable in the majority of participants within 3-6 months, based on the ACCORD experience (The ACCORD Study Group, 2010). The standard BP protocol is designed to achieve a SBP of 135-139 mm Hg in as many participants as possible. Participants in this group may or may not be on treatment with one or more antihypertensive medications at the initial intervention visit. The treatment algorithm (MOP Figure 5.2) should be used at the initial visit and subsequent visits to decide on the appropriate regimen. If antihypertensive medication(s) is indicated per protocol, consideration should be given to including a thiazide-type diuretic as initial therapy or as part of the regimen, unless there is a compelling indication for another drug class or intolerance to a thiazide.

At the randomization visit, Standard Group participants on previous antihypertensive drug therapy should be converted to SPRINT medications or no medications, depending on what the investigator believes is most likely to achieve a SBP level between 135-139 mm Hg. Because we expect a decrease in average SBP within the Standard Group following randomization due to improved adherence, lifestyle counseling, and intra-individual variation, sometimes described as “regression to the mean”, treatment should not be intensified at the randomization visit for Standard Group participants unless SBP ≥160 mm Hg or there is a compelling reason to add medication, e.g., management of fluid balance in participants with CKD. Following the randomization visit, medication dose titration or addition of another drug is indicated if SBP is ≥160 mm Hg at a single visit or is ≥140 mm Hg at two successive visits.

Because it is not known if lowering SBP to the more intensive SPRINT goal of <120 mm Hg, compared with the standard goal of <140 mm Hg, is beneficial, neutral, or harmful in patients such as those entered into the SPRINT trial, careful step-down (a reduction of the dose or number of antihypertensive drugs) is allowed for participants in the Standard Group. Down-titration was not permitted in the HOT Trial if DBP was well below the goal for a participant (Hansson and others, 1998) – this likely contributed to the small differences in achieved BP between the three randomized groups and limited the study's ability to detect differences in outcomes. Therefore, down-titration was included in the ACCORD and AASK standard BP protocols and was successful in generating the planned differences in BP between treatment arms. Down titration should be carried out if the SBP is <130 mm Hg at a single visit or <135 mm Hg at two consecutive visits (Figure 5.2).

Figure 5.2 Treatment Algorithm for Standard Group (Goal SBP < 140 mm Hg)

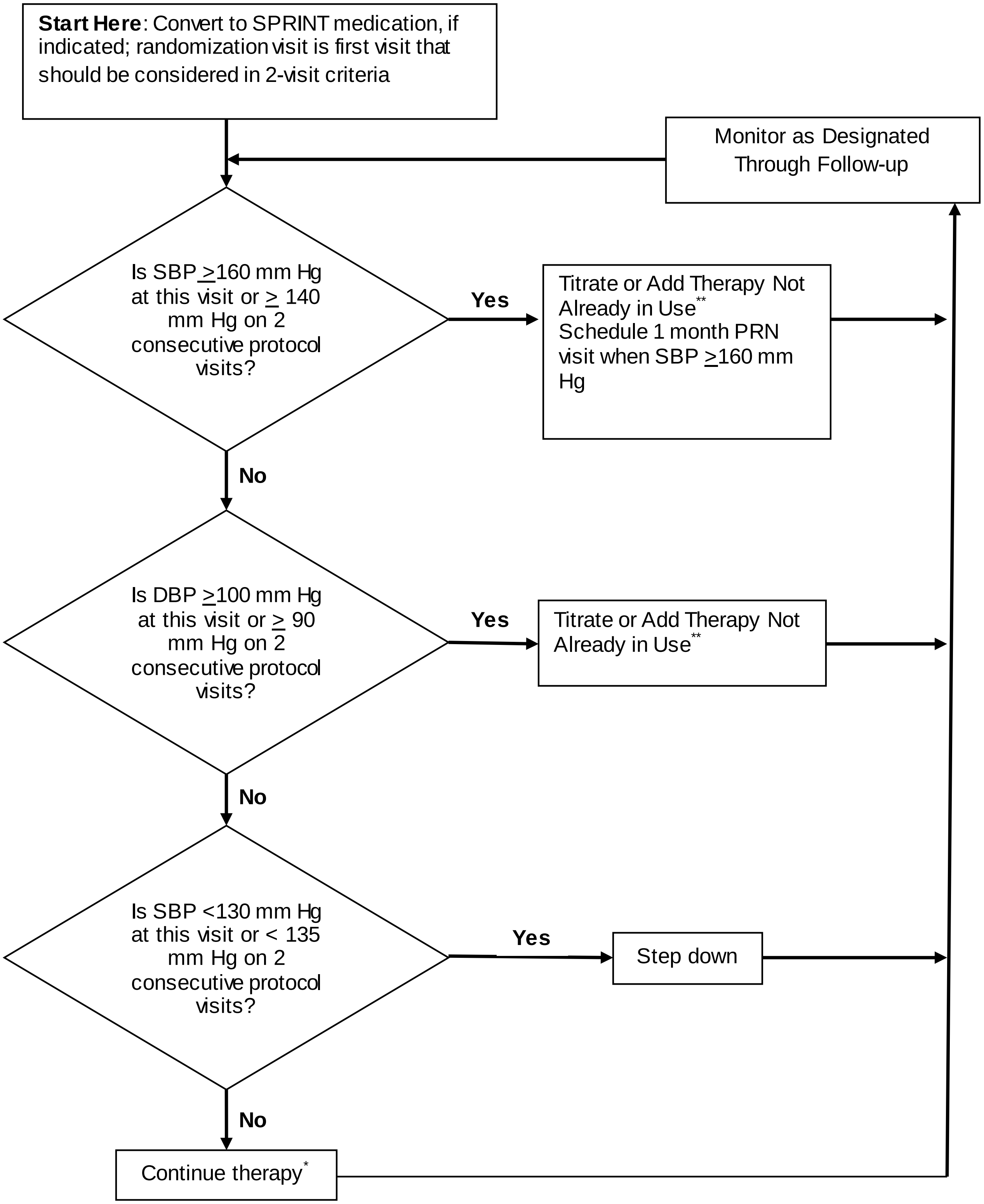

Include β –blocker or other agents as appropriate for compelling indications

* Unless side effects warrant change in therapy

** Consider consulting with the Clinical Center Network before adding a fifth anti-hypertensive medication

5.8 Diastolic Blood Pressure Treatment

Once the SBP goal has been achieved in any participant, the antihypertensive regimen should be intensified if DBP remains ≥100 mm Hg at a single visit or ≥90 mm Hg at two successive visits to achieve DBP <90 mm Hg. The visit intervals and decisions for titration (other than the BP levels) will be similar to those used for the SBP goal. Since beta-blockers reduce DBP more than SBP relative to other antihypertensive medications, a beta-blocker could be considered for such participants (Cushman and others, 2001).

5.9 Use of Home BP Devices

Home BP devices will not be provided to all participants by the trial. Since virtually all BP outcome trials have used office BP determinations and home readings are subject to more bias and error, in SPRINT titration of medications to goal should be based on office readings rather than home BP determinations. However, if home BP readings are available, they may be used to involve participants in order to enhance adherence.

Home readings may also be useful in several additional situations:

1) If significant hypotensive symptoms develop and the home BP is low (or the participant has orthostatic hypotension on home readings), the SPRINT clinic may allow reduction in the dosage of a specific drug, e.g. an alpha blocker, and follow up in clinic.
2) When starting a new drug (in Intensive BP group only), clinic staff/investigator could have the next titration done by participant at home based on level of his/her home BP.

5.10 Safety Issues Related to Initiation and Titration of Medications

Some anti-hypertensive medications, especially ACE inhibitors, ARBs and Diuretics, can result in alterations in serum electrolytes and renal function. Thus, chemistry profiles should be obtained shortly after initiation or up-titration of these medications. Details on collection of these safety labs can be found in the Safety section of the MOP (Section 8.2.1).

5.11 Blood Pressure Medication Alerts

SPRINT participants are likely to be taking multiple medications. We anticipate that most participants will require 2 or more antihypertensive medications to achieve blood pressure treatment goals, and many are likely taking other medications for both acute and chronic conditions besides HTN. Per good clinical practice, the site clinician should review all of the participant's medications, including non-study medications, to look for potential drug-drug interactions before prescribing or adjusting a study medication.

With regards to blood pressure therapy, there are three medication combinations that are strongly discouraged by SPRINT. These are 1) combinations of RAS blockers, 2) the combination of a non-dihydropyridine CCB with a beta-blocker, and 3) high dose Azilsartan (80 mg or more per day) with any diuretic. Submission of a BP Med Log form including one or more of these combinations as current therapy will generate an email to the site coordinator(s),

site PI(s), and CCN coordinator(s) indicating the participant is taking a discouraged combination and directing the recipient to a report on the SPRINT website for additional details. A similar message will also appear on the cover page for the next SPRINT visit packet, unless the combination has been discontinued in the interim. Generally, both the automated emails and the cover page messages will continue as long as the participant remains on the combination. However, in specific cases where the site and CCN investigators agree the use is justified, the CCN investigators may place an override on the SPRINT website to stop further emails for that participant and combination.

Combinations of RAS blockers (ACE inhibitor, ARB, and/or renin inhibitor) are strongly discouraged, based on lack of benefit and adverse outcomes seen in several large randomized controlled trials. The Aliskiren Trial in Type 2 Diabetes Using Cardiovascular and Renal Disease Endpoints (ALTITUDE) was a randomized CVD outcomes trial comparing the renin inhibitor aliskiren versus placebo added to an angiotensin converting-enzyme (ACE) inhibitor or an angiotensin-receptor blocker (ARB) in high-risk participants with type 2 diabetes. A 12/20/11 press release announced the ALTITUDE DSMB stopped the trial due to "… increased risk for non-fatal stroke, renal complications, hyperkalemia, and hypotension in patients taking aliskiren after 18-24 months" and futility for seeing CVD or renal benefits. This follows the results of the ONTARGET trial several years ago, which compared the ARB telmisartan to the ACE inhibitor ramipril, or the combination of the two in a large CV outcomes trial of adults at high risk for CV events (like the HOPE entry criteria). Although ONTARGET showed no difference between the ACE inhibitor and ARB, the combination showed no further CV benefit, but more serious adverse outcomes, especially renal. Therefore, we are discouraging routine usage of combined RAS blockade in SPRINT.

The use of 2 nodal blockers (beta blocker and a non-dihydropyridine calcium channel blocker) is strongly discouraged. Additive reductions in heart rate, cardiac conduction, and cardiac contractility may occur when non-dihydropyridine calcium channel blockers, e.g., verapamil or diltiazem, are used concomitantly with beta blockers. While this combination may be useful and effective in some situations, potentially serious cardiovascular adverse effects such as congestive heart failure, heart block, severe bradycardia, ventricular asystole, and sinus arrest, have been reported. If a non-dihydropyridine CCB (e.g., diltiazem) is to be used, it should not be combined with a beta-blocker.

Azilsartan is available in the U.S. in a single pill combination with chlorthalidone (Edarbyclor®) in 40/12.5 and 40/25 mg doses, but an 80/25 mg dose was not approved/recommended. In clinical trials comparing combinations of high dose Azilsartan and chlorthalidone (80/25 mg dose) to the approved 40/25 mg dose and to maximum dose combinations of other ARBs and HCTZ, the 80/25 fixed dose combination of Azilsartan/chlorthalidone was associated with higher rates of adverse events leading to drug discontinuation, including increases in serum creatinine. In SPRINT, the 80 mg azilsartan dose should **not** be used with any diuretic due to increased risk of SAEs.

In addition to the 3 discouraged medication combinations, there is one combination that is specifically encouraged in SPRINT whenever a participant is prescribed Minoxidil. A few SPRINT participants may require Minoxidil to achieve their BP target, especially those who have failed to achieve control on maximum doses of hydralazine (100 mg BID). Since Minoxidil is a potent vasodilator, participants not receiving adequate doses of a diuretic (usually a loop diuretic with or without a thiazide diuretic) will face a substantial risk of fluid overload. Many patients should also be prescribed a sympatholytic (e.g., beta blocker, guanfacine or clonidine).

Therefore, whenever a participant is prescribed Minoxidil, additional therapy with either chlorthalidone or a loop diuretic is strongly encouraged. Although the diuretic dose requirement may vary from patient to patient, most patients on Minoxidil will require at least 12.5 mg/day chlorthalidone **or loop diuretic equivalent to furosemide** 40 mg/day (usually BID unless prescribed with a thiazide diuretic). Participants on Minoxidil doses above 20 mg/day will require even higher diuretic doses and the need for a combination of a thiazide and loop diuretic is not uncommon. Participants prescribed Minoxidil without also receiving chlorthalidone or a loop diuretic will receive email alerts and cover page messages similar to those described above for discouraged combinations.

5.12 Low seated systolic pressures

The Study Coordinator must notify the site PI or M.D. Co-investigator or his/her designate (M.D., nurse practitioner or physician assistant) and ask for instructions prior to discharging the participant from the study clinic when either:

- The mean seated SBP at a given study visit is below 100 mm Hg AND the participant's mean seated SBP has decreased by at least 10 mm Hg from the last visit,
  OR
- The mean seated SBP is below 90 mm Hg

It should be emphasized that notification of the PI, M.D. Co-investigator or his/her designate about the low SBP does not oblige any action. The M.D. or designate should exercise his/her best clinical judgment on whether action is warranted, taking foremost into consideration the safety of the participant.

5.13 Assessment of Orthostatic Hypotension (OH), Measurement of Standing Blood Pressure

Standing BP will be measured at screening, baseline, 1 month, 6 months, 12 months, and annually thereafter using the same BP device that is used to measure seated BP. After seated determinations, participants will be asked to stand. Beginning when their feet touch the floor, BP will be taken one minute later in the same arm used for the seated measurements, using the BP device. Participants will be asked after the standing determination if they had any symptoms of orthostatic hypotension during the standing BP measurement. The Coordinating Center will calculate BP change using the standing measurements minus the mean of the seated measurements.

Participants with standing SBP <110 mm Hg will not be eligible for randomization (may be rescreened if corrected). However, the detection of asymptomatic orthostatic hypotension, i.e., orthostatic hypotension unaccompanied by orthostatic symptoms of dizziness, presyncope or syncope will not influence the antihypertensive drug treatment algorithm. Symptomatic orthostatic hypotension will be managed as follows.

Definition

Orthostatic hypotension (OH) is usually defined as a decline of systolic blood pressure (SBP) ≥20 mm Hg or a decline of diastolic BP ≥10 mm Hg that occurs within 3 minutes after moving from a supine or seated to a standing position.

Symptoms

OH may be asymptomatic or may be accompanied by dizziness, lightheadedness, feeling faint, or syncope. Generally, asymptomatic OH should not change adherence to the ACCORD protocol, although medication classes may be adjusted to continue appropriate sitting BP control while minimizing postural hypotension (see below).

Predisposing Conditions

In epidemiologic studies OH is more likely to occur in older individuals with high SBP. Diabetes mellitus and other autonomic neuropathies, volume depletion, varicose veins, alcoholism, and certain medications are also associated with OH.

Note: Occasionally, hypotension occurs if a patient previously nonadherent to prescribed medications begins taking the medications. Because BP typically declines after a meal due to splanchnic blood pooling, standing BP should not be measured within 90 minutes after a meal if possible.

Predisposing Medications

Some classes of medications are more likely to cause OH than others. The most frequent offenders currently in use are alpha-blocking drugs, such as prazosin, terazosin, or doxazosin, and central alpha agonists, such as clonidine, methyldopa or guanfacine. Although beta-blockers are perhaps the least likely antihypertensive class to cause OH, one of the most common adverse effects of combined alpha-beta blockers, such as labetolol or carvedilol, is OH or dizziness, primarily because of the alpha-blocking component of these drugs. Rarely, beta-blockers may cause OH because of severe bradycardia (e.g., heart rate <40-50/min) and inability to increase heart rate/cardiac output on upright posture, especially if other drugs or conditions have lowered BP excessively. Occasionally, reserpine, nitrates, or calcium channel blockers may contribute to or cause OH. Certain psychotropic drugs, most notably phenothiazines, can also cause OH. [Consider expanding this list to include some of the older antidepressants.]

Thiazide diuretics rarely cause OH, unless the patient is significantly volume depleted or another agent is added to a diuretic that may cause first-dose hypotension (e.g., a short-acting ACE inhibitor like captopril or a short-acting alpha blocker like prazosin). High dose loop diuretics, such as furosemide, or combinations of diuretics may lead to excessive volume depletion and hypotension, with or without OH.

Management

Patients with poor oral intake, dehydration from whatever cause, GI or renal causes for excessive fluid loss, or hemorrhage, may need to have their diuretic or other antihypertensive medications stopped temporarily until the volume-depletion is corrected. If a participant with symptomatic OH has no obvious cause of excessive volume depletion, the medication regimen should be reviewed. Psychotropic drugs may need to be changed to alternative agents (many newer ones are equally effective without hypotensive effects) or reduced in dose. If the patient is on an alpha-blocker, alpha-beta blocker, or central alpha agonist, the dose should be reduced or the potentially offending agent discontinued and, if necessary for BP control, replaced with another class of antihypertensive drug less likely to cause symptomatic OH. If the participant requires an alpha-blocker for BPH/bladder outlet obstructive symptoms, a more selective alpha-blocker (e.g., tamsulosin) may be considered.

Patients with symptomatic OH should eat small meals and avoid standing up rapidly after eating. Such individuals should avoid hot showers and other excessive heat exposure. An increase in sodium intake may be considered in patients without hypertension or heart failure. In those with large varicose veins, fitted elastic hose or compression stockings may reduce venous pooling in the legs. In refractory cases of symptomatic OH, drug therapy with vasoconstrictors such as midodrine or dihydroergotamine or with mineralcorticoids may be considered, but this should be unlikely to be needed in SPRINT.

Implications for protocol

The presence of orthostatic hypotension, particularly if asymptomatic, should not be considered as an exclusion from the study, particularly since some studies indicate that control of hypertension can lead to improvement in orthostatic hypotention. Likewise, decisions regarding uptitration toward protocol blood pressure targets should be made primarily on the basis of the seated blood pressure measurements. Orthostatic hypotension should also not preclude uptitration toward blood pressure targets. When symptomatic, it is suggested that initial efforts described above directed toward identification of other causes (such as psychotropic drugs, recent meal ingestion) and preferential use of hypertension medication classes that have less predilection to orthostatic hypotension.

5.14 Consultation with CCN Office (PI)

Consultation with your local CCN Office or your CCN Intervention Subcommittee representative about a participant's regimen may be requested at any time for any concern relative to the participant, protocol or regimen. It is strongly recommended that such contact occur, if a participant is on 4 or 5 antihypertensive agents and remains above their assigned goal, or prior to discontinuing study therapy because of non-serious adverse effects. Consultation with the CCN office is also required prior to prescribing medications in the SPRINT restricted use formulary.

5.15 Lifestyle Recommendations and Background Therapy

The lifestyle and background therapy section of the protocol addresses a broad range of therapies for patients with hypertension. The Lifestyle and Background Therapy protocol reflects national and (when available) evidence-based guidelines. These therapies are recommended in order to foster high-quality care in all SPRINT participants.

The background therapy recommendations include smoking cessation counseling, anti-thrombotic therapy, and treatment of dyslipidemia. Making uniform recommendations in these areas is intended to reduce bias by increasing the likelihood that background therapies that alter the risk of cardiovascular events, and that are not being studied in SPRINT, are utilized equally across all study arms. The background therapy recommendations should be provided to the participants and their physicians in written form (see section XXX below). Background therapy is considered part of usual recommended care for hypertension and, as such, is not covered by research study costs. The delivery of these background therapies will be left up to the participants' own physicians.

Lifestyle therapy is intended as an adjunct to drug therapy. It is anticipated that both lifestyle modifications and medication interventions will be required to meet goals for blood

pressure control. Lifestyle and MNT recommendations are equivalent between the intensive and standard treatment arms.

5.15.1 Medical Nutrition Therapy (MNT)

The overall goal of MNT is to assist individuals with hypertension in making changes in nutrition and exercise habits leading to improved blood pressure control. MNT consists of dietary modification and weight management. Participants in SPRINT should receive detailed instruction in MNT at the baseline visit and reminders of appropriate lifestyle changes at every visit Instruction should be delivered by the SPRINT clinic staff (nutritionist or similarly qualified clinician.) The following recommendations and general principles will apply to all participants in SPRINT, regardless of randomized assignment.

5.15.2 Weight Management

Moderate weight loss (5-9 kg [10-20 lb.]), irrespective of starting weight, has been shown in short-term studies to reduce blood pressure. Although long-term data assessing the extent to which these improvements can maintained is not available, several strategies can be recommended:

1. Moderate calorie restriction (250-500 calories less than average daily intake as calculated from a food record. Participants needing assistance with monitoring their food intake can be referred to the menu planner available at http://hp2010.nhlbihin.net/menuplanner/menu.cgi) and a nutritionally adequate food plan with a reduction of total fat, especially saturated fat.
2. Spacing of meals in which calorie intake is distributed in 3 or more meals/snacks throughout each day
3. Increase in physical activity (see section X.2.4)

Participants who are considered overweight (BMI $\geq$ 25 kg/m^2) should be encouraged to lose 10% of their current weight or 5-9 kg, whichever is less, over a 6 month period of time. Once weight is lost, weight maintenance strategies should be implemented. Participants who are not overweight should be encouraged to maintain weight. Weight will be measured at all clinic visits, providing an opportunity for feedback and counseling.

5.15.3 Dietary Modification

Dietary modifications will be recommended based both on blood pressure control and control and prevention of CVD risk factors. The main focus of nutrition intervention for SPRINT participants without chronic kidney disease will be the DASH Diet recommendation. Patients with stage 3 or greater will receive alternate dietary instruction. Recommendations will also be made for alcohol intake.

The DASH diet is an eating plan that is low in saturated fat, cholesterol, and total fat. It emphasizes fruits, vegetables, fat-free or low-fat milk and milk products, and includes whole grain products, fish, poultry and nuts. Red meat, sweets, added sugars and sugar-containing beverages are reduced on the DASH eating plan. The DASH diet is rich in potassium, magnesium and calcium as well as protein and fiber.

SPRINT participants who do not have chronic kidney disease should be introduced to the DASH diet by study coordinators. Each participant will receive a copy of the DASH diet prepared especially for SPRINT participants. Study coordinators should review the table that provides the number of daily servings for the DASH eating plan (see Box 3 of the DASH diet) with study participants.

Dietary recommendations for participants with chronic kidney disease

The DASH is high in nutrients and foods which can be detrimental to participants with chronic kidney disease. Participants meeting the criteria for chronic kidney disease should instead be counseled on a prudent renal diet and provided with the National Kidney Foundation educational material (see http://www.kidney.org/atoz/index.cfm). Specifically, patients with chronic kidney disease should be counseled to limit foods that are high in phosphorus (see Appendix 5A). They may also need to limit their potassium intake (see Appendix 5B for a list of food high in potassium) and their intake of protein. Protein-rich foods include red meat, poultry, fish and other seafood, eggs, dairy product and vegetables and grains. SPRINT participants with chronic kidney disease, due to their many dietary restrictions, may need additional dietary counseling by a trained clinical nutritionist. Referrals to clinical nutritionists should be done in conjunction with the participant's primary health care provider.

Sodium

Dietary sodium intake should be reduced to less than 2400 mg/day for the SPRINT study population and to less than 2000 mg/day for those with chronic kidney disease.
High sodium foods should be avoided, including chips, nuts, lunch meats, most canned foods, most fast foods, pickles and olives. Instead of adding salt to foods for flavor, encourage trying pepper, lemon juice, mustard, garlic, spices, herbs and salt substitutes. Participants with chronic kidney disease should be counseled to discuss use of salt substitutes with their health care provider or clinical nutritionist before using. Many of these products substitute potassium for sodium. Increased potassium intake is not recommended for patients with CKD. Emphasize choosing more fresh foods - vegetables, fruits, grains, meats, and minimally processed foods. Encourage more home preparation of foods and reading food labels to help make lower sodium choices.

Alcohol

It is recommended that trial participants limit daily alcohol intake to no more than one ounce (30 mL) of ethanol for men and 0.5 ounce (15 mL) for women. One ounce of ethanol is equivalent to 24 ounces (720 mL) of beer, (2 bottles of beer), 10 ounces (300 mL) of wine, (2 glasses), or two ounces (60 mL) of 100- proof hard liquor.

5.15.4 Physical Activity

Physical activity is a crucial component of lifestyle therapy for hypertension; thus, at least brief counseling for physical activity should be included at all clinic visits. Participants should be encouraged to accumulate 30 minutes or more of moderate-intensity aerobic physical activity on 5 or more days of the week. Moderate-intensity aerobic activity is defined as repetitive motion using large muscle groups that increases the heart rate to 50-70% of maximal, is perceived as fairly light to somewhat hard, or is equivalent in perceived intensity to brisk walking (3-4 miles per hour for most people, or walking "as if you are in a hurry"). Maximal

heart rate can be estimated by subtracting age from 220. For example, in a 60 year old the target heart rate range is (220 – 60) X (0.5) to (220 – 60) X (0.7), or 80 to 112 beats per minute. Persons on beta-blockers cannot use the heart rate criterion, as beta-blockers prevent the increase in heart rate, so perceived exertion or comparable intensity to brisk walking should be recommended.

Thirty minutes of physical activity may be accumulated in bouts of 8-10 minutes in a 24-hour period. Warm-up and cool-down activities should be encouraged. Participants should be instructed to drink plenty of fluids, to wear socks and appropriate footwear, and to inspect their feet on a daily basis. Clinics should develop and maintain lists of low cost or free local resources for safe physical activity to provide to participants. The general exercise prescription above may need to be modified for some participants. The following groups of participants will need tailored instructions:

SPRINT participants who do little or no physical activity at baseline should be encouraged to increase their physical activity levels gradually, starting with lower-intensity, shorter-duration, and less frequent activities (e.g., moderately paced walking for 5 minutes twice a week) and increasing gradually over weeks or months to moderate-intensity and longer-duration activities until the goal of 30 minutes or more of moderate-intensity aerobic physical activity on 5 or more days of the week is achieved.

For SPRINT participants beginning an unsupervised exercise program or increasing their intensity of physical activity, screening for coronary heart disease should be considered. **Persons continuing their current regular physical activity or increasing duration of activity at the same intensity do not need this screening.** The recommended screening is an exercise stress test, or documentation of an exercise stress test within the previous 3 months, that is negative for ischemia and significant arrhythmias at a workload of 4-5 METS (i.e., moderate intensity, equivalent to brisk walking).

Persons experiencing symptoms of ischemia during physical activity should undergo diagnostic evaluation. If a participant complains of chest pain, shortness of breath or other equivalent angina symptoms, please consult the SPRINT clinic provider to determine if immediate referral is needed or if the evaluation can be scheduled by the participant's main health care provider.

5.15.5 Counseling approaches for behavior change

Counseling can assist SPRINT participants in achieving lifestyle changes. Although SPRINT is not a trial of lifestyle changes, modifications as described above can help SPRINT participants achieve their blood pressure goals

Behavioral counseling approaches are designed to help patients make changes in their lifestyles in order to achieve the recommendations for diet, physical activity, and weight. The underlying principle of these counseling approaches is to engage in an interchange where counselor and patient work together toward a common goal. The counseling is highly individualized based on the particular patient's motivation, past experience, knowledge, and personal circumstances.

Behavioral approaches shown to be effective include self-assessment, goal setting, self-monitoring, identifying barriers and influences, problem solving, and receiving feedback and reinforcement. A brief description of these approaches follows along with a description as to how to combine them.

Self-assessment: The patient determines their current motivation for making behavior changes. Patients with their counselor determine their current diet and physical activity behaviors and weight status and how close they are to the recommendations. Keeping a record of current diet and physical activity patterns is part of the initial self-assessment. Many people do not realize what their actual behavioral patterns are without engaging in some directed self-assessment activities. For example, one patient may not realize that she snacks in front of the television every evening until she writes down everything she eats and when and where she eats it.

Goal setting: The patient sets individualized, realistic goals for their diet and physical activity behaviors and their weight. For example for diet, one patient may select switching from whole milk to low-fat milk to reduce his/her calories from fat; another patient may select eliminating desserts. For physical activity, one patient may select walking for five minutes each day to start becoming more active, while another patient may select swimming twice a week. For weight, one patient may select calorie restrictions that should result in losing 2 pounds in the next month; another patient may decide not to work on weight yet, but to focus on achieving a more healthful diet. There are more successes in behavior change when small, achievable goals are selected first and then increased gradually to achieve the recommended diet and physical activity behaviors. Goal setting is revisited at each session.

Self-monitoring: Self-monitoring consists of keeping regular records of one's own behaviors, such as diet and physical activity patterns, or one's own weight. Diet and physical activity diaries can be kept by writing down everything an individual eats for 3 days in a row, or writing down all the moderate-to-vigorous physical activities he/she does for a week. These diaries should not only include what is eaten, or what activities are performed, but the time of day and context (for example, in the evening in front of the television, or after work). Self-monitoring helps the patient and counselor determine the patient's progress toward goals and identify specific issues that might need to be addressed. For example, one patient may see that she never completes her 15-minute walk on weekdays after work because her work demands are too high. This recognition can help the patient identify alternative approaches to obtaining physical activity, for example walking first thing in the morning before she goes to work. Continual self-monitoring has proven very effective in weight loss and in achieving dietary and physical activity changes.

Identifying Barriers and Influences: The patient identifies personal barriers he/she might have to implementing changes, including lack of knowledge or skills, low motivation, lack of social support, environmental constraints, etc. The patient also identifies any positive influences that might aid them in making changes. Two common barriers/influences are social support and environment. It is very difficult to make behavior changes in an unsupportive social environment, and engaging a person's spouse, other family member, or friend in providing support may be very important. For example, a husband can provide social support for physical activity by offering to go for a walk with his wife. It is difficult for some people to ask for social support, so practice in asking can be incorporated into a counseling session. The influence of the immediate environment is also very important. For example, if a patient keeps a supply of potato chips and beer in the house, he is more likely to snack on these when at home. Alternately, if grocery shopping is limited to only healthful foods, unhealthful foods will not be available in the home to eat on a whim. Similarly, buying exercise equipment and putting it in an obvious

location, for example in front of the television, is an environmental cue to exercise that has been shown to be effective.

Problem solving: Problem-solving to overcome barriers or to increase positive influences follows the identification of the barriers and influences. The patient identifies possible things that can be done and then selects viable options to try. For example, one patient may say she cannot exercise because she has to take care of her grandchildren. One possible way to overcome that barrier is to take the children to the park and walk with them on the trails. The solutions should emanate from the patient, not be imposed by the counselor.

Feedback and Reinforcement: Feedback is simply providing back to the patient a description of what he/she is doing, e.g., describing the diet and physical activity patterns. For example, you may review a patient's diet diary with him/her and provide feedback that he/she is still eating dessert every night. Feedback is nonqualitative. Reinforcement, on the other hand, is a qualitative assessment of a patient's actions by giving praise or a small reward for goals achieved or positive efforts. The counselor can provide verbal reinforcement, and/or the patient can reinforce him or herself by some reward. For example, a patient could treat herself to buying a new dress when she achieves her weight goal.
These various approaches are combined in regular, ongoing counseling sessions. Each session should build on the previous one, and the sessions should continue throughout the patient's treatment. If the patient achieves the goals, then the sessions should focus on maintaining the changes. The effective use of behavioral counseling is an "art" and requires practice, but here is one scenario for the first three sessions.

Session 1: Self-assessment
Identify motivation for making changes
Have the patient start keeping track of diet and physical activity behaviors in a diary
Session 2:
Review self-assessment diaries
Reassess motivation
Set short-term goals
Identify barriers to achieving the goals
Problem solve
Start self-monitoring
Session 3:
Review self-monitoring results
Assess progress toward the short-term goals
Provide feedback and reinforcement
Identify barriers and influences
Problem solve
Set new short-term goals, or confirm previous goals
Continue self-monitoring

5.15.6 Smoking Cessation

Cigarette smoking will be ascertained at baseline on the **SPRINT Baseline Medications and Physical Exam Form** and annually on the **SPRINT Annual Medications and Physical**

Exam History Form. All participants who are tobacco users should be strongly encouraged to stop using a brief, unambiguous, strong and personalized message. Current smokers' willingness to quit may be assessed. Smokers who are interested in quitting may be provided with self-help materials, referred to their regular source of medical care or a smoking cessation program, or assisted by the SPRINT clinician. If a SPRINT participant desires pharmaceutical assistance (e.g. Chantix) to assist with smoking cessation, they should be referred to their primary healthcare provider.

All participants who are tobacco users will be strongly encouraged to stop. The widely accepted AHRQ guidelines and model include the following steps:

- ASK – each participant about tobacco use
- ADVISE – current smokers to quit using a brief, unambiguous, strong and personalized message
- ASSESS – current smokers' willingness to quit
- ASSIST – current smokers who express willingness to make a quit attempt by developing a quit plan, encouraging adjunctive pharmacotherapy, and providing supplementary materials
- ARRANGE follow-up, either by a health care provider or in a specialized smoking cessation program

5.15.7 Anti-thrombotic Therapy

While aspirin use will be appropriate for most SPRINT participants (unless contraindicated by allergy, bleeding disorder, recent GI bleeding or need for anticoagulant therapy), the decision to recommend daily aspirin and the dose should be deferred to the participant's primary healthcare provider. Aspirin use will be assessed at baseline and annually thereafter.

5.15.8 Treatment of Dyslipidemia

Treatment of elevated lipids is recommended by national guidelines (REF). SPRINT participants will have regular assessment of lipids and those with lipid levels outside of Report of the Expert Panel on Detection, Evaluation and Treatment of High Blood Cholesterol in Adults (currently third report but if new guideline are published, the most current report will be used) will be provided with information on recommended behavioral and/or pharmacologic treatments. In general, lifestyle modification will be the first line therapy followed by treatment with a statin medication for those not meeting goal lipid levels. The SPRINT study will not supply lipid-lowering therapy, but will provide educational material that the participant can share with their health care provider.

Appendix 5.A: Foods that are high in Phosphorus

HIGH PHOSPHORUS FOOD TO LIMIT OR AVOID

**Beverages**	ale	beer
	chocolate drinks	cocoa
	drinks made with milk<br>canned iced teas	dark colas
**Dairy Products**	cheese	cottage cheese
	custard	ice cream
	milk	pudding
	cream soups	yogurt
**Protein**	carp	crayfish
	beef liver	chicken liver
	fish roe	organ meats
	oysters	sardines
**Vegetables**	***dried beans and peas:***	
	baked beans	black beans
	chick peas	garbanzo beans
	kidney beans	lentils
	limas	northern beans
	pork ' n beans	split peas
	soy beans	

**Other foods**	bran cereals	brewer's yeast
	caramels	nuts
	seeds	wheat germ
	whole grain products	

Appendix 5.B: Foods that are high in potassium
HIGH POTASSIUM FOODS TO LIMIT OR AVOID

Fruits	Vegetables	Other foods
Apricot , raw (2 medium) dried (5 halves)	Acorn squash	Bran/Bran Products
Avocado (¼ whole)	Artichoke	Chocolate (1.5-2 ounces)
Banana (½ whole)	Bamboo Shoots	Granola
Cantaloupe	Baked Beans	Milk, all types (1 cup)
Dates (5 whole)	Butternut squash	Molasses (1 Tablespoon)
Dried fruits	Beets, fresh then boiled	Nuts and seeds (1 ounce)
Figs, dried	Black Beans	Peanut Butter (2 tablespoons)
Grapefruit Juice	Broccoli, cooked	Salt substitute/Lite salt
Honeydew	Brussels sprouts	Salt free broth
Kiwi (1 medium)	Chinese Cabbage	Yogurt
Mango(1 medium)	Carrots, raw	Snuff/chewing tobacco
Nectarine(1 medium)	Dried Beans and Peas	
Orange(1 medium)	Greens, except Kale	
Orange Juice	Hubbard Squash	
Papaya (½ whole)	Kholrabi	
Pomegranate (1 whole)	Lentils	
Pomegranate Juice	Legumes	
Prunes/Prune Juice	Mushrooms, canned	
Raisins	Parsnips	
	Potatoes, white and sweet	
	Pumpkin	
	Rutabagas	
	Spinach, cooked	
	Tomatoes/tomato products	
	Vegetable Juice	

Chapter 6. DDC

6.0 Central Drug Distribution Procedure

6.1 Responsibilities for Drug Accountability

The principal investigator (PI) at each participating clinical site is responsible for a complete and accurate accounting of all SPRINT study materials received and dispensed by the facility. The Drug Distribution Center (DDC) in Albuquerque, New Mexico, will provide forms, instructions, and assistance as necessary to assure proper use and accountability of all study drugs and supplies. Each center is responsible for adhering to any local policies, applicable state, and federal regulations concerning custody, dispensing, and disposition of drugs and supplies.

6.2 Description of SPRINT Study Drugs, Devices, and Expendable Supplies

Open Label and Ancillary Supplies

Supplies (drugs and ancillary supplies) used in SPRINT are provided as open-label items. All are supplied in commercial packaging, which can be dispensed directly to the patient without transfer to another container (“unit of issue” packaging). **Open label** medications will contain the manufacturer’s label (drug, strength, quantity, expiration date, and lot number). In addition, a 2-part label affixed by the DDC contains study name, unique identification number, bar codes, and applicable federal warnings. Bottles are shipped in packages containing two or four (4) bottles; the shrink wrapped tray of bottles is labeled as SPRINT Study Drug with generic name.

Pill splitters are provided to the site from the DDC to be used when a dosage requires splitting of a tablet. Rolls of prescription labels are provided to the sites at their request, if needed.

Sample for outside package label of supplies and drug

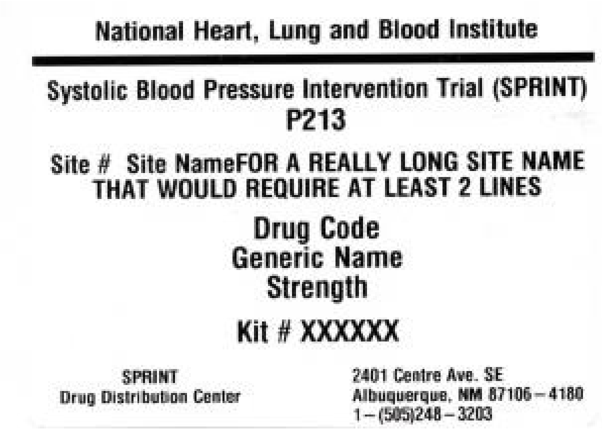

Sample for bottle label

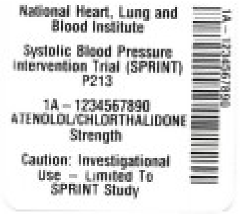

Sample for Prescription label

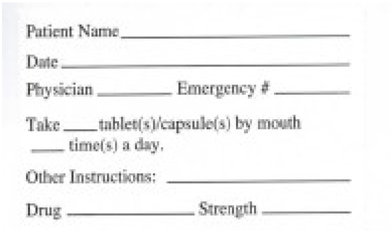

Patient Name ____________________

Date ____________________

Physician ________ Emergency # ________

Take ____ tablet(s)/capsule(s) by mouth
____ time(s) a day.

Other Instructions: ____________________

Drug ____________ Strength ________

6.3 Custody and Storage Requirements

The SPRINT study supplies should be stored between 15° and 30°C (59° and 86°F) in a secure area. Drugs and supplies may be stored either in the study clinic or pharmacy depending on local policy. If the study drug is not stored in a pharmacy, it must be kept in a locked room or locked cabinet, or under "double-locked" storage if so required. Items should be stored in a specific area and distinguished from non-SPRINT medications.

6.4 Shipment of Study Materials

6.4.1 Initial Shipment

After receiving notification from the Wake Forest Data Coordinating Center that all required regulatory documents have been received from the clinical site and the site is eligible to receive medication and supplies, the DDC will send the clinical site an initial shipment of study materials. The initial shipment is based on standard initial par levels set by the study. Subsequent shipments are based on par levels established by the individual site to accommodate local prescribing practice. New sites must complete all required paperwork before medications will be sent. A **Shipping Notice** listing items and quantities of all supplies shipped is included with each shipment. Shipping notices should be filed with study documents. Inventory that is shipped to the site will also be automatically uploaded to the web-based inventory system

6.4.2 Subsequent Shipments and Par Level Maintenance

Changes in inventory levels and drug/supply needs at each site must be communicated by the Site to the DDC. Each time a SPRINT supply item is dispensed to a patient the bar code on the label must be scanned to the website and through this mechanism the item is assigned to the patient and inventory is adjusted accordingly. When a specific drug inventory reaches the re-order point a replacement order will be automatically generated on a weekly basis. Data is transmitted nightly and orders are generated Friday mornings and will ship the following week. Any additional drug requests should be submitted by Thursday close of business to be added to the weekly orders. Thus clinical sites should not need to routinely manually order study drugs and supplies from the DDC. Sites are responsible for timely electronic submission of usage date to maintain adequate inventory. Inventory par levels may be adjusted at the request of the site. If an inventory question or problem arises or additional study drug/supply item is needed, contact the DDC at (505) 248-3203. In addition, contact the DDC if the increased use is planned or expected,.so the sites inventory can be increased.Par levels and "on hand" inventory can be reviewed on the SPRINT website. All changes should be communicated to the DDC via phone or email. Site name, number, and contact information should be included on all correspondence.

6.4.3 Restricted Use Items

The formulary includes select restricted use items. The study coordinator must submit the request to the CCN PI (CCN Intervention Representative) for approval. Once approved, the site should email the DDC with the approval included.

6.5 Prescriptions for Study Drug

All study drugs and supplies must be ordered on either standard prescribing practices such as a prescription blank or a physician order form. Each time study drug is dispensed the investigator or other authorized physician should write a prescription or order with the following:

1. Patient's name and current address
2. Study name [SPRINT Trial]
3. Participant I.D.
4. Drug Name
5. Strength
6. Quantity to be dispensed
7. Directions for use
8. Signature of investigator or other authorized physician
9. Date prescription or order is written
10. Refills (if applicable). A new prescription may be required at each study visit where medication is dispensed.

6.6 Dispensing

Open label drugs for SPRINT are dispensed in the manufacturer's packaging. A 2-part label is affixed to each item.

The peel-off portion of this label (bar code that runs vertically on right side of label) should be removed and placed in the appropriate section of the SPRINT **"Drug Dispensing Form."** . **It is extremely important that these bar-code labels be scanned to the website as quickly as possible so that the information is entered into the SPRINT study web database. Electronic information is managed daily by Coordinating Centers and the DDC for inventory tracking. Importantly, supplies are replenished based upon this data, which is received through this scanning process. <u>Maintaining up-to-date information in the electronic database is critical for ensuring an adequate drug supply at your clinical site.</u>** Bottles should not be split. Full bottles should always be given to the participant. A first in, first out inventory process should be used to properly maintain drug supply.

6.7 Labeling

The clinical site is responsible for complying with regulations on prescription labeling for prescription legend drugs. **Do not cover labels applied by the DDC or the manufacturer's lot number and expiration date.** Prescription labels should contain the following information at a minimum:

1. Rx Date
2. Prescription Number (if dispensed by pharmacy)
3. Patient's Name
4. Drug Name, Strength and Quantity
5. Directions for use
6. Prescriber's Name
7. Name of facility
8. Other information required by State or Local laws.

The site is responsible for labeling all dispensed medication in accordance with state and federal regulations.

6.8 Expired Drugs and Supplies

The DDC strongly recommends that sites print a list of drugs that will expire within the next 90 days (<3 months) from the dispensing website each month. This report is site-specific and lists the bottle ID numbers that will expire in the next 3 months. This form can be used for removal of expired drug once the DDC replenishes the inventory. The list will be sorted by drug name and strength. Each bottle number, lot, and expiration date is included in the data. All expired drugs that are removed for destruction can be noted on the form. Once scanned, new replenishment supply will be shipped to the site by the DDCUnless otherwise directed by the DDC all expired SPRINT drugs and supplies will be destroyed by the local clinical sites in accordance with local policies for drug destruction. They are not to be returned to the DDC. When destroying expired drug, clinical sites must enter the drug destruction function on the SPRINT web site and scan the bottle to a generic destruction bar code. Through this mechanism, expired drugs are assigned to a corresponding destruction bar code. This process updates inventory at the clinical site. The **"SPRINT Local Destruction Documentation"** form should then be printed through the website for documentation of study drug disposition at the clinical site. Data regarding the destruction of expired drugs are necessary to adjust the remaining inventory and maintain appropriate supplies.

6.9 SPRINT Supplies from Site to Site

Once drugs and supplies are shipped from the DDC to a clinical site, products may not be returned to the DDC. Any excess SPRINT supplies may be transferred to another clinical site as needed. A site that wishes to transfer a drug must complete their portion of the Drug Transfer Form and include that form in the shipping box. Upon arrival of the drug(s), the receiving site will complete their portion of the Drug Transfer Form and email it immediately to the DDC. Once the form is received by the DDC, the supplies will be transferred in the electronic database from the original site to the receiving site. This form can be found on the SPRINT Website.

6.10 Loss of Inventory

Any loss of inventory should be documented in writing (memorandum or e-mail) and sent to the Coordinating Center and the Drug Distribution Center. The document will be signed by the Principal Investigator and will include a complete description of circumstances relating to the loss, listing quantities of drug, supplies or devices, and corrective action to prevent future losses of inventory.

6.11 Website Reports

Reports on dispensing, destruction, par levels, and inventory can be located on the SPRINT website. Instructions for performing quarterly drug inventories can be found on the SPRINT website.

The Drug Distribution Center address is as follows:

ATTN: SPRINT Trial Project Manager
VA Cooperative Studies Program
Clinical Research Pharmacy Coordinating Center
2401 Centre Avenue, SE
Albuquerque, NM 87106-4180

Telephone: (505) 248-3203
FAX: (505) 248-3205
E-mail: Orders and par changes: ABQCSPSPRINTORDER@va.gov.
Subject Line: SPRINT Drug Order - Site #xxx
Brandi.Dillard@va.gov
Robert.ringer@va.gov

Central Chemistry Laboratory Biospecimen Collection and Processing Manual

University of Minnesota
Advanced Research and Diagnostic Laboratory

SPRINT Study website - https://www.sprinttrial.org

Biospecimen Collection and Processing Table of Contents

1. **BACKGROUND** 5

2. **PREPARATION**.. 6

   2.1. Staff Certification Requirements.. 6
   2.2. Blood Collection Trays and Tubes.. 6
   2.3. Laboratory Specimen Collection Form at Participant Arrival 7

3. **VENIPUNCTURE PROCEDURE**.. 10

   3.1. Phlebotomy Room.. 10
   3.2. Participant Preparation.. 10
   3.3. Venipuncture... 12
   3.4. Blood Tube Mixing and Storage During Venipuncture 16

4. **BLOOD AND URINE PROCESSING**.. 17

   4.1 Operating the Centrifuge.. 17
   4.2 Visit Specific Processing 17
   4.3 Urine Collection 21
   4.4 Overview of Specimen Collection 23

5. **PACKAGING AND SHIPPING** 27

   5.1 Packaging and Shipping Instructions.. 28

6. **QUALITY CONTROL** 34

   6.1. Quality Control Clinic Performance... 34
   6.2. Internal Laboratory Control 34
   6.3. Reporting Results 35

7. **TRAINING PROCEDURES**... 35

8. **SPECIMEN STORAGE PLAN** 36

APPENDIX:

1. Tests, Reference Ranges, and Alert Values 37
2. Equipment and Supplies 38
3. Daily Temperature Logs.. 40
4. Venipuncture and Processing Certification.. 41
5. Sample Exam for Certification... 42
6. Supply Reorder Form.. 43
7. Redraws or Missed Sample Collections 44
8. Sample Destruction.. 45
9. SPRINT Chemistry Profiles…………………………………………………………46

BIOSPECIMEN COLLECTION AND PROCESSING

1. Background

The objective of the **S**ystolic Blood **Pr**essure **In**tervention **T**rial (SPRINT) is to conduct a multicenter, randomized clinical trial to determine whether treating systolic blood pressure (SBP) to a lower goal than currently recommended will reduce cardiovascular disease (CVD). The proposed trial will test the effects of intensive SBP lowering compared with standard lowering, specifically a comparison of a SBP goal of <120 mmHg versus <140 mmHg for a SBP difference of 10-15 mmHg between the two randomized groups. Although epidemiologic evidence strongly suggests that lowering SBP will reduce CVD risk in nearly all adults, for practical and public health reasons the hypothesis is most efficiently studied in high-risk individuals. Thus, the trial will recruit patients 55 years or older with SBP ≥130 mmHg and at least one additional CVD risk factor. Three high risk groups will be excluded – patients with diabetes, patients with polycystic disease, and patients who have had a stroke – because they are the target groups of ongoing NIH trials that are testing a lower BP goal. SPRINT will focus on three other high risk groups: patients with clinical CVD other than stroke, patients with stage 3 chronic kidney disease (estimated glomerular filtration rate 30-59 mL/min/1.73 m^2), and patients without clinical CVD who have other risk CVD factors (e.g., smoking, low levels of HDL, high levels of LDL). This trial is expected to enroll 50% women and 30% members of minority groups (African Americans, Hispanics, Native Americans, and Asians) according to the approximate prevalence of hypertension in these various subgroups. Blood and urine samples are collected to study these factors through specialized, state-of-the-art laboratory assays.

The SPRINT study plans to randomize approximately 9,250 participants, which are to be recruited through five Clinical Center Network (CCN) hubs and their associated clinics. These CCN hubs include: Case Western, Southeast, University of Alabama-Birmingham, Utah, and the VA. There are approximately 80 clinical sites associated with the five CCN hubs. Five additional academic centers will serve as scientific and logistical support centers: Coordinating Center (Wake Forest University), Central Laboratory (University of MN), Drug Distribution Center (VA Medical Center), MRI reading center (University of Pennsylvania), and an ECG reading center (Wake Forest University).

The Central Laboratory performs the tests on the blood and urine specimens donated by the study participants who have been asked to fast for at least 8 hours for the RZ, 12MO, 24MO, and 48MO visits. Non-fasting samples are acceptable for the other visits. The clinical sites will collect serum, EDTA plasma, EDTA whole blood, and urine samples. The packed cells of the EDTA whole blood and aliquots of serum, plasma, and urine collected at the clinical sites will be processed and stored at the Central Laboratory. The SPRINT Central Laboratory is located at the University of Minnesota Advanced Research and Diagnostic Laboratory in Minneapolis MN. A complete list of the tests performed is located in Appendix 1.

Laboratory tests are performed on specimen samples that are collected, centrifuged, and shipped by the clinical sites. Probably the most important step in this process (and potentially the most difficult to standardize) is the blood collection and handling at the clinical sites. Laboratory tests can be repeated, but if the blood sample itself is not correctly collected, centrifuged, and shipped, the laboratory results may not be accurate even if the laboratory assays are precise. For the study to succeed, it is important that variation in measurement values reflect true differences between the study participants rather than differences in blood collection or processing procedures. Thus, it is important that all clinical site technicians are

well-trained, certified, and fully compliant with the protocol for drawing and processing the specimens in the field, and also willing to take pride and responsibility in their work.

2. PREPARATION

Since participation in this study is voluntary, every effort must be made to make the entire procedure as easy and painless as possible for participants. Technicians must remain calm and project an attitude of competence even when faced with the most nervous or inquiring participant. The best way to achieve this is for the technicians to be thoroughly knowledgeable about all aspects of the procedures. The SPRINT study collects 7.5 – 41 mL of blood, depending on which visit, from each participant. Depending on which visit, one to five tubes of blood are collected. The technician should reassure any participant who is concerned about the volume of blood collected that the total amount drawn is only about 1 – 3 tablespoons, although it may look like more to them. The technician may also assure participants that they donate almost 10 times as much blood (450 mL) when they donate a pint of blood.

2.1. Staff Certification Requirements

Blood drawing and processing are performed by a certified SPRINT technician(s) at each clinical site. The technicians complete a central training course taught by certified laboratory staff. Each technician must complete the training before becoming SPRINT certified. New staff will be trained by clinical site supervisory personnel. Recertification takes place annually and is authorized by the clinical site supervisory personnel.

2.2. Blood Collection Trays and Tubes

Prior to a scheduled participant visit, the technician prepares a tray for each participant. The tray holds the blood collection tubes and a plastic transport vial for the urine sample which are ultimately shipped daily to the Central Laboratory for analysis and storage. Label these sets of tubes/vial with the appropriate code numbers for the participant. A list of equipment, suppliers, and vendors is provided in Appendix 2.

2.2.1 Blood Collection Tray

First, the technician organizes and prepares the blood collection tray. The blood collection tray is made of hard unbreakable plastic that can be easily cleaned. The tray has individual compartments that are filled with the following supplies:

- test tube rack to hold the blood collection tubes
- sterile, disposable 21 gauge butterfly needles
- plastic Vacutainer tube guides
- Vacutainer Luer adapters
- sterile alcohol swabs
- gauze sponges
- tourniquet
- bandages ("Band Aids")

Ammonia spirits ampules, ice packs, and wash cloths should be readily available in the blood collection area for participants who become faint during the blood collection.

2.2.2 Blood Collection Tubes/Urine Sample

Technicians must be familiar with: the arrangement of blood collection tubes, the order in which the tubes are to be filled, the type of anticoagulant in each tube, and the possible sources of error in handling each tube. These tubes are organized in the test tube rack according to the specific visit. Each visit will include a different combination of tubes; see visit processing charts provided on pages 23-25 of this manual. The list below refers to the organization and types of tubes for each specific visit.

Randomization Visit:

Tubes #1 and #2 are 7.5 mL red stoppered tubes. Although these tubes do not contain anticoagulant, they do have a clot activator and therefore require mixing following collection. Invert these tubes 8 times to mix thoroughly. The serum from these tubes will be used for testing lipids (fats), glucose, creatinine and other biochemical markers.

Tube #3 is a 10 mL lavender-stoppered tube containing EDTA anticoagulant and requires mixing following collection. Invert this tube 8 times to mix thoroughly. This tube will be used for the collection of packed cells to be used for DNA isolation.

Tubes #4 and #5 are 8.0 mL lavender-stoppered tubes containing EDTA anticoagulant and a gel separator. These tubes require mixing following collection. Invert these tubes 8 times to mix thoroughly. The plasma from these tubes will be stored for future analytical tests. (Plasma will be transferred from the collection tubes into a 10.0. mL, clear plastic, purple capped, Transport Tube for shipping).

Urine sample will be collected and shipped in a clear plastic 10 mL, blue cap, Transport Tube.

1 Month, 3 Month, 18 Month, 30 Month, 42 Month, and 54 Month and PRN Visits:

Tube #1 is a 7.5 mL red stoppered tube. Although this tube does not contain anticoagulant, it does have a clot activator and therefore requires mixing following collection. Invert this tube 8 times to mix thoroughly. The serum from this tube will be used for testing creatinine and other biochemical markers.

6 Month, 36 Month, and 60 Month Visits:

Tube #1 is a 7.5 mL red stoppered tube. Although this tube does not contain anticoagulant, it does have a clot activator and therefore requires mixing following collection. Invert this tube 8 times to mix thoroughly. The serum from this tube will be used for testing creatinine and other biochemical markers.

Urine sample will be collected and shipped in a clear plastic 10 mL, blue cap, Transport Tube.

12 Month, 24 Month, and 48 Month Visits:

Tube #1 and #2 are 7.5 mL red stoppered tubes. Although these tubes do not contain anticoagulant, they do have a clot activator and therefore require mixing following

collection. Invert these tubes 8 times to mix thoroughly. The serum from these tubes will be used for testing lipids (fats), glucose, creatinine and other biochemical markers.
No tube #3

Tubes #4 and #5 are 8.0 mL lavender-stoppered tubes containing EDTA anticoagulant and a gel separator. These tubes require mixing following collection. Invert these tubes 8 times to mix thoroughly. The plasma from these tubes will be stored for future analytical tests. (Plasma will transferred from the collection tubes into a clear plastic 10.0. mL, purple capped, Transport Tube for shipping)

Urine sample will be collected and shipped in a clear plastic 10 mL, blue cap, Transport Tube.

2.2.3 Blood Collection Tubes: Labeling and Set-Up

Blood collection tubes can be set up in advance of the participant visit. Remove the tubes from the kit for labeling. Labeling the tubes in advance will also improve workflow efficiency. The Central Laboratory will provide the SPRINT Clinical Sites with pre-made kits specific to each visit type. There are four different kit types and are labeled as follows: Randomization; (1, 3, 18, 30, 42, and 54 Month and PRN Visits); (6, 36, and 60 Month Visits); (12, 24, and 48 Month Visits). Place the Styrofoam container containing the gel pack inside the -20 freezer 24 hours in advance so the gel pack will be frozen the next day and ready to use for shipping. Do NOT freeze the outer cardboard sleeve of the shipping box.

It is important to note that **blood collection tubes do expire** and this will have to be monitored by the clinical sites. The expiration date is printed on all of the blood collection tubes within the kit. **Helpful Hint:** Label the kit bag with the expiration date of the collection tube(s) with the shortest outdate. The collection tubes within the kit expire at different times. Extra blood collection tubes can be ordered to have on hand for when tubes expire or difficult or problematic collection situations arise.

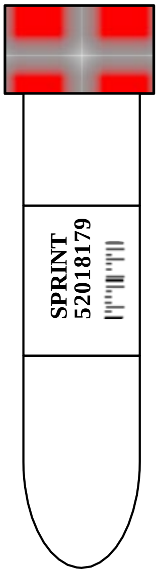

1. Obtain appropriate visit specific kit. Apply pre-numbered barcode laboratory ID labels to each blood collection tube. Place the labels on the tubes with the bar-code oriented from the bottom of the tube to the top of the tube. Handle only one participant's specimens at a time so the chance of mislabeling is minimized.

2. Arrange the blood collection tubes in the test tube rack in the same order in which they are to be collected. The five tubes are collected in the following order according to specific visit:

   Randomization Visit:

   Tube #1: 7.5 mL red stoppered tube (Serum)
   Tube #2: 7.5 mL red stoppered tube (Serum)
   Tube #3: 10 mL lavender stoppered tube (EDTA)
   Tube #4: 8 mL lavender stoppered tube (EDTA) with gel separator
   Tube #5: 8 mL lavender stoppered tube (EDTA) with gel separator
   10.0 mL plasma Transport Tube (for tubes #4 and #5)

1 Month, 3 Month, 18 Month, 30 Month, 42 Month, and 54 Month and PRN Visits:

Tube #1: 7.5 mL red stoppered tube (Serum)

6 Month, 36 Month, and 60 Month Visits:

Tube #1: 7.5 mL red stoppered tube (Serum)

12 Month, 24 Month, and 48 Month Visits:

Tube #1: 7.5 mL red stoppered tube (Serum)
Tube #2: 7.5 mL red stoppered tube (Serum)
Tube #3: **Not Collected**
Tube #4: 8 mL lavender stoppered tube (EDTA) with gel separator
Tube #5: 8 mL lavender stoppered tube (EDTA) with gel separator
10.0 mL plasma Transport Tube (for tubes #4 and #5)

3. Additional laboratory ID number labels will be used when the participant arrives to provide a documented match between their SPRINT participant ID number and the laboratory specimen ID number on the Laboratory Specimen Collection Form.

4. After the tubes have been removed from the kit for labeling; place the Styrofoam container containing the gel pack inside the -20 freezer so it will be ready for shipping.

2.2.4 Preparation for Specimen Collection

In the morning, prior to drawing blood from the participants:

1. Check to make sure the blood collection tray is properly equipped. Every item on the checklist must be ready before proceeding.
2. Check that each Vacutainer tube and transport container is properly labeled with the correct laboratory barcode ID label.
3. Perform and record QC check on refrigerator temperature (4°C ± 2°C).
4. Perform and record QC check on freezer temperature (-20°C ± 2°C).
5. Perform and record QC check on room temperature. (Optimum 19°C-25°C) (Acceptable 17-27°C).

2.3 Laboratory Specimen Collection Form at Participant Arrival

1. Obtain the pre-printed Laboratory Specimen Collection Form via the SPRINT website that corresponds to the participant visit.

2. Check that the participant's SPRINT Participant ID number on the Laboratory Specimen Collection Form is correct. Place the laboratory ID label that matches the label on the collection tubes and plasma and urine transport containers onto the Laboratory Specimen Collection Form.

3. Confirm the match between the participant name, the SPRINT participant ID number, and the laboratory ID number on the blood collection tubes, plasma and urine transport containers, and the Laboratory Specimen Collection Form.

3. VENIPUNCTURE PROCEDURE

Handle all specimens as potentially infectious for laboratory phlebotomists and staff. Blood borne pathogens such as hepatitis B and human immunodeficiency virus (HIV) can be transmitted following contact of a tainted blood sample through "broken skin" or intact mucous membrane (mouth, eyes, or nose) or as a result of an inadvertent needle stick. Examples of "broken skin" include open cuts, nicks and abrasions, dermatitis, and acne. OSHA rules mandate that technicians always wear disposable protective gloves when collecting and processing specimens. When performing a venipuncture, the protective gloves worn by the phlebotomist must be intact (e.g., a fingertip cannot be torn off of the glove in order to locate a venipuncture site). If the phlebotomist accidentally sustains a stick with a contaminated needle, clean the wound thoroughly with disinfectant soap and water, notify a supervisor, and consult the SPRINT physician. Never take lab coats worn during the collection and processing of samples outside of the laboratory area except for laundering. Before leaving the laboratory, the technician will remove the lab coat and disposable gloves and wash hands with a disinfectant soap.

Use OSHA-approved cleaning solution to clean up any spills of blood, plasma, serum, or urine. Use this solution to clean all laboratory work surfaces at the completion of work activities. OSHA regulations require that all needles and sharp instruments be discarded into puncture resistant containers. Do not attempt to bend, break, or recap any needle before discarding it. Discard the butterfly set following each specimen collection. Do not perform any pipetting by mouth; especially of any blood, plasma, serum, or urine.

Avoid formation of potentially infectious aerosols when removing the rubber stoppers from Vacutainer tubes. In addition to wearing protective gloves, hold a piece of gauze over the stopper while slowly removing it from the tube. Creation of aerosols can also be diminished by careful pipetting and centrifugation techniques. Further steps to minimize infection risk while processing samples are described in the OSHA regulations stated in the Federal Register of December 6, 1991 (Vol. 56, No. 235, page 64177). Wear a mask in combination with an eye protection device, such as goggles or glasses with solid side shields or a chin-length face shield when working with potentially infectious materials that have the potential for splashing, spraying, or spattering. An alternative to these devices would be a desk-mounted or under-shelf-mounted clear plastic shield, which would offer similar protection from possible infectious splashes or sprays.

Place all blood-contaminated products in biohazard bags for proper disposal.

Universal Precautions websites: http://www.osha.gov or http://www.niehs.nih.gov

3.1. Phlebotomy Room

The blood drawing takes place in an isolated room or in a room with dividers. The room is equipped with all of the necessary blood drawing supplies. A separate work area is equipped

with all of the supplies that are used in the blood processing. The centrifuge, refrigerator, and freezers should be nearby.

3.2. Participant Preparation

Informed consent must be obtained before drawing any blood, to ensure that the participants understand the purpose and possible complications of the venipuncture procedure. A standard informed consent has been prepared for this study. The consent statement informs study participants that although there may be some minor discomfort, their blood (about 1-3 tablespoons) will be drawn by trained technicians. The consent also states that a copy of clinically important test results will be sent to their respective clinical site and that they will be contacted if clinically important tests are abnormal.

Before blood is collected, the participant is asked the following safety questions:

1. …if they have had a radical mastectomy or other surgery where lymph nodes were removed from their armpits. If they have, blood should not be collected from the arm where this has occurred.
2. …whether he/she has a bleeding disorder. If such a disorder is present, ask the participant whether he/she has had blood drawn previously and if so, whether he/she had any problems with excessive bleeding or bruising at the venipuncture site. When the participant reports a bleeding disorder, specify the type of bleeding disorder(s) as briefly as possible in the Blood Collection/Processing Comments section of the Laboratory Specimen Collection form. In general, a bleeding disorder is not a reason for participant deferral. A gauze and tape bandage is applied. The participant is instructed to maintain pressure on the venipuncture site for 2 minutes and to keep the bandage on the site for the remainder of the examination visit.
3. …if they have ever had a graft or shunt for kidney dialysis. If they have, blood should not be collected from the arm where this has occurred.

Complete the remainder of Laboratory Specimen Collection form:

1. Complete the Blood Collection section of the form that includes the date and time of collection, as well as the initials of the phlebotomist.

2. Indicate if the participant has been fasting for >8 hours on the collection form. **Note: Fasting for >8 hours is required for the RZ, 12MO, 24MO, and 48MO visits. The (1, 3, 18, 30, 42, 54 Month); (6, 36, 60 Month); and (PRN) visits do NOT need to be fasting.**

3. After the venipuncture, use the checkboxes for each tube to indicate draws. If you are unable to obtain a specific tube, please indicate the reason why in the “Comments” section of the Laboratory Specimen Collection Form.

4. After the participant has provided a urine sample, complete the Urine Collection section of the form that includes date and time of the urine collection, as well as the initials of the technician who processes the urine.

The participant should be seated during the blood draw. It is difficult to standardize the length of time that a person is in the sitting position prior to venipuncture, but to the extent possible

attempt to have the participant be sitting for a minimum of five minutes. This allows the participant to relax before the venipuncture takes place.

Perform venipuncture with a 21-gauge butterfly needle and 12 inches of plastic tubing between the venipuncture site and the blood collection tubes. The butterfly has a small thin-walled needle that minimizes trauma to the skin and vein. The use of 12 inches of tubing allows tubes to be changed without any movement of the needle in the vein. Give the participant enough time to feel comfortable both before and after the blood collection. In many cases the most memorable part of the experience for participants will be the contact with the technicians who draw the blood and their general attitude and competence.

If the participant is nervous or excited, the technician briefly describes the procedure, e.g., "I am going to be drawing about 3 tablespoons of blood. This blood will be used in tests for lipids (fats), glucose (sugar), and other biochemistry tests. We hope to be able to use the results of these tests to better understand health risks related to high blood pressure." HANDLING PARTICIPANTS WHO ARE EXTREMELY APPREHENSIVE ABOUT HAVING BLOOD DRAWN: Do not under any circumstances force the participant to have blood drawn. It may help to explain to the participant that the blood drawing is designed to be as nearly painless as possible. It is sometimes best to let the participant go on with another part of the visit. It may also be helpful to have the participant relax in the blood drawing chair just so the phlebotomist can check the veins in the participant's arms, without actually drawing blood. If the participant is very anxious, he/she may lie down during the blood collection. A reclining individual will undergo an extravascular water shift, resulting in a dilutional effect on lipid values. If this option is taken, note it on the Laboratory Specimen Collection Form.

3.3. Venipuncture

With jacket or sweater removed, have the participant sit upright with the sleeves rolled up to expose the antecubital fossa (elbow). Use a tourniquet to increase venous filling. This makes the veins more prominent and easier to enter. The preferred arm to draw from is the left arm. Use the right arm only if blood collection is not possible from the left arm. This does not mean you must stick the left arm. Only do so if an adequate vein is apparent.

PRECAUTIONS WHEN USING A TOURNIQUET: The tourniquet should be on the arm for the shortest time possible. Never leave the tourniquet on for longer than two minutes. To do so may result in hemoconcentration or a variation in blood test values. If a tourniquet must be applied for preliminary vein selection, and it remains on the arm for longer than two minutes, it should be released and reapplied after a wait of two minutes. Instruct the participant that he/she should not clench their fist prior to the venipuncture. Doing so could cause fluctuations in the results in several of the analytes being measured. If the participant has a skin problem, put the tourniquet over the participant's shirt or use a piece of gauze or paper tissue so as not to pinch the skin.

A. **Apply tourniquet.**
   1. Wrap the tourniquet around the arm 3 to 4 inches (7.5 to 10.0 cm) above the venipuncture site.
   2. Tuck the end of the tourniquet under the last round.
   3. If a Velcro tourniquet is used, adhere the ends to each other.

B. **Identify vein:** Palpate and trace the path of veins several times with the index finger. Unlike veins, arteries pulsate, are more elastic, and have a thick wall. Thrombosed veins lack resilience, feel cord-like, and roll easily. If superficial veins are not readily apparent, lowering the extremity over the arm of the chair will allow the veins to fill to capacity. Identify the best available vein.

C. **Assemble the butterfly-Vacutainer set.**
1. Attach the Luer adapter to the Vacutainer holder.
2. Attach the Luer end of the butterfly needle set to the Luer adapter.

D. **Cleanse the venipuncture site.**
1. Remove alcohol prep from its sterile package.
2. Cleanse the vein site with the alcohol prep using a circular motion from the center to the periphery.
3. Allow the area to dry to prevent possible hemolysis or contamination of the specimen and a burning sensation to the patient when the venipuncture is performed.
4. If venipuncture becomes difficult, the vein may need to be touched again with a gloved hand. If this happens, cleanse the site again with alcohol and allow the skin to dry.

E. **Perform venipuncture.** (**Randomization Visit)**
1. Grasp the participant's arm firmly, using your thumb to draw the skin taut. This anchors the vein. The thumb should be 1 or 2 inches (2.5 or 5.0 cm) below the venipuncture site.
2. With the needle bevel upward, enter the vein in a smooth continuous motion.
3. Once blood appears in the butterfly tubing, place tube #1 (7.5 mL red top) into the Vacutainer holder. Grasp the flange of the needle holder and push the tube forward until the butt end of the needle punctures the stopper, exposing the full lumen of the needle.
4. Make sure the participant's arm is in a flat or downward position while maintaining the tube below the site when the needle is in the vein. It may be helpful to have the participant make a fist with the opposite hand and place it under the elbow for support. DO NOT HAVE THE PARTICIPANT MAKE A FIST IN THE HAND OF THE ARM FROM WHICH BLOOD IS TO BE DRAWN.
5. Remove the tourniquet after tube #1 fills. Once the draw has started, do not change the position of a tube until it is withdrawn from the needle. The tourniquet may be reapplied if blood flow is slow without it. If the color of the arm turns red or blue, the tourniquet is applied too tightly. Loosen it and continue.
6. Keep a constant, slight forward pressure (in the direction of the adapter) on the end of the tube. This prevents release of the shutoff valve and stopping of blood flow. Do not vary pressure nor reintroduce pressure after completion of the draw.
7. Fill remaining Vacutainer tubes #2 (7.5 mL red top), #3 (10 mL EDTA), #4 (8 mL EDTA), and #5 (8 mL EDTA). Fill all Vacutainer tubes as completely as possible; i.e., until the vacuum is exhausted and blood flow ceases. If a Vacutainer tube fills only partially, remove the tube and attach another without removing needle from vein.
8. When the blood flow into the collection tube ceases, remove the tube from the holder. The shutoff valve covers the point, stopping blood flow until the next tube is inserted (if necessary). Gently invert each tube eight times immediately following removal of the tube from the adapter while the next tube is filling. (See section 3.4 for mixing instructions.)

9. To remove the needle, lightly place clean gauze over venipuncture site. Remove the needle quickly and immediately apply pressure to the site with a gauze pad. Discard needle with its cap into needle box. DO NOT ATTEMPT TO RECAP NEEDLES! Have the participant hold the gauze pad firmly for one to two minutes to prevent bruising.
10. If the blood flow stops before collecting all of the tubes, repeat the venipuncture on the participant beginning with the first unfilled tube. As always, the tourniquet must never be on for longer than two minutes.
11. Skip to letter F. Troubleshooting.

E. **Perform venipuncture**. **(1, 3, 18, 30, 42 54 Month, PRN and Visit 6, 36, 60 Month Visit)**

1. Grasp the participant's arm firmly, using your thumb to draw the skin taut. This anchors the vein. The thumb should be 1 or 2 inches (2.5 or 5.0 cm) below the venipuncture site.
2. With the needle bevel upward, enter the vein in a smooth continuous motion.
3. Only one blood collection tube is collected. Once blood appears in the butterfly tubing, place tube #1 (7.5 mL red top) into the Vacutainer holder. Grasp the flange of the needle holder and push the tube forward until the butt end of the needle punctures the stopper, exposing the full lumen of the needle.
4. Make sure the participant's arm is in a flat or downward position while maintaining the tube below the site when the needle is in the vein. It may be helpful to have the participant make a fist with the opposite hand and place it under the elbow for support. DO NOT HAVE THE PARTICIPANT MAKE A FIST IN THE HAND OF THE ARM FROM WHICH BLOOD IS TO BE DRAWN.
5. Remove the tourniquet after tube #1 fills. Once the draw has started, do not change the position of a tube until it is withdrawn from the needle. The tourniquet may be reapplied if blood flow is slow without it. If the color of the arm turns red or blue, the tourniquet is applied too tightly. Loosen it and continue. As always, the tourniquet must never be on for longer than two minutes.
6. Keep a constant, slight forward pressure (in the direction of the adapter) on the end of the tube. This prevents release of the shutoff valve and stopping of blood flow. Do not vary pressure nor reintroduce pressure after completion of the draw.
7. Fill the Vacutainer tube as completely as possible; i.e., until the vacuum is exhausted and blood flow ceases. If the Vacutainer tube fills only partially, remove the tube and attach another without removing needle from vein.
8. When the blood flow into the collection tube ceases, remove the tube from the holder. The shutoff valve covers the point, stopping blood flow until the next tube is inserted (if necessary). Gently invert the tube eight times immediately following removal of the tube from the adapter. (See section 3.4 for mixing instructions.)
9. To remove the needle, lightly place clean gauze over venipuncture site. Remove the needle quickly and immediately apply pressure to the site with a gauze pad. Discard needle with its cap into needle box. DO NOT ATTEMPT TO RECAP NEEDLES! Have the participant hold the gauze pad firmly for one to two minutes to prevent bruising.
10. Skip to letter F. Troubleshooting.

E. **Perform venipuncture. (12, 24, 48 Month Visit)**

1. Grasp the participant's arm firmly, using your thumb to draw the skin taut. This anchors the vein. The thumb should be 1 or 2 inches (2.5 or 5.0 cm) below the venipuncture site.
2. With the needle bevel upward, enter the vein in a smooth continuous motion.
3. Once blood appears in the butterfly tubing, place tube #1 (7.5 mL red top) into the Vacutainer holder. Grasp the flange of the needle holder and push the tube forward until the butt end of the needle punctures the stopper, exposing the full lumen of the needle.
4. Make sure the participant's arm is in a flat or downward position while maintaining the tube below the site when the needle is in the vein. It may be helpful to have the participant make a fist with the opposite hand and place it under the elbow for support. DO NOT HAVE THE PARTICIPANT MAKE A FIST IN THE HAND OF THE ARM FROM WHICH BLOOD IS TO BE DRAWN.
5. Remove the tourniquet after tube #1 fills. Once the draw has started, do not change the position of a tube until it is withdrawn from the needle. The tourniquet may be reapplied if blood flow is slow without it. If the color of the arm turns red or blue, the tourniquet is applied too tightly. Loosen it and continue.
6. Keep a constant, slight forward pressure (in the direction of the adapter) on the end of the tube. This prevents release of the shutoff valve and stopping of blood flow. Do not vary pressure nor reintroduce pressure after completion of the draw.
7. Fill remaining Vacutainer tubes #2 (7.5 mL red top), #4 (8 mL EDTA), and #5 (8 mL EDTA).Tube #3 is NOT collected for these visits. Fill all Vacutainer tubes as completely as possible; i.e., until the vacuum is exhausted and blood flow ceases. If a Vacutainer tube fills only partially, remove the tube and attach another without removing needle from vein.
8. When the blood flow into the collection tube ceases, remove the tube from the holder. The shutoff valve covers the point, stopping blood flow until the next tube is inserted (if necessary). Gently invert each tube eight times immediately following removal of the tube from the adapter while the next tube is filling. (See section 3.4 for mixing instructions.)
9. To remove the needle, <u>lightly</u> place clean gauze over venipuncture site. Remove the needle quickly and immediately apply pressure to the site with a gauze pad. Discard needle with its cap into needle box. DO NOT ATTEMPT TO RECAP NEEDLES! Have the participant hold the gauze pad firmly for one to two minutes to prevent bruising.
10. If the blood flow stops before collecting all of the tubes, repeat the venipuncture on the participant beginning with the first unfilled tube. As always, the tourniquet must never be on for longer than two minutes.
11. Go to letter F. Troubleshooting.

F. **Troubleshooting:** If a blood sample is not forthcoming, the following manipulations may be helpful.

1. If there is a sucking sound, turn needle slightly or lift the holder in an effort to move the bevel away from the wall of the vein.
2. If no blood appears, move needle slightly in hope of entering vein. Do not probe. If not successful, release tourniquet and remove needle. A second attempt can be made on the other arm. The same technician should not attempt a venipuncture more than twice (once in each arm). If a third attempt is necessary, a different phlebotomist should attempt the venipuncture.

3. Loosen the tourniquet. It may have been applied too tightly, thereby stopping the blood flow. Reapply the tourniquet loosely. If the tourniquet is a Velcro type, quickly release and press back together. Be sure, however, that the tourniquet remains on for no longer than two minutes at a time.

G. **Bandaging the arm.**

1. Under normal conditions:
   a. Slip the gauze pad down over the site, continuing mild pressure.
   b. Apply an adhesive or gauze bandage over the venipuncture site after making sure that blood flow has stopped.
2. If the participant continues to bleed:
   a. Apply pressure to the site with a gauze pad. Keep the arm elevated until the bleeding stops.
   b. Wrap a gauze bandage tightly around the arm over the pad.
   c. Tell the participant to leave the bandage on for at least 15 minutes.

H. **Precautions** - When a Participant Feels/Looks Faint Following the Blood Drawing:

1. Have the person remain in the chair. If necessary, have him/her lie on the floor with legs elevated. Use of a transfer belt may be indicated in this situation.
2. Take an ammonia spirits ampule, crush it, and wave it under the person's nose for a few seconds.
3. Provide the person with a basin if he/she feels nauseous.
4. Have the person stay seated until the color returns and he/she feels better.
5. Have someone stay with the person to prevent them from falling and injuring themselves if he/she should faint.
6. Place a cold wet cloth on the back of the person's neck or on their forehead.
7. Once the episode has passed, some fruit juice may be given to the participant in order to counteract any possible hypoglycemia due to their pre-clinic visit fast.
8. If the person continues to feel sick, take a blood pressure and pulse reading. Contact a medical staff member for further direction.

3.4. Blood Tube Mixing and Storage During Venipuncture

All tubes must be mixed with the anticoagulant to prevent clotting. Even tubes #1 and #2 that do not contain an anticoagulant, have a clot activator that needs to be mixed with the blood. Begin by holding the tube horizontal to the floor. Gently tip the stopper end down while watching the air bubble rise to the butt (1st inversion). Now, lower the butt end slightly while watching the bubble float to the stopper (2nd inversion). Lower the stopper end again when the bubble reaches the stopper. This is the third inversion. Invert each tube eight times, which should take 6 to 8 seconds.

Tube #1 and #2: 7.5 mL red stoppered tube containing no anticoagulant. Invert tube gently 8 times immediately after collection. Place tubes in collection rack at room temperature and allow the blood to clot for 20-30 minutes after collection.

Tube #3: 10 mL lavendar-stoppered tube contains EDTA anticoagulant. Invert gently 8 times immediately after collection. Store this tube in the refrigerator until same-day shipment to the Central Laboratory on frozen gel pack.

Tube #4 and #5: 8.0 mL lavendar-stoppered tube contains EDTA anticoagulant and a gel separator. Invert gently 8 times immediately after collection. Place the tubes #4 and #5 in refrigerator for 20-30 minutes until centrifugation.

4. BLOOD AND URINE PROCESSING

4.1 Operating the Centrifuge

Refer to Centrifuge Operating Manual for specific operating and balancing instructions. In order to achieve 1800-1900 RCF (g) within the centrifuge, the corresponding revolutions per minute (RPM) vary from centrifuge to centrifuge depending on the radius of the centrifuge's rotor. **Consult the centrifuge's operating manual for the appropriate RPM to achieve 1800-1900 RCF (g) for each centrifuge. Note: 1800-1900 is the optimal RCF (g) speed; if 1800-1900 RCF is unattainable in a certain centrifuge, then1200-1300 RCF (g) is the minimal acceptable speed.** To balance the centrifuge, place tubes of the same size and with equal volume of blood as determined visually in opposite positions in the bucket adaptors. For tubes of blood that do not have another tube of equivalent blood volume, use a "balance tube" of the same size containing an equivalent volume of water. Wait for centrifuge to come to a complete stop before opening the lid.

The centrifuge should be routinely cleaned monthly, as directed in the Operator's Manual. We recommend removing the trunions (label or take note of the positions), soaking them for 20 minutes in Virex (Johnson Wax Company) diluted 1-128. Klero-ro may also be used. Rinse well, and allow to dry overnight. Wipe out the centrifuge with Virex diluted 1-128. Replace the trunions in the same positions as you removed them to ensure correct balancing. In case of a blood spill, wipe out the blood using 0.5% sodium hypochlorite (household bleach diluted 1:10), and then clean the centrifuge in the same manner as you do for monthly maintenance.

4.2 Visit Specific Processing

RANDOMIZATION VISIT

4.2.a Stage One: Immediate Processing

After completion of venipuncture:

1. Tubes #1 and #2 remain at room temperature for 20-30 minutes to allow the blood to clot (blood at 4°C clots extremely slowly). Set a timer for 30 minutes as a reminder to centrifuge these tubes.

2. Place tubes #4 and #5 in the refrigerator for 20-30 minutes. Set a timer for 30 minutes as a reminder to centrifuge these tubes.

3. Place tube #3 in refrigerator until daily shipment on frozen gel pack to the Central Laboratory. (This tube is NOT centrifuged).

4.2.a Stage Two: Processing of Serum/Plasma

Stage two begins approximately 30 minutes after venipuncture.

1. As close to 30 minutes after venipuncture as possible, spin the red top tubes #1 and #2 (blood must be clotted) and the 8.0 mL purple top/gel EDTA tubes #4 and #5 at 1800-1900 RCF (g) for 10 minutes (if swinging bucket type) at room temp. Increase the time to 15 minutes if using a fixed angle type centrifuge. Consult the centrifuge manual for appropriate RPM setting to achieve 1800-1900 RCF (g).

2. When the centrifuge has come to a complete stop, remove tubes #1 and #2 and place them in a wire rack. These tubes are ready to be prepared for shipping. If packaging for shipment cannot be done immediately, place the tubes in the refrigerator until they can be prepared for shipping. Tubes #1 and #2 need no further processing before shipping.

3. When the centrifuge has come to a complete stop, remove tubes #4 and #5 and place them in a wire rack. These tubes are ready to be prepared for shipping. **Process Tubes #4 and #5 accordingly: Label a purple capped, 10.0 mL Transport Tube with the same Lab ID number that is on tubes #4 and #5. Using a plastic transfer pipette, transfer all of the plasma from tubes #4 and #5 into one purple cap, 10.0 mL Transport Tube before shipping. After the transfer of plasma is complete, the empty tubes #4 and #5 can be discarded into the biohazard waste. If packaging for shipment cannot be done immediately, place the tubes in the refrigerator until they can be prepared for shipping.**

   Note: *Avoid formation of potentially infectious aerosols when removing the rubber stoppers from Vacutainer tubes. In addition to wearing protective gloves, hold a piece of gauze over the stopper while slowly removing it from the tube. Creation of aerosols can also be diminished by careful pipetting and centrifugation techniques. Further steps to minimize infection risk while processing samples are described in the OSHA regulations stated in the Federal Register of December 6, 1991 (Vol. 56, No. 235, page 64177). Wear a mask in combination with an eye protection device, such as goggles or glasses with solid side shields or a chin-length face shield when working with potentially infectious materials that have the potential for splashing, spraying, or spattering. An alternative to these devices would be a desk-mounted or under-shelf-mounted clear plastic shield, which would offer similar protection from possible infectious splashes or sprays.*

Plasma Transfer Process

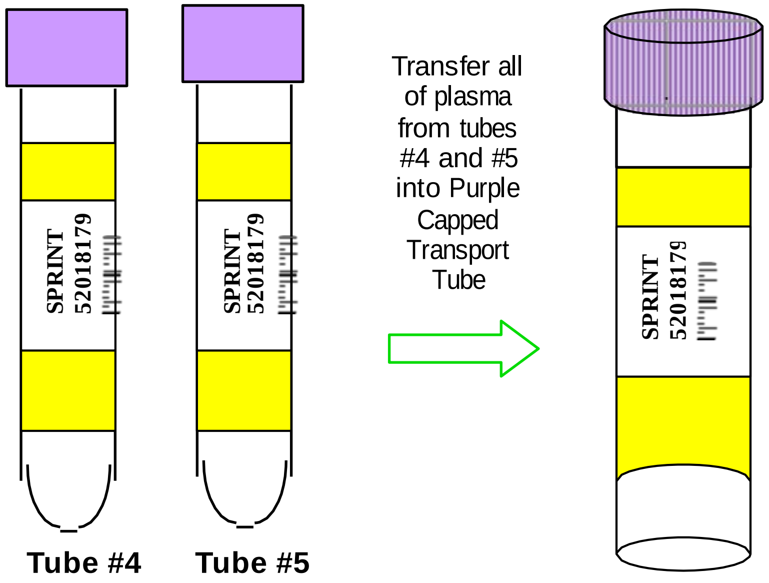

1 MO, 3 MO, 18 MO, 30 MO, 42 MO, 54 MO & PRN VISITS

4.2.b Stage One: Immediate Processing

After completion of venipuncture:

1. Tube #1 remains at room temperature for 20-30 minutes to allow the blood to clot (blood at 4°C clots extremely slowly). Set a timer for 30 minutes as a reminder to centrifuge this tube.

4.2.b Stage Two: Processing of Serum

Stage two begins approximately 30 minutes after venipuncture.

1. As close to 30 minutes after venipuncture as possible, spin the red stoppered tube #1 at 1800-1900 RCF (g) for 10 minutes (if swinging bucket type) at room temp. Increase the time to 15 minutes if using a fixed angle type centrifuge. Consult the centrifuge manual for appropriate RPM setting to achieve 1800-1900 RCF (g).

2. When the centrifuge has come to a complete stop, remove tube #1 and place it in a wire rack. This tube is ready to be prepared for shipping. If packaging for shipment cannot be done immediately, place the tube in the refrigerator until it can be prepared for shipping.

6 MO, 36 MO, AND 60 MO VISITS

4.2.c Stage One: Immediate Processing

After completion of venipuncture:

1. Tube #1 remains at room temperature for 20-30 minutes to allow the blood to clot (blood at 4°C clots extremely slowly). Set a timer for 30 minutes as a reminder to centrifuge this tube.

4.2.c Stage Two: Processing of Serum

Stage two begins approximately 30 minutes after venipuncture.

1. As close to 30 minutes after venipuncture as possible, spin the red top tube #1 at 1800-1900 RCF (g) for 10 minutes (if swinging bucket type) at room temp. Increase the time to 15 minutes if using a fixed angle type centrifuge. Consult the centrifuge manual for the appropriate RPM setting to achieve 1800-1900 RCF (g).

2. When the centrifuge has come to a complete stop, remove tube #1 and place it in a wire rack. This tube is ready to be prepared for shipping. If packaging for shipment cannot be done immediately, place the tube in the refrigerator until it can be prepared for shipping.

12 MO, 24 MO, AND 48 MO VISITS

4.2.d Stage One: Immediate Processing

After completion of venipuncture:

1. Tubes #1 and #2 remain at room temperature for 20-30 minutes to allow the blood to clot (blood at 4°C clots extremely slowly). Set a timer for 30 minutes as a reminder to centrifuge these tubes.

2. Place tubes, #4 and #5 in the refrigerator for 20-30 minutes. Set a timer for 30 minutes as a reminder to centrifuge these tubes.

3. No tube #3 is collected

4.2.d Stage Two: Processing of Serum and Plasma

Stage two begins approximately 30 minutes after venipuncture.

1. As close to 30 minutes after venipuncture as possible,spin the red stoppered tubes #1 and #2 (blood must be clotted) and the 8.0 mL purple top/gel EDTA tubes #4 and #5 at 1800-1900 RCF (g) for 10 minutes (if swinging bucket type) at room temp. Increase the time to 15 minutes if using a fixed angle type centrifuge. Consult the centrifuge manual for the appropriate RPM setting to achieve 1800-1900 RCF (g).

2. When the centrifuge has come to a complete stop, remove tubes #1 and #2 and place them in a wire rack. These tubes are ready to be prepared for shipping. If

packaging for shipment cannot be done immediately, place the tubes in the refrigerator until they can be prepared for shipping. Tubes #1 and #2 need no further processing before shipping.

3. When the centrifuge has come to a complete stop, remove tubes #4 and #5 and place them in a wire rack. These tubes are ready to be prepared for shipping. **Process tubes #4 and #5 accordingly: Label a purple capped, 10.0 mL Transport Tube with the same Lab ID number that is on tubes #4 and #5. Using a plastic transfer pipette, transfer all of the plasma from tubes #4 and #5 into one purple cap, 10.0 mL Transport Tube before shipping. After the transfer of the plasma is complete, the empty tubes #4 and #5 can be discarded into the biohazard waste. If packaging for shipment cannot be done immediately, place the 10.0 mL Transport Tube containing plasma in the refrigerator until it can be prepared for shipping.**

   Note: *Avoid formation of potentially infectious aerosols when removing the rubber stoppers from Vacutainer tubes. In addition to wearing protective gloves, hold a piece of gauze over the stopper while slowly removing it from the tube. Creation of aerosols can also be diminished by careful pipetting and centrifugation techniques. Further steps to minimize infection risk while processing samples are described in the OSHA regulations stated in the Federal Register of December 6, 1991 (Vol. 56, No. 235, page 64177). Wear a mask in combination with an eye protection device, such as goggles or glasses with solid side shields or a chin-length face shield when working with potentially infectious materials that have the potential for splashing, spraying, or spattering. An alternative to these devices would be a desk-mounted or under-shelf-mounted clear plastic shield, which would offer similar protection from possible infectious splashes or sprays.*

Plasma Transfer Process

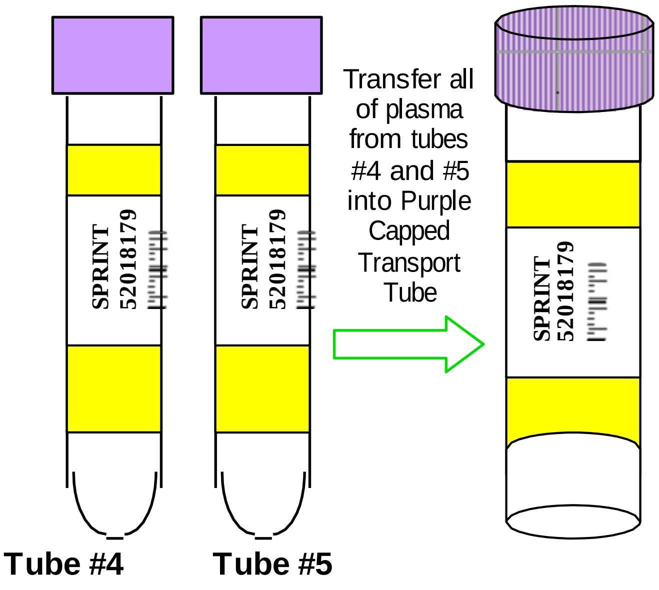

4.3 Urine Collection

RANDOMIZATION, 6, 12, 24, 36, 48, AND 60 MONTH VISITS

A random urine sample is collected from each participant (preferably) at the beginning of the clinical exam at randomization, 6 Mo, 12 Mo, 24 Mo, 36 Mo, 48 Mo, and 60 Mo visits. A specimen cup (labeled with the participant's Lab ID) and cup lid are provided by the staff member working with the participant at that time. The participant is instructed to:

1. void in the cup, filling it if possible, and place the lid securely on top of the container
2. record the time of voiding on the label, and
3. bring the specimen cup back to the staff member, OR
4. place the sample container in a refrigerator designated for urine samples, and report to a staff member that the specimen has been collected, depending on locally approved OSHA regulations.

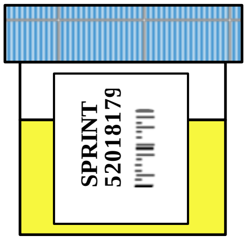

Bathrooms are equipped with a wall clock and pencils for participants to use in recording the time of voiding on the label. The staff member verifies the participant has written the "time voided" on the label, and assesses the adequacy of the sample for processing. At least 10 mL of urine is required for processing. If insufficient, the participant is requested to void again in a clean container prior to leaving the clinical site.

Prior to processing, the laboratory staff records whether a urine sample was obtained and transcribes the collection time of the urine void from the ID label onto each participant's Laboratory Specimen Collection Form.

Labeled urine samples should be placed in the designated specimen refrigerator for storage prior to processing and as soon as possible after the specimen has been voided. This can be done either by the participant or a staff member, as determined by local option. However, procedures need to be set up at each field center to verify that urine samples are not inadvertently left out at room temperature. Urine may be left at room temperature for a maximum of 4 hours. Refrigerated urine samples need to be processed and shipped the same day they are collected.

4.3.1 Urine Processing

The technician prepares the work area by laying out a plastic transfer pipet and one urine transport vial in a rack. A Lab ID label that matches the Lab ID label on the urine cup is affixed to the urine transport vial. Place the label on the tube with the bar-code oriented from the bottom of the transport tube to the top of the transport tube.

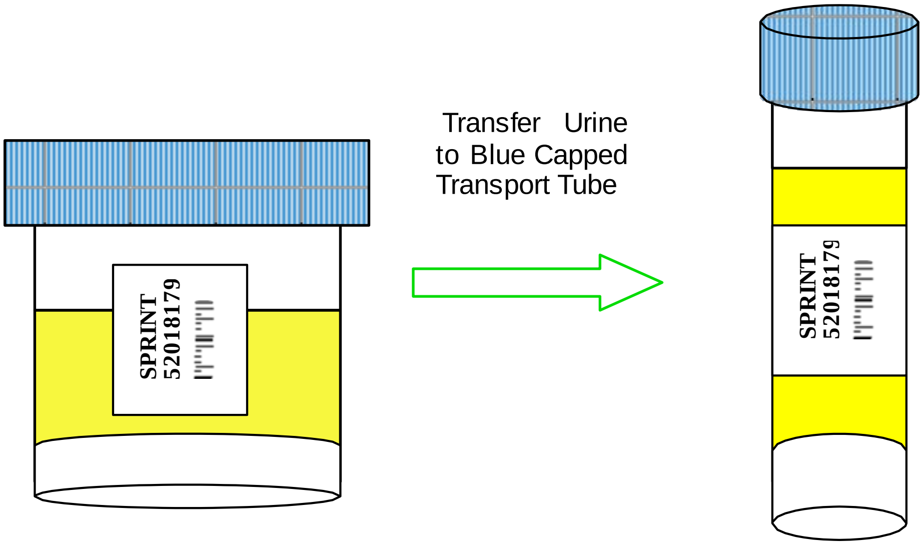

Eye protection, gloves and lab coat must be used for all urine processing. All other rules regarding the safe blood specimen handling must be observed when processing urines.

1. Mix the urine container by inverting eight times.

2. Record the date and time of collection and the processing technician's initials on the Laboratory Specimen Collection Form.

3. Using the plastic transfer pipette, aliquot 10 mL of the urine into the blue capped transport tube. The urine transport vial is now ready for shipping. If packaging for shipment cannot be done immediately, place the urine transport vial in the refrigerator until the shipment can be prepared.

4.3.2 Procedures for Small Urine Samples

If the volume of urine sample is inadequate to process, check to see if a second sample can be obtained. If there is a second sample and it (in and of itself) is adequate for processing, use the second sample (record the time voided on the Laboratory Specimen Collection Form based on that sample) and discard the first sample. If neither is adequate, combine the specimens, and transcribe the latest voiding time on the Laboratory Specimen Collection Form.

4.3.3 Procedures for Urine Samples Contaminated with Blood

Although urine samples contaminated with blood will affect the measurement of albumin, these specimens should not be thrown out. All urine samples collected from participants that have adequate volume for processing are kept, including those that are (appear to be) contaminated with blood. If a urine sample is contaminated with blood, ask the participant to provide a second urine sample at the end of the examination. Use the second sample if it has adequate volume and is less contaminated. Document urine blood contamination by entering the comment, "sample contaminated with blood" in the Blood Collection/Processing Comments section of the Laboratory Specimen Collection form.

4.4 Overview of Specimen Collection

A summary overview of the protocol steps for the collection and processing of blood and urine specimens is presented below in Charts 1-4. (Collection/ Processing Flow Charts)

RANDOMIZATION VISIT (CHART 1) *Centrifuge Times: Swinging Bucket type is 10 minutes or Fixed Angle type is 15 minutes

Test	Tube Name	Fasting Condition	Blood Collection Tube	Visual Reference	Collection/Processing
Chemistry Profile<br><br>Lipid Profile<br><br>Glucose	**Red Top Tube #1**	Fasting	7.5 mL red-top w/gel separator	Label Tube 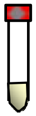	• Fill labeled tube completely with blood<br>• Invert tube 8 times<br>• Tube must sit 20-30 min at **Room Temp**<br>• Blood will clot<br>• Centrifuge (tubes #1, #2, #4, #5 at same time)<br>• Refrigerate until Shipment<br>• Ship to Lab Same Day
Serum Storage	**Red Top Tube #2**	Fasting	7.5 mL red-top w/gel separator	Label Tube 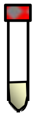	• Fill labeled tube completely with blood<br>• Invert tube 8 times<br>• Tube must sit for 20-30 min at **Room Temp**<br>• Blood will clot<br>• Centrifuge (tubes #1, #2, #4, #5 at same time)<br>• Refrigerate until Shipment<br>• Ship to Lab Same Day
Genomic Sample	**Purple Top Tube #3**	Fasting	10.0 mL purple top	Label Tube 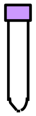	• Fill labeled tube completely with blood<br>• Invert tube 8 times.<br>• **DO NOT** Centrifuge<br>• Place tube in Refrigerator until Shipment<br>• Ship to Lab Same Day
Plasma Storage (1)	**Purple Top Tube #4**	Fasting	8.0 mL purple-top w/gel separator	Label Tube 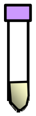	• Fill labeled tube completely with blood<br>• Invert tube 8 times/**Refrigerate** for 20-30 min<br>• Centrifuge (tubes #1, #2, #4, #5 at same time)<br>• Transfer plasma from tubes #4 & #5 into labeled Transport tube<br>• Refrigerate until Shipment; ***Pl. Transport Tube has purple cap***<br>• Ship to Lab Same Day
Plasma Storage (2)	**Purple Top Tube #5**	Fasting	8.0 mL purple-top w/gel separator	Label Tube 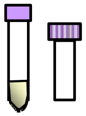	• Fill labeled tube completely with blood<br>• Invert tube 8 times/**Refrigerate** for 20-30 min<br>• Centrifuge (tubes #1, #2, #4, #5 at same time)<br>• Transfer plasma from tubes #4 & #5 into labeled Transport tube<br>• Refrigerate until Shipment; ***Pl. Transport Tube has purple cap***<br>• Ship to Lab Same Day
Urine Albumin, Creatinine, Storage	**Urine Cup & Urine Transport Tube**	Non-fasting acceptable	Urine Collection Cup & 10 mL poly-transfer tube	Label Urine 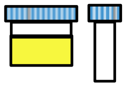	• Refrigerate Urine sample<br>• Mix urine 8 times<br>• Transfer 10 mL of Urine to labeled Transport tube<br>• Refrigerate until Shipment; ***Urine Transport tube has blue cap***<br>• Ship to Lab Same Day

.

1 MO, 3 MO, 18 MO, 30 MO, 42 MO, AND 54 MO VISITS & PRN VISITS (CHART 2)

**Serum tests vary by specific visit: 1MO, 3MO, 18MO, 30MO, 42MO, and 54MO visits all have a chem. profile; PRN battery includes: Sodium, Potassium, Creatinine, BUN*

Test	Tube Name	Fasting Condition	Blood Collection Tube	Visual Reference	Collection/Processing
Chemistry Profile<br><br>*PRN battery only includes: Na, K, Creat, and Bun	**Red Top Tube #1**	Non-fasting acceptable	7.5 mL red-top w/gel separator	Label Tube 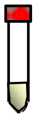	• Fill labeled tube completely with blood.<br>• Invert tube 8 times.<br>• Tube must sit 20-30 min at **Room Temp**<br>• Blood will clot<br>• Centrifuge<br>• Refrigerate until Shipment<br>• Ship to Lab Same Day

*Centrifuge Times: Swinging Bucket type is 10 minutes or Fixed Angle type is 15 minutes

6 MO, 36 MO, AND 60 MO VISITS (CHART 3)

Test	Tube Name	Fasting Condition	Blood Collection Tube	Visual Reference	Collection/Processing
Chemistry Profile	**Red Top Tube #1**	Non-fasting acceptable	7.5 mL red-top w/gel separator	Label Tube 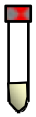	• Fill labeled tube completely with blood<br>• Invert tube 8 times<br>• Tube must sit 20-30 min at **Room Temp**<br>• Blood will clot<br>• Centrifuge<br>• Refrigerate until Shipment<br>• Ship to Lab Same Day
Urine Albumin, Creatinine	**Urine Cup & Urine Transport Tube**	Non-fasting acceptable	Urine Collection Cup & 10 mL poly-transfer tube	Label Urine 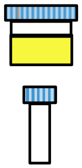	• Refrigerate Urine sample<br>• Mix urine 8 times<br>• Transfer 10 mL of Urine to labeled Transport Tube<br>• Refrigerate until Shipment<br>• Ship to Lab Same Day

*Centrifuge Times: Swinging Bucket type is 10 minutes or Fixed Angle type is 15 minutes

12 MO, 24 MO, AND 48 MO VISIT (CHART 4) *Centrifuge Times: Swinging Bucket type is 10 min. or Fixed Angle type is 15 minutes

**Serum tests vary by specific visit: 12MO includes a chem. profile and lipids; 24MO includes a chem. profile, glucose, and lipids; 48MO includes a chem. profile and glucose*

Test	Tube Name	Fasting Condition	Blood Collection Tube	Visual Reference	Collection/Processing
Chemistry Profile<br>Lipid Profile<br>Glucose	**Red Top Tube #1**	Fasting	7.5 mL red-top w/gel separator	Label Tube 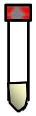	• Fill labeled tube completely with blood<br>• Invert tube 8 times<br>• Tube must sit 20-30 min at **Room Temp**<br>• Blood will clot<br>• Centrifuge (tubes #1, #2, #4, #5 at same time)<br>• Refrigerate until Shipment<br>• Ship to Lab Same Day
Serum Storage	**Red Top Tube #2**	Fasting	7.5 mL red-top w/gel separator	Label Tube 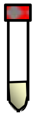	• Fill labeled tube completely with blood<br>• Invert tube 8 times<br>• Tube must sit for 20-30 min at **Room Temp**<br>• Blood will clot<br>• Centrifuge (tubes #1, #2, #4, #5 at same time)<br>• Refrigerate until Shipment<br>• Ship to Lab Same Day
No Tube #3					
Plasma Storage (1)	**Purple Top Tube #4**	Fasting	8.0 mL purple-top w/gel separator	Label Tube 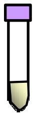	• Fill labeled tube completely with blood<br>• Invert tube 8 times/**Refrigerate** for 20-30 min<br>• Centrifuge (tubes #1, #2, #4, #5 at same time)<br>• Transfer plasma from tubes #4 & #5 into labeled Transport tube<br>• Refrigerate until Shipment; ***Pl. Transport Tube has purple cap***<br>• Ship to Lab Same Day
Plasma Storage (2)	**Purple Top Tube #5**	Fasting	8.0 mL purple-top w/gel separator	Label Tube 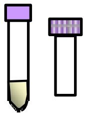	• Fill labeled tube completely with blood<br>• Invert tube 8 times/**Refrigerate** for 20-30 min<br>• Centrifuge (tubes #1, #2, #4, #5 at same time)<br>• Transfer plasma from tubes #4 & #5 into labeled Transport tube<br>• Refrigerate until Shipment; ***Pl. Transport Tube has purple cap***<br>• Ship to Lab Same Day
Urine Albumin, Creatinine, Storage	**Urine Cup & Urine Transport Tube**	Non-fasting acceptable	Urine Collection Cup & 10 mL poly-transfer tube	Label Urine 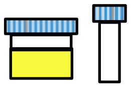	• Refrigerate Urine sample<br>• Mix urine 8 times<br>• Transfer 10 mL of Urine to labeled Transport tube<br>• Refrigerate until Shipment; ***Urine Transport Tube has blue cap***<br>• Ship to Lab Same Day

PACKAGING AND SHIPPING

Samples are shipped to the Lab the same day of collection.
Packaging and shipping instructions for refrigerated daily shipments (See Figure 1-4, pg.28-31)

5.1. Packaging and Shipping Instructions

1. Check to be sure that each tube is properly labeled. Wrap tube(s) in paper toweling to cushion it (them) and place in a 5" x 8" storage bag. (One patient's tubes per 5" x 8" bag). *It is acceptable to place more than one participant's samples in a shipping box for specified visits (see shipping charts)*. Include an absorbent square in each bag.

2. Place bag into a small Styrofoam cooler with a frozen gel pack (frozen at -20° C in the Styrofoam cooler) to keep the samples cold (not frozen) during shipment. Place bag with sample(s) on top of frozen gel pack. Place the Styrofoam cooler into its cardboard sleeve. **Note: In order to ensure that the gel pack and tubes will fit inside the Styrofoam container, freeze the 8 oz gel pack 24 hours ahead of time inside of the Styrofoam container. Do NOT freeze the outer cardboard sleeve of the shipping container.**

3. Shipping boxes received from the Central Laboratory will have a "Biological Substance Category B UN 3373" label affixed to outside of the box. If the shipping box does not have this label on the outside, contact the Central Laboratory.

4. Place shipping box in orange, plastic "FedEx UN 3773 Pak" mailing bag. Note: Two shipping boxes may be placed inside the mailing bag if shipping more than one participant in a day.

5. Insert the original Laboratory Specimen Collection Form for the participant into a 9" x 12" plastic bag and place it inside the orange, plastic "FedEx UN 3773 Pak" mailing bag. (Keep a copy of the Laboratory Specimen Collection Form for your files.)

6. The Central Laboratory will supply the SPRINT clinical sites with pre-printed FedEx billable stamps. *Puerto Rico clinical sites can NOT use FedEx billable stamps. Puerto Rico Clinical Sites will have to use International Air Waybills and Commercial Invoice forms. The phone number for FedEx International shipping questions is 1-800-247-4747.

7. Record the clinical site number, address, and telephone number in section 1 of the FedEx Billable Stamp. Peel off the right side of the FedEx Billable Stamp and affix it to the outside of the orange, plastic "FedEx Clinical Pack" mailing bag. The left side of the form may be kept for your records. Contact Federal Express (1-800-GO-FEDEX) for pickup the same day that the samples are collected. Some clinical sites may have a scheduled FedEx pick up; this will vary from site to site. The packages may also be mailed at FedEx drop-off locations that will accept UN 3373 Pak shipments.

8. If a clinical site runs out of FedEx pre-printed billable stamps and cannot get them in time from the Central Lab for a specimen shipment, then a regular FedEx Airbill can be used. Choose **"Priority Overnight"** as the delivery service. Do **NOT** use "First Overnight" delivery service as this option is four times more expensive and is not

needed. “Priority Overnight” delivery service will ensure that the package is delivered by 9:00-10:00 am the next morning.

9. It is the clinical site’s responsibility to ensure that the package is picked up by FedEx and delivered to the Central Laboratory. Follow these steps to track your package: Go to the FedEx website www.fedex.com/us/, click on <Track> drop-down menu, click on <Track by Tracking Number>, enter tracking number, and click on <Track>. The tracking information will be displayed on the <Summary> screen. If the <Summary Results> state “Not Found”; this means your package has not been picked up and FedEx should be contacted. Check to see if your package has been delivered to the Central Laboratory the morning following shipment using the same tracking procedure.

10. The Central Laboratory will check the “FedEx Insight Tracking Log” daily to view what SPRINT packages should be arriving. However, only those packages actually picked up and scanned into the FedEx system will appear on this log.

11. See Figure 1-4 below for specific visit shipping diagrams.

Note: All shipping containers are sent to the SPRINT Central Laboratory by overnight courier to ensure receipt within 24 hours. The empty Styrofoam containers are recycled by returning them to the Clinical Centers via FedEx Express Service. Shipping containers to the Central Laboratory are addressed as follows:

SPRINT Central Laboratory
University of Minnesota, Advanced Research and Diagnostic Laboratory (ARDL)
1200 Washington Ave S., Suite 175
Minneapolis, MN 55415
Telephone: (612) 625-5040
Main Fax: (612) 625-4142
Alternate Fax: (612) 625-4831

SPRINT Central Laboratory Hours: Phone # 612-625-5040

Monday-Friday	7:00 am-3:30pm
Saturdays	Open; hours vary
Sundays	Closed

*A holiday schedule will be posted on the SPRINT website.

RANDOMIZATION VISIT

Figure 1. Refrigerated Sample Packaging/Only One Participant's Samples Per Bag and Shipping Box

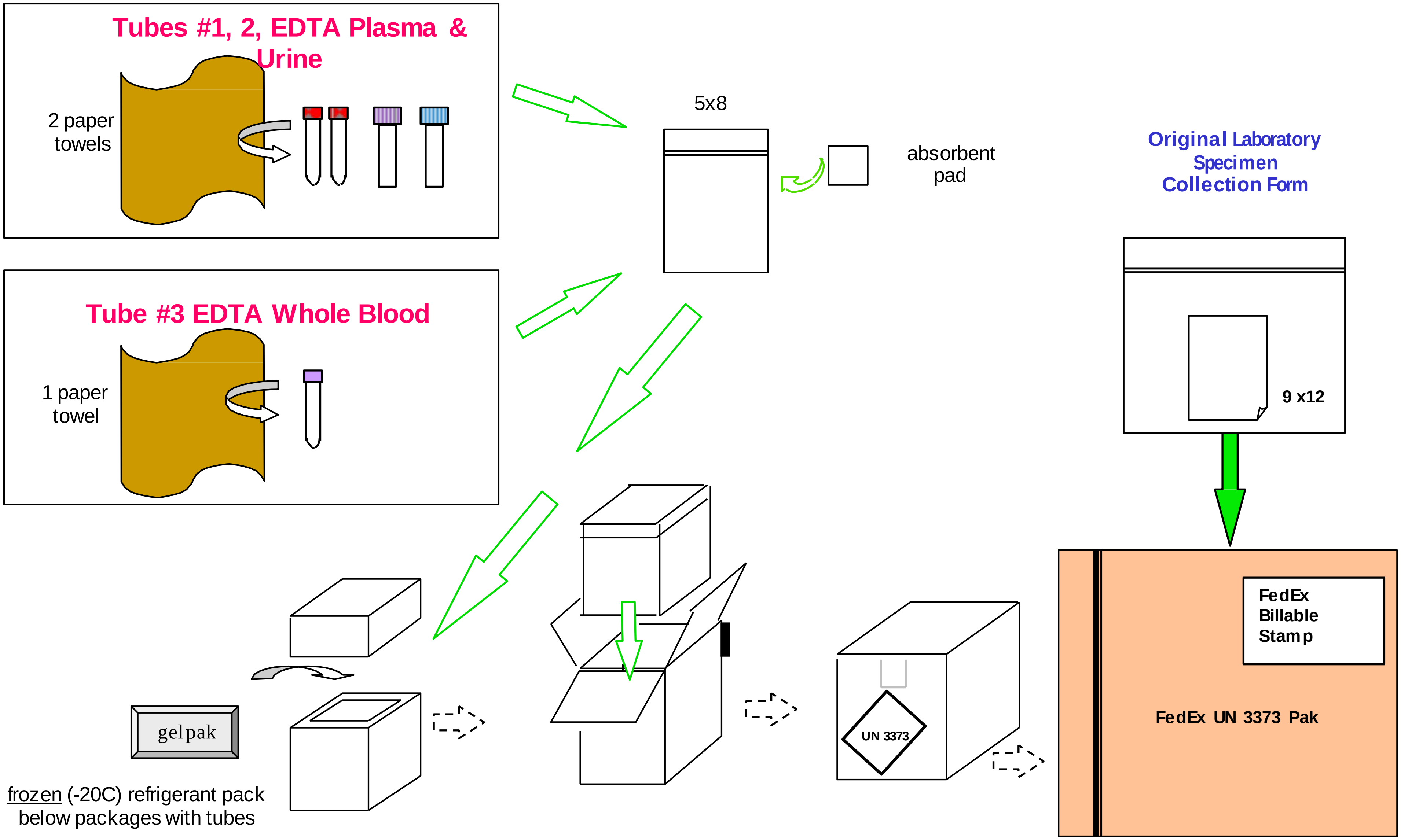

1 MO, 3 MO, 18 MO, 30 MO, 42 MO, AND 54 MO VISIT & PRN VISITS

Figure 2. Refrigerated Sample Packaging/ One Participant's Samples Per Bag/Up to Three Participants in Shipping Box is Acceptable

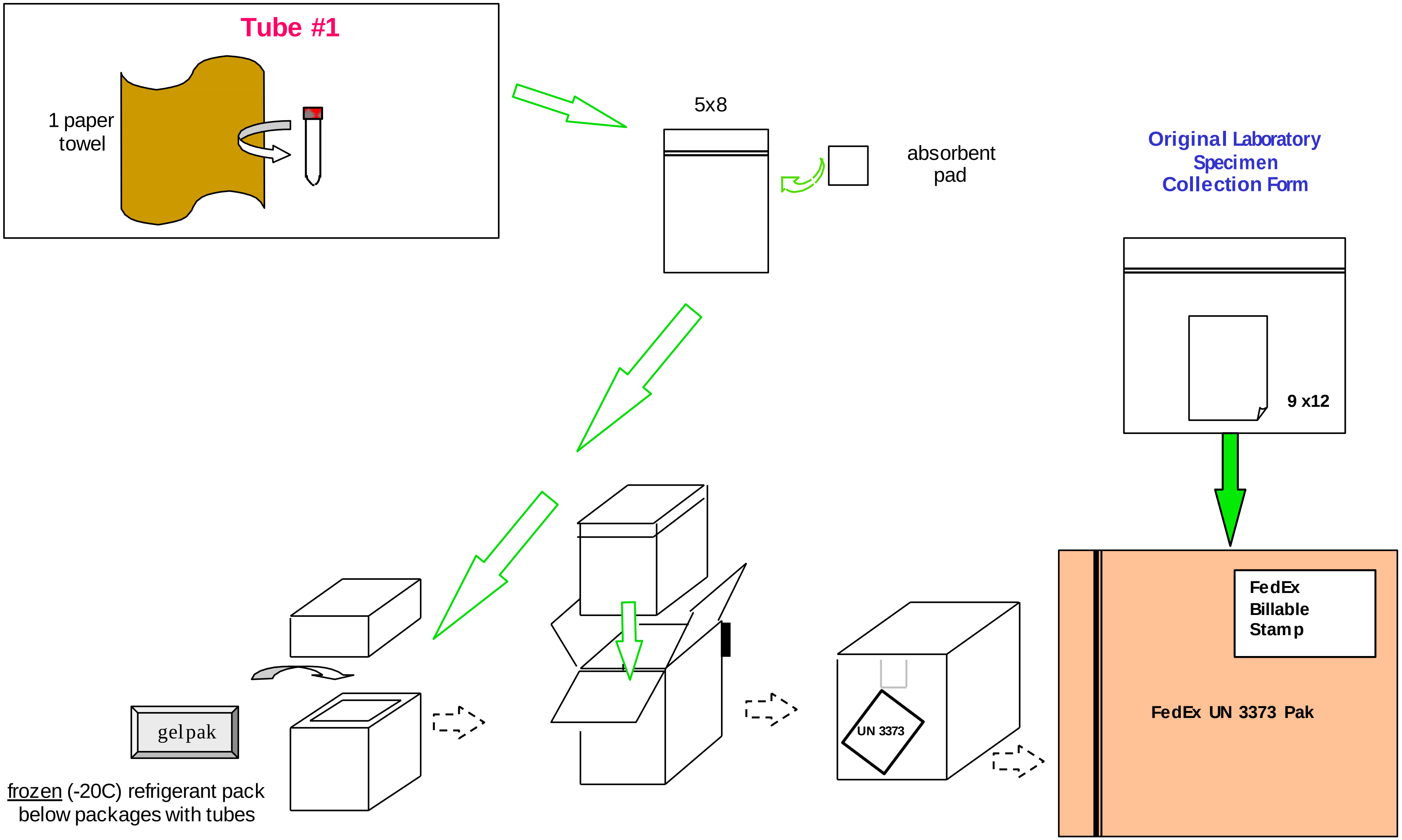

Figure 3. Refrigerated Sample Packaging/One Participant's Samples Per Bag/Up to Two Participants in Shipping Box is Acceptable

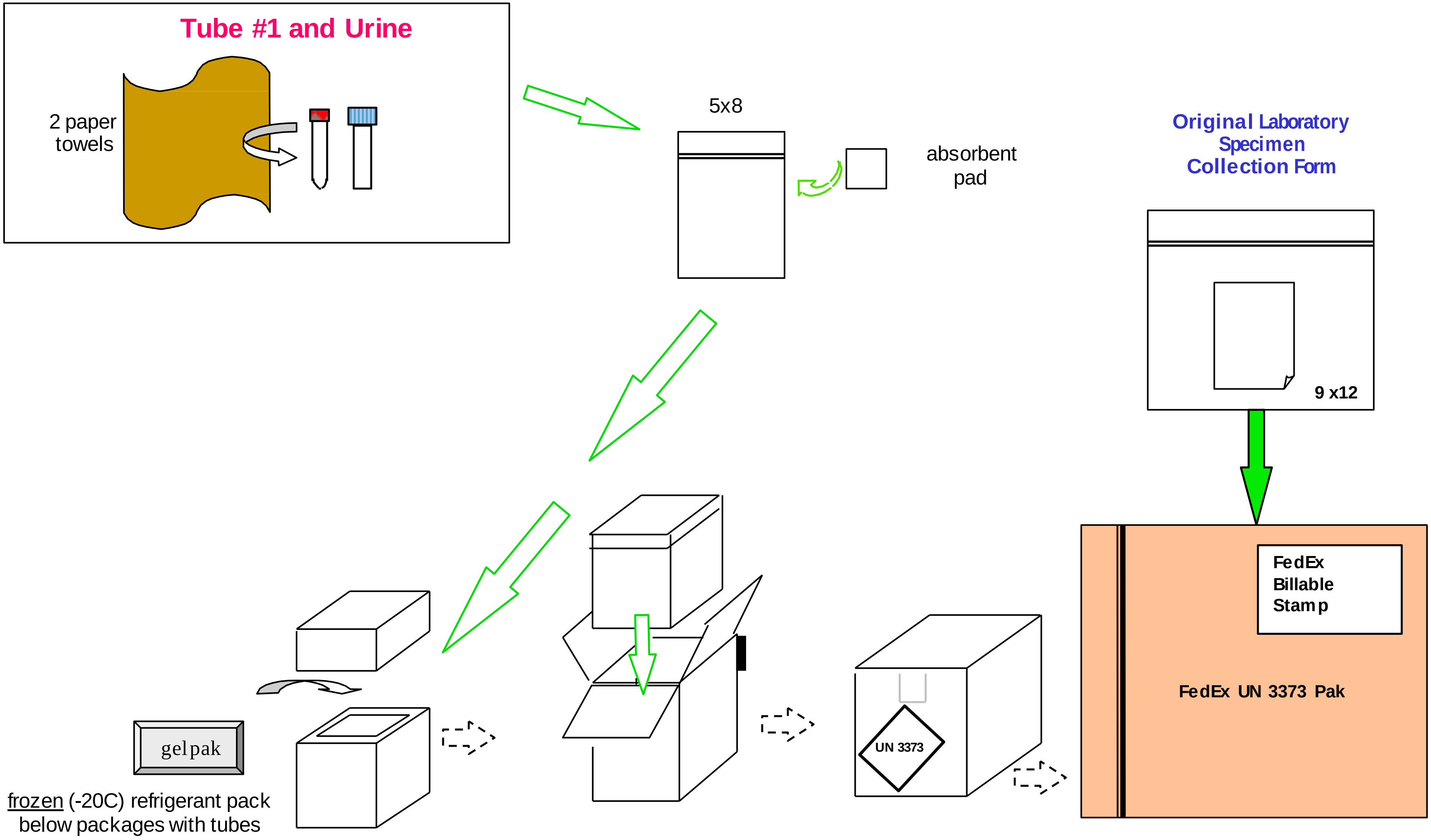

12 MO, 24 MO, AND 48 MO VISIT

Figure 4. Refrigerated Sample Packaging/Only One Participant's Samples Per Bag and Shipping Box

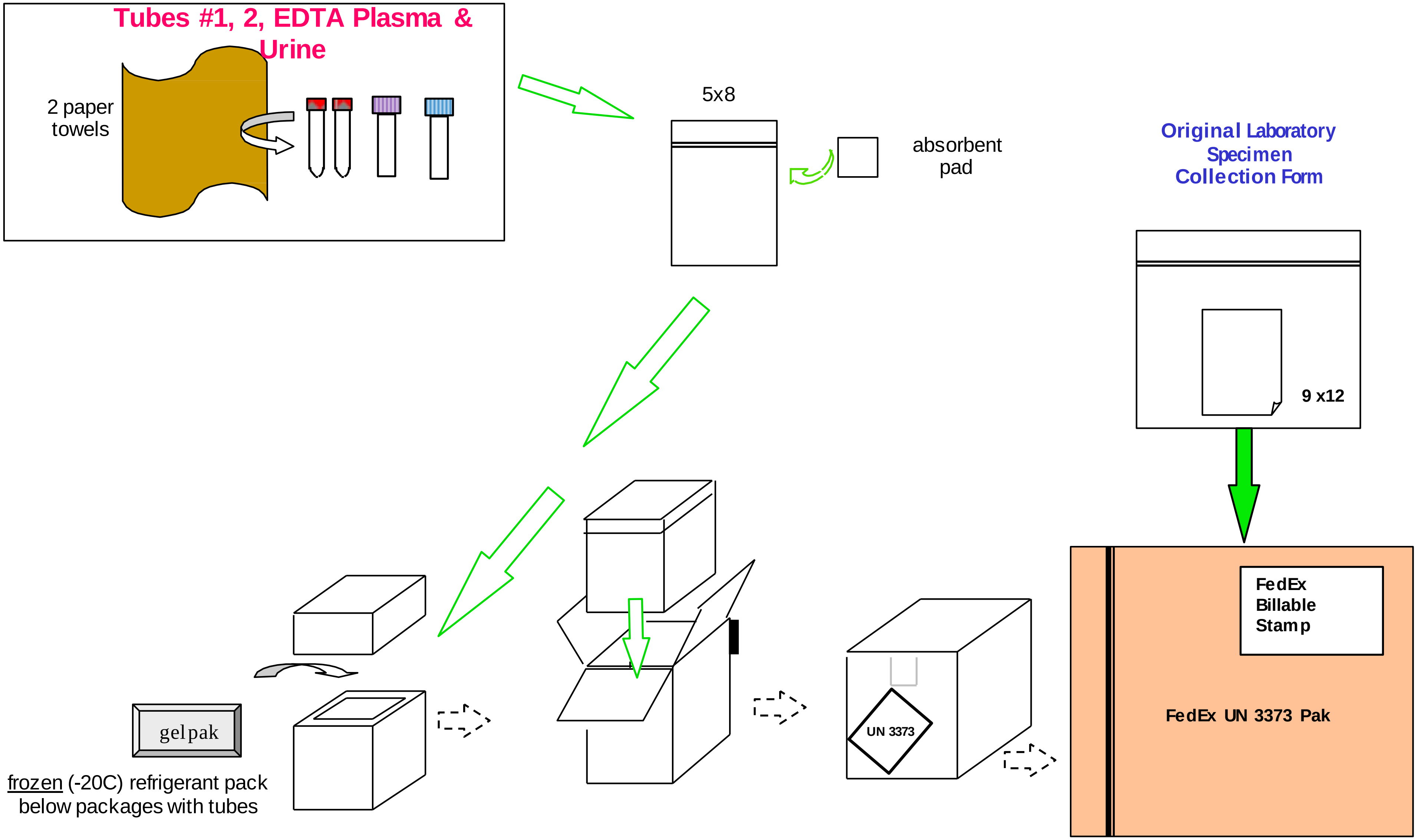

6. QUALITY CONTROL

There are two different aspects of quality control. One is the daily or monthly record of the performance of the refrigeration and freezer equipment. Daily and monthly measurements (e.g., temperatures) are recorded on a log, as described below. The other aspect of quality control is documentation of problems with blood collection and processing which is part of each participant's record. Blood collection/Processing problems can be recorded in the "Blood Collection/Processing Comments" section of the Laboratory Specimen Collection Form.

- all or some blood samples not drawn
- tourniquet reapplied
- fist clenching
- needle movement
- incomplete blood collection causing missing tubes
- broken tubes
- clotted tubes
- hemolyzed serum or plasma
- lipemic serum or plasma
- other processing problems

This record provides documentation that blood was drawn in a standardized manner and that the equipment was functioning properly. This quality control documentation is the best evidence that samples in each of the clinical sites are being drawn and processed identically. Differences in the way the samples are collected or processed could potentially create a significant difference in assay results, which could seriously compromise the laboratory test data. It is very important that the quality control records of the procedures and the equipment be properly maintained.

Daily, log the temperatures of the laboratory, all refrigerators and freezers. (Appendix 3) In addition, check and record the actual speed of the centrifuge annually with a tachometer. (This is usually performed by a biomedical engineer.)

6.1. Quality Control Clinic Performance

As part of the overall quality control program for Clinic Performance , the Central Laboratory will use the Biological Specimen Inventory system (BSI) to track clinic performance issues such as shipment delay, sample collection problems (inadequate volume, hemolysis etc…), and processing issues. The Central Laboratory will prepare a Clinic Performance Summary Report quarterly to be reviewed by the SPRINT MPQC committee. The Clinic Performance Summary Report will be used to identify specific clinical sites that are having problems with the Laboratory protocol.

6.2. Internal Laboratory Control

Internal quality control procedures monitor analytical performance of the test relative to medical goals and alert analysts to unsatisfactory analytical performance. Quality control statistics are used to make judgments about the quality of analytical results, whether system correction is necessary, whether participant data should be accepted or rejected, and for estimating performance parameters which can be compared to analytical and medical goals. Testing is monitored by two control samples analyzed daily in each batch of samples. A permanent standard deviation (SD) and coefficient of variation (CV) is determined by analyzing the material on 50 – 100 separate days. The mean for new lots of material is established by analyzing the

material on 20 separate days. The SD and CV from the data collected over 20 days is used to monitor the permanently established SD. Quality control results are plotted on Levy-Jennings plots and acceptability (i.e. in statistical control) is determined using three Westgard rules (1-2s, 1-3s, and 2-2s). Documentation is made on the control charts when there is a change in reagent lot numbers, any action is taken due to unacceptable control results, and when other pertinent information is observed.

In addition, the Central laboratory will monitor SPRINT assays for long term drift by plotting monthly control means on Levy-Jennings plots. The Central Laboratory will prepare a Long Term Drift quarterly report to be reviewed by the SPRINT MPQC committee. Identified trends will be further investigated by the Central Laboratory.

6.3. Reporting Results

The Central Laboratory has the responsibility for reporting results to the Coordinating Center. All test results are transmitted to the Coordinating Center via a Web File Upload. This transmission will occur daily, Monday through Friday. In addition, all laboratory results will be auto faxed to the Clinical Centers. The auto faxed result reports will print between 2:00-3:00 am daily. Clinical Sites must leave their fax machines on at all times to ensure the timely receipt of result reports. If a fax cannot get through, the auto-fax system will re-dial 3-4 times within an hour's time and then it will cease further attempts. If there is a transmission problem during the auto-fax process, the Central Laboratory should be notified (612-273-3645) and our LIS department will manually resend the reports. The Central Laboratory has no way of knowing whether a clinical site receives its faxes so we are dependent on the sites contacting us when they do not receive their lab reports. All alert values will be called to a specified coordinator at each Clinical Site. It will be the responsibility of the Clinical Site Coordinator to notify the participant of their alert value results and arrange for appropriate medical care. Any test results which are out of the reference range or are an alert value will be flagged with stars on the laboratory report. Reference ranges and alert values can be found in Appendix 1.

7. TRAINING PROCEDURES

Blood drawing and processing are performed by a certified SPRINT technician(s) at each clinical site. The technicians complete a central training course taught by certified laboratory staff. Each technician must complete the training before becoming SPRINT certified. New staff will be trained by clinical site supervisory personnel. Recertification takes place annually and is authorized by the clinical site supervisory personnel.

Technicians will be trained in actual procedure of phlebotomy by their respective institutions. The study does not provide phlebotomy training.

An informative phlebotomy website is http://phlebotomy.com/Links.htm.

A check list of the venipuncture and processing procedures that the SPRINT technicians must know and be prepared to demonstrate is listed in Appendix 4 and 5. The technician must study the SPRINT Biospecimen Collection and Processing Manual and watch a few participant samples being processed. Then the technician may proceed to a mock drawing and mock processing of samples, without performing any actual venipuncture. Mock venipuncture is performed with the Vacutainer system. A piece of latex tubing with a knot in one end leading to a glass of water is used as a target vein. Practice tubes are collected in the correct order, and then placed at their proper positions. The sample is processed from start to finish exactly as if

real blood were being used. Each technician performs a minimum of two mock draws from beginning to end. Although the mock draws take time, they provide hands-on experience and allow the technician to become comfortable with the procedures before proceeding to live participants.

At this point the technicians are ready to practice on live volunteers. The technicians practice at least once with just one volunteer at a time and again process the blood entirely by themselves from start to finish. If the technicians do not feel comfortable, they can always go back and repeat the process with dummy tubes. If volunteers are available, it may be beneficial to repeat this several times. Any questions or problems that the technicians have must be solved before the technicians actually proceed to drawing the SPRINT participants. Before the technicians draw blood from any SPRINT participants, they must be certified by clinical site supervisory personnel.

8. SPECIMEN STORAGE PLAN

Specimens will be stored frozen at -70°C at the Central Laboratory Repository at University of Minnesota Medical Center, Fairview. All samples will be stripped of identifiable information prior to storage, i.e. samples will be labeled at the Clinical Centers with a laboratory number only, which will not permit the laboratory repository to identify a sample with an individual person. Aliquots from the same participant will be stored in at least two different -70°C freezers. The -70°C freezers are equipped with emergency power, an opening mechanism that is lockable, audible and visual alarms, and an alarm test button. These freezers are either monitored 24 hours/day, 7 days/week or maintained on an automatic alarm system that will transmit a signal to a computerized telephone alarm system. All freezers are kept locked, and the key is stored in an area accessible to authorized personnel only. In addition, the freezer room itself is kept locked, with the lock code available to authorized personnel only.

A computerized inventory system (Biological Specimen Inventory, BSI, by Information Management Services, Inc.) is in place for tracking all specimens. The BSI system is a secured system which can be accessed only by laboratory personnel having a secured user profile and password. The BSI system is backed up by a secured redundant server.

During the SPRINT study, your samples will be kept by the SPRINT Central Laboratory. At the conclusion of the SPRINT study, all samples will be moved to a long term storage facility called a biorepository that will be maintained by the National Institutes of Health or one of its agencies or subsidiaries.

Appendix 1. SPRINT Laboratory Tests, Reference Ranges, and Alert Values

Test Name	Reference Range	Units	Alert Value
Sodium (Na)	133-145	mmol/L	<133 and >150
Potassium (K)	3.3-5.1	mmol/L	<3.0 and >5.5
Chloride (Cl)	96-108	mmol/L	
Bicarbonate (CO2)	22-29	mmol/L	
Urea Nitrogen (BUN)	6-23	mg/dL	
Creatinine	0.40-1.20	mg/dL	Result increase by 50% to >=1.5
Glucose	60-99	mg/dL	<60 and >200
Cholesterol	0-200	mg/dL	
Triglycerides	<150	mg/dL	
HDL-Cholesterol	Male >40	mg/dL	
	Female >50	mg/dL	
LDL-Cholesterol, calculated	0-129	mg/dL	
Urine Creatinine	Male 40-278	mg/dL	
	Female 29-226	mg/dL	
Urine Albumin (Microalbumin)		mg/L	
Urine Albumin/Creatinine ratio (ACR)	<30	mg/g Cr	

Appendix 2. Equipment and Supplies

Supplies to be provided by the Central Laboratory as a Kit to the Clinical Sites and used by the Central Laboratory for processing SPRINT samples upon receipt:

***Note: Supplies must be re-ordered by clinical sites using the Supply Reorder form (Appendix 6) and take 7-10 business days to receive**

*Note: Some of these supplies will be used only by the Central Laboratory for processing and will not be included in the kits.

Supplier	Catalogue No	Description
Cardinal Health	C1300-82	Microvials, clear (2 mL) 500/pk
Cardinal Health	C1310-15	Red Screw Caps 1000/pk
Cardinal Health	C1310-20	Purple Screw Caps 1000/pk
Cardinal Health	C1310-12	Clear Screw Caps 1000/pk
Cardinal Health	C1310-14	Yellow Screw Caps 1000/pk
Cardinal Health	C1310-18	Orange Screw Caps 1000/pk
Cardinal Health	C1310-17	Green Screw Caps 1000/pk
Cardinal Health	T1234-3	Screw Top Vials/w/caps (5 mL) 1000/pk
Cardinal Health	T1233-5 (581-8)	10 mL Transport Tube, 1000/cs
UStores	268395	Vacutainer Tube; Serum SST double gel(7.5 mL), red top,100/pk
Cardinal Health	B2970-48	Vacutainer Tube; EDTA (10 mL), purple top, 100/pk
Cardinal Health	GR-455040B	Vacutainer Tube; EDTA-gel (8 mL), purple top, 50/pk
Cardinal Health	P5214-12	Plastic Disposable Transfer Pipettes, 500/bx
Cardinal health	C8827-4	Sterile Urine Container w/white screw cap, 100/cs
Fischer Scientific	SA56-500	6.0 N HCL, 500 mL , acid urine
Fischer Scientific	SS148-1	1.0 N sodium carbonate, 1L, alkaline urine
Coridian Tech, Inc.	CPN-3449	Bar-coded Lab ID Labels
UStores	CX10017	Ziplock Freezer Bags 9” x 12”, 100/pk
UStores	CX10019	Ziplock Freezer Bags 5” x 8”, 100/pk
Cardinal Health	M1050-7	50 mL Absorbent Pads for shipping, 100/pk
UStores	035286	UTEK Gel Packs for shipping samples, 8 oz, 36/cs
UStores	GC21250	Paper Towels, 4000/cs
UStores	0352831	Thermosafe 601 Styro Specimen Mailer (6 x 3 x 2.5 in.), 50/cs
UStores	035256	Thermosafe 602 Cardboard Sleeve for Specimen Mailer, 50/cs
ULINE	GS90325	18 x 12 x 12 Box (ship out kits),15/bu
FedEx		FedEx Mailing Bags (UN 3373 Paks)
FedEx		FedEx pre-printed Billable Stamps
FedEx		UN 3373 Labels
FedEx		International Air Waybills & Commercial Invoice forms
		Colored Dot Stickers

Supplies to be obtained by the CCN/Clinical Sites:

Supplier	Catalogue No	Description
Cardinal Health	B3036-14	Butterfly Needles, 21G x 3/4", #367250
Cardinal Health	B3035-12	Luer Adapters, #367290
Cardinal Health	364815	Vacutainer Tube Holders 1000/cs BD #364815
Cardinal Health	40000-110	Alcohol Swabs 2,000/cs
Cardinal Health	KC913A	Gauze Sponges 200/pk
Cardinal Health	JJ5644	Band Aids 100/pk
Cardinal Health	367203	Tourniquets, Latex free, 50/pk
		Wire Tube Rack
		Disposable Gloves (powder-free)
		Lab Coats
		Goggles or Face Shield
		Bleach decontaminant: 1 part bleach to 9 parts water
		Biohazzard waste containers
		Sharps/Biohazzard waste containers
		Sharpie Fine Point Pens
Cardinal Health	B3062-40	PDI Ammonia Inhalant
Cardinal Health	B2922-1A	Blood Collection Trays (optional)
Cardinal Health	T2941-3 or T2960-4	Thermometers -20 C-+70 C
Cardinal Health	C6510-1	Timer- 3 channel digital

Equipment purchased and maintained by CCN/Clinical Sites and Staff Requirements:

Centrifuge: Swinging bucket type preferred but a fixed angle type will work also. A refrigerated or non-refrigerated centrifuge is acceptable. Samples can be spun down at room temperature but should be placed immediately into a refrigerator after centrifugation until shipment. Leaving tubes in a non-refrigerated centrifuge or at room temperature will compromise the accuracy of testing. Centrifuge should be capable of a speed of 1800-1900 RCF (g). *Note: 1800-1900 RCF (g) is optimal; if 1800-1900 RCF is unattainable in a certain centrifuge then 1200-1300 RCF is the minimal acceptable speed.*

Freezer: A -20° C freezer will be used for freezing gel packs for shipping specimens. A - 70° C freezer can **NOT** be used to freeze the gel packs.

Refrigerator: Used to store specimens until they are shipped.

Staff: Staff trained in phlebotomy, blood borne pathogens, and universal precautions

Appendix 3. Daily Temperature Logs SPRINT DAILY TEMPERATURE RECORD

DATE Mo/Da/Yr	-20 Frzr	Refrig	Room	Initials

DATE Mo/Da/Yr	-20 Frzr	Refrig	Room	Initials

Appendix 4. Venipuncture and Processing Procedures Certification Checklist

Optional Training Tool: For Site Use Only; Does NOT have to be sent to Central Laboratory

VENIPUNCTURE	Satisfactory/ Unsatisfactory	Comments
1. Labels checked	___	________________________
2. Participant prepared and procedure explained	___	________________________
3. Venipuncture Form completed.	___	________________________
4. Tourniquet application and release	___	________________________
5. Venipuncture technique	___	________________________
6. Tube collection sequence	___	________________________
7. Inversion technique	___	________________________
8. Stasis obtained (Bandaging of arm)	___	________________________
9. Tube incubation location	___	________________________
10. Needle disposal	___	________________________
PROCESSING		
1. Knowledge of centrifuge operation	___	________________________
3. Spinning red top serum tubes	___	________________________
5. Spinning purple top plasma tubes	___	________________________
6. Urine Processing	___	________________________
7. Disposal of contaminated supplies	___	________________________
8. Time management	___	________________________
PACKAGING AND SHIPPING		
1. Specimens bagged appropriately	___	_______________________
2. Gel pack frozen inside shipping container	___	_______________________
3. Shipping paperwork	___	_______________________
4. Containers correctly labeled for shipping	___	_______________________
5. Shipment pickup and tracking	___	_______________________
MISCELLANEOUS		
1. Quality Control temps and documentation	___	_______________________
2. Laboratory MOP has been reviewed	___	_______________________

Appendix 5. Sample Exam for Certification

Optional Training Tool: For Site Use Only; Does NOT have to be sent to Central Laboratory

PRACTICAL EXAM FOR SPRINT LABORATORY TECHNICIAN

1. What blood collection tube(s) are collected for the randomization visit and in what order?
2. What blood collection tube(s) are collected for the 1, 3, 18, 30, 42, and 54 Month visits and in what order?
3. What blood collection tube(s) are collected for the 6, 36, and 60 Month visits and in what order?
4. What blood collection tube(s) are collected for the 12, 24, and 48 month visits and in what order?
5. Which visits include a urine specimen?
6. Which tubes should be placed in a refrigerator for 20-30 minutes before centrifugation?
7. Which tubes must incubate for 20-30 minutes at room temperature before centrifugation?
8. T or F; the gel pack must be frozen inside of the Styrofoam shipping container in a -20 degree C freezer.
9. T or F; the centrifuged blood samples and urine specimens should remain in the refrigerator until shipment.
10. T or F; it is the clinical site's responsibility to track their lab sample packages by using the FedEx website to ensure that the packages are picked up from their clinic and delivered to the Central Laboratory.

Appendix 6. Supply Reorder Form

Supply Reorder Form

Please complete this form and **FAX** it to the SPRINT Central Laboratory at **612-625-4142 or 612-625-4831 (alternate fax number).** *Please allow **7-10** working days for receipt of your supply order Thanks.*
Central Lab phone number: 612-625-5040

Clinical Field Center ID Number ________ Date: _____ / _____/ _____

Shipping Address: ________________________________

____

____

____

Phone Number: ________________________

Kits *Please indicate desired quantity for each 'Visit type'. To minimize expiration of collection tubes supplied in the kits, do not order more kits than you expect to use within a few months.*

___ 1,3,18,30,42,54,PRN Month Visit Kit

___ 6,36,60 Month Visit Kit

___ 12,24,48 Month Visit Kit

Replacement blood collection tubes/Urine Cups *Please monitor expiration dates on blood collection tubes supplied in the kits. NOTE: collection tubes expire on the last day of the month printed on the label*

___ 7.5-mL SST/gel (red top) ____ 8-mL EDTA/gel (purple top) ___10-mL EDTA (purple top)

___ Sterile Urine Cups ___ Urine Transport Tubes (blue cap) ___ Plasma Transport Tubes (purple cap)

Additional supplies:

_____ FedEx Billable Stamps, pre-printed

_____ FedEx International Air Waybills, pre-printed (For Puerto Rico sites)

_____ Shipping Boxes with gel packs

_____ FedEx UN 3373 Pak Mailing Bag

Appendix 7. Sample Re-Collections (Redraws or Missed Samples)

Occasionally, results for protocol required blood or urine analyses may not be obtained due to collection, processing or storage problems at the sites or damage to or loss of samples during shipment. The text below describes the standard procedure for re-collecting missing or incomplete samples, and then lists several exceptions to the standard procedure for specific circumstances.

Standard Procedure:

You may collect missed or partial blood and urine samples within **two weeks** of the original visit date, using the REDRAW (RDW) Lab form. Only the missing samples need to be recollected, although a complete set is preferable if allowed by your local IRB.

Exceptions:

1) If the participant is unable to return within two weeks of the original visit date, you may collect samples 2 – 4 weeks after the original visit using the REDRAW (RDW) lab form. You should re-collect the entire sample set unless specifically prohibited by your IRB. Samples should not be re-collected more than 4 weeks after the original visit date except as noted in (2) and (3) below.

2) Samples for 12 month, 24 month and 48 month visits, where we collect lipids plus serum and plasma for storage, should be collected at the next visit, but may be collected up to 9 months after the original visit date. Use the REDRAW (RDW) Lab form for these collections and be sure to indicate the ANNUAL Visit code at the bottom of the form.

3) The DNA (genomic) sample can be re-collected at any visit (no time limits; re-collect as soon as possible).

Additional Notes:

Send all incomplete sample sets to the Central Lab in case the participant is unable to come back to have samples re-collected (some samples are better than no samples).

Please communicate clearly all details involved with sample re-collections on the laboratory collection form that is sent with each sample set.

These sample re-collection time frames have been established and approved by the SPRINT MPQC subcommittee.

Appendix 8. Stored Samples

The SPRINT study stores genetic material, blood and urine.

Access to genetic material is tightly controlled according to participant responses to the three genetic consent questions on the model informed consent:

- I agree to allow my genetic sample (DNA) to be used to study hypertension, heart and vascular disease, kidney disease, memory and brain disorders, and their risk factors
- I agree to share my genetic sample (DNA) with other investigators who meet National Institutes of Health (NIH) standards and procedures
- I agree to allow my genetic information to be transferred to the NIH GWAS Data Bank and to be shared with investigators who may not be associated with the SPRINT study

These three genetic consent questions are data entered by the clinical site via the SPRINT web site, and can be edited as needed (e.g., if the participant is re-consented and changes their answer) by clicking on "Edit Layered Consent Questions" on the participant page. For each participant, the values entered into the website should correspond to the most recent consent where these questions were administered.

If the answer to the genetic layered consent question is data entered as 'No', samples will not be used for that purpose.

At the conclusion of the SPRINT study, samples with participant permission will be moved to a long term storage facility called a biorepository that will be maintained by the National Institutes of Health or one of its agencies or subsidiaries. Those without consent for long-term storage will be destroyed, and the destruction of stored samples will be documented. Should samples need to be destroyed prior to the end of the study, clinic staff will notify their CCN Coordinator. The CCN Coordinator will communicate with the CC staff responsible for notifying the lab of samples that must be destroyed. The CC is responsible for communicating with the Central Lab whenever stored samples need to be withdrawn for use or destroyed.

There are no model consent questions of this sort for urine or non-genetic blood storage, since those are considered less sensitive. If the clinic has specific elements related to these types of blood storage in their consent, they should contact their CCN Coordinator, who will discuss with the CC and the lab how to transfer this information to the necessary parties.

SPRINT Chemistry Profiles 01/29/2011

Patient Data Screen

NOTE: Begin with a search for existing patient by HMO ID

Patient ID format:	**U4007**-x	Location plus **auto-assigned ID**
Patient Name Format:	S,12345678	(*S, Lab ID*)
Date of Birth:	01011900	
Sex:	U	
HMO ID:	8-character SPRINT Patient Study ID (ex. 10162615)	

Event Data Screen

Client ID:	**U4007xxx**	Location plus site ID, where xxx=site ID (ex., U4007101)
Attending phys 1:	098 (enter the letter "O")	

New Order Screen

*Order Cmt:	enter visit number (ex., RZ1, 9M, 60M, PRN, etc.)	
* Modifier(s):	specimen-related comments, if any (ex. RBCP)	

Special Admin Data Screen

Charge to:	**C**

Auto Assign Accn

Auto Assign Accn #	**Y**
*Specimen Comment:	8-digit bar coded Lab ID

VISIT	RZ1,RZ2	1M	3M	6M	12M	18M	24M
	ASSESS	NA	NA	NA	NA	NA	ASSESS
	CLIPID	K	K	K	K	K	CLIPID
	CRDUR	CL	CL	CL	CL	CL	CRDUR
	UMALBR	CO_2	CO_2	CO_2	CO_2	CO_2	UMALBR
	FASTYN*	BUN	BUN	BUN	BUN	BUN	FASTYN*
		CREATR	CREATR	CREATR	CREATR	CREATR	
				CRDUR	CLIPID		
				UMALBR	CRDUR		
					UMALBR		
					FASTYN*		

VISIT	30M	36M	42M	48M	54M	60M
	NA	NA	NA	ASSESS	NA	NA
	K	K	K	CRDUR	K	K
	CL	CL	CL	UMALBR	CL	CL
	CO_2	CO_2	CO_2	FASTYN*	CO_2	CO_2
	BUN	BUN	BUN		BUN	BUN
	CREATR	CREATR	CREATR		CREATR	CREATR
		CRDUR				CRDUR
		UMALBR				UMALBR

PRN VISITS	PRN
	NA
	K
	CREATR
	BUN

result at Order Entry (Yes or No)

Orderable Test/Panel Battery	Test(s) included	Pre-fill Container	Wksht
ASSESS	NA sodium	RG	FRCH
	K potassium		
	CL chloride		
	CO2 bicarbonate		
	BUN urea nitrogen		
	CREATR creatinine		
	GLUR glucose		UVR
CLIPID	CHR cholesterol	RG	FCOBAS
	TRR triglycerides		
	HDL HDL – cholesterol, direct		FRHDL
	LDLR LDL-cholesterol		
CRDUR	Urine creatinine	UR	FCX3
UMALBR	Urine microalbumin, ProSpec		FRPS
UMALI, mg/L	Microalbumin, mg/L		
UMALIN, mg/dl (masked)			
UMALCR, mg/g creat	Albumin:Creatinine Ratio(ACR), mg/g creat		
FASTYN*	Fasting status (Yes or No)		FRCH3

*result at Order Entry

Chapter 8. ECG

Electrocardiography Assessment Manual

THE SYSTOLIC BLOOD PRESSURE INTERVENTION TRIAL (SPRINT)

The SPRINT Central ECG Reading Center (CERC)
Epidemiological Cardiology Research Center (EPICARE)
Division of Public Health Sciences
Wake Forest University School of Medicine
Winston Salem, NC

Table of Contents

8.1. Introduction

8.2. Background and Purpose

8.3. Field Center Procedures
- 8.3.1. ECG Acquisition Procedures
  - 8.3.1.1 Electrocardiograph
  - 8.3.1.2 Supplies
  - 8.3.1.3 Preparation for ECG Recording
  - 8.3.1.4 Location of the ECG electrodes
  - 8.3.1.5. ECG recording
- 8.3.2. Local ECG reading
  - 8.3.2.1 Rationale
  - 8.3.2.2 Alert ECGs
- 8.3.3. Data management procedure
  - 8.3.3.1 Communications setup for transmission
  - 8.3.3.2 Before transmitting ECGs to the CERC
  - 8.3.3.3 Transmitting ECGs to the CERC
  - 8.3.3.4 Directory management

8.4. Reading Center Technical Details
- 8.4.1. Data processing
- 8.4.2 Data reporting

8.5. Quality Control Procedures
- 8.5.1 Quality grades
- 8.5.2 Certification/Recertification procedures
- 8.5.3. Examples of common ECG quality problems and possible solutions

Appendix
- A. The SPRINT ECG reading center contact list
- B. MAC 1200 programming and setup
- C. Transmission of SPRINT study ECGs to the CERC
- D. SPRINT ECG form
- E. ECG data flow

8.1 INTRODUCTION

The Epidemiological Cardiology Research Center (EPICARE) is the SPRINT Central ECG Reading Center (CERC). It is located at Wake Forest University Health Sciences, Winston Salem, NC. The CERC main contacts are listed in Appendix A.

8.2 BACKGROUND AND PURPOSE

In the SPRINT study, ECGs will be recorded at the baseline visit and at months 24, 48 and 60 of the follow-up period. The ECG recording will serve to establish the distribution of ECG findings at the baseline and development of new findings in the follow up visits. The SPRINT CERC will use the Minnesota ECG classification as basis for detection of myocardial infarction, myocardial ischemia, left ventricular hypertrophy, arrhythmias, and conduction defects. A number of continuous ECG measurements that are known to be associated with a poor prognosis will be also detected from the study ECGs.

8.3. FIELD CENTER PROCEDURES

The field center procedures include ECG acquisition (section 5.3.1) and local ECG reading by the clinic physician (section 5.3.2).

8.3.1. ECG ACQUISITION PROCEDURES

At each SPRINT ECG examination visit, *three immediately sequential* digital ECGs will be recorded for each participant using a GE MAC 1200 electrocardiograph. The ECGs stored in the MAC1200 will be transmitted to the CERC at least twice weekly.

8.3.1.1 Electrocardiograph

The electrocardiograph to be used for ECG recording and transmission in the SPRINT study is the GE MAC 1200 portable electrocardiograph The MAC1200is a portable device and can easily be moved from one location to another.

- Each machine will be configured specifically for the SPRINT study ECG acquisition and transmission.
- The MAC1200 is to be used for resting ECG recording only.
- It is not intended for use as a vital signs physiological monitor.
- The MAC1200 has a customized menu specific to the SPRINT study.
- Appendix B includes the instructional charts that outline the SETUP for the SPRINT MAC 1200 ECG machines.
- All of the SPRINT ECG technicians should become familiar with the GE MAC 1200 Operator’s Manual.

8.3.1.2. Supplies

Table 1 summarizes the equipment and supplies needed for recording and transmitting ECGs. Always order supplies in advance.

Table 1

- GEMSIT MAC1200 Electrocardiograph with its 10 lead acquisition module and attached modem
- Flexible measuring tape
- Telephone jack cable
- Scissors
- Felt tip non-toxic washable markers
- The CERC contact list (Appendix A)
- Reference guides for "Patient Data Entry" (Table 2)
- Reference guide for "Transmission of ECG" (Appendix C)
- GEMSIT MAC1200 operation manual
- MAC1200 ECG paper
- GEMSIT disposable silver chloride electrodes
- Alcohol swabs and gauze pads
- Cotton surgical tape
- Examining table disposable paper

8.3.1.3 Preparation for ECG recording

- Participant should be relaxed and comfortable in supine or semi-recumbent position.
- Examination table/bed should be adequate to comfortably accommodate the participant.
- Supply drape for exposed upper torso.
- An additional covering may be needed to prevent the participant from becoming chilled.
- Make sure ankles and wrists are accessible for electrode application.
- ECG electrode placement should be performed with the technician standing to the participant's left side.
- Reference guide for "Participant Data Entry" instructions should be available to insure accuracy.
- Supplies needed for ECG acquisition should be assembled and arranged efficiently.

8.3.1.4 Location of the ECG electrodes

8.3.1.4.1 Location of limb electrodes (Figure 1)

RIGHT LEG (RL) and LEFT LEG (LL):

- On the inner side of the right leg (RL), above the ankle, rub briskly an area about 1-2 inches in diameter with an alcohol swab using firm, circular motions
- Mark the position to place the electrode later.
- Repeat this procedure for the left leg (LL).
- In amputees, the leg lead electrode may be placed higher up on the torso.

RIGHT ARM (RA) AND LEFT ARM (LA):

- Rub the inner side of the right arm (RA) above the wrist similar to what you did with the right and left legs.
- Mark the position to place the electrode later.
- Repeat the process for the left arm (LA).
- In amputees, the arm electrode may be placed on the shoulder, below the clavicle.

FIGURE 1

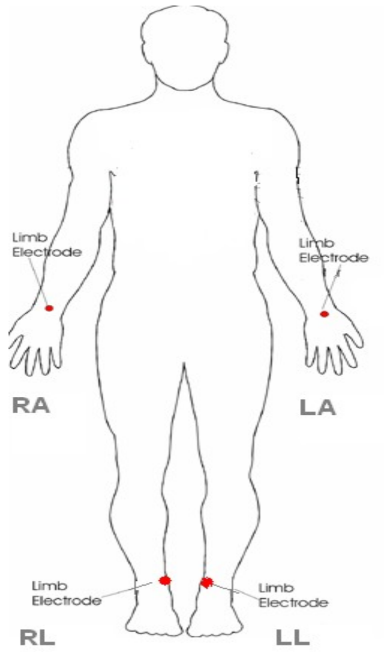

8.3.1.4.2 Location of chest electrodes

V1 and V2:

- First, locate the sternal angle about the width of your 3 middle fingers below the sternal notch (Figure 2). Mark a dot over the sterna angle.
- Feel the sternal angle between the index and middle fingers of your right hand, keeping the fingers wide apart and moving your fingers firmly up and down. While feeling the sternal angle, move your fingers to the left side of the sternum and feel the 2nd rib between your fingers where it joins the sternal angle.
- Move your middle finger to the interspace below the second rib and with your index finger locate the interspace below the next rib (3rd) and again below the next (4th) rib. This is the 4th intercostal space. Mark an **X** at this level at the midsternal line. **X** is the reference level for V1 and V2. Mark their locations at the right and left sternal border (Figures 2 and 3).
- Measure the distance in inches (to the nearest ½ inch) from the sternal notch to the **X** mark using a flexible measuring tape. This is the NV distance (Figure 4).
- Enter the NV measure as at least two digit number (ignoring the decimal point) in the height field in the participant information screen of the ECG machine e.g. NV measure of 5.0 should be entered as 50, NV measure of 5.5 should be entered as 55, and NV of 11.0 should be entered as 110, etc. You need to enter the NV

measure in the ECG form too (Appendix D)

FIGURE 2

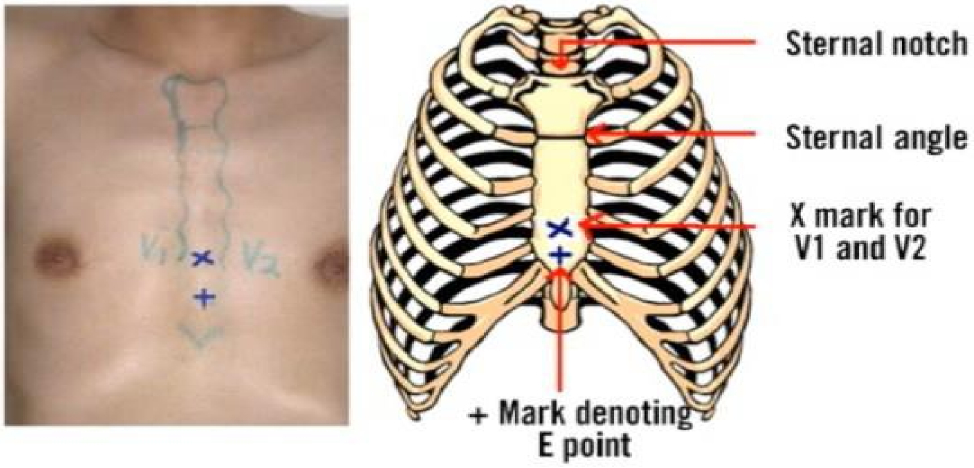

FIGURE 3

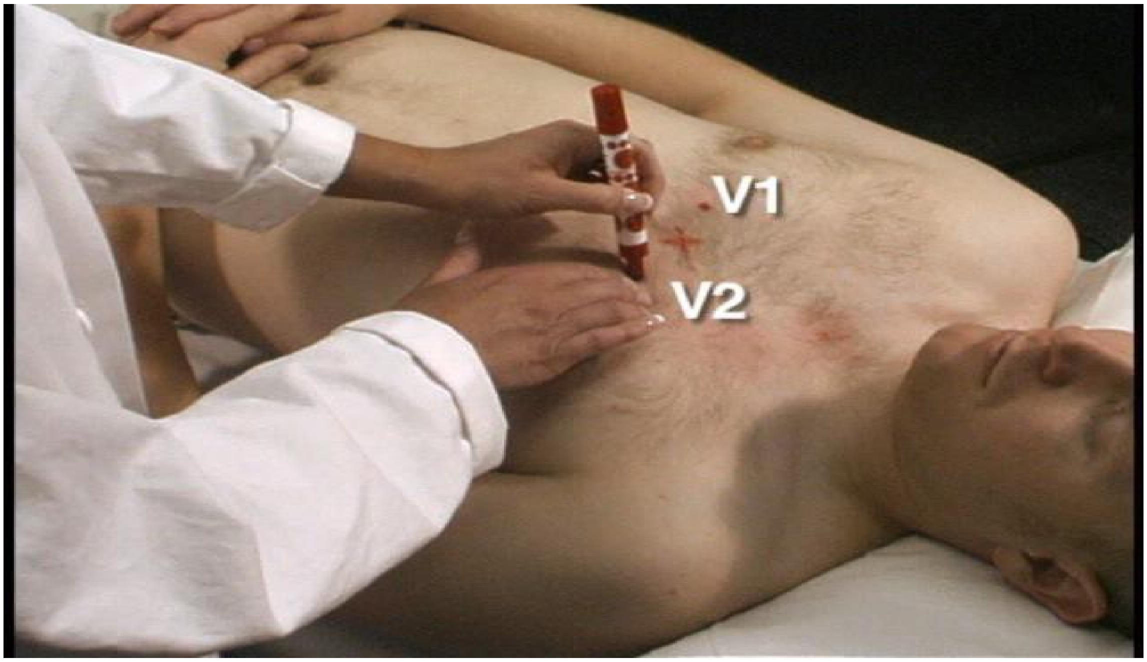

FIGURE 4

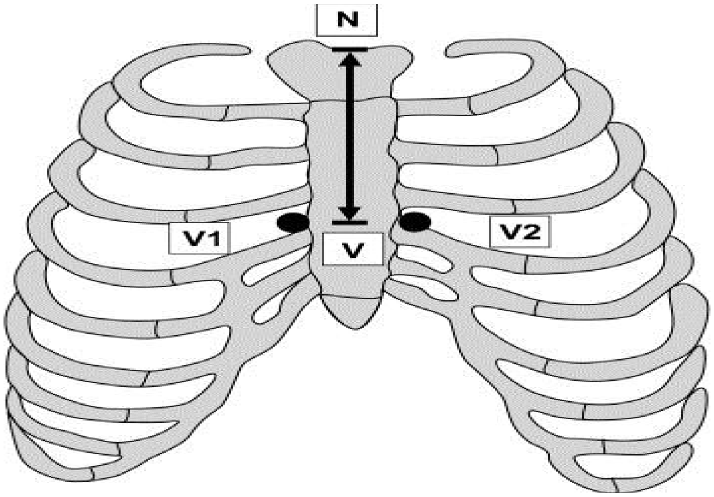

V4 and V6

- From the location of V2, palpate with the middle finger of your right hand the intercostal space and follow it laterally outside the sternal border and at a slight angle down. Feel the 5th rib between your index and middle fingers and then feel the 5th intercostal space with your index finger.
- At the level of the 5th intercostal space, mark a **+** sign at the midsternal line below your **x** mark for V1-V2 level. This **+** is the reference level "E" for V4, V5, and V6 (Figure 2 and Figure 5).
- In overweight persons and in women with tender breast tissue, it is often difficult to locate the 5th intercostal space. In such a case, mark the **+** sign for E point 1 ¼ in below your reference level **X** for V1 and V2 (in smaller adults, 1 inch is enough).

FIGURE 5

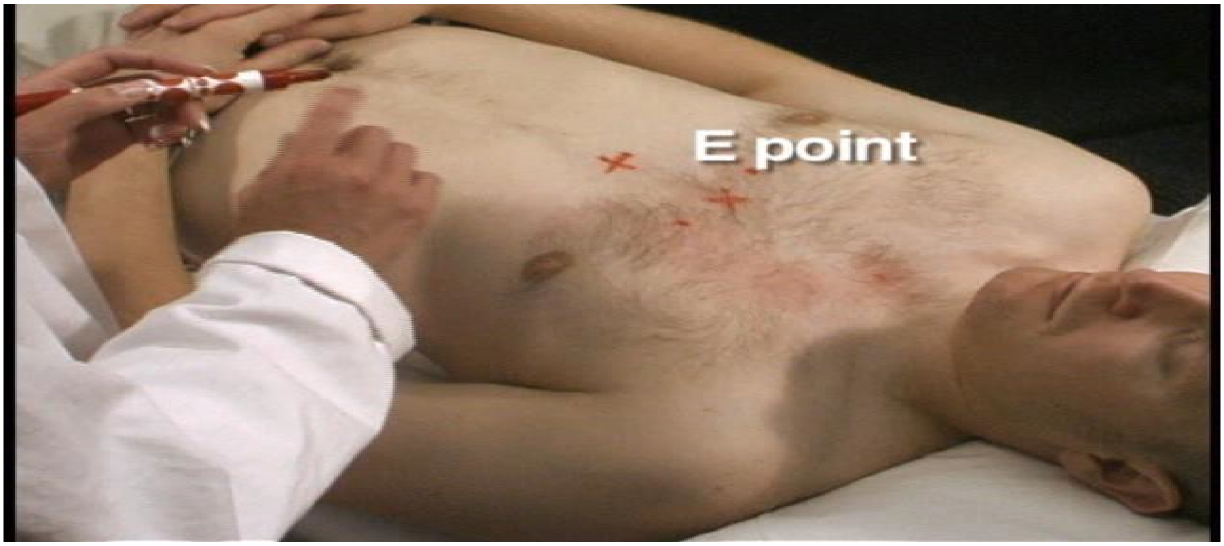

APPROXIMATE LOCATION OF V6

- Move the left elbow laterally without moving it anteriorly or posteriorly, while observing the anterior and posterior axillary folds. The left elbow must be supported properly.
- Follow a line exactly in the vertical midplane of the thorax (mid-axillary line - Figure 6) down where the line meets the horizontal plane of E point. Using your marker, make a vertical 1-2 inch long line there as an approximate location of V6 (Figure 7).

FIGURE 6

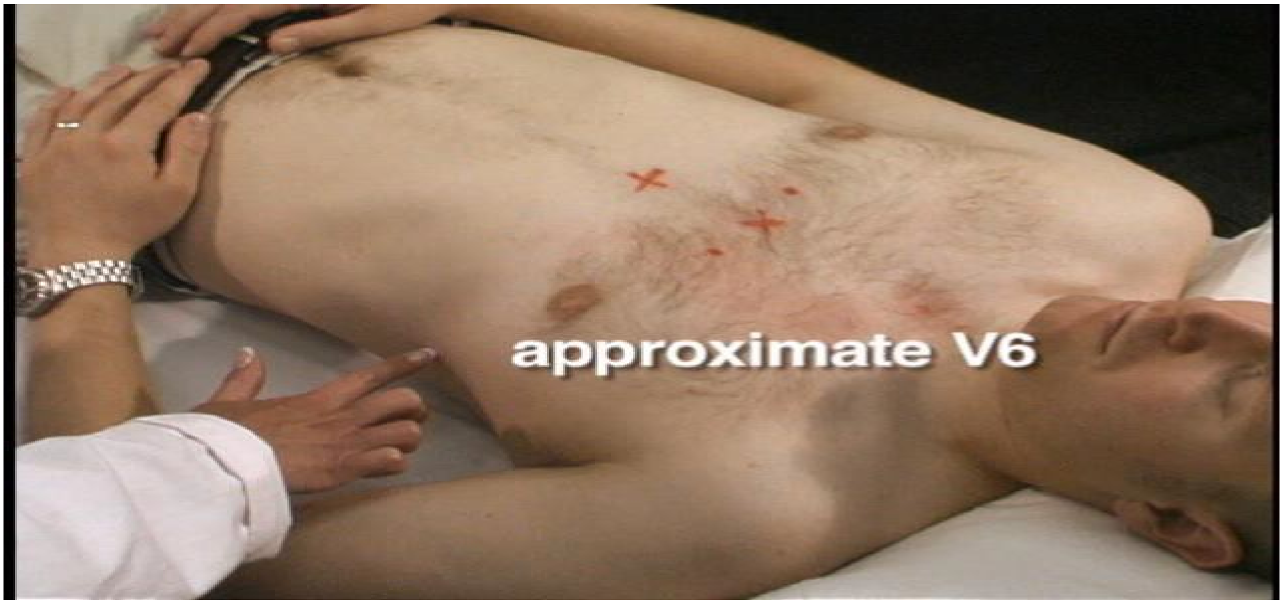

FIGURE 7

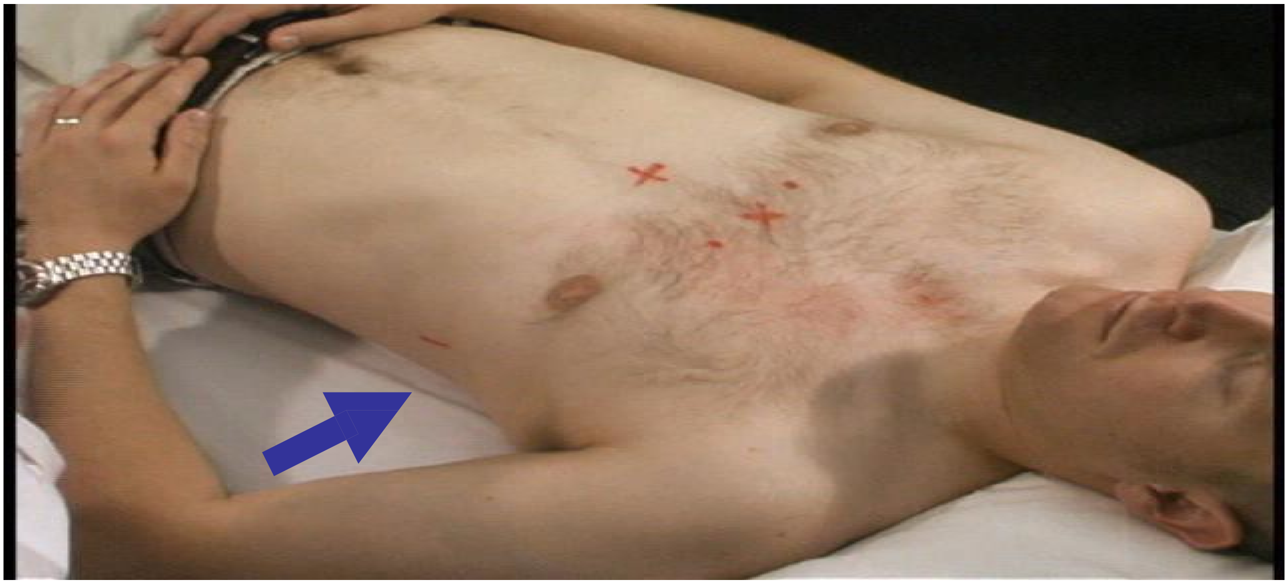

EXACT LOCATION OF V6

- Exact location of V6 is determined by using a flexible measuring tape.
- Place the measuring tape horizontally on the chest and extend it from the E point until it crosses the 1-2 inch vertical mark you made before (the approximate V6 location). The crossing point would be the exact V6 location (Figure 8)
- Mark the exact location of V6

FIGURE 8

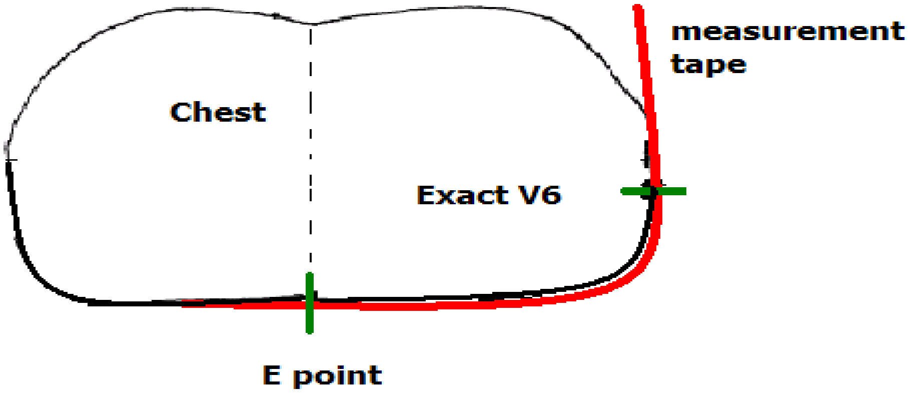

EXACT LOCATION OF V4

- V4 is also located by using a flexible measuring tape
- Use the measuring tape to identify the halfway point between the exact location of V6 and the E point. This mid-chest point is the location of V4.
- Measure the distance from the V4 location to the E point in inches (to the

nearest ½ inch). This is the mid-chest measurement (Figure 9).

- Enter the mid-chest measurement (Figure 9) as at least two digit number (ignoring the decimal point) in the weight field in the participant information screen of the ECG machine e.g. a mid-chest measure of 6.0 should be entered as 60, 12.0 as 120, etc. You need to enter the mid-chest measurement in the ECG form too (Appendix D)

FIGURE 9

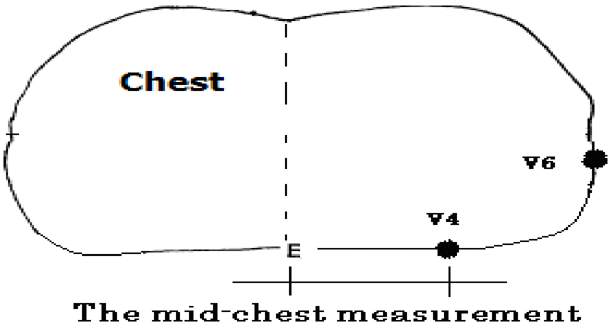

LOCATIONS OF V3 and V5

- Mark V3 exactly halfway between V2 and V4 (Figure 10).
- Mark V5 exactly halfway between V4 and V6 (Figure 10).

FIGURE 10

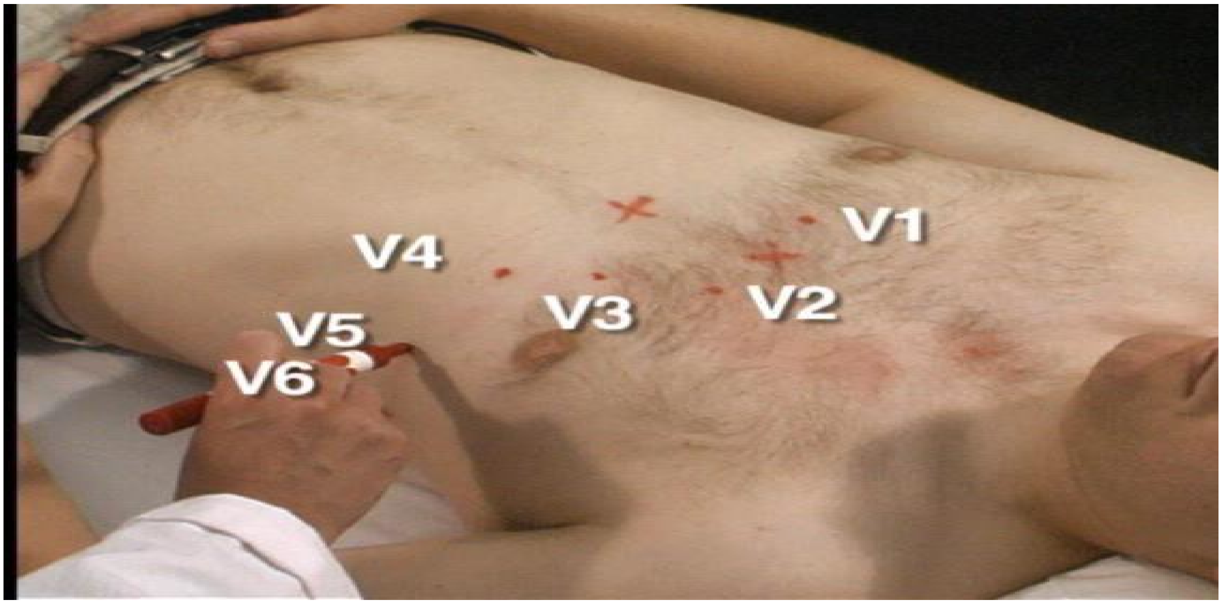

8.3.1.4.3 Attaching the electrodes:

- After you have marked electrodes positions and rubbed them with alcohol swabs, you may apply the electrodes.
- Lower limb electrodes should be facing up, while upper limb electrodes could be facing up or down
- Do not place electrodes directly over bone.
- Attach lead wires in the same, correct order every time to establish routine and to eliminate lead swaps.
- Position the MULTI-LINK on the participant's abdomen.
- Grasp each lead at the MULTI-LINK attachment point.
- Follow lead wire to the electrode attachment end.
- Attach wire to electrode, making sure clip is not in contact with electrode adhesive.
- Make sure lead wires have some slack and are hanging loosely.
- You may secure the lead wire to the skin by applying paper tape 1-inch below the clip, especially if the ECG shows baseline noise despite careful preparation.

8.3.1.5. ECG recording

Three ECGs will be recorded for each participant.

- Turn on the MAC1200.
- Allow the machine to go through the "self-test." Do NOT press "R."
- Press "Pat info" key to enter the participant information
- Use the participant data entry sheet as a reference guide (Table 2).
- Press the START/STOP key to return to the ECG screen on the ECG machine.
- Press the START/STOP key again to record the 1st ECG.
- After printing the 1st ECG, press the START/STOP key to record the 2nd ECG.
- After printing the 2nd ECG, press the START/STOP key to record the 3rd ECG

Table 2 Participant Data Entry into the MAC1200 for the SPRINT Study

**Category Listed on Mac1200**	**Entry to Machine by ECG Technician**
NEW PATIENT	YES
LAST NAME	Do not enter the participant's last name. Enter **SPRINT**.
FIRST NAME	Enter SPRINT exam visit number: 0, 24, 48 or 60
DATE OF BIRTH	Enter MM/DD/YYYY
PARTICIPANT ID	Enter the ID number given by the CC
SECONDARY ID	Enter same as Participant ID
PACEMAKER	Select YES or NO
GENDER	Select Male or Female
HEIGHT	Enter the NV measurement **DO NOT ENTER HEIGHT**

WEIGHT	Enter the mid-chest measurement **DO NOT ENTER WEIGHT**
RACE	Choose Other and highlight defined race codes (defined on the MAC1200)
REFERRING PHYSICIAN	No action required
TECHNICIAN	Choose Other and select technician
LOCATION	No action required

8.3.2. Local ECG reading (Alert ECGs)

8.3.2.1. Rationale

Because there are no available diagnostic statements from the CERC except as monthly measurement reports to the SPRINT CC, the local clinic reading of the ECGs is essential for safety of the participants.

8.3.2.2. Alert ECGs

The ECG technician should look for the following in the printed diagnostic statement on top of the ECG printout:

a) Heart rate < 40 beats/minute
b) Heart rate >120 beats/minute
c) Acute myocardial infarction (Figure 11)
d) Acute myocardial ischemia
e) Ventricular tachycardia (Figure 12)
f) Complete atrioventricular block (Figure 13)
g) *Atrial fibrillation or flutter (Figure 14 and 15)

* Only new atrial fibrillation or flutter defined as atrial fibrillation or flutter with no documentation of prior history of these conditions. You may ask the participant about his/her history of atrial fibrillation or flutter.

These ECG findings need to be reviewed by a physician at the clinical sites before the participant leaves the clinic. The clinic physician will decide if any further action is needed. It is important not to alarm the participant by immediately revealing these unconfirmed interpretative statements before discussing the ECG with a site physician. This is because the diagnostic statements on the ECG printout are not always correct. In SPRINT study, these statements are only used as a safety measure to avoid missing an acute condition that needs prompt treatment or notification to the participant.

There are other significant ECG abnormalities that warrant treatment, but because they do not require prompt action or immediate notification to the participant, they are not included in the above list. Since local reading of the study ECGs for “alerts” is not part of the ECG reading center procedure, this list of ECG abnormalities may be modified by adding or deleting more ECG abnormalities to match the overall safety measures implemented by the SPRINT study. Please refer to the safety MOP chapter for further details.

Figure 11 Acute inferior (upper panel) and acute anterior (lower panel) myocardial infarction

Diagnosis key points: Elevated ST segment in a group of adjacent leads with or without Q waves and with or without ST depression in other leads. Patients usually will have chest

pain

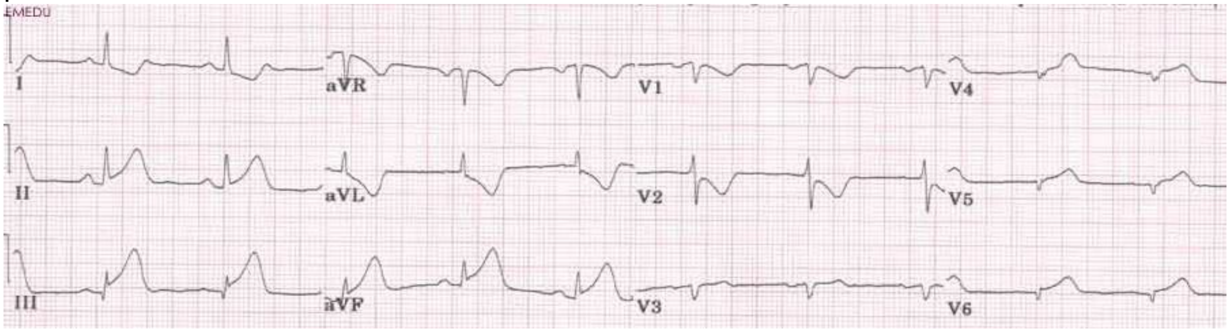

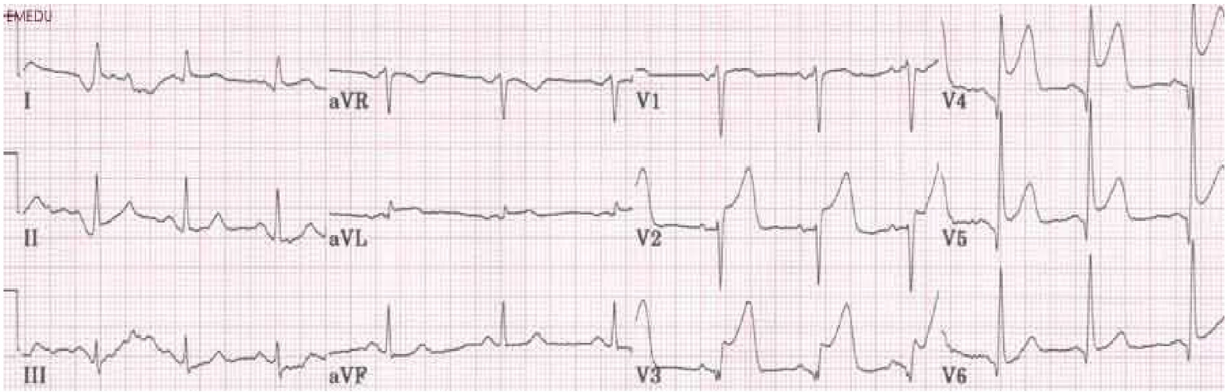

Figure 12 Ventricular tachycardia

Diagnosis key points: Wide complex tachycardia (HR≥110) with QRS not preceded by P wave. The participant will be mostly restless

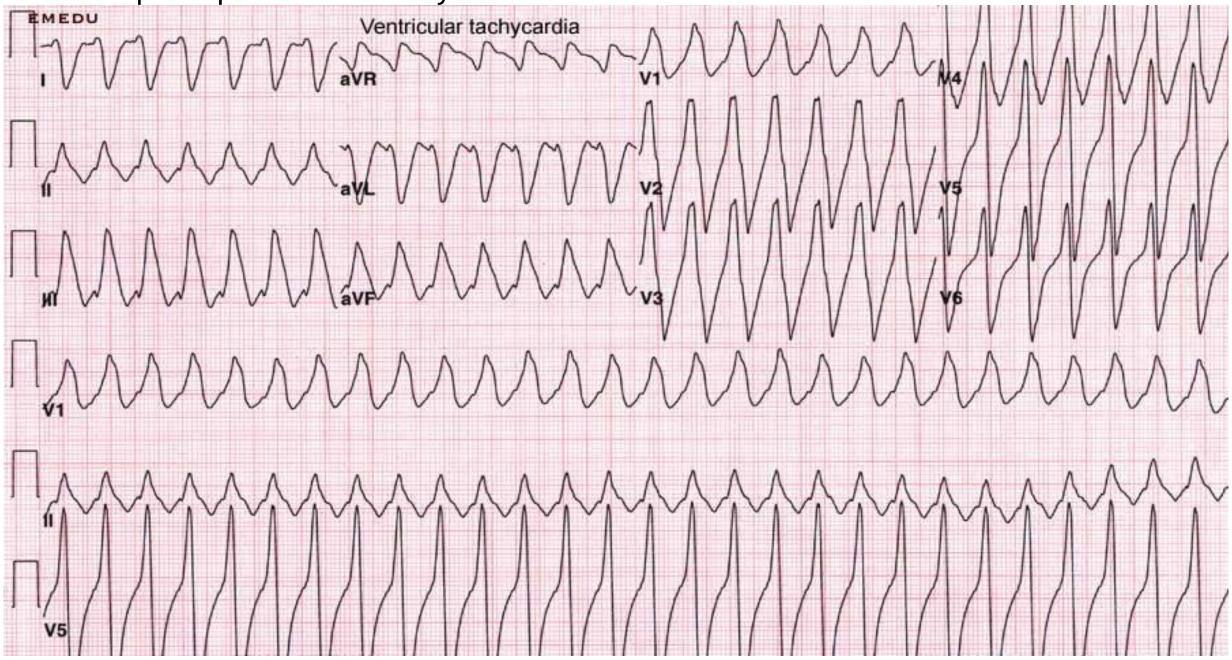

Figure 13 Complete (3rd degree) atrioventricular block

Diagnosis key points: Slow heart rate (around 40 beats per minute) with no relation

between the P wave and the QRS

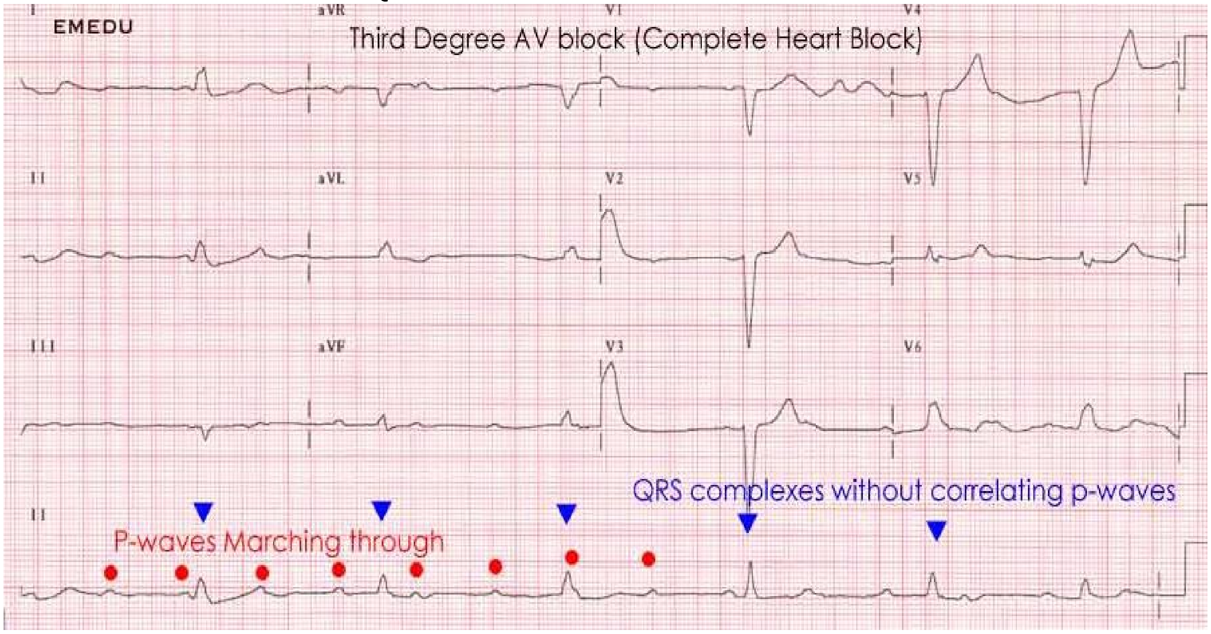

Figure 14 Atrial fibrillation

Diagnosis key points: irregular QRS complexes (heart rate) and absence of the P wave

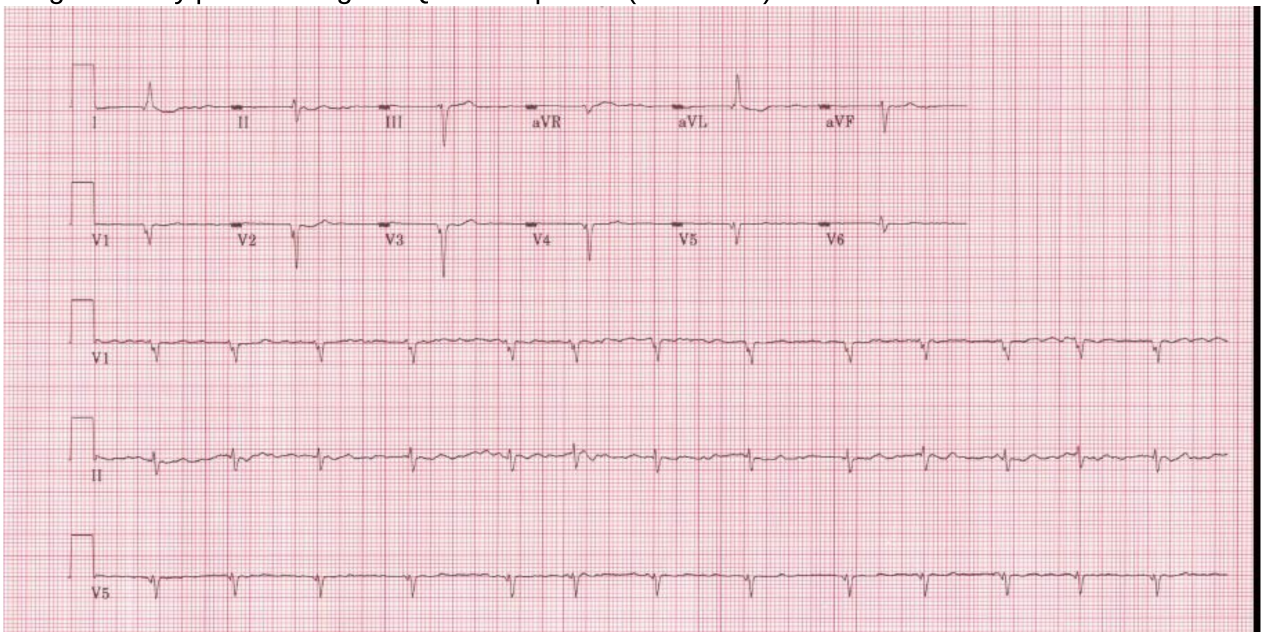

Figure 15 Atrial flutter

Diagnosis key points: multiple P waves; saw-teeth pattern (as in V1), mostly regular but could

be irregular with a certain pattern (regular irregularity)

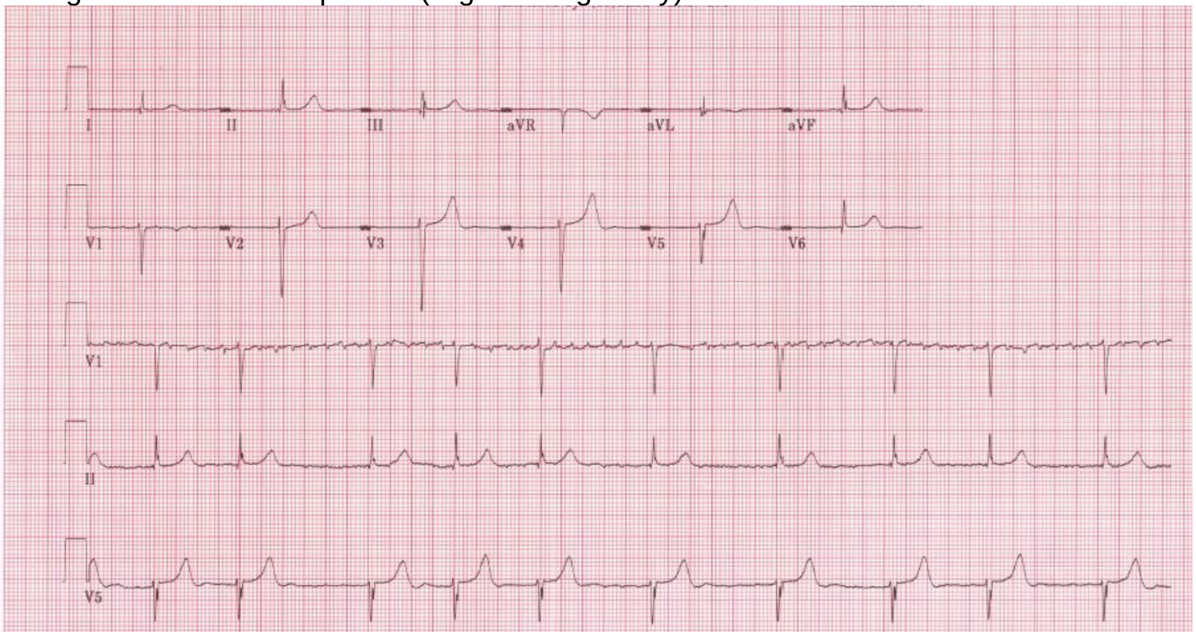

8.3.3. Data management procedure

8.3.3.1 Communications setup for transmission

Internal set up of the ECG machines must be done according to the instructions established by the CERC. Correct internal set up should enable the clinics to transmit the study ECGs via a phone line to the reading center. Adding 9 (or other number) to get an outside line and/or adding an access code for long distance are taken into consideration. [NOTE: Contact the CERC any time with questions]

8.3.3.2 Before transmitting ECGs to the CERC

- Ensure that all previously transmitted ECGs are deleted.
- Check to ensure that all IDs are valid.

8.3.3.3 Transmitting ECGs to the CERC

- Secure the modem cable into the 9-pin connector found on the right side of the MAC1200 and the 25-pin connector found on the rear of the modem.
- Plug one end of the phone cable into the connector marked "**LINE**" on the rear of the modem and the other end into any "**analogue**" (fax) phone line**.**
- Start at the 12-lead screen. While holding the "Shift" key down, press the "Store/Retrieve" key. Press the down arrow 3 times and then hold the shift key and the down arrow together to get to the desired ECG to be transmitted. The screen will show black squares on the right and left sides of the ECG selected for transmission.
- To skip an ECG press the down arrow without using the shift key.
- Repeat this procedure until all ECGs that are to be transmitted have been selected.

- Once selections are made, press the “Enter” key. This will return you to the top of the screen.
- Use the right arrow to highlight “Send” and press the “Enter” key.
- Another screen will appear which states “to start transmission, press enter”. Once transmission is complete, press the “Start/Stop” key, located on the far bottom right of the keyboard, to return to the 12-lead screen.
- Confirmation of receipt of transmitted ECGs could be made by logging into the SPRINT/EPICARE website using a user name and password specific to each clinic. Allow 24 hours between transmission and confirmation of receipt through the website and deletion of ECGs from the ECG machine to allow system backup at EPICARE. Instruction on how to log into the SPRINT/EPICARE web-site, along with screen instructions, will be communicated to the clinical sites before the study starts.

8.3.3.4 Directory management

Keep your directory correct and current by doing the following:

- BEFORE TRANSMISSION: Delete all unwanted ECGs like those with flat lines, poor quality or duplicates. Correct any errors in participant data entry like ID numbers, or NV and mid-chest measurements
- AFTER TRANSMISSION: Delete transmitted ECGs ONLY after confirming that EPICARE has successfully received the ECGs.

8.4 READING CENTER TECHNICAL DETAILS

Set-up of the machines is ONLY allowed to be done at the CERC or with assistance of one of the CERC staff or an authorized study personnel if it has to be done at the clinic. It may be necessary to re-program the machine after the start of the study if a malfunction occurs, or the battery has been allowed to become dead. The machine set-up and programming instruction are listed in Appendix B.

8.4.1. Data processing

All SPRINT scheduled ECGs (three per participant per scheduled visit) will be electronically transmitted to CERC. ECGs will be received via the GE- MUSE ECG management server. The digital ECGs are stored in an electronic database at the SPRINT CERC, in a Marquette measurement matrix, by participant ID. This database will remain unaltered. Additionally, a second and third database will be created after technician editing of correct onset and offset of the waveforms. These two databases are then transformed into Minnesota Code and Novacode categories by the EPICARE ECG coding program. A diagram of the data flow is outlined in Appendix G

8.4.2 Data reporting

The format and route of data transfer will be determined by agreement between the Coordinating Center (CC) and the CERC. Monthly reports will be sent from the CERC to the CC. All electronic ECGs will be processed and reported within 30 days from receipt.

8.5 QUALITY CONTROL PROCEDURES

8.5.1 Quality grades

The ECG reading center evaluates and ranks the ECG quality through an automated system with visual confirmation of the results if needed. There are 4 grades; 0, 1 and 2 (which are automatically assigned by the GE-MUSE) and 5 which is manually decided by EPICARE staff for poorest quality- No grade 4. The best grade is 0 and the worst is 5. Generally, grades 0 and 1 are difficult to separate visually and they are considered good. Grade 2 is given to ECGs that have problems that will not significantly interfere with appropriate reading. Grade 5 ECG are given for the ECGs that there have major problems that interfere with accurate reading. The alarming level of poor quality is having more than 5% of the ECGs with quality grade 5 and 2. The CC and the network will be contacted if alarming level of poor quality is reached. A monthly QC report will be sent to the SPRINT CC along with the ECG data results. Networks can use these monthly reports to track the quality of their ECGs.

8.5.2 Certification/Recertification procedures

- All ECG technicians **must go through the certification** process before they are allowed to acquire study ECGs.
- Each technician must acquire and successfully transmit 2 good quality ECG sets (3 ECGs each).
- The 2 ECG sets should be performed on 2 different volunteers or on 1 volunteer provided that there is at least 30 minutes between each ECG set**.**
- After evaluation of certification ECGs by EPICARE staff, the technicians will be notified of their certification status.
- Rectification may be requested if quality issues arise. Recertification process is the same as the certification process.
- The participant data entry should be done according to the instructions in table 3 after pressing the “pat info” key on the MAC 1200 keyboard

Table 3 Entry into the MAC1200 for certification of technicians **ONLY**

**Category**	**Entry**
New Patient	YES
Last name	Enter technician’s last name
First name	Enter technician's first name
Date of birth	Enter volunteer’s birth date (MM/DD/YY)
Participant ID	Enter 99999999 (Press “Shift” key to enter numbers)
Secondary ID	Enter 99999999 (Press Shift key to enter numbers)
Pacemaker	YES or NO
Gender	M or F
Height	Enter the NV measurement **DO NOT ENTER HEIGHT**
Weight	Enter the mid-chest measurement **DO NOT ENTER WEIGHT**
Race	Choose “Other” and choose defined race codes
Referring physician	No action required. Pre-programmed data
Technician	Choose “Other” and select technician’s last name
Location	No action required. Pre-programmed data

8.5.3. Examples of common ECG quality problems and possible solutions

- EXCESSIVE BASELINE DRIFT (**Figure 17**): This occurs if the participant is moving around or there is tension on the lead wires. Ask the participant to lie still for a few

seconds. Drift in excess of 1 mm between baseline points (QRS onset) of any two successive complexes is a sign of significant drift.

- EXCESSIVE MUSCLE NOISE (**Figure 18**): The participant is either tense due to lack of body support or may be cold. Use a wide bed and blanket to cover the participant.

- BASELINE DRIFT DUE TO TANGLED WIRES (**Figure 19**): Ensure that the wires are not pulling. Be sure to establish a good electrode connection. Lay a towel across the wires, if necessary. Adjusting the angle of the clip at the electrode often helps. You may need to tape down the chest leads; use only hypoallergenic medical tape to prevent allergic reactions. Use a U loop (not a cross loop) with the electrode wires, i.e., the wire should not cross but remain open like a U; never crossover wires.

- LOOSE ELECTRODE CONNECTION (**Figure 20**): Loose electrode connection may cause a wavy baseline in some ECG leads. Check each electrode to ensure that it is secure.

- SIXTY HZ NOISE (**Figure 21**): Periodic 60 HZ noise is sometimes visible in the record. This may be caused by AC interference from a nearby machine. Make a visual check of this before recording the ECG. Unplug any unnecessary surrounding electric equipment *Note:* Jewelry does not cause 60 HZ noise.

- MISSING LEADS AND LEAD REVERSAL (**Figures 22-24**): To minimize the chances of having lead reversal and missing leads, always make sure that there are no flat lines in the ECG recording and/or mainly positive QRS in aVR lead. Also, always have a second look at the connections before recording

Figure (17) Excessive baseline drift due to sudden movement of the participant

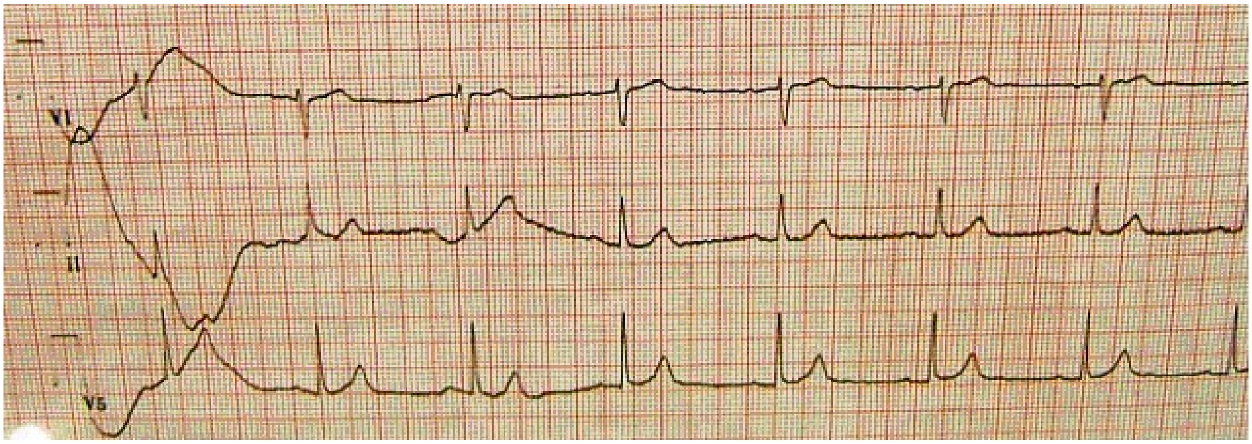

Figure (18) Excessive muscle noise

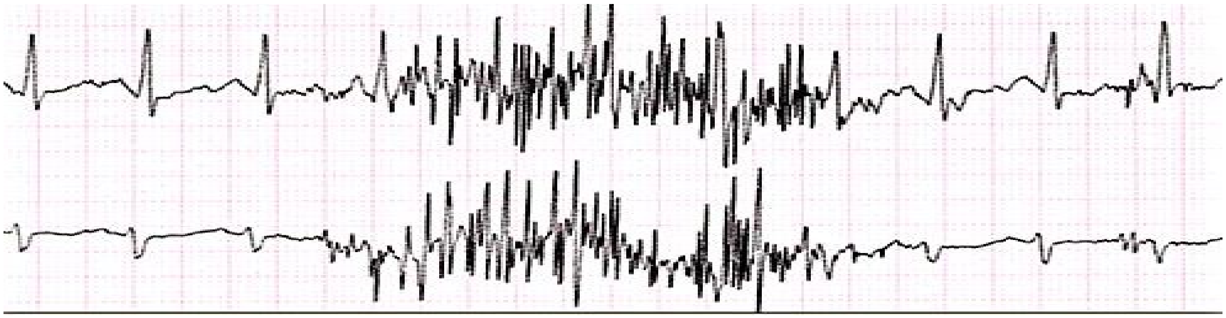

Figure (19) Baseline drift due to tangled wires

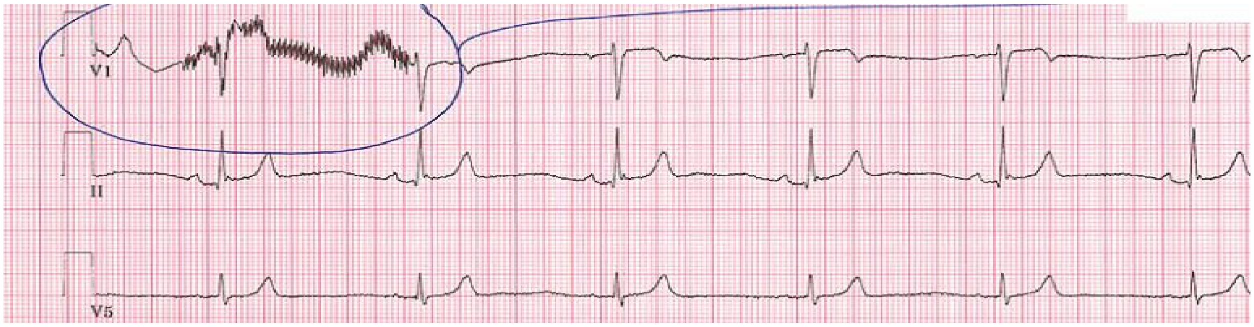

Figure (20) Wavy V1 baseline due to loose electrode

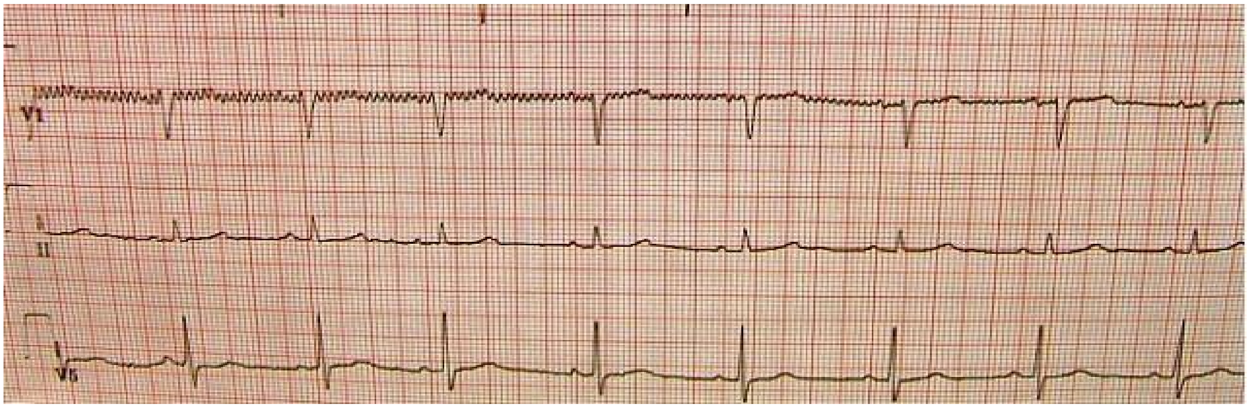

Figure (21) Sixty Hz electrical interference

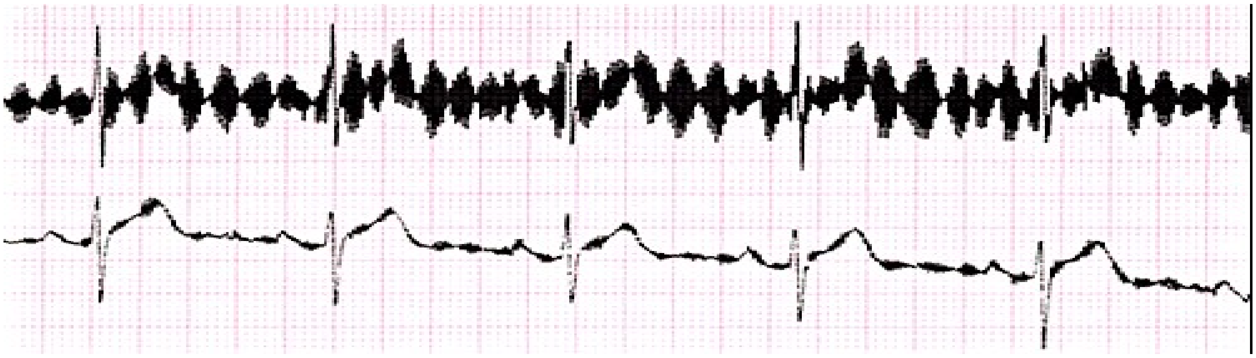

Figure (22) Flat line due to missing V1 lead

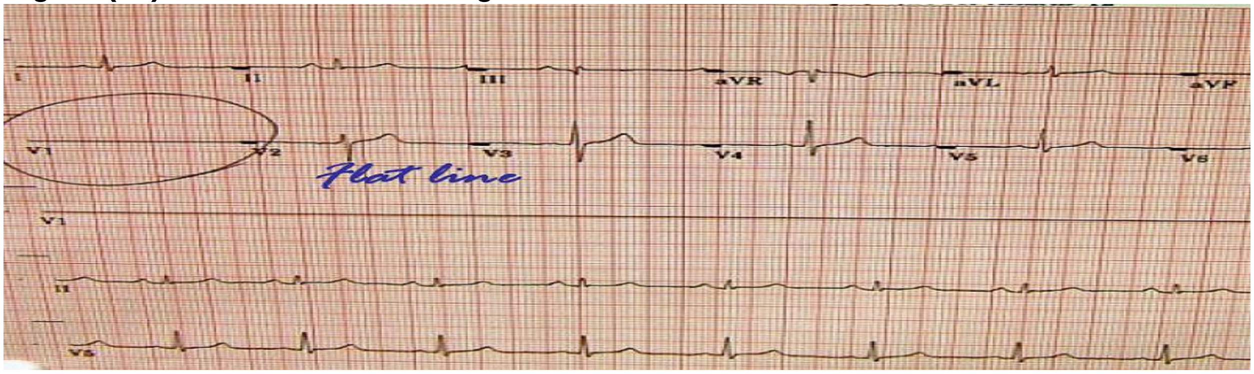

Figure (23) Lead reversal denoted by positive aVR (upper panel) **compared to the normal** (lower panel)

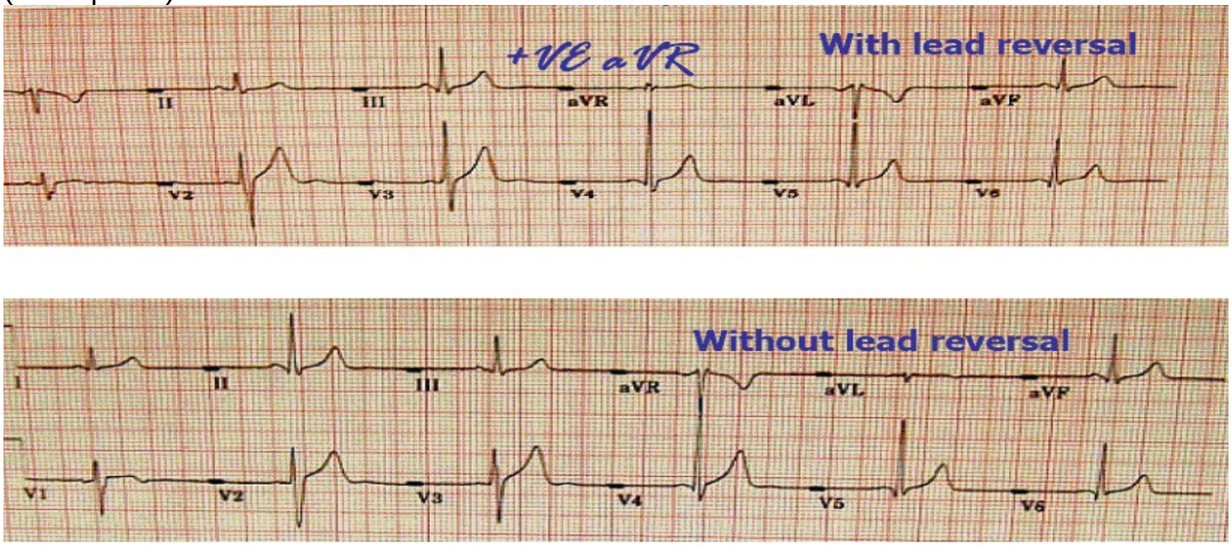

Figure (24) Lead reversal denoted by flat line in one of the limb leads (upper panel) **compared to the normal** (lower panel)

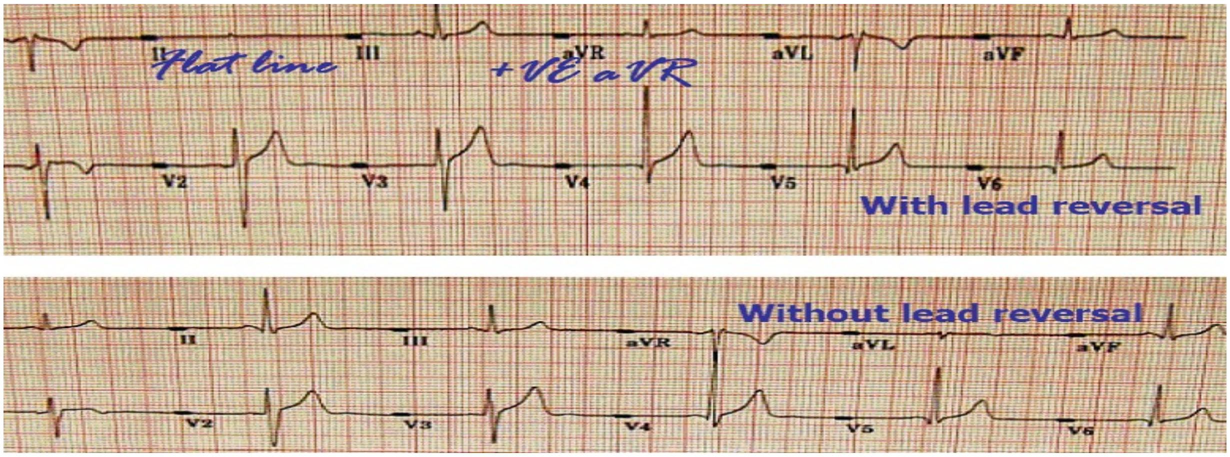

Appendix A

The SPRINT Central ECG Reading Center (CERC) main contacts are:

Elsayed Z. Soliman, MD, MSc, MS, Director of the ECG reading center
Phone: (336) 716-8632
Fax: (336) 716-0834
esoliman@wakehealth.edu

Susan Hensley, BS, Computer ECG Technician
Phone: (336) 716-9616
Fax: (336) 716-0834
shensley@wakehealth.edu

Lisa Keasler, AAS, Assistant Project Manager
Phone: (336) 716-0387
Fax: (336) 716-0834
lkeasler@wakehealth.edu

Charles Campbell, AAS, BS, Data Manager
Phone: (336) 716-3915
Fax: (336) 716-0834
chcampbe@wakehealth.edu

Contact Susan Hensley or Lisa Keasler with questions and/or comments pertaining to ECG acquisition and transmission as well as hardware malfunction.

Appendix B
MAC 1200 PROGRAMMING AND SETUP

In order to setup a MAC1200 for the SPRINT study, turn the ECG machine ON. After the self-test completes, the ECG machine will be at the 12-lead screen (3 flat lines). Press the "Setup" key. Press "Enter" to select either 12-lead setup, system setup, communication setup or participant data setup. To make a selection, use the four arrow keys to highlight any selection and press "Enter".

12-Lead Setup

CATEGORY	SELECTION
REPORT SEQUENCE	[STANDARD]
RHYTHM LEADS	[II]
GAIN	[10]
REPORT FORMAT	[4x2.5R1]
DETAILED RESULTS	[NO]
MUSCLE FILTER	[NO]
MUSCLE FILTER FREQUENCY	[40 Hz]
AC FILTER	[YES]
MANUAL COPY TO	[HOST]
NO. OF COPIES	[1]
DELETE ECG AFTER TRANSMISSION	[NO]
AUTOSAVE ECG	[YES]
USE SCREENING CRITERIA	[NO]
SUPPRESS NORMAL STATEMENTS	[NO]
SUPPRESS ABNORMAL STATEMENTS	[NO]
INTERPRETATION	[YES]
PRINT INTERPRETATION	[YES]
OVERRIDE FUNCTION	[YES]

When finished, press the STOP key

Press the Down Arrow key to highlight System Setup, and press ENTER.
System Setup

CATEGORY	SELECTION
ORDERING PHYSICIAN	Name of the local clinic coordinator
REFERRING PHYSICIAN	Enter SPRINT and the 3-digit field clinic number.
TECHNICIAN	Choose OTHERS, press ENTER. Press ENTER until the cursor is under the LAST NAME; type the technician's LAST NAME then press ENTER. Type the technician's FIRST NAME then press ENTER. Press the Stop key.
INSTITUTION NAME	Type SPRINT plus name of institution
CART NUMBER	Enter your **3-digit clinic number**. Use this number for all SPRINT ECG machines at your location
SITE NUMBER	Enter **180** This is EPICARE's Study Number for SPRINT.
LOCATION NUMBER	Enter 1 for one of your ECG machines and 2 for the other ECG machine, etc...
DATE (mm/dd/yyyy)	Enter the correct date using the mm/dd/yyyy format.
TIME (hh:mm)	Enter the correct time in the hh:mm format.
LEAD FAIL BEEP	[NO]
HIGH HR BEEP	[NO]
LEAD LABELS	[AAMI]
PACE ENHANCEMENT	[NO]
BASELINE ROLL FILTER	[0.08]
DATE	[MM/DD/YYYY]
TIME	[24]
UNITS	[Cm, Kg]
MAINS	[60 Hz]
LCD LIGHT OFF AFTER	[5 MINS]
LOW BATTERY BEEP	[0 sec]
DEFAULT MODE	[12 LEAD]
LANGUAGE	[ENGLISH]
ENABLE PASSWORD	[NO]
TEST DATA	[NO]
RESTORE DEFAULTS	[NO]
PRINT SETUP LISTS	[NO]

When finished, press the STOP key. Press the Down Arrow key to highlight Communication, and press ENTER.

Communication Setup

CATEGORY	SELECTION
BAUD RATE (PC)	[9600]
PROTOCOL	[CSI]
MODEM	MultiTech 56k
DIAL MODE	TONE
PHONE NO.	• Enter 336-713-1218 if your clinic number ends with an even number **OR** enter 336-713-1219 if your clinic number ends with an odd number<br>• If an Access Code is required to dial a long distance number, enter the Access Code and the transmission telephone number at EPICARE, the same way you would dial a long distance number from your institution (using your Access code), For example:<br>• If the Access Code is needed AFTER entering the transmission number, then enter: 13367131102,,,123456789 where 123456789 is the Access code<br>• If the Access Code is needed BEFORE entering the transmission number, then enter 123456789,,„13367131102 where 123456789 is the Access Code<br>• **Note:** Access codes are separated from EPICARE transmission telephone number by three commas (,,,). This allows the MAC1200 to pause before another telephone number is dialed.
OUTSIDE LINE	If you need a digit to obtain an outside line, like a “9” or and “8”, please enter that digit here. Otherwise, leave it blank.

When finished, press the STOP key

Press the Down Arrow key to highlight Patient Data Setup, and press ENTER.

Participant Data Setup

CATEGORY	SELECTION
NEW PATIENT	[YES]
PACEMAKER	[YES]
GENDER	[YES]
HEIGHT	[YES]
WEIGHT	[YES]
RACE	[YES]
SYSTOLIC BP	[NO]
DIASTOLIC BP	[NO]
ORDERING PHYSICIAN	[NO]
REFERRING PHYSICIAN	[YES]
TECHNICIAN	[YES]
PHONE NO.	[NO]
MEDICATION	[NO]
COMMENTS	[NO]
ID REQUIRED	[YES]
PATIENT ID LENGTH	8 (May change once the final ID structure is decided)
SECONDARY ID	[YES]
SECONDARY ID REQUIRED	[YES]
LAST NAME (Required)	[YES]
FIRST NAME (Required)	[YES]
LOCATION #	[NO]
ROOM #	[NO]
ORDER NUMBER	[NO]
EXTRA QUESTIONS	[Leave Blank]

When finished, press the STOP key. Press the STOP key once again to exit the Setup menu. The Option Code Setup requires NO action.

Appendix C

Transmission of SPRINT study ECGs to the CERC

Before transmitting ECGs to the CERC

1. Ensure that all previously transmitted ECGs are deleted <u>only</u> after confirmation of receipt by the CERC.
2. Check to ensure that all IDs are valid.
3. You can correct any variable from your participant data information by doing the following:
    a. While holding the "Shift" key down, press the Store/Retrieve key,
    b. Move the cursor to the ID in question,
    c. Select ECG
    d. Press "Enter" to return to top screen
    e. Highlight "change/edit"
    f. Proceed to correct information

Transmitting ECGs to the CERC

1. Plug one end of the phone cable into the connector marked "LINE" on the rear of the modem and the other end into any "analog" (fax) phone line.
2. Start at the 12-lead screen.
3. While holding the "Shift" key down, press the "Store/Retrieve" key.
4. Use arrow keys to move the cursor to the ECG to be transmitted. While holding down uppercase key, use up or down arrow key to select more ECGs (Black box will appear at either side of a selected ECG). Repeat this process until all ECGs that are to be transmitted have been selected. Press the enter key to return to the top of the screen.
5. Select "Send" and press the enter key to start the transmission.
6. Once transmission is complete, press the "Start/Stop" key, located on the far bottom right of the keyboard, to return to the 12-lead screen.
7. Confirmation of receipt of transmitted ECGs could be made by logging into the SPRINT/EPICARE website using a user name and password specific to each clinic.

Appendix D

Full Header

Electrocardiogram

1. Time of Day _______:_______ ___A.M. _______/_______/_______
___P.M. Month day year

2. Has it been 8 or more hours since you last ate and/or drank anything other than water, including candy and chewing gum?
__Yes
__No

3. Results of examination
__ completed
__ not completed → a. Reason test incomplete or not done:
__ hardware malfunction (contact EPICARE @ 336-716-9616/0387)
__lack of supplies
__ insufficient time available or room not available
__ other, specify [ ]

4. Electrode location measurements (approximated to the nearest 0.5")
NV line [ ][ ].[ ]

Mid-chest [ ][ ].[ ]

5. Were any alert conditions noted?
__ Yes → Specify: [ ]
__ No
Action taken: [ ]

Technician ID: __________________

Appendix E

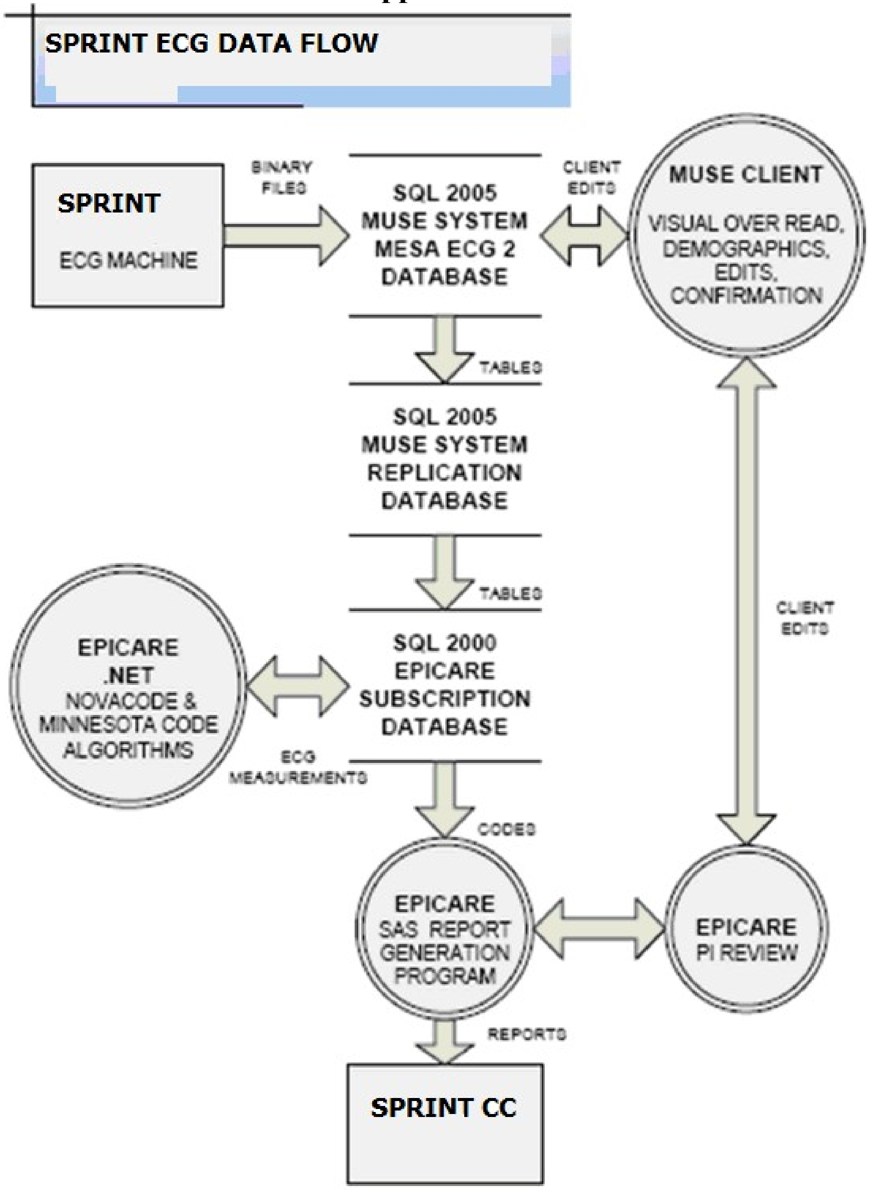

SPRINT ECG DATA FLOW
SPRINT
ECG MACHINE
BINARY FILES
SQL 2005 MUSE SYSTEM MESA ECG 2 DATABASE
CLIENT EDITS
MUSE CLIENT
VISUAL OVER READ, DEMOGRAPHICS, EDITS, CONFIRMATION
TABLES
SQL 2005 MUSE SYSTEM REPLICATION DATABASE
TABLES
CLIENT EDITS
EPICARE .NET
NOVACODE & MINNESOTA CODE ALGORITHMS
SQL 2000 EPICARE SUBSCRIPTION DATABASE
ECG MEASUREMENTS
CODES
EPICARE
SAS REPORT GENERATION PROGRAM
EPICARE
PI REVIEW
REPORTS
SPRINT CC

Chapter 9. SPRINT-MIND Brain MRI Study

Table of Contents

Page

1. MRI Reading Center Contact Information 1
2. Brain MRI study Introduction 1
3. Brain MRI study Overview 2
4. MRI Procedure Manual Overview.. 3
5. Test MRI Scan Procedures 8
6. Logging in a Participant for an MRI Scan 9
7. MRI Scan Procedures 10
8. MRI Scanning Protocol 10
9. MRI Protocol Parameters.. 11
10. Completion of Study Documentation 12
11. MRI Image Transfer—Field Center to MRI Reading Center.. 13
12. Completion of MRI Acquisition Form 14
13. Phantom Quality Control Quarterly Scans.. 15
14. Clinical Alert Procedure 18
15. Quality Control—MRI Reading Center to Field Center.. 19
16. Correspondence.. 19

Appendix 1. MRI Screening Form 20

Appendix 2. MRI Acquisition Form 22

Appendix 3. MRI Summary Form 23

Appendix 4. MRI Fact Sheet 24

Appendix 5. MRI Preparation/Instruction Form 25

Appendix 6. MRI Appointment Confirmation Fax Form 27

Appendix 7. SPRINT MRI Protocol (sequence order).. 28

Appendix 8. SPRINT MRI Sequence Positioning.. 29

Appendix 9. SPRINT Study Log 33

1. MRI Reading Center Contact Information

R. Nick Bryan MD, PhD
Principal Investigator
Phone: 215-662-3030
Email: nick.bryan@uphs.upenn.edu

Christos Davatzikos, PhD
Co- Principal Investigator
Phone: 215-349-8587
Email: Christos.davatzikos@uphs.upenn.edu

Elias R. Melhem, MD
Co- Principal Investigator
Phone: 215-662-6865
Email: elias.melhem@uphs.upenn.edu

Michel Bilello, MD
Co-Principal Investigator
Phone: 215-662-6865
Email: bilellom@uphs.upenn.edu

Lisa Desiderio, RT (R) (MR) CCRC
Project Manager
Phone: 215-662-6573
Fax: 215-662-7011
Email: lisa.desiderio@uphs.upenn.edu

2. Brain MRI Substudy Introduction

The SPRINT-MIND STUDY will include a brain MRI component as a part of the assessment of brain structure and function in the trial. The Brain MRI Substudy will be conducted at five participating field centers with a subset of 640 participants enrolled into the MRI study.

Two types of MRI studies will be obtained during the course of the substudy:

1) Test and QC Scans:
    - Before a field center can enroll subjects into this study protocol, a set of test scans on the ADNI and FBIRN phantoms and a volunteer using the SPRINT MRI protocol must be submitted for review and approved by the MRQC at the University of Pennsylvania Medical Center Philadelphia, Pennsylvania.

- QC scans performed on the ADNI and FBIRN phantoms will be on a quarterly basis and sent to the MRQC.

2) On-Study Participant Scan:
   - Performed at the protocol defined time-points per approved MRI protocol for data analysis.

Approximately 640 subjects will be enrolled into the SPRINT Brain MRI study from each of the field centers participating in the MRI component.

1. Boston University (Boston, Massachusetts)
2. Wake Forest University (Winston-Salem, North Carolina)
3. Case Western University (Cleveland, Ohio)
4. Vanderbilt University (Nashville, Tennessee)
5. University of Alabama at Birmingham (Birmingham, Alabama)
6. University of Miami (Miami, Florida)

MR imaging data will be transmitted from the **field centers** to the MRQC center at the University of Pennsylvania utilizing a designated, HIPAA compliant DICOM image transfer system (Section 11) where it will be analyzed and archived. The **MRQC** center will be responsible for the monitoring and compliance of the SPRINT Study participating **field centers**. The **MRQC** center will review the image data upon receipt from the participating **field centers** for acceptance or rejection of the MRI data into the trial. Additionally, one dicom copy of each participant scan will be stored at the **field center** for future access and audit. The MRQC center will centrally archive the data for permanent storage.

3. Brain MRI Substudy Overview

Overview of Field Center MRI Manual
Procedures for MRI scanning on the SPRINT study

This is an overview of the MRI manual that will be provided to each of the five SPRINT participating field centers. It will provide specific instructions for the following:

1. Performing MRI scans for the SPRINT study protocol.
2. Data Transmission of MRI imaging data to MRI Reading Center (**MRQC**).
3. Archiving and Correspondence of study data.

Introduction: machine specifications, personnel

Section 1- Test scans and field center training.
Section 2- MRI Exclusion /Inclusion criteria
Section 3- MRI Procedure

Section 4- MRI scanning protocol
Section 5- Archiving Instructions
Section 6- MRI Acquisition Forms
Section 7- Clinical Alerts
Section 8- Data transmission to the MRQC
Section 9- Correspondence from the field center to MRQC
Section 10- Quality Control and Communication from MRQC to field center

4. MRI Procedure Manual Overview

Section 1—Test scans

Before each **field center** is accepted into the trial, test scans need to be performed. This is often referred to as a "dry run." The procedure enables the **MRQC** to determine a number of issues:

- Scanner performance is within specification for entry into trial
- The **field center's** choice of scan sequence parameters is consistent with the SPRINT MR protocol.
- The **field center** is able to produce and transmit the SPRINT imaging data to the **MRQC** reading center at the University of Pennsylvania Medical Center.

To facilitate the objectives listed above, each **field center** is required to perform a test scan as listed **PRIOR** to any subjects being scanned as part of the trial.

After receiving the test scans, the **MRQC** center will communicate to the **field center** for confirmation of acceptance and quality of the scans.

Field Center Training
After the test scans and shortly after the SPRINT MRI study, and in addition to the central training at the Coordinating Center, the **MRQC** will report to each participating **field center** to instruct and train all personnel involved in the trial. The following training will be provided:

1. Overview of the ADNI and FBIRN requirements for scanner performance and quality control
2. How to register participant identification and preparation prior to Brain MRI Substudy.
3. MRI procedure
   a. Test scan
      i. Vendor equipment and software platform
      ii. Scanner performance
   b. Patient positioning
   c. MRI protocol
   d. Archiving

e. Transfer of image data from MRI scanner to the ACRIN/TRIAD image transmission system.

4. Transmission of MR imaging data from the American College of Radiology Imaging Network (ACRIN) **TRIAD** system to the University of Pennsylvania
5. Quality Control and correspondence of study data and related information from the MRQC to the field center. (MRI protocol compliance/acceptance, Clinical Alerts)

Section 2—MRI Exclusion/Inclusion criteria

MRI Exclusion criteria – Participants with a known contraindication to an MRI examination will be excluded from the study. (severe claustrophobia, pacemaker, defibrillator, neuro-stimulator, ferro-magnetic or unknown aneurysm clip, 3T MR incompatible metal implant of any kind or potentially dangerous foreign metal objects in the body such as bullets, shrapnel, metal slivers, etc.). Any female participant of childbearing age who has not tested negative on a pregnancy test prior to the scheduled MRI exam will also be excluded from participation in the Brain MRI Substudy.

MRI Inclusion criteria – A qualified participant from the **SPRINT** trial that *does not* have any of the above MRI contra-indications.

Refer to Appendix 1—MRI Screening Form.

Section 3—MRI Procedure

3A. Participant preparation and Instructions

The participant will have been oriented to the MRI procedure at the SPRINT clinic. As a follow-up at the MRI field center, the assigned MRI technologist will explain the procedure to the participant **prior** to the study and answer any questions. The participant will be screened for MRI contra-indications and instructed to remove all metal objects. The technologist will explain to the participant that the exam should take approximately 1 hour for completion. The participant should be told that movement, and/or speaking during the exam could cause the images to be less than optimal, and to refrain from such activity during the scan. They will also be told there is a button they can press to contact the technologist in case they want to stop the protocol.

3B. Participant Positioning

Subject comfort during the scan is important. Adequate padding should be used for patient comfort and immobilization. Reducing motion artifact is important for scan quality. Use of standard *Velcro* head straps, wedges, and earplugs is recommended.

A radiofrequency (RF) coil that has performed satisfactorily on the test scans sent to the **MRQC** center for review and approval will be utilized for the participant scans.

It is important that the position of the participant and orientation of the MR images are as defined by the MRI protocol in order to optimize visualization of the anatomical structures.

3C. Participant Identification

For purposes of data transmission, archiving, retrieval, and participant confidentiality, the **SPRINT** institution number and participant ID will be used for identification. The MRQC staff will instruct the **field center** personnel at the training visit.

Section 4—MRI scanning protocol

The MRI scanning protocol must be rigorously followed. The study requires one MRI scan for each subject participating in this trial. An MRI technologist at each **field center** should be assigned to this study to facilitate MRI protocol compliance, technical issues, and communication. If the **field center** needs to revise the protocol due to modifications in personnel, equipment or software, the revised protocol must be approved by the **MRQC** center prior to scanning a SPRINT participant.

Scanning pulse sequences-should be performed in the following order:

Series 1 - 3 plane Gradient echo localizer for positioning.

Series 2 - Sagittal 3D T1-weighted sequence for entire brain coverage.

Series 3 - Sagittal 3D FLAIR images from matching slice positions in Series 2.

Series 4 - Sagittal 3D T2-weighted images matching slice positions in Series 2.

Series 5 - Axial DTI (Diffusion Tensor Imaging) sequence.

Series 6 - Axial BOLD (Blood Oxygen Level Dependent) FMRI sequence (resting)

Series 7 - Axial ASL (Arterial Spin Labeling) perfusion sequence.

Series 8 - Axial ASL (Arterial Spin Labeling) calibration sequence.

Series 9 - Axial BOLD (Blood Oxygen Level Dependent) FMRI sequence (16 second breath-hold)

The MRI protocol will be provided in its entirety in the field center MRI manual. Included will be patient positioning methods, scanning parameters and vendor specific techniques. The **MRQC** staff will review all MRI test scans from each participating **field center** prior to the start of the SPRINT participant recruitment. Priority will be given to the sequences in the above order in case a participant cannot complete the whole protocol.

Section 5—Archiving Instructions

1. Download the MRI (archive) data to archiving medium specific to your facility. (CD-Rom, PACS).
    - **Only 1 copy of the archiving medium will be needed for storage at the field center for future access and audit. The MRQC center will centrally archive the data for permanent storage.**

Section 6—MRI Acquisition Form

The MRI technologist should review all scans for technical issues, protocol compliance and patient motion. If a series needs to be repeated, it should be done before the participant is removed from the scanner to avoid bringing the participant back for a repeat study. The MRI Acquisition Form will be provided in the field center MRI manual. This form is to be filled out by the **field center** for each participant's MRI visit. The form is then sent electronically to the MRQC for review. The following information will be entered:

1. Study, institution identification and participant identification number
2. Name of institution
3. MRI technologist/radiologist
4. MRI scan date
5. Clinical Alert findings
6. Date of Transmission
7. Additional comments (patient, protocol and technical issues)

Section 7—Clinical Alerts

If a clinically significant finding is identified after review by the assigned MR technologist and/or radiologist at the **field center or MRQC** personnel, the participant will be notified according to local field center protocol for clinical alerts. The **field center** is not responsible for diagnosis and management of its participants. The MRI study performed for the SPRINT trial is not intended to be used for clinical purposes. However, the field center radiologist does have an obligation to refer any **alerts** to their local FC for follow-up medical care. This procedure will be based on the urgency of the MR abnormality. An "**Alert**" is defined as an immediate or urgent referral.

<u>ALERT Referral Procedure</u>

Category 1- **No participant referral required-** Normal study.

Category 2- **No participant referral required**- No clinically significant changes. (old infarcts, white matter ischemic changes, atrophy, other chronic abnormalities)

***Category 3*- Urgent referral (Alert) -** Finding possibly having clinical significance (tumor without significant mass effect, sub-acute infarct, AVM, aneurysm, obstructive hydrocephalus).

***Category 4*- Immediate referral (Alert) -** Finding possibly having immediate impact on patient care (acute subdural or epidural hematoma, sub-arachnoid hemorrhage, acute intra-parenchymal hematoma, acute infarct, abscess and suspected tumor with significant mass effect.

Section 8—Data transmission to the MRQC Center

The American College of Radiology Imaging Network Group (**ACRIN**) will be providing all the necessary hardware and software for image transmission at each participating **field center** prior to the start of the Brain MRI Substudy. The transmission of imaging data will be performed utilizing the **TRIAD** dicom image transfer software from the MRI facility to the ACRIN PC with subsequent encrypted transfer to the permanent archive via the Web. The ACRIN staff will provide installation and training to the **field center** personnel prior to the start of the test scan and the start of the Brain MRI Substudy. The **MRQC** staff will report to each **field center** to train and instruct the **field center** personnel on the entire MRI protocol, including the transfer of the image data to the **MRQC** at the University of Pennsylvania. Participant's MR imaging data should be transmitted from the **field centers** to the **MRQC** within 24 hours of the scan performance. Data management and transfer training will involve the following:

1. DICOM Transfer of imaging data from MRI scanner to designated ACRIN/TRIAD workstation.
2. Patient Re-Identification, including case number, study number, and Institution ID at the ACRIN/TRIAD workstation.
3. Schedule transmission of MRI imaging series from the ACRIN/TRIAD workstation to the MRQC and any other approved SPRINT site destination.

Section 9—Correspondence to MRQC Center

All correspondence between the **field center** and the **MRQC** Center will be electronically filed and stored by the assigned research coordinator. The manuals will be provided to participating **field centers** involved in the substudy.

Section 10—Quality Control and Communication from MRQC Center to Field Centers

The **MRQC** center will be responsible for the monitoring and compliance of the Brain MRI Substudy participating **field centers**. The **MRQC** center will review the image data within two weeks after receipt from the participating **field centers**. Any additional information needed or comments regarding the MRI data will be included in the form.

MRI protocol compliance and Scanner Performance Quality Control/Assurance will be evaluated prior to recruitment. A volunteer test scan will be performed utilizing the **SPRINT** MRI protocol specific to the vendor/scanner platform. The ADNI and FBIRN phantom test scans will also be performed to measure scanner stability and image distortion. The test scans need to be completed by the **field center** and reviewed for site approval by the **MRQC**. In addition, the ADNI and FBIRN test scans will be performed on a quarterly basis by the field center and sent to the MRQC to assure scanner performance during the study time period.

Test scans
The MRQC will review the test scans for protocol compliance, technical issues and scanner performance. Any changes or modifications made to the scanner, hardware, or software at the participating field centers will require repeat test scans for QC evaluation by the MRQC before any further participants enter into the substudy.

A MRQC Center contact list has been provided (Section 1) for any questions or concerns regarding the SPRINT-MIND Brain MRI study.

5. Test MRI Scan Procedures

MRI PERFORMANCE ACCEPTANCE
Before each field center is accepted into the study, a test scan needs to be performed. This procedure enables the MRQC Center to determine a number of issues:

1. Scanner performance is within specification for entry into study.

2. The MRI site's choice of scan sequence parameters provides adequate tissue contrast for the purpose of the trial.

3. The MRI site is able to send the MRI data to the MRQC center via the dicom transfer mechanism that has been provided. (Section 11)

To facilitate the objectives listed above, each MRI site is required to perform a test scan as listed below ***PRIOR*** to acquiring a **SPRINT** participant MRI scan.

Further communication will be made after the MRQC center receives the test scans from the field center to confirm the acceptance and quality of the scans from your site.

Test scan instructions
To perform the **volunteer test scan**, please follow the instructions carefully, and in order.

1. The test scan procedure will be performed on a normal volunteer utilizing a 3 Tesla (3T) MRI scanner and an 8 or 12 channel Head Coil (Sections 7 and 8).

2. Enter your **SPRINT** site number and enter the name “SPRINT test scan” in the participant identification field.

3. Perform the scan using the sequence parameters given in the MRI protocol. (Sections 9)

4. Send the MRI images using the ACRIN.TRIAD dicom transfer. (Section 11)

ACRIN.TRIAD version 3.0 instruction manual can be found online at https://triad.acr.org/downloads.htm

To perform the **ADNI and FBIRN test scan**, please follow the instructions carefully, and in order:

1. The test scan procedure will be performed on the QC phantom scans utilizing a 3 Tesla (3T) MRI scanner.
2. Enter your TRIAD site number in (omitted ACR scan) the participant identification field when sending the phantom scans.
3. Perform the scan using the sequences given in the ADNI and FBIRN protocol. (Section 13)

6. Logging in a Participant for an MRI Scan

1. The subject information will be provided by the **field center** clinic coordinator and will be given to the MRI facility network radiologist/technologist. The following information will be provided:

   SPRINT Participant Identification number **
   Date of MRI scan **

2. The information provided will be used to complete the MRI Acquisition Form (Section 12), the identifier information on the MRI scan and MRI Study Log.

3. Transcribe the following information to the MRI Acquisition Form for the study.

   - SPRINT participant identification number **
   - Date of scan **
   - Date of Transmission
   - Scan Completion/Incompletion
   - Patient/Technical and other MRI scan related issues
   - Clinical Alert Findings
   - MRI Technologist Signature
   - Site Radiologist Signature

Proper participant scheduling and completion of the MRI participant information is the responsibility of the **SPRINT field center**. If there are scheduling problems or incomplete subject information, please contact the clinic coordinator at your site.

** Note: These are the only identifiers to be used for the substudy. For participant confidentiality, no protected health information (i.e. Medical Record Number, participant name, social security number, etc.) is to be used that identifies the participant other than the SPRINT participant identification number.

7. MRI Scan Procedures

General Introduction

This study requires the analysis of two MRI brain scans (baseline and 48 month) for participants participating in the SPRINT Study. Consistency is the key word in all aspects of this study. To assist with the adequate reproducibility and communication, an assigned MRI technologist is required at each field center.

It is important that the position and orientation of the MRI images are adequate for proper coverage of the participant's anatomy. Subject comfort during the scan is important. Adequate padding and neck support is recognized to be important, but no specific recommendations are made beyond the standard practice at your institution. Please provide ear plugs, eye masks, prism glasses or other items required for subject comfort as routinely used by your institution.

Minimizing motion artifact is important for scan quality. Use of standard practice of Velcro head straps and foam wedges is recommended. However, other methods of stabilizing the head which are routinely used at your institution are acceptable provided these methods were employed on the accepted test scan by the MRQC center at the University of Pennsylvania.

The radiofrequency head coil for the **SPRINT** Brain MRI Substudy must be approved by the **MRQC**, and found acceptable on the test scans sent to the **MRQC** at the University of Pennsylvania for review and approval and prior to recruitment.

8. MRI Scanning Protocol

For the main evaluation the following MRI sequences are needed:

1. 3 plane localizer
2. 3D T1-weighted - Sagittal scan plane
3. 3D T2 Fluid Attenuated Inversion Recovery (FLAIR) sequence - Sagittal scan plane
4. 3D T2-weighted Fast Spin Echo (FSE) sequence sequence - Sagittal scan plane
5. Diffusion Tensor Imaging (DTI) sequence - Axial scan plane (repeat sequence for total of 2 DTI scans)
6. BOLD (Blood Oxygen Level Dependent) sequence- Axial scan plane (resting state)
7. Arterial Spin Labeling (ASL) Perfusion perfusion sequence - Axial scan plane
8. Arterial Spin Labeling (ASL) Perfusion calibration sequence - Axial scan plane
9. BOLD (Blood Oxygen Level Dependent) sequence- Axial scan plane (16 second breath hold)

9. MRI Protocol Parameters

SPRINT 3T PROTOCOL:
8 or 12 Channel Head Coil

SEQUENCE ACQUISITIONS

1.	Plane **3plane**	Coil **12channel**	File name **Localizer (GRE)**
	Tr **20**	Te **5**	Fov **350mm (fov phase=100)**
	Thickness **10**	distance factor **20%**	Base Res **256** Phase res **50%**
	Matrix **144X192**	NSA **1**	Slices **18**
	Time: **25 sec**		
2.	Plane **Sagittal**	Coil **12channel**	File name **3D T1 MPRAGE**
	Tr **1900**	Te **2.89**	Fov **250mm**
	Thickness **1mm**	Slices **176 slices**	Base Res **255** Phase res **100%**
	Matrix **256X256**	NSA **1**	TI **900 ms** Pixel BW **170hz**
	Time: **4:26**	ETL=**1**	Flip= **9**
3.	Plane **Sagittal**	Coil **12channel**	File name **3D FLAIR**
	Tr **6000**	Te **160**	Fov **250mm (fov phase=85%)**
	Thickness **1**	Slices **160 slice**	Base Res **202** Phase res **91%**
	Matrix **258 X 221**	NSA **1**	TI=**2200 ms** Pixel BW **930**
	Time: **8:20**	ETL **203**	
4.	Plane **Sagittal**	Coil **12channel**	Psd File name **3DT2**
	Tr **3200**	Te **409**	Fov **250 (fov phase=80%)**
	Thickness **1**	Slices **176**	Base Res= **246** Phase res= **80%**
	Matrix **258x256**	NSA **1**	Center Freq. **water**
	Time: **4:08**	ETL **141** Flip **120**	Pixel BW **750**

Series 5 is repeated for a total of 2 DTI scans

5.	Plane **Axial**	Coil **12channel**	Psd File name **ep2d diff MDDW**
	TR **7300**	TE **84**	Fov **245**
	Thickness **2.2mm**	distance factor **0%**	Diff Directions- **33**
	Concatenations=**1**	number sl**=64**	Flip **90**
	Matrix **128x128**	NSA **1**	Center Freq. **water**
	Phase FOV= **100**	Pixel BW= **1860**	diff mode= Free
	Phase part fourier= **7/8**	Echo spacing= **.59**	diff weighting=1
	Accel factor= **3**	EPI factor= **112**	Base Res **112** Phase res **100%**
	Time= **4:37**		
6.	Plane **Axial**	Coil **12channel**	Psd File name **ep2d_bold_moco_rest**
	Tr **2000**	Te **25**	Fov **224 mm**
	Concatenations **1**	number sl **35**	Base Res **64**
	Thickness **3.5**	distance factor **0%**	Center Freq. **water**
	Matrix **64 x 64**	NSA **1**	Flip **75 measurements 120**
	Time: **4:06**	**EPI factor 64**	Phase res **100%**

Place pulse oximetry on patient finger for reading

7. Plane **Axial**
Tr **4000**
Concatenations **1**
Thickness **5**
Matrix **64 x 64**
Time: **5:32**
Coil **12channel**
Te **11** number sl **20**
distance factor **20%**
NSA **1**
Flip= **90**
Psd File name **pCASL**
Fov **220 mm**
Base Res **64** Phase res**=100%**
Center Freq. **water**
Fat suppression**=ON**
Echo spacing**= .47**

8. Plane **Axial**
Tr **150**
Concatenations **1**
Thickness **5**
Matrix **256 x 256**
Time: **1:04**
Coil **12channel**
Te **10** number sl **1**
distance factor **20%**
NSA **1**
Flip=**15**
Psd File name **fl_pc**
Fov **200 mm**
Base Res **256** Phase res=**100%**
Center Freq. **water**
Pixel BW **260**
Velocity**= 100cm/s**

9. Plane **Axial**
Tr **2000**
Concatenations **1**
Thickness **3.5**
Matrix **64 x 64**
Time: **3:36**
Coil **12channel**
Te **25**
number sl **35**
distance factor **0%**
NSA **1**
EPI factor =64
Psd File name **ep2d_bold_moco_bh**
Fov **224 mm**
Base Res **64**
Center Freq. **water**
Flip **75** **measurements 105**
Phase res **100%**

10. Completion of Study Documentation

MRI Study Log

1. Transcribe the following information to the MRI Study Log (Appendix 3) and keep in study binder for records.

    a. SPRINT Participant Identification Number **
    b. Date of scan **
    c. Date of transmission
    d. Technologist's signature

MRI Acquisition Form

2. Transcribe the following information to the MRI Acquisition Form for the SPRINT Study.

    - SPRINT Participant identification number **
    - Date of scan **
    - Date of Transmission
    - Scan Completion/Incompletion
    - Patient/Technical and other MRI scan related issues
    - Clinical Alert Findings

- MRI Technologist Signature
- Site Radiologist Signature

** Note: These are the only identifiers to be used for the study. For participant confidentiality, no other identifiers (i.e. Medical Record Number, Participant name, Social Security Number) are to be used that identifies the participant other than a SPRINT study participant.

11. MRI Image Transfer—Field Center to MRI Reading Center

The American College of Radiology Imaging Network Group (**ACRIN**) will be providing all the necessary hardware and software for image transmission at each participating **field center** prior to the start of the SPRINT-MIND Brain MRI study. The transmission of imaging data will be performed utilizing DICOM image transfer from the MRI to the ACRIN PC with subsequent encrypted transfer to the permanent archive via the Web. The ACRIN staff will provide an operator's manual and instruct the **field center** personnel prior to the start of the test scan and the start of the Brain MRI Substudy. The **MRQC** staff will report to each **field center** to train and instruct the **field center** personnel on the entire MRI protocol, including the transfer of the image data to the **MRQC** at the University of Pennsylvania. Participant's MR imaging data should be transmitted from the **field centers** to the **MRQC** within 3 business days from the initial MRI scan date. Data management and transfer training will involve the following:

1. DICOM transfer of imaging data from MRI scanner to designated ACRIN workstation.
2. Patient Re-Identification, including the **SPRINT** participant ID and Institution ID at the ACRIN workstation.
3. Schedule transmission of MRI imaging series from the ACRIN workstation to the **MRQC**.

For complete DICOM transfer instructions, please refer to TRIAD website address https://triad.acr.org/downloads.htm

TRANSFER OF MRI IMAGES

Once the images are stored on the ACRIN workstation, the images can be sent to the MRQC center using the ACRIN PC located at each field center.

MRI Transfer Instructions

1. Transfer the MRI images from the MRI scanner to the ACRIN PC workstation.

2. Connect to the ACRIN.TRIAD site server and highlight MRI study.

3. Highlight all the MRI series and select "send" at the top of the toolbar.

4. Enter the **SPRINT** Study information in the fields (**bold**) below:

Select Project-American College of Radiology #6
Select Group-ACRIN Image ARCHIVE
Select Site #________
Submission type- Clinical Trial
Study ID –SPRINT
Subject ID-SPRINT PPT ID
Time point-SPRINT
Time point description-SPRINT

5. Select the "submit" button. At this point, the study will begin the transfer process. A "**Complete**" message will appear once the study has transferred and will = successful transmission.

Additional information can be found on the TRIAD website under Learning Center-Rich Client.
https://triad.acr.org/pdf/ACR%20Triad%20Rich%20Client%20Users%20Guide.pdf

NOTE: The Brain MRI Substudy should take no more than 5 minutes to transfer. The study will be completely transferred after 100% arrives in the ftp transfer box that will be seen on the workstation as the study is transferring from the field center to the MRQC. If a problem is encountered, please contact the project manager of the MRQC center.
*(*Refer to Section 1 for MRQC contact information.)

6. When completed, close out of the ACRIN Dicom transfer on the workstation.

12. Completion of MRI Acquisition Form

The MRI Acquisition Form will be provided in the field center MRI manual. This form is to be filled out by the **field center** for each participant's MRI visit. The form is then sent electronically to the MRQC for review. The following information will be entered by the MR personnel:

a. Study, institution identification and participant identification number
b. Name of institution
c. MRI technologist/radiologist
d. MRI scan date
e. Clinical Alert findings
f. Date of Transmission
g. Additional comments (patient, protocol and technical issues)

13. Phantom Quality Control Quarterly Scans

Each participating field center will perform the quarterly phantom scans for the evaluation of scanner stability and image distortion using both the FBIRN (BOLD imaging sequence) and ADNI (3D imaging sequences) phantoms.

Phantom Setup and Positioning Accuracy:

1. The phantoms are placed in the volumetric head coil and detailed protocol instructions are provided in the manual. To ensure consistent measurements, it is important to place the phantom within the coil in the same position every time.

ADNI Phantom QC scan: The phantom scan should be performed on a quarterly basis for the SPRINT study. Please refer to the QC protocol below for positioning and MRI image sequences.

Position the phantom in the head coil as demonstrated below.

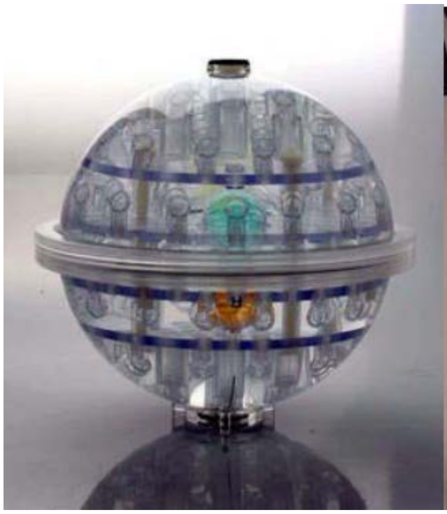

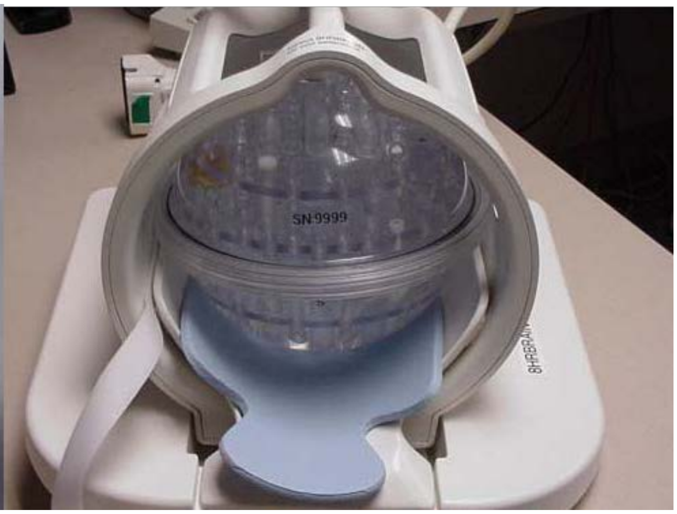

ADNI Phantom Scanning protocol for SPRINT 3D image sequences:

1. **3 plane localizer**- Assure phantom is positioned correctly in the head coil
2. **3D MP-RAGE T1 sequence**- The MPRAGE T1 phantom sequence will be acquired with the same MR image parameters as the SPRINT protocol except the slice thickness will be increased to 1.3mm for phantom coverage.
3. **3D T2 FLAIR sequence-** Follow the same parameters for the 3DT2 FLAIR SPRINT sequence except the slice thickness will be increased to 1.3mm for phantom coverage.
4. **3D T2 sequence-** Follow the same parameters for the 3DT2 SPRINT sequence except the slice thickness will be increased to 1.3mm for phantom coverage.

FBIRN Agar Phantom: The 17cm spherical agar-filled phantom will be performed on a quarterly basis to measure scanner stability during the fMRI BOLD SPRINT scanning sequence.
The phantom scan will consist of 200 separate image volumes captured over roughly a 10 minute interval.

FBIRN Agar Phantom Scanning protocol for SPRINT FMRI (BOLD) sequence:

Image parameters:
Scan plane= Axial
TR= 2000ms
TE= 25 ms
Matrix= 64x64
FA= 75
FOV= 224 mm
BW= 2232Hz/Px
EPI factor= 64
Base Resolution= 64
Phase Resolution= 100%
NSA= 1
Number of slices= 35
Slice Thickness= 3.5mm
Distance factor= 0

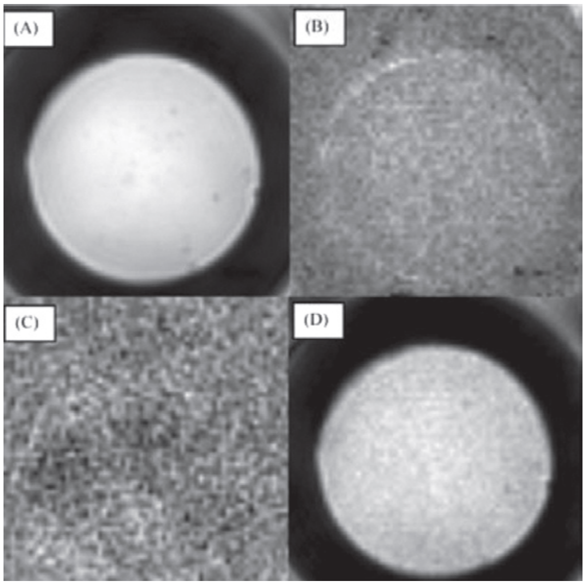

14. Clinical Alert Procedure

If a clinically significant finding is identified after review by the assigned technologist and/or radiologist at the **field center or MRQC** personnel, the participant will be notified according to local field center protocol for clinical alerts. The **field center** is not responsible for diagnosis and management of its participants. The MRI study performed for the SPRINT trial is not intended to be used for clinical purposes. However, the field center radiologist does have an obligation to refer any **alerts** to the FC principal investigator. It is the responsibility of the principal investigator to refer any alert to the participant's local source of medical care. This procedure will be based on the urgency of the MR abnormality. An "**Alert**" is defined as a non-urgent, urgent, and immediate referral.

ALERT Referral Procedure

***Category 1*- No participant referral required.**
No clinically significant findings.

***Category 2*- No participant referral required**- No clinically significant changes. (old infarcts, white matter ischemic changes, atrophy, other chronic abnormalities)

***Category 3*- Urgent referral (Alert) –** Finding possibly having clinical significance. (tumor without significant mass effect, sub-acute infarct, AVM, aneurysm, obstructive hydrocephalus.)

***Category 4*- Immediate referral (Alert) –** Finding possibly having immediate impact on patient care(acute subdural or epidural hematoma, sub-arachnoid hemorrhage, acute intra-parenchymal hematoma, acute infarct, abscess and suspected tumor with significant mass effect.)

In the event of a category 4 referral:
The field center radiologist and/or the MRQC will notify the proper **SPRINT** site personnel as defined by the SPRINT site principal investigator:

15. Quality Control—MRI Reading Center to Field Center

Subject scans
The **MRQC** center will be responsible for the monitoring and compliance of the SPRINT Brain MRI Substudy participating **field centers**. The **MRQC** center will review the image data upon receipt from the participating **field centers**. The **MRQC** will notify the **field center** and the **SPRINT** Coordinating Center for acceptance or rejection of the MRI data along with additional information needed or comments regarding of the MRI participant scans.

Test scans
The MRQC will review the test scans for protocol compliance, technical issues and scanner performance. Any changes or modifications made to the scanner, hardware, or software at the participating field centers will require repeat test scans for QC evaluation by the MRQC before any further participants enter into the SPRINT Brain MRI Substudy.

Quarterly QC checks
The MRQC will review the quarterly ADNI and FBIRN phantom scans for scanner stability and image distortion from each of the field centers

16. Correspondence

All correspondence between the **field center** and the **MRQC** Center should be filed and stored in the field center MRI manual. A copy of the MRI Acquisition Form (Appendix 2) can be printed out from the SPRINT Internal Website.

Please keep any other correspondence for the SPRINT-MIND BRAIN MRI STUDY in this section of the binder.

Appendix 1

SPRINT MRI INITIAL CONTACT SCREENING FORM (Printed from the SPRINT Website)

"I would like to ask if you'd be willing to have an MRI scan of your brain within the next month and again in 4 years. Unlike other scanning or imaging tests (x-rays, ultrasound, CAT scans), the MRI test provides the most clear and detailed pictures of the brain. It can help provide an understanding of brain changes in association with hypertension which is what we are investigating in the SPRINT-MIND study."

1. ***"If you are eligible, would you be willing to have an MRI?"***(Mark participant response)

   ☐ Yes (Go to #2)

   ☐ No (please go to #3))

2. ***"Do you have . . ."*** (Mark all that apply)

	Yes	No	
A.	☐	☐	Cardiac Pacemaker and/or Automatic Implantable Cardioverter Defibrillator?
B.	☐	☐	Cerebral aneurysm clip?
C.	☐	☐	Neurostimulator?
D.	☐	☐	Cochlear, otologic, or other ear implant?
E.	☐	☐	Exposure to metal fragments in or around the eyes? Or work with metal such as arc-welding, grinding, drilling metal, tool and die work?

"Or are you …." (Mark all that apply)

	Yes	No	
F.	☐	☐	Pregnant or at risk for pregnancy
G.	☐	☐	Severely claustrophobic (see Claustrophobia protocol)
H.	☐	☐	Other ________________

If <u>any</u> of A-H are marked **YES – STOP HERE**. State to the participant:
"Unfortunately we cannot ask you to participate in this portion of the SPRINT study. MRI scans are unsafe for persons who have any of these devices or conditions. We appreciate your willingness to participate and because of your participation in the SPRINT study, you have already made an important contribution."

If there are no absolute contraindications, review the SPRINT MRI Consent Form, answer any questions, and have the participant sign the consent.

GIVE THE PARTICIPANT THE MRI INFORMATION FORM WITH INSTRUCTIONS REGARDING HIS/HER APPOINTMENT, WHERE TO GO, AND PHONE NUMBERS TO CALL WITH QUESTIONS.

Date of Scheduled MRI: ______/_____/_______ Time of Scheduled MRI: ______:_______ am/pm

3. ***If the participant is not willing, please indicate the reason for refusal.***

A. ☐ too busy to participate?
B. ☐ not interested in participating?
C. ☐ fearful of the MRI procedure?
D. ☐ transportation issues?
E. ☐other reason? (Please specify): ___________

CLAUSTROPHOBIA PROTOCOL – to be used only if participant expresses concern about being severely claustrophobic:

Some participants may state that they are claustrophobic. If "severe", the participant may be ineligible for participation in the SPRINT MRI study. At the time of screening, if the participant expresses claustrophobia, (and the screening is completed in person), the Study Coordinator should show the participant what the MRI scanner looks like and provide further description of the procedure:

"During an MRI scan, you lie on a table and the table slowly slides into a cylinder shaped unit. Some people with severe panic disorder or severe claustrophobia cannot go through with an MRI scan. If you think you can go through with the MRI scan, you are eligible to participate. However, you do not have to participate if you feel that your condition will not allow you to be placed inside of the scanner."

Appendix 2
MRI Acquisition Form (Printed from SPRINT Website)

1. Participant Name: ______________________________ Participant ID: ____________________

☐ SPRINT MRI Consent Received

2. Date of MRI scan: ____/____/____

3. Was the scan:
   - ☐ Completed → Go to #4
   - ☐ Attempted but not completed → Why?
     - ☐ Participant related issues → Describe
       - ☐ Claustrophobia
       - ☐ Difficulty Breathing
       - ☐ Pain/Discomfort
       - ☐ Other, specify ________________
     - ☐ MRI scan technical issues
   - ☐ Not attempted → Why?
     - ☐ Participant did not show up
     - ☐ Participant withdrew consent
     - ☐ Participant called to reschedule (refer to SPRINT Clinic Coordinator)
     - ☐ Failed eligibility criteria
     - ☐ Other, specify: ____________________

4. Were all MRI protocol imaging sequences obtained for the following components of the exam?

	No	Yes	If NO, reason:
3DT1	1☐	2☐	____________
3D FLAIR	1☐	2☐	____________
3DT2	1☐	2☐	____________
DTI	1☐	2☐	____________
DTI (repeat)	1☐	2☐	____________
Bold resting	1☐	2☐	____________
ASL	1☐	2☐	____________
ASL calibration	1☐	2☐	____________
Bold breath hold	1☐	2☐	____________
Breath hold compliant	1☐	2☐	____________

Comments: ____

5. Date of transmission of MRI scan: ____/____/____

Appendix 3
MRI Summary Report

Participant ID: ___________________ **Date of MRI Scan: ___/____/____**

Findings that Require Routine Reporting to the SPRINT PI (check one or more)

______Level 1 – Normal Brain MRI

______Level 2 – Non-urgent disease related findings (Check one or more):
_____Non-specific white matter disease
_____Mild-moderate cerebral atrophy
_____Other non-urgent findings ____________________________

______Level 3 – Non-urgent disease related findings (Check one or more):
_____Significant remote stroke
_____Severe white matter disease
_____Severe cerebral atrophy
_____Other non-urgent findings _____________________________

Level 4 Findings Require Timely Notification of SPRINT PI

______Level 4 – Urgent findings (Check one or more):
______Acute or subacute infarct
______Acute or chronic subdural hematoma
______Epidural hematoma
______Subarachnoid hemorrhage
______Aneurysm
______Arteriovenous malformation
______Obstructive hydrocephalus
______Cerebral tumor
______Cerebral abscess
______Other urgent findings _________________________________

Level 4 Referral Process:
For any Level 4 finding, email Lisa Desiderio at desiderio.lisa@uphs.upenn.edu

If this finding requires attention in the next 12 hours, contact the clinic coordinator at the phone number listed on the appointment confirmation form.

_________________________________, MD, Radiologist SPRINT Clinical Site
Signature

Appendix 4

SPRINT-MIND MRI

As people age, or in certain health conditions such as cardiovascular disease, and/or hypertension, changes in the white matter portions of the brain become visible. The white matter connects different sections of the brain in ways that are similar to how cables network computers. When we age, the connections become less reliable, and these changes may lead to dementia or other brain conditions. While we do not expect to see major changes in the MR images of SPRINT-MIND participants due to your young age, having a reference picture is useful to look at relative to future changes. Also, some people in the SPRINT-MIND age range may have early minor changes due to diabetes, atherosclerosis or other cardiovascular conditions that have occurred earlier than expected; we think this will be rare.

Why is it so important to have an MRI Test ('Magnetic Resonance Imaging') for the SPRINT-MIND Study?

Unlike other scanning or imaging test (x-rays, ultrasound, CAT scans), the MRI test provides the most clear and detailed pictures of the brain. It can help provide an understanding of brain changes in association with hypertension which is what we are investigating in the SPRINT-MIND study.

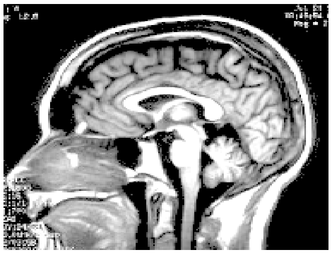

Features

- MRI provides a remarkably clear picture of the brain that can identify changes that might possibly explain declines in memory/mental function
- MRI can show differences between healthy brain tissue vs. unhealthy brain tissue, like areas of the brain that may have suffered from a stroke, etc.
- There is NO radiation in an MRI Test (unlike traditional x-rays) and little to no known side effects

Benefits

- Other doctors working on the study will review *your individual* MRI information
- There is NO cost to you for the MRI—normally an expensive procedure
- Your MRI information contributes to the medical knowledge and understanding of brain changes in association with cardiovascular disease

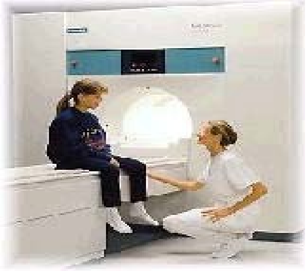

Appendix 5

Participant Preparation & Instruction Form
SPRINT MIND Magnetic Resonance Imaging (MRI)

Your MRI for the SPRINT Study is scheduled for:

Date: ______________________________ Time: ____________________________

<u>Where:</u> You will report to MRI Reception located ….

(Please add the information for your site)

<u>Contact Information:</u> If you have any questions regarding this appointment please call:

(Please add the information for your site)

<u>Purpose</u>:

MRI is a scanning technique that allows your doctor to see areas of your body without the use of x-rays. With a large magnet and radio frequencies, we can provide detailed images of internal body structures. MRI is considered an extremely safe procedure for most people, but because of the high magnetic field and radio frequencies there are some people who cannot have an MRI.

<u>Before You Arrive:</u>

Please plan on arriving 30 minutes before your scheduled MRI scan. If you do not arrive on time it is possible that we may need to reschedule your study for another day.

The total time of the MRI scan will be approximately 1 hour: 15-20 minutes preparing for the scan and approximately 45 minutes in the scanner. Please dress comfortably and remove ALL metal items including the following: jewelry, watches, hair clips, dentures, glasses, etc.

<u>When You Arrive:</u>

For your safety, you will be asked questions concerning implants and metals by technologist and/or physician. These questions are similar to the questions you were asked by the SPRINT coordinator.

You will also be asked to remove **ALL** jewelry, watches, hair clips, dentures, glasses and hearing aides. You will be asked to place all items including your credit cards and wallet into a locker. You may be asked to change into a patient gown. It is important to dress comfortably and avoid wearing any type of clothing and/or items that contain metal. You may be asked to remove belts, shoes, and other clothing items that may contain metal. It is easiest if you wear/bring as few of these items as possible.

Prior to the MRI scan, the technologist will provide you information which will include specific breath hold instructions (description included in this packet). You will also be offered an opportunity to use the restroom before you have the MRI scan.

Your Scan:

The MRI technologist will help you on the scan table and position you for the exam. The table will slide into a cylinder shaped unit. There is an intercom system so that you can hear and talk to the technologist who is operating the scanner. You will be given earplugs to wear to decrease the banging noise caused by the imaging process. When you hear a knocking or banging sound, you need to hold perfectly still. Please note that the earplugs do not block out all sound since we wish to stay in communication with you at all times. The MR operator will inform you of the progress of the study and prepare you for each sequence.

The scan is very sensitive to motion. If you move even a little during the scan, the scan may need to be repeated, so please do your best to hold still whenever the scanner is making noise. If you must move to make yourself comfortable, cough, or scratch your nose, please do so when the scanner is silent. At times you may feel like the magnet is shaking or vibrating. MRI is a safe imaging procedure, and these are all events that are commonly experienced while undergoing this type of MRI examination. If you feel uncomfortable or have a question regarding your experience, please communicate with the technologist.

When the study is complete, you will be moved out of the MRI machine. We ask that you please get up slowly since you will have been lying still for a relatively long period of time.

Appendix 6

SPRINT MRI Appointment Confirmation For MRI Center

Participant Name: ___________________________

Participant ID: _______________________

Appointment scheduled:

Date: ____________________

Time: ____________________

☐ **Participant has a signed MRI consent form on file at the clinic site.** **(A copy of the SPRINT MRI consent must accompany this appointment confirmation.)**

Special needs:

____

Clinic Contact (name and phone number):

____

For extremely urgent findings, please call the number listed above.

FAX TO MRI FACILITY TO CONFIRM APPOINTMENT

Appendix 7
SPRINT MRI Protocol (sequence order)

SPRINT 3T MRI SCAN PROTOCOL

Scanning Sequential Order (Measure)	Scan Time (1 hour timeslot-prep/scan)
1. 3Plane Gradient Echo Localizer	25 seconds
2. 3D Sagittal MPRAGE T1 (Morphological)	4:26
3. 3D Sagittal FLAIR (Morphological)	8:20
4. 3D Sagittal T2 (Morphological)	4:08
5. DTI 30 direction (anisotropy, mean diffusivity)	4:37
6. DTI 30 direction (anisotropy, mean diffusivity)	4:37 (repeat sequence-motion correction)
7. Resting BOLD (Functional Reactivity)	4:06
8. pCASL Axial(Arterial Spin Labeling/CBF)	5:32
9. pCASL Axial Calibration	1:04
10. Breath-hold BOLD (Vascular Reactivity-C02)	3:36
**Total scan time**	**41 minutes (40:51)**

SPRINT Protocol: Scanning Acquisition

Pulse Sequences (Structural):

1) **T1 Localizer**
2) **3DT1 MPRAGE** 5 minutes

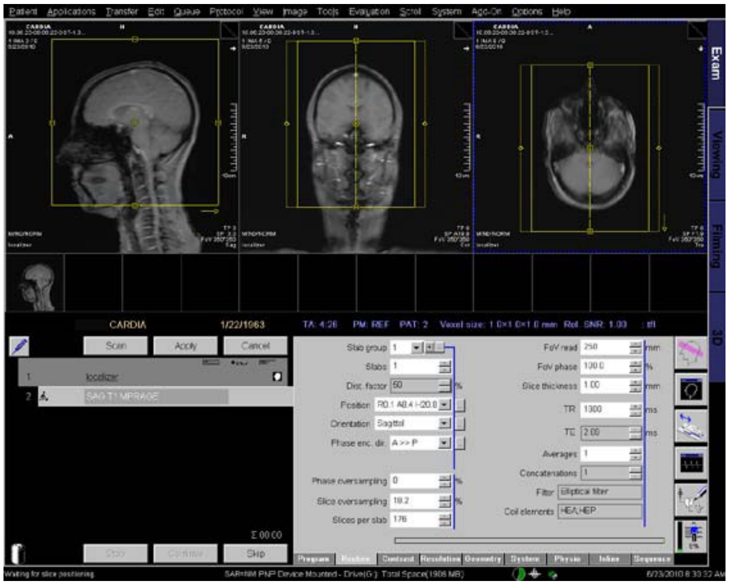

3) T2 FLAIR 8 minutes

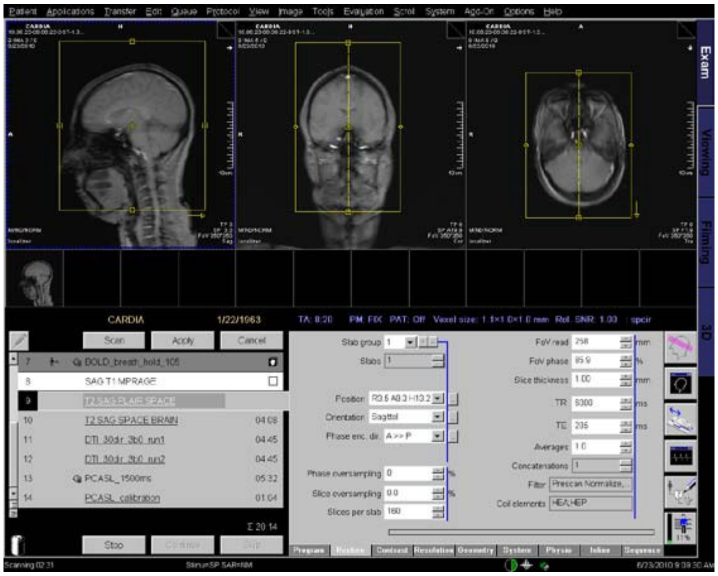

4) T2 SPACE 8 minutes

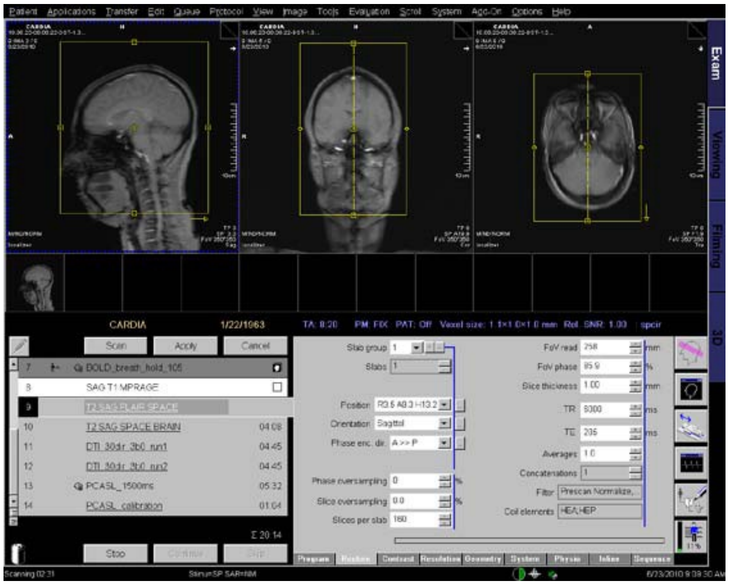

5) DTI 30 dir 5 minutes (x2)

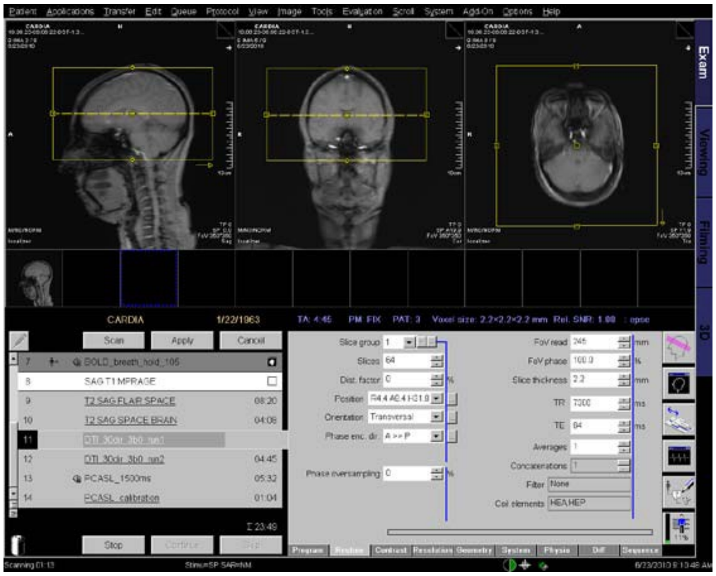

6) BOLD (resting) 4 minutes

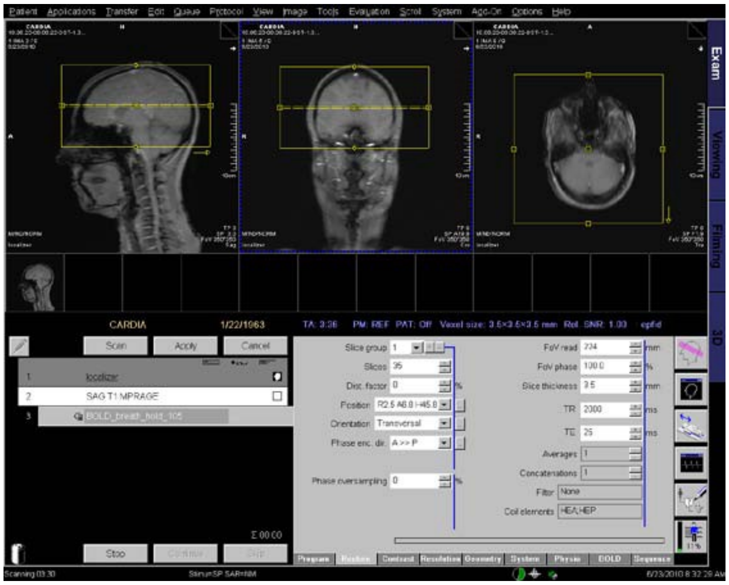

7) pseudo-continuous ASL (pCASL) 6 minutes

- Use Siemens pulse-oximetry on patient finger and ensure a stable reading

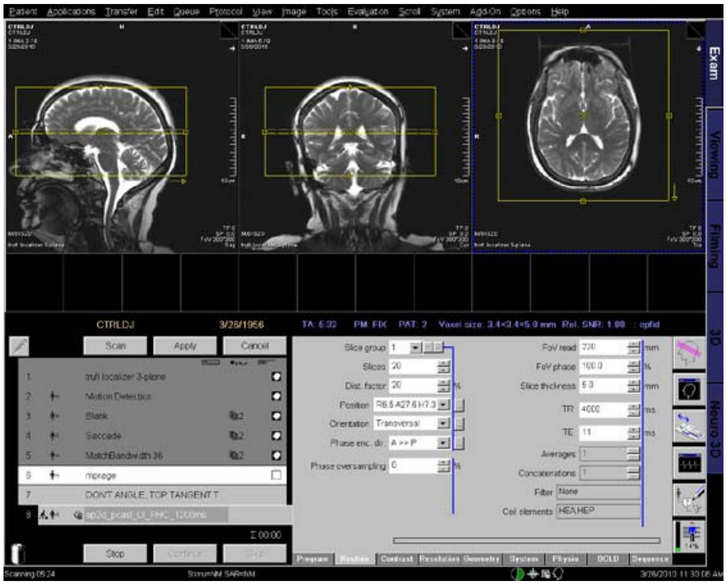

Note: Don't angle slices on the pCASL, BOLD, DTI imaging sequences (select transverse), the top slice should be tangent to the cortical vertex

8) Phase contrast MRI at the location of ICA and VA

45sec (depending on patient heart rate)

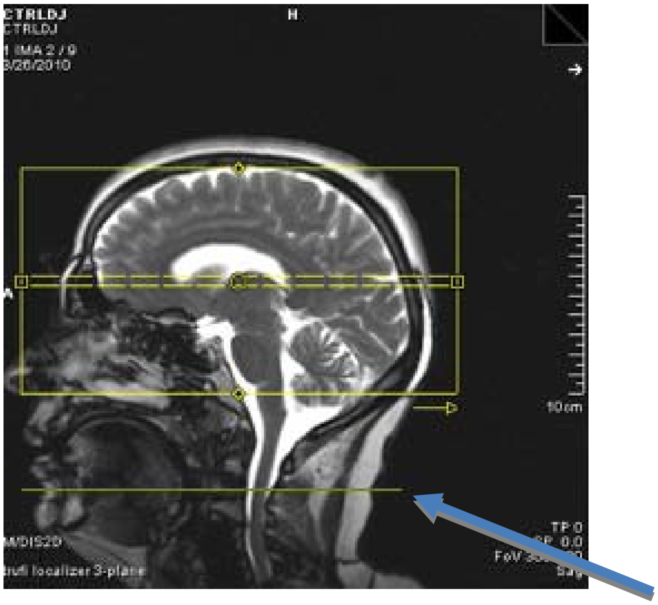

Position: Transversal plane through lower jaw and mouth orifice

9) BOLD (breath-hold) 4 minutes

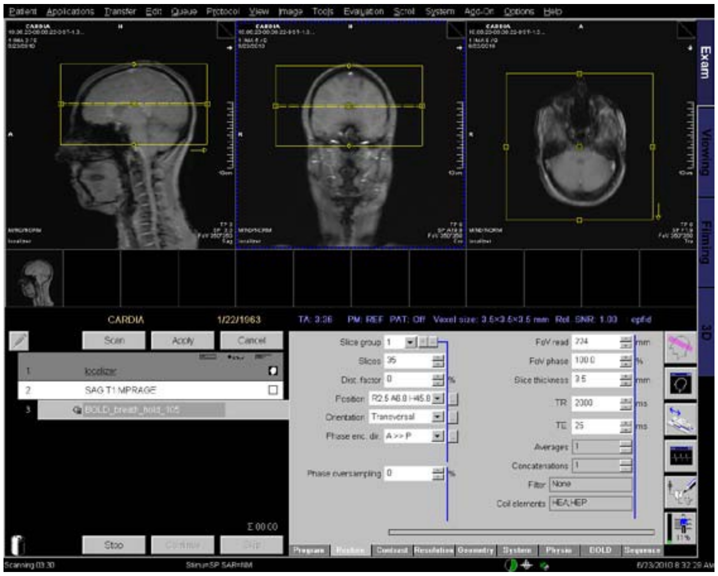

Appendix 9
MRI Study Log

MRI STUDY LOG

Subject ldenti fication	Date of scan	Date of transmission	MRI technologist

Chapter 10. Retention, Adherence and Participant Transfers

10.1 Overview

10.1.1 Background and Rationale. Adherence to study medications and dosing protocols is crucial to evaluating the effects of clinical trials, and therefore a major goal of SPRINT is implementation of strategies that maximize participant adherence to the intervention and study protocol for the duration of the trial. This manual chapter covers strategies for assessing risk of non-adherence prior to randomization, for monitoring and promoting adherence and retention during the study, and for aiding in drop-out recovery. For purposes of this chapter, a*dherence* refers to the degree to which a study participant follows the treatment protocol, e.g. study medications. *Retention* refers to continued involvement of study participants through the intervention and clinic follow-up phases of the protocol, including attendance at clinic visits, participation in any telephone contacts, medical assessments, procedures and assessments that are scheduled. A *dropout* is a study participant who is unwilling or unable to participate in treatment and/or follow-up assessments.

Maximizing adherence in a trial involves a multi-faceted strategy. Attention to adherence and retention ideally begins even before a participant is enrolled in the trial, during the screening process, and continues throughout the trial. Potential participants who cannot attend or complete study visits and/or take study medication as indicated should not be enrolled in SPRINT. During screening, individuals who demonstrate a significant risk for non-adherence to study medication or for completing study visits will be excluded from trial participation. Once participants are enrolled, a baseline assessment of adherence using standardized, valid adherence measures in conjunction with assessment of behavioral "red flags" by clinic staff allows for early identification of potential problems so that study resources can be devoted to improving adherence and retention for these individuals. Throughout the trial, ongoing monitoring of adherence is critical for identifying low adherence and enabling clinic staff to intervene and address problems as they develop.

10.1.2 Overview of SPRINT Approach. The overall adherence approach for SPRINT is based on two essential principles (Probstfield 1986). First, keys to good adherence and retention in a clinical trial are anticipation and a prevention-oriented approach. Second, effective adherence and retention plans for a clinical trial are implemented during the protocol development and recruitment periods, and revised during follow-up as needed.

In SPRINT, adherence monitoring and enhancement will reflect these broad principles, relying on previously used, validated tools as well as behavioral observations from clinic staff to proactively track and intervene when necessary to enhance adherence, and to ensure retention of study participants. To monitor adherence to medication, the 8-item Morisky adherence measure (Morisky et al, 2008; Krousel-Wood et al., 2009) will be administered to all participants at baseline, 12 and 48 month visits, and a single-item Visual Analogue Scale (VAS) will be used to screen for adherence at medication management visits (Giordano et al., 2004; Kalichmann et al., 2009; Walsh et al, 1998). In addition, a checklist of behavioral "red flags" will be provided to clinic staff to enable identification of participants who may be at risk for problems with adherence and/or

retention. Participants' responses on the adherence measures and/or observation of "red flags" by clinic staff will require specific actions to be taken by clinic staff – these will be outlined in this chapter, and described in the "Survival Kit", a standardized system for monitoring and improving adherence similar to one originally developed for the ALLHAT clinical trial (Lusk et al, 2004) and revised for use in the ACCORD trial.

As part of a central pretrial training session, all investigators and clinical coordinators will receive instruction on adherence and retention issues. This will include distribution of the "Survival Kit,"– a binder that contains information on common reasons for non-adherence, successful approaches to improving adherence, adherence monitoring tools and "checklists" for identifying participants who may have difficulty with adherence and retention during the trial, and adherence-enhancing materials and strategies that can be used as needed with participants demonstrating low adherence. Additionally, study staff will periodically have refresher and retraining instruction in the overall adherence program throughout the trial.

Also critical to maintaining good adherence and retention is the routine discussion of participants who show problems with adherence and brain-storming about problem-solving strategies during clinic team meetings and Study Coordinator meetings and conference calls. Of particular importance is the involvement of all members of the clinic team, including clinic leadership, in adherence-related monitoring and problem-solving. The key components of an overall adherence program are described in section 10.5.

10.2 Determining Adherence Potential

<u>10.2.1 Pre-randomization screening.</u> Risk factors for poor adherence and study drop-out will be assessed by study staff prior to participant enrollment in SPRINT and individuals judged to be at high risk of non-adherence and/or study drop-out will be excluded from the study.

The screening period is a critical time to identify potential barriers or "red flags" which might prevent a patient from adhering to the study protocol. Clinic staff should be alert to any situations that might suggest a patient is at risk for non-adherence, including:

- Patient's motivation to participate is not clear or patient is hesitant to participate
- Patient has expressed or unexpressed reservations about randomization (i.e., really wants assignment only to one or the other condition)
- Patient does not fully understand commitment (i.e. long duration of study, time and effort requirements)
- Missed visits during screening
- Adherence problems during screening (regarding assessments, failure to bring in requested medication bottles etc.)
- Lack of family support for participation
- Current family crisis or transition
- Frequent job changes
- Cultural, emotional, cognitive, or psychosocial issues that mitigate against protocol adherence (for example, beliefs or attitudes regarding medication use, depression, cognitive impairment, alcohol or drug abuse)

It is important to thoroughly assess factors which could influence a patient's ability to adhere to any aspect of the study, including treatment protocol, phone calls, study visits and medical or behavioral assessments and procedures. Some approaches that will help to identify potential adherence problems during recruitment include:

- Clarifying motivation for participation
- Fully exploring willingness to be randomized
- Fully exploring understanding of participation commitment
- Discussing missed screening visits in the context of continuing schedule of visits during study
- Discussing potential future barriers in order to ensure that there is nothing foreseeable that might interfere with long-term adherence
- Discussing family support for participation and any current or imminent family crises or transitions
- Discussing job situation in context of potential problems/transitions
- Discussing any cultural, emotional, or psychosocial issues that might facilitate or hamper participation

Careful screening prior to randomization will help to identify potential participants who are unlikely to complete the study or fully participate in the study protocol. If barriers are felt to be insurmountable by study coordinators or investigators, they have the discretion to not enroll the patient (please see exclusion criteria, chapter three of trial protocol).

10.2.2 Baseline screening. The use of these broad adherence-based exclusion criteria will screen out many individuals who would be at high risk for non-adherence and/or drop-out during SPRINT, but many participants who may have difficulty adhering to study medications and protocols will not be excluded. Therefore, all individuals enrolled in SPRINT will be administered the 8-item Morisky Medication Adherence Scale (MMAS-8) during the baseline visit to identify those who may have difficulty with the adherence requirements of the trial and who may be in need of additional attention during the trial.

Detailed instructions for administration and scoring of this measure are provided in Section 10.3 (Monitoring Adherence). Because not all participants enrolled in SPRINT will be on hypertensive medications at the time of enrollment, those who are on hypertensive medications will be asked to respond with respect to those medications. For participants with no other prescribed medications, these items will be skipped.

10.3 Monitoring Adherence

Identifying participants with low medication and protocol adherence is a challenge. Over recent years, research efforts have focused on the use and evaluation of methods for measuring adherence that are practical in the clinic setting. In SPRINT, two approaches will be used for monitoring adherence to medication and to the study protocol: (1) Self-report measures will be used to document adherence to medication at key study time points – at baseline, 12 and 48 months – and at medication management visits; and (2) A “checklist” of behavioral signs that may be indicative of adherence problems will be used by clinic staff to identify participants who may be having trouble with adherence or who may be at risk for dropping out of the study. Both of these monitoring approaches to adherence are discussed in detail below.

10.3.1 Self-report measures. Self-report adherence scales are considered relatively simple and economically feasible to use, and can have the added advantage of soliciting information regarding situational factors which may act as barriers to medication adherence (e.g., forgetfulness). For several of these self-report tools, high reliability and validity have been reported. One of the most commonly used measures of antihypertensive medication adherence is the 8-item Morisky Medication Adherence Scale (MMAS-8). This scale has been used as a self-administered questionnaire as well as through telephone administration. Items on this scale reflect reasons for non-adherent behavior (e.g., forgetfulness, health beliefs, and side-effects) and thus are useful in enabling study personnel to tailor their strategy for improving adherence to the underlying reason for non-adherence.

In addition to use of the MMAS, at every medication management visit, participants will be asked to complete a single item to screen for low adherence, called a Visual Analogue Scale (VAS) for medication adherence. To complete the VAS, participants are asked to mark a line at the point along a continuum showing how much of a drug/drug regimen they have taken in the past month. The response format uses a 100-point tool anchored at 0%, 50%, and 100%. The specific instructions read as follows, ‘‘Many people do not take their medications 100% of the time. On the line below, 0% means you have taken none of your blood pressure pills since your last visit as they were prescribed for you; 50% means you have taken half of your blood pressure pills as prescribed for you since your last visit; and 100% means you have taken every single dose of your blood pressure pills as prescribed for you since your last visit.

Participants indicate the percentage of medications taken by circling the percentage on the 100-point slide bar continuum.

1. Many people do not take their medications 100% of the time. On the line below, 0% means you have taken none of your blood pressure pills since your last visit as they were prescribed for you; 50% means you have taken half of your blood pressure pills as prescribed for you since your last visit; and 100% means you have taken every single dose of your blood pressure pills as prescribed for you since your last visit.

Please circle the percentage below that shows your best guess about how many days you have taken all of your blood pressure medications as prescribed since your last visit.

0% 10% 20% 30% 40% 50% 60% 70% 80% 90% 100%

In SPRINT, a response of 80% will be used to indicate a possible problem with adherence. If a participant’s response is 80% or less, or if the participant is not at the appropriate blood pressure target, study personnel will use materials and information provided in the Survival Kit to address the specific issues and barriers for each study participant that may be preventing

optimal adherence. In addition to use of the VAS, use of the MMAS-8 is encouraged at any study visit for any participant whose blood pressure does not achieve targeted levels at the discretion of the clinic staff.

10.3.2 Behavioral "red flags." The Survival Kit used in ALLHAT and ACCORD listed a number of "red flags" or behavioral and situational items that can alert clinic staff to a participant's potential problems with adherence and/or retention. The following are items that clinic staff should be aware of and use to identify potential adherence problems, along with possible actions that can be taken to address the problems. A fuller discussion of strategies for discussing adherence with patients and/or addressing adherence issues is contained in Section 10.4.

Participant Behavioral Changes From Previously Consistent Behavior

- ☐ Missed visits
- ☐ Difficulty in reaching by phone or failure to return calls
- ☐ Rescheduling twice or more for a clinic appointment
- ☐ Imminent major change in lifestyle (before next visit)
- ☐ Unexplained change in adherence to study medication

Actions to be taken: Engage the participant in a discussion of any possible problems being encountered with medication or protocol adherence. Listen carefully to any participant concerns or problems. If possible, tailor trial requirements so they will not be burdensome.

Participant Behavioral Signs

- ☐ Complaints about study clinic visits
- ☐ Impatience during study clinic visits
- ☐ Non-interactive demeanor during study clinic visits
- ☐ Unconcerned about adherence rate to study medication
- ☐ Participant expresses that they may stop study medication
- ☐ Sarcastic remarks about study medication
- ☐ Inappropriate humor about study issues, e.g., their own death or having a heart attack

Actions to be taken: Listen to participant complaints and determine if the problem is related to study participation. Address concerns promptly. These issues, particularly, may require a between visit phone follow-up.

Medical Signs

- ☐ Participant is hospitalized for any reason
- ☐ Participant experiences adverse event or illness
- ☐ Participant reports side effects from medication

Action to be taken: Talk to participant or family members about their illness and how it may relate to continued study participation. Address their concerns about the relation of study medication and whether or not it should be continued. Address concerns about side effects and revise the regimen if possible.

Clinic Environment Changes

- ☐ Reassignment to new primary care manager
- ☐ Reassignment to new clinic personnel for any procedures or tests
- ☐ Less frequent interaction between physicians and participants
- ☐ Delays in timely progression of clinic visits

Actions to be taken: Let the person know in advance about changes at the clinic. Introduce them to new personnel. Listen and address any concerns.

If a participant misses two consecutive visits, the clinic staff member should call their home. If they do not respond, a family member or friend should be contacted; if unsuccessful, a letter or postcard should be sent to their home with 'Address Service Requested.' If this occurs, the CCN Project Coordinator should be contacted. It is important for participant safety and the study integrity that every participant is followed in the trial.

10.4 Procedures for Maintaining/Improving Adherence

The following are recommendations, adapted from the ALLHAT and ACCORD Survival Kits, for promoting patient adherence and retention in all patients. These tips will be provided in the SPRINT Survival Kit, along with relevant materials, forms, checklists, postcards and other adherence-enhancing documents.

10.4.1 To encourage protocol adherence & retention:

¾ Provide patients with a copy of their study visit schedule, and include a list of procedures (i.e. blood tests, questionnaires, etc.) to be completed at each visit. It would be most helpful to provide this information in the form of a calendar, and to provide telephone or post card reminders to them 24-48 hours in advance of scheduled follow-up assessments.

¾ Participants who do not show up for an appointment should be called the same day to have the appointment rescheduled.

¾ Remind patients by phone or mail of upcoming visits one week ahead of the scheduled visit.

¾ If available, take advantage of automated calling service for reminding patients of scheduled SPRINT visits the day before their visit.

¾ Try to coordinate SPRINT visits with other upcoming clinic visits that the patient may have at your hospital.

¾ Where feasible, arrange visits in order to enable family or friends to travel together to clinic visits and, if acceptable to the patient, to "sit in" on at least part of the clinic visit. Be aware of and encourage the involvement of supportive others in the patient's study participation. If problems with adherence arise, it may be possible to explore with both the patient and support person the potential obstacles preventing adherence and to problem-solve ways to address the problems.

¾ Verify that the contact information for the patient and his/her closest family member (not living with patient) are current at each visit.

¾ Use incentives to reward adherence and retention. Incentives might include study-wide and/or site-specific lotteries, as well as a system of earning points toward a reward (e.g., T-shirt, mug).

¾ Develop a personal relationship with each participant by familiarizing yourself with the patient's life outside of SPRINT. Make notes about what they tell you, so that you may regularly inquire about their life.

¾ Arrange periodic "social get-togethers" with punch and appetizers, with the PI giving a talk on some aspect of health.

¾ Send out greeting cards to patients for birthdays and Christmas, and even get-well-soon cards (and/or even a nice house plant) in the event of a hospitalization or sympathy cards in the event of a death in the family. This small gesture is greatly appreciated.

¾ Annually recognize each patient's year anniversary of being in the SPRINT study by presenting them with a "SPRINT" certificate!

¾ To assist with patient retention and to maintain contact with and involvement of participants, newsletters will be written by the SPRINT team and provided biannually to all regional centers for distribution to their patients. Each newsletter will feature a SPRINT participant.

¾ Reimburse patients for transportation, parking and other logistical barriers, if the expense is a barrier to coming in for clinic visits.

¾ Provide a "next visit" tear-off appointment sheet to each patient for posting on their fridge.

¾ Recognize efforts with praise and encouragement. For example, have the PI present a sticker (i.e. "Super Patient!" "Great Job!" etc.) to patients when they achieve target BP levels.

¾ Introduce new participants to all SPRINT staff, even if they will primarily be seen by only 1-2 members. This will give them a sense of being a member of a team, and that they are being well cared for.

¾ Advise the patient in advance about how long their visit will be, and what kind of procedures will take place. This way, the patient can plan their day better, and know what to expect at their visit.

10.4.2 To encourage adherence to medication:

¾ Encourage the use of adherence aids such as daily or weekly pill organizers, calendars, alarms.

¾ When starting a new therapy, spend some time talking about the medication with the patient. For example, how the pill works, whether it needs to be taken with food, and when it should be taken. Patient monographs, like those given out at pharmacies, are often helpful for reinforcing information; the PDR or USP DI, as well as www.rxlist.com are helpful resources.

¾ When new medications are started or doses increased, make the patient aware of the possibility of side effects, and provide them with tips on how to either prevent or deal with these side effects.

¾ Inquire about the patient's pill adherence at different times of the day. Many people have no problem remembering to take their morning dose of pills, but forget to take their lunch or evening doses. Helping the patient identify cues to taking their medication or alternatively choosing a long-acting agent that can be dosed in the morning may enhance adherence.

¾ Provide the Medication Reconciliation Sheet to the patient at the end of this visit. This form provides a summary of all the medications the patient is taking at the end of each visit. This form will pre-print with all other study forms with the list of medications the participant was on at the beginning of the study visit. The form should be updated based on medication changes made during the current visit. Not only do these help the patient and their GP keep track of what they're taking, but a copy can be included in the chart as a quick reference for the study team.

¾ Encourage patients to take a rough inventory (i.e. list of part vs. full bottles) of their current stock of medications at home prior to coming in for their clinic visit. This will help avoid oversupplying or "weighing" the patient down with unnecessary medication at the end of the visit.

¾ For medications that are packaged in difficult-to-open containers, transfer pills into a more accessible pill vial.

¾ If possible, tailor the medication regimen, schedule, and dose to patient's preferences (e.g., some people have difficulty swallowing large pills, and therefore may be happier and more adherent if given smaller pills).

¾ Regularly emphasize the importance of taking their medication on schedule, every day, and that you will be inquiring about their medication-taking routine at each visit.

¾ Try to involve patients as "partners" in the decision-making process on therapy adjustments. This could be done by eliciting their feedback about what type of agent they prefer taking if several options exist.

¾ Help educate the patient about his/her hypertension in general. Identify a resource – e.g., one with short lay articles on hypertension and related issues. These can be photocopied and given to the patient at their visit.

¾ When calling to remind patients about an upcoming visit, especially a Medication Management visit, remind patients not to skip their morning or evening dose(s) of blood pressure lowering medication. By ensuring that the medication is "working" at the time of their visit, appropriate decisions can be made with regard to therapy adjustments.

¾ When adding new medication or increasing doses of existing therapy, initially caution patients to get up slowly from a sitting or lying position to avoid excessive dizziness or lightheadedness that may result from the extra blood pressure lowering effect.

¾ Explain to patients that even though they may not “feel” any different after taking their BP pills, the pills are, in fact, working as long as the pills are taken regularly.

¾ Remind patients to take diuretic pills in the morning vs. in the evening in order to avoid extra trips to the bathroom during the night.

¾ Encourage patients to take an active role in monitoring their BP progress. This may include recommending a home BP machine* for some patients or keeping a BP log from measurements taken at their pharmacy. By suggesting a source of feedback, greater adherence to therapy may be promoted. (*Many insurers will cover the cost of a home BP unit with a physician’s prescription.)

The following sections discuss strategies for addressing non-adherence in patients who are at risk for or are demonstrating problems with adherence.

<u>10.4.3 Addressing Low Adherence.</u> When you identify a non-adherent patient or one who may be at risk for non-adherence or for dropping out of the study (i.e., a patient who scores below a 6 on the MMAS, below 80% on the VAS; and/or who demonstrates one or more “red flags” from the behavioral checklist), your primary goal is to find out what factor or factors are interfering with the patient’s ability to adhere to the study requirements. Some of the monitoring tools (especially the MMAS) contain items that allow patients to indicate problem areas or barriers to good adherence (e.g., forgetting medications, taking a vacation, side effects) which can form the basis for further discussion between clinic staff and patient about those barriers and how to address them. Some of the possible barriers that may be encountered are as follows:

Logistical barriers – e.g., child care, transportation. For these barriers, investigate whether reimbursement is possible and make arrangements to relieve logistical or financial burdens where possible on study participants.

Time barriers, forgetfulness, schedule disruption – e.g., forgetting to take medications due to work or family responsibilities, or vacation. For these barriers, one approach is to assist the patient in planning a consistent medication-taking schedule. For example, have the patient choose a specific time or times of the day that is convenient and that involve behaviors that are consistent from day to day (e.g., brushing teeth in the morning and evening). Counsel the patient to take their medication at the specific identified time(s), and if possible, have them leave their medication container(s) near the area where the consistent daily task is performed, to serve as a cue for them to take the medication. If the patient plans a vacation in between study visits, counsel him or her to place the container in a place or with a note that will trigger medication taking at the usual time.

Once the patient has identified a plan for scheduling taking their medication at convenient times using reminders, notes, daily tasks and other “cues” to promote a consistent habit of medication-taking, the coordinator should ask the patient to imagine what kinds of things might come up that would interfere with their ability to stick to this plan. Coming up with solutions to potential barriers ahead of time may help to preempt adherence problems.

Motivational barriers – e.g., boredom, lack of energy, lack of interest, no clear reason stated. For these barriers, clinic staff will need to spend some additional time with the

patient. It is important not to appear rushed or impatient. It is also important not to try to convince the patient why they should comply. One useful strategy is to identify the patient's initial motivation to participate in the study. There are four main reasons why the patient might have initially agreed to participate in the trial:

- He/She perceived possible benefits from treatment
- His/Her spouse, significant other, family, doctor, etc. wanted him/her to participate
- He/She agreed for reasons other than personal benefit, such as contributing to science, pleasing the recruiting nurse, benefiting future patients, etc.
- He/She did not really understand what he/she was signing on to.

Reluctance may be due to the fact that the original reason is no longer compelling or valid. As such, an understanding of the reason for the current reluctance can be aided by determining why the patient initially agreed to participate. The recruiting case coordinator is an excellent source of information about this and should be consulted very early. In addition, it is a good idea to meet family members and discuss the goals and requirements of participation before beginning treatment. The patient was "sold" on the idea initially, and it may be possible to do so again by reminding him/her what he/she expected to gain.

When exploring barriers to adherence, it is useful to keep in mind that the patient's stated reasons are not necessarily the reason they have not been adherent. They may be unwilling or even unaware of the real reasons. It is important to understand the real reasons in order to know how to intervene. Taking a direct approach to the situation can help the patient to be direct with you. For example, "You were so interested in being in this trial initially, but I sense reluctance now. What accounts for that?" or "It seems like it has been hard for you to do the things we are asking of you. What would make it easier?"

<u>Motivational Enhancement</u>

If behavioral signs, patient self-report or other indications point to a lack of motivation on the part of the patient to adhere to the medication or other aspects of the protocol, or to continue involvement in the study, several strategies are available to re-engage the patient and improve his/her motivation to continue with the intervention and study protocol. These strategies are based on *motivational interviewing*, an approach to adherence assessment and intervention based on the Stages of Change model that is designed to identify and reinforce an individuals' personal self-motivating statements and reasons to change behavior. This approach to health-promotion interventions emphasizes the use of individualized risk appraisal, identification of potential risk-reduction strategies, techniques to increase self-efficacy for behavior change, and strategies to prevent relapse and promote retention. It incorporates several strategies to facilitate transition from one stage to the next, thereby preparing an individual to initiate and/or maintain a recommended behavior. Objective feedback is provided and ambivalence about behavior change explored, with specific attention to eliciting an individual's personal goals and self-motivational statements, formulating personal goals in behavioral terms and problem-solving barriers to change. Reflective listening skills are particularly effective as a method of interaction with patients in eliciting and clarifying their personal goals and self-motivational statements. Motivational interviewing seeks to

evaluate the discrepancy between participants' stated goals and their current behaviors in a style that increases motivation for change.

Motivational interviewing, as a formal method used to enhance adherence, requires training in the counseling techniques upon which it is based and thus is not recommended as a method for addressing adherence problems in SPRINT. However, several relatively simple ideas and strategies based on motivational interviewing can and have been effectively used to address motivational issues in clinical trials and clinical care settings. These methods, known as *motivational enhancement*, borrow from motivational interviewing but do not require the same degree of intensive training.
Several motivational enhancement "tips" are presented below:

Use reflective listening skills

- Use open-ended questions
- Reflect patient's statements and feelings without repeating back verbatim
- Elicit and reinforce self-motivating statements
- Elicit information about "red flags"

Develop discrepancy

- Change is motivated by a perceived discrepancy between present behavior and the patient's personal goals or values
- The patient rather than the clinician should present the arguments for change

Roll with resistance

- Ambivalence is normal
- Avoid arguing for change; acceptance facilitates change
- Do not directly oppose resistance; resistance is a signal to respond differently
- Invite but do not impose new perspectives
- Use the patient as the primary resource in findings answers and solutions to problems and barriers

Support self-efficacy

- The patient's belief in their ability to change is an important motivator
- The patient, not the clinician, is responsible for choosing and carrying out change
- The clinician's belief in the patient's ability to change can become a self-fulfilling prophecy

Use problem-solving skills to work toward solutions to adherence barriers

- Identify/clarify the problem
- Brainstorm potential solutions
- Weigh the pros and cons of potential solutions
- Select and implement the most attractive potential solution
- Evaluate solution and revise or replace if necessary

Elicit change talk

- Discuss disadvantages of the status quo.
- Discuss potential advantages of behavior change.
- Discuss patient's confidence and hope about his or her ability to change.
- Discuss patient's intention, desire, willingness, or commitment to change.

Approaches to Avoid

- **Arguing for change.** Taking up the pro-change side of ambivalence on a particular issue and seeking to persuade the patient to change.

- **Assuming the expert role.** Structuring the conversation in a way that communicates that you "have the answers". This includes the question-answer trap of asking many closed-ended questions, as well as lecturing the patient.

- **Criticizing, shaming, or blaming.** Shocking or jarring the patient into changing by instilling negative emotions about the status quo.

- **Labeling.** Proposing a specific label or diagnosis to characterize or explain the patient's behavior. Focusing on what the patient "is" or "has" rather than on what he or she does.

- **Being in a hurry.** Sometimes a perceived shortness of time causes the clinician to believe that clear, forceful tactics are called for in order to get through to patients. However, this often has the opposite effect if the patient feels pushed or rushed.

- **Claiming preeminence.** Using the "I know what is best for you" approach. Ignoring the patient's goals and perspectives is more likely to produce resistance than change.

10.4.4 Site specific and study-wide adherence monitoring and enhancement. Site PIs are expected to maintain their involvement with SPRINT patients as they move through the trial. In order to facilitate this, each site is expected to hold regular meetings among the PI, study coordinator, and other clinic staff during which the status of each patient's participation in the trial is reviewed. Each patient's level of adherence should be discussed and any problems identified should prompt intervention by the PI. In addition, the Network Coordinator and Principal Investigator should be alerted to the adherence problem. Real-time reports are available on the SPRINT web site to track adherence study-wide, at the site level, and at the individual participant-level. These data should be discussed at the site team meetings and a plan should be developed to deal with low adhering patients. Each site is expected to discuss adherence problems (along with steps taken to address the problems) to the Executive Committee for the purpose of receiving additional assistance as needed.

The Recruitment, Retention, and Adherence (RR&A) Subcommittee will meet by conference call on a periodic basis during the follow-up portion of the trial for the purpose of monitoring adherence performance. They will review data provided by the Coordinating Center that will be directed primarily at assessing adherence at the study and CCN level. The data reviewed will include the level of adherence to medication, the number of participants assigned to each level of intervention, the number of dropouts, the number of BP inactives, the number of participants lost-to-follow-up, and the number of participants who withdraw their consent. Adherence at the individual Clinical Site level will also be reviewed by the RR&A Subcommittee and will be reviewed by the respective CCN hub staff. Guidance to the individual CCNs will be provided as needed.

10.5 Components of an Overall Adherence Program

The specific techniques discussed in this chapter will be included in a Survival Kit that each site will have at their clinics, along with samples of letters and ongoing additional tools for the clinical sites and CCNs to use. Centralized training of techniques will occur for all SPRINT clinic staff. Additionally, to ensure adequate training of new staff and as a refresher for current staff, a "Training" page has been added to the SPRINT website. To access the Training page and its topics, click the Training tab on the SPRINT website. However, maintaining and improving participants' adherence and retention is dependent not just on the availability of strategies and materials for adherence management, but on the active participation and involvement of clinic, CCN and Coordinating Center staff in managing adherence and retention of all trial participants on a continual, proactive basis.

It is important to recognize the challenges involved in encouraging good adherence and retention in order to stay on top of problems even before they occur and especially in the early stages of adherence management. **Managing the adherence and retention of trial participants is one of the most difficult activities in clinical trials.** Since promoting adherence to the protocol and intervention regimen is difficult, it is fundamental that investigators and clinical staff maintain a position of equipoise -- a neutral position on the state of proof regarding the primary question in all the trial components and other outcomes being investigated in the trial. Since the questions being addressed by the trial are therefore worthy of investigation, investigators and staff can whole-heartedly support the study and promote participant adherence to the protocol.

Adherence and retention are issues in every clinical trial regardless of the type (prevention or treatment) and regardless of the type of measure used to evaluate it (visit attendance; dietary, medication or appliance intervention performance). Adherence lost is frequently difficult to regain. Therefore, prevention and early treatment of reduced adherence is imperative. Trial design allows us to make some adjustment for adherence that will predictably be lost. Adjustment for reductions in adherence is a squared function in estimating sample size calculations. Simply put, that means 80% adherence requires a 36% increase in sample size while 50% adherence requires a four-fold increase in sample size. Such serious consequences associated with reductions in adherence testify to the need for an overall adherence and retention management program. Key components of such a program include the following:

***Assure a Bottom Line of Minimum Retention* - We must know the primary outcome status of every participant at trial's end**. Some will choose not to participate in a trial after early commitment and randomization. However, at a minimum telephone ascertainment of the primary outcome status for every randomized participant at the end of the study will allow one to follow the principle of "once in, always counted" without any penalty, since outcome status would be known for all.

***Start Adherence Management during Screening and Recruitment* - Not all potential participants who meet eligibility criteria should be enrolled in the trial--think carefully before enrolling.** Every "number eligible screenee" should not necessarily be randomized. While physician and clinical staff have only moderate abilities to predict good adherence, some behaviors can serve as tips to possible poor adherence performance, as discussed earlier (e.g., multiple calls to accomplish a single

appointment). Common sense and input from all staff that have screenee contact should be useful (see “red flags” discussion, above).

***Intervene on Those with Threatened or Reduced Adherence* - Participants sometimes have trouble following the study protocol- listen carefully, be alert and be proactive!** Despite our best efforts at anticipation and prevention, some participants in clinical trials will be confronted with threatened or reduced adherence. Anticipation of those who are about to experience reduced adherence is very useful. The list of “red flags” (signs and symptoms of potential non-adherence) known to all staff can be used to identify behaviors which signal potential trouble. While simple preventive steps in managing a participant’s life events will frequently obviate adherence difficulties, occasionally reduced adherence to the intervention regimen or overall medication(s) regimen may be required. Direct involvement of the Principal Investigator or clinic physician in these adherence management activities may be particularly useful, especially if continued trial participation of the individual is threatened.

***Intervene on Those Who are not Completing the Protocol* - Don’t forget to “invite participants back” who have stopped visits or medication.** A special intervention plan is key for those who discontinue the intervention or even cease attendance in the study. Specific staff members who are trained in methods for dealing with these individuals are useful. Those participants with extreme non-adherence can be recovered and returned to productive performance in the study with a caring attitude, open negotiation, hearing the participants real concerns and a “time out” from trial participation with agreement to try again later (as necessary). Most dropouts occur early. A watchful approach to those newly randomized is crucial.

***Discourage Enrollment into Another Interventional Study* - The SPRINT protocol and MOP indicate that participants should not be recruited if already participating in another interventional study.** Once randomized into SPRINT, participants should be discouraged from joining another interventional study. If, despite this discouragement, a SPRINT participant enrolls in a second interventional study, it is the responsibility of the SPRINT investigators to determine whether continued active BP management by SPRINT is advisable. Regardless of whether active BP management is continued, the participant should be followed for outcomes per protocol. The following process should be followed.

- The site PI, CCN PI, CCN Medical Officer (if any), and the CCN’s Intervention representative need to review the situation and approve the request to continue active BP management in SPRINT.
- If there are concerns following this initial review, the case should be referred to the SPRINT Safety Officer who may elect to bring the case to the Steering Committee.
- Both the initial and, if needed, final reviews will consider the impact of the second intervention on the SPRINT intervention, as well as any safety concerns that may arise from the second interventional study.

***Intervene for Maintenance of Adherence in the Majority of the Cohort* - Many participants who do well with the study protocol will still require information periodically on SPRINT study progress and related information.** At least 50% of trial

participants will experience no adherence difficulties. Nonetheless, average adherence deteriorates for the whole cohort over time and is virtually impossible to maintain without some reduction. Motivation to continue the study is sometimes difficult and hard for staff to maintain among the participants and among themselves. Continuing educational efforts for trial participants about issues related to hypertension and the development of cardiovascular disease can be very useful. Some materials will be shared by the Coordinating Center with study staff, but be on the lookout for your own and share it with everyone. Examination of the reasons for continuing the study keeps fresh in everyone's mind the rationale for the investigation at hand.

Use Good Clinical Staff and an Adherence Team Approach **- Everyone on your carefully chosen staff who is in contact with the participant can help with adherence, including the PI--communicate with each other!** Having or hiring sensitive and personable clinical staff is crucial. Although any one individual's ability to predict adherence may be only moderate, the cumulative information of the clinic staff team is frequently useful in taking preventive or early therapeutic steps when reduced adherence is either threatened or has occurred. If possible, identify a primary staff member for each participant and use a constant care-taker model as much as possible. "Hand off," when possible, to another staff member openly and in the presence of the participant when a staff change is to be made. Set goals for those that are recovering their adherence to full levels and evaluate those goals regularly.

10.6 Transferring Participants to a Different SPRINT Site

During the course of the study, participants may leave the area in which they enrolled in the study and re-locate to another area where the study is taking place. To maximize participant retention, participants who re-locate from one study location to another should be encouraged to continue their study participation at their new location. To accomplish this, study staff at both the original site (called the "transferring" site) and the new site (called the "accepting" site) will complete the process of a participant transfer as detailed below. Please note that transferring "snowbirds" for certain periods of time in SPRINT is discouraged.

<u>SPRINT Participant Transfer Procedures</u>

1. The Clinic Coordinator notifies Clinical Center Network (CCN) Coordinator of the need for a participant transfer to another SPRINT clinical site. The CCN and site coordinators will discuss the participant's history of adherence to the visit schedule and protocol and their distance to the new clinic to ensure they are a good candidate for transfer (receiving sites retain approval/disapproval determination for transfers).
2. The CCN Coordinator notifies Coordinating Center (CC) of the need for a transfer. The CCN Coordinator and CC Staff will work together to identify an appropriate accepting clinic (e.g., geographically convenient for the participant, record of strong clinic performance, able to accept transfers).
3. For transfers within a CCN, the CCN Coordinator will contact the respective accepting site's coordinator to notify them of a potential participant transfer and verify that the potential accepting site remains appropriate and able to accept transfers. For transfers across CCNs, the CC will contact the respective accepting site's CCN Coordinator and Clinic Coordinator to notify them of a potential participant transfer and verify that the potential accepting

site remains appropriate and able to accept transfers. Upon confirmation, the CC will notify staff at both originating and accepting sites and CCN offices of the transfer arrangements, including the ID of the affected participant and contact information for all staff involved.

4. The transferring Clinic Coordinator:
   - Completes all relevant CRFs/data entry/data edits and event documentation (if indicated).
   - Notifies the respective CCN Coordinator that the data entry is complete for the participant and that all outstanding data edits and event and/or SAE documentation have been addressed and completed.
5. The transferring CCN Coordinator notifies the CC that data entry (and all related data procedures) is complete for the participant.
6. The CC then verifies that the data edits and expected SAE/event documentation are complete in the database at the CC.
7. The transferring Clinic Coordinator:
   - Contacts the accepting site with the participant's information (e.g., name, study ID number, treatment assignments, new contact information and other pertinent medical information) and with the participant's agreement arranges at least a <u>tentative</u> clinic appointment with the accepting clinic.
   - Gives the participant the accepting clinic's contact staff and information.
   - Assures participant has sufficient drug supply to carry them to the first appointment with the accepting clinic.
   - Sends paper copies of the participant's records to the accepting site. The participant's records will include all SPRINT forms for all study visits the participant has completed to date and any PRN forms completed to date.
   - Notifies the respective CCN Coordinator that paper copies of the participant's records have been sent (via a method that allows tracking receipt) to the accepting site.
   - The transferring site retains responsibility for participant follow-up (all protocol mandated visits) until the transfer is complete (seen and consented at accepting site).
8. The transferring CCN Coordinator notifies the CC that paper copies of the participant's records were sent to the accepting site.
9. The accepting site:
   - Confirms with the CC that the paper copies of the participant's records have been received.
   - Contacts the participant to verify (the tentatively arranged) or change the first clinic follow-up appointment.
   - Prints the next visit forms packet- without the participant ID. This will be done by printing the visit forms packet but not through the Participant Status Page. Blank forms can be printed from the Data Management Tab (Print Backup Forms) on the SPRINT website. The forms will not contain the pre-printed information for this visit, but the accepting site does have all the participant case report forms from previous visits that were sent from the transferring site.
10. Upon seeing the participant (clinic visit), the accepting site should <u>re-consent the participant before initiating any SPRINT activities.</u> Once data entry access has been granted for the accepting site, staff should review the layered consents entered at randomization and edit as indicated.
11. The accepting site confirms to the CC that the participant has been seen and notifies the CC of the re-consent date. This is very important; the accepting

site will not have data entry access for this participant until the site notifies the CC with the re-consent date.

12. The CC Project Manager will transfer the participant to the accepting site. After this has been completed, the transferring clinic will no longer be able to view the participant's data through the SPRINT website and the participant will not appear on the transferring site's reports. The accepting site will now have data entry access for this transferred participant.
13. The CC will notify all central units about the transfer including the Central Lab, DDC, Epicare and MRI Reading Center, and will copy both CCNs on those notifications.
14. The randomizing site will still maintain credit for the randomization. The accepting site becomes responsible for the participant and all related data and data edits collected at the (new) accepting site once the participant has been seen and reconsented at the accepting clinic. At this point the transfer will be considered complete.
15. If the participant does not consent to be seen at the accepting site, the transfer will be canceled and the originating site will retain responsibility for following the participant. Any paper copies of SPRINT forms sent to the accepting clinic should be returned in this event.

10.7 SPRINT Add/Drop Clinical Sites Study Policy

Whether or not randomized participants are involved, the addition or removal of a clinical site within a SPRINT Clinical Center Network (CCN) should be recognized as an event with possible implications for the entire trial. Thus, the impact of adding or removing a clinical site must be carefully considered. It is critical to balance the success of the overall trial and the contract oversight responsibilities of the Project Office with the autonomy of CCN operations.

The process for the decision to add or remove a clinic begins with due diligence on the part of the CCN to assess the criteria described below and to develop an internal consensus. The proposal can then be brought to the SPRINT Executive Committee, which will make a recommendation to the Project Office, where the final decision will be made.

<u>10.7.1 Adding Clinic Sites.</u> In considering whether a clinic should be added, a number of criteria should be addressed, including:

- The clinic's past performance in NIH and non-NIH clinical trials
    - Adherence to past protocols
    - PI's commitment to equipoise on the key SPRINT questions
    - Ability to recruit patients for SPRINT
    - Number of patients per SPRINT clinician (preference is for sites to have a limited number of clinicians who implement the SPRINT protocol)
    - Experience of trial personnel
- Collaborative experience with CCN members
- How the addition will affect the overall trial's racial and ethnic diversity, as well as the overall recruitment goals with respect to age, gender, and CKD status.
- How the addition will affect the overall trial's geographical distribution
- Cost impact on CCN budget
- Clinic and investigators' commitments to other research projects

- The CCN PI's assessment of whether the clinic will be effective throughout the entire course of the study.

10.7.2 Dropping Clinic Sites. The policy and process for removing a clinic is substantially affected by whether or not the clinic is following any randomized participants. If the clinic is following randomized participants, the ability to transfer those participants to another study clinic is a critical concern which can affect the integrity of the trial. In considering whether a clinic can be dropped, the same criteria as described above should be addressed; however, the specific circumstances leading to the decision should be highlighted.

Additional issues to consider prior to making the decision to close a site:

- Recruitment activity to date in SPRINT and projected capacity for future recruitment
- The CCN PI's assessment of clinic effectiveness throughout the entire course of the study, including intervention and retention record in SPRINT
- Specific circumstances leading to the decision

Close-Out Check List

The following steps should be completed, if applicable, and a site has screened or randomized at least one participant:

_____ Review screening status of all participants; i.e., determine where in the screening process all participants are

_____ Complete data entry on all participants

_____ Review all reports:

- No missing forms
- No outstanding blood samples
- No outstanding ECGs
- No outstanding queries

_____ With the help of the CCN, prepare a plan for transfer/follow-up of participants

- Is there currently another SPRINT site(s) nearby to which participants can be transferred? Refer to public website to search for nearby SPRINT site. Determine the number of participants the site is willing/able to receive
- If no sites are nearby, determine who will be responsible for follow-up of these participants
- CCN only: Consider incentivizing sites to accept transfer participants
- CCN only: Contact the Project Office to discuss redistribution of resources as need

_____ With the help of the CCN, prepare letter to be mailed to participant requesting permission to contact participants

- Letter should include:
    - o Description of plans for transfer/close-out
    - o Contact information for closing site as well as CCN
    - o Contact information for site IRB

- o Medical release form
- o Place for participant to indicate their selection (select site they choose to transfer to, request to be followed for outcomes only, request to discontinue participation in the study)

- Follow local CCN and clinic IRB requirements about submission of letter to IRB

_____ Transfer or plan for follow-up for (or loss of) each screened and randomized participant

- The process of closing down a site is an ACTIVE one; it is important that the participants continue feeling connected to the study
- Ensure that all participants have up to a 90 day drug supply, per local IRB requirements
- If participant(s) can be transferred to another SPRINT site: Follow participant transfer procedures (See Section 10.6)
- Randomized Participants ONLY: If participant cannot be transferred to another SPRINT site, determine whether they can be followed for outcomes only; determine which clinic will follow them for outcomes only
- If screened participants do not wish to transfer to another site OR if a randomized participant refuses to be followed for outcomes only:
  - o Close-out letter sent to participant (includes letter to the participant and letter to the participant's PCP describing SPRINT study and medications)
  - o Complete "Participant Status Log" indicating refusal
  - o Original forms must be retained at the CCN level; if this is not possible due to local IRB restrictions, the forms must be accessible to the study according to IRB and NIH regulations

The following steps should be completed, if applicable, regardless of whether a site has screened or randomized a participant:

_____ Proposal and plan to close site brought to the SPRINT Executive Committee as soon as possible; Project Office will make final decision about site close-out

_____ CCN PI drafts a site termination letter

- E-mail termination letter to Dave Reboussin, Brenda Craven, Larry Fine, and Joni Synder for review and approval of letter to send to site PI
- Upon approval by above, CCN PI e-mails Cheryl Jennings and Ali Amrana for approval of termination letter
- Letter signed by both CCN PI and others as deemed appropriate by the CCN
- Send letter to Site PI
- Provide copies of correspondence and final termination letter to all persons listed above

_____ Perform inventory of all remaining drug

- DDC will send file with all available bottles to be verified against physical inventory

- Work with CCN to determine where remaining supply will be shipped (another SPRINT clinic or CCN)
- Confirm inventory and notify DDC and CC of location where drugs will be shipped
- DDC will mark all remaining data with the new site's number
- If drug needs to be destroyed, follow instruction in the DDC MOP

_____ Work with the CCN to determine where remaining supply will be shipped

At time of termination (after all above steps are complete):

_____ CC notify central units to no longer send supplies to the closed site

_____ Return equipment as follows:

- Equipment returned to Coordinating Center
  - 2 barcode scanners
  - 2 OMRONs
  - 1 OMRON stand
  - MIND supplies
- Equipment returned to CCN
  - ECG and ECG supplies
  - Printer and supplies

_____ Site will notify IRB that they are closed

- Send IRB closure letter to CCN and CoC

_____ CCN notify CC of individuals who should have access to the website deactivated

_____ CC Data Systems Group actions

- Deactivate website access
- Update reports as required
  - Work with the CCN to determine proper actions for fee-for-service reports

References

Giordano TP, Guzman D, Clark R, Charlebois ED, Bangsberg DR. Measuring adherence to antiretroviral therapy in a diverse population using a visual analogue scale. *HIV Clin Trials*. 2004; 5:74-79.

Krousel-Wood et al, *The American Journal of Managed Care*, January, 2009, Vol 15 (1).

Kalichman SC, Amaral CM, Swetzes C, Jones M, Macy R, Kalichman MO, Cherry C. A Simple Single-Item Rating Scale to Measure Medication Adherence: Further Evidence for Convergent Validity. *Journal of the International Association of Physicians in AIDS Care*. 2009, 000(00) 1-8

Lusk CM, Bettencourt J, Carroll L, Egan D, Ford CE, Kingry C, Johnson J, Sullivan S, Nwachuku C, Probstfield JL. The Adherence Survival Kit. *Applied Clinical Trials*, October, 2004, Vol 13 (10).

Morisky DE, Ang A, Krousel-Wood M, Ward HJ. Predictive Validity of a Medication Adherence Measure in an Outpatient Setting. *J Clin Hypertens (Greenwich). 2008; 10:348–354.*

Probstfield JL, Russell ML, Henske JC, Reardon RL, Insull W Jr. Successful program for recovery of dropouts to a clinical trial. *American Journal of Medicine*, 1986; 80(5): 777-84.

Walsh JC, Dalton M, Gazzard BG. Adherence to combination antiretroviral therapy assessed by anonymous patient self-report. *AIDS*. 1998; 12(17):2361-2363

Chapter 11. Procedures for BP Inactive/Lost to Follow-up/Withdrawn Participants and Missed or Incomplete Visits

11.1 Overview

11.1.1 Drop-out Recovery. SPRINT will use a systematic approach in attempting to recover reluctant participants, i.e., those who have either expressed interest in dropping out of the study or appear to be likely to drop-out due to noncompliance with any aspect of the study. Many of these individuals may be inclined to drop-out due to life changes (e.g., divorce, illness of family members, changes in jobs, etc.) and/or from misunderstandings with study staff or from concerns about study procedures.

The goal of drop-out recovery is three-fold: 1) to ensure that participants are not pushed to the point that they refuse to participate further; 2) to continue to engage participants through some form of contact (e.g., phone, home visits) and allow an opportunity to determine participants' concerns and problem-solve for solutions to concerns and barriers to participation; and 3) to foster some form of continued participation (e.g., even an agreement to allow future contact). With time, it is hoped that participants can be moved to increased levels of adherence to the protocol.

The general approach to drop-out recovery will involve contact by the study coordinator in an attempt to: 1) identify barriers to participation, 2) problem-solve for solutions to overcome identified barriers, 3) apply motivational enhancement methods. With systematic efforts to recover these participants, many who initially express the desire to drop out can be recovered when either their life circumstances change so that it is more feasible for them to participate again or when their concerns with study staff and/or clinic procedures have been addressed. In situations where a participant's behavior suggests that he/she does not wish to participate, but does not give a hard withdrawal of consent, the above strategies should be implemented. However, attempts to re-integrate a participant should be discontinued as soon as a hard withdrawal of consent is communicated.

The Adherence Survival Kit will contain samples of letters that should be used to contact reluctant patients. In the early stages of non-adherence, a letter should be sent asking the patient to contact the study coordinator to discuss any problems or concerns they have regarding their participation in SPRINT. If this is unsuccessful, or if a patient specifically asks to be withdrawn from the study, a dropout letter is available. This letter acknowledges the patient's request, and asks the patient to agree to minimal follow-up to determine health status, without participating in the study protocol. Please see the Adherence Toolkit for copies of these letters that you can tailor for use with individual patients.

11.1.2 Definition of Participant Status. At baseline all SPRINT participants consent to having their blood pressure treatment managed by their SPRINT physician and clinic staff. Participant status is assessed at the end of each clinic visit. Appendix 11.A provides the Protocol Adherence Flowchart that can be used as a tool when determining the participant status. Appendix 11.B provides suggestions for increasing communication and activity for participants who are BP inactive or have the potential for becoming lost to follow-up or withdrawing consent. For purposes of the study, we define the following terms related to trial participation status:

- **BP Active** status – a SPRINT participant is considered to be BP active if his or her blood pressure medication therapy is managed according to the SPRINT study protocol.

There may be instances where a SPRINT physician is managing the participant's medications in accordance with the study protocol, but the participant is obtaining medications from a (non-study) pharmacy of his/her choosing; in such situations, the participant is considered to be BP active as well. If the SPRINT physician is no longer exclusively managing the participant's blood pressure, but is working in conjunction with the participant's PCP (or other non-study doctor) to manage the participant's blood pressure according to the SPRINT study protocol, this participant is BP active as well. Note that the SPRINT protocol allows providers the exercise of clinical judgment in therapy decisions for individual participants including temporarily or permanently backing off treatment goals in response to adverse events. In such cases, the participant is still considered BP active so long as their blood pressure therapy is managed by their SPRINT physician.

- **BP Inactive** status – a SPRINT participant is considered to be BP inactive in SPRINT if his or her blood pressure is no longer being managed by the study *and* he/she is not lost to follow-up or withdrawn. A BP inactive participant must meet both of these criteria. This may include participants who are being followed exclusively by phone, participants who have indicated that they can be followed for events only, or participants who agree to a final close-out visit at the end of the study.
- **Lost to follow-up** status – a SPRINT participant is considered to be lost to follow-up if the clinic has not collected data from the participant within the past 12 months and an alternative follow-up plan has not been established (e.g., end of study contact only). This may include participants that cannot be contacted by any ordinary means (e.g., home phone, cell phone, mail, email, fax, etc.), **and** alternative contacts do not know where the participant is or cannot be contacted themselves. A participant who steadfastly avoids contact is also considered to be lost to follow-up.
- **Withdrawn** status – a SPRINT participant is considered to be withdrawn if he/she has withdrawn consent to participate in the study and refuses further contact for any reason.

11.2 Lost to Follow-up/Withdrawal Retention Tenets within SPRINT

The SPRINT study goal regarding participants is to follow/collect as much data as possible on all randomized participants to the end of the trial. Sites should update contact information and medical records release (if necessary) at every study contact. Ensuring the SPRINT study has the most up-to-date information is critical. The Participant Baseline Contact Information should be printed and given to the participant to update.

11.2.1 Follow-up Contact Choices.

If a participant is having treatment or visit adherence problems, negotiate levels of commitment as follows.

Follow-up Contact Choices (in order of preference):

1. Clinic visit/active treatment
2. Home visit/active treatment*
3. Phone follow-up/active treatment (e.g. snow-birds)*
4. Clinic visit/inactive treatment
5. Home visit/inactive treatment*
6. Phone follow-up/inactive treatment*

*See MOP Chapter 3c for details about conducting home and phone visits

11.2.2 Frequency of contact.
In addition to contact type, frequency of contact should also be negotiated in the following manner:

a) On protocol schedule (e.g. every 3 months) (clinic preferred, or a combination of clinic, home and phone or phone alone) or if not acceptable;
b) Not on protocol schedule, perhaps one in person contact/year (annual clinic visit preferred) with phone follow-up in the interim or if not acceptable;
c) Phone contact once a year or if not acceptable;
d) Clinic (preferred), home or phone contact at the end of the study.

11.3 Procedures for Incomplete Data Collection and Missed Visits
In SPRINT, post-randomization visit schedules are based on target dates of contact as specified in the study Protocol. By design, follow-up contact for all participants falls into one of the following two categories: (1) monthly during the first three months of follow-up, and (2) every three months thereafter. For purposes of visit classification, the follow-up periods for each visit are divided into continuous visit windows.

In SPRINT, a **missed visit** occurs when a given visit window lapses without data collection having occurred. When a clinic visit has been missed, it should be documented as such by completing an ***Encounter and Disposition Form***. The visit is specified as missed by responding "No" to question 2 ("Was study data collected for this visit?"), and indicating in Part D, Missed Visits the reason that the visit was missed.

There may be situations when all data collection cannot be completed within a single clinic visit. In these situations, a visit may be split into two separate encounters or the data elements that were missed may be collected at a subsequent quarterly visit. Appendix 11.C provides the Missed Visit Flow Chart that can be used as a tool when completing a split visit. Appendix 11.D provides the Incomplete Visit Completion Flow Chart which serves as a tool when determining elements of the visit that can be collected at subsequent quarterly visits.

Splitting visits should be rare, as it presents a burden to the participant and separates the various study measurements from their protocol-defined time course. When scheduling a study visit, clinic staff should always prepare participants by notifying them of the expected length of the visit. However, sites are allowed some flexibility to determine the need to conduct a visit across two encounters if necessary. This should only be done when absolutely necessary and if allowed by the local IRB. In these situations, the two encounters should be scheduled as closely together as possible with the second occurring within 14 days of the first. When a visit is split across two encounters, only one Encounter & Disposition Form should be completed. The Encounter & Disposition Form should be completed on the second of the two days. For example, if all data collection elements are completed on the first day, with the exception of the MIND testing and the MIND testing is completed two days after the first visit, the Encounter & Disposition Form should be completed with the later of the two dates and Question 8 ("Was this protocol visit completed as specified in the protocol?") should be answered 'Yes.' If a visit is split across two days, the Event Ascertainment Form should be reviewed at both encounters to ensure that an event did not occur between the two.

If a second encounter cannot be performed within 14 days to complete the visit then the missed data collection elements should be collected at the next quarterly visit (i.e., for non-routinely collected items such as physical exam, ECG, and adherence). Note: in this circumstance the Encounter & Disposition Form should be completed to document the items that were missed. The Encounter & Disposition Form from the visit where the element was scheduled to be

collected should not be updated. Late data collection procedures for lab samples are outlined in Appendix 7 of Chapter 7, Central Lab. Late data collection for routinely collected items (i.e., BP Medications Log, Event Ascertainment, BP Management Form, and Participant Contact Information) cannot be collected at the next quarterly visit. Late data collection for non-routinely collected items may be performed up to 6 months after the time they were scheduled. "Time they were scheduled" indicates visit date (for partially completed visits) or target date (for completely missed visits). For example, if a participant does not complete the Annual History and Physical Exam at the 24M visit it may be collected at either the 27M or 30M visit. The exception to this rule is the ECG and MIND testing. If ECG testing is missed, this can be completed up to 1 year after the time they were scheduled. As an example, if an ECG is not completed at the 24M visit, it can be completed at any of the quarterly visits up through the 36M visit. MIND testing can be completed up to 1 year and 9 months after the time they were originally expected to be collected. Data collected late at a subsequent quarterly visit should be entered in the PRN data entry area and associated with the date of the quarterly visit. A PRN Encounter & Disposition (E&D) Form should be completed to document items collected late in order to receive payment. If the late data collection occurs in conjunction with a planned quarterly visit: complete and E&D Form for both the PRN data collection and the quarterly visit (both forms will have the same visit date).

11.4 Procedures for BP Inactive Participants

A participant's BP activity status does not affect form completion. The **Encounter and Disposition Form** will be used to document BP activity status at each visit. This form should be completed to document all protocol mandated clinic visits that were completed or missed as a result of trial inactivity and/or non-compliance. Also use this form to document any forms and/or procedures expected but not performed for each planned clinic visit.

BP Active/inactive status is assessed at the end of each clinic visit. As described above, if the participant's blood pressure is being managed by the SPRINT study, the participant is considered BP active. At the discretion of the clinic PI and study coordinator, BP inactive participants should be asked and encouraged at each encounter to resume BP active participation in the trial(s). If the participant is BP inactive, the reason why the participant is BP inactive is indicated in question 1, as well as indicating whether the site has taken actions at this visit to try to make the participant BP active.

All forms and procedures as outlined in the protocol are expected to be completed for BP inactive participants. The Encounter and Disposition Form is used to indicate which forms and procedures were not collected.

The **Encounter and Disposition Form** is completed for each visit for BP inactive participants. If a participant has agreed to be followed for outcomes only, the form should indicate that the visit was not completed as specified in the protocol (Q8) and the forms/procedures that were to be collected but were not should be selected as part of the subquestion to Question 8. If a participant has agreed to be contacted only at the end of the study, the **Encounter and Disposition Form** is completed for each visit, indicating that the visit was not completed because the participant is BP inactive/alternative follow-up plan has been established (option 6 on Question 9).

11.5 Procedures for Lost to Follow-Up and Withdrawn Participants

The clinical site must follow every randomized participant until the end of the study unless death or a withdrawal of consent precedes planned study duration. Loss of participants will adversely affect study power and the ability of the study to address its hypotheses. For SPRINT to validate

the primary outcomes, diligence in participant adherence is essential to the success of the trial. However, there will be times when participants either withdraw consent for further participation or become lost to follow-up and cannot be located. The **Participant Status Log** should be used to document such circumstances.

If all of the participant adherence recommendations found in this chapter have been addressed but the participant either refuses further attempts at contact or the clinic has not collected data from the participant within the past 12 months and an alternative follow-up plan has not been established (e.g., end of study contact only), the clinic staff should contact the CCN Coordinator and complete the SPRINT Participant Status Log. This form will be used to document a participant's status as either a "true withdrawal" (e.g. desires no further contact of any kind with SPRINT staff) or a participant that becomes lost to follow-up. The Participant Status Log is also used to document a lost to follow-up participant's return to BP active study management or BP inactive status with study surveillance. Data entry of the Participant Status Log is not allowed without prior approval from your CCN Coordinator. Completion of this form will negate the need to complete any study visit forms for missed clinic visits for participants identified as refused or lost to follow-up.

Once the Participant Status Log has been completed and saved, future clinic visits for that participant are removed from the "expected" column in reconciliation reports. That is, the Participant Status Log proactively documents future visits as missed. For all visits with a window open prior to the date listed as date of visit on the Participant Status Log, the Encounter and Disposition Form must be completed. If a lost to follow-up participant is returned to follow-up, expected visits will be resumed from the point that the participant is returned by updating the Participant Status Log.

11.5.1 Designating a Participant as a withdrawal or lost to follow-up. Prior to designating a participant as a 'withdrawal' or 'lost to follow-up', clinical site staff will attempt to negotiate follow-up contact choices (as above) with the participant or designated alternate contact (in the case of a possible lost to follow-up or non-responding participant).

Prior to designation as a 'withdrawal', all of the choices listed above should be discussed, CCN discussions should have taken place and the participant should have officially withdrawn consent (e.g. refusing any type of communication [phone, clinic, home]). The clinical site should document all attempts and participant wishes.

Prior to the participant being designated as 'lost to follow-up', clinical site will work through the contact decision tree below. Attempts should be documented (along with CCN discussions) in order for the participant to be designated as lost to follow-up.

11.5.1.1 Participant contact decision tree (for finding lost to follow-up participants):

1. Phone participant (minimum 5 attempts at different times of day/evening/weekends and by different staff including PI)
2. Email participant (if available)
3. Letter to participant (PI)
4. Phone other contacts
5. Letter to other contacts
6. If phone #/addresses believed to be inaccurate - EMR review, website searches, etc.

The site PI is to be involved in contact decision tree steps (as needed).

11.5.2 Documenting Withdrawals. A withdrawn participant has withdrawn consent to participate in the study in any way including denial of access to any personal future contact. All efforts should be made by the investigator and staff to negotiate a final clinic visit for the purpose of data collection, event ascertainment and medication adherence. If the participant refuses to come for a clinic visit, request some acceptable minimum protocol adherence that provides approval until the end of the study. This minimum acceptable protocol adherence is a final phone call to allow documentation of vital status and primary outcomes. If these negotiations are acceptable to the participant, DO NOT complete a Participant Status Log.

In SPRINT all randomized participants will be:

- followed even off study medications
- followed even after study events
- followed even after a serious adverse event

If after all reasonable efforts at retention have failed and the participant is adamant about discontinuing any further involvement in SPRINT, complete "Part A" of the Participant Status Log.

11.5.3 Documenting Lost-to-follow-up. Use the Participant Status Log to document participants if the clinic has been unable to collect data from the participant within the past 12 months and an alternative follow-up plan has not been established. Complete Part B of the form and complete the appropriate information to document contact attempts. Attempts to reach lost to follow-up participants should be performed on a bi-annual basis at minimum. Those contacts should be documented on this form as well. Once the clinic has documented the first three contact attempts, use the "most recent contact attempt" column to continue to document contact attempts. If the participant does contact the SPRINT clinic again, and wishes to return to the study, check the box in the Returning to Study section and data enter the date (Part C).

11.5.4. Completion of the Participant Status Log. Once a participant has been deemed as a refusal or lost to follow-up, the Participant Status Log should be completed. Sites are blocked from data entering this form initially. Prior to the CCN Coordinator opening the form, conversations and documentation about steps completed to try and retain the participant must be done. Only CCN Coordinators are able to initiate data entry of this form. Sites should follow this procedure:

1. Site print off the Participant Status Form (they are blocked from data entry) and complete the paper version.
2. Site calls or e-mails the CCN Coordinator to get approval to have this participant marked as lost to follow-up. Site and CCN Coordinator should discuss the contact attempts. Within this communication, they should notify the CCN Coordinator of the barcode on the form.
3. If the CCN approves the request, the CCN Coordinator will go to Data Management > Data Entry and enter the barcode. The CCN Coordinator will record the date they approve the status change in the "date of visit" field on the Participant Status Log and save the form. Note that the CCN Coordinator will see warning messages about missing fields; these will not prevent the form from saving and can be ignored by the CCN Coordinator.
4. After the CCN Coordinator has saved the form once, the form will be unlocked and the site can go in and update the date of visit to indicate the appropriate date and data enter all other information on the form.

Appendix 11.A

Protocol Adherence Flowchart

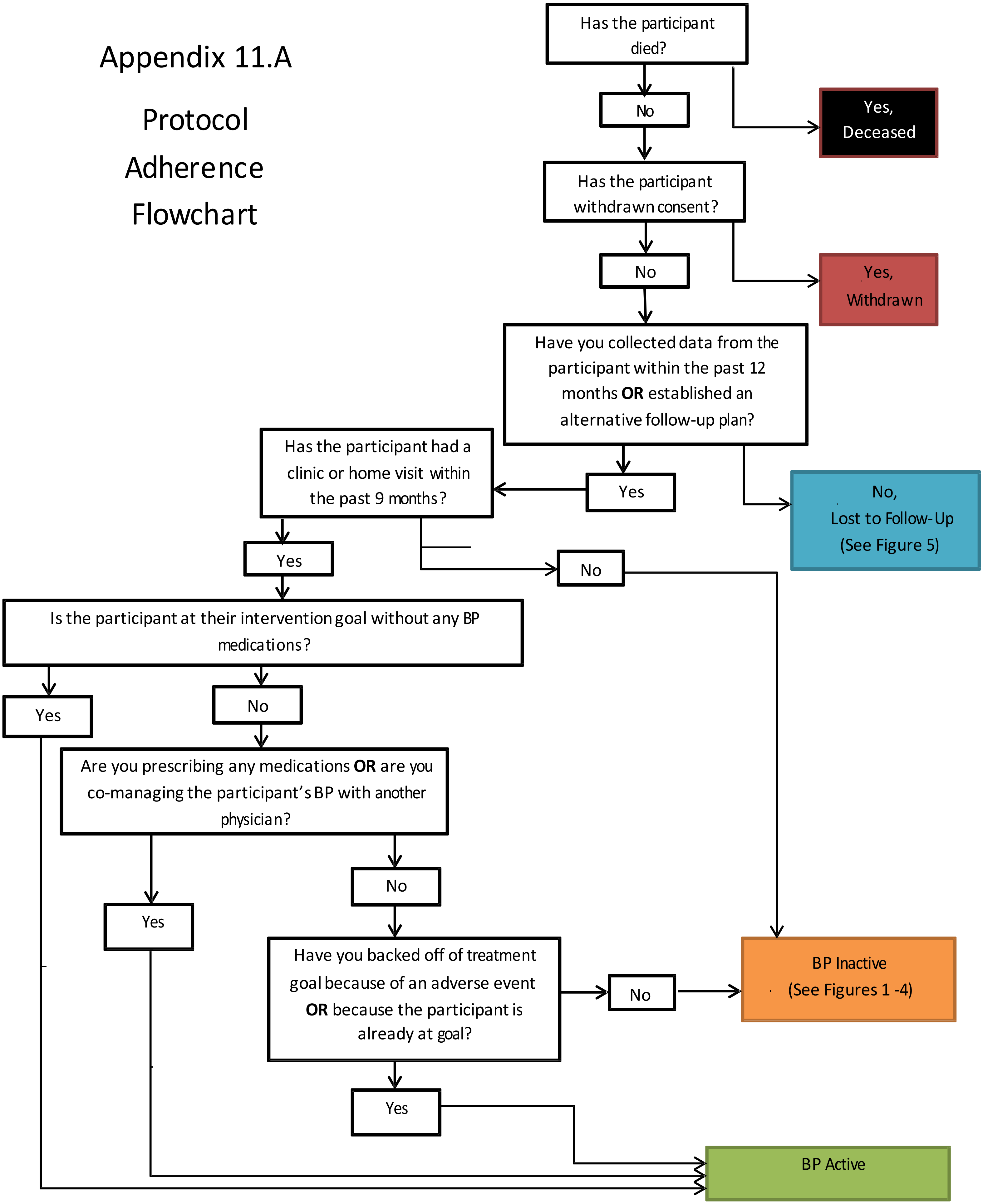

Appendix 11.B Adherence Figures

Figure 1. How to Increase Communication and Activity for participants who do not want to take any BP medications

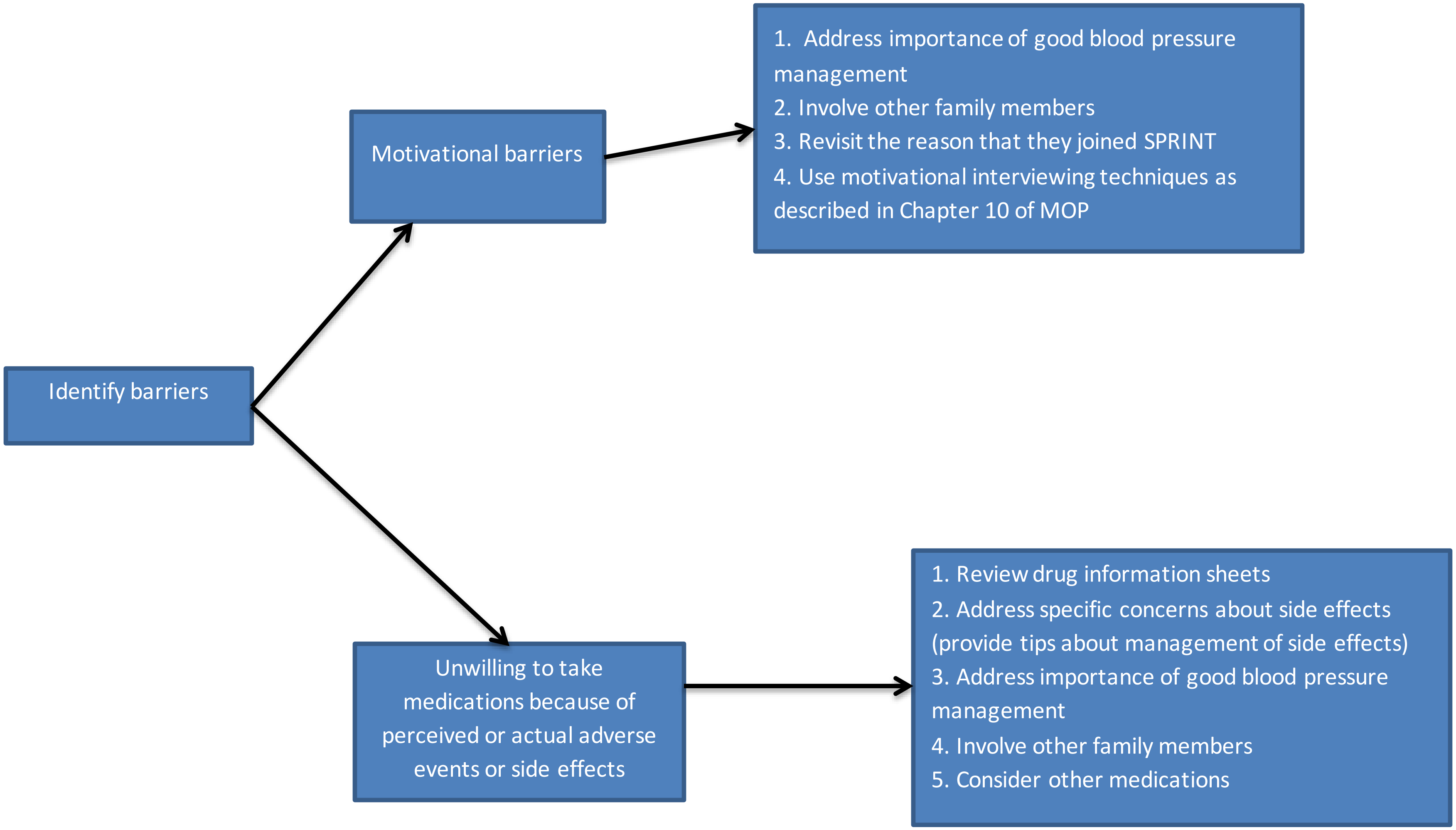

Appendix 11.B Adherence Figures

Figure 2. How to Increase Communication and Activity for participants who are having BP meds prescribed exclusively by PCP or non-SPRINT physician

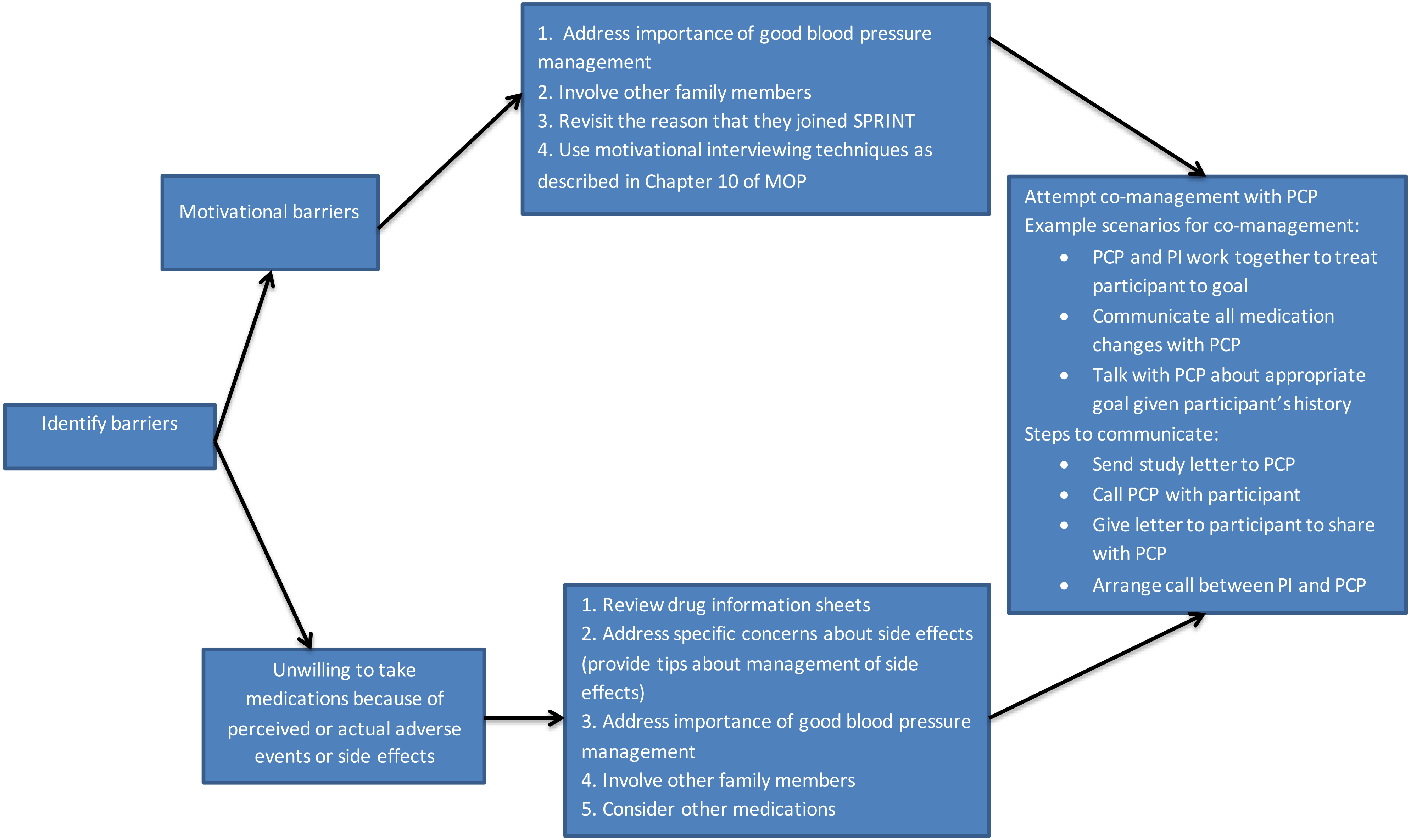

Figure 3. How to Increase Communication and Activity for participants who have agreed to be followed by phone exclusively

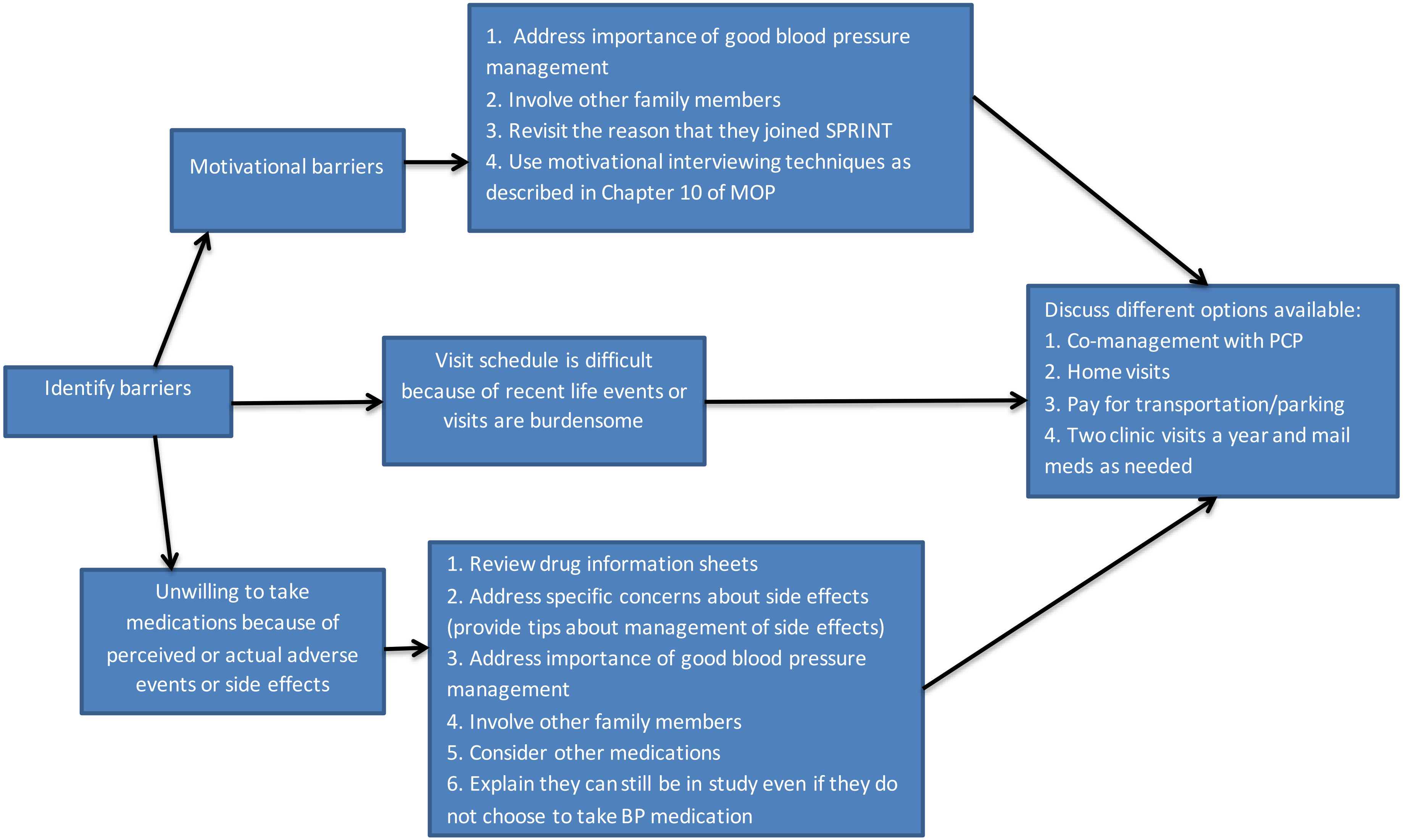

Appendix 11.B Adherence Figures

Figure 4. How to Increase Communication and Activity for participants who have agreed to only contact at the end of the study

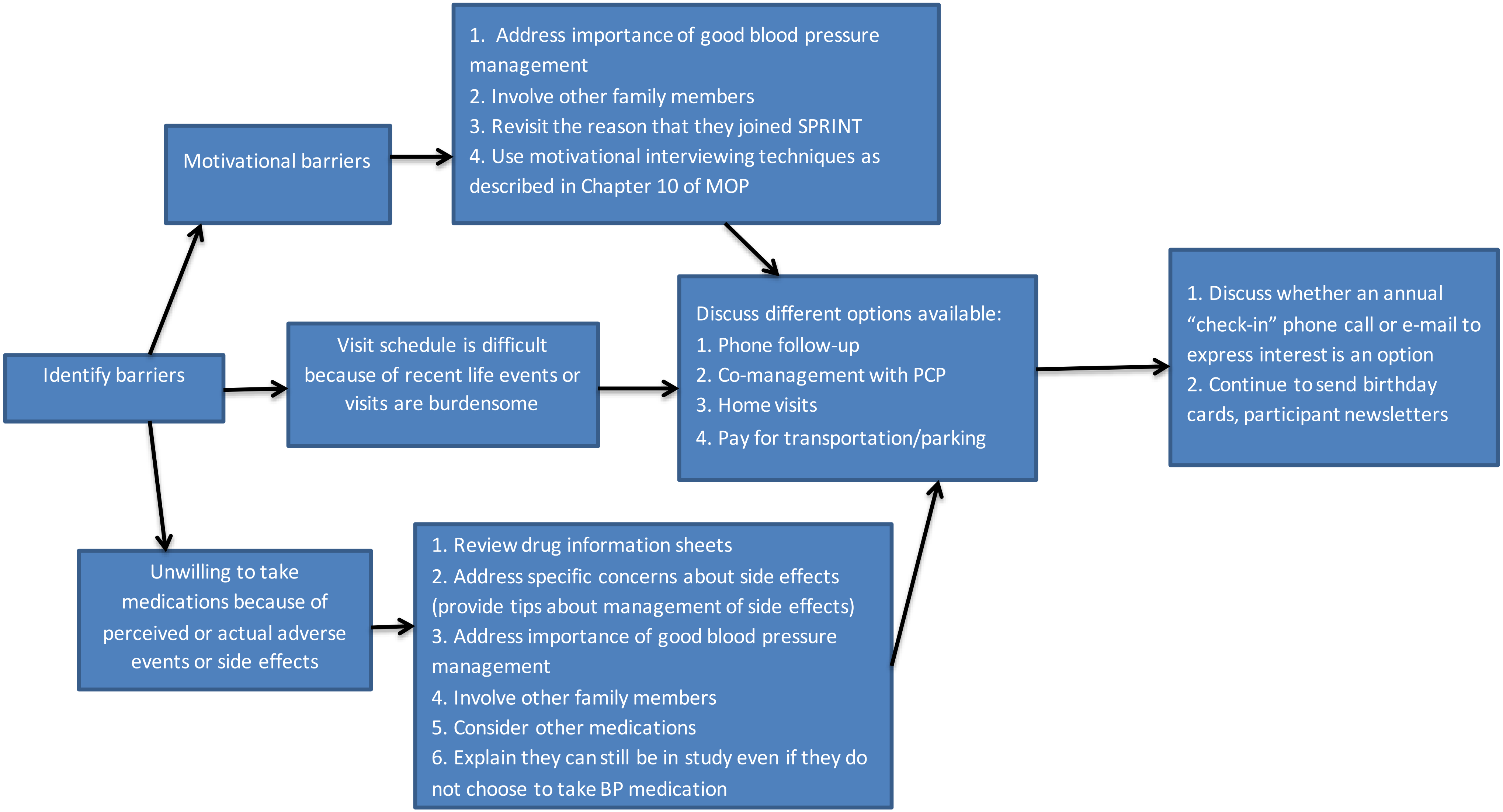

Appendix 11.B Adherence Figures

Figure 5. How to Increase Communication and Decrease Lost-to-Follow-Up

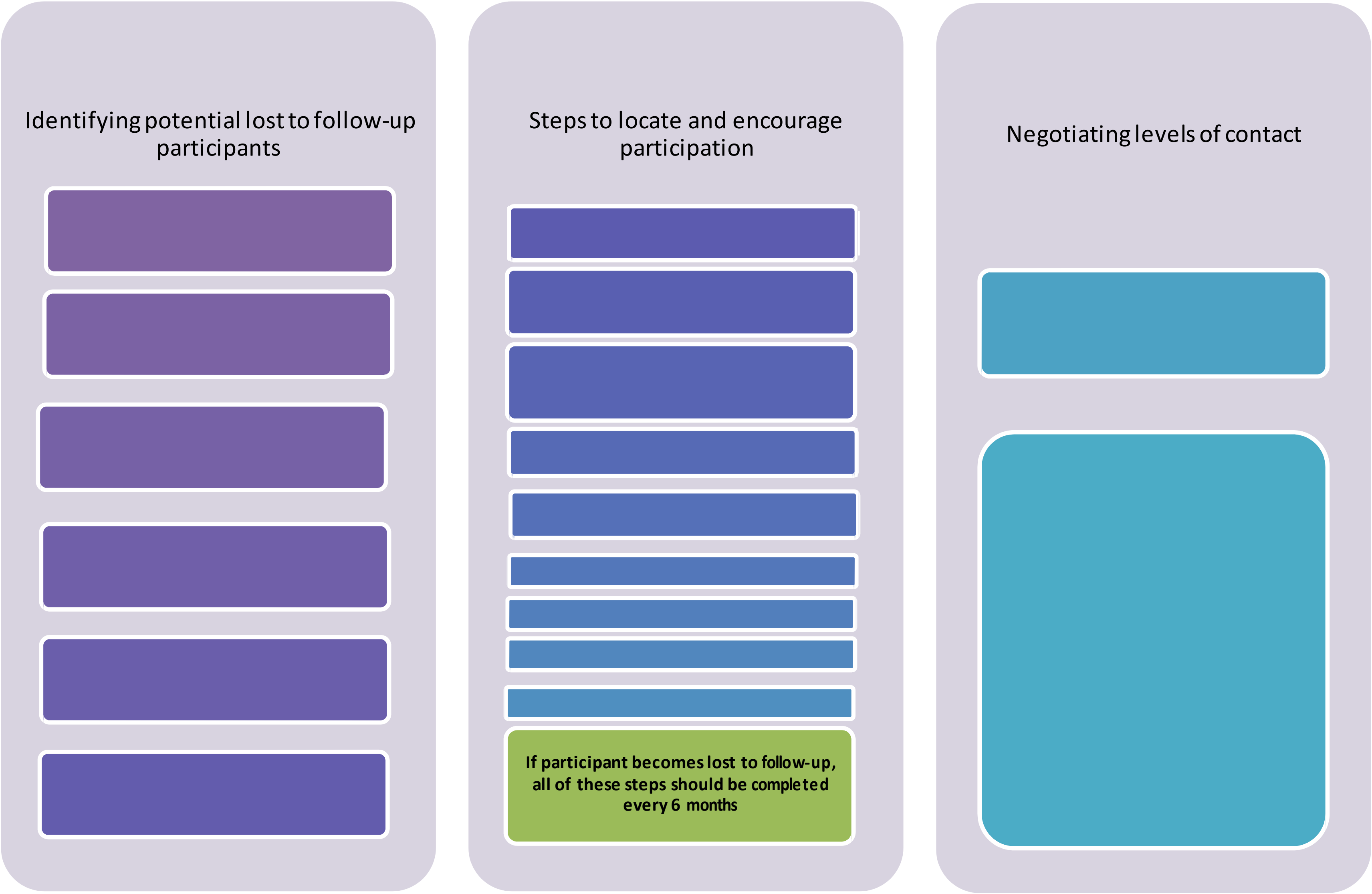

Appendix 11.C

Missed and Incomplete Visit Flowchart

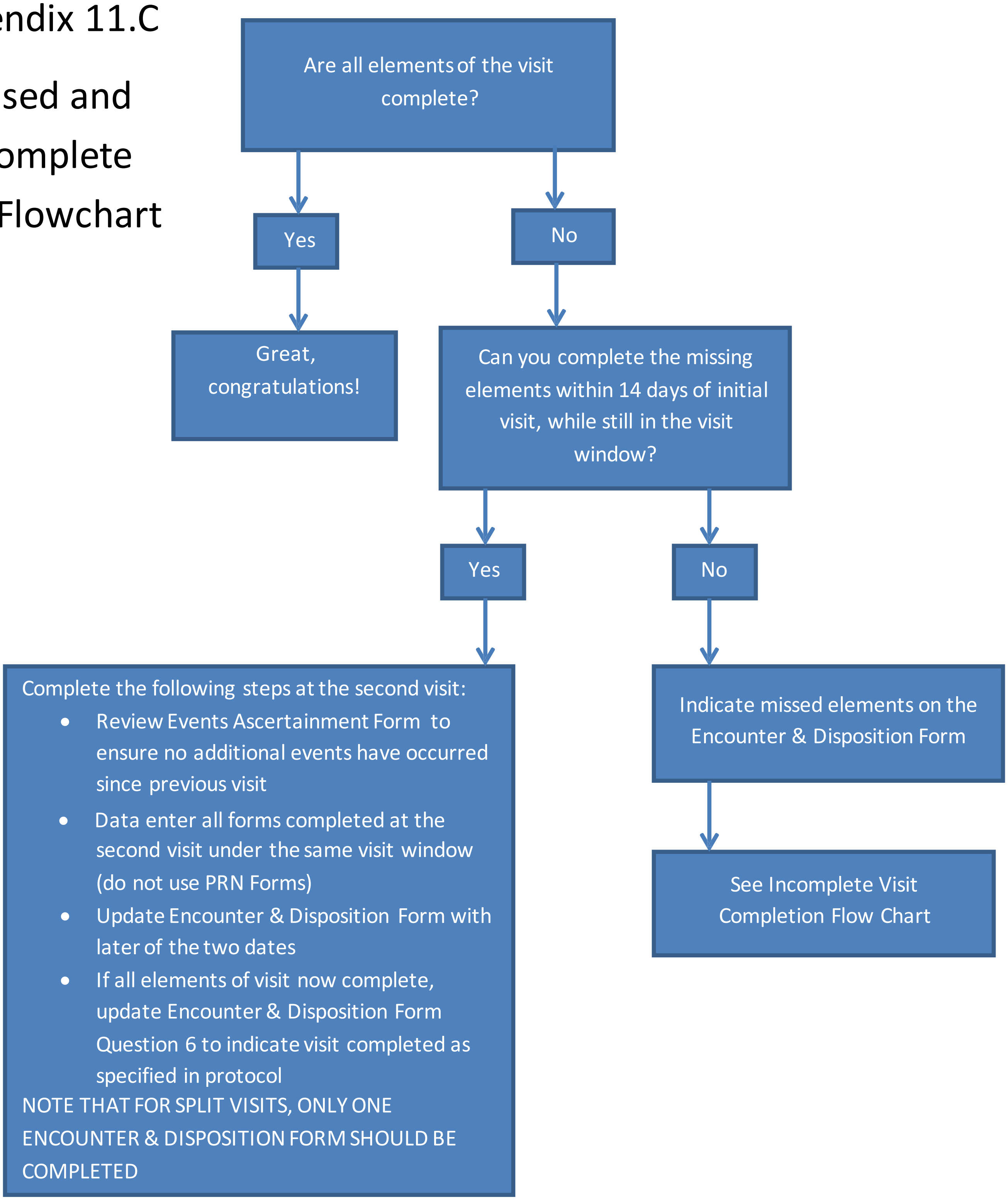

The exception to the 14 day window for the subsequent visit is for blood collection samples:

- Blood collection samples may be collected up to 4 weeks after the original visit date using the REDRAW Form (located under PRN)

Appendix 11.D Incomplete Visit Completion Flow Chart

Identify missing elements below:

One or more of the following:

- Events Ascertainment
- Blood Pressure Management
- Blood Pressure Medication Log
- Participant Contact Information

CANNOT RETROSPECTIVLEY BE COLLECTED AT NEXT VISIT

Blood Collection

DNA sample can be recollected at any time

Is this for samples at 12M, 24M, 48M visit?

Yes

Using a REDRAW form, can be completed up to 9 months after the time they were scheduled*

No

Do not collect at later visit

One or more of the following:

- Annual History and Physical Exam (with 4 meter walk, if applicable)
- My Health Form
- Men's/Women's Health Form
- Falls Self Efficacy Form
- Morisky Adherence Scale

Can be completed up to 6 months after the time they were scheduled *

MIND Screening OR Extended Battery

Can be completed up to 1 year and 9 months after the time it was scheduled *

ECG

Can be completed up to 1 year after the time it was scheduled*

*"Time they were scheduled" indicates visit date (for partially completed visits) or target date (for completely missed visits)

Forms that can be mailed for completion include: My Health Form; Men's/Women's Health Form; Falls Self Efficacy Form; and Morisky Adherence Scale

Note that any elements completed at a subsequent visit should be entered as a PRN form. A PRN Encounter & Disposition (E&D) should be completed to document items collected late in order to receive payment. If the late data collection occurs in conjunction with a planned Q3the visit where the element was scheduled to be collected SHOULD NOT be updated.

Chapter 12. Management of Participants with CKD

1. ESTIMATION OF GLOMERULAR FILTRATION RATE

• <u>4-variable MDRD equation</u>

The glomerular filtration rate is a commonly used measure of kidney function and correlates highly and inversely with the incidence of cardiovascular events and death. The GFR can be estimated using serum chemistry values and clinical characteristics. The 4-variable equation derived using the data obtained from the Modification of Diet in Renal Disease (MDRD) Study is the most commonly used method to calculate the estimated GFR (eGFR) among the clinics in the U.S. This MDRD equation is as follows:

$$\text{eGFR (in mL/min/1.73m}^2) = 186.3 \times (\text{serum creatinine in mg/dL})^{-1.154} \times (\text{age in years})^{-0.203} \times 1.212 \text{ (if black)} \times 0.742 \text{ (if female)}.$$

There are other estimation equations that are available, for example, the CKD-Epi equation. However, they are less commonly used and do not provide significant improvement in the accuracy of predicting the true GFR. Thus, SPRINT will use the 4-variable MDRD equation to derive the eGFR in individuals, for the purposes of (i) determining eligibility (≥ 25 mL/min/1.73m^2); (ii) classifying participants into the CKD subgroup (25-59 mL/min/1.73m^2) or non-CKD (> 60 mL/min/1.73m^2) subgroups; and (iii) assessing renal endpoints (e.g., 50% decrease in eGFR). Since many clinical chemistry laboratories routinely report the eGFR along with the serum creatinine concentration, the Clinical Sites can use these reported values for SPRINT purposes, provided that the Site has verified that the 4-variable MDRD equation is used by the clinical laboratory for eGFR calculations. Otherwise, the Clinical Site will need to calculate the eGFR using the above equation or website calculators (e.g., that found in the URL http://nkdep.nih.gov/professionals/gfr_calculators/orig_con.htm) by entering the four variables, serum creatinine concentration, age, race (Black or non-Black) and gender for the individual participant.

• <u>Caveats of MDRD equation</u>

It should be noted that the MDRD equation, and all available equations that are used to estimate GFR, may not be accurate for a given individual, especially if the patient has extreme body habitus or if the GFR is rapidly changing. Although SPRINT has adopted the 4-variable MDRD equation to determine eGFR for research purposes, caution should be exercised to apply the eGFR value for other clinical purposes (e.g., drug dosing), since the eGFR may significantly over-estimate or under-estimate the true GFR. Further, the clinician should be aware that the eGFR is normalized to the body surface area in the MDRD equation; estimation of the person’s GFR without this normalization would require additional calculations.

Persons who have rapidly changing serum creatinine concentrations over the preceding weeks or months probably have acute kidney injury. Further, the eGFR determined using the MDRD equation is inaccurate under this circumstance. Therefore, these persons should not be enrolled into SPRINT, at least not until the serum creatinine concentration has been stabilized for 3 months, and the eGFR and inclusion/exclusion criteria reassessed.

2. ASSESSMENT OF PROTEINURIA FOR ELIGIBILITY

- In subgroup analysis of randomized trials, lower blood pressure was associated with renoprotection in subgroups with heavy proteinuria (>1 g/d). Therefore, SPRINT excludes persons with significant proteinuria. Various methods are available for the determination of proteinuria, each has its advantages and disadvantages. Some considerations are as follows.

- Proteinuria often fluctuates during a 24-hr period and is usually higher in the upright position and during exercise than in the supine sedentary position. Therefore, a 24-hr urine collection is preferable under the ideal circumstances. The major problem with the 24-hr urine collection is that it is cumbersome for the patient to collect, store and submit and the completeness of the collection is often unreliable.

- A random (spot) urine sample without timed collection is more convenient, but has the drawback of variations in proteinuria depending on the level of physical activity around the time of urine collection. This is particularly problematic for monitoring longitudinal changes in proteinuria over time.

- Albuminuria is more specific for kidney disease, especially those related to glomerular disorders, and is therefore preferable to total proteinuria. The assay of albumin is, however, more expensive and is not always available in the clinical medical records that are used for eligibility screening for SPRINT.

- The dipstick method to detect proteinuria as part of the urinalysis in a random urine sample is the least desirable, since it has the additional disadvantage in that the dipstick method is only semi-quantitative and is influenced significantly by the variation in urine concentration (i.e., specific gravity).

- With these advantages and disadvantages taken into consideration, SPRINT accepts the following methods of urine collection and assays performed within 6 months of screening to determine if a participant is ineligible for enrollment:

  (a) 24-hr urinary protein excretion ≥1 g/day, or
  (b) If measurement (a) is not available, then 24 hour urinary albumin excretion ≥ 600 mg/day, or
  (c) If measurements (a) or (b) are not available, then random urine protein/creatinine ratio ≥ 1 g/g creatinine, or
  (d) If measurements (a), (b), or (c) are not available, then random urine albumin/creatinine ratio ≥ 600 mg/g creatinine, or
  (e) If measurements (a), (b), (c), or (d) are not available, then urine dipstick ≥ 2+ protein.

  The results of the urine tests listed in items (a)-(e) above are expected to be extracted from the medical records. The quantitative relationship between urinary total protein and albumin varies, depending on the magnitude and origin of the proteinuria, which in turn are influenced on the presence or absence and type of kidney disease.

- It is recognized that RAAS blockade may mask the degree of proteinuria; hence, one may question that the assessment of proteinuria for eligibility determination should be performed

in the absence of RAAS blockers. However, it is unclear if modest proteinuria in the presence of RAAS blockade affects the relationship between blood pressure levels and GFR decline. In addition, it would be cumbersome to withhold RAAS blockade for screening purposes. More importantly, the requirement of withholding these drugs may also deter the recruitment of subjects who are already on RAAS blockers prescribed for renoprotection or cardiac protection. Thus, the assessment of proteinuria or albuminuria (and also the assessment of eGFR) as part of screening for SPRINT can be evaluated in the presence or absence of RAAS blockers.

3. USE OF RAAS BLOCKERS IN CKD

- In proteinuric patients, especially those with diabetic nephropathy, ACEi or ARB has been shown to decrease proteinuria and decrease the rate of decline in GFR. Thus, ACEi or ARB is recommended in patients with proteinuria >0.3 g/day, unless there are contraindications, such as hypersensitivity to these drugs or hyperkalemia. In patients with milder degree of proteinuria or no proteinuria, the renoprotective effects of ACEi or ARB are less certain. The Clinical Site investigators should take these notions into consideration when prescribing anti-hypertensive medications for SPRINT.

- There is currently no credible evidence that the combination of ACEi with ARB has meaningful renoprotective effects over that of ACEi or ARB alone. There are limited randomized-trial data, exclusively in diabetic patients, showing that (i) the direct renin inhibitor aliskirin provides additional renoprotective effects when added to an ARB; and that (ii) the aldosterone antagonist spironolactone provides additional renoprotective effects when added to an ACEi. However, the additional anti-hypertensive effects are modest at best and there are also additional side-effects with these combinations. Therefore, combinations of RAAS blockers (ACEi, ARB, aldosterone antagonists and direct renin inhibitors) are not recommended, although there is no prohibition for the use of these combinations by SPRINT. Their use will be dependent upon the discretion of the individual Site investigators and primary-care providers.

- A common effect of ACEi and ARB is an increase in serum creatinine concentration. It is largely caused by a decrease in efferent arteriolar resistance, intraglomerular filtration pressure and hence a decreased in GFR, without structural damage. This hemodynamic-mediated increase in serum creatinine is therefore expected with the administration of ACEi or ARB and is readily reversible. In fact, many investigators believe that this acute decrease in intraglomerular pressure, with its attendant decrease in GFR, is necessary for the long-term renoprotective effect of ACEi and ARB.

- The increase in serum creatinine associated with ACEi or ARB often occurs gradually and stabilized within two months. However, it can also occur within days, depending on whether there are co-existing conditions that acutely decrease glomerular blood supply, such as volume depletion with or without diuretics and the use of NSAIDs. Decompensated heart failure and significant bilateral renal artery stenosis are chronic conditions that diminish glomerular blood flow and therefore also potentiate the negative effects of ACEi and ARB on GFR.

- Two other modulating factors are of particular importance to SPRINT. Lower systemic SBP, with diminshed perfusion to the kidney, is a risk factor for decreased GFR following

the initiation of ACEi or ARB. This risk is accentuated in patients with CKD, in which auto-regulation of the intra-glomerular pressure is impaired. Therefore, in the presence of these predisposing conditions, heightened caution is warranted.

- Although the increase in serum creatinine concentration associated with ACEi or ARB is readily reversible, it could still lead to significant functional and metabolic consequences, such as fluid retention, hyperkalemia, acidosis and symptoms of uremia. Such an increase in serum creatinine concentration is less likely when using mineralcorticoid receptor antagonists (MRA), such as spironolactone and epleronone, since these agents do not decrease the production or action of angiotensin II.

- Prior to the initiation of ACEi or ARB, factors that predispose to an increase in serum creatinine concentration should be eliminated or minimized. In particular, volume depletion should be corrected. It is reckoned that the optimal volume status is sometimes difficult to determine on clinical grounds, but every attempt should be made to achieve that goal.

- A side-effect of ACEi and ARB that is also shared by mineralcorticoid receptor antagonist is hyperkalemia, which is primarily the consequence of decreased mineralcorticoid activity in the kidney. In addition, ACEi and ARB can also induce hyperkalemia by impairing GFR. Therefore, it would be prudent to refrain from initiating or escalating the dose of RAAS blockers (ACEi, ARB, MRA and direct renin inhibitors) when the serum potassium concentration ≥ 5.0 mEq/L, although some clinicians initiate these agents and institute measures to lower serum potassium simultaneously.

- A recent meta-analysis shows that ARB is associated with an increased risk of cancer development, without an increase in cancer death. These results have not been confirmed in other large databases. Since ARB has renoprotective and cardioprotective effects in certain populations, there is currently no prohibition of using these agents for these indications, pending further decisions by the FDA. ACEi could probably be used in lieu of ARB for these indications; however ACEis are associated with a higher incidence of cough and angioedema than are ARBs.

4. INCREASE IN SERUM CREATININE CONCENTRATION

- An increase in serum creatinine concentration may be a Renal Outcome, an Adverse Event (that should be reported to the CC, who will in turn report the summarized data to the DSMB and in publications), or officially a non-event in SPRINT. Regardless, it often requires medical attention. It should also be noted that local IRBs may have different reporting requirements that the Site investigators must fulfill, in addition to the criteria for reporting to the SPRINT CC as an AE.

- Serum creatinine values will be obtained 1 month, 3 months, 6 months then every 6 months post-randomization in SPRINT. The threshold for the diagnosis of acute kidney injury (AKI) in SPRINT is a 50% increase in serum creatinine concentration within a 3-month period. The CC will alert the Clinical Site of these events. The Clinical Site should decide whether and which interventions for AKI are indicated as part of routine medical practice; there is no mandate from SPRINT that actions must be taken. If the participant has non-SPRINT chemistry performed that shows an elevated serum creatinine concentration, the Site

investigator or the primary provider should manage the situation per routine clinical practice. That particular serum creatinine value or other follow-up chemistry should not be reported to the CC unless: (i) the patient is subsequently hospitalized with an admission or discharge diagnosis of acute renal failure (the current nomenclature in the scientific literature and used by the NIDDK is AKI; however, acute renal failure is the terminology used in the International Classification of Diseases or ICD codes); or (ii) the follow-up chemistry is part of the official SPRINT laboratory test.

- It should be emphasized that inter-assay variations in serum creatinine, especially if the assays are performed in different laboratories, on the order of ± 0.2 mg/dL are not uncommon. A change from 0.5 mg/dL to 0.7 mg/dL could very well be due to assay variability or even heavy cooked meat intake. If the change is due to a real change in GFR, however, it would represent a rather substantial decrease. Clinical judgment and/or consultation with the CCN CKD Consultant are warranted for proper interpretation of the results and management.

<u>Management of increase in serum creatinine concentration</u>.

- The management of an increase in serum creatinine concentration, especially AKI, should follow good medical practice (GMP) or published practice guidelines. The CCN CKD Consultant or local nephrologist should be consulted for management, as necessary. Details of the management are beyond the scope of this MOP. A few important points are described as follows.

- In general, serum chemistry should be repeated within one month of the AKI occurrence or sooner, depending on the baseline values and the magnitude and rate of change in blood urea nitrogen (BUN) and serum creatinine, potassium and bicarbonate concentrations.

- A urinalysis is often useful to rule out a new or exacerbation of parenchymal kidney disease. For example, an active urine sediment (i.e., red blood cells, white blood cells and/or cellular casts) and a significant increase in proteinuria may be clues to glomerulonephritis or drug-induced interstitial nephritis. Nephrology consultation is recommended under these circumstances.

- Review of the medications, with particular attention to interim use of NSAIDs or other drugs that inhibit renal tubular secretion of creatinine (e.g. trimethoprim, cimetidine), and the discontinuation of these agents should be considered.

- Proper hydration and avoidance of kidney insults are generally recommended prior to repeating the serum chemistry.

- ACEi or ARB often increases serum creatinine concentration. An increase of < 20% is expected and usually of no significant concern, unless it reflects a decrease in GFR to a level that is associated with significant uremic complications, such as hyperkalemia, hyperphosphatemia and fluid retention. *The decision to withhold or reduce the dose of ACEi or ARB should be individualized and would partly depend on the absolute GFR value, rate of GFR decline and its consequences.*

- Urinary tract obstruction is a relatively uncommon cause of GFR decline, but should be considered particularly in the elderly male, in whom prostate hypertrophy is the most likely cause. A bladder scan in the clinic may reveal a large residual urine volume and/or a retroperitoneal ultrasound may show hydronephrosis.

- Large dietary intake of meat, which is a source of creatine, can increase serum creatinine concentrations.

- The frequency of follow-up phone calls and ad hoc clinical visits for elevated serum creatinine should be individualized, depending on the magnitude and rate of increase, the absolute creatinine concentration and the presence of systemic complications of uremia.

5. HYPERKALEMIA

- An increase in serum potassium concentration can be a life-threatening event. In most instances hyperkalemia is asymptomatic and discovered only by laboratory testing. In SPRINT, major causes of hyperkalemia to consider include, but are not limited to, deterioration of GFR, medications, dietary indiscretion and isolated hypoaldosteronism particularly in the elderly. The role of dietary indiscretion cannot be over-emphasized. These causes should be sought and corrected if possible. Some examples of drugs and herbals that are associated with hyperkalemia are as follows:

- Drugs that can cause hyperkalemia

  - Potassium supplements
  - ACEi
  - ARB
  - Aldosterone antagonists (spironolactone, eplerenone)
  - Direct renin inhibitors
  - Triamterene
  - Amiloride
  - NSAIDs
  - Cyclosporine
  - Tacrolimus
  - Beta-blockers
  - Trimethoprim
  - Pentamidine

- Herbals/other agents that can cause hyperkalemia

  - KCl-containing salt substitutes
  - Amino Acids (aminocaproic acid, arginine, lysine)
  - Dandelion
  - Dried toad skin
  - Hawthorne Berry
  - Horsetail
  - Liliy of the Valley
  - Milkweed
  - Nettle

– Noni Juice
– Siberian Ginseng

Definition of hyperkalemia for reporting in SPRINT

- Hyperkalemia is operationally defined in SPRINT as a serum potassium level ≥ 6.0 mEq/L (or ≥1.0 mEq/L above the upper limit of the normal range for the Central Laboratory) on any given scheduled SPRINT visit. Such a value will be recorded as an AE in SPRINT; confirmation by repeat testing is not required for the purposes of data analysis, or reporting to the DSMB and IRB. The rationale for not confirming the serum potassium value by repeat testing prior to reporting is that serum potassium varies significantly over time in a given individual. Nonetheless, any significant hyperkalemia, albeit transient, is of clinical interest.

- Serum potassium level >5.0 mEq/L (or above the upper limit of the normal range for the Central Laboratory) will also be compiled for data analysis, but will not be considered as an AE for the purposes of reporting to the DSMB or IRB.

- The DSMB may choose to examine data on potassium in other manners.

Management of hyperkalemia

- Independent of reporting in SPRINT, hyperkalemia is clinically important because it is potentially life-threatening. The management of hyperkalemia should follow good medical practice or published practice guidelines, with nephrology consultation as necessary.

- Serum K >6.0 mEq/L requires immediate actions. Serum chemistry should be repeated at the local laboratory. A common spurious cause of hyperkalemia is in vitro hemolysis in the collection tube; gross hemolysis with pink serum can often be confirmed by visual inspection of the specimen by the laboratory staff. If this degree of true hyperkalemia is confirmed, however, RAAS blockers should be withheld and other strategies of lowering serum potassium may need to be instituted, followed by repeat serum potassium measurement within one week.

- The history of dietary potassium intake should be reviewed and recommendations to curtail potassium intake should be made. The effect of dietary potassium restriction can at times be very substantial, depending on the participant's usual dietary habit. Foods that are typically high in potassium content are: (1) fruits (e.g., orange, peach, nectarine, apricot, banana, kiwi, avocado, melons); and (2) vegetables (e.g., tomato, potato, nuts, spinach, artichoke, beet, turnip). An important source of dietary potassium is salt substitutes, that are often used by individuals with hypertension or heart failure who have been advised to limit salt intake. Lowering the serum potassium concentration by dietary restriction may allow the use of RAAS blockers for their cardioprotective or renoprotective effects.

- Non-potassium-sparing diuretics, such as chlorthalidone, furosemide, metolazone and bumetanide, at dosages that promote diuresis, are effective in eliminating potassium. If diuretics are used as an adjunctive strategy to lower serum potassium, it is important to avoid volume depletion and consequently AKI. Sometimes, the combination of diuretics and liberal salt intake can be used for the chronic treatment of hyperkalemia, while

maintaining euvolemia. The oral administration of sodium bicarbonate can lower serum potassium concentration if the acidemia is present; however the sodium load may aggravate the hypertension or heart failure.

- If the serum potassium concentration is > 6.4 mEq/L, an immediate electrocardiogram is recommended. If ECG features of hyperkalemia are present (e.g., peaked T-wave and widened QRS complex), the participant should be immediately referred to a facility in which acute care is available, such as the emergency department, for urgent treatment. The Site PI or co-investigator or their colleagues, the primary provider and/or other physicians should be notified immediately.

- Other treatment modalities of hyperkalemia are beyond the scope of this MOP. The benefit/risk ratio of enteral sodium polystyrene sulfonate has been increasingly questioned. Bowel necrosis is perhaps the most feared complication. Some have challenged the effectiveness of polystyrene sulfonate in eliminating body potassium, beyond that induced by the cathartics, such as sorbitol, that are commonly mixed with the polystyrene sulfonate in commercial preparations. The use of insulin with or without glucose, intravenous calcium or acute dialysis for more severe cases of hyperkalemia requires close monitoring and should be performed in other settings, such as emergency departments, instead of the SPRINT research clinics.

6. SPECIAL CONSIDERATIONS FOR OTHER MEDICAL MANAGEMENT IN PATIENTS with CKD

- Diet

Because of the risk of hyperkalemia, the DASH diet (Dietary Approaches to Stop Hypertension) that is often prescribed to hypertensive individuals may not be suitable for patients with moderate or advanced CKD. There is no convincing evidence that dietary protein restriction slows the progression of CKD. However, high-protein diets, including some of those used for weight reduction, should be avoided in advanced CKD. Since many patients become malnourished as their CKD advances, in general diets in CKD patients should be quite liberal, with the exception of sodium restriction for most. Potassium and phosphorus restriction should not be universal, but should be instituted on an as-needed basis. Patients should be warned to avoid salt substitutes that contain potassium.

- Lifestyle

There are no specific considerations for CKD patients concerning life-style. All recommendations for promoting general and cardiovascular health for the non-CKD population apply to the CKD population as well. These include exercise, abstinence from cigarettes, weight reduction for obesity and management of psychological factors that predispose to cardiovascular diseases. There is, however, an apparent paradoxical relationship between lower body mass index and mortality in the chronic dialysis population, similar to that seen in populations with other chronic illnesses.

- Drugs

There are several considerations for drugs and the kidney:

(i) The class and dose of diuretics may need to be altered with declining GFR. For example, chlorthalidone and loop diuretics (furosemide and bumetanide) are more effective than hydrochlorothiazide at lower GFR (e.g., < 30 mL/min). Heavy proteinuria (that may occur in SPRINT participants during follow-up) may affect the pharmacodynamics and decrease the effectiveness of diuretics. Therefore, in the presence of both low GFR and heavy proteinuria, up to 480 mg of oral furosemide per day in divided dosages may be required to achieve adequate diuresis. The addition of metolazone to a loop diuretic may also be helpful.

(ii) Some drugs cause an increase in serum creatinine concentration, by inflicting injury to the kidney (e.g., aminoglycosides or drugs causing allergic interstitial nephritis) or decreasing the GFR without injury (e.g., the acute effect of ACEi or NSAID).

(iii) Some drugs are eliminated, at least partially, by the kidney via either metabolism (e.g., insulin) or excretion (e.g., lisinopril and atenolol); therefore these drugs accumulate in the body in patients with decreased GFR. On the other hand, the proteinuric kidney may enhance drug elimination, by increasing the filtration of protein drugs (e.g., epoietin) or protein-bound drugs (e.g., phenytoin).

(iv) The side-effects of certain drugs are accentuated in CKD. Notable examples are ACEi (causing hyperkalemia), hypertonic sodium phosphate enemas (causing hyperphosphatemia) and citrate (e.g., Shohl's solution and sucralfate that increase aluminum absorption from the GI tract).

(v) There is no specific recommendation for the CKD population on the use of aspirin for cardiovascular protection, although uremia predisposes to platelet dysfunction. In post-hoc subgroup analyses, statins were found to be effective in decreasing cardiovascular events and all-cause mortality in patients with Stage 3 CKD. In contrast, statins did not decrease cardiovascular events in chronic dialysis patients.

- Strategies for the attenuation of CKD progression and management of metabolic complications associated with CKD (e.g., hyperkalemia, anemia, metabolic acidosis, bone and mineral metabolism) should follow published practice guidelines, such as those provided by the Kidney Disease Outcome Quality Initiative (K/DOQI) and other literature, supplemented by sound clinical judgment.

- When eGFR declines to values lower than 30 mL/min/1.73m^2, referral to nephrologists for co-management of the patient is recommended.

7. ASCERTAINMENT OF AKI (ARF)

- <u>Data collection by Clinical Site</u>

STEP 1: Ask the participant during the Q3month formal SPRINT visit, whether he/she has been:

(a) hospitalized for kidney problems
(b) developed kidney problems while in hospital
(c) received dialysis while in hospital

(d) stayed in the intensive care unit while in hospital (where kidney failure and dialysis are more prone to occur).

STEP 2: If the answer is YES for any one of the above, request the following:

(a) Hospital discharge summary AND
(b) Serial blood chemistry in hospital from admission to discharge.

STEP 3: Send discharge summary and serial chemistries to Coordinating Center Safety Officer.

STEP 4: Follow-up of the AKI, if it occurred, should have been arranged as part of the hospital discharge planning. The SPRINT study coordinator may choose to contact the provider who is supposed to follow-up or the primary care provider if there are questions about the follow-up.

- Ascertainment of AKI by CC Safety Officer

There will be 3 different definitions for AKI:

(A) AKI requiring acute dialysis
(B) AKI (a.k.a acute renal failure) as one of the discharge diagnoses
(C) Increase in serum creatinine of >50% within 3 months

The two AKI outcomes will be:

(A) Definition (A) only;
(B) Composite of (A), (B) or (C)

Rationales for definitions of AKI:

(A) AKI requiring acute dialysis

Out of three definitions above, this is the most clinically significant outcome and perhaps the easiest to confirm. The indications for dialysis may be fluid overload, hyperkalemia, uremic signs and symptoms, abnormal chemistry per se and others. Dialysis for poisoning will NOT be included in this definition. It is anticipated that the threshold of these criteria for acute dialysis may vary from patient to patient and from physician to physician.

(B) Discharge diagnosis of acute renal failure according to ICD-9-CM diagnosis code 584.

This definition depends on the decision of the author of the discharge summary and does not necessarily follow any rules or standards. Nonetheless, it is quite easy to confirm.

(C) Increase in serum creatinine of >50% within 3 month to a value of at least 1.5 mg/dL

This is defined as an increase in serum creatinine concentration by > 50% within 3 months, *upon hospital admission or during the hospitalization*, from an initial value obtained from any source. These creatinine values will be extracted from the discharge summary and/or the serial chemistry values from the hospitalization. For uniformity, there is no need to search for data obtained outside the hospital if the data are not available in the hospital record. For example, (i)

if the discharge summary indicates that an outpatient serum creatinine was 1.0 and the patient was admitted because the creatinine was 4.0 or the serum creatinine on admission happened to be 2.0, this case qualifies as AKI in SPRINT. (ii) If the serum creatinine was 1.0 on admission and 90 days later, while still in the ICU or rehabilitation unit, the creatinine was 2.0 but eventually it went up to 3.0 at 120 days. This case qualifies as AKI in SPRINT, i.e., the peak creatinine may occur beyond the limit of 90 days, but the increase satisfies the magnitude (> 50%) and time frame (within 3 months). The Safety Officer does not need to compare the hospital values with the latest official SPRINT creatinine value to make this determination.

For logistical reasons, AKI occurring as an outpatient will NOT be counted as AKI, because of the difficulty in tracking and, more importantly, these occurrences are probably clinically less significant than those requiring hospitalization. An increase in serum creatinine confirmed by official SPRINT central laboratory measurements six months apart will be considered as a Renal Endpoint. The Renal Endpoint may be the result of de novo AKI or AKI superimposed on CKD. However, it will still be classified as a Renal Endpoint.

8 SAFETY CHEMISTRY MONITORING

- Rationale

The SPRINT protocol has incorporated a rigorous scheme of monitoring the acute (within 1 month) and subacute (within 6 months) changes in serum creatinine concentrations that may occur following the lowering of SBP to the respective randomized targets. According to this scheme, serum chemistry will be obtained at 1, 3 and 6 months after randomization, and every 6 months thereafter, on all participants. It is anticipated that most participants who have specific indications for RAAS blockers (e.g., CKD and heart failure) would already be placed on these agents prior to enrollment into SPRINT, according to the inclusion/exclusion criteria, although some participants will be placed on these agents during the follow-up of the trial.

As discussed in Section 3 above, both the lower SBP target (< 120 mm Hg) and the presence of significant CKD predispose to serum creatinine increase when ACEi or ARB are initiated. While participants randomized to the higher SBP target may be at a lower risk of serum creatinine increase, less rigorous monitoring and less medical attention provided to this group would create additional bias in the trial conduct. Therefore, the following safety chemistry monitoring procedures will be applied to both randomized groups.

In SPRINT, RAAS blockers (ACEi, ARB, MRA and direct renin inhibitors) should not be initiated when the serum potassium concentration ≥5.0 mEq/L. The following safety laboratory monitoring will be based on this assumption. Hyperkalemia can often be corrected in most participants, often by moderate restriction in dietary potassium intake. RAAS blockers can be initiated at a later date after the serum potassium concentration has been normalized.

- Safety laboratory monitoring following initiation or first dose increase of RAAS blockers (ACEi, ARB, MRA or direct renin inhibitor)

A serum basic metabolic panel (BMP) that consists of at least sodium, potassium, BUN and creatinine, should be obtained after the initiation or first dose increase of a RAAS blocker. The serum sample should be sent to the SPRINT Central Laboratory for processing. Please refer to

Table 14. 2 in MOP Chapter 14: Safety Monitoring and Reporting for recommended timing of safety labs after initiation or first dose increase of RAAS blockers and diuretics based on eGFR.

Note that there are no serum potassium criteria in this laboratory monitoring. RAAS blockers should not be initiated until the hyperkalemia has been corrected.

- Safety laboratory monitoring following initiation or first dose increase of diuretics

A BMP should be obtained after the initiation and first dose increase of diuretics, regardless of eGFR at the time. The serum sample will be sent to the SPRINT Central Laboratory. Please refer to Table 14. 2 in MOP Chapter 14: Safety Monitoring and Reporting for recommended timing of safety labs after initiation or first dose increase of RAAS blockers and diuretics.

Rationales: There are several reasons to check serum chemistry following the initiation of diuretics. (i) Both thiazide-type and loop diuretics tend to decrease serum potassium concentrations. (ii) Intravascular volume depletion induced by diuretics potentiates the effects of ACEi or ARB to decrease GFR. (iii) In the elderly participants, diuretics not infrequently cause hyponatremia.

The following table (modified from the K/DOQI Guideline) is recommended for management in response to the results of the first monitoring laboratory test following the initiation of ACEi or ARB. It considers the acute changes in serum creatinine concentration only but not the serum potassium concentration. It outlines the recommended actions on (i) whether the dose of the RAAS blocker should be altered; (ii) when the next safety monitoring laboratory test should be performed; and (iii) the causes of increase in serum creatinine concentration that should be considered.

	Acute increase in serum creatinine following initiation of ACEi or ARB			
	0-15%	15-30%	30-50%	>50%
Dose adjustment for ACEi or ARB	None	None	Reduce	Discontinue
Next chemistry test	Next study visit	2-8 weeks	7-10 days	7-10 days
Evaluate causes of increase in serum creatinine	Investigate if volume depletion, NSAID and other causes of AKI are present		In addition to other causes of AKI, specifically consider heart failure and renal artery stenosis	

If hyperkalemia is present in the initial monitoring laboratory test or the rate of change in serum potassium concentration is high, appropriate actions to lower serum potassium concentrations should be considered. The strategies to treat hyperkalemia are presented in Section 5 above in this chapter.

It must be emphasized that every attempt should be made to maintain the SBP target to which the participant has been randomized in SPRINT. The CCN CKD Consultants and the CCN PI must be consulted for any consideration of deviation from this target.

- Additional safeguards

The algorithms described above are designed to promote the safety of the SPRINT participants and the integrity of the trial, while allowing the flexibility for the investigators to tailor the management to the specific circumstances. In addition to unnecessary burdens to the trial and study personnel, over-zealous monitoring also generates undue burden on the participants.

(b) Early monitoring laboratory tests. In the setting of initiation of RAAS blockers or diuretics, safety monitoring laboratory tests can be obtained earlier than the mandated time point (e.g., one month for eGFR ≥ 30 mL/min/1.73m^2 and 2 weeks for eGFR < 30 mL/min/1.73m^2 for ACEi initiation). In that case, the monitoring safety laboratory test at the pre-specified time point would no longer be mandated. The Site Investigator will notify the CC about earlier laboratory test and will again use clinical judgment to decide when additional follow-up laboratory tests should be performed.

(c) Practice guidelines. The Site Investigator is advised to consult published practice guidelines, such as the K/DOQI Guidelines, to help determine when safety monitoring laboratory tests should be performed for additional follow-up after the initiation of RAAS blockers or diuretics under specific circumstances. It should be noted that the K/DOQI Guidelines in this respect are largely opinion-based. Consultation of these practice guidelines following dose changes is also recommended for formulating the decision to obtain safety monitoring laboratory tests.

(d) Medication dose decrease. It should also be noted that, besides dose escalation, a decrease in dose of these agents may also incur risks. For example, a decrease in diuretics may induce hyperkalemia, while a decrease in RAAS blockers may induce hypokalemia. These potential events should be considered in medication dose reductions.

(e) CCN Consultants. In addition to consulting published practice guidelines, the Site investigator is encouraged to discuss cases with the CCN CKD Consultant, if there are uncertainties about the appropriate course of action. Each CCN will have two CKD Consultants designated for this purpose.

(f) Review of safety data early in the trial. The unblinded members of the CC will present the individual and composite safety data to the DSMB during the first year of the trial to examine (i) the frequency and extent of changes in serum creatinine and potassium concentrations following the initiation of RAAS blockers and diuretics; (ii) whether the current safety laboratory test monitoring algorithm is adequate; (iii) whether the responses of the Site investigators to the safety laboratory results are adequate; and (iv) whether changes in the algorithm in safety monitoring and actions are necessary.

- Summary of recommended safety monitoring laboratory tests for RAAS and diuretics

(a) For participants with eGFR ≥ 30 mL/min/1.73m^2 at the time of initiation or first dose increase of any RAAS blocker, a serum basic metabolic panel (BMP), comprising of sodium, potassium, BUN and creatinine, should be obtained within 1 month.
(b) For participants with eGFR < 30 mL/min/1.7^{3}m^2 at the time of initiation or first dose increase of any RAAS blocker, a serum BMP should be obtained within 2 weeks.

(c) A serum BMP should be obtained within 1 month after the initiation or first dose increase of diuretics, regardless of the eGFR at the time of initiation.

(9) END-STAGE RENAL DISEASE (ESRD) PATIENTS IN SPRINT

- Depending on the number of Stage 3A and Stage 3B CKD participants enrolled, the number of patients reaching ESRD in SPRINT is estimated to be 150-200.

- Blood pressure interventions

  Continuing the blood pressure intervention with the randomized target is problematic in chronic hemodialysis patients, since blood pressure fluctuates substantially over time, especially during the hemodialysis session. Patients on chronic peritoneal dialysis and kidney transplant recipients do not have similar problems with blood pressure fluctuation compared to chronic hemodialysis patients, but the number of SPRINT participants on these modalities of ESRD therapy is expected to be quite small. *Therefore, the randomized blood pressure intervention will be discontinued in all SPRINT participants once ESRD has been reached, regardless of the ESRD modality (hemodialysis, peritoneal dialysis or transplant).*

  Nonetheless, data collection will continue with Q3 phone calls and annual in-clinic visits and the events for the primary (cardiovascular) outcome will not be censored after reaching ESRD. There are several shortcomings associated with the censoring of cardiovascular events after reaching ESRD: (i) it violates the intent-to-treat principle; (ii) it risks informative censoring; (iii) it would eliminate a non-trivial number of outcome events, since ESRD patients typically have high cardiovascular event rates. Sensitivity analysis will also be performed in which the cardiovascular events that occur after the participants reach ESRD are censored.

- Timing of outcome measurements.

Immediately pre-dialysis, the patients are usually fluid overloaded which may impair exercise tolerance and testing in SPRINT. During hemodialysis, patients often experience hemodynamic and neurological signs and symptoms, including hypotension, dizziness, disequilibrium syndrome, muscle cramps, restless legs, and pruritis. Post-dialysis asthenia and a washed-out feeling occur frequently and may last several hours or for the remainder of the day. While conducting SPRINT study visits around the hemodialysis session is convenient for the patient, the information obtained in this setting may not be representative of the interdialytic period. This is not an issue for patients on chronic peritoneal dialysis or those who received kidney transplant; again, the number of patients in the two latter categories is expected to be small.

Therefore, SPRINT outcome measurements should preferably be performed during the off-dialysis days for chronic hemodialysis patients. If this cannot be accomplished, the measurements should be obtained pre-dialysis and not within one hour post-dialysis. The Site investigator should use discretion to decide the best timing of the SPRINT visits in relationship to the hemodialysis schedule, depending on the individual participant and the type of data that need to be collected during that visit. The investigator and Study Coordinator should also be cognizant that the disease and medical treatment (thrice-weekly or more frequent dialysis)

burdens are very high for the dialysis patients; however, the reliability of the SPRINT data is essential for the integrity of the trial.

(10) DEATHS FROM KIDNEY DISEASE

While cardiovascular disease and infection are the two most common causes of death in patients with end-stage renal disease and probably in patients with advanced CKD, occasionally these patients die from causes that are complications of kidney failure that are preventable by adequate dialysis. The most common cause of death within this category is uremia, which is characterized by the gradual deterioration in mental status over days, sometimes accompanied by fluid overload, pericarditis, bleeding and other uremic manifestations, culminating in cardiopulmonary arrest. They may or may not have hyperkalemia or acidosis, but the presence or absence of these electrolyte disturbances is not essential for the diagnosis of uremic death. The patient may be already on chronic dialysis, but the patient or the family decides to withdraw from dialysis. Alternatively, the patient may be deemed to require dialysis by the medical provider, but the decision is made not to initiate dialysis. In some instances, the patient may have documented hyperkalemia with progressive ECG changes and/or pulmonary edema that are likely to be the cause of death.

Deaths from kidney disease are defined as one of the following, *in the absence of other causes*:

- that occur within 30 days after the patient withdraws from chronic dialysis;
- that occur when the dialysis patient dies from hyperkalemia (judgment will need to be made by the adjudicators whether the hyperkalemia is the likely cause of death);
- that occur when the patient has been diagnosed to have ESRD but dialysis was not initiated for any reason.

It is anticipated that the etiology or classifications of some cases will be nebulous. An example is hyperkalemia with RAAS blockade in a patient with advanced CKD who is not on dialysis; we would arbitrarily not classify this case as death from kidney disease. It is important to rule out other obvious causes of death. For example, if a patient has terminal cancer and decides to withdraw from dialysis, the cause of death should be classified as death from malignancy, rather than uremia.

(11) DEATHS RELATED TO DIALYSIS

These are deaths that are related to the dialysis procedure and not the result of impaired kidney function. Examples are intradialytic hemolysis or air embolism as a result of extracorporeal circuit malfunctioning, and sepsis as a result of infected hemodialysis catheters or peritoneal dialysis catheters. Death from calciphylaxis or arrhythmia related to severe heart failure, which are systemic disorders that are more likely related to kidney failure, should not be classified as deaths related to dialysis.

There are other cases in which the death is related to ESRD treatment but does not fit into this category of “Death Related to Dialysis”, e.g., death as a result of acute complications of kidney transplant surgery. These cases will be classified without regards to the kidney failure.

Chapter 13. Ascertainment and Documentation of Study Outcomes

Outcomes procedures, outcomes forms

13.1 INTRODUCTION

After randomization, a SPRINT participant may be hospitalized, undergo an outpatient surgical procedure, or die, and thereby experience any number of conditions that could be clinical outcomes of interest to the study. These clinical outcomes include a new or recurrent myocardial infarction or stroke, development of heart failure, hospitalization for an acute coronary syndrome, development of end-stage renal disease (ESRD) requiring chronic dialysis or kidney transplant, and undergoing a cardiovascular procedure. These events require completion of specific study forms and collection of specific medical records (explained further in this chapter of the MOP). In addition, we will periodically collect 12-lead ECG tracings and blood and urine samples in SPRINT participants to detect development of silent myocardial infarction (MI, or heart attack), and development or progression of chronic kidney disease, another important study outcome.

Collecting study outcomes data is one of the most critical aspects of SPRINT. These are defined in the protocol (Chapter 1). The primary outcome for the study is a composite, or combination, of major cardiovascular disease (CVD) events comprised of the first occurrence of:

- fatal or non-fatal myocardial infarction (MI, or heart attack),
- non-MI acute coronary syndrome (non-MI ACS, sometimes called unstable angina),
- fatal or non-fatal stroke,
- fatal or non-fatal heart failure (HF), or
- death attributable to cardiovascular disease (CVD).

The primary outcome of a study is used to calculate the number of participants needed for the study to test its main hypothesis. Thus, the aim of SPRINT is to determine whether the intensive BP treatment strategy will, when compared to a standard BP treatment strategy, reduce the number of new cases of serious cardiovascular events. Other important study outcomes will also be examined, including coronary revascularization (PTCA or angioplasty and CABG or bypass surgery), peripheral arterial disease (for example, carotid artery endarterectomy and abdominal aortic aneurysm repair), atrial fibrillation (a heart rhythm disorder), transient ischemic attack (TIA), and development of ESRD requiring chronic dialysis or kidney transplantation.

Outcomes assessment can be a lengthy process, and requires thorough collection of corroborating medical records by the Clinical Site staff at each site. The process culminates in the careful scrutiny of the materials (assembled into an adjudication case packet) by a team of central adjudicators who make the final outcomes determination.

Because SPRINT is not a masked trial, that is the participant and the clinic staff managing his or her blood pressure and collecting study outcomes information, are aware of the participant's randomization assignment, it is critical that sources of bias in outcomes ascertainment be

minimized. Bias is a systematic difference between study groups. This means that both the intensive and the standard BP treatment goal groups must have equal chances for reporting study outcomes, and that outcomes information is collected in the same way at the same visits in both groups. Because the intensive group will have more visits, generally PRN visits, for BP control, outcomes data must only be collected at the quarterly visits that are attended by both groups. Also, the same forms are used for outcomes data collection for both groups and must be administered exactly as written with no additional probes or information used for outcomes reporting in both groups so that outcomes data are collected in the same way in both groups.

13.2 ASCERTAINMENT OF STUDY OUTCOMES

Participants will be asked about potential study outcomes at each quarterly visit following randomization using the SPRINT Q3 Event Ascertainment Form. This form asks participants about potential outcomes of interest, including hospitalizations, outpatient procedures, and initiation of dialysis or transplantation, as well as the occurrence of potential serious adverse events (collected for safety assessments). The SPRINT Q3 Event Ascertainment form should be administered by a trained SPRINT staff member at the Clinical Site. The form asks about several types of potential study outcomes: hospital admissions, Emergency Room/Department visits for specific types of events (e.g., heart rate problems, heart failure, life threatening events); outpatient surgeries and procedures for problems related to the heart, brain, or circulation; kidney dialysis or transplantation. Other questions on the form collect information about admissions to rehabilitation centers/nursing homes, fainting, and falls, and are collected for other purposes, including safety monitoring.

Be aware that there is some unavoidable overlap in the collection of study outcomes data and in safety monitoring (the collection of serious adverse events, or SAEs). Some medical events or experiences will be collected for both reasons - as a potential outcome for outcome adjudication and as a serious adverse event for safety monitoring (e.g., death, or hospitalization for myocardial infarction or stroke), and is the reason why there are not completely separate forms for SAE and outcomes reporting. Outcome events are specific to SPRINT hypotheses and are only collected at the quarterly visits. SAEs, however, are defined the same way in all studies, and safety information is collected at any visit when a participant reports the occurrence of a potential SAE. Detailed information on safety monitoring and reporting is contained in Chapter 14 of the Manual.

In SPRINT, a hospitalization is defined as an overnight stay in an acute care hospital, for any reason. There is no minimum length of stay required beyond an overnight stay. Short stays, observation stays, and day surgeries may be referred to in medical records as outpatient visits; for SPRINT these stays are NOT considered hospitalizations unless they result in overnight stays at an acute-care facility due to a complication or need for close observation. Note that overnight stays in a rehabilitation facility are not considered an overnight hospitalization unless that facility is affiliated with an acute care hospital. Psychiatric admissions are not investigated or adjudicated in SPRINT, meaning Clinical Site staff are not responsible for collecting medical records from these types of stays. Transfers from one hospital to another, on the same day, are considered one case for SPRINT purposes, and if collection of medical records is required, records should be obtained from all facilities. In cases of transfer between acute care hospitals, the beginning date is the admission to the first hospital and the discharge date is the date of discharge from the last acute care hospital. Staff should obtain records from all acute care hospitals, so be sure to collect all the information you will need to collect the records.

For hospitalizations, record each hospital admission on a SPRINT Serious Adverse Event (SAE) Form. Previously reported hospital admissions will be pre-printed on the top of the Q3 Event Ascertainment Form, as will the date of the last Q3 Event Ascertainment Form, so that you will know if a given hospital stay was already reported or not. Do not use a list of SAEs reported between quarterly visits to help remind the participant to report them on the next Q3 Event Ascertainment Form. This could result in ascertainment bias and is not allowed. The information on the Serious Adverse Event Form for the event includes the name of the hospital where the participant was admitted, the dates of the admission and discharge, and the condition treated or reason for the admission. Make every effort to obtain accurate information for the hospital admission and dates to ensure that, if medical records are needed, they can be obtained in a timely fashion.

Emergency Room/Department visits for diagnosis and/or care of specific conditions will be collected, including heart rate/rhythm, heart failure, stroke, or other life threatening event. Heart failure in particular may be treated in the ED and may meet criteria for study outcomes, so medical record information is needed for these visits.

Outpatient surgeries or procedures for problems related to the heart, brain, or circulation are also potential study outcomes. If a participant reports one of these on the Q3 Event Ascertainment Form, complete one SPRINT Day Surgery or Procedure Form for each surgery/procedure that meets these criteria. This information will be needed to collect the necessary medical records.

Chronic kidney dialysis or transplantation for care of end-stage renal disease are also potential study outcomes. If the participant receives a kidney transplant, record this on the Serious Adverse Events form. If the participant has started dialysis and received dialysis for 30 or more days, complete the SPRINT Dialysis Form. These potential outcomes will require collection of medical records.

Death of a SPRINT participant may be discovered by the Clinical Site in several ways: during contact to schedule a study visit, a phone call to the Clinical Site staff by informants, during attempts to locate a SPRINT participant, or through other sources. Information from such reports should be used to complete the Serious Adverse Events Form. See Manual chapter 14 for information on completing the Serious Adverse Events Form. Of particular importance are the date of death and the city and state of death; this information is critical in obtaining the death certificate. Be aware that deaths should be reported to the Coordinating Center promptly in all participants, no matter when the Clinical Site staff become aware of the death. In other words, death events do not need to wait for the next quarterly visit to be reported.

It is critically important that potential outcomes be collected on all participants possible and that there be no differences in the procedures for collecting these outcomes between the study groups (intensive blood pressure control vs standard blood pressure control). In SPRINT, we have taken steps to help avoid bias in the ascertainment of study outcomes. Participants are only asked about potential study outcomes at each quarterly visit following randomization using the SPRINT Q3 Event Ascertainment Form. This is important because there needs to be an equal chance for all participants to have these outcomes reported. For study outcomes, report only those events that the participant tells you about when the Q3 Event Ascertainment Form is administered. In other words, only record study outcomes at the protocol specified quarterly visits. The Q3 Event Ascertainment Form should be administered verbatim, that is, word for

word. Staff should not introduce bias by prompting study participants to recall specific events about which they have knowledge because an SAE was reported between quarterly visits.

If a participant cannot attend a visit in person, you should take steps to collect information by phone, or possibly schedule a home visit to complete the Q3 Event Ascertainment Form and the SAE form (if needed).The first priority for any in-clinic, home, or phone visit is to complete the Q3 Event Ascertainment Form, and the SAE form, if necessary. If it is not possible to complete the Event Ascertainment Form or the SAE form with the participant (e.g. in a participant with severe speech problems due to a stroke or cognitive impairment), these forms can be completed by interviewing a proxy informant designated by the participant. Keep in mind that participants who are difficult to contact may have died or become institutionalized (e.g., placed in a nursing home). In such cases, use your information on friends, family, and other contact information you have to trace the participant. Helpful tips for finding participants are included at the end of this chapter. When a participant is incapacitated and unable to furnish this information, interview a family member or friend instead, if possible, and indicate this on the Q3 Event Ascertainment form and/or Serious Adverse Event form in the space provided. However, if the participant is well and able, using substitute interviewees should be rare. In general, substitute interviewees should be a last resort, used by the participant's permission, and named by the participant.

13.3 COLLECTION OF MEDICAL RECORDS

To ensure that the identified clinical outcomes represent true disease states, detailed outcomes ascertainment procedures and diagnostic criteria for adjudication have been developed by study investigators. Application of the diagnostic criteria requires that medical records be obtained and submitted to the Coordinating Center for adjudication by the SPRINT Morbidity and Mortality Committee.

13.3.1. Requesting Medical Release Forms

In order to obtain medical records from participants, each SPRINT Clinical Site should have the participant sign a "**Release of Medical Information**" form. This form should be developed by the Clinical Site in keeping with local institutional policies; however, a sample is included with this manual chapter for your use. SPRINT recommends that Clinical Sites obtain a signed Release of Medical Information form at the time of, and preferably before, randomization. Since many providers will accept these forms for the duration of a study, it will be very useful to have a signed form on file. Also, Clinical Site staff will be able to answer any questions about the procedure at the very beginning of their participation in the study. Participants who are reluctant to provide medical records to the study will affect the ability of SPRINT to answer the study hypotheses, because their study outcomes will not "count" unless those outcomes can be adjudicated. Also, reluctance to supply medical records may serve as a potential "red flag" that a potential participant may be an adherence or retention risk. Finally, be aware that some facilities will only accept a Release of Medical Information form that is fairly current, so you should obtain a signed Release at any visit when the participant reports a potential study outcome or serious adverse event that will require collection of medical records.

If a participant refuses to sign a Medical Release Form, you cannot request medical records for potential SPRINT outcomes. Try to probe for reasons why the participant refused. Explain to him/her the importance of the records for the study and why they are needed. Some participants may be willing to sign a release that is specific to information needed for a particular outcome. You should spend considerable time working with these participants to obtain

releases for major study outcomes (particularly stroke and heart attack), but you may choose not to pursue other outcomes (such as hospitalization for treatment of a fracture) and thus not annoy the participant unnecessarily. If the participant continues to refuse, note this in the participant's chart and continue to follow them according to the protocol if they will consent to contacts and/or visits.

13.3.2. Requesting Documentation

The investigation of potential SPRINT outcomes takes time, but is a critically important activity that involves locating relevant health care providers (e.g., hospitals, clinics, physicians) and requesting medical records that may support a diagnosis of an outcome. The documents requested from a particular provider will depend on the outcome type. Documents should be requested according to Table 1. The success of an outcome investigation (i.e., obtaining copies of required supporting documents) will depend on the expertise, resourcefulness, and communication skills of the Clinical Site staff. Institutional, local, and state regulations will also impact the ease and expense of completing an investigation.

SPRINT requires full investigation of the first and all subsequent outcomes of a certain type. This means that if a participant has repeated heart attacks, documents for each will need to be obtained. Another situation is the case of a participant who reports a complex medical history with several illnesses. Each separate hospitalization will be treated as a separate event, unless the participant is transferred from one acute care facility to another, and each occurrence of a potential SPRINT outcome will need to be investigated.

Table 1. Documentation requirements for SPRINT outcomes

	MI	ACS	CHF	PAD	Stroke/TIA	Outpatient revascularization	ESRD	Death
Discharge summary (dictated or handwritten)[1]	x	x	x	x	x	x	x	x
Operative or procedural report for treatment of disease	x	x	x	x	x	x	x	x
Outpatient Day Surgery, short stay				x		x	x	x
ER reports	x	x	x	x	x		x	x
Emergency Medical Service (EMS) or ambulance report	x	x	x		x			x
Admission and/or Outpatient History and physical (dictated or handwritten)	x	x	x	x	x	x	x	x
Physician Notes						x	x	x
12-lead ECG: All those done	x	x	x		x	x		x
Baseline ECGs	x	x	x		x	x		x
24M ECGs	x	x	x		x	x		x
Cardiac enzyme report (Lab)	x	x	x					x
PTCA report (angioplasty, cardiac stent, atherectomy)	x	x	x			x		x
Cardiac catheterization/ angiogram/ arteriogram report, contrast ventriculogram	x	x	x	x		x		x
Stress test by ECG, echo, or perfusion scintography report (with thallium , technetium or other isotope)	x	x	x					x
CABG report (operative note)	x	x	x					x
RVG or MUGA report2	x	x	x					x
Chest X-ray report	x	x	x					x
Echocardiography report			x		x			x
CT scan/CT angiography report	x	x	x	x	x			x
MRI report (magnetic resonance imaging)/MRA					x			x

(magnetic resonance arteriography)								
Ultrasound report				x	x			x
Doppler flow study report				x	x			x
Carotid studies (Doppler ultrasound, angiography etc)				x	x			x
Transcranial Doppler					x			x
Holter monitor					x			x
Autopsy/Medical examiner/coroner's report								x
Neurology consult notes					x			x
Nephrology notes							x	x
Death Certificate								x
Records from last 6 months of life								Out of hospital deaths ONLY
Dialysis notes							x	x

1 A final progress note or written discharge note may be substituted for the discharge summary for short stays (frequently less than 48 hours) or if typed discharge summary is not available.

2 RVG – radionuclide ventriculogram (usually done with cardiac cath) or MUGA – Multigate acquisition

A hospitalization, for SPRINT purposes, is defined as any overnight stay in an acute care hospital for any reason. There is no minimum length of stay required beyond an overnight stay. Short stays, observation stays, and day surgery may be referred to in medical records as outpatient visits, but for SPRINT these stays are only considered hospitalizations if they result in an overnight stay (due to a complication or need for close observation). Note that an overnight stay in a rehabilitation facility (not affiliated with an acute care hospital) is not considered an overnight hospital stay. Any overnight hospitalization for a psychiatric admission will not be investigated.

The medical record from a hospitalization consists of documents dating from the first health care contact for the event to the participant's death or discharge. If a participant is transferred from one acute care facility to another (e.g., to receive more intensive treatment at a specialty hospital), the entire stay at both facilities is considered one hospitalization. The physician adjudicator and the Clinical Site staff responsible for collecting medical records will need to be able to understand the course from admission to discharge or death to generally reconstruct the hospitalization and events.

The Coordinating Center will assist Clinical Site staff by providing a check list of the specific documents needed to adjudicate a possible outcome, based on the outcome type. This checklist should be included in the request to the provider. Once you mail the request to the provider, usually a hospital medical records department, it is their responsibility to find the relevant documents you requested. Be sure when medical records come in from providers that you check to see that your entire request has been filled. In some cases, you may have to follow-up with the provider to request missing records that are needed and requested but were not provided. Also be sure to read the information sent in response to the request; in some cases additional outcomes may have occurred that you were not aware of but that will require additional documentation requests to complete the package for adjudication. For example, if a participant reports a hospitalization for chest pain, you may only have requested documents for a possible MI. If the discharge summary also refers to heart failure, you should then obtain any additional documents needed for heart failure cases before sending the case in to the Coordinating Center.

Various medical record components and their contents may be needed to complete SPRINT adjudication case packets. If you collect documents directly from the medical records department, look in the indicated sections for the required documents. Be aware that some medical records may not be well organized, and documents may be scattered throughout the record. The list below is in the order that documents might commonly be found in a medical record, not in any specific order required for SPRINT. Do not routinely add additional documentation to your document requests or adjudication packets; there are privacy rules against such practices. Select the appropriate documents from Table 1 – documentation requirements for SPRINT outcomes. Several items require some clarification and are thus defined below.

- Discharge Summary – Narrative summary of entire hospital course, including reasons for admission, significant findings, procedures performed, treatment(s), patient's condition on discharge, and any specific instructions given to the patient and/or family. The discharge summary is one of the most important documents for adjudicating any SPRINT outcome. A final progress note, discharge note, or the hospital face sheet may be substituted for the discharge summary for short-stays (i.e., events or procedures that require less than a 48-hour hospital stay).

- Operative Reports – Surgical reports for coronary bypass graft (CABG), percutaneous transluminal coronary angioplasty (PTCA), carotid endarterectomy, aortic aneurysm repair and other procedures are often found in this section. These reports may also be interspersed throughout the record.

- Ambulance Report or Emergency Room Report – Description of symptoms, initial treatment en route to the hospital, vital signs, dates and time of symptoms, treatment, responses to treatment, and disposition. This report is most useful for patients who were dead on arrival at the hospital or for those dying in the ER before admission.

- Admission History and Physical Exam (H&P)/Outpatient H&P – Detailed description of symptoms leading to admission, condition of the patient on admission, medical history, review of systems, vital signs, medications before and at admission, provisional diagnoses, and treatment plan. This document is very helpful for determining symptoms on admission, needed for acute coronary cases (MI and ACS), for stroke and TIA, and for deaths occurring during the hospital stay. Attending physicians at teaching hospitals may refer in their H&P to sections of resident admission H&P rather than reiterate historical and/or exam findings. If a complete physical exam has been performed within 30 days before admission, such as in a physician's office, a copy of that report may be the only H&P in the patient's hospital chart (provided there had been no changes or the changes had been recorded at the time of admission).

- Neurology Consultations/Nephrology notes – Neurology consultation reports are needed for stroke/TIA cases only. They will assist the adjudicators by documenting the symptoms, physical exam findings, and their time course that will assist the SPRINT adjudicators. These will be found in a separate section of the chart with typed or handwritten notes from the consulted medical or surgical specialists, in notes interspersed through the records, or in the progress notes. Nephrology notes from the inpatient or outpatient setting, depending on in what setting the participant started dialysis will assist the SPRINT adjudicators to determine if the case meets criteria for end-stage renal disease, or is limited to acute kidney injury/acute renal failure only.

- ECGs (electrocardiogram) – 12-lead ECGs performed during the hospitalization are often contained in a separate section of the chart, but may also be interspersed with other documents, such as progress notes. Only 12- lead ECGs are required for SPRINT cardiovascular outcomes, not the individual rhythm strips (one to three leads) that might be found attached to daily progress notes. You should request all ECGs from each hospitalization. ECG dates and times are crucial during adjudication. If ECG dates and times are located near the edge of the page, handwrite them in a more central location of the page. Any information on the edge of the page has a risk of being cut off during duplication. Cardiac cases received at the Coordinating Center with undated ECGs will be put "on hold" while we contact Clinical Site staff and wait for receipt of the required documents or information.

- Laboratory Results – Standard blood and other specimen analysis results, for example cardiac enzyme (troponin or CK) results for MI, BNP, creatinine, and hemoglobin reports for heart failure, are usually found in this section. Laboratory results may be interspersed with other documents. If a required test is noted pending in the medical records, be sure to go back and request the results if missing.

It is not uncommon for cardiac enzymes to be recorded on a separate lab sheet. Try very hard to get the actual lab reports rather than relying on values stated in the discharge summary or

physician notes. If not in the chart, try to get them from the lab. If staff absolutely cannot get the values after repeated attempts, obtain the lab's normal ranges for cardiac enzymes contained in the record and make a note of them in the adjudication packet. Since different assays with different normal ranges are used for the important lab tests SPRINT adjudicators are using to classify cases, it is critical that normal ranges be included in the adjudication case packet.

- Diagnostic or Radiology (including Nuclear Medicine) Procedures – Chest x-rays, stress tests, CT scans, MRIs, echocardiograms, coronary angiograms (heart catheterizations), doppler flow studies, autopsy reports, and all other diagnostic procedures are often found in this section. These reports may also be interspersed in the medical record. For example, coronary angiogram reports may be found in the progress notes. Be sure to obtain radiology records for IV Persantine- Thallium stress tests (a test for patients who cannot exercise on a treadmill) or other stress tests with an imaging component (e.g., SPECT) in addition to the ECG portion of the test. The final impression of the EKG report will say "await radiology report" or something similar. It is important to look for phrases like this and then make sure the report is included.

Every effort should be made to obtain all possible records pertinent to a particular type of outcome. If for some reason a particular document is not obtainable, the reason for its absence should be noted on the checklist. The reasons for their absence may include the fact that the test or procedure was not done. Knowing that these records are not obtainable will assist your decision on whether to complete a case and forward it to the Coordinating Center for adjudication. Thus, the absence of a particular document should not unduly delay submission of the other information for adjudication. You must strike the proper balance between timeliness and completeness. Case packets should not be submitted piecemeal. Rather, all required documents should be collected and submitted at once, along with a completed checklist specific for the outcome(s) contained in the packet.

For each outcome, there are "essential" documents. These records are more important than others, and are the most critical required to adjudicate an outcome. For most outcomes in SPRINT, essential records will include the <u>discharge summary</u> for those cases occurring during an overnight hospitalization because the information on the presenting symptoms and physical findings, a summary of test results, and how the patient responded to treatment is included. Essential records for specific potential outcomes also include:

MI: cardiac enzymes (e.g., CK, CK-MB, troponin), hospital ECGs, diagnostic procedure reports (CABG, PTCA)
CHF: Chest x-ray, cardiac imaging (e.g., echo)
Death: death certificate
Coronary revascularization: procedure report
Stroke: MRI or CT report, carotid studies (e.g. Doppler)
PAD: Procedure report
ESRD: procedure report (transplant), dialysis notes

If two potential SPRINT outcomes are included in one packet (i.e., from the same hospitalization there is both an MI and a stroke), you do not need to make two copies of the documents. However, be sure to check that all necessary documents for all possible SPRINT outcomes are in the packet; the physician adjudicator can adjudicate all outcomes in a packet at one time. You will be notified if additional documentation is needed.

Case packets will be uploaded to adjudicators through the Coordinating Center. The preferred method of submitting records is through the Outcome Tracking system on the SPRINT website. In the rare case that this is not possible, records should be submitted in a scanner/copy-friendly manner with the PID on every page. If mailing, each packet should be bound with a single binder clip (or paper clip). Do not use Post-it Notes on cases, as they often cover pertinent information. Instead, make notes on the Materials checklist or include a separate sheet of paper with notes.

Again, every effort should be made to obtain all possible records pertinent to a particular type of outcome, but most certainly to obtain the minimum (essential) documentation needed for adjudication.

13.3.3. Summary of Process for Requesting Records

When requesting medical records, the Clinical Site staff should proceed as follows:

1. Identify all appropriate providers (sources of documents) from the pertinent forms, including the SPRINT Q3 Event Ascertainment, Serious Adverse Event, Dialysis, Day Surgery or Procedure Forms, and the death certificate, if appropriate.

2. Prepare a Request for Medical Record Information for each provider. You must complete other relevant information including the participant's name, ID number, date of birth, social security number, date of admission/care, requesting SPRINT Clinical Site, and staff ID number. Include the participant's signed Medical Release Form, or for death, a release signed by the next-of-kin or other acceptable party (e.g., executor).

3. Mail a request to each provider for each identified outcome.

4. Create an outcome file for each participant with an identified outcome. Keep it separate from other SPRINT participant charts, ideally in a designated location for outcomes information, and identified on the return address for receipt of records. Information and documents in this file will be used to assemble the adjudication case packet. The adjudication case packet will contain the required subset of the participant outcome file documents that are appropriate for the outcome being investigated.

5. Every attempt should be made to obtain the minimum documentation for adjudication. If some requested documents cannot be obtained after diligent effort, their absence should not unduly delay the submission of the other information for adjudication. However, their absence must be documented on the checklist for that outcome with the reason why they could not be obtained.

6. Receive requested documents. Match documents you receive with the Request for Medical Record Information Form that you submitted to each provider. Match demographic data from the medical records to study data to ensure accurate identification of a participant.

Once you are sure you have correct information on the participant and have obtained all documents, attach a SPRINT ID label to each document, ideally on each page.

Make a copy of the documents and, on the copy, mark out identifying information (e.g., name) where it appears on each page of a document that will be submitted for adjudication. You

should maintain the original documents with identifying information in your outcomes file. This step is very important, and you should look hard for anything that is protected health information (PHI) such as name, social security number, telephone number, medical record number etc. Be sure that the PHI cannot be read on the copy.

Clip together documents that will be required for adjudication of a particular outcome.

7. Extraneous documents are sometimes included when records are sent by a provider. Documents that are not required for adjudication should be placed in the file and destroyed in a confidential manner (i.e., shredded) when the adjudication case is closed. You should not request entire medical records to avoid excessive accumulation of documents and breaching participant confidentiality. If you are unsure whether a document is appropriate or may be needed for adjudication, or if you have other questions regarding the outcomes process, ask your PI or contact Loretta Cloud at the Coordinating Center.

8. Follow-up on any missing records that were requested, but were not received. This is unfortunately common, but a necessary step. Be sure to fully document any follow-up calls or correspondence with providers (e.g., name, date, document requested, result) so that you can at any time reconstruct the steps you have already taken to locate records. Do not trust your memory.

9. Progress regarding obtaining medical records should be monitored by Clinical Site staff. You may want to develop a tracking system for all reported outcomes, noting which documents have been requested, which received, and when follow-up requests need to be made (requests outstanding for more than four weeks).

Two reports, one internal and one external to the Coordinating Center, are available for monitoring:

- External - The Outcome Event Tracking System lists all outcomes with any documentation outstanding, including follow-up requests by the adjudicators. The report lists ID, type of event, event date, all documents requested for that event and whether they were received. Once events have passed the 90 day mark an indicator will turn red to indicate they are overdue. There will also be an area for the Coordinating Center to post comments for the clinical sites, and for clinical sites to post comments for the Coordinating Center.
- Internal - The Event Summary report will be used internal to the CoC. It encompasses all items in the Outstanding Event Documentation Report, plus adjudication information.

13.3.4 Some Special Considerations in Requesting Medical Records Documents

Following are special considerations regarding medical records request:

- Safety outcomes – visits to Emergency Room/Department including those for injurious falls, syncope, arrhythmia, electrolyte abnormality will be specifically ascertained as they may be related to SPRINT interventions and may be considered Serious Adverse Events (SAE). These visits and potentially other hospitalizations not related to treatment for study related outcomes (eg, not for treatment of MI or stroke) but thought related to the SPRINT intervention may require collection of medical records. Clinical sites will be informed of whether records are required for a specific SAE by the SPRINT coordinating center.

- Death certificate – A copy of the death certificate is required to confirm a death. Other documents may be required, such as autopsy report or medical records from personal physician. Death certificates may be obtained from State or City Vital Records Departments. Usually this requires filing an IRB application with the agency. Once approval is obtained, it generally does not need to be repeated with each case. If conflicting information is obtained regarding the date of death, use the information from the most reliable source (hospital records or death certificates are considered more reliable than informant reports).

- Merging adjudication case packets – Each hospitalization or other provider visit (e.g., outpatient revascularization procedures) should be a separate adjudication case unless:

1. a second hospitalization represents a transfer on the same day from the first hospitalization or
2. outpatient diagnostic procedures (e.g., exercise test) are conducted preparatory to a hospitalization for further work-up of cardiovascular disease.

13.3.5 Additional information for out of hospital deaths

Out-of-hospital death – For all out-of-hospital deaths, request and submit documentation for care received in the last 6 months of life, such as a hospitalization 1 month prior to the death. Also, include a copy of the Serious Adverse Event form and the death certificate.

Participants who die out of the hospital, particularly those who die fairly suddenly and without a known history of serious disease, can be difficult cases for adjudicators to assign a specific cause of death. Out of hospital death includes participants who die in the ER, who die at home, or in a setting outside of an acute care hospital. In these cases, Clinical Site staff can provide critical information to the adjudicators by conducting an informant interview with a knowledgeable family member or friend.

SPRINT clinical center staff members will perform a brief interview with either next-of-kin or contacts provided by the participant for this purpose. This interview will gather information essential for adjudication. Elements will include:

1. Where did the death occur? (out of hospital, nursing home, emergency department, etc.)
2. Presence of symptoms prior to the death (e.g., angina, chest pain or discomfort, increased fatigue or tiredness, shortness of breath)?
3. Was death sudden and unexpected? (definitely, possibly, no)
4. Was death witnessed? (If not, were they found in bed or a chair?)
5. Time from onset of symptoms (or last seen) to death, in hours

6. Was there a hospitalization or emergency department admission or doctor visit either immediately before death, or since the last follow-up contact?

13.4 LOST PARTICIPANTS

As discussed in Chapter 11 (Procedures for Inactive/Lost/Refused Participants and Missed Visits), use the Participant Status Log to document participants who have missed their last two consecutive visits and cannot be contacted at last known phone number or address, or through relatives, neighbors, or other contacts. Use Part B of the form to record the appropriate information to document contact attempts.

As part of the efforts to contact lost participants, it is highly recommended that SPRINT Clinical Site staff perform vital status searches annually if you have not had contact with a participant in the previous 12 months. These searches should use all available resources, not only contacting participant's friends or family members listed on their contact sheet, but also searches of the Social Security Death Index.

13.5 PREPARING ADJUDICATION CASE PACKETS

Once all documents are received, assemble them for adjudication in the order below (noting that not all documents listed will be requested or available for each case). Review the records carefully to see if a missed outcome is referred to in the records either during or before a particular hospitalization. Date order ECGs before submitting cases for adjudication.

- Materials checklist for the outcome, noting when and why items are not available
- ER notes
- Admission History and Physical
- Operative Report
- Diagnostic Procedure Results
- Laboratory Results
- Outpatient/Short Stay Records
- Discharge summary
- Death Certificate
- Autopsy Report

Be sure to place a participant ID label on each document, and preferably on each page. Make a copy of the entire adjudication case packet. Make sure that you have removed identifying information from the copy (i.e., blocked out with correction tape, china marker, or other means) before sending it to the Coordinating Center, as sending records with identifiers may be a HIPAA violation. Identifying information includes a participant's name, address, phone number, social security number, account numbers, next of kin information, etc. Do not obscure dates of hospitalizations, ECGs, labs, or other reports. These dates are critical in determining that all data are associated with the correct hospitalization or potential study outcome. Send the copy to the coordinating center (specifics listed below). Please limit the use of faxes to brief case packets.

Mail to:

Loretta Cloud, BA, CCRP and
Marjorie Howard, BA
SPRINT Coordinating Center
Wake Forest University Health Sciences
Department of Biostatistical Sciences – WC21
Division of Public Health Sciences
Medical Center Blvd.
Winston-Salem, North Carolina 27157

Fax brief reports:
Fax: 336-713-5308

Scan and email:
lcloud@wakehealth.edu
mhoward@wakehealth.edu

Medical Abbreviations and terms

Angina Pectoris = Chest Pain / Heart Pain

Anticoagulation

Atherectomy

Bronchoscopy

CABG = Coronary Artery Bypass Graft

CAD = Coronary Artery Disease

Cardiac Enzymes (CK, CK-MB, Troponin, Myoglobin)

Catheterization = Cardiac Cath

Chest x-ray

CHF = Congestive Heart Failure

Coronary Stenting = Stent

CT = Computerized Tomography (CAT scan)

CVA = Cerebrovascular Accident (Stroke)

CVD = Cardiovascular Disease

Dialysis

Diuresis

Doppler Ultrasound

ECG = Electrocardiograph (EKG)

ECHO = Echocardiogram

Edema

Ejection Fraction = EF

ESRD = End stage renal disease

ETT = Exercise Tolerance Test

Fracture = Fx

IVUS = Intravascular ultrasound

LP = Lumbar puncture

Malignant = Cancer (adenocarcinoma)

Metastasis

MI = Myocardial Infarction (Heart Attack)

MRI = Magnetic Resonance Imaging

Neoplasm = New growth (lipoma)

PAD = Peripheral Artery Disease

Includes surgery, angioplasty, or thrombolysis for peripheral vascular disease (including renal artery). It does not matter in what setting the procedure was done (inpatient or outpatient).

Pathology and Cytology Reports

PTCA = Percutaneous Transluminal Coronary Angioplasty (Balloon Angioplasty)

Pulmonary Edema

SOB = Short of Breath

TIA = Transient Ischemic Attack

US = Ultrasound

LOST PARTICIPANT RESOURCES

a) Tips for locating participants
   i) Each case is different and may require different actions
   ii) Look for clues in recent hospital records (e.g., was participant discharged to a nursing home or other medical facility?)
      (1) Online resources for locating medical facilities
         (a) The American Hospital Directory - www.ahd.com/
         (b) Medical facilities in the US and abroad - http://www.hospitalsoup.com/hospitalsearch.asp
b) Resources on the World Wide Web:
   i) www.ancestry.com/
      (1) This is a search by name and provides general information for a fee. See website for details.
      (2) Annual membership is $14.95 per month, billed in annual payments of $179.40, as of 12/30/2005
      (3) Monthly membership is $23.95 per month, billed in monthly payments, as of 12/30/2005
      (4) Be sure to read the "Terms and Conditions"
   ii) www.ancestry.com/search/rectype/vital/ssdi/main.htm
      (1) This is a Social Security Death Index (SSDI) search tool and is part of ancestry.com listed above. It is purportedly the most up to date and powerful SSDI available on the internet.
      (2) Search will provide minimal information free of charge, but you will need further information to verify that the information provided is for the correct person.
      (3) Index is purportedly updated monthly (other SSDI are updated less frequently).
      (4) Paid membership information is as indicated above for ancestry.com.
   iii) http://ssdi.genealogy.rootsweb.com/cgi-bin/ssdi.cgi?o_xid=0031936443&o_lid=0031936443&o_xt=31936443
      (1) This is another SSDI search option and will provide DOB, date of death, last residence, and social security number for decedent free of charge.
      (2) Index purportedly updated semi-annually.
   iv) http://www.ancestorhunt.com/prison_search.htm
      (1) This is a prison inmate search. Scroll down to select state and begin search. Search will provide DOB, picture, location, sentence, etc. free of charge for some states. Other states may provide instructions for verifying that a person is incarcerated.
   v) http://www.ancestorhunt.com/county-jail-inmates-search.htm#County%20Jail%20Inmates%20Search%20by%20State
      (1) This is a county jail inmate search and is not provided by all counties.
      (2) You may also call the county jail of interest and inquire by telephone.
   vi) http://www.clearhq.org/boards.htm
      (1) This is a licensure, enforcement, and regulation search. If the participant's profession requires a professional license or certification regulated by the state, you can check the status for most states. This information is free of charge.
      (2) The search requirements vary according to state and profession, so read instructions carefully.

UAB SCHOOL OF MEDICINE
Department of Medicine.Division of Preventive Medicine
1530 3''' Avenue South. MT 700,Birmingham. AL 35294-4410

Phone:(205) 9342294
Fax: (205) 934-1851

AUTHORIZATION TO OBTAIN MEDICAL RECORDS (Protected Health Information)

By signing this document. I authorize the release and disclosure of all of *my* medical information/protected health information (PHI), whether contained in my medical record or otherwise, by the health care facility listed below, including its doctors. nurses or staff. I further authorize the University of Alabama at Bimn1ngham Division of Preventive Medicine and the research staff involved to use or disclose for the purposes described in my original consent to participate in the CARDIA Study and as stated below. This authorization has no expiration date. My signature is further acknowledgement that I have read and understand this document. I also agree to pemnit my doctors and other health care providers. including those listed below. to disclose PHI to these Researchers for the purposes in this accompanying request.

Participant Name: —————— ———— ——— — ———

Date of Birth: ____________________

Relationship: ____________________

legal representative Name: —— ———— —— — ———

Participant Social Security #:

Reports requested inclusive for procedures or hospitalization From _____/_____/_____ To ______/_____/_____

Name of Hospital/Doctor	Name of Institution	Full Address/Phone

Initial

Initial

Initial

Right to Revoke: I understand that I may revoke (take back) this authorization at any time by submitting my written request to Cora E. lewis, MD, at the above address. I also understand that my revocation will not affect any actions taken by CARDIA researchers in reliance upon this authorization before it was revoked.

Right not to Sign: I also acknowledge my right not to sign this document but that my refusal to do so will not prevent my participation in the CARDIA study.

Re-disclosure: I acknowledge that there is a potential risk that PHI will be re-disclosed by the recipient and no longer protected by the Privacy Rule. However, I understand that when a research study is the recipienit is required by law to de-identify (remove personal identifiers) before re-disclosing for research purposes. Further infomnation on confidentiality of study records is covered in mv study consent form.

TYPE OF RECORD REQUESTED

- O Electrocardiogram
- O Cardiac Cath/Angiogram/ Arteriogram
- O Venogram
- O Facesheet
- O Physicians Attestaion -Coding Abstract
- O Operative or Procedure Report
- O ER Report
- O Transfer Record
- O PTCA, Stent, Artherectomy Report
- O Other
- O Outpatient Record
- O Consun (Oncology/RadiOlogy)
- O Radiology Scan/Bone Scan
- O LabERNPRA
- O X-ray Report= --.,..-
- O Cardiac Enzyme Lab Report
- O Coronary Artery Bypass Graft
- O RVG or MUGA
- O Pathology Report
- O
- O Stress Test
- O CT Scan and/or MRI Report
- O Carotid Studies
- O Pulmonary Angiogram
- O Echocardiograph Report
- O Lumbar Puncture
- O History and Physical
- O Death Certificate
- O Discharge Summary
- O

I authorize all health care facilities to accept a photocopy of this document as my official consent to release medical records.

Those who may have access to and review this information include the Division of Preventive Medicine CARDIA Study Researchers and Staff. You may RELEASE this INFORMATION and records obtained as a part of my CARDIA research participation to:

Name of person/organization UAB Preventive Medicine/ Cora E. Lewis, MD, MSPH, Principal Investigator

Signature of participant or legal representative ____________________ Date ____________

Chapter 14. Safety Monitoring and Reporting

14.1 Overview

The SPRINT trial is testing whether lowering SBP to a goal of <120 mm Hg results in better outcomes than a goal of <140 mm Hg in patients at risk for CVD events. SPRINT is not a study of specific anti-hypertensive agents. All antihypertensive agents provided by the trial or recommended by SPRINT have been approved by the Food and Drug Administration (FDA) and are routinely prescribed for lowering blood pressure.

Patient safety will be carefully monitored in SPRINT. Each clinical site investigator has primary responsibility for the safety of the individual participants under his/her care. In addition, an independent Data and Safety Monitoring Board (DSMB) will have primary responsibility for monitoring the accumulating study data for signs of adverse trends in morbidity/mortality and treatment-related serious adverse events.

Management of adverse events, or potential side effects, is based on the philosophy of protecting the safety of the participant, while at the same time making every effort to adhere to the treatment programs. In those instances where deviations from the treatment protocol are necessary in the judgment of the clinic physician, these deviations should be as minimal as possible. Suggested approaches to some of the more notable potential problems are specified in some detail. Other, less common problems are not described, reflecting the philosophy that each clinic physician will need the flexibility to use his or her own judgment for handling the wide variety of situations that may develop, in a way that will maintain both the safety of the participant and the integrity of the trial.

14.2 Expected events

The potential adverse effects of the blood pressure drugs used in SPRINT have been well documented. For example, electrolyte abnormalities (hyponatremia or hypokalemia are known to be associated with diuretics; hyperkalemia and short-term decline in GFR with RAAS blockers, hyperkalemia with potassium-sparing diuretics); bradycardia with beta blockers and non-dihydropyridine calcium channel blockers, and orthostatic hypotension with any antihypertensive. Expected events are not considered serious adverse events (SAEs) unless they meet criteria for an SAE (see 14.3). However, Clinical Safety Alerts are provided to the site PI for his/her review and action in order to enhance management and safety of participants (Table 14.1).

Table 14.1 Clinical Safety Alerts

**Measure**	**Alert Value**
Serum sodium	<133 or >150 mEq/L
Serum potassium	<3.0 or >5.5 mEq/L
Serum creatinine	Increase by at least 50% to a value >=1.5 mg/dL since the last study lab
ECG	acute ischemia, acute MI, complete heart block, ventricular tachycardia, heart rate <40 or >120 bpm, new atrial fibrillation
PHQ-9 (depression screen)	Suicidal ideation (score 2 or 3 on question 9) or PHQ-9 total score >=15
Dementia Assessment	Adjudicated dementia

14.2.1 Laboratory studies

A chemistry profile will be drawn on all participants for safety at months 1, 3, 6 and every 6 months thereafter. When any laboratory measurement attains the defined alert level, the Central Laboratory will immediately notify the clinical site and the CCN. Site clinicians may also obtain local labs if safety is a concern at non-scheduled intervals. Site clinicians are responsible for timely review of all labs drawn locally and when central lab results become available.

14.2.1.a As needed safety labs for RAS blockers and diuretics

RAAS blockers (ARB or ACE inhibitor) and diuretics can result in clinically significant alterations of serum electrolytes and renal function. Thus, after the initiation of a RAAS blocker or diuretic, a chemistry profile should also be obtained according to table 14.2 (based on the National Kidney Foundation Kidney Disease Outcomes Quality Initiative (KDOQI) guidelines).

Table 14.2. Recommended timing of safety labs

		eGFR ≥ 60	30 ≤ eGFR < 60	eGFR < 30
RAAS	Initiation*	1 month	1 month	≤ 2 weeks
	First dose increase	1 to 6 months	1 month	1 month
Diuretic	Initiation*	1 month	1 month	1 month
	First dose increase	1 to 6 months	1 month	1 month

For most participants, initiating or titrating up a blood pressure medication will result in a PRN visit in 1 month per protocol. Thus, the chemistry panel will typically be drawn at the next PRN visit. Participants who have an eGFR <30 ml/min/1.73m^2 and are started on a RAAS blocker, will need to return within 2 weeks for a "labs only" PRN visit. Similarly, in the standard care group there may be participants who do not meet criteria to return in 1 month for a PRN visit after initiation or titration blood pressure medication (see Figure on Treatment Algorithm in Standard Group), and they should be asked to return in 1 month for a "labs only" PRN visit.

* Note: If a participant is on a RAAS blocker or diuretic that needs to be changed to one in the SPRINT formulary, this is not considered initiation. It may be considered a dose increase if the SPRINT drug is given at a higher dose equivalent than the participant had been on.

14.2.2 Electrocardiograms

ECGs will be done at specified visits and read by the ECG reading center. However, before the participant leaves the clinic, a site staff member must review the printed diagnostic statement on top of the ECG printout. If any of the following are noted, the ECG must be reviewed by the site PI or designated clinician before the participant leaves the clinic:

a) Heart rate < 40 beats/minute
b) Heart rate >120 beats/minute
c) Acute myocardial infarction
d) Acute ischemia
e) Ventricular tachycardia
f) Complete atrioventricular block
g) Atrial Fibrillation*

* If the ECG tracing reports Atrial Fibrillation, ask the participant about his/her history of Atrial Fibrillation. If the Atrial Fibrillation is NEW, the PI or designated clinician should be notified.

See MOP Chapter 8 for more details.

14.2.2.a SPRINT Process for Notifying Participants of Silent MI Findings
ECG abnormalities at baseline and changes from baseline to scheduled follow-up ECG recordings have been shown to be independently predictive of future cardiovascular disease morbidity and mortality (1, 2). In SPRINT, a 12-lead ECG is obtained at baseline and at the 24 month, 48 month, and close-out visits to ascertain the occurrence of silent MI. Silent MI is defined in SPRINT as the appearance of significant serial QRST changes in the follow up ECGs indicative of new myocardial infarction and ischemia according to the standard Minnesota ECG Classification (3) **and** the Coordinating Center has no history or record or a clinical MI prior to the evidence on a follow-up ECG. Many of these serial changes are subtle and therefore they may not be observed by the study physician at the time of the SPRINT study visit. However, the participant and/or site may be aware of a clinical MI which has not yet been reported as an SAE or outcome to the Coordinating Center.

SPRINT Central ECG Reading Center (CERC) Responsibility:
The SPRINT CERC is responsible for the review and comparison between study ECGs and with the identification of new MI using the standard Minnesota ECG Classification. The CERC will provide the SPRINT Coordinating Center a monthly report which includes a listing of participants that have ECG criteria for MI which is new compared to previous ECG. The Coordinating Center will cross-check this list biannually with reports of clinical MI events and will generate a list of participants with potential silent MIs.

SPRINT Coordinating Center Responsibility:
Twice a year, the SPRINT Coordinating Center will notify clinics of participants who have ECG evidence of a new MI in the absence of a clinical event known to the Coordinating Center.

- The Safety Officer or designee will provide the site PI and coordinator a list of participants at their site that have met the criteria for a silent MI. The CCN PI and Coordinator will be copied on this correspondence.
- A Coordinating Center IRB approved letter informing the participant and the participant's PCP (if the participant has provided permission) about the silent MI finding will be available on the website (Documents>Survival Kit>Chapter 5 – Safety and Outcomes Tools) and should be downloaded and personalized for each participant on the list. The local site is responsible for obtaining local IRB approval of the letter.

SPRINT Clinical Site Responsibility:
The SPRINT clinical site is responsible for notification of participants and their PCP (if permission is granted) when the Coordinating Center has alerted the site of a possible silent MI. The site should provide the letter and copies of the two SPRINT ECG tracings (most recent ECG and last recorded study ECG where the change was detected) to the participant and PCP.

It is the clinical site PI's decision about the best way to communicate with the participant and the PCP. For example,

- The site can send the letter to the participant; or
- The site can discuss the finding with the participant in person and give them the letter.
- Sites should document when and how the participant was provided this information. A copy of the letter and ECG documents should be placed in the source documents.
- Copies of the letter and ECGs should be given to the participant to take to his/her primary care physician; or with permission, they can be mailed to the participant's PCP.

It may be helpful (and is suggested) for the SPRINT site PI to call the PCP in these circumstances to discuss the findings and decide between the two who will approach the

participant with this information and how. It is generally left to the PCP to decide whether further studies and/or treatment changes are needed based on this finding of silent MI.

14.2.3 Depression

The Patient Health Questionnaire-9 (PHQ-9) is a self-reported measure of depressive symptoms and is administered annually. Before the participant leaves the clinic, site staff should review the form with particular attention to question 9 ("Thoughts that you would be better off dead or of hurting yourself in some way"). If the response to question 9 is "more than half the days" or "nearly every day", then the PI or designated site clinician needs to assess the participant for risk of suicide before he/she leaves the clinic. Per the clinician's judgment, the participant may be safe to leave the clinic with follow-up from either a PCP or mental health professional or require further assessment and treatment emergently. Staff is required to document that action was taken by the PI or site clinician prior to the participants leaving the clinic. This is documented on the Encounter and Disposition Form in Part C, Protocol-Specified Visit by answering a series of questions in Question 6. Sites indicate that action was taken by answering the two following questions: "Was the response to PHQ-9 Question 9, 'more than half the days' or 'nearly every day?' (yes or no) and if yes, "Did the PI or a designated site clinician assess the participant for risk of suicide before the participant left the clinic?" (yes or no).

For urgent, but non-emergent, depressive symptoms, staff are notified of a PHQ-9 positive depression screen (PHQ-9 score>=15) immediately after data entry of the form on the SPRINT website. Sites may choose one of the following actions in response to the alert within one week:

- Notify the participant's physician (if the participant has given permission to do so) that he patient may have depression and further evaluation and treatment may be indicated;
- Make an appointment for the participant with an appropriate mental health provider (e.g. a psychiatrist);
- Elect to evaluate and treat the participant at the clinical site;
- Or other actions that are in accordance with the clinic's standard practice regarding urgent mental health concerns.

Sites are alerted by email when a PHQ-9 form is data entered on the SPRINT website and the answer to question 9 is 'more than half the days' or 'nearly every day' and when the total score is greater than or equal to 15. Sites are also notified on the cover page which is printed at each participant visit if a participant has had a positive depression screen and/or suicidal ideation. Sites can review PHQ-9 alerts in a report on the SPRINT website which is located in Reports>Clinical Operations>PHQ-9 Alerts. CCN coordinators can review a report of all PHQ-9 alerts within their CCN at Reports>CCN Coordinators>PHQ-9 Alerts-CCN. Documentation of actions taken should be placed in the participant folder.

14.2.4 Incident Dementia

If a participant is adjudicated to have incident dementia, site PIs will be notified by the coordinating center. While this is not an "urgent" problem, the participant's PCP should be notified as further testing and/or treatment may be indicated. In addition, the information may be helpful to clinic staff in maintaining compliance. A standardized letter regarding the results is

provided by the CC for the site PI to use in notifying PCPs if he/she desires (web site location: Documents > MIND and Documents > Study Documents).

14.3 Adverse Events and Serious Adverse Events

An adverse event (AE) is defined as any untoward or unfavorable medical occurrence in a human subject, including any clinically significant abnormal sign (for example, abnormal physical exam or laboratory finding), symptom, or disease, temporally associated with the subject's participation in the research, whether or not considered related to the subject's participation in the research. The burden of collecting and reporting data on every possible AE in SPRINT is excessive and side effects from the drugs to be used in SPRINT have been well defined in previous studies. Therefore, in SPRINT, sites will report all serious adverse events and selected AEs on the SAE form to the Coordinating Center.

Consistent with NHLBI guidelines and OHRP policy, SAEs are adverse events that meet any of the following criteria:

- fatal
- life-threatening (places the participant at immediate risk of death from the event as it occurred);
- result in significant or persistent disability;
- requires inpatient hospitalization or prolongation of existing hospitalization;
- are important medical events that investigators judge to represent significant hazards or harm to research participants and may require medical or surgical intervention to prevent one of the other outcomes listed in this definition (e.g. hospitalization, death, persistent disability) and may require medical or surgical intervention to prevent one of the other outcomes listed in this definition (e.g. hospitalization, death, persistent disability).

Any adverse event that meets any of these criteria should be documented and reported as a serious adverse event.

14.3.1 Hospitalizations

In SPRINT, a hospitalization is defined as an overnight stay in an acute care hospital, for any reason. There is no minimum length of stay required beyond an overnight stay. This includes planned hospitalizations for elective procedures (such as knee replacement surgery). "Observation" admissions (usually <24 hours) are also counted as admissions as long as the participant was moved out of the emergency department. Spending the night in an emergency department is not considered an overnight admission. An individual is considered in-patient if he or she has been formally admitted to the hospital with a doctor's order. The doctor's order should be supported by an admission history and physical. If there are documents titled "history and physical" and/or "discharge summary," the stay was an admission. Absence of these documents does not preclude an admission, especially absence of a discharge summary.

The following example is provided for additional clarification: A participant went to the ER shortly after midnight and was admitted for ACS rule-out. The participant was discharged on the same calendar date at 5:00 p.m. Although the participant was in the hospital for less than 24 hours, this event is considered a hospital admission because he was admitted overnight for observation. SPRINT does not require "overnight" to include midnight.

14.3.1.a Admission to Physical Rehabilitation Center: For the purposes of SAE reporting, an admission to a rehabilitation center is not considered a hospitalization. Since the vast majority

of admissions to inpatient, subacute, or skilled nursing facility for physical rehabilitation are associated with an admission to an acute care hospital, the SAE event will be captured in the report of the hospitalization. Thus, an admission for inpatient physical rehabilitation does not need to be reported in a separate SAE form.

14.3.1.b Admission for Drug or Alcohol Rehabilitation: An admission to a rehabilitation center for substance abuse is not considered a hospitalization unless that facility is associated with an acute care hospital. Outpatient substance abuse rehabilitation (day program) does not need to be reported.

14.3.1.c Transfers

Transfers from one hospital to another, on the same day, are considered one SAE event for SPRINT purposes. In cases of transfers between acute care hospitals, the beginning date is the admission to the first hospital and the discharge date is the date of discharge from the last acute care hospital. If records are required, staff should obtain records from all acute care hospitals.

14.3.2 Other Conditions of Interest

In addition, the following other important adverse events of interest to SPRINT should also be reported on the SAE form if they resulted in evaluation in an Emergency Room/Department (ER), regardless of whether they resulted in hospitalization:

- New or worsening heart failure
- Stroke

If the primary <u>reason</u> for the ER visit was a problem with any of the following, the ER visit should also be reported:

- Injurious falls

    A fall is defined as “a sudden, unintentional change in position in which the participant comes to rest on the ground, floor, or a lower level, NOT as the result of syncope or overwhelming external force.” For example, if someone is hit by a car or crashes a bicycle, this would not be considered a fall (but rather an accident). Falls that resulted in an injury that required medical attention (such as fracture, sprain, head trauma, etc) in an ER or urgent care facility should be reported.

- Syncope

    Syncope is defined as the temporary loss of consciousness, also known as fainting or passing out. Sensation that one is about to faint or might faint, but doesn't, is not syncope. There are many causes of syncope including cardiac and non-cardiac causes. Each episode of syncope that was evaluated in an ER should be reported on the SAE form.

- Arrhythmia

    Arrhythmia is defined as an abnormality in the heart rhythm and can be slow (bradyarrhythmia) or fast (tachyarrhythmia). Atrial fibrillation, atrial flutter, and supraventricular tachycardia are examples of arrhythmias. If a participant had an ER visit for a problem with his/her heart rate, it should be reported on the SAE form. An ER visit for <u>sensation of palpitations</u> without a documented abnormal rhythm in the ER would not be reportable.

- Electrolyte abnormality: hypo or hyperkalemia or hypo or hypernatremia.

    If a participant reports being seen in the ER related to a problem with his/her sodium or potassium, an SAE should be reported. Specific lab values should be included on the SAE form. (An SAE is not needed if the electrolyte abnormality was ***<u>only incidentally found</u>*** and was not part of the reason or symptoms that brought the participant to the

ER. For example, if a participant is in the ER because she has the flu and potassium of 3.2 is found on the labs, you do not need to report this as an SAE.)

- Hypotension
  Hypotension may be asymptomatic or may be accompanied by dizziness, lightheadedness, feeling faint, syncope, or other symptoms. If the participant reports being seen in the ER due to ***symptomatic*** low blood pressure, it should be reported on the SAE form.
- Unexpected events for which the investigator believes that the SPRINT intervention caused the event or contributed to the immediate cause of the event.

Other events that may be considered adverse events may need to be reported to your local IRB, even if they are not reportable to the SPRINT coordinating center. Follow your local reporting guidelines.

14.3.3 Reporting Serious Adverse Events
At each quarterly visit, SPRINT staff will specifically query participants for serious adverse events. In addition, information on serious adverse events may also be reported to study staff spontaneously by participants through telephone calls or emails between study visits. In addition to local reporting requirements, all serious adverse events should be data entered by clinic staff **within 72 hours** of knowledge of the event for review by the CC Medical Safety Officer. SAEs will be collected and reported from screening to the end of the study follow-up period for an individual participant. SAEs will be followed until resolution, stabilization, or until it is determined that study participation is not the cause. If a participant is seen in the ER and within seven days is admitted to the hospital for a procedure related to that ER visit, a separate SAE does not need to be completed. The original SAE for the ER visit should be edited to reflect the ER visit and the hospitalization.

For question by question directions on completing the SAE form, go to the SPRINT website main page, select Data Dictionary, select the QbyQ for the most recent version of the SAE form.

Figure 14.1. Flow for SAE ascertainment and reporting

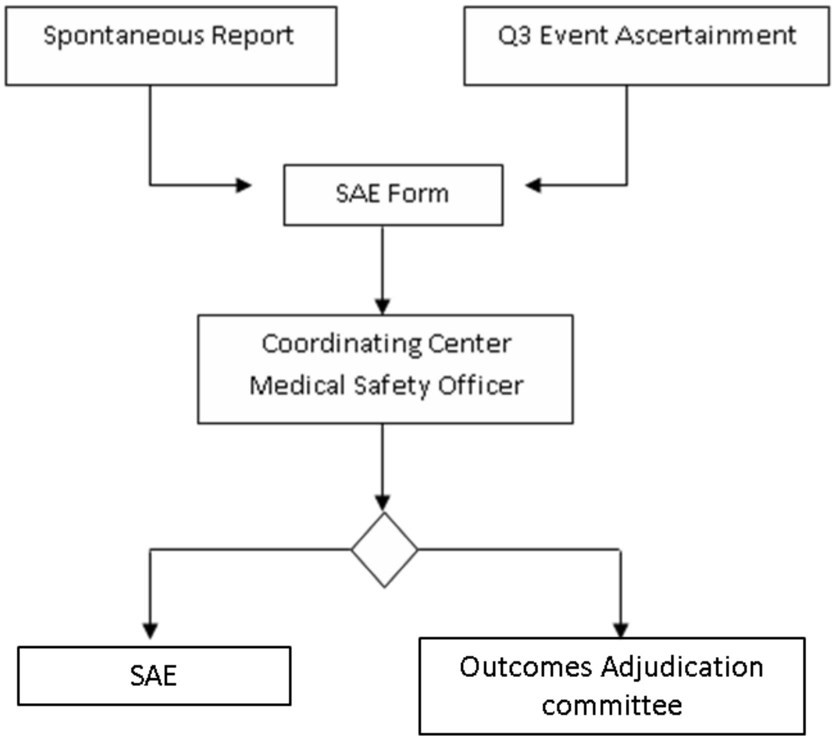

14.3.4 Clinical Site Tracking of SAEs

The SPRINT CC has a web-based SAE tracking system for CCNs and clinical sites to review and manage SAEs that have been reported. Step by step documentation about how to use this system can be found on the SPRINT website under Documents>Survival Kit>Chapter 5 – Safety and Outcomes Tools>Tutorial: Serious Adverse Event Tracking System. A training video for site tracking of SAEs can be found on the website: click on the SPRINT logo, select 'training', select 'training videos', select 'SAE Tracking System'.

14.3.5 Requesting documentation and sending records

For selected SAEs, the CC requires that medical records be sent to the CC for the safety officer's review. These include SAEs for the following conditions: bradycardia, electrolyte problems, acute kidney injury, syncope, hypotension and injurious falls. If the event is an emergency room visit, ER notes should be sent to the Coordinating Center. If the event is a hospitalization, the Admission History and Physical and the Discharge Summary are required. Please note, that "Discharge Instructions" are not the same thing as "Discharge Summary" and that we require the Discharge Summary. In order to obtain medical records from participants, each SPRINT Clinical Site should have the participant sign a "**Release of Medical Information**" form. It is recommended that this be done at every quarterly visit so that up to date Release forms are available should the participant have a medical event prior to the next quarterly visit. Release of information is discussed in detail in MOP Chapter 13, Section 13.3.1.

14.3.5.a De-identifying and sending records

Once you are sure you have the correct information on the participant and have obtained all documents, attach a SPRINT ID label to each document or write the participant ID on each page of the medical record. Mark out any identifying information where it appears on each page of a document that will be submitted. Use a grease pencil or redacting software if available. Be sure that the PHI cannot be seen. This step is very important, and you should look carefully for anything that is protected health information (PHI) such as name, social security number, telephone number, medical record number etc. It is preferred that records be uploaded on the website in the SPRINT Site SAE tracking system. Sites can also send records by fax (336) 713-5308 or by email to Deborah Felton (dfelton@wakehealth.edu).

14.3.6 Reporting the death of a study participant

The death of a SPRINT participant is only captured on the SAE form (the participant status form does not need to be completed for deaths). Death of a SPRINT participant may be discovered by the Clinical Site in several ways: during contact to schedule a study visit, a phone call to the Clinical Site staff by informants, during attempts to locate a SPRINT participant, or through other sources such as the obituary page in the newspaper. Information from such reports should be used to complete the Serious Adverse Events Form. Of particular importance are the date of death and the city and state of death; this information is critical in obtaining the death certificate. The Encounter and Disposition form should also be completed for the visit window in which the death occurred and the death should be indicated in Part D Question 9 as the reason the visit was missed.

14.4 Unanticipated problems

The vast majority of SAEs in SPRINT will not meet the definition of an "unanticipated problem" that needs to be reported to the NIH (even if the event itself was unexpected).

To determine if your participant has had an unanticipated problem that needs to be reported, answer the following 3 questions:

1) Was the event unexpected? The vast majority of SAEs will not be unexpected in SPRINT due to the nature of the condition and population we are studying. The OHRP defines unexpected AEs as those whose nature, severity, or frequency is not consistent with:

- the foreseeable risks associated with the study procedures (things listed in the protocol, consent, or product labeling for any of the SPRINT blood pressure medications) OR
- the natural progression of an underlying condition OR
- the risks of the population being studied

2) Was the adverse event caused by (partially or wholly) participation in SPRINT?

3) Does the adverse event suggest that the SPRINT study is placing participants at a greater risk of physical or psychological harm than was previously known or recognized?

If the answer to ALL 3 questions is YES, then the event is an unanticipated problem that needs to be reported as such.

If the answer to ANY of the above questions is NO, then it is NOT an unanticipated problem.

Unanticipated problems will usually result in protocol changes intended to keep participants safe. If you are unsure about whether an event at your site is an unanticipated problem, please call Deborah Felton at the Coordinating Center to discuss it. The flow chart found on page 14-8 is helpful in determining if an adverse event is an unanticipated problem.

A printable flow chart is also available on the website at Documents>Survival Kit> Chapter 5 – Safety and Outcomes Tools.

How to determine if an adverse event is an unanticipated problem

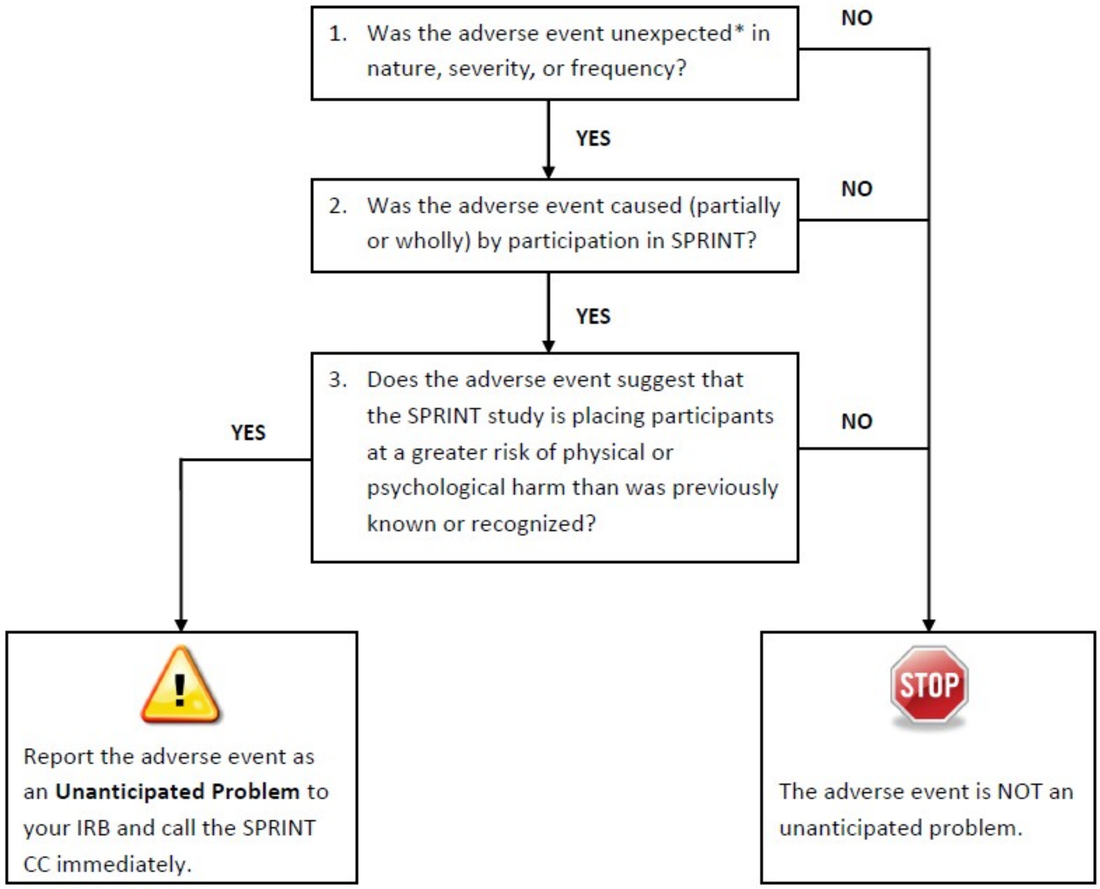

* The OHRP defines unexpected AEs as those whose nature, severity, or frequency is NOT consistent with:

- the foreseeable risks associated with the study procedures (things listed in the protocol, consent, or product labeling for any of the SPRINT blood pressure medications) OR
- the natural progression of an underlying condition OR
- the risks of the population being studied

14.5 Relationship of an event to the SPRINT study intervention
Adverse events may be caused by the SPRINT study intervention. Determination about the relatedness of adverse events to participation in research fall along a continuum between definitely related to the research to definitely unrelated to participation in the research. Events that are determined to be at least partially caused by the intervention would be considered related to participation in the study. Possibly related means there is a reasonable possibility that the event may have been caused by the study intervention. The safety committee reviews all events thought to be related to the study on regularly scheduled committee calls. When discussion of an event deemed definitively related to the intervention may be warranted, the appropriate CCN PI will be notified and asked to follow-up with the site PI per his/her discretion.

14.6 Modification of treatment in response to safety concerns
SPRINT is testing two different SBP treatment goals. The study physician may add, increase or reduce the dose, stop, or change antihypertensive drugs in the interest of participant safety. Depending on the situation, the change may be temporary or permanent. Situations that may require temporary reduction or elimination of a study medication include: side effects, electrolyte abnormalities, worsening congestive heart failure, acute kidney injury, symptomatic hypotensive episodes, and other illnesses. The MOP is not intended to replace clinical judgment and decision making in management of side effects and thus not all possibilities are covered here. Instead, we have provided guidelines for common situations.

14.6.1 Hypotension
14.6.1.a Orthostatic hypotension
Orthostatic hypotension (OH) is usually defined as a decline of systolic blood pressure (SBP) ≥20 mm Hg or a decline of diastolic BP ≥10 mm Hg that occurs within 3 minutes after moving from a supine or seated to a standing position. Orthostatic hypotension is usually related to specific drug classes and not BP level per se and thus should NOT usually alter target blood pressure goals. The following guidelines are adapted from the ACCORD blood pressure working group (2005).

Symptoms
OH may be asymptomatic or may be accompanied by dizziness, lightheadedness, feeling faint, or syncope. Generally, asymptomatic OH should not change adherence to the SPRINT protocol, although medication classes may be adjusted to continue appropriate sitting BP control while minimizing postural hypotension (see below).

Predisposing Conditions
In epidemiologic studies OH is more likely to occur in older individuals with high SBP. Diabetes mellitus and other autonomic neuropathies, volume depletion, varicose veins, alcoholism, and certain medications are also associated with OH.

Note: Occasionally, hypotension occurs if a patient previously non-adherent to prescribed medications begins taking the medications. Because BP typically declines after a meal due to splanchnic blood pooling, standing BP should not be measured within 90 minutes after a meal if possible.

Predisposing Medications
Some classes of medications are more likely to cause OH. The most frequent offenders currently in use are alpha-blocking drugs, such as prazosin, terazosin, or doxazosin, and central alpha agonists, such as clonidine, methyldopa or guanfacine. Although beta-blockers are perhaps the least likely antihypertensive class to cause OH, one of the most common adverse effects of combined alpha-beta blockers, such as labetolol or

carvedilol, is OH or dizziness, primarily because of the alpha-blocking component of these drugs. Rarely, beta-blockers may cause OH because of severe bradycardia (e.g., heart rate <40-50/min) and inability to increase heart rate/cardiac output on upright posture, especially if other drugs or conditions have lowered BP excessively.

Occasionally, reserpine, nitrates, or calcium channel blockers may contribute to or cause OH. Thiazide diuretics rarely cause OH, unless the patient is significantly volume depleted or another agent is added to a diuretic that may cause first-dose hypotension (e.g., a short-acting ACE inhibitor like captopril or a short-acting alpha blocker like prazosin). High dose loop diuretics, such as furosemide, or combinations of diuretics may lead to excessive volume depletion and hypotension, with or without OH.

Certain psychotropic drugs, most notably phenothiazines and tricyclic antidepressants can also cause OH.

Management
Patients with poor oral intake, dehydration from whatever cause, GI or renal causes for excessive fluid loss, or hemorrhage, may need to have their diuretic or other antihypertensive medications stopped temporarily until the volume-depletion is corrected.

If a participant with symptomatic OH has no obvious cause of excessive volume depletion, the medication regimen should be reviewed. Psychotropic drugs may need to be changed to alternative agents (many newer ones are equally effective without hypotensive effects) or reduced in dose. If the patient is on an alpha-blocker, alpha-beta blocker, or central alpha agonist, the dose should be reduced or the potentially offending agent discontinued and, if necessary for BP control, replaced with another class of antihypertensive drug less likely to cause symptomatic OH. If the participant requires an alpha-blocker for BPH/bladder outlet obstructive symptoms, a more selective alpha-blocker (e.g., tamsulosin) may be considered.

Patients with symptomatic OH should eat small meals and avoid standing up rapidly after eating. Such individuals should avoid hot showers and other excessive heat exposure. An increase in sodium intake may be considered in patients without hypertension or heart failure. In those with large varicose veins, fitted elastic hose or compression stockings may reduce venous pooling in the legs. In refractory cases of symptomatic OH, drug therapy with vasoconstrictors such as midodrine or dihydroergotamine or with mineralcorticoids may be considered, but this should be unlikely to be needed in SPRINT.

Conclusion: Decisions regarding up-titration toward protocol blood pressure targets should be made primarily on the basis of the seated blood pressure measurements. Orthostatic hypotension should not preclude up-titration toward blood pressure targets. When symptomatic, it is suggested that initial efforts described above directed toward identification of other causes (such as psychotropic drugs, recent meal ingestion) and preferential use of hypertension medication classes that have less predilection to orthostatic hypotension.

14.6.1.b Management of Participants with Documented Hypotension in the Clinic
The Study Coordinator must notify the site PI or M.D. Co-investigator or his/her designate (M.D., nurse practitioner or physician assistant) and ask for instructions prior to discharging a participant from the study clinic when either:

- The mean seated SBP at a given study visit is below 100 mm Hg AND the participant's mean seated SBP has decreased by at least 10 mm Hg from the last visit,

  OR

- The mean seated SBP is below 90 mm Hg

Notification of the PI, M.D. Co-investigator or his/her designate about the low SBP does not oblige any action. The M.D. or designate should exercise his/her best clinical judgment on whether action is warranted, taking foremost into consideration the safety of the participant.

14.6.2 Acute kidney injury/acute renal failure/acute tubular necrosis
Acute renal failure (ARF), or acute kidney injury (AKI), is defined as an abrupt or rapid decline in renal filtration function. Acute tubular necrosis (ATN) is the most common cause of AKI. It is usually marked by a rise in serum creatinine concentration or by a rise in blood urea nitrogen (BUN) concentration. However, immediately after a kidney injury, BUN or creatinine levels may be normal, and the only sign of a kidney injury may be decreased urine production. A rise in the creatinine level can result from medications that inhibit the kidney's tubular secretion. A rise in the BUN level can occur without renal injury, resulting instead from such sources as GI or mucosal bleeding, steroid use, or protein loading, so a careful inventory should be taken before determining if a kidney injury is present.

14.6.3 Hyperkalemia
Hyperkalemia can be life threatening and is often asymptomatic. SPRINT participants may develop hyperkalemia in the setting of RAAS blocker use or potassium sparing diuretics, particularly in the elderly and those with poor renal function. Participants with severe hyperkalemia (>6.5 mEq/L) should be referred to an emergency department for emergent treatment and monitoring (Nyirenda MJ, et al. Hyperkalemia. BMJ 2009; 339;b4114). Participants with potassium 6.0-6.5 mEql/L may also need referral to an ER depending on the acuity of the hyperkalemia and renal function. Such cases should be reviewed by the site PI or designated clinician. For more detailed information on causes and management of hyperkalemia, see Monitoring Participants with CKD (MOP Chapter 12).

14.6.4 Hypokalemia
Hypokalemia may occur with thiazide or loop diuretic use. Potassium supplementation may be needed. Current guidelines recommend the use of potassium chloride to keep serum potassium levels near 4.0 in patients with hypertension (Cohn JN, et al. New Guidelines for Potassium Replacement in Clinical Practice. Arch Intern Med. 2000;160:2429-2436). 20 mmol/day of oral potassium chloride is often sufficient to prevent hypokalemia; 40-100 mmol/day may be needed to treat hypokalemia. The dose of potassium needed depends on the degree of hypokalemia, renal function, and other participant characteristics.

14.6.5 Hyponatremia
Hyponatremia results from an excess of water in relation to sodium and may occur with use of thiazide diuretics, serotonin re-uptake inhibitors, dehydration, and many other medical conditions such as adrenal insufficiency, hypothyroidism, and SIADH. Symptoms depend on

the degree of hyponatremia, but include dizziness, confusion, and lethargy. Treatment is aimed at the underlying cause and thus exceeds the scope of this MOP chapter. However, if a participant has developed hyponatremia to <133 meq/L after initiation of a thiazide diuretic, consideration for decreasing the dose or discontinuing therapy may be needed. If Na <133, sites are encouraged to bring the participant back prior to the next visit for medication change or a repeat lab per the site clinician's judgment.

14.6.6 Hypernatremia

In outpatients, hypernatremia typically results from an excess loss of free water and may be found incidentally in SPRINT chemistry profiles, particularly in older adults who have a decreased sense of thirst and/or cognitive impairment. Treatment of hypernatremia requires correction of the water deficit. The water deficit can be calculated using the following formula:

Free water deficit = Wt in kg x %water* x ((serum Na/140)-1)

* % water for men=0.6, women=0.5, elderly men=0.5, elderly women=0.45

Participants with asymptomatic hypernatremia and Na 146-155 meq/L may be able to tolerate oral correction. Participants with symptomatic hypernatremia or Na >155 meq/L will likely need IV fluids and close monitoring.

14.6.7 Creatinine rise/ decline in eGFR

Initiation or up-titration of ACE inhibitors or ARBs may be associated with a rise in creatinine/ decline in eGFR. Decline in eGFR up to 30% may be tolerated. If a participant’s eGFR declines by more than 30%after initiation or up-titration of a RAS blocker, reasons for decline in renal function should be sought (for example dehydration, concomitant NSAID use, etc.) If no contributing factors are found, the ACEi/ARB dose may need to be reduced or discontinued. See CKD Chapter 12 for more details.

14.6.8 Bradycardia

Sinus bradycardia (heart rate <60 beats/min) is not unexpected in SPRINT. Participants in SPRINT are likely to be on one or more drugs that are known to lower heart rate such as beta-blockers, non-dihydropyridine calcium channel blockers, clonidine, digoxin, or antiarrhythmic drugs. SPRINT has discouraged the use of beta-blockers in combination with non-dihydropyridine calcium channel blockers to reduce the risk of significant bradycardia. Asymptomatic sinus bradycardia may not require changes in management. Participants with symptomatic bradycardia (light-headedness, pre-syncope, syncope, chest pain, shortness of breath, new fatigue, etc) should be evaluated with an EKG to evaluate for pathologic causes of or consequences of bradycardia. Symptomatic participants on a drug known to lower heart rate should have the drug(s) discontinued or the dose lowered as deemed appropriate by the site PI or designated clinician. Severe or persistent symptomatic bradycardia may need further evaluation and treatment in an ER or acute care hospital.

14.6.9 Allergy, Severe Dermatitis

If a participant displays signs or symptoms of allergy or severe rash, the suspected offending SPRINT drug should be discontinued. It may be appropriate to re-challenge some participants with the same drug or another in the same class.

14.6.10 Angioedema, neutropenia, agranulocytosis

Development of angioedema, neutropenia, or agranulocytosis requires permanent discontinuation of the suspect SPRINT drug. If the study site physician deems this as serious, further evaluation or treatment may be necessary.

14.7 Drug-Drug Interactions

Participants enrolled in SPRINT are likely to be taking multiple medications. In order to reach target blood pressures, it is anticipated that SPRINT participants will be on at least 2, and often 3 or 4, study medications. In addition, this cohort of patients is likely to be taking multiple medications for other chronic conditions besides HTN. Per good clinical practice, the site clinician should review all of the participant's medications, including non-study medications, to look for potential drug-drug interactions before prescribing or adjusting a study medication. The use of 2 RAS blockers (for example an ACE inhibitor and a RAS blocker) and the use of 2 nodal blockers (beta blocker and a non-dihydropyridine calcium channel blocker) are strongly discouraged. It is beyond the scope of this chapter to provide a comprehensive list of possible drug-drug interactions between study and non-study medications. Clinical investigators and staff should familiarize themselves with all agents to be utilized in SPRINT and consult with local clinical pharmacists or drug-drug interaction applications should questions arise with regard to use of any of the SPRINT agents.

14.8 SPRINT participants enrolled in other interventional trials

The SPRINT protocol and MOP indicate that participants should not be recruited if already participating in another interventional study. Once randomized into SPRINT, participants should be discouraged from joining another interventional study. In situations where a participant enrolls in another interventional trial, the site PI, CCN PI, CCN Medical Officer (if any), and the CCN's Intervention representative should review the situation for the safety of continuing active BP management in SPRINT. If there are safety concerns following this initial review, the case should be referred to the SPRINT Safety Officer who may elect to bring the case to the Steering Committee. See MOP Chapter 10, Section 10.5, page 10-17 for additional information.

14.9 Reports

The Coordinating Center will provide reports to the Safety Officer to assist in the review and monitoring of serious adverse events, clinical safety alerts and other reports necessary for monitoring the safety of study participants. The Coordinating Center will be responsible for timely reporting to the NIH and the DSMB. The Coordinating Center will provide reports of serious adverse events for review by the DSMB at their meetings.

14.10 Data Safety Monitoring Board

A **Data Safety Monitoring Board (DSMB)** is established, with responsibility to monitor all aspects of the study. The **Medical Safety Officer** reports to the DSMB for issues related to participants' safety. This independent Data and Safety Monitoring Board is established to monitor data and oversee participant safety. The SPRINT DSMB may include experts in cardiovascular medicine (particularly hypertension), kidney disease, clinical trials, geriatrics, biostatistics, quality of life, cost effectiveness, cognitive function and other areas as needed. DSMB participants include the Steering Committee Chair and Vice-Chair, CC PI and senior staff, and representatives from the NHLBI and other NIH sponsors. The DSMB normally meets twice a year to monitor safety, to advise the NHLBI about study progress and performance, and to make recommendations to the NHLBI regarding study continuation and protocol changes. In addition, the CC may provide data to the DSMB Chair to ensure early identification of any major adverse outcomes of therapy. The DSMB has the responsibility to recommend to the NHLBI whether the trial should continue, whether the protocol should be modified, or whether there

should be early termination. The DSMB will provide reports to the NHLBI through the Executive Secretary, who is appointed by the NHLBI. Recommendations by the DSMB must be approved by the NHLBI prior to implementation.

References

1. Crow RS, Prineas RJ, Hannan PJ and Blackburn H. Prognostic associations of Minnesota Code serial electrocardiographic change classification with coronary heart disease mortality in the Multiple Risk Factor Intervention Trial. *Am J Cardiol* 1997;80(2):138–144.

2. Zhang ZM, Prineas RJ, Soliman EZ, Baggett C, Heiss G; ARIC Research Group. Prognostic significance of serial Q/ST-T changes by the Minnesota Code and Novacode in the Atherosclerosis Risk in Communities (ARIC) study. *Eur J Prev Cardiol*. 2012 Dec;19(6):1430-1436.

3. R.J. Prineas, R.S. Crow, H. Blackburn. The Minnesota Code Manual of Electrocardiographic Findings. John Wright PSB, Boston (1982).

Chapter 15. Quality Control

15.1 Overview

Quality control and assurance are the responsibility of every member of the SPRINT team. This section of the MOP will described the quality control and assurance procedures to be used in SPRINT.

15.2 Division of Responsibilities

In order to maximize the quality of all of the data obtained in SPRINT, all members of the SPRINT team must strive for excellence. Everyone is responsible for mastering the material covered in the protocol and the MOP. All clinic personnel are responsible for achieving and maintaining a high level of understanding and performance of all clinic procedures. The Quality Assurance Matrix (Appendix) provides a detailed description of the study units that are responsible for various aspects of quality assurance. A brief description follows here.

15.2.1 Coordinating Center (CC)

The Coordinating Center staff are responsible for the following:

1. maintaining the integrity of the protocol, MOP, and regulatory document binder, and providing access to these materials on the SPRINT website;
2. organizing and, with the collaboration of the Steering Committee and its subcommittees, conducting central training sessions;
3. developing a data entry system that incorporates real-time data quality assurance features, such as range and logic checks and limited double data entry;
4. generating data query reports for dissemination to clinics and Clinical Center Networks (CCNs);
5. monitoring site initiation requirements;
6. monitoring the screening and randomization processes to ensure randomization of eligible participants and appropriate randomization allocation of participants;
7. generating timely web-based reports describing clinic, network and study performance, including but not limited to:
   a) recruitment,
   b) visit adherence and visit completeness,
   c) blood pressure treatment & control,
   d) data entry,
   e) data completeness,
   f) outcomes documentation;
8. producing case reports for 10 participants per clinic site for review by the CCN during bi-annual site visits;
9. receiving, maintaining and distributing to the MPQC Subcommittee copies of clinic site visit reports;
10. monitoring documentation of 100% of potential outcomes;
11. participating in MPQC Subcommittee activity;
12. monitoring the performance of the CCNs;
13. participating in clinic and other study component site visits as needed;
14. maintaining documentation of internal monitoring activities conducted by the CCNs, Laboratory, and Reading Centers;

15. providing rapid feedback to the CCN, Laboratory, Reading Centers and MPQC Subcommittee on the quality of data submitted and proposed corrections;
16. developing procedures, in collaboration with the MPQC Subcommittee, for the oversight of quality control studies conducted by the Laboratory and Reading Centers;
17. providing leadership to the Laboratory and Reading Centers for their development and distribution of specific measurement procedures and timely data gathering.

15.2.2 Clinical Center Networks (CCNs)

The CCN staff are responsible for the following:

1. maintaining current copies of the protocol, MOP, training binder and regulatory document binder through any revisions;
2. contributing to central training sessions;
3. assuring (and documenting) that the clinic sites have adequately trained personnel;
4. assuring that regulatory activity (e.g., IRB) is current;
5. reviewing a copy of the proposed consent form for required elements (and any revisions to the consent form);
6. collaborating with and assisting the Coordinating Center (CC) in implementation and standardization of the protocol within their network;
7. monitoring the performance of the clinic sites in all aspects of the study, including but not limited to:
   a) clinic burden and adequacy of staffing,
   b) responses to data edit queries,
   c) performance as indicated on monthly reports described above (CC responsibility #7),
   d) communicating with their clinic sites on at least a monthly basis regarding site performance from monthly reports,
   e) communicating areas of concern to the clinic sites,
   f) collaborating in the development of plans to solve problems faced by the clinic sites;
8. conducting sites visits to each clinic site on at least a bi-annual basis;
9. reviewing the participant charts for 10 participants per clinic site during bi-annual site visits;
10. producing and submitting to the CC, in a timely manner, a site visit report for each clinic site visit;
11. following up on issues of concern identified during site visits;
12. maintaining documentation of clinic monitoring activities;
13. participating in MPQC Subcommittee activities;
14. participating in other study component site visits as needed.

15.2.3 Clinic Sites

Staff at the clinic sites are responsible for:

1. maintaining current copies of the protocol, MOP, training binder and regulatory document binder through any revisions;
2. attending central training sessions, or being trained by CCN staff or their designee;
3. demonstrating proficiency in the conduct of SPRINT components prior to participating in study activities as described below;
4. maintaining adequate source documentation to support participant data forms, including eligibility, follow-up and events ascertainment;
5. performing double data entry on a select set of items for each participant (blood pressures & consent);
6. reviewing, and developing plans to respond to data edit queries and performance reports described above (CC responsibility #7);

7. developing plans to address areas of concern;
8. facilitating site visits on at least a bi-annual basis;
9. providing the participant charts for 10 participants during bi-annual site visits by the CCN;
10. reviewing and responding to the CCN site visit report for each site visit.

15.2.4 Measurement Procedures and Quality Control (MPQC) Subcommittee

The committee structure within SPRINT has been designed to assure quality in all aspects of protocol implementation. The Measurement Procedures and Quality Control (MPQC) Subcommittee has special responsibility for assuring quality of data collection as described below:

1. monitoring the performance of the clinic sites and CCNs in all aspects of the study, including:
   a) clinic burden,
   b) responses to data edit queries,
   c) performance as indicated on monthly reports,
   d) completeness and timeliness of data entry,
   e) outcomes documentation submission (in collaboration with the Morbidity & Mortality Subcommittee),
   f) data quality for forms submitted by the clinic sites;
2. communicating areas of concern to the CCNs;
3. collaborating in the development of plans to solve problems faced by the CCNs and clinic sites;
4. reviewing 10% of site visit reports on a regular basis to evaluate effectiveness of the CCN site visits;
5. monitoring data quality and performance of the Laboratory and Reading Centers;
6. providing reports regarding the quality of study implementation and conduct to the Steering Committee, Project Office, and DSMB.

15.3 Approaches to Maximize the Uniformity of Procedures and Measurements

The development of the MOP and study forms provide the foundation for maximizing the uniformity of procedures and measurements in SPRINT. Central training sessions will be held to train clinic investigators and staff in study procedures. At least one individual from each clinic site will receive intensive training in order to function as a trainer as needed for refresher training and in cases of staff turnover. The existing clinic staff will be the front-line trainers of new clinic staff with assistance from the appropriate CCN. Refresher training will be conducted as needed, as judged during the bi-annual clinic site visits. The training materials developed for the central training session will be made available to the CCNs and clinic sites for use in training new staff and in cases of refresher training.

The central training session will provide didactic and hands-on experience for the following components: blood pressure measurement, ECG, specimen collection, intervention and drug distribution, cognitive battery, study outcomes ascertainment, regulatory issues (informed consent and SAEs), and data management and data entry. Attendance at the initial central training session and subsequent training of new staff by the CCN Coordinator will require that the trainee read and understand the following materials: (a) the SPRINT protocol; (b) relevant sections of the SPRINT MOP; and (c) any additional materials provided at the central training session that are relevant to the trainee responsibilities (e.g., videos, slide presentations, etc).

The one component that will bi-annually require demonstrated proficiency in SPRINT is blood pressure measurement. This will be accomplished at the bi-annual site visit performed by the CCN Coordinator to the clinic site. Proficiency will be demonstrated by conducting the blood pressure measurement protocol on three volunteers, and completing the appropriate forms in the presence of a CCN representative, who will observe and evaluate the demonstration. Additional tasks may be requested to fulfill the criteria for proficiency. Documentation of proficiency will be maintained by the CCN.

Quality of data collection for other SPRINT components will be evaluated as the data are received at the appropriate laboratory, reading center, or coordinating center. For example, quality of specimen collection will be assessed by the Central Laboratory as specimens are received; quality of ECG tracings will be assessed by the ECG Reading Center; quality of outcomes ascertainment will be assessed by the Coordinating Center as outcomes packets are received and processed for adjudication.

Additional aspects of the study plan that will contribute to maximizing the quality of data include the use of standard equipment for measurement of blood pressure and ECGs; and central resource units for the measurement of blood and urine, ECGs, and MRI scans.

15.4 Data Entry and Management

An overview of data entry procedures is contained elsewhere in the MOP. The data entry system is an important component of the overall QC plan for SPRINT. The system allows for the printing of forms in advance of the study visit. The header on the form will contain a participant specific barcode which includes ID number and name.

Real-time data entry checks are performed as the data are entered, to include:

1. Eligibility checks of Inclusion/Exclusion criteria prior to randomization assignment;
2. Checks to make certain that forms cannot be entered for a subsequent visit until the status of a previous visit has been entered;
3. Range and logic checks on individual items;
4. An audit process that identifies values that have been changed after having been previously saved to the database;
5. Valid participant ID check.

In addition, data edit queries and data quality reports will be generated by the system. Elements that will require double data entry in SPRINT are consent form items and blood pressure measurements.

15.5 Site Visits to Clinic Sites

Site visits are an integral tool for clinic monitoring and the primary responsibility of the CCNs. Site visitors will typically be CCN coordinators with other CCN, CC and Project Office staff as needed. Site visits will be made bi-annually to each clinic site and will evaluate the following:

1. Assessment of recruitment strategy;

2. Confirm presence of regulatory documents, e.g., IRB approvals, annual renewal, protocol(s), and compliance with study reporting requirements;

3. Evaluate protocol adherence including verification of consents, inclusion/exclusion criteria, lab reports, ECGs and source documentation for 10 participant charts per visit.

4. Confirm safety monitoring is in place and appropriate action taken when indicated;

5. Assure that participants are being seen according to protocol guidelines;

6. Assess the site's performance in achieving study goals for key parameters (e.g. visit adherence, BP intervention, data entry/edits, etc.);

7. Verification of accurate and complete event ascertainment including completion of study forms and submission of supporting source documentation for outcome events;

8. Review drug accountability, confirm adequate drug inventory and aid in drug recall procedures if necessary;

9. Confirm study equipment and supplies (BP and ECG devices) are present and in good working order;

9. Trouble-shoot clinic and study problems (e.g. retraining of new personnel, searching for lost to follow-up participants, or editing on-site corrections, etc.) and provide technical assistance as needed;

10. Assess proficiency of blood pressure measurement with study staff;

11. Discuss site visit findings with site PI and staff (at conclusion of visit - ideally on site);

12. Provide follow-up report to the site and CCN PI and to the CC and Project Office if necessary or requested;

13. Confirm requested site visit issues have been addressed/completed at the clinic site.

15.6 Site Visits to CCNs and Central Units

The CC will conduct site visits to the CCNs and central units (Central Laboratory, ECG Reading Center, and MRI Reading Center) once in the first year of data collection. Other visits may be conducted as needed.

15.7 Site Visits to CC

The Project Office will conduct a site visit to the CC once in the first year of data collection. Other visits may be conducted as needed.

Appendix. Quality Assurance Responsibility of Study Components

Function	Project Office	Coordinating Center	Committee	Central Units	CCN	Clinic
**Recruitment and Randomizations**	Participate in committee functions	Generate reports, support committee	R & R: Review report and advise CCN & other study committees as appropriate. Other Committees to respond as appropriate.	NA	Review report, consider advice and interact with clinic	Review report, consider and act on advice
**Visit Adherence**	Participate in committee functions	Generate reports, support committee	R & R: Review report and advise CCN & other study committees as appropriate. Other Committees to respond as appropriate.	NA	Review report, consider advice and interact with clinic	Review report, consider and act on advice
**Data Entry**	Participate in committee functions	Generate reports, support committee	MPQC: Review report and advise CCN & other study committees as appropriate. Other Committees to respond as appropriate.	NA	Review report, consider advice and interact with clinic	Review report, consider and act on advice

Function	Project Office	Coordinating Center	Committee	Central Units	CCN	Clinic
**ECG Data Quality**	Participate in committee functions	Collaborate with ECG Reading Center in generating reports, support committee	MPQC: Review report and advise CCN & other study committees as appropriate. Other Committees to respond as appropriate.	ECG Reading Center: Collaborate with CC in generating report, participate on MPQC	Review report, consider advice and interact with clinic	Review report, consider and act on advice
**Lab Sample Collection and Shipping Quality**	Participate in committee functions	Collaborate with Lab in generating reports, support committee	MPQC: Review report and advise CCN & other study committees as appropriate. Other Committees to respond as appropriate.	Lab: Collaborate with CC in generating report, participate on MPQC	Review report, consider advice and interact with clinic	Review report, consider and act on advice
**Lab Data Quality**	Participate in committee functions	Generate reports, support committees	MPQC: Review laboratory data, advise Lab & other study committees as appropriate. Other Committees to respond as appropriate.	Lab: Collaborate with CC in generating report, participate on MPQC	NA	NA

Function	Project Office	Coordinating Center	Committee	Central Units	CCN	Clinic
**BP Control**	Participate in committee functions	Generate reports, support committee	INT: Review reports and advise CCN & other study committees as appropriate. Other Committees to respond as appropriate.	NA	Review report, consider advice and interact with clinic	Review report, consider and act on advice
**Outcomes & SAE Documentation**	Participate in committee functions	Generate reports, evaluate completeness of documentation, support committee	M&M: Review reports and advise CCN & other study committees as appropriate. Other Committees to respond as appropriate.	NA	Review report, consider advice and interact with clinic	Review report, consider and act on advice
**Outcomes Adjudication**	Participate in committee functions	Generate reports, support committee	Steering Committee: Review reports and advise M&M & other study committees as appropriate. Other Committees to respond as appropriate.	NA	NA	NA

Function	Project Office	Coordinating Center	Committee	Central Units	CCN	Clinic
**Monitoring Clinics**	Participate in committee functions	Generate reports, support CCN, Maintain copies of site visit reports; Advise other study committees as appropriate. Other Committees to respond as appropriate.	Operations & MPQC: Develop site visit content and procedures	ALL: Contribute to development of site visit content and procedures	Conduct site visits, generate reports to clinic and CC	Facilitate site visits, review reports and act on recommendations
**Monitoring CCNs**	Participate in committee functions & site visit teams	Generate reports, Conduct site visits, generate site visit reports to CCN and PO & other study committees as appropriate. Other Committees to respond as appropriate.	Steering Committee: Contribute to development of site visit content and procedures	ALL: Contribute to development of site visit content and procedures	Facilitate site visits, review reports and act on recommendations	NA

Function	Project Office	Coordinating Center	Committee	Central Units	CCN	Clinic
**Monitoring CC**	Participate in committee functions & site visit teams; Create site visit report; Advise other study committees as appropriate; Other Committees to respond as appropriate.	Facilitate site visit, review reports and act on recommendations	Steering Committee: Contribute to development of site visit content and procedures	ALL: Contribute to development of site visit content and procedures	Contribute to development of site visit content and procedures; Participate in site visits as needed.	Participate in site visits as needed
**Monitoring Central Units (ECG, Lab)**	Participate in committee functions & site visit teams	Generate reports, Conduct site visits, generate site visit reports to Central Unit and PO & other study committees as appropriate. Other Committees to respond as appropriate.	Steering Committee and all relevant subcommittees (i.e., MPQC, Operations): Contribute to development of site visit content and procedures; Participate in site visits	ALL: Facilitate site visits, review reports and act on recommendations	Contribute to development of site visit content and procedures; Participate in site visits as needed.	Participate in site visits as needed

Chapter 16. Study Forms and Q by Qs

The SPRINT study forms and question by question (Q by Qs) instructions can be located on the SPRINT website under Data Management >> Data Dictionary >> Data Entry Forms. All current and past version of forms are available.

Chapter 17. Data Management and Security

The SPRINT data management system utilizes a Web browser-based interface, with electronic data being stored and managed centrally at the Coordinating Center. Web based data entry systems work using familiar browser interfaces and can incorporate highly sophisticated programming, making collection of clinical and other information quick, efficient and flexible. The data entry screens similar in appearance to printed hard copies of study forms have been developed using hypertext mark-up language (HTML). Participant data entered by clinic staff is transmitted to a server at the Coordinating Center, where it resides throughout the study.

Data security in the web-based data system uses 128-bit encryption and Secure Socket Layer (SSL). Though unlikely and difficult, it is technically possible to intercept Internet communications and access the content of transmissions sent as plain text. To eliminate this threat, we employ a digital server certificate which allows communications between the web server and the client system to be encrypted. The banking industry and electronic commerce use this procedure; it is the best available.

Restricted areas of the web site are protected by user login. Prior to gaining access to the restricted area, the user is required to enter a username and password that will be checked against a database. If the combination is correct, a "flag" will be set to allow the user to enter certain areas of the web site. For security purposes, once a user has successfully logged into the system, inactivity for a period of 60 minutes will automatically force the user to re-authenticate prior to using the system again.

WFUSM is protected by a firewall that limits the source and type of traffic coming into the institution. This product remains under constant monitoring and control.

Each night, all data, programs, code, documents, etc. will be backed up to a DLT tape library. These tapes are kept indefinitely and are located in a fireproof cabinet that remains locked at all times. Periodically, copies of tapes are moved to a secure off-site location for storage. In the event that there is any loss of data, the information can be restored from tape in a matter of hours. The entire PHS computer facility is provided with conditioned power, UPS capability and environmental sensors with notification protocols.

SPRINT Data Management/ Data Entry

General Data Entry Guidelines
You will need access to the SPRINT web site (https://www.SPRINTTRIAL.ORG). This is obtained by contacting your CCN Coordinator to request access for you. The request should include name, contact information and study role. Once web access is complete, you will receive an automatic email from the SPRINT web site that includes a temporary password and instructions to set your own password. We then recommend you go to the web site and become familiar with all of the information. Additionally, to edit your contact information (name, address, email, fax and phone numbers, specialty if applicable) click on your name in the top right corner, and click Edit Your Profile Information.

SPRINT is utilizing an approach to data entry that we believe will make it easier for sites. This includes pre-printing of participant information on study forms, as well as listing all applicable forms needed for each study visit. PRN forms are also listed, and can be checked and printed as needed for each visit. Each form will print with the participant ID on the header and a unique bar code for each individual form. Data entry involves scanning the bar code that is printed on each form; scanning will take you directly to the form for data entry. Details for screening and randomization are below.

Data entry should be completed within 7 days of the visit for all study forms, except for a few exceptions. The My Health Form must be data entered within 2 days. The My Health Form needs to be reviewed immediately for the participant's response to question 9, page 5 (PHQ-9: Thoughts that you would be better off dead or hurting yourself in some way). Instructions for dealing with participants at risk of harming themselves are provided in the Safety MOP chapter. The Encounter & Disposition Form and any Serious Adverse Event Forms must be entered within 72 hours (3 days). The MIND Screening and Extended Battery must be entered within 5 days.

Screening Data Entry Instructions and Procedures
The CC requires documentation of IRB approval before you can screen participants. Once received, the CC will then set up the ability to print screening forms. Please contact your CCN Coordinator who will contact the Coordinating Center with IRB approvals.

The screening form is called the Inclusion/Exclusion Summary Form. In order to screen participants and print this form, click on the Data Management tab on the top of the menu list. Once here, click the Print Screening Form button. You will then have a drop down box and should see and select your site number (if not, contact your CCN Coordinator). You will have the option to print up to 20 Screening forms at one time. The participant ID numbers and bar codes will be pre-printed on the forms Review these forms and keep them for use in screening your participants. The participant IDs will be pre-filled for you on all study forms at each study visit. Each ID will stay with that participant throughout the trial.

Screen participants using the Inclusion/Exclusion Summary form (detailed instructions are contained in MOP Chapter 3a, Screening Visit Procedures). Part A of the form should be completed for all participants, regardless of eligibility status. The rest of the form should be completed until the end or until the participant is deemed ineligible.

Remember, you should data enter this form within 7 days for all participants screened, including those ineligible. Note that the gray shaded areas on the form are for clinic notes and are not data entered.

To data enter this form, go to the website, and click on the Data Management tab. From here, click on the Data Entry tab, and scan the bar code that was printed on the Inclusion/Exclusion Summary Form. This will take you directly to data entry of the form. You will now data enter the Inclusion/Exclusion Summary form here. Please have the signed informed consent form with you at the time of data entry. Be sure to pay special attention to Part F- Contact Information, as this will be important for you to have if the participant is eligible and you need to contact them for the randomization visit.

> The window between screening and the baseline visit is as follows:
> If the baseline (RZ) visit is within 7 days of screening visit, there is no need to re-screen for anything except a hospital or emergency department (ED) visit. If the screening visit is between 7 – 30 days from the baseline (RZ) visit, we need to assure that meds have not changed and if so, that BP and meds still qualify. If the screening visit is more than 30 days from the baseline (RZ) visit, then you should re-screen the participant.

If you re-screen the participant at any time, please keep the same ID and edit the Inclusion/Exclusion Summary form at each re-screening visit including the date of visit of the re-screen. You can do this by clicking on the ID of the participant that is listed for your site, or by scanning the bar code on the Inclusion/Exclusion Form. Once the participant is randomized, you cannot edit the Inclusion/Exclusion form. Use the Report a Problem (RAP) link from the top menu bar, to contact the CC if you need assistance. When submitting a RAP, please be sure to include the Patient ID, Barcode ID, Form and Visit, if applicable. If you mistakenly re-screen the same participant using 2 separate ID numbers, please send the CC the ID you want deleted and a brief reason for the deletion request.

Randomization Data Entry Instructions and Procedures
The CC requires that your site have MIND and ECG certification before you can randomize participants. Until that is completed and the CC has received this information, you will not be able to randomize and will not see a Randomization button. If your site does have these certifications and you do not see the randomization button, contact the Coordinating Center.
ONLY eligible participants will have the Randomization button displayed.

The randomization visit includes 2 sets of forms, RZ1 and RZ2:

> Randomization 1 (RZ1) forms set includes the Baseline Medication and Physical Exam form, the Baseline BP Management form, Self Administered Baseline History form, Encounter and Disposition form, ECG and Lab form, and the Participant Baseline Contact Information Form. These RZ1 forms are completed prior to randomizing the participant.
>
> Randomization 2 (RZ2) forms set includes the MIND Screening battery, My Health, Drug Dispensing Form, BP Medications Management Log, the Morisky Medications Adherence Scale and all of the subset forms that the participant was randomized into (can be Men's Health, Women's Health, MIND Extended Battery

and/or MIND MRI). Follow the procedures listed in the applicable MOP chapter for exact instructions on how to obtain measures and administer the forms.

The flow for randomizing participants involves completing the RZ1 (Randomization 1) forms packet first, then hitting the randomization button and obtaining randomization and sub-study assignments, and then printing and completing the RZ2 (Randomization 2) forms packet.

To proceed with randomization and to print these RZ 1 and RZ2 forms, you can click on the Data Management tab on the SPRINT menu bar, and then click on the Data Entry tab from the drop down menu. On the data entry page, select your site ID to see a listing of participants with inclusion/exclusion forms entered. Click on the participant ID to go to the **Participant Status Page**. Here you will see a visit schedule and visit window table listed on the right hand side of the page. Click on the "RZ1" link in the "CODE" column of the VISIT LIST **(see screen shot below)**. This will open the printing interface and will show you the participant's name, ID and visit code that will be pre-printed on the forms based on information entered on the Inclusion/Exclusion form. We recommend you have the Inclusion/Exclusion Summary form with you so you can again verify eligibility prior to randomization. The required forms for the RZ1 visit will be checked for you. **These should be printed from the participant's status page prior to randomization**. Make sure the correct language is selected (it will automatically default to English) and if PRN forms are needed at that visit, be sure those are checked. Click the "Generate Form(s)" button at the bottom of the page to print the forms packet. Follow the procedures listed in the applicable MOP chapter for exact instructions on how to conduct each visit and administer the forms.

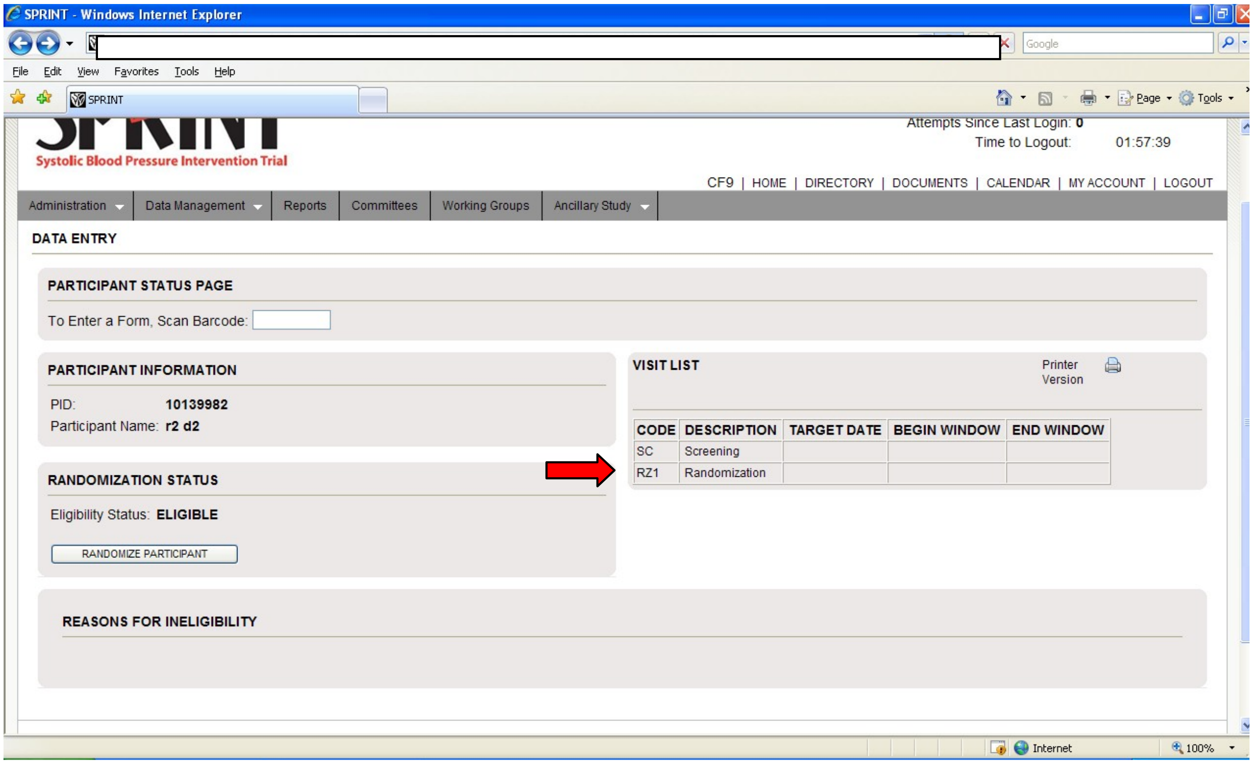

Next you hit the "Randomize Participant" button **(see screen shot below)**. Remember, you cannot print RZ2 forms packet without first randomizing the participant!

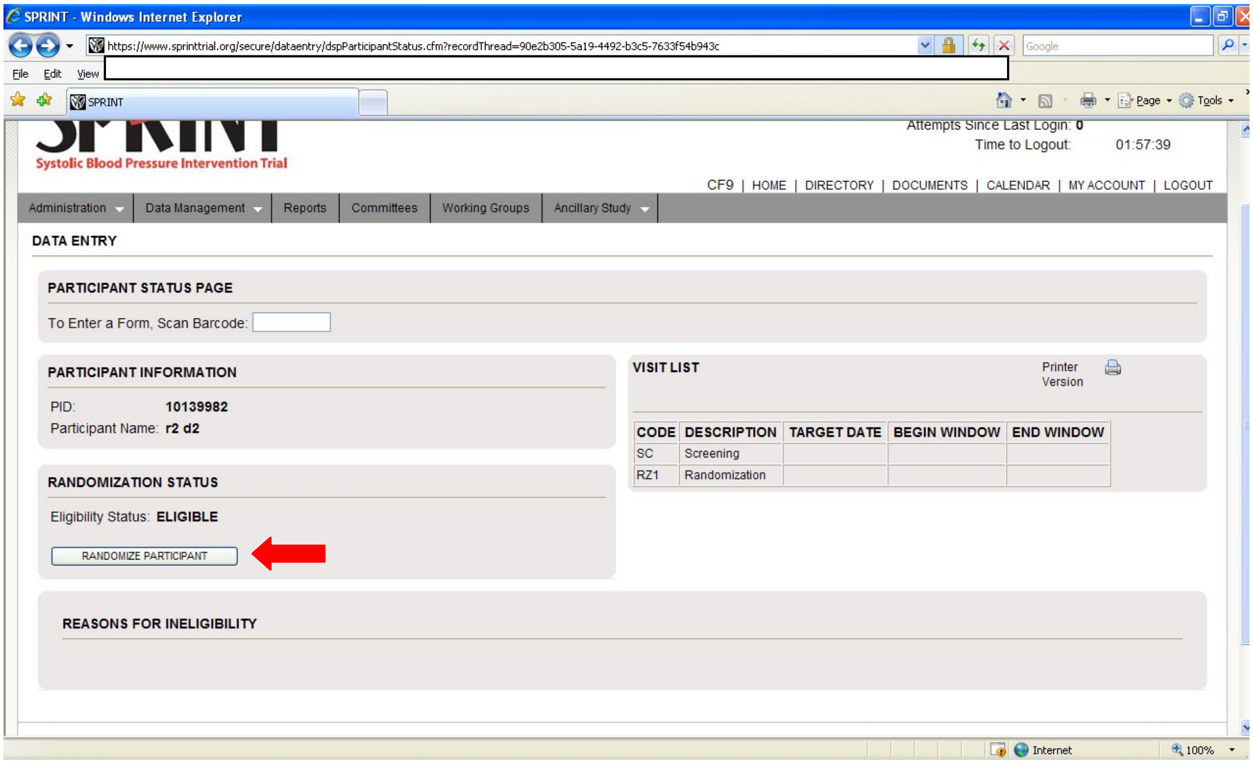

Please note that when you click the Randomize Participant button, you will be presented with a screen that asks a series of questions (responses are Yes or No). Data entry for the layered consent questions and the BP Management Form and Medical Release form **MUST BE ENTERED** or you will not be allowed to randomize the participant!

Participant ID:

Randomization Checks

Has a Blood Pressure Management form been completed for this participant?	1 - Yes
Has a Medical Release been signed by this participant?	1 - Yes
**Layered Consent**	
Agreement for reporting medical results to your physician	1 - Yes
Agreement for audio recording	1 - Yes
Agreement to participate in genetic studies	1 - Yes
Agreement for sharing DNA	1 - Yes
Agreement for sharing with GWAS Data Bank	1 - Yes
Agreement for long term follow-up	1 - Yes

submit

You will then be guided to data enter these consent layers a second time:

Randomization Checks

**Double Data Entry - Layered Consent**	
Agreement for reporting medical results to your physician	---Select---
Agreement for audio recording	1 - Yes
Agreement to participate in genetic studies	1 - Yes
Agreement for sharing DNA	1 - Yes
Agreement for sharing with GWAS Data Bank	1 - Yes
Agreement for long term follow-up	1 - Yes

submit

NOTE: Both entries for these layered consent questions must match or you cannot randomize!

After completing the RZ1 form set and randomizing the participant, you then click on RZ2.

For **data entry** of the RZ1 and RZ2 randomization forms, you scan the bar code label of each form and it will take you directly to that specific form data entry screen. The exception is the Central Lab Specimen Collection Form - this is not data entered by the site but is placed in the shipping package that is sent to the Central lab.

Emergency randomization procedures IF the SPRINT web site is down
In the event that the SPRINT web site is down and you have a participant in your clinic waiting to be randomized, please call a CC Project Manager (listed below) during the hours of 8:00 AM Eastern – 5:00 PM Eastern, Monday through Friday. If this emergency situation occurs during the hours of 5:00 PM – 8:00 PM Eastern Time, Monday through Friday, then call the emergency pager at **336-806-7467** and leave your phone number. A CC Project Manager will call you back and assist you. The Project Manager will assist you with the randomization if the web site is down at your site but not at the CC. If the web site is down even at the CC, the Project Manager will assist and assign the participant a treatment arm. In the event the web site is down at the CC, there will not be assignment into any substudies (Men's Health, Women's Health, MIND Extended Battery or MIND MRI). The Inclusion/Exclusion Summary Form should have been data entered already prior to the randomization visit. Once a participant is randomized, the Inclusion/Exclusion Form cannot be edited by the site; this will need done at the CC. If the site has NOT entered the Inclusion/Exclusion Summary Form, it will need to be faxed directly to the CC for data entry at the CC during the emergency randomization.

CC Project Managers
Debbie Felton (336-713-5274)
Brenda Craven (336-716-7067)
Loretta Sanders (336-713-5178)
Pam Nance (336-713-5402)
Tisha Perdue (336-716-1336)
Laurie Russell (336-713-4292)

Data Entry After Randomization
The visit windows for visits after randomization are calculated for you on the web site. Within each Participant's Status page, there is a visit table that shows you the "BEGIN WINDOW" and "END WINDOW" with dates corresponding to each visit. There is an option to print this page for placing in the participant chart as well.

Pre-printing of forms for each visit should occur no more than one week prior to the participant's visit. To pre-print forms, click on the Data Management tab on the SPRINT menu bar, and then click on the Data Entry tab from the drop down menu. On the data entry page, select your site ID to see a listing of participants. Click on the participant ID to go to the **Participant Status Page**. Here you will see a visit schedule and visit window table listed on the right hand side of the page. Click on the appropriate visit (or the PRN visit). This will open the printing interface and will show you the participant's

name, ID and visit code that will be pre-printed on the forms and the list of all applicable forms to be completed at the visit.

Each form will print with the participant ID on the header and a unique bar code for each individual form. Data entry involves scanning the bar code that is printed on each form; scanning will take you directly to the form for data entry.

Data Entry Error Messages
When data entering forms, there are checks built into the process that will prevent data entry errors and subsequent data queries. If you data enter a form, hit submit, and are given a red error message, none of the data will be saved until the errors are corrected (these will be highlighted in red). If you receive a yellow warning message, the data is saved but an error needs to be addressed. You can click on each error message and it will take you directly to the specific error so it can be corrected.

Chapter 18. Presentations and Publications

I. Introduction

A. The purpose of the policy is to encourage and facilitate the presentation of SPRINT background, rationale, design, and analyses; to ensure appropriate use of the SPRINT data, timely completion of manuscripts and presentations, equitable access to authorship, and adherence to established principles of authorship; and to coordinate the reporting of results.

B. This policy applies to all investigators analyzing, presenting, and publishing SPRINT data, except for those using the NHLBI Data Repository data (see https://biolincc.nhlbi.nih.gov/home/).

II. Principles

A. Research questions and hypotheses to be addressed using SPRINT data should be formulated *a priori* and clearly stated in a manuscript proposal to reduce the likelihood that study results are attributable to type I error.

B. Publication of scientific findings from the SPRINT Study should proceed in a timely fashion once relevant analyses are complete.

C. Publications arising from the SPRINT should avoid overlap and conflicting representation of SPRINT findings.

D. Recognition through authorship will be distributed among the *SPRINT Investigators* so that:
   - i) All *SPRINT Investigators and Team members* have equitable opportunity to lead and co-author SPRINT publications
   - ii) All *Ancillary Investigators* have the opportunity to lead and be co-authors on publications resulting from their ancillary studies.

E. The SPRINT should promote the career development of trainees and junior faculty by providing them the opportunity to lead and be co-authors of SPRINT publications.

F. Standards for authorship on SPRINT publications will adhere to the Uniform Requirements for Manuscripts Submitted to Biomedical Journals of the International Committee of Medical Journal Editors (e.g., NEJM 1997;336:309-315) and those established by the destination journals.

III. Structure of the Publications and Presentations Subcommittee

A. Implementation of this Publications Policy and management of day-to-day activities are the responsibility of the SPRINT Publications and Presentations Subcommittee, (abbreviated as the P&P SC for the remainder of this document). Nominations for Chair and co-Chair of the P&P SC are assembled by the CC from all voting members of the SC.

B. The Coordinating Center (CC) will provide staffing for the P&P SC.
   - i) This Publications Coordinator will:
     - (1) schedule conference calls and meetings of P&P SC,
     - (2) draft agendas for P&P SC meetings,
     - (3) review all paper proposals for conformity to the template,
     - (4) communicate needs for missing information to the proposer
     - (5) assemble manuscript proposals and drafts for review by P&P SC,
     - (6) record decisions made by the P&P SC and transmit them to the proposers,
     - (7) post approved paper proposals on a secure SPRINT web site (to which all investigators have access),
     - (8) facilitate communication between P&P SC and authors,
     - (9) assign a publication number and enter into tracking system, and
     - (10) other agreed to activities in support of the P&P SC's responsibilities.

IV. Procedures

A. Types of Manuscripts

There are three main categories of manuscripts and anticipated authorship:

i) Main results developed based on core SPRINT data and study aims/hypotheses (which will bear the corporate authorship, "The SPRINT Study Research Group"). The design and baseline papers will also be corporate authored. Main results papers and other corporate authored manuscripts will be described early in the study by the P&P SC

ii) Other SPRINT investigator authored manuscripts developed in response to an approved manuscript proposal after approval by the P&P SC

iii) Ancillary study results led by investigators bringing external funding into SPRINT for a specific project.

(1) Unless specific justifications and arrangements are made, all SPRINT analyses will be performed by the CC and ancillary study budgets should include funds allocated to the CC for that purpose.

(2) Ancillary studies that will conclude their funding period prior to the end of the SPRINT study, the P&P may allow the CC unblinded statisticians to run interim ancillary study analyses as long as the data (or analyses) are not released to the ancillary study investigators or any other blinded investigator on the study until the primary results paper has been released.

(3) Ancillary study manuscripts are subject to similar review and tracking procedures as other SPRINT Manuscripts.

B. Development of Manuscript Proposals

i) Proposals for manuscripts may originate from any investigator (including NIH project office team) or staff member.

ii) A proposal from a SPRINT Clinical Center should be reviewed by that Clinical Center Network's Principal Investigator before submission to the P&P SC.

iii) Proposals for SPRINT manuscripts must include "Summary Information" and a description of "Proposal Details" (see template, Appendix 1)

(1) <u>Summary Information</u>

(a) Full Proposal Title

(b) Abbreviated Title (Up to 50 letters and spaces)

(c) Proposed Writing Committee (including sponsoring SPRINT investigator if first author is not a *SPRINT Investigator and up to 2 conveners*)

(d) Abstract/Brief Description (including whether Events, Longitudinal, Cross-sectional, or Methods)

(e) Type of Manuscript (Main or Ancillary Study, including Title & PI for Ancillary)

(f) Data Analysis location (if other than SPRINT CC)

(g) Keywords

(h) Additional Comments

(2) <u>Proposal Details (limited to 3 pages)</u>

(a) Introduction (Brief rationale and background)

(b) Research Hypothesis (Clear statement of scientific questions to be addressed)

(c) Data (List of variables to be used)

(d) Analysis plan and methods in consultation with the SPRINT CC [description of proposed statistical analyses including available sample size, *a priori* selected confounders (covariates), and statistical power calculations]

(e) Relationship of the proposed manuscript to other SPRINT abstracts, manuscripts and approved/pending manuscript proposals, including potential overlap

(f) Proposed mock-up tables and figures

(g) Proposed journal(s) for submission
(h) References

C. Review of proposals
   i) The P&P SC will review all study paper proposals and reserve the right to nominate additional coauthors. (See Appendix 3 for review criteria.)
   ii) Manuscripts proposing to use data other than baseline data during the operational phase of SPRINT will be reviewed closely to ensure that SPRINT study objectives are not compromised. In general, the following will not be allowed:
      (1) Publication of follow-up data according to randomized group
      (2) Longitudinal analyses of outcomes pre-specified in the main protocol
      All proposals will be considered on a case-by-case basis.
   iii) Modifications to original paper proposals
      (1) If initial analyses suggest that a proposal should be split into two manuscripts, the rationale for this split should be submitted by the writing committee chair to the P&P SC for approval. In general, the writing committee members will remain the same for both papers.
      (2) If a writing committee decides that it will not produce a manuscript or it will combine its analyses with those of another writing committee for the purpose of generating one integrated manuscript, the writing committee chair(s) will submit the rationale for these changes to the P&P SC for approval. If the manuscript proposal abstract no longer applies to the manuscript, the proposal will need to be resubmitted to the P&P SC.
      (3) If a writing group decides to otherwise significantly modify its topic, the writing committee chair should submit a revised proposal to the P&P SC for approval.

V. Formation of Writing Committees

A. The P&P SC will provide oversight in the process of convening Writing Committees and nominating the Chairs of these Writing Committees.
B. General Principles for Selection of Writing Committee Chairs
   i) The Chair of the P&P SC will receive nominations for chairs of writing committees from P&P SC members and other SPRINT Investigators, including self-nominations.
      (1) .
   ii) Ordinarily, the individual submitting an authored SPRINT manuscript proposal and the principal investigator of a SPRINT ancillary study will serve as the Writing Committee Chairs for these two types of manuscripts, respectively.
   iii) Selection should also consider:
      (1) Equitable access to leadership of Writing Committees as delineated in this policy
      (2) Expertise of proposed Writing Committee chair based on prior publications and interest as demonstrated through ancillary study proposals and other activities.
      (3) Proposed Writing Committee chair's available time and commitment to moving manuscript development forward.
         (a) To assist in selection when more than one candidate to chair a Writing Committee is identified, a priority ranking can be used as follows: first – *SPRINT CCN PIs*, second – *SPRINT Clinical Center PIs, SPRINT Core Investigators*, and third – *SPRINT Team Members*. This priority ranking is only a rough guide and the P&P SC need not adhere to it explicitly if there are other considerations specified in this policy that dictate otherwise.
         (b) Except for ancillary study manuscript proposals, a specific request and rationale must be provided to P&P as justification for individuals to serve as Writing Group Chair on more than two active manuscript writing committees (defined by period between manuscript proposal approval by P&P and manuscript journal submission), especially addressing time availability for new manuscripts and effort on previously approved manuscripts.
C. General Principles for the Selection of Writing Committees:

After the manuscript proposal is approved by the P&P SC, the goals of writing group selection will include balanced involvement in writing activities by SPRINT investigators, including widespread representation of SPRINT Clinical Centers and NIH whenever appropriate and feasible, and inclusion of content-area experts throughout the SPRINT research network.

i) It is anticipated that for investigator initiated-authored manuscripts and ancillary manuscripts, the Writing Committee Chair will nominate up to two individuals to serve on the writing committee. Thus, a proposal can be submitted with up to three 'conveners'. In all cases, the composition of these writing committees must be approved by the P&P Subcommittee. Nomination to serve on a writing committee does not guarantee co-authorship. At least one member of the CC will have membership on each writing committee.

ii) Once a manuscript proposal has been approved by the P&P SC, the CC will send an email to all SPRINT core and ancillary investigators to solicit interest in being included in the writing group, from individuals who were not already nominated by the manuscript proposal convener. **The deadline will be 3 weeks for interested staff to complete the Statement of Interest for Participation in a SPRINT Writing Group Form and send this to their CCN PI, CC or NIH leadership**. At the end of 3 weeks, the CCN PI, CC or NIH must submit this information to the P&P SC within 1 week. This form is located on the web site under Documents > P&P. Below is information about completing the form.

iii) All interested investigators will be instructed to include the following in their statements:
    (1) Acknowledgment of availability to adhere to timelines outlined in the Publications Policy, and
    (2) Their level of interest in the manuscript (on a scale from 1 to 5, with 1 being the highest interest)
    (3) Any additional relevant information regarding particular experience in the content area (this will not be a prerequisite for inclusion on writing committees).
    (4) How they will fulfill sufficient criteria to warrant authorship as outlined in the Uniform Requirements (see below).
    (5) The deadline will be 3 weeks to provide this information back to each CCN PI, CC or NIH.
    (6) Individuals will normally not be considered for authorship on more than 20 active manuscripts at a time (manuscripts under development prior to journal submission)
        (a) CCN PIs will be advised by the P&P Coordinator when network investigators are approved for 15 or more active manuscript writing groups.
        (b) Specific request and rationale must be provided to P&P as justification for individuals to serve on more than 20 active manuscript writing committees, especially addressing time availability for new manuscripts and effort on previously approved manuscripts.

iv) For authored SPRINT manuscripts, potential authors **must identify their intention** to fulfill the criteria for authorship to be considered by the P&P SC.

v) For ancillary study manuscripts writing committee membership will also be open to all SPRINT PIs, SPRINT Core Investigators, and SPRINT Team Members based at Clinical Centers participating in data collection for the related ancillary study.

vi) Core SPRINT investigators at SPRINT's Clinical Center Networks, CC, and NIH will be asked to submit their statements of interest to the PI of their Clinical Center Network. The CCN PI will then be asked to review all such statements from within their network and send a prioritized list of investigators to potentially be included on the manuscript to the CC within 4 weeks after the date of the email solicitation for writing group membership.

vii) Ancillary investigators requesting membership on writing committees not related to their ancillary study and who are not core SPRINT investigators will be asked to submit their statements, including all requested elements above, directly to the CC for submission to the P&P SC.

viii) After four weeks, all nominations will be compiled and presented to the P&P SC for deliberation and decision-making. Inclusion in the writing group will depend principally on representation of Centers, equitable distribution of writing assignments across individuals, and the anticipated size of the committee that will be acceptable to the journals that are the likely target for submission. In addition, particular levels of expressed interest or experience with the content area will be considered.

ix) Procedures for the P&P SC to guide achievement of adequate representation of SPRINT core and ancillary investigators in writing groups:

(1) For SPRINT papers **not** directly addressing a specific aim from an ancillary study, representation, if possible, of all 7 SPRINT entities including the five Clinical Center Networks, the NIH, and the CC, ***at which individuals indicate an interest and commitment***. A maximum of two individuals can be nominated from each SPRINT entity. It is expected that the number of authors will usually not exceed 17: two from each of seven entities and three conveners. The CC assigned statistician is not included in the count of maximum of authors. **Exceptions to this policy must be clearly justified and approved by the P&P subcommittee and Steering Committee.**

(2) For SPRINT papers directly addressing a specific aim from an ancillary study, representation, if possible, of all clinical sites involved in data collection for an ancillary study, the NIH, and the CC, at which individuals indicate an interest and commitment.

(3) Inclusion of representatives from Reading Centers and the Laboratory, when appropriate.

(4) Conveners of manuscripts may propose inclusion of non-SPRINT researchers who bring particular expertise in a topic (basis for inclusion will need to be clearly justified).

(5) P&P SC will consider these potential authors, respecting the primary goals of the writing selection process.

x) The entire process described above will typically take place during the period of one month following the approval of any manuscript proposal by the P&P SC.

xi) SPRINT-wide authorship by each SPRINT entity (CC, Clinical Center Networks and Clinical Centers (with coordinator representation), Reading Centers, Laboratories, NIH) will be reviewed at least three times per year and presented to the Steering Committee as needed.

D. Writing Committee Responsibilities:

i) In the notification that they have been assigned to a writing committee, writing committee members will receive notification of the following agreement: *I agree to the following conditions regarding the use and disclosure of any SPRINT results I receive from the SPRINT Coordinating Center:*

(1) I will not use or further disclose SPRINT results for any purpose other than accomplishing work on the approved SPRINT publication and/or presentation.

(2) I will abide by all requirements of the SPRINT Publications Policies with regard to the use and disclosure of any SPRINT results.

ii) The Writing Committee Chair in collaboration with the CC is responsible for all phases of manuscript preparation including:

(a) Preparation of the manuscript proposal, the identification of data analyses needed, and submission of interim status reports to the P&P SC

(b) Assignment of tasks to writing committee members with clear deadlines for completion of these tasks and determination that the tasks are completed on schedule;

(c) Preparation and circulation of drafts for approval by each member of the writing committee before submission of a penultimate draft to the P&P SC;
(d) Determination of the order of authorship on the manuscript.
(e) Selection of a journal to which the manuscript will be submitted
(f) Journal submission
(g) Correspondence with co-authors, communication with the CC and Steering Committee, responses to the P&P SC and NIH reviews, and to journal editors.

iii) Writing Committee Members

(a) In addition to being reminded of Uniform Requirements, all members of writing committees will be required to participate in writing committee activities and be able to confirm that they:
(i) gave final approval of the submitted manuscript.
(ii) participated sufficiently in the work to take public responsibility for its content.
(iii) made substantial contributions to the intellectual content of the paper through:
(iv) conception and design of the work, OR acquisition of data, OR analysis and interpretation of data,
AND
(v) drafting OR critical revision of the manuscript for important intellectual content,
AND
(vi) statistical analysis, OR obtaining funding, OR administrative or technical support, OR supervision.
(b) Members are responsible for performance of tasks assigned by the Chair within the allotted time.
(c) Each member must participate actively in the preparation of the manuscript.
(i) If a writing committee member does not accomplish the tasks assigned to him/her and has not contributed to the manuscript, he/she may be removed from the writing committee.
(ii) Prior to a request for removal of any writing committee member, the Writing Committee Chair must contact the member in writing with a request for participation or performance of a task, and indicate that non-response within two weeks will be considered notice that the writing committee member no longer wishes to participate in the writing activity.
(iii) The Chair must then send a letter to the P&P SC requesting the removal from the writing committee of non-contributing members. Recommendations to remove a writing committee member must be approved by the P&P SC. Decisions may be appealed to the Steering Committee.

VI. Schedule for Manuscript Preparation

A. Monitoring paper progress

i) The P&P SC will discuss all papers on a regular conference call and develop consensus decisions on each proposal.
ii) All approved manuscript proposals will be posted on the secure portion of the SPRINT web site, to which all SPRINT investigators have access.
iii) The P&P SC, in consultation with the CC, will determine priorities for data analyses for manuscripts and abstracts that will occur at the CC. Publications arising from training grant ancillaries (e.g., K01, K23, etc.) may have one of the highest priorities for analysis because of their limited resources and the particular needs of trainees to be academically productive during their training.

B. Production and revision of drafts
   i) It is ordinarily expected that writing committees will complete a first draft (minimum, of Introduction, Methods, and Results sections) within two months after receipt of a set of complete analyses from the CC.
   ii) The Writing Committee Chair should send this draft to the members of the writing committee. It is recommended that a response deadline of 2 (two) weeks be given to writing committee members to prevent unnecessary delays.
   iii) The Writing Committee Chair should respond to substantive and major editorial comments from Writing Committee members.

C. Penultimate Draft
   i) The penultimate draft is expected four (4) to six (6) months after the first draft is distributed to the writing committee.
   ii) A penultimate draft should be only be submitted when considered ready for submission to a journal. After review and approval of the penultimate draft by writing committee members, the writing committee should send the penultimate draft to the P&P SC.
   iii) The P&P SC Coordinator will assure that appropriate acknowledgements, including contract and grant numbers, are included.

D. Review
   i) The manuscript will be reviewed by two SPRINT investigators who are not members of the writing group. At least one of these will be a member of the P&P SC. It is assumed that the expertise of ancillary study investigators may also be utilized in the review process.
   ii) The reviewers will complete the review within two weeks and provide comments to the P&P SC to be forwarded to the Writing Committee Chair.
   iii) It is expected that the Writing Committee will respond to the reviewers' comments within three weeks of receipt. It is understood that appropriate revision of the manuscript may take longer if further analyses are required.
   iv) The P&P SC will review the response(s) from the Writing Committee to assure that all comments were addressed, then forward to the SPRINT Steering Committee and NHLBI for review prior to submission to the journal. If Steering Committee members have no comments one week after manuscript circulation, this will be accepted as an endorsement for submission. Per the SPRINT contract deliverables, the NHLBI will have 3 weeks to review all manuscripts PRIOR to journal submission. This may be done simultaneously with the SPRINT Steering Committee review if possible.

E. Verification
   i) The CC will initiate verification of manuscript results if analyses were not initially conducted by the CC prior to approval by the P&P SC.
      (1) This verification will also confirm the insertion of the appropriate acknowledgements of SPRINT components and sponsoring agencies

F. Submission to a Journal
   i) Within thirty days of receiving NHLBI, P&P SC and SPRINT Steering Committee comments and verification confirmation, the Writing Committee chair will circulate the revised manuscript to the writing committee for final sign-off.
   ii) Final manuscript for initial and all resubmissions, if necessary, must be sent for final review by P&P SC , NHLBI and Steering Committee with a 1 week turnaround (or approval assumed); NHLBI requires a 3 week turnaround.
   iii) The P&P SC, the CC, and all co-authors must receive a copy of the journal cover letter and final draft of the manuscript.

G. Deviations from Schedule
   i) Deviation from this schedule requires approval from the P&P SC.
   ii) Failure to adhere to this schedule will prompt a review of circumstances.

iii) If it is determined that a manuscript is delinquent, this could be the basis for replacing the member(s) of the writing committee responsible for the delay, or for disbanding or reconstituting the writing committee.

H. Tracking Manuscripts

i) The Writing Committee Chair must keep the P&P SC and the co-authors informed as to the manuscript's progress through journal review.

ii) Upon publication of the manuscript, the Writing Committee Chair will provide donna.woerner@va.govan

electronic copy of the final publication to the CC for posting on the SPRINT web site.

iii) The Writing Committee Chair will be responsible for depositing the final manuscript in PubMed Central within six months after publication of the manuscript.

I. If there are substantive changes (adding new data or re-analyzing the existing data set used for the initial submission) made in the manuscript during journal review (major findings or conclusions, alterations of the sample, exclusion/inclusion of major covariates), the revised manuscript should be submitted to the P&P SC for re-review.

VII. Appeals

If one or more writing group members disagree with the data analyses, interpretation of the data, or authorship, the members should discuss the disagreement with the lead author, who makes a decision on how to resolve the dispute. If either the members disagree with the decision, or the lead author does not respond to the request for changes, the writing group member(s) should ask for a polling or formal vote of the entire writing group relating to the issue(s) in dispute. If this does not resolve the issue(s), and the writing group member(s) believe(s) that it is in the best interests of SPRINT to not allow the paper to proceed, an appeal may be made to the P&P Committee Chair, who will attempt to resolve the issues or appoint an appropriate P&P member to resolve the issue(s) in a meeting or conference call with the lead author and the member(s) who are in disagreement. If this is unsuccessful, and if the P&P Committee Chair, with the approval of the committee, cannot make a decision, then the P&P Committee Chair should solicit expert opinion from within SPRINT and if necessary from outside the study. If final arbitration is necessary, the P&P Subcommittee (through the chair) will refer sequence of events and final decision to the SPRINT Steering Committee.

VIII. Abstracts and Presentations

A. Preparation and Submission of Abstracts for Scientific Meetings

i) No abstracts may be submitted to any national or international organization for consideration without prior review and approval by all co-authors, SPRINT P&P SC, SPRINT Steering Committee and the NHLBI.

ii) Proposals for SPRINT abstracts requiring data analyses must be approved before the CC can honor specific data requests.

iii) The CC requires at least four weeks preparing data for use in proposed abstracts or presentations.

iv) An investigator who submits an abstract without these approvals may be asked to withdraw the abstract or presentation in question.

v) It is the intention of this policy to promote the conversion of as many abstracts as possible into full manuscripts. Abstracts should be submitted for review and approval on the SPRINT web site (P&P Tab, then under “Navigate” select Enter New Abstract) .

vi) If a Writing Committee has been assembled previously, it is encouraged that all writing committee members be identified as an author of that abstract, whenever possible.

(1) It is recognized that time and space constraints may preclude the inclusion of all approved writing committee members as authors on an abstract.

(2) Writing committee chairs are encouraged to include as many co-authors as can be accommodated and whose approval for the submitted work can be obtained prior to established deadlines for the abstract.
(3) Any co-author who has not approved of the content of the abstract should not be included in the final list of authors for the abstract.
(4) In the special circumstance where a writing committee chair has received permission from contributing authors, an abstract may be submitted listing only the writing committee chair and the corporate SPRINT authorship.

vii) The full text of abstracts is due to the P&P SC for review no less than two weeks before the abstract submission deadline. Abstracts submitted too late for review may not be approved for submission if the P&P SC is not able to approve in time.

B. Principles and Guidelines for SPRINT Presentations
i) The following guidelines apply to all presentations including poster presentations, oral communications at national meetings, grand rounds, invited presentations, and talks to community physicians, etc. that include SPRINT data:
(1) Presenters are encouraged to freely present <u>published</u> material from SPRINT.
(2) Presenters are encouraged to freely present SPRINT data that has appeared in publications or in abstract form at national meetings with appropriate acknowledgements.
(3) Distribution of written handout material containing SPRINT data that have <u>not</u> been published is prohibited.
(4) Presenters who have questions about unpublished material that they would like to present must seek approval from the SPRINT <u>Steering</u> Committee (rather than the P&P SC).
(5) Presentation of ancillary data from SPRINT must adhere to these guidelines.
(6) Slide presentations given for regional, national and international audiences will be made available on the SPRINT internal website.
(7) Presentation of published SPRINT material must include the SPRINT Research Group Disclosure Statement.

C. Accepted Abstracts and Invited Presentations
i) Copies of accepted abstracts or invited presentations (including tables and graphs) must be submitted to the P&P SC.

IX. Acknowledgements

All SPRINT publications must acknowledge the funding sources, contract numbers and a list of investigators-

APPENDICES

1. Manuscript Proposal Template
2. Manuscript Proposal Review Criteria and Template
3. Manuscript Review Prior to Submission Template
4. Timeline for SPRINT Manuscripts and Abstracts
5. SPRINT Research Group Disclosure Statement
6. SPRINT Policy on Slide-Set Availability

APPENDIX 1
MANUSCRIPT PROPOSAL TEMPLATE

1. Summary Information
    a. Full Proposal Title
    b. Abbreviated Title (Up to 50 letters and spaces)
    c. Proposed Writing Committee, no more than three individuals total (Including sponsor if first author is not a *SPRINT Investigator*)
        1. Confirm that all writing committee members have read and approved the proposal
    d. Abstract/Brief Description (Events, Longitudinal, Cross-sectional, Methods)
    e. Type of Manuscript (Main, Ancillary Study, Title & PI for Ancillary)
    f. Data Analysis locations (if other than SPRINT CC) and timing (interim data needed?)
    g. Keywords (if applicable)
    h. Additional Comments
2. Proposal Details (limited to 3 pages)
    a. Introduction (Brief rationale and background)
    b. Research Hypothesis (Clear statement of scientific questions to be addressed)
    c. Data (List of variables to be used, biological samples including volume of samples if relevant)
    d. Analysis plan and methods in consultation with the SPRINT CC (Detailed description of proposed statistical analyses including available sample size and power calculations after consultation with CC)
    e. Relationship of the proposed manuscript to other SPRINT abstracts, manuscripts and approved/pending manuscript proposals, including potential overlap
    f. Proposed mock-up tables and figures
    g. Proposed journal(s) for submission
    h. Key references

APPENDIX 2

SPRINT Manuscript Proposal Review Template

Reviewer:

Date:

Full Proposal Title & Number:

Research Hypotheses and Scientific Scope

1. Are the research questions stated in the proposal? YES NO

2. Are the research questions addressed in this proposal of scientific importance?

YES NO

3. Is the scope of the proposal appropriate for a single manuscript?

YES NO

4. If this proposal is based on an ancillary study, do the aims of the proposal map to the ancillary study aims?

YES NO

5. Does the proposal overlap with any previously approved proposals?

YES NO

IF YES: Is strategy to manage appropriate and approved by the convener of approved proposal?

YES NO

6. Does this proposal overlap with other submitted proposals under review?

YES NO

IF YES:

a. **Is a strategy to manage overlap readily apparent?**
b. **Is there potential for proposals to be combined?**
c. **Does one or more of proposals have sufficiently higher merit compared to other proposals to be considered for approval on its own despite overlap?**

Please explain any negative responses, including those related to potential overlap. Please indicate the question number in your response.

Data and Analysis Plan

1. **Is a complete list of variables to be used provided? YES NO**

2. **Are the variables requested appropriate for the scope of the proposal?**

   YES NO

3. **Do the mock-up tables and figures appropriately represent the intended output?**

   YES NO

4. **Is a clearly explained and adequate outline description of the proposed statistical analysis provided?**

   YES NO

Please explain any negative responses and indicate the question number in your response.

Will public data release by NHLBI jeopardize potential publication?

YES NO

Summary Opinion:

The reviewer should provide a short paragraph that summarizes the strengths and weaknesses of the manuscript and any additional comments not addressed in the questions above.

My final recommendation is to:

Approve:

Approve pending revisions, specify in summary:

Reconsider after major revisions including strategy to manage overlap with overlapping proposal(s), specify in summary:

Priority Level:

1 – high

2 – medium

3 – low

Do you perceive this proposal to be put forward by someone in training status?

YES NO

Does this proposal utilize the Morisky Medication Adherence Scale (MMAS-8-Item)?

YES NO

ADDITIONAL COMMENTS

APPENDIX 3
SPRINT Manuscript Review Criteria

The SPRINT P&P Sub Committee aims to assure that each manuscript is an appropriate and valuable use and interpretation of SPRINT data and represents the study well.

Criteria for Approval:

☐ **The manuscript is consistent with the approved paper proposal.**

- Paper does not “drift” into areas not proposed, particularly such that publication of other papers on the topic would be compromised. In addition, it does not include already-published main results (beyond “Table 1” descriptive information).
- All coauthors, including those nominated by the Steering Committee, are included and have approved the submitted draft.

☐ **Main study description is appropriate.**

- Accurately describes study in appropriate detail
- Contains no significant errors

☐ **Analysis is appropriate.**

- Statistical analysis reasonable, although the reviewer may have used different methods
- Alternate appropriate methods would not have resulted in significantly different conclusions.

☐ **Interpretation of results is appropriate.**

- Conclusions do not extend inappropriately beyond the data.

☐ **The manuscript is reasonably well-written.**

- The Abstract is clear and accurate.
- There are no significant grammatical, syntactical, or spelling errors that should have been corrected during earlier draft stages.
- Note: Coauthors whose primary language is English have a special obligation to assist authors whose primary language is not English and assure that language corrections are made before submission to the P&P Committee.

☐ **Study funding is properly acknowledged, per the SPRINT Acknowledgement List posted at SPRINT web site.**

☐ **APPROVE** ☐ **APPROVE WITH REVISIONS** ☐ **DISAPPROVE**

The SPRINT P&P Committee expects that all of these criteria are addressed prior to journal submission. If the Committee determines that changes are necessary, it will Disapprove (a rare situation) if the changes needed are substantial or will Approve with Revisions and request that the revised manuscript be reviewed and approved by one or more committee members prior to journal submission.

APPENDIX 4A
Timeline for SPRINT Manuscripts: Proposal to Publication

SPRINT Investigator develops proposal and submits via the SPRINT website.	Time 0	• Proposal Received
P&P reviews proposal, provides feedback and a decision to the chair of the manuscript proposal.	≤ 6 weeks	• Proposal Approved<br>• Proposal Approved<br>• w/Comments<br>• Proposal Pending Approvals<br>• Proposal Conditional Approval<br>• Proposal Not Approved
Once approved, the P&P via the CC solicits writing group nominations by email and study newsletter, to all SPRINT Core & Ancillary Study investigators and staff.		Time from approval of proposal to final writing group should take ~ 2 months
Investigators and staff interested in participation submit their statement of interest to their CCN/CC/PO leadership within 3 weeks after announcement of the open writing group solicitation.	3 weeks	
CCN/CC/PO leadership sends prioritized lists of authors to the P&P via the CC coordinator.	1 week	
The CC compiles the list of nominations for P&P review.	2 weeks	
The P&P selects and approves the writing group members.	2weeks	
The chair and writing group members work with the CC biostatisticians to develop an analysis plan and receive results. Time from receipt of final analyses to approved paper is ideally 9-11 months.		
The writing group completes first draft after receipt of complete analyses	2 months	1st Draft
The writing group completes penultimate draft and sends the completed draft to the P&P and Project Office via the CC coordinator.	4-6 months	Penultimate Draft Received
The CC reviews the penultimate draft for completeness and sends to 2 P&P reviewers.	1 week	P&P Primary Reviewers
Reviewers complete review and send comments to the CC.	2 weeks	Primary Reviews Returned to CC
The CC sends comments to the P&P and the writing group chair. The writing group chair responds to reviewer's comments and sends revised paper to the P&P via the CC coordinator.	3 weeks	Revised Manuscript Received
The P&P reviews responses from the writing group chair and forwards the paper to the Steering Committee (SC) for approval.	2 weeks	P&P Review/SC Approval
The P&P submits the manuscript to the NHLBI for review; this can be simultaneous with the SC review	3 weeks	NHLBI Review
The writing group chair sends revised paper to the writing group.	30 days	Distribution to Writing Group
The writing group chair submits the final paper to the targeted journal and is responsible for depositing it into PubMed Central.		Journal Submission
(Note: all revisions/resubmissions must be sent back to P&P, NHLBI and SC for review – to be accomplished within one week).	1 week	Revisions/Resubmissions

*CC refers to P&P Coordinator (Pam Nance)

.

APPENDIX 5

SPRINT RESEARCH GROUP APPROVED DISCLOSURE

"The views expressed in this .... are those of the authors and do not necessarily represent the official position of the National Institutes of Health (NIH), the Department of Veterans Affairs, the U.S. Government, or the SPRINT Research Group."

APPENDIX 6

SPRINT POLICY ON SLIDE SET AVAILABILITY

Purpose: This policy is designed to define how and when unpublished data or slides with unpublished data which were presented by a SPRINT investigator at a national or international meeting are available to other SPRINT investigators and to the public.

Policies:

1) Slides which have been presented at national meetings and for which the underlying paper has been published will be made available as quickly as possible on the public side of the SPRINT website, specifically, at https://www.sprinttrial.org/public/dspScience.cfm (click on "SPRINT Science" at the top of the website prior to logging in). These are available to the public.

2) Slides which have been presented at national meetings and for which the underlying paper has not been published will not be disseminated by default. If a SPRINT investigator would like to use these slides, she/he must make a request to the lead author of the paper (not the presenter of the slides) and the ancillary study PI (if the data were collected from an ancillary study and the ancillary study PI is not the lead author of the paper). If permission is not granted, the investigator may appeal to P&P which would make a recommendation to SC. For national or international presentations using the slides described under (2), the presenter should also submit presentation to P&P review

For all presentations, the following disclaimer needs to be included in the presentation:

"The views expressed in this .... are those of the authors and do not necessarily represent the official position of the National Institutes of Health (NIH), the Department of Veterans Affairs, the U.S. Government, or the SPRINT Research Group."

Chapter 19. Ancillary Science and Other Policies

1. Ancillary Studies Policy
2. Conflict of Interest Policy
3. Add/Drop Clinical Sites Policy

1. Ancillary Studies Policy

Introduction

In addition to the main SPRINT protocol, investigators may wish to perform Ancillary Studies using the SPRINT population, blood or urine samples, or other collected data. An ancillary study is an investigation not initiated by the SPRINT Steering Committee, with objectives that are not within the main SPRINT specific objectives and not part of the SPRINT protocol but uses SPRINT participants, samples, and/or data collected by SPRINT. In most cases, an ancillary study will involve acquisition of additional data that are not compiled as part of the SPRINT data set. SPRINT will reserve 50% of all biospecimens until SPRINT has ended. The highest priority for SPRINT biospecimens is to allow investigators to explain the main findings of SPRINT. SPRINT should not be used as a convenience sample if there are other samples available but consideration will be made if the science is highly meritorious and/or is aligned with the goals of SPRINT. An ancillary study may or may not use all randomized participants. Investigators are encouraged to propose and conduct ancillary studies. Such studies enhance the value and productivity of SPRINT and help ensure the continued interest of the diverse group of investigators who are critical to the success of the trial as a whole. These studies provide an exceptional opportunity for investigators, either within or outside of SPRINT, to conduct additional projects at relatively low cost. In general, ancillary studies will require additional funding from the NIH or other sources.

Application Review Process

To protect the integrity of SPRINT, all ancillary studies must be reviewed and approved by the SPRINT Steering Committee before access to SPRINT data, samples, or participants is permitted. Investigators will not be allowed access to the SPRINT participants, samples, or database without approval. New ancillary study proposals will be submitted to the SPRINT Ancillary Science (AS) Subcommittee, which will review all ancillary study proposals and make a recommendation to the Steering Committee. In the event that investigators wish to modify an ancillary science protocol that has already been approved by the SPRINT SC, they will need to first obtain AS Subcommittee and SC approval. All ancillary science proposals resubmitted to the AS Subcommittee must be comprised of a complete application form, including a complete narrative of the rationale and research plan, with changes highlighted. In addition, there should be a point-by-point response to the reviewers' comments. Ancillary study forms can be obtained by contacting the Coordinating Center or accessing the SPRINT website.

If an investigator with an approved and funded AS proposal would like additional biospecimens, they must submit a written request detailing their request and justifying the additional biospecimen. In addition the AS PI must provide a letter of approval from their project's Project Officer. The AS Subcommittee will decide if a more extensive review, beyond what can be accomplished with existing AS Subcommittee members, is necessary and will likely depend on the source of funding.

Studies submitted for approval less than four months prior to a funding application deadline may not receive timely approval. When the application is complete, the study proposal will be sent to the AS Subcommittee for review. The AS Subcommittee will

have monthly calls to discuss proposals, which will be circulated at least one week prior to the calls. After review and approval by the AS Subcommittee, approval/disapproval will be made by the Steering Committee. Ancillary Science investigators must include one or more SPRINT investigators in their ancillary study proposals.

The Coordinating Center will usually be responsible for all data management and analysis for all ancillary studies. Specialized expertise external to the Coordinating Center (e.g., processing of images) may be needed at the coordinating center's discretion. Costs associated with ancillary study data management and analysis must be budgeted into each ancillary study, even if the applicants have the necessary expertise in data management and analysis.

Prior to grant submission (or study initiation if no external funding is required), the CCN PI must approve participation of sites in her/his network. This is required as the CCN PI is responsible for the conduct of all aspects of SPRINT within her/his network. Part of this is management and oversight of clinic and participant burden. As needed, the CCN will include funding for oversight (e.g., investigator, coordinator, and fiscal personnel time, travel). SPRINT routinely monitors all ancillary studies to ensure that they do not significantly interfere with conduct of the main study. If an ancillary study is interfering with the main study, appropriate action, including the possibility of halting that ancillary study or other ancillary studies, may be taken. All ancillary study applicants must understand this provision. Investigators with approved ancillary studies will report the status of the studies annually to the Chair of the AS Subcommittee. More information may be needed in the interim. The AS PI must seek permission from the AS Subcommittee and the Steering Committee, prior to the conduct of the AS, for all significant changes made to the project, including but not limited to, change in investigator, performing clinic, specific aims, sample size, funding source and type and amount of biospecimens. After study initiation, investigators also need to notify the AS for these or similar changes.

Applicants should provide NIH biosketches of the AS PI and key Co-Investigators whose expertise and resources are essential for the AS. The applicants would need to make their best judgment on whose biosketches fit into that category and the AS Subcommittee may ask for additional biosketches. There is no need to submit biosketches of CCN PIs and Site PIs, if their involvement is limited to recruiting SPRINT participants and/or monitoring the sites. However, if the AS calls for very special techniques, equipment or other resources, the applicant should make her/his best judgment to decide if the CCN/Site investigators' biosketches and descriptions of equipment and resources would be helpful for the AS review.

A step-wise approach to statistical analysis, including power calculations should be used. First, the applicant should develop a rough sample size estimate and analytical plan prior to AS submission. If the proposal is approved by the AS Subcommittee, the CC will briefly review the plan to ensure that the sample size estimate is reasonable before submission to the SC. If the SC approves the proposal, the CC will work with the applicant to finalize the sample size for grant submission. If, during this process, it becomes clear that the sample size was substantially inaccurate, the revised sample size estimate and analytical plan will be sent back to the AS Subcommittee and SC for further deliberation.

Time Line for Submission to Funding Agency

The AS proposal must be submitted for funding within 9 months of notification of the approval by the Steering Committee; the SPRINT Steering Committee may issue the support letter to the applicant any time during these 9 months. Resubmission to the funding agency must occur within 9 months after the receipt of the funding agency's

critiques from the first submission. After this period or if the funding of the proposal has been declined twice for funding (either by the same or different agencies), the right to that particular topic is forfeited. Under unusual circumstances, exceptions to these guidelines can be made by the Steering Committee; however, adequate justification must be provided.

The AS Subcommittee will keep track of the status of individual AS applications and may choose to close the file of a particular proposal if there is no intention by the applicant to pursue the proposal further or if there is no tangible progress made or foreseen. The AS PI will be notified when an AS proposal is closed.

Additional Requirements of Ancillary Science Investigators

All ancillary study investigators will be required to budget adequately for all necessary resources for their studies. This includes, but may not be limited to, costs for data collection, sample collection, sample shipping, sample extraction, sample analysis, data entry, website development, data analysis, dataset preparation, data storage and publication of results. The final budget may be determined after AS and SC approval.

If an AS proposal will be resubmitted to a funding agency, the Principal Investigator is strongly encouraged to share the critiques of the previous submission from the funding agency with the AS Subcommittee. These critiques will only be available to the AS Subcommittee Chair and the CC's primary representative on AS, AS reviewers of that particular proposal and the SPRINT Executive Committee. If significant changes will be made in the Specific Aims, methodology, and/or burden to the main SPRINT study or other important aspects, the Principal Investigator must notify the AS Subcommittee of these changes and state clearly if these changes are made in response to the funding agency critiques. If an Ancillary Science PI is unsure if a change is significant, they are encouraged to contact the AS Subcommittee Chair or the CC's primary representative on AS to discuss. Re-approval by the AS Subcommittee and the Steering Committee must be obtained prior to resubmission to the funding agency. Re-approval is not guaranteed. Failure to do so may jeopardize the approval of the current and/or future AS Proposals.

The AS investigators should be aware that, even after the approval has been granted by the SPRINT Steering Committee and the funding agency, the timing of the release of the biospecimens, images and data will be decided by the Steering Committee and may be delayed for scientific and administrative reasons. The AS investigators should discuss this possibility with the AS Chair or the CC's primary representative on AS, even before submission of the grant to the funding agency.

Each ancillary study will cause an increase in utilization of main SPRINT study resources, particularly by the SPRINT Presentations and Publications (P&P) Subcommittee. To help with study operations, each ancillary science proposal team should budget for and will be asked to contribute efforts to the main SPRINT study by, for example, assigning a person to serve as a reviewer for the P&P Subcommittee, at the discretion of the Steering Committee. For AS proposals in which the Principal Investigator is already actively participating in Subcommittee functions, the assignment of additional personnel is not mandatory but encouraged.

Investigators proposing the use of laboratory measurements are encouraged to use the SPRINT Central Laboratory if at all possible. This will facilitate sample processing and shipping and may reduce the amount of sample required. Expertise, costs, and other factors should be taken into consideration in these decisions. The applicant is

encouraged to discuss with the AS Subcommittee Chair or the CC's primary representative on AS if the use of laboratories other than the Central Laboratory is contemplated, prior to finalizing the budget for grant submission.

The biospecimens collected by the AS belong to the AS investigators. However, AS investigators must transmit numerical data to the Coordinating Center for storage and sharing. In addition, AS investigators are strongly encouraged to share the images (e.g., MRI), tracings (e.g., ECG) and other data with other SPRINT investigators. Publication of any data that include any resources, including data and biospecimens, from the main study must be approved by the SPRINT P&P Subcommittee. The use of residual biospecimens collected by the AS beyond the intended use for the Specific Aims approved by SPRINT is up to the discretion of the AS investigators, but should follow the IRB policies of the individual institutions.

Reporting of AS to AS Subcommittee

After an AS proposal has been funded, the Principal Investigator must submit the Abstract and Specific Aims of the proposal approved for funding to the AS Subcommittee. These documents will become part of the AS application. In addition, the Principal Investigator must explicitly notify the AS Subcommittee and seek re-approval if the scope, methodology, and/or burden to the main SPRINT study or other aspects have changed from the time of SC approval to the approval by the funding agency. Approval of these changes by the AS subcommittee and the Steering Committee is not guaranteed.

The progress of the funded AS proposals should be reported to the AS Subcommittee on an annual basis and promptly when requested, for oversight and report to the DSMB. Additional information may be required by the AS Subcommittee in the interim.

Publication Policy

The purpose of the policy is to encourage and facilitate the presentation and publication of SPRINT Study background, rationale, design, and analyses; ensure appropriate use of the SPRINT data, timely completion of manuscripts and presentations, equitable access to authorship, and adherence to established principles of authorship; and coordinate the reporting of trial results. The policy applies to all investigators analyzing, presenting, and publishing data from main SPRINT, SPRINT-MIND, SPRINT-Senior (hereafter collectively called "SPRINT")and ancillary studies, except for those using the NHLBI Data Repository data (see https://biolincc.nhlbi.nih.gov/home/).

There are several principles underlying this policy:

A. Research questions and hypotheses to be addressed using SPRINT Study data should be formulated *a priori* and clearly stated in a manuscript proposal to reduce the likelihood that study results are attributable to type I error.

B. Publication of scientific findings from the SPRINT Study should proceed in a timely fashion once relevant analyses are complete.

C. The publications arising from the SPRINT Study should avoid overlap and conflicting representation of SPRINT Study findings. Overlaps are, however, acceptable for review articles.

D. Recognition through authorship will be distributed among the SPRINT investigators so that:
   i) all SPRINT investigators and team members have equitable opportunity to lead and co-author SPRINT publications and, if appropriate, publications from ancillary studies;
   ii) all Ancillary Study investigators have the opportunity to lead and be co-authors on publications resulting from their ancillary studies.

E. The SPRINT Study should promote the career development of trainees and junior faculty by providing them the opportunity to lead and be recognized as co-authors of SPRINT publications, as appropriate.

F. Standards for authorship on SPRINT publications will adhere to the Uniform Requirements for Manuscripts Submitted to Biomedical Journals of the International Committee of Medical Journal Editors (NEJM 1997;336:309-315) and those established by the destination journals.

G. The concept, in the form of a proposal, for all manuscripts must be approved by the P&P Subcommittee prior to preparation.

There are three categories of manuscripts and anticipated authorship:

i) Main results developed based on core SPRINT data and study aims/hypotheses (which will bear the corporate authorship, “The SPRINT Research Group”). The design and main baseline papers will also be corporate authored.

ii) Manuscripts developed and authored by investigators using data that are not considered to be main SPRINT results.

iii) Ancillary study results led by investigators bringing external funding or resources into SPRINT for a specific project.

   (1) Unless specific justifications and alternative arrangements are made, all SPRINT analyses will be performed by the Coordinating Center (CC), with specialized expertise external to the Coordinating Center as needed at the Coordinating Center's discretion. Ancillary study budgets should include funds allocated to the CC for that purpose.

   (2) Ancillary study manuscripts are subject to similar review and tracking procedures as other SPRINT manuscripts.

During the operational phase of the trial, manuscripts proposing to use data other than baseline data will be reviewed closely to ensure that the SPRINT study objectives are not compromised. In general, the following will not be allowed:

(1) Publication of follow-up data according to randomized group

(2) Longitudinal analyses of outcomes pre-specified in the main protocol

All such proposals will be considered on a case-by-case basis, with input from the Data and Safety Monitoring Board.

The final responsibility for review and approval of manuscript proposals, including composition of writing committees, readiness for submission, and abstracts and material for presentations at meetings and conferences, rests with the Steering Committee. The P&P Subcommittee will oversee and facilitate these processes, assisted by a Publications Coordinator based at the Coordinating Center.

2. Conflict of Interest Policy

SPRINT Conflict of Interest Policy

General Principles

- All of the study PIs, Co-PIs, and other members of the Steering Committee and its various subcommittees are covered by this policy.

- The primary concerns are twofold: (1) that we maintain the internal integrity of the study, by which we mean the confidence among SPRINT investigators and staff that advice is being given and decisions are being made in as unbiased and fully informed manner as possible; and (2) that we maintain the external integrity of the study, by which we mean that our process and results have met public standards of conduct. Accordingly, this conflict of interest policy will be on the public part of the SPRINT website and referenced in publications when possible.

- It is recognized that SPRINT is not testing specific drugs or medical devices. Nevertheless, we have adopted a policy similar to those that have proved useful and workable in other major NIH-sponsored multicenter trials.

Specific Goals

1. To meet the goals listed above under General Principles, we will obtain disclosure of financial relationships judged to have an active or potential interest in the conduct and outcome of the study. These are to be reported on a standard form, which will be reviewed on at least an annual basis, or more frequently if there is a significant change from the last report, by a subset of the Executive Committee (termed the SPRINT Conflict of Interest Committee). The Conflict of Interest Committee will be comprised of the Chair of the Steering Committee, the PI of the Coordinating Center, and the NHLBI Project Officer. If any member of this Committee has financial disclosures requiring review, he or she will be recused from the review of his own form and replaced by the CCN PI currently serving on the Executive Committee. The information to be reported will be detailed, and the level of compensation will be categorized in broad classes (see 3a below).

2. All financially relevant relationships are to be reported. These relationships include but are not limited to employment, consultancies, board and committee memberships, honoraria (if received directly from industry or a company acting on behalf of an industrial sponsor, or as a result of a CME program funded directly to the individual by a single industrial sponsor), stock ownership or options, grants, contracts, patents received or pending, and royalties. The Conflict of Interest Committee will decide whether any of these and other relationships in each individual case are significant enough to warrant recusal from voting, discussions, or authorship (see paragraph 5 below). Only those relationships that are between the individual and the specific company (rather than between the individual's parent institution and the specific company, for

example) present the potential for a significant financial conflict of interest, defined under paragraph 3 below. Specifically, research funding for contracts or grants to the parent institution that provides support to the individual, his/her laboratory, or his/her close scientific collaborators is not ordinarily judged to present the potential for a financial conflict of interest, although such awards are to be fully disclosed as a part of this policy.

3. A significant financial relationship is defined to exist in the following instances:

a. When the dollar amount awarded on an annual basis, or expected-to-be awarded on an annual basis, with regard to any related corporate relationship exceeds $25,000 at the time of disclosure. The Conflict of Interest Committee may also judge lower dollar amounts as significant in specific/individual circumstances. The level of compensation will be categorized as follows:

≤ $25,000
> $25,000 and ≤ $100,000
> $100,000

b. When there is any equity holding of $10,000 or more in a related company (excluding mutual funds and blind trusts). The level of equity holdings will be categorized as follows:

≤ $10,000
> $10,000 and ≤ $25,000
> $25,000 and ≤ $100,000
> $100,000

4. Significant financial relationships in existence between SPRINT investigators and all pharmaceutical and biomedical companies judged to have an active or potential interest in the conduct and outcome of the study will be described in all study reports and publications. Similar relationships, but which are not significant, as well as actions taken early in the design phase of SPRINT that end significant financial relationships (e.g., stock divestment) will all be described on the SPRINT website, but will not ordinarily be listed in study reports or publications. In addition, we will meet or exceed the reporting standards of the journals publishing our manuscripts.

5. Consequences: The Conflict of Interest Committee will decide whether a financial relationship is significant enough to warrant any consequences. These cover a range of actions.

    a. A conflict of interest will not necessarily exclude any member of the study from participating in study *discussions*, unless required by the Conflict of Interest Committee in individual cases of significant financial conflict. However, full disclosure of all significant financial conflicts of interest will be made at each Steering Committee meeting to all attendees in an effective but non-cumbersome manner.

    b. A *significant* conflict of interest, defined above, will cause a person to recuse himself or herself from *voting* on all issues related to the conflict. All such actions will be recorded and kept as part of the study record in the Coordinating Center. This policy applies to deliberations of the Steering Committee and its subcommittees.

c. Any individual who fails to submit conflict of interest forms will not be permitted to participate in Steering Committee or subcommittee votes or discussions or to participate on any paper-writing groups. The forms should be completed carefully, and omission of information may lead to suspension of participation in trial-wide activities or exclusion from one or more writing groups. Individuals will also be responsible for updating conflict of interest information at the time of acceptance of a manuscript in accordance with journal policies.

d. If the Conflict of Interest Committee determines that a significant financial relationship exists, the Committee will take action to limit the potential impact of such conflict. This could include not being eligible to serve on writing teams of specific manuscripts.* The involved individual may appeal the proposed remedy to the full Executive Committee and subsequently to the Steering Committee.

* Such exclusions will ordinarily be based on the most recent relationships reported at the time a writing committee is formed. However, because work on a paper may continue for several years, participation may be modified if new significant relationships are reported subsequently.

Chapter 20. Regulatory Information

Every SPRINT CCN and clinical site, as well as the SPRINT Coordinating Center, must obtain approval from an Institutional Review Board (IRB) before conducting screening visits and enrolling participants in the study. An IRB is charged with the review of all research, development, and related activities in which human subjects will participate in that institution. All study protocols are reviewed for purposes of:

- approving the appropriateness of methods used to secure informed consent;
- protecting the rights and welfare of study participants;
- evaluating the risks to subjects in relation to the potential medical benefits of the investigation.

IRB Website Tracking
All IRB submissions, approvals and annual continuing reviews will be tracked on the SPRINT web site. The tracking report is located under the “Data Management” tab. After selecting IRB Tracking, you will have the choice of running a summary report or entering data for the sites you have access to. The date fields include the date of expected submission, date of submission, date of review and date of approval. The Coordinating Center will generate an upload utility whereby all approval memos are required to be uploaded upon IRB approval. This utility also includes annual continuing reviews.

To run a summary report, choose an IRB Event from the drop down menu and then click the GO button. Currently there is only one IRB Event listed, but over time there will be more to choose from. This report will list all clinical sites and the status of various tracking dates. All clinical site IRB information will be visible to all SPRINT study staff.

To enter data for a clinical site, choose a site from the drop-down menu. This list contains only the clinical sites for which you have access. If you do not have access to a clinical site that you should, please notify the Coordinating Center.

Training in the Protection of Human Subjects
The NIH requires training in the protection of human research participants for all investigators and study staff who submit NIH applications for grants or contracts, as well as those investigators who receive new or non-competing awards for research involving human subjects. This requirement applies to SPRINT. For more information please see the following web site:
HYPERLINK http://grants.nih.gov/grants/policy/hs http://grants.nih.gov/grants/policy/hs educ faq.htm

20.1 What needs IRB Approval?

An IRB is constituted to review and approve studies to be carried out on human subjects in that institution. The review focuses on the ethics of the proposed research and on the adequacy of the proposed patient informed consent process. Some local IRBs are also initiating certification for programs on Good Clinical Practice (GCP) Guidelines that each investigator may need to receive prior to study approval and initiation. These efforts coincide with federal guidelines for education and training of clinical investigators, IRB members and associated IRB and institutional staff.

Patient-oriented research studies need to be approved by an IRB before any participant examination or data collection can begin. Once a study has been approved, any additional information about the study that relates to participant safety (i.e., protocol changes, significant adverse events, changes in the consent form) also needs to be submitted to the IRB. Retrospective chart reviews with patient identifiers, as well as study recruitment ads, brochures, phone scripts and any materials that would be sent or given to potential participants must also be reviewed and approved by an IRB. All IRBs must follow national principles for assuring human subject safety, but each IRB is unique and may have specific requirements, for example on the structure of an informed consent document.

For local approval, the IRB will be provided all of the following documentation as deemed appropriate by the local institution:

- Study protocol and any future revisions or amendments;
- Informed consent documents;
- Any required local organizational approvals for the conduct of the study;
- Research subject advertisement including posters, press releases, videos, Internet WEB page and flyers;
- Patient brochures, pamphlets and guides, or form letters;
- Phone call scripts (If any phone calls are made to prospective patients to tell them about the trial and solicit interest, the individual IRB should be given a "script" of what will be said to the person); and
- Data Collection forms and explanation of electronic data transmission procedures.

20.2 Full versus Expedited Review/Approval

Any study involving more than minimal risk of harm to human participants needs to undergo a full review by the entire IRB committee. The term “minimal risk of harm” refers to being exposed to nothing more than everyday occurrences of routine procedures for standard health care. Studies involving only collection of blood samples from healthy individuals, collection of hair and nail clippings, and research involving data, documents of records that have already been collected, for example, may be eligible for expedited review. Other documents that may qualify for expedited review include recruitment brochures, advertisements, and form letters to participants.

20.3 Interim IRB Review/Approval

After initial approval, an IRB must be notified any time about:

- Recruitment Brochures and Advertisements,
- Form Letters or Study information sent to participants,
- Protocol Amendments,
- Protocol Deviations,
- Serious Adverse Events (SAEs),
- Consent Form Revisions,
- Other issues required by local reporting requirements.

In addition, an IRB must be notified of serious adverse events after DSMB review and discussion of those events. The Project Office and Coordinating Center will prepare a report after each DSMB discussion for PIs to convey this information to their IRBs.

20.3.1 Recruitment Brochures and Advertisements

Before circulating study brochures/fliers, placing ads in newspapers (including other publications and on a web page), or placing any kind of public service announcements about the study on TV or radio, all individual IRB's must approve the text/script of these materials. If any of these materials are developed by the Coordinating Center, they must then also receive IRB approval prior to use. Key elements that the IRB members look for when reviewing these ads include:

- Use of the word "research",
- List of basic eligibilities,
- No "overpromising" with respect to the results of the study.

20.3.2 Protocol Amendments and Consent Form Revisions

All protocol changes must be sent to an IRB as protocol amendments. Minor changes that do not affect participant care may be considered for expedited review. Call your clinical site's research office to inquire about their specific, local requirements. Most amendments necessitating a change in the consent form need to undergo full IRB review; however changes in the consent form for clarification may be eligible for expedited review.

20.4 Adverse Events

A serious adverse event (SAE) is defined as any adverse experience that is significantly life threatening and/or results in death, permanent disability, hospitalization or prolongation of hospitalization, whether or not the event is related to study interventions. Also included, as a serious or unexpected adverse event, would be congenital anomalies, cancer or overdoses due to the medication.

Unexpected adverse events refer to adverse experiences that are not listed in the current labeling for the drug. These may be symptomatically and pathophysiologically related to an event listed in the labeling, but differ from the event because of the greater severity or specificity (e.g., hepatic necrosis constitutes an "unexpected event" by virtue of greater severity if labeling refers only to elevated hepatic enzymes or hepatitis).

The SPRINT study also considers any ER visits for heart failure, problem with heart rate, stroke, TIA, or electrolyte problems, and any syncope or injurious falls as serious adverse events.

Local IRBs must be notified about any serious or unexpected adverse event that the PI feels is directly caused by a study medication or participation in the study. The SPRINT data collection process incorporates the collection of information on all serious and unexpected adverse events.

All SAEs need to be data entered <u>within 72 hours</u> of discovering the event. Supplemental information will be requested if necessary. See the Safety Monitoring and Reporting MOP Chapter 14 for detailed information.

20.5 Record Keeping of Regulatory Documents

<u>Regulatory responsibilities</u> for the CCNs, clinical sites, Coordinating Center and Sponsors are noted below:

The regulatory responsibilities of the SPRINT Clinical Center Network are to:

- Assure the study sponsor that IRB review is being carried out in accordance with federal regulations;
- Assure that CCN staff are certified in the conduct of human subjects research;
- Oversee clinical sites to ensure Good Clinical Practices are conducted;
- Obtain IRB approval for the network hub office;
- Assist clinical sites with all IRB submissions and approvals, annual continuing reviews, protocol amendments and protocol deviations;
- Review each site's informed consent document for verification of the required essential elements (see section ???? below);
- Assist clinical sites with the uploading of all required regulatory documents to the study web site (Initial IRB approval letter, annual continuing review approval letters, approved informed consent documents, protocol amendment approval letters);
- Maintain the CCN Office Regulatory Binder and review the clinical sites' Regulatory Binder for the presence of required documents.

The regulatory responsibilities of each clinical site are to:

- Assure the study sponsor that IRB review is being carried out in accordance with federal regulations;
- Assure that clinical site staff are certified in the conduct of human subjects research;
- Ensure Good Clinical Practices are conducted;
- Ensure that the participants understand the purpose of the study, risks and rights, study procedures and visit schedules as stated in the informed consent document(s);
- Obtain and document informed consent and HIPAA authorization on all participants prior to any study procedures;
- Work with the CCN Hub office in all IRB submissions;
- Upload all IRB approval letters, annual continuing review approval letters, approved informed consent documents and any pertinent IRB materials to the SPRINT web site IRB tracking utility;
- Assure all visit procedures are implemented as defined in the protocol
- Assure participant safety with respect to the implementation and conduct of the trial.
- Assure compliance with all drug accountability and handling procedures.
- Obtain documentation of local lab CAP certification.
- Maintain the site Regulatory Binder.
- Comply with all regulatory and local IRB reporting requirements.

The regulatory responsibilities of the Coordinating Center (CC) are to:

- Assure the study sponsor that IRB review is being carried out in accordance with federal regulations;
- Assure that CC staff are certified in the conduct of human subjects research;
- Obtain initial IRB approval and annual continuing review IRB approval for the Coordinating Center;
- Provide template and CC IRB-approved informed consent documents, protocol and protocol amendment documents to the CCNs for circulation to clinical sites;
- Provide a document that lists the essential elements of informed consent to assist the networks in monitoring the sites;

- Maintain the CC Regulatory Binder;
- Monitor the annual IRB status of networks and clinical sites;
- Provide a website utility that allows clinical sites and CCN coordinators to upload all IRB approval letters, annual continuing review approval letters and IRB informed consent documents;
- Periodic quality control review of the uploaded informed consent documents at every clinical site;
- Send information to the CCN offices if the clinical site or network annual renewals have expired.

The regulatory responsibilities of the Sponsor include:

- Random reviews of the clinical sites' IRB approved informed consent documents and other regulatory issues as deemed appropriate.

20.5.1 SPRINT Regulatory Binder

The Study Regulatory Binder is the administrative binder that serves as the regulatory record of each clinic's participation in the SPRINT study. It should be kept current and available for review by the Clinical Center Network Coordinators and/or representatives of SPRINT agencies during site visits or in the event of an audit. Suggestions for the contents of the binder include current copies of (but depends upon local requirements):

- Protocol and revisions;
- All protocol amendments;
- IRB submissions and approvals, IRB renewals and any submitted protocol deviations and log, IRB correspondence (adverse events reports);
- Copies of IRB approved informed consent document(s);
- Research participant advertisements, (e.g. patient brochures, pamphlets) patient education materials, newsletters, etc.
- Current correspondences relating to human subjects research (may keep separate correspondence file);
- Site visit log;
- Enrolled patient log with pertinent identifier information (randomization number if needed).
- List of study drug formulary and package inserts

Other items that may also be included are: recruitment plans, a set of study forms, CVs or biosketches for study staff, staff responsibility logs, essential elements of informed consent checklist, protocol deviation logs, or other local requirements from your institution.

Required Elements in SPRINT Informed Consent Documents

General Issues

☐ Study involves research, voluntary participation with no penalty/loss of benefits

☐ Purpose of the study

☐ Description of study design, expected duration, randomization, treatments received by randomized groups, approximate number of subjects enrolled at this site and study-wide

☐ Visit type and frequency

☐ For research involving more than minimal risk, an explanation as to whether any medical treatments are available if injury occurs and, if so, what they consist of, whether there will be any compensation for injury and where further information may be obtained:

  ☐ Emergency care resulting from study treatment provided

  ☐ Care for problems resulting from study treatments not paid for by the study

  ☐ Participant notification that:

  ☐ Changes to current treatment will likely be made

  ☐ Other non-study clinical care is to be provided by participant's own physician

  ☐ Alternative treatments are available and will be described

☐ No guarantee of personal benefit

☐ Any additional costs to the subject that may result from participation

☐ Right of participant to discontinue participation; consequences and procedures

☐ Right of study to discontinue the participant's participation; circumstances

☐ A statement that significant new findings developed during the course of the research which may relate to the subject's willingness to continue participation will be provided to the subject

☐ Genetic studies – purpose, storage/sharing of DNA, privacy, samples donated, layered consent allowing different degrees of participation, with mutually exclusive choices

<u>SPRINT Specific Issues and Risks of participation/ treatments</u>

☐ Risks from blood drawing

☐ Risk of hypotension or orthostatic hypotension

☐ Potential side effects/risks from the classes of study medications used

<u>Administrative/confidentiality Issues</u>

☐ Data are confidential, no participant identifiers released

☐ Name, SSN, Medicare numbers collected by the study

☐ Records can be reviewed by NHLBI, FDA, CCN, IRB, CC Records locked, stored and destroyed per study and/or local regulations (whichever is longer)

☐ Data and specimens are sent to NHLBI and may be shared with other investigators

☐ U.S. Certificate of Confidentiality

☐ Contact name and number for questions

☐ Contact name and number for questions related to participant rights (i.e., IRB name/number)

An example of a form that sites can use to document the informed consent and process is below; please check with your IRB for local requirements.

DOCUMENTATION OF INITIAL CONSENT PROCESS

Study Name: ____

Protocol: ____

Patient Number: ____

Patient Initials: ____

Date of Consent: ____

___ Patient / subject was seen and referred by delegation of principal investigator

___ Consents (including HIPAA form) were reviewed with the subject

___ All questions / concerns were answered or addressed

___ Verified an adequate level of comprehension by asking the subject to restate his/her understanding of the research:

- ___ **Goal of the Research and Protocol:** "Tell me in your own words about the goal of this research and what will happen to you if you agree to be in this study."
- ___ **Benefits and Compensation:** "What do you expect to gain by taking part in this research?"
- ___ **Risks:** "What risks would you be taking if you joined this study?
- ___ **Voluntariness:** "What do you think will happen to you if you refuse to be in this study?"
- ___ **Discontinuing Participation:** "What should you do if you agree to be in the study but later change your mind? What will happen to information already gathered if you change your mind?"

___ Reviewed any misinformation (e.g. "Let's talk about the goal of the study again because I think I have not explained the project clearly."

- ___Adequate level of comprehension has been confirmed
- ___Not eligible due to lack of comprehension

___ Consents were signed and dated

___ A copy of signed consents were given to patient / subject

Signature of Person Obtaining Consent: **Date:**

Signature of Principal Investigator: **Date:**

Close-out Manual of Procedures

Close-out Manual of Procedures
Table of Contents

Section 1. Close-out Calendar 1

Section 2. SPRINT Website 1

Section 3. Close-out Visit Information...1

3.1 Close-out Visit Timeline and Overview 1

3.1.A. Clinic Pre-Close-out Preparations (November 2015) 1

3.1.B. Activation to Begin Close-out Visits...3

3.1.C. Participant Pre-Close-out Preparations 4

3.1.D. One week prior to the participant's scheduled close-out visit: 4

3.1.E. At the close-out visit:...5

3.1.F. Following the close-out visit, participant close-out:..6

3.1.G. Clinic close-out:...7

3.2 Close-out Encounter & Disposition Form 7

Section 4. Close-out Procedures by Status 7

4.1 Close-out Procedures by Contact Type 8

4.1.A. Clinic or Home Visit..8

4.1.B. Phone Visit 8

4.1.C. Visits by Proxy 9

4.1.D. No Visit...9

4.1.E. PRN Visits 9

4.1.F. Late Data Collection and Split Visits...10

4.2 Lost to Follow-Up Participants 10

4.3 Withdrawn Participants 11

4.4 Deceased Participants 11

Section 5. MIND and MRI Visit Procedures... 12

5.1 MIND Procedures.. 12

5.2 MRI Procedures 13

Section 6. Documents for Participants and the Close-out Participant Kit..13

6.1 Generic Materials 14

6.2 Certificate of Appreciation 15

6.3 Healthcare Provider Letter 15
6.4 SPRINT Study Medical Summary Report........ 15
6.5 Guide to Assistance Programs 15
6.6 Notification of Study Results to Participants 16
Section 7. Helping Participants find a Healthcare Provider or Insurance 16
Section 8. Guide to Assistance Programs (GAP) Information 16
Section 9. Medications........ 16
9.1 Manual Orders for Medications 17
9.2 Mailing Medications to Participants after a Phone Visit........ 18
9.3 Destroying Study Drugs 18
Section 10. Laboratory Kits and Specimen Collection 18
10.1 Laboratory Kits 18
10.2 MRI-CBC Study........ 19
Section 11. Outcomes and Serious Adverse Events 19
11.1 Outcome Collection 19
11.2 Serious Adverse Events........ 19
11.3 Reporting Deaths 20
11.4 Medical Records 20
Section 12. Close-out Reports and Tools 20
12.1 Reports to Review Prior to Close-out........ 20
12.2 Death Records Search Report 20
12.3 Close-out Visit Scheduling Aid 21
12.4 Participant Close-out Checklist 21
12.5 Monitoring and Aggregate Reports........ 21
12.6 Cleaning Data 21
Section 13. IRB Information 22
Section 14. Equipment Information 22
Section 15. Data and Record Storage 22
List of Appendices........ 24
APPENDIX 1 – Definitions 25
APPENDIX 2 – Measures and Frequency 26

APPENDIX 3 – Drug Order Form (Manual Orders).. 28
APPENDIX 4 – Lab Supply Reorder Form.. 30
APPENDIX 5 – Computer Search Tips... 32
APPENDIX 6 – Internet Resources for Finding Lost to Follow-up Participants.................................. 33
APPENDIX 7 – Close-out Reminder Letter for Lost to Follow-up Participants (Participant) 35
APPENDIX 8 – Close-out Reminder Letter for Lost to Follow-up Participants (Proxy) 36
APPENDIX 9 – Permission to Contact You about Health Status 37
APPENDIX 10 – Addendum to Consent Form.. 38
APPENDIX 11 –Close-out Appointment Reminder Letter 40
APPENDIX 12 – Close-out Encounter and Disposition Form 41
APPENDIX 13 – Close-out Blood Pressure Management Form 47
APPENDIX 14 – Close-out Visit A Lab Form.. 49
APPENDIX 15 – Close-out Visit B Lab Form.. 51
APPENDIX 16 – MRI CBC Lab Form 53
APPENDIX 17 – Tips for Finding a Healthcare Provider or Insurance 55
APPENDIX 18 – Letter to Healthcare Provider 56
APPENDIX 19 – Study Medical Summary 57
APPENDIX 20 – Certificate of Appreciation 61
APPENDIX 21 – Participant Bulletin... 63
APPENDIX 22 – Drug Transfer Form (Clinic to Clinic) 69

Section 1. Close-out Calendar

October/November 2015	Submit close-out protocol and close-out materials to IRB
October 31, 2015	Last day for central phone calls for event ascertainment
November 13, 2015	Complete drug inventory, including destroying expired SPRINT drugs
December 1-3, 2015	Close-out Training Meeting (Atlanta, GA)
December 7, 2015	Close-out visits begin (site by site basis)
January 11, 2016	Par levels “zeroed”; only manual orders accepted by DDC
June 30, 2016	Last day for close-out visits
July 31, 2016	Last day for MRI visits
	Forms data entry complete *(No new form submissions allowed)*
August 31, 2016	Final data queries complete *(No more changes to forms allowed)*
	Final outcomes documentation due
September 30, 2016	All site activities related to close-out complete
	Last day for central phone calls for MIND telephone battery
2017	Participants are notified of additional study results

Section 2. SPRINT Website

Templates and documents related to the close-out visit are available on the SPRINT website under Documents >> Close-out Materials. Reports specifically designed to monitor close-out activities are available under Reports >> Close-out Reports. Additional tools related to close-out are integrated into the SPRINT study website (e.g., via data management menu, the participant status page). Sites should contact their CCN Coordinator if they have questions about the close-out materials on the website. Definitions listed throughout this document are described in Appendix 1.

Section 3. Close-out Visit Information

3.1 Close-out Visit Timeline and Overview

Close-out visits will occur after the site has been activated by the Coordinating Center (CC) to begin close-out visits and no later than June 30, 2016. Once a site has been activated by the CC (see section 3.1.B), all visits at that site will become close-out visits. End window dates for all close-out visits are June 30, 2016.

In planning for close-out visits to begin, it will be helpful to understand the two primary categories of visits (Close-out A and Close-out B). Measures to be collected are outlined in Appendix 2. Depending on whether the participant has completed his/her 48 month visit at the time of close-out, the close-out A visit forms packet will be available or the close-out B visit forms packet will be available (see Section 3.1.E).

3.1.A Clinic Pre-Close-out Preparations (November 2015)

- Submit Protocol 5.0 and all close-out materials to the local IRB. These documents are posted on the SPRINT website under Documents >> Close-out Materials. The CC IRB Approval Letters also are available in this folder if they are required for local IRB submissions. Translated documents only need to be sent to the clinic IRB if there are Spanish speaking participants at the clinic. Documents to be submitted are:
    - Protocol 5.0 (Clean, Track Changes, Summary of Changes)
    - Addendum to Consent Form (English and Spanish)
    - SPRINT Study Medical Summary Report

    - Letter to Health Care Provider (English and Spanish)
    - Close-out Appointment Reminder (English and Spanish)
    - Close-out Appointment Reminder for Lost to Follow-Up (Participant) (English and Spanish)
    - Close-out Appointment Reminder for Lost to Follow-Up (Proxy) (English and Spanish)
    - Tips for Finding a Healthcare Provider or Insurance (English and Spanish)
    - Permission to Contact You about Health Status (English and Spanish)
    - Certificate of Appreciation (English and Spanish)
    - Participant Bulletin (English and Spanish)
    - Guide to Assistance Programs
    - Encounter and Disposition Form
    - Close-out BP Management Form
- Complete drug inventory reconciliation by November 13, review the reports available on the SPRINT website (Data Managements >> Pharmacy >> Drug Reporting) and update par levels as needed in order to ensure adequate supplies to distribute a 3 month supply to participants, while also minimizing waste. Beginning January 11, only manual orders will be accepted by the Drug Distribution Center. See Section 9 for instructions about ordering drugs after this date and Appendix 3 for the Drug Order Form (manual orders).
- Ensure that MIND testing supplies are adequate. Contact the MIND Coordinating Center to reorder supplies if necessary (e.g., Digit Symbol Coding, HVLT-R Test booklets, Trail Making participant worksheets).
- Ensure blood collection supplies are adequate. The current “6, 36, 60 month” kits can be used for close-out A visits and the “12, 24, and 48 month” kits can be used for close-out B visits. Sites should first use up any “6, 36, 60 month” and “12, 24, 28 month” kits that they have available and then order additional close-out A and B kits as necessary. (See Appendix 4 for *Lab Supply Reorder Form*.)
    - For sites with MRI participants (either 640 MRI participants or Mind the Kidney participants), lab collection supplies to collect the complete blood count (CBC) on all MRI participants were mailed in November 2015. If you need additional supplies related to the CBC collection, please contact the central laboratory. See Section 10.2 for instructions about the MRI CBC Study.
- Ensure ECG is working appropriately and supplies are adequate; reorder supplies if necessary. (See MOP Chapter 8.)
- Ensure OMRON is working appropriately. (See MOP Chapter 3.)
- Complete a Death Records search:
    - Review the Death Records Search Report (Reports >> Close-out Reports). This report will list all participants at your site that are not known to be deceased but have withdrawn consent, been classified as lost to follow-up (LTF), or missed their last visit.
    - For all LTF participants or participants who missed their last visit, search available records (review electronic medical records as available, review online obituaries, etc.) to determine whether the participant has died.
    - For withdrawn participants, review online obituaries and other sources to determine whether the participant has died.
    - For tips on locating all SPRINT participants, see Computer Search Tips (Appendix 5) and Internet Resources for Finding Lost to Follow-up Participants (Appendix 6). Both of these documents are available under Documents >> Close-out Materials.
    - Complete a Serious Adverse Event (SAE) form if it is determined that a participant is deceased.

- If you do not find that the participant has died:
    - For withdrawn participants, document the searches used in Part D, "Comments" on the Participant Status Log.
    - For LTF participants, document the searches completed on the Participant Status Log by updating the date in Section B and document the searches used in Part D, "Comments" on the Participant Status Log.
    - For participants who missed their last visit, continue searching for these participants until June 30, 2016. Use the participant notes section to document the searches that were completed. If the clinic is still unsuccessful in contacting the participant, on June 30, complete the Close-out Encounter and Disposition Form indicating a missed visit.
  - A Death Records search will also be completed prior to clinic close-out for all participants who missed their close-out visit (including withdrawn and lost to follow-up participants).
  - Any death of a participant (including withdrawn and lost to follow-up participants) who are identified as deceased through the Death Records Search or National Death Index (NDI), will be assigned an outcome ID. Records collection and adjudication will follow the usual process for the outcome.
- For participants who have agreed to end of study contact only or participants categorized as lost to follow-up, send Close-Out Reminder Letter for Lost to Follow-up Participants (Appendix 7) in the mail. At the site's discretion or if the letter to the participant is returned as undeliverable, send the Close-out Reminder Letter for Lost to Follow-up Participants specific to the proxy (Appendix 8).
  - These letters should not be mailed until the clinic has received IRB approval.
  - Call the participant and/or his/her proxies and use all available sources to try to locate these individuals for a close-out contact.
  - For tips on finding all SPRINT participants, see Computer Search Tips (Appendix 5) and Internet Resources for Finding Lost to Follow-up Participants (Appendix 6). Both documents are available under Documents >> Close-out Materials.
- For withdrawn participants, if allowed and approved by your local IRB, send Permission to Contact You about Health Status letter (Appendix 9) in the mail as soon as possible.
- Prior to activation, please ensure that the Participant Status Log has been completed on all participants classified as lost to follow-up or withdrawn consent.

3.1.B. Activation to Begin Close-out Visits

- Once IRB approval is obtained for all close-out documents, upload the IRB approval letter (Site Admin >> IRB Uploads >> REQUIRED Close-out Protocol Amendment Submissions).
- The Addendum to Consent Form (permission to contact participants about future research; Appendix 10) is uploaded under Site Admin >> IRB Uploads >> Consent Forms >> Addendum to Consent form (Close-out).
- Ensure all data entry is up to date. Review the following reports:
  - Unresolved Visit Report (Reports >> Clinic Operations)
  - Participants with E&D for current visit printed but not yet entered (Reports >> Close-out Reports)
  - Participants in or past 48M with missing MIND data (Reports >> Close-out Reports)
- CCN staff will contact the clinic to confirm all data entry is complete.
- CCN staff will notify CC staff when a site is ready to be activated.

- It is strongly recommended for clinic staff to contact their CCN Coordinator on the Monday prior to the date the clinic wishes to be activated. Some back and forth regarding data entry completion is to be expected.
- Sites will only be activated on Friday afternoons. The Coordinating Center will notify the clinic and the CCN when a site has been activated. At this point, all future visits (with the exception of PRN visits needed for safety concerns) become close-out visits.
- Clinic staff can continue to see participants for regular visits up until the Friday prior to activation. When contacting the CCN staff prior to activation, provide the participant IDs for those participants for whom a visit will be conducted that week.
- Prior to activation, the CCN Coordinators and CC will check to make sure of the following:
    - The clinic has a certified MIND technician
    - The IRB approval letter for close-out has been uploaded
    - The IRB approved consent addendum has been uploaded
    - Data entry is up to date, specifically for the "Participants with E&D for current visit printed but not yet entered" report; if the clinic has printed forms, but the visit was not/will not be completed, the clinic must confirm that these forms have been destroyed
    - The clinic has provided a list of participants who are scheduled for the week prior to activation.
- Clinic staff should review the Scheduling Aid and reschedule participants as needed to accommodate the measures that will be collected at the close-out visit.

3.1.C. Participant Pre-Close-out Preparations

- Transition visits occur between September 9, 2015 (the date that clinic staff were notified of the decision to stop the SPRINT intervention) and the date that the site begins close-out visits.
- During transition visits, the site should discuss with the participant the end of the SPRINT study and determine whether the participant has a health care provider (HCP) and/or health insurance.
- Review Close-out Visit Scheduling Aid (Data Management >> Select a clinic from the list of available clinics >> Scheduling Aid) to ensure adequate time is available for each close-out visit.
- Ensure appropriate staff is available for each close-out visit and reschedule visits if necessary.
- In order to provide the participant the most accurate information on the SPRINT Study Medical Summary Report, review the below listed reports and take appropriate steps to resolve ALL outstanding data entry or missing information from prior visits before close-out.
    - Data Validation (Data Management >> Data Validation >> By Site)
    - List of Unresolved Data Entry (Reports >> Clinical Operations)
    - List of Unresolved Visits (Reports >> Clinical Operations)
    - List of Visits with Missing Lab Data (Reports >> Clinical Operations)
- Send the Close-out Appointment Reminder Letter (Appendix 11) in the mail, including what to expect for the close-out visit, information about whether visit is required to be fasting and reminders about things to bring to the close-out visit.

3.1.D. One week prior to the participant's scheduled close-out visit:

- Sites will call each participant and remind them of the appointment, including instructions regarding:
    - Expected length of the visit,
    - Fasting, if required,
    - Bringing all of their medications to the clinic, including their SPRINT medications, and
    - Bringing information on the HCP that will be taking care of health care needs after the close-out visit.
- Prepare Participant Kit and close-out materials for participant (see Section 6).

3.1.E. At the close-out visit:

- Depending on whether the participant has completed their 48M visit at the time of close-out, close-out visit A forms and measures will be completed or close-out visit B forms and measures will be completed. The appropriate forms and measures associated with the visit type will be displayed based on the data entry prior to the participant's close-out visit.
- Based on the close-out visit (A or B) and subgroup assignments, all expected procedures and forms must be completed. The list below encompasses all possible procedures and forms that may be expected in a close-out visit and are grouped by expectations for a close-out visit type and subgroup.
- In clinic visits are always the preference. See Section 4.1 for instructions about data collection for phone and proxy visit.
- If the participant has not yet completed the 48M MIND testing, the MIND Screening and/or Extended Battery will be completed at the close-out visit. The appropriate forms will be displayed based on the data entry prior to the participant's close-out visit. If the MIND Screening Battery is completed at the close-out visit and the participant triggers, the participant will need to be scheduled to return (prior to June 30th) to the clinic to complete the MIND Extended Battery (See Section 5).
- If the participant is in a substudy, and forms and measures are expected to be completed at close-out, these forms will be displayed.
- The following procedures are completed regardless of whether the participant is completing close-out visit A or close-out visit B and will always appear in the close-out visit packet:
    - **Close-out Encounter and Disposition Form** (Appendix 12)
    - **Events Ascertainment Form** (Event Forms and SAE form as needed based on Event Ascertainment Form)
    - **Close-out BP Management Form** (with standing BP) (Appendix 13)
    - **BP Medication Log**
    - **Participant Contact Information**
    - **Drug Dispensing Form** (if appropriate, ensure participants have 3 months of SPRINT medications; see Section 9)
    - **Close-out Lab Form** (chemistry and urine all participants; fasting and storage samples on subset; two different forms depending on whether participant is completing close-out visit A or close-out visit B) (Appendices 14 and 15)
    - **Annual History and Physical Exam Form** (includes 4 meter walk in subset; note that a physical exam is encouraged but not required, unless required by local IRB)
    - **My Health**
    - **Consent Form for continued contact**
    - **Obtain updated Release of Information**
    - **Provide Close-out Participant Kit**
- The following procedures will appear when the conditions listed are met and must be completed if they appear in the close-out visit packet:
    - **ECG** (if the participant has not reached their 48M visit; use visit code CLO)
    - **Morisky Adherence Scale** (if the participant has not reached their 48M visit)
    - **MIND Screening Battery** (if the 48M MIND testing has not been completed)
    - **MIND Extended Battery** (if the participant is in the subset or the participant triggers)
    - **Men's/Women's Health** (if the participant is in the subset and if the participant has not reached their 48M visit)
    - **Falls Efficacy** (if the participant is in the subset and has not reached their 48M visit)
    - **MRI Screening Form** (MRI participants only who have not already completed the MRI Screening Form)

- **MRI Appointment Confirmation Form** (MRI participants only)
  - **MRI CBC Lab form** (MRI and Mind the Kidney participants only) (Appendix 16)
  - **Provide participant MRI Retention Letter** (MRI participants only, located on the Participant Status Page)

- Applicable ancillary study forms and procedures should be completed following the appropriate ancillary study protocol. However, participant safety and main study procedures must be given the highest priority.
- The Cover Page will be printed for all close-out visits. This should always be reviewed to ensure that all required visit elements are collected or addressed appropriately.
- If the SPRINT study has been providing medications to the participant, the participant will be provided with a 3-month supply of SPRINT medication.
- All participants should be provided the Addendum to the Informed Consent to provide an opportunity for participants to consent for continued contact. (See Appendix 10 for model addendum.)
- Regardless of whether a current Release of Information is in place, all participants should sign an updated Release of Information. This will ensure the maximum length of time for obtaining any medical records requested by the study.
- All participants will receive a SPRINT Close-out Participant Kit (see Section 6).
- For MRI participant, provide the participant with the MRI Retention Letter. This letter is located on the Participant Status page (see Section 5.2).
- If the participant does not have a HCP and/or health insurance, the site should provide the participant with “Tips for Finding a Healthcare Provider or Insurance” document (Appendix 17) and assist as able to find a HCP and provide information about health insurance for the participant. Materials from the Guide to Assistance Programs (GAP) should be provided to participants as appropriate (see Section 8). These documents are located on the SPRINT web site under Documents >> Close-out Materials.

3.1.F. Following the close-out visit, participant close-out:

- If the site has permission to contact the HCP and the participant has requested that the Healthcare provider letter (Appendix 18) and SPRINT Study Medical Summary Report (Appendix 19) be mailed to the HCP instead of provided to the participant to be given to the HCP by the participant, the site should mail these documents.
- All data entry for the participants should be completed within 2 weeks after the visit per the protocol and must be completed NO later than **July 31, 2016** with the following exceptions:
  - The Close-out Encounter & Disposition Form and any Serious Adverse Event Forms must be data entered within 72 hours (3 days).
  - The MIND Screening and Extended Battery must be entered within 5 days.
  - The MRI Screening Form must be entered within 5 days.
- All MRI visit scans must completed by July 31, 2016. If the participant is in the MRI subset, ensure that the MRI visit has been scheduled and completed.
- Follow up about any My Health (PHQ9) Responses, as required.
- Once lab results are received, any alert values will be reported to the participant by the clinic.
- In the rare event a participant is seen after the close-out visit for safety reasons, a PRN visit Encounter and Disposition Form may be completed and entered for payment purposes. Please follow your network’s instructions about the need for a PRN Encounter and Disposition Form,. No SAE form or BP Med Log should be entered for the contact. The site should follow local requirements to determine whether the event should be reported to their IRB.

- Review the Participant Close-out Checklist and take appropriate steps to resolve outstanding information.
- All data entry queries for the participants will be completed NO LATER than August 31, 2016.
- All outcomes documentation will be completed within 60 days of notification of the event and NO LATER than August 31, 2016.
- The CC will notify the clinic PI of silent MI and/or cases of probable dementia. For cases received after clinic funding ends, it is the clinic PI's responsibility to notify the participant.

3.1.G. Clinic close-out:

- Use the Participant Close-out Checklist (Data Management >>Data Entry>> select a clinic from the list of available clinics >> Checklist) to monitor close-out progress on a participant-by-participant basis. When a participant row is all green checks (✓) or blue "N/A" then no further action is required for that participant.
- All remaining SPRINT drug inventory will be destroyed on site following local destruction policy (see Section 9).
- All unexpired lab collection supplies can be considered donated to the local clinic and can be destroyed or used at their discretion.
- All MIND testing supplies must be returned to the SPRINT MIND Coordinating Center (ATTN: Nancy Woolard). This includes:
    - All unused test forms: HVLT, MoCA worksheets, Digit Symbol Coding, Trails A&B
    - Digit Symbol Coding Scoring Template
    - Boston Naming Picture Book
    - Digital recorder
- See Section 14 for instructions for disposition of other study equipment.
- All study documents must be stored for 5 years after the end of the study, per NIH regulations, or for the length of time required by the local IRB, whichever is longer (see Section 15).

3.2 Close-out Encounter & Disposition Form

A Close-out Encounter & Disposition Form (see Appendix 12) will be collected and data entered for each participant. This form is the encounter documentation for the close-out visit and replaces the usual Encounter and Disposition form. The form will be printed with the forms for the close-out visit.

Unlike the previous Encounter & Disposition Form, the Close-out Encounter & Disposition Form has sections of the form that are questions directed towards the participant. **Clinics are advised to complete this portion of the form with the participant during the close-out visit.**

NOTE: If a participant is known to be deceased prior to the close-out visit, the Close-out Encounter & Disposition Form does not need to be completed. If the participant died since the last contact, but within the close-out visit window, then the Close-out Encounter & Disposition Form should be completed (see Section 4.4). Whenever the site is notified of a participant's death, an SAE form should be completed within 72 hours.

Section 4. Close-out Procedures by Status

The procedures for the close-out visit are dependent on the length of time from randomization to close-out, substudies the participant is enrolled in and the current status of each participant. If the site is unable to bring a participant in for a clinic visit, a phone or home visit prior to June 30 should be substituted.

For participants who have not been classified as LTF, withdrawn or deceased, follow procedures outlined in Section 3.1.E.

4.1 Close-out Procedures by Contact Type

4.1.A. Clinic or Home Visit

For participants that <u>come into the clinic</u> at the close-out visit, perform all applicable study procedures and complete applicable forms as indicated in Section 3.1. If a participant cannot come into the clinic, a home visit may be arranged. For home visits, follow procedures outlined in Section 3.1 and MOP chapter 3c for instructions on home visits. The suggested sequencing of the close-out visit follows. Note that some procedures will not be applicable for all participants.

**Recommended Sequence of Close-out Visits**
Review Cover Page
Greeting
Review and Update Informed Consent/Consent Form for continued contact/Update Medical Release/HIPAA
Blood Pressure (Close-out BP Management Form)
Blood draw, urine collection (Close-out Lab Form and MRI-CBC Lab Form, if needed)
ECG (ECG Form)
Review Events (Events Ascertainment Form)<br>As needed: SAE, Day Surgery, Dialysis
Participant directed questions on the Close-out Encounter and Disposition Form
Review contact information (Participant Contact Information Form)
Snack, rest, self-administered forms (My Health, Men's/Women's Health, Falls Self Efficacy)
MIND Testing (Screening and Extended, if Needed)
MRI Screening Form and schedule MRI, if needed
Height, weight, physical exam and 4-meter walk (if needed) (Annual History and Physical Exam Form)
Adherence scale (Morisky Medication Adherence Scale)
Record medications (Blood Pressure Medication Management Log)
Ensure participant has 3 months of SPRINT medications and dispense medication, as needed (Drug Dispensing Form)
Provide participant all materials from the Participant Kit (all participants) and MRI Retention Letter, if needed
Update and provide Medication Reconciliation Form to participant
Exit
Study Management Forms:<br>Encounter and Disposition Form (staff-directed questions)<br>As needed: Participant Status Log

4.1.B. Phone Visit

For participants <u>not able to come in for a clinic or home visit</u>, a phone visit is encouraged. Follow procedures outlined in MOP chapter 3c for instructions on phone visits. When completing a phone visit, complete the following (listed in order by priority):

- Events Ascertainment Form (Event Forms and SAE form as needed based on Event Ascertainment Form)
- Update Participant Contact Information

- Review and Update Informed Consent/Consent Form for continued contact/Update Medical Release/HIPAA (if allowed locally over the phone)
- Close-out Encounter & Disposition Form (participant-directed questions)
- BP Medication Log
- Annual History and Physical Exam Form (exclude questions 2, 3, 8-11)
- My Health Form
- HRQL questionnaires, if applicable (Women's Health, Men's Health, Falls Efficacy)
- If the study has been providing medications to the participant, dispense 3-month medications supply (see Section 9.2) and complete Drug Dispensing Form
- If the 48M MIND testing has not been completed, please inform the participant that someone from the SPRINT CC will be calling to administer the MIND Phone Battery. Document any feedback from this notification in the participant's notes section.
- Close-out Encounter & Disposition Form (staff-directed questions)
- Mail the Close-out Participant Kit and MRI Retention Letter (if needed) to the participant

4.1.C. Visits by Proxy

For a participant that cannot come for a close-out clinic visit AND is unable to make a home or phone visit, follow-up via proxy is encouraged. Follow procedures outlined in MOP chapter 3c for instructions on proxy phone visits. When completing a visit via proxy, please note that some forms cannot be collected. Complete the following (listed in order by priority):

- Events Ascertainment Form (Event Forms and SAE form as needed based on Event Ascertainment Form)
- Update Participant Contact Information
- Review and Update Informed Consent/Consent Form for continued contact/Update Medical Release/HIPAA (if allowed locally for these to be completed by proxy)
- If the study has been providing medications to the participant, dispense 3-month medications supply (see Section 9.2) and complete Drug Dispensing Form
- If the 48M MIND testing has not been completed, please inform the proxy that someone from the SPRINT CC will be calling him/her to administer one additional questionnaire. Document any feedback from this notification in the participant's notes section.
- Close-out Encounter & Disposition Form (staff-directed questions)
- Mail the Close-out Participant Kit and MRI Retention Letter (if needed) to the participant

4.1.D. No Visit

For a participant who has no study data collected at the close-out visit (unable to complete visit via clinic, home, phone or proxy), then:

- Complete Close-out Encounter & Disposition Form (staff-directed questions)
- Mail the Close-out Participant Kit to the participant
- Document in the participant notes section the methods attempted to contact the participant. Please also document that the contact attempts were unsuccessful.

4.1.E. PRN Visits

As sites become activated, all visits become close-out visits. The exception to this rule is for PRN visits. PRN visits can occur before or after close-out visits for safety reasons. For those participants whose close-out visits will occur late in the close-out visit window (April – June 2016), it may be necessary to have a PRN visit to ensure that the participant has an appropriate supply of blood pressure medications before the close-out visit. Once a participant has had his/her close-out visit and

been supplied with a three-month drug supply, additional study medications should not be supplied to the participant.

4.1.F. Late Data Collection and Split Visits

The only missed data from previous visits that should be collected at the close-out visits is the MIND data collection. For example, if a participant previously completed their 48M visit but did not complete the ECG, this should not be collected late at the close-out visit. If the MIND testing was not completed at the 48M visit, the forms will automatically print at the close-out visit and the MIND tests should be administered. For all other measures (ECG, fasting blood, etc.), late data collection should not be conducted at the close-out visit. Measures that should be collected at the close-out visit will display as part of the participant's close-out visit form set.

At times it may be necessary to split the close-out visit; in these cases, sites should follow directions as specified in Chapter 11 of the MOP (review the Event Ascertainment Form at both visits; update the Encounter & Disposition form to the second visit date). Examples of reasons for splitting visits include rescheduling a blood draw if a participant forgot to fast, or completing the MIND testing at a second visit if the participant cannot stay to complete this testing at the first visit. The current procedures specified in Chapter 11 of the MOP indicate that the second visit should occur within 14 days of the first visit. For close-out visits, while it is strongly recommended that visits occur within this time frame, it is not required.

4.2 Lost to Follow-Up Participants

Attempts should be made to find and contact participants that have been lost to follow-up before June 30, 2016 in order to schedule a visit.

If the participant <u>cannot be located after multiple attempts</u>:

- Document the type of contact and final attempt dates on the Participant Status Log.

If the participant <u>can be located</u>:

- If the participant agrees to a close-out visit, indicate the participant has returned to the study on the Participant Status Log using the procedures as described in the MOP Chapter 11.5.3. This includes completing Part C of the Participant Status Log.
- Request to schedule them for the close-out visit in the clinic.
    - If they agree to a clinic or home visit, complete the close-out visit per the procedures as stated in Section 4.1.A.
    - If they do not agree to a clinic or home visit, complete the close-out visit as a phone visit per the procedures for a phone visit as stated in Section 4.1.B.
    - If they do not agree to a phone visit, request a close-out contact with the proxy and complete the proxy visit per the procedures for a proxy visit as stated in Section 4.1.C.
    - If contact is made but the participant refuses all data collection at the close-out visit, document the contact attempt on the Participant Status Log. Be sure to note in the comments that contact was made but the participant would or could not provide any data.

See Appendices 5 and 6 for tips on finding lost to follow-up participants.

If the Participant Status Log has not been entered for a lost to follow-up participant prior to clinic activation for close-out, or if a participant not previously classified as lost to follow-up becomes lost to

follow-up during close-out, the Participant Status Log and the Close-out Encounter and Disposition Form (indicating a missed visit) must be completed in order to resolve the close-out visit.

4.3 Withdrawn Participants

If allowed by your local IRB, send the Permission to Contact You about Health Status letter (Appendix 9).

If the participant agrees to a phone visit or for the study to contact the proxy, indicate the participant has returned to the study on the Participant Status Log using the procedures as described in the MOP, Chapter 11.5.3. This includes completing Part C of the Participant Status Form.

If the participant agrees to a phone visit, complete the close-out visit per the procedures for phone visits as stated in Section 4.1.B.

If the participant agrees to allow the proxy to be contacted, complete the close-out visit per the procedures for proxy visits as stated in Section 4.1.C.

If the participant does not wish to be contacted or fails to respond, the site should not attempt to contact them. Document the date the letter was sent to the participant in in Part D, “Comments” on the Participant Status Log.

If the Participant Status Log has not been entered for a withdrawn participant prior to clinic activation for close-out, or if a not previously withdrawn participant withdraws consent during close-out, the Participant Status Log and the Close-out Encounter and Disposition Form (indicating a missed visit) must be completed in order to resolve the close-out visit.

4.4 Deceased Participants

If a participant is known to be deceased prior to the beginning of the participant’s close-out visit window (i.e., prior to the site becoming activated for close-out), the Close-out Encounter & Disposition Form does not need to be completed. Check to ensure that an SAE form has been completed.

If the participant died since the last contact, but within the participant’s close-out visit window, the Close-out Encounter & Disposition Form and a SAE Form must be completed. Deaths that occur during the close-out window will be assigned an outcome ID. Records collection and adjudication will follow the usual process for the outcome.

As stated in the SPRINT protocol, the SPRINT CC will obtain a Dementia Questionnaire (DQ) on all participants who have died more than 1 year after their last MIND testing. The central phone callers will administer the DQ to the person listed as the participant’s contact.

Any death of a participant (including withdrawn and lost to follow-up participants) who are identified as deceased through the Death Records Search or National Death Index (NDI), will be assigned an outcome ID. Records collection and adjudication will follow the usual process for the outcome.

After the close-out visit has been completed: SAE forms should be completed for deaths that are reported or discovered after the participant has completed close-out. For these deaths, records will not be requested. Site staff should, however, send a note to the Coordinating Center in SAE tracking noting that this death was discovered after the close-out visit had been completed. Knowledge of

these deaths will provide helpful information about participant status should there be an extension of the SPRINT study. In all cases, whenever the site is notified of a participant's death, an SAE form should be completed within 72 hours.

Section 5. MIND and MRI Visit Procedures

5.1 MIND Procedures

The 48 Month MIND cognitive testing must be completed on all participants. All sites should follow the guidelines described below for the cognitive testing.

The Close-out Visit Schedule (Data Management >>Data Entry>> Select a clinic from the list of available clinics >> Scheduling Aid) will identify participants who need to have 48M MIND testing completed at the close-out visit. For participants that have completed the 24M testing within the past 12 months, if possible, schedule their close-out visits toward the end of the close-out window in May/June 2016.

The MIND Screening and, if expected, MIND Extended batteries will print as part of the close-out visit forms packet. <u>Please administer all MIND forms that print in the packet.</u>

The following MIND scenarios may occur during the close-out visit period:

1. 48M testing has not been completed: the MIND Screening forms will print in the visit packet. The MIND Extended forms will print as well if the participant is in the 2800, MIND the Kidneys, or required adjudication at 24M and was not classified as Normal or Probable Dementia. <u>Administer all tests that print in the visit packet</u>.

2. 48M MIND Screening battery was completed at a previous visit and the participant triggered the Extended battery: the MIND Extended battery forms will print in the visit packet and should be administered during the close-out visit.

3. 48M MIND Screening battery is completed at the close-out visit and the participant triggers the Extended battery: Following data entry of the Screening battery, sites will be notified of this trigger will appear in three ways:
   a) A pop-up note will display when the Screening Battery is data entered.
   b) The "MIND EXTENDED NEEDED" column on the Scheduling Aid will turn red.
   c) The Closeout Visit Triggers (in the Closeout Reports folder) lists participants who have triggered at their Closeout Visit and the number of days since their Screening Battery.

   You should schedule a follow-up appointment to administer the Extended Battery within the next 30 days. The Extended Battery will now display under the close-out visit: select close-out visit, then select only the MIND Extended Battery.

Clinic staff have the option to data enter the MIND Screening forms during the close-out visit to determine if the participant will trigger. The staff at the clinic also has the option of completing the extended battery at the close-out visit if their clinical judgment or subject performance leads them to believe the subject will trigger.

Your communication with the participant is very important. At the end of the close-out visit, if the participant completed the Screening Battery, please let the participant know that you might be calling in the next few days to schedule just one more visit to complete some additional testing. This will

eliminate the surprise and help prepare the participant in the event that you need them to return to the clinic.
If an FAQ is needed, it must be completed within 2 weeks of the Screening Battery. Sites doing local FAQ administration can use the report Clinical Operations → Listing of LOCAL FAQs Needed. Also, upon data entry of the screening battery if an FAQ is needed you will see a pop-up. Some FAQs may be referred back to the site if the CC is unable to reach the proxy. These will appear on the task list (FAQs returned to local site). These will also need to be completed within 2 weeks of being referred back to the site. If a family member or friend accompanies the participant to the clinic and staff are able to complete the FAQ right then – please do so. It will save time and effort in the long run.

If the site exhausts all possibilities of completing the FAQ, print out an FAQ form from PRN and scan the barcode. The data entry screen contains a check box indicating the FAQ is unobtainable; this question is not on the actual form. Sites are encouraged to make multiple attempts at varying times before indicating a FAQ is unobtainable.

At the close-out visit, please review all MIND-related materials to assure that all cognitive testing has been completed and, if participating in the MRI substudy, the MRI has been scheduled.

The central callers will complete the close-out cognitive testing if the close-out visit is conducted via phone. Clinics should inform the participant that someone from the SPRINT CC will be calling to administer the MIND Phone Battery. Document any feedback from this notification in the participant notes section.

For participants that refuse all 48M MIND testing, the SPRINT CC will administer the DQ to the participant's contact.

The study will continue the same dementia adjudication process and notification as outlined in MOP Chapter 4.

5.2 MRI Procedures

The MRI Initial Contact Screening Form is available under PRN forms. This can be completed and data entered any time prior to or during the Closeout Visit. Sites are encouraged to screen and schedule MRIs as soon as possible in order to complete the process by July 31, 2016.

The Screening Form should be data entered as soon as possible, but no later than 5 days from completion. The personalized MRI Retention Letter is on the Participant Status Page. This letter should be printed and given to the participant at the closeout visit (if local IRB permission has been obtained). Once the MRI is scheduled, it is important for sites to phone the participant a day or two before the scheduled scan to remind him/her of the appointment. After the appointment, confirm that the scan was completed or, if needed, contact the participant to reschedule.

Section 6. Documents for Participants and the Close-out Participant Kit

The Close-out Participant Kit provided to the SPRINT participant will contain all information needed following the close-out visit. The Coordinating Center will supply the jar openers and the Certificates of Appreciation (see Appendix 20; to be personalized at the clinic) to the CCNs for distribution to the clinics. If the site needs more of these items, contact the CCN Coordinator. It is the site's responsibility to print the remaining components of the Close-out Participant Kit.

In order to alleviate last minute effort, it is recommended that the site compile all necessary documents prior to the participant's close-out visit. All generic materials (participant bulletin and a template for the Healthcare provider letter to be personalized by the site) are available on the SPRINT website under Documents >> Close-out Materials. The CC has provided one hard copy of the GAP binder to each SPRINT clinic and all materials are posted on the SPRINT website under Documents >> Close-out Materials >> Guide to Assistance Programs. The SPRINT Study Medical Summary Report is available on the participant's page in the Participant Information box.

The Close-out Participant Kit can be mailed to participants who do not come to the clinic for the close-out visit, although all efforts should be made to get all possible participants, except for those who withdrew consent or died, into the clinic for the close-out visit. Participant Kits are not provided to participants who are classified as LTF, withdrawn or deceased at the time of the close-out visit

The SPRINT Close-out Participant Kit will include:

1. **Generic Materials**
    a. **Participant Bulletin** (Appendix 21) – a bulletin which includes a note of appreciation from the study as well as information on reporting trial results, possible study extension, and frequently asked questions.
    b. **SPRINT Jar Opener –** a jar opener will be provided to all participants to assist participants in activities of daily living and express the study's appreciation of the participant's involvement in the SPRINT study.

2. **Personalized Materials**
    a. **Certificate of Appreciation** (Appendix 20) – a personalized certificate to indicate the appreciation of the Project Office, Steering Committee, and Clinical Site for the participant's contribution to SPRINT.
    b. **Healthcare Provider Letter** (Appendix 18) – a personalized letter for the site to mail to the participant's HCP or for the participant to take to their HCP regarding their participation in SPRINT.
    c. **SPRINT Study Medical Summary Report** (Appendix 19) – an abbreviated, personalized SPRINT medical history of the participant. Two copies of the SPRINT Study Medical Summary Report should be included; one will be provided to the participant and the other should be mailed to the HCP along with the Healthcare Provider Letter or provided to the participant for the participant to give to their HCP.
    d. **GAP Information** – patient information sheets on the various medications the participant has been prescribed with information on assistance programs for these medications and applications to the programs. Sites should determine which documents are relevant to the participant and make photocopies of documents from the GAP binder at the clinic or print these documents from the SPRINT website (available under Documents >> Close-out Materials >> Guide to Assistance Programs). See Section 8 for additional information.

6.1 Generic Materials

A participant bulletin and a SPRINT jar opener will be included for each participant in their Close-out Participant Kit. The participant bulletin will be printed by the SPRINT clinic. The participant bulletin is available in Appendix 21 and under Document >> Close-out Materials. The jar opener will be distributed to the clinics from the CCN. If the clinic needs more jar openers, contact the CCN.

6.2 Certificate of Appreciation

A certificate of appreciation (Appendix 20) will be included for each participant in their Close-out Participant Kit. The certificate will indicate the appreciation of the SPRINT Study Team. The CC will provide certificates signed by the Project Office and the SPRINT Steering Committee Chair and pens to personalize each certificate. The CC will distribute these materials to the clinics for personalization by the clinic. Spaces for the participant's name, the clinical site Principal Investigator and Coordinator's signatures are left blank, to be filled in at the site.

6.3 Healthcare Provider Letter

A letter to the participant's Healthcare Provider (Appendix 18) will be included for each participant in their Close-out Participant Kit. The letter will be personalized by the SPRINT clinic with health care provider contact information and participant name. Spaces for the clinical site Principal Investigator's signatures will be left blank, to be filled in at the site. Contact information for the clinic will be included on the letter. The template letter is available in Appendix 18 and under Documents >> Close-out Materials.

If a Healthcare Provider Wants More Information on SPRINT or Participant's Care

The letter to the Healthcare Provider given to the SPRINT participant in their SPRINT Participant Kit should have information as to who to contact at a clinical site for further information on a participant. The site should ensure, through proper medical release documentation, that the Healthcare Provider has received permission from the participant before giving any requested information to the Healthcare Provider. If the HCP requests additional information about the SPRINT study, sites can direct him/her to the SPRINT study website (https://www.sprinttrial.org) where additional information about the SPRINT trial is available.

6.4 SPRINT Study Medical Summary Report

The SPRINT Study Medical Summary Report (Appendix 19) will be given to the participant at the close-out visit as part of the Close-out Participant Kit. Two copies will be provided; one for the participant's records and one to give to their healthcare provider. If the participant has provided consent, the copy for the HCP can be mailed by the SPRINT clinic to the participant's HCP.

Note that this report will not include the last lab values, last BP and heart rate measurements and modifications to the medications (should be rare).

The SPRINT Study Medical Summary Report is available on the Participant Status Page. See Appendix 19 for an example of this report. This report is a pdf file, showing the participant name, SPRINT treatment arm, graphs of systolic and diastolic blood pressure readings during the trial, a graph of heart rate readings during the trial, lab measurements over the course of the study, adverse events reported during the study, and a list of antihypertensive medications at the time of the close-out visit.

6.5 Guide to Assistance Programs

The CC has provided one hard copy of the GAP binder to each SPRINT clinic and all materials are posted on the SPRINT website under Documents >> Close-out Materials >> Guide to Assistance Programs. Clinics should photocopy or print materials specific to the participant and distribute these to

the participant along with other parts of the Participant Close-out Kit. See Section 8 for additional information about the GAP materials.

6.6 Notification of Study Results to Participants

Following the close-out visit, the Coordinating Center will contact all participants to convey the study results.

Section 7. Helping Participants find a Healthcare Provider or Insurance

SPRINT clinical sites should assist those participants who do not have insurance and/or a regular healthcare provider for hypertension related health needs to ensure continued care after SPRINT has ended. (NOTE: A SPRINT clinic may accept a participant as a patient if so desired.)

Sites should reiterate that having a healthcare provider for hypertension related care is important for maintaining continued good health and can offer several suggestions on how to transfer care from the site to a healthcare provider.

During the transition visits, the site should discuss with the participant the end of SPRINT and determine whether the participant has a health care provider and/or health insurance. Once the site has received IRB approval for these documents, the site should provide all participants with the Tips for Finding a Healthcare Provider or Insurance (Appendix 17) and materials from the GAP specific to the participant's needs. See Section 8 for additional information about GAP materials.

Section 8. Guide to Assistance Programs (GAP) Information

In order to provide participants with information on obtaining assistance with medications that they may not be able to afford, the CC will provide each SPRINT clinic with one copy of the Guide to Assistance Programs (GAP) binder. The materials in the GAP are also available on the SPRINT website under Documents >> Close-out Materials >> Guide to Assistance Programs (GAP). In addition to providing information about medications, the GAP provides summary descriptions for locating an insurance provider. This includes information about Medicare, the Affordable Care Act and VA.

The GAP includes patient information sheets on the various medications the participant has been prescribed along with information on assistance programs for these medications and applications to the programs. The medication sheets are alphabetized by generic name. On the reverse side of each drug sheet there is information stating if and/or where the drug is available through a discount or assistance program. If an assistance program is available, the clinical site can then go to the section for applications and find the appropriate application form.

Appropriate documents should be photocopied from the GAP binder or can be printed from the SPRINT website and given to the participant.

Section 9. Medications

At the close-out visit, SPRINT participants who are currently taking SPRINT medications will be dispensed a 3-month supply of medications from the SPRINT formulary. If the participant is not currently taking any SPRINT medications and the time of the close-out visit, he/she will not receive any medications. Once a participant has had his/her close-out visit and been supplied with a three-month drug supply, additional study medications should not be supplied to the participant.

Medications should not be changed at the close-out visit unless there are safety concerns. An investigator may use his/her clinical judgment if it is believed in the best interest of the participant to alter therapy, e.g., for patient safety.

At the close-out visit, participants should be provided a 90-day supply of SPRINT medications, with the intent that the participant will begin getting study medications prescribed through his/her health care provider within the 90-day period. Make certain that participants are aware that this is the last medication supply they will get from SPRINT, and they need to make other arrangements for their medical care. In order to avoid any overdosing of blood pressure medications, remind the participant to take all the provided SPRINT medication, along with the SPRINT Study Medical Summary Report, to their next visit with their health care provider.

Contact information and instructions to call the SPRINT clinic in the case of a study medication related problem will be provided to every participant per standard local procedures. These instructions should include first calling 911 in the event of a medical emergency. It should be explained to participants that once they are under the care of a healthcare provider they no longer need to contact their former SPRINT clinic.

Drug information sheets from the GAP will be distributed for all medications, regardless of whether or not the handout has been given out previously. Although the majority of SPRINT medications are relatively inexpensive, sites may use the tools provided in the GAP and other available materials to discuss with participants reasonable inexpensive alternatives to the SPRINT medications as well as any available programs for obtaining medications (e.g., Edarbi, Edarbyclor) at a discounted rate. The drug information sheets in the GAP list some examples of programs for each drug that may assist participants in obtaining the medication.

PLEASE NOTE: The local PI retains responsibility for his/her participants while they are taking SPRINT study medications. Once the participant is seen by a healthcare provider, the responsibility of care will be transferred to the new healthcare provider.

9.1 Manual Orders for Medications

All par levels will be “zeroed” on Friday, January 8, 2016. Beginning the week of January 11, 2016, clinics must order drugs manually by emailing the DDC at ABQCSPSPRINTORDER@va.gov. The Drug Order Form should be completed and emailed to the DDC at ABQCSPSPRINTORDER@va.gov (see Appendix 3 for the form). Orders sent to the DDC and received by the end of the day Wednesday will be processed and shipped out to sites on the following Monday via overnight delivery. The DDC will still ship medications to clinics weekly and on an expedited basis if there are extenuating circumstances.

Sites should review the schedule of upcoming participants and plan appropriately to ensure adequate supply to provide a three-month supply to all participants at their close-out visit. Overnight shipments are available, but should not be necessary with proper inventory management.

In order to conserve resources, if a clinic has an excess amount of medication that will not be used for SPRINT participants, sites are encouraged to contact their CCN coordinator and find another clinic that needs these drugs. The Drug Transfer Form (Clinic to Clinic) should be completed and sent to the DDC (see Appendix 22 for the form).

9.2 Mailing Medications to Participants after a Phone Visit

Medications dispensed at the close-out visit through the mail should only occur when a participant who was receiving study medications prior to the close-out visit (regardless of whether the participant has transitioned care to a HCP) completes the close-out visit by phone or proxy. Mailing medications to participants is permitted ONLY under these circumstances. In some states, medications cannot be mailed to a participant. It is important that staff is familiar with state and local regulations. If mailing medications is not permitted, the site should use their judgment to determine the best way to get medications to the participant

****NOTE:*** No medications should be mailed to any participant for whom you cannot complete a close-out visit in the clinic, home or by phone or proxy.

9.3 Destroying Study Drugs

No drugs should be shipped back to the Drug Distribution Center. All unused drugs are to be destroyed using the local drug destruction procedures. Document the drug destruction electronically on the SPRINT website using the Drug Destruction System under Data Management (Pharmacy section). This link allows sites to scan drugs that are destroyed, and automatically decreases inventory levels. Sites should show no medications in their inventory at the end of this process.

PLEASE NOTE:

- All drugs that are being destroyed locally should be scanned or manually entered in the destruction table via the SPRINT website.

Section 10. Laboratory Kits and Specimen Collection

10.1 Laboratory Kits

In order to efficiently manage supplies, the central laboratory recommends that clinics order enough supplies for a 2-month period and then place additional orders.

There are two types of close-out lab visits, either Close-out A (CLA) or Close-out B (CLB). Participants who have already had their 48M visit prior to their close-out visit will have a Close-out A lab visit. Participants who have not had their 48M visit prior to their close-out visit will have a Close-out B visit.

- Close-out A: The Close-out A lab visit will include the same lab components as a current “6, 36, 60 month” visit. In order to conserve resources, sites should use up any “6, 36, 60 month” kits that are available at the site for the Close-out A lab visits and then order additional Close-out A kits as necessary.
- Close-out B: The Close-out B lab visit will include the same lab components as a current “12, 24, 28 month” visit. In order to conserve resources, sites should use up any “12, 24, 48 month” kits that are available at the site for the Close-out B lab visits and then order additional Close-out B kits as necessary.
- It is acceptable to use the “6, 36, 60 month” kits for the Close-out A visit and the “12, 24, 48 month” kits for the Close-out B visit even if there is not a specific “Close-out A” or “Close-out B” label on the kit.
- When placing orders for lab kits, remember to allow for 7-10 business days for receipt of your order.

10.2 MRI-CBC Study

There is a cohort of approximately 700 participants who will receive a complete blood count (CBC) blood collection sample as part of the close-out visit. This includes participants in the MRI substudy and Mind the Kidney (MTK) ancillary study. The Central Laboratory has received a list of the specific sites that will be involved in the ancillary study and has sent CBC blood collection tubes directly to these sites. There is a separate specimen collection form (Appendix 16) for this collection but the CBC blood sample should be shipped with the close-out visit samples.

The 4-ml EDTA tube is to be collected at the same time as the close-out lab visit; either close-out A or close-out B, depending on the participant. Utilize the SPRINT Close-out A or B lab kit and ***remove the Lab ID stickers and discard them. Instead, use a new lab ID label set that has been sent with the 4 ml EDTA tubes.*** This is to ensure that the clinic has enough labels to label all of the collection tubes, transport tubes, and specimen collection forms. The lab ID label should be placed on both the Close-out A/Close-out B Lab form and the MRI-CBC Lab Form. The same shipping instructions should be used and are outlined in the SPRINT Laboratory Biospecimen Collection and Processing Manual. The extra 4-ml tube and MRI-CBC Collection Form is shipped with the SPRINT close-out specimens and collection form.

Section 11. Outcomes and Serious Adverse Events

11.1 Outcome Collection

The close-out visit will be the last opportunity to ascertain any SPRINT study outcomes as defined in the SPRINT protocol. As such, clinical sites should make every effort to contact participants and perform outcomes assessment, especially for participants who are lost to follow-up.

11.2 Serious Adverse Events

For safety events that occur in association with the SPRINT close-out visit and are reportable according to the SPRINT safety criteria, an SAE form should be completed within 72 hours of knowledge of the event. Examples include: ER visit/admission for symptomatic hypotension, abnormal SPRINT lab findings, ECG findings, or any other emergent health concerns associated with the close-out visit or that occur **while** the participant is in the clinic for their close-out visit. Staff should follow the same process for determining whether an event meets reporting criteria for SPRINT. Sites should follow local and institutional procedures for reporting these events.

After a participant's close-out visit, should the participant call and report an adverse event, an SAE form should not be entered into the SPRINT website. Serious adverse events should be reported following local and institutional procedures. If a participant calls and is in need of medical assistance, related to blood pressure medications or management, he/she should be referred to their HCP for management. However, if the participant does not yet have a HCP, then site should see them per good clinical practice, up to 90 days post close-out.

In the rare event a participant is seen after the close-out visit for safety reasons, a PRN Encounter and Disposition form may be completed and entered for payment purposes. Please follow your network's instructions about the need for a PRN Encounter and Disposition Form. No SAE form or BP Med Log should be entered for the contact. The site should follow local requirements to determine whether the event should be reported to the local IRB.

11.3 Reporting Deaths

In all cases, whenever the site is notified of a participant's death, an SAE form should be completed within 72 hours. Depending on where the participant is in their visit schedule, please use the below directions for additional forms required:

Prior to the close-out visit: If a participant is known to be deceased prior to the site becoming activated for close-out, the Close-out Encounter & Disposition Form does not need to be completed. Check to ensure that an SAE form has been completed. Deaths that occur prior to the participant's close-out visit will be assigned an outcome ID. Records collection and adjudication will follow the usual process for the outcome.

During the close-out visit window: If the participant died since the last contact, but within the participant's close-out visit window, the Close-out Encounter & Disposition Form and an SAE Form must be completed. Deaths that occur during the close-out window will be assigned an outcome ID. Records collection and adjudication will follow the usual process for the outcome.

After the close-out visit has been completed: SAE forms should be completed for deaths that are reported or discovered after the participant has completed close-out. For these deaths, records will not be requested. Site staff should, however, send a note to the Coordinating Center in SAE tracking noting that this death was discovered after the close-out visit had been completed. Knowledge of these deaths will provide helpful information about participant status should there be an extension of the SPRINT study.

11.4 Medical Records

Beginning July 1, 2016, clinical sites will have 60 days to obtain medical records for study events. At the close-out visit, sites should request that all participants sign an updated medical release form.

Section 12. Close-out Reports and Tools

12.1 Reports to Review Prior to Close-out

Prior to beginning close-out visits, all data entry must be up to date. In order to ensure that all data entry is up to date, review:

- List of Unresolved Data Entry (Reports >> Clinical Operations)
- List of Unresolved Visits (Reports >> Clinical Operations)
- Participants with E&D for Current Visit Printed by not Entered (Reports >> Close-out Reports)
- In or past 48M window with missing MIND data (Reports >> Close-out Reports)

The CCN and CC will review these reports prior to activating a clinic to being close-out visits. In order for the data entry system to work smoothly and for the proper forms to display, there must be no open visit windows without an Encounter and Disposition Form data entered and all MIND forms must be data entered.

12.2 Death Records Search Report

This report is a list of participants who have been identified as withdrawn or lost to follow-up on a Participant Status form, and participants who missed their last visit. Record searches should be

performed for these participants to determine whether any are deceased. In addition, every effort should be made by the clinical site to locate LTF participants and those that missed their last visit. Tips for locating LTF participants are provided in Appendices 5 and 6. This report is located on the SPRINT Website under Reports >> Close-out. See Section 3.1.A for additional information.

12.3 Close-out Visit Scheduling Aid

The scheduling aid provides a listing of participants with their target visit dates, date of last MIND data collection, identifies participants as requiring close-out visit A or close-out visit B, and identifies whether the MIND testing is required. Once the Close-out Encounter and Disposition Form has been entered, the date of the close-out visit will be listed to reflect the date on the Close-out Encounter and Disposition Form. This does not mean that all close-out visit components have been resolved. This aid is integrated into the participant pick-list for each site as a new tab (Data Management >> Data Entry >> select a clinic >> Scheduling Aid.)

12.4 Participant Close-out Checklist

This report is a compilation of key information by participant. Columns include: close-out visit resolved, extension consent resolved, data queries resolved, data entry resolved, expected ECGs resolved, expected MIND Screening resolved, expected MIND Extended resolved, MIND MRI resolved, MIND FAQ resolved, outcome events closed and SAE events closed. This report should be used to address key concerns regarding missing data. A green check indicates that the item has been resolved; a red 'x' indicates that an action is required by the clinic, blue 'N/A' indicates this item is not applicable for the participant. Instructions for how to resolve each item are available on the website.

Prior to clinic close-out the Participant Close-out Checklist will be reviewed to determine if there is additional action required for any SPRINT participant. Note: a written response to the CCN for expected but not done will not be required for this new report.

12.5 Monitoring and Aggregate Reports

Additional reports will be made available to the subcommittees, CCNs, CC and Project Office in order to monitor the close-out process. These reports are available under Reports >> Close-out.

Reports will include the Close-out Visit Completeness reports overall, by CCN, and by site. The Closeout Visit Completeness – by Site report summarizes the progress of close-out visit compliance and obtaining the addendum informed consent from participants at close-out. The Close-out Checklist Summary – by Site report provides a summary of the items on the close-out checklist that were expected and resolved. The Close-out Visit: List of Partial Visits provides a list of participants for whom the clinic indicated on the Close-out Encounter and Disposition Form that the visit was not completed as specified. Measures that are identified as missed reflect the responses from Question 10 on the Close-out Encounter and Disposition Form.

12.6 Cleaning Data

Data cleaning is an essential part of every clinical trial. Trial results are only as good as the information collected during the study. Therefore, extensive efforts will be made to ensure data are as accurate as possible.

Keep participant files handy for any future data cleanup that may be necessary as a result of the data analysis process.

Section 13. IRB Information

At the time of submitting Protocol 5.0 to the local IRBs, the sites should also submit all close-out materials that will be given or mailed to participants. As with previous protocol amendments, sites should submit a copy of the approval letter and a copy of the approved consent form to the Coordinating Center via the SPRINT website (Site Admin >> IRB Tracking >> IRB Uploads). The IRB approval letter is uploaded under Site Admin >> IRB Uploads >> REQUIRED Close-out Protocol Amendment Submissions. The Addendum to Consent Form (permission to contact participants about future research) should be uploaded under Site Admin >> IRB Uploads >> Consent Forms >> Addendum to Consent form (Close-out).

Until notified by the Coordinating Center, all sites should continue to file annual/continuing reviews at their regular schedule. If you have questions at any time, please contact your CCN Coordinator or the CC.

Section 14. Equipment Information

Due to the length of the SPRINT Study, most of the purchased equipment (Omron BP machines, ECG machines, desktop computers, laptops, etc.) will be out of date. The CCNs and the CC will contact the clinic to determine the condition of the equipment and determine whether the equipment needs to be returned or can be disposed or donated to the clinic. For equipment purchased with study funds, if they are disposed or donated, the CC or CCN will send the NHLBI Contracting Officer a letter indicating the intent to dispose or donate the equipment, along with any additional information requested by the Contracting Officer.

Section 15. Data and Record Storage

<u>Electronic Data</u>
Institutional procedures for storing and removing electronic participant data from computers should be followed at each local site. Contact the local IT department or other computer support group for information on the correct destruction of electronic data and computer cleaning.

<u>Non-Electronic Source Documentation</u>
Hard copies of source documents should be kept for at least 5 years, or longer if required by your local, state, or institutional regulations/policies.

At a minimum, each locally approved consent form should be reviewed to verify the length of time stated. If there is a discrepancy between current policy and the time stated in the consent form, the local IRB should be consulted for a determination. At the end of the required storage time, documents should be destroyed using local policies governing secure document destruction.

Long-term, warehouse-type storage is permissible; however please do not put records into off-site storage until after clearance has been received by the CC. Electronic storage (e.g., scanning onto DVDs or other media) is permissible; however appropriate backup measures and other steps should be taken to ensure data is not lost. Records should not be placed into long-term storage—electronic or otherwise—until after all data has been cleaned and the database has been locked. The CC will notify the SPRINT clinics when this has occurred.

Additionally, local IRBs may have policies about long-term storage. Because they have the right to audit studies under their jurisdiction, it is recommended that all documentation be kept within easy access until the local notification of the close-out procedures is approved or acknowledged by the IRB. For specific questions, contact the local IRB office.

List of Appendices

Appendix 1 – Definitions

Appendix 2 – Measures and Frequency

Appendix 3 – Drug Order Form (Manual Orders)

Appendix 4 – Lab Supply Reorder Form

Appendix 5 – Computer Search Tips

Appendix 6 – Internet Resources for Finding Lost to Follow-up Participants

Appendix 7 – Close-out Reminder Letter for Lost to Follow-up Participants (Participant)

Appendix 8 – Close-out Reminder Letter for Lost to Follow-up Participants (Proxy)

Appendix 9 – Permission to Contact You about Health Status

Appendix 10 – Addendum to Consent Form

Appendix 11 –Close-out Appointment Reminder Letter

Appendix 12 – Close-out Encounter and Disposition Form

Appendix 13 – Close-out Blood Pressure Management Form

Appendix 14 – Close-out Visit A Lab Form

Appendix 15 – Close-out Visit B Lab Form

Appendix 16 – MRI CBC Lab Form

Appendix 17 – Tips for Finding a Healthcare Provider or Insurance

Appendix 18 – Letter to Healthcare Provider

Appendix 19 – Study Medical Summary

Appendix 20 – Certificate of Appreciation

Appendix 21 – Participant Bulletin

Appendix 22 – Drug Transfer Form (Clinic to Clinic)

Note that Spanish versions of participant materials are available on the SPRINT study website under Documents >> Close-out Materials

APPENDIX 1 – Definitions

CC: The Coordinating Center at Wake Forest University School of Medicine.

Close-out Visit: The final SPRINT study visit for a participant. Participants that trigger the MIND Screening Battery may need to return to the clinic for additional MIND testing as part of their close-out Visit.

GAP: “Guide to Assistance Programs”, a tool for the sites to utilize when addressing participant needs for medication.

Healthcare Provider (HCP): The doctor, physician assistant, nurse practitioner or other healthcare provider that will be responsible for a participant's health care after SPRINT.

Incomplete Visit: A visit where partial or incomplete information is collected from a participant.

Lost-to-Follow-up (LTF) Participant: A SPRINT participant is considered to be lost to follow-up if he/she has missed more than two consecutive follow-up visits, cannot be contacted by any ordinary means (e.g., home phone, cell phone, mail, email, fax, etc.), **and** alternative contacts do not know where the participant is or cannot be contacted themselves. A participant who steadfastly avoids contact is also considered to be lost to follow-up.

Missed Visit: A visit where no data collection elements are collected.

SAE: Serious Adverse Event.

Withdrawn (Refused): A SPRINT participant is considered to be withdrawn if he/she has withdrawn consent to participate in the study and refuses further contact for any reason.

APPENDIX 2 – Measures and Frequency

	Screening /RZ	1 mo	2 mo	3 mo	6 mo	9 mo	1 yr	Q 3 mo	Q 6 mo	2 yr	3 yr	4 yr	Close-out A*	Close-out B**
Blood collection														
Chemistry profile		X		X	X				X		X		X	
Fasting Chemistry profile	X						X			X		X		X
Fasting glucose	X									X		X		X
Fasting lipid profile	X						X			X		X		X
Fasting serum and plasma storage	X						X			X		X		X
Genomic material	X													
Complete Blood Count (CBC)***													X	X
Urine collection														
Albumin, creatinine	X				X		X			X	X	X	X	X
Fasting urine storage	X						X			X		X		X
Physical measures														
Seated blood pressure, pulse,	X	X	X	X	X	X	X	X		X	X	X	X	X
Standing blood pressure	X	X			X		X			X	X	X	X	X
Weight	X						X			X	X	X	X	X
Height	X													
ECG	X									X		X		X
Physical examination	X						X			X	X	X	As required locally	As required locally
4 meter walk (≥ 75 ONLY)	X						X			X	X	X	X	X
Questionnaires														
Medical history	X													
Sociodemographics	X													
Alcohol use	X													
Smoking	X						X			X	X	X	X	X
Concomitant medications	X						X			X	X	X	X	X
Adherence & Adverse Events		X	X	X	X	X	X	X		X	X	X	X	X
Outcomes Ascertainment				X	X	X	X	X		X	X	X	X	X
**Health related quality of life**														
EQ-5D	X						X			X	X	X	X	X
Veterans Rand 12	X						X			X	X	X	X	X
PHQ-9 Depression	X						X			X	X	X	X	X
Patient satisfaction/Morisky	X						X					X		X
**Health related quality of life**														
Falls Efficacy (FESI-I)	X				X		X			X	X	X		X
Sexual Function (FSFI/IEFF)	X				X		X			X	X	X		X

MIND Questionnaires/Tests	Screening or RZ	2 yr	4 yr	Close-out A*	Close-out B**
**Dementia Screening**					
MoCA	X	X	X		X
Digits Symbol Coding Test	X	X	X		X
Logical Memory Test<br>Story A	X	X	X		X
**Cognitive Battery (subset)**					
Hopkins Verbal Learning Test	X	X	X		X
Trail Making Tests A and B	X	X	X		X
Digit Span	X	X	X		X
Boston Naming Test	X	X	X		X
Modified Rey-Osterrieth Figure	X	X	X		X
Verbal Fluency Animals	X	X	X		X

APPENDIX 3 – Drug Order Form (Manual Orders)

Please e-mail completed forms to **ABQ CSPSPRINTORDER@va.gov**

Contact:					Site No.
**Class**	**Drug Code**	**Drug**	**Strength**	**Restricted Formulary Item**	**Bottle Quantity (DDC will round to nearest box size)**
**Diuretic**					
	1J	Chlorthalidone	25 mg		
	1T	Furosemide	20 mg		
	1U	Furosemide	40 mg		
	1V	Furosemide	80 mg		
	2R	Spironolactone	25 mg		
	3S	Spironolactone	50 mg		
	3B	Amiloride	5mg		
	3D	Triamterene/HCTZ	75/50mg		
	1D	Amiloride/HCTZ	5/50 mg	x	
	3H	HCTZ	12.5mg	x	
	3J	HCTZ	25mg	x	
**Calcium Channel Blockers**					
	1K	Diltiazem ER	120 mg		
	1L	Diltiazem ER	180 mg		
	1M	Diltiazem ER	240 mg		
	1N	Diltiazem ER	300 mg		
	1A	Amlodipine	2.5 mg		
	1B	Amlodipine	5 mg		
	1C	Amlodipine	10 mg		
**Beta Blockers**					
	2J	Metoprolol Tartrate (short acting)	25 mg		
	2K	Metoprolol Tartrate (short acting)	50 mg		
	2L	Metoprolol Tartrate (short acting)	100 mg		
	2X	Metoprolol XL (long acting)	25 mg	x	
	2Y	Metoprolol XL (long acting)	50 mg	x	
	2Z	Metoprolol XL (long acting)	100 mg	x	
	3A	Metoprolol XL (long acting)	200 mg	x	
	1E	Atenolol	25 mg		
	1F	Atenolol	50 mg		
	1G	Atenolol	100 mg		
	3R	Atenolol/Chlorthalidone	100/25 mg		
	1H	Atenolol/Chlorthalidone	50/25 mg		
**Vasodilators**					
	2B	Hydralazine	25mg		
	2C	Hydralazine	50mg		
	2D	Hydralazine	100mg		
	2M	Minoxidil	2.5mg		
	2N	Minoxidil	10mg		
**Alpha 2 Agonist**					
	1W	Clonidine Patch - 1 Box of 4 Patches	0.1mg/24 hr	x	
	1X	Clonidine Patch - 1 Box of 4 Patches	0.2mg/24 hr	x	
	1Y	Clonidine Patch - 1 Box of 4 Patches	0.3mg/24 hr	x	
	1Z	Guanfacine	1 mg		
	2A	Guanfacine	2 mg		
**Alpha Blockers**					
	1P	Doxazosin	1 mg		
	1Q	Doxazosin	2 mg		
	1R	Doxazosin	4 mg		
	1S	Doxazosin	8 mg		
**ACEi**					
	2V	Lisinopril/HCTZ	20/12.5 mg	x	
	2W	Lisinopril/HCTZ	20/25 mg	x	
	3C	Linsinopril	5mg		
	2F	Linsinopril	10 mg		
	2G	Linsinopril	20 mg		
	2H	Linsinopril	40 mg		
**Angiotensin Receptor Blockers - Either Valsartan OR Losartan**					
	3G	Losartan	25mg		
	3E	Losartan	50mg		
	3F	Losartan	100mg		
	2S	Valsartan	80 mg	x	
	2T	Valsartan	160 mg	x	
	2U	Valsartan	320 mg	x	
	3K	Azilsartan Medoximil	40 mg		
	3L	Azilsartan Medoximil	80 mg		
	3M	Azilsartan Medoximil/Chlorthalidone	40/12.5 mg		
	3N	Azilsartan Medoximil/Chlorthalidone	40/25mg		
**Potassium supplements**					
	3P	Potassium Chloride Solution 10%	20MEQ/15ML		
	2E	KCL	20MEQ		

APPENDIX 4 – Lab Supply Reorder Form

Appendix 6. Supply Reorder Form

SPRINT
Systolic Blood Pressure Intervention Trial

Supply Reorder Form

Please complete this form and **FAX** it to the SPRINT Central Laboratory at **612-625-4142 or 612-625-4831 (alternate fax number).** *Please allow **7-10** working days for receipt of your supply order Thanks.*
Central Lab phone number: 612-625-5040

Clinical Field Center ID Number ________ Date: _____/ _____/ _____

Shipping Address: ______________________________

____

____

____

Phone Number: ______________________

Kits *Please indicate desired quantity for each 'Visit type'. To minimize expiration of collection tubes supplied in the kits, do not order more kits than you expect to use within a few months.*

___ 1,3,18,30,42,54,PRN Month Visit Kit

___ 6,36,60 Month Visit Kit (*Close Out A kit)

___ 12,24,48 Month Visit Kit (*Close Out B kit)

Replacement blood collection tubes/Urine Cups *Please monitor expiration dates on blood collection tubes supplied in the kits. NOTE: collection tubes expire on the last day of the month printed on the label*

___ 7.5-mL SST/gel (red top) **____ 8-mL EDTA/gel (purple top)** **___10-mL EDTA (purple top)**

___ Sterile Urine Cups **___ Urine Transport Tubes (blue cap)** **___ Plasma Transport Tubes (purple cap)**

Additional supplies:

_____ FedEx Billable Stamps, pre-printed

_____ FedEx International Air Waybills, pre-printed (For Puerto Rico sites)

_____ Shipping Boxes with gel packs

_____ FedEx UN 3373 Pak Mailing Bag

APPENDIX 5 – Computer Search Tips

If you are not having success in finding a lost participant, try some of the following tips:

> Search by possible alternate names or by the spouse's or other contact's name.

> Change dates around (e.g. instead of searching for 10/05/1984 try switching the month and day: 05/10/1984)

> Use all other possible spellings and punctuation of the name and perhaps some that are not so likely (e.g., when searching for a name like O'Hare, try Ohare; when searching for a name like Susanne, try Suzanne, Susane or Sue; when searching for someone named Robert, also search Bob or Rob).

> If you are looking for someone using a first name but don't find what you are looking for, try using just the initial. There are also some instances of what appear to be middle initials included in the last name field, so your may want to try this strategy in the last name field as well.

> Switch last name and first name around. Also try searching for a middle name as a first name.

> Even if you know a piece of information, try omitting it (e.g., if you know the first and last name and SSN, try searching only by SSN or by last name).

> With Hispanic names often there are several name combinations that could be possible (e.g., Luis Raul Huitron Azcuaga may be listed as Raul Huitron, Raul Azcuaga, Luis Azcuaga, or Luis Huitron).

> Try using the last address as a reverse check for neighbors who may know where the person is.

APPENDIX 6 – Internet Resources for Finding Lost to Follow-up Participants

Almost anyone can be located using the Internet, which essentially is a huge set of databases with consumer (e.g., credit card companies) and public (government) records. The process for finding lost individuals is not difficult but can be time-consuming. In searching for someone, four important pieces of information are needed: **name, SSN, date of birth, and last known address.** You should have all of this information on the Participant Contact Information Form. Companies may require a fee to access their databases; however, many are free.

WHITE PAGES: Phone numbers and addresses of participants, family member, or neighbors
http://www.555-1212.com
http://www.whitepages.com
http://www.smartpages.com
http://www.whowhere.lycos.com

VITAL RECORDS: Checking for possible deaths and requesting death certificates
http://ancestry.com/search/rectype/vital/ssdi/main.htm
http://www.vitalchek.com
http://www.archives.ca/01/01_e.html

NEWSPAPERS: Obituaries
http://www.obits.com
http://www.cyndislist.com/obits.htm
http://www.obitlinkspage.com/obit/canada.htm

PUBLIC RECORDS: Resources (May be Fees and Membership required)
http://www.vericheckinfo.com
http://www.publicdata.com
http://www.brbpub.com/
http://www.wdia.com/mem/

SEARCH SERVICES: (Fee Based)
http://www.ustrace.com
http://www.peoplelocate.com

GOVERNMENT GUIDE: Information
http://www.pueblo.gsa.gov/call/
http://www.governmentguide.com/

HOMELESS MISSING PERSONS:
http://www.nationalhomeless.org/direct1.html

GENEOLOGY SERVICES: (Fee Based)
http://ancestry.com
http://genealogy.com
http://www.usgenweb.com
http://www.rootsweb.com
http://itsnet.com

SOCIAL MEDIA:
http://www.facebook.com
https://www.twitter.com
http://www.linkedin.com
https://plus.google.com

APPENDIX 7 – Close-out Reminder Letter for Lost to Follow-up Participants (Participant)

<<Insert Date Here>>

Dear <<Insert Participant Name>>,

We would like the chance to talk to you about your participation in the Systolic Blood Pressure Intervention Trial (SPRINT). Please recall that this is an important study about treating high blood pressure.

Even if it has been some time since your last visit and you are no longer taking SPRINT medications, your participation is still valuable. Information about your health is still very important to us.

Although the main portion of the study has ended, we would like to see you for a visit to update our records on your blood pressure management and health status since we saw you last.

Please contact us as soon as you can for an appointment at <<insert clinic phone number>>. If you call and we are unavailable, please leave your name and number so that your call can be returned. If you are unable to make it into the office for a clinic visit, we can discuss other options, such as a phone visit.

Thank you for your participation in this important study. We look forward to seeing you again soon.

Sincerely,

<<Insert Clinic PI Name>>

<<Insert Clinic Contact Information>>

APPENDIX 8 – Close-out Reminder Letter for Lost to Follow-up Participants (Proxy)

SPRINT
Systolic Blood Pressure Intervention Trial

<<Insert Date Here>>

Dear <<Insert Proxy Name>>,

Mr./Ms.<<insert participant name>> has given us permission to contact you regarding the Systolic Blood Pressure Intervention Trial (SPRINT), an important research study about treating high blood pressure. We have recently had trouble contacting <<insert participant name>>.

We would appreciate your calling us at << insert clinic phone number>> to provide us with contact information for this SPRINT participant. As the study is ending, we must contact every SPRINT participant to update our records on their blood pressure management and health status.

If you call and we are unavailable, please leave your name and number so that your call can be returned.

Thank you for your assistance in contacting this SPRINT participant. We look forward to speaking with you soon.

Sincerely,

<<Insert Clinic PI Name>>

<<Insert Clinic Contact Information>>

APPENDIX 9 – Permission to Contact You about Health Status

PERMISSION TO CONTACT YOU ABOUT YOUR HEALTH STATUS

You have previously informed your study doctor that you were no longer interested in participating in the Systolic Blood Pressure Intervention Trial (SPRINT) at <<Insert Institution Name Here>>. Knowing your state of health after treatment is important to the final analysis of the study.

We would like to know if you would agree to the study staff contacting you (or your legal representative) when the study is scheduled to end, before May 2016, to ask only about your state of health. This is the only time that we will contact you.

By signing this document, you acknowledge that you have read and understand this permission. Further, you authorize your health information to be used or as follows:

☐ I authorize the study staff to contact me by telephone in order to follow-up with my state of health.

☐ I authorize the study staff to contact my legal representative in order to follow-up with my state of health.

☐ I do not wish to be contacted, nor have my legal representative contacted, by study staff in order to follow-up with my state of health.

Please notify the study doctor or his staff if you move or your contact information changes.
Study Doctor: <<Insert PI Name Here>>
Telephone Number: <<Insert Clinic Contact Phone Number Here>>

____
Printed name of participant

____ ____________________
Signature of participant/authorized legal representative Date

____
Relationship of authorized legal representative

Please insert contact information:

Name: ____

Street Address: ____

City: ________________________________ State: _______ Zip: ______________

Phone number: (____) ______-__________

APPENDIX 10 – Addendum to Consent Form

Model Addendum to the Informed Consent
For Individuals Already Participating in SPRINT
(Systolic blood pressure intervention trial) *First Name*
Last Name, Degree, Principal Investigator

As you were notified in September 2015, the SPRINT study found that treating the systolic blood pressure to a goal of 120 mm Hg (compared to the 140 mm Hg) goal reduced the risk of major complications or death due to heart problems or stroke, as well as the overall risk of death. SPRINT has advised you to talk with your personal health care provider about which blood pressure goal is best for you.

The SPRINT consent form that you signed when you joined the study allows the study to collect medical records for specific study information through national databases. This addendum to the main trial informed consent allows for future contact about SPRINT extension or follow-up studies. Please ask the study doctor or the study staff to explain any words or information that you do not clearly understand. You may take home an unsigned copy of this consent form to think about the information or talk with family or friends before making your decision.

STUDY EXTENSION AND FOLLOW-UP STUDIES

We are working on plans for additional extension and follow-up studies that could include a variety of topics such as blood pressure treatment, memory testing, or other research related to healthy aging for which you could be eligible. Because this research is in the planning phase, at this time we are asking that you sign a consent form that allows us to contact you in the future to ask if you are interested in any SPRINT extension or follow-up study.

If an extension or follow-up study begins, SPRINT staff will ask you if you would like to learn more about it. At that time, you will be given all available information about the extension or follow-up study and will be asked to sign a consent form if you are interested in participating. If we are unable to reach you, we will contact the person you have identified as your alternative contact person. If you do not agree to be contacted for future extension or follow-up studies, we will not contact you or your family members.

WHAT ARE THE RISKS OF EXTENDING YOUR SPRINT ASSOCIATION?

There is no risk to you of consenting to future contact. By consenting to this extension, you are only agreeing to allow us to contact you or your designated contact if an extension or follow-up study is approved.

WHAT OTHER CHOICES ARE THERE?

Taking part in any future extensions or follow-up studies of SPRINT is voluntary. You may choose not to take part or you may leave the study at any time. Leaving the study will not change your relationship with this institution or cause you to lose any benefits to which you are entitled. If you decide to stop participating, you should inform the study investigator as soon as possible. Data collected up until that point may still be used; however no new data will be collected from you.

WHOM DO I CALL IF I HAVE QUESTIONS OR PROBLEMS?

For questions about the study or this new information, contact the study investigator, *Name* at *telephone number (also include afterhours number)*.

The Institutional Review Board (IRB) is a group of people who review the research to protect your rights. If you have a question about your rights as a research participant, or you would like to discuss problems or concerns, have questions or want to offer input, or you want to obtain additional information, you should contact the Chairman of the IRB at *telephone number*.

You will be given a copy of this signed consent form.

Signatures

I have read the information provided above. I voluntarily consent to be contacted in the future about extension or follow-up SPRINT studies. I agree that either the (PI) or someone whom he/she chooses may contact me or my designated contact in the future to ask about taking part in more research.

Participant Name:______________________________

Participant Signature: __________________________Date: _________Time:_______am/pm

Person Obtaining Consent:______________________Date:__________Time:_________am/pm

APPENDIX 11 –Close-out Appointment Reminder Letter

Dear <<Insert participant name here>>,

You are scheduled for a SPRINT visit on <<insert date of appointment>> at <<insert time of appointment>>.

In preparation for this visit please follow these directions:

- ❒ **If checked, this is a FASTING visit.** Please do not eat or drink anything except water for at least 8 hours prior to your visit. Unless directed differently, you may take your normal morning medications with water.
- ❒ **If checked, this is NOT a fasting visit.** You do not need to fast for this visit. Unless directed differently, please take your medications per your regular routine.

PLEASE BRING THE FOLLOWING ITEMS TO YOUR CLINIC VISIT:

- All current medications or updated information on medications that you have taken since your last visit.
- Information on illnesses, injuries, hospitalizations, and medical care that you have experienced since your last visit.
- Any change in your address or phone numbers or the addresses and phone numbers of your contacts.
- Reading glasses, if you need them.
- If you have not already done so, please bring your primary doctor or health care provider's name and contact information so that we can provide him/her with a summary of your study involvement. For convenience you can write it below and bring it to your visit.
  My primary health care provider is: ____________________________
  Address: ____
  ____
  Phone: ______________________Fax: _______________________

Please contact us right away, at <<insert clinic phone number>> if you need to reschedule or if you have any questions. Thank you for being a part of the SPRINT team and for your time and effort in making this study a success. We appreciate your hard work and dedication to the study. We look forward to seeing you for this important study visit.

Sincerely,
The SPRINT Team

APPENDIX 12 – Close-out Encounter and Disposition Form

SPRINT - Encounter & Disposition - Closeout

Participant Name: ______________________

Participant ID: ___ ___ ___ ___ ___ ___ ___ ___

Visit: 12M Site: ___ ___ ___

Date of Visit: __ __/ __ __/ 20 __ __ (mm/dd/yyyy)

Form Completed by: __ __ __ __

Date Entered: __ __/ __ __/ 20 __ __ (mm/dd/yyyy)

Data Entered by: __ __ __ __

Part I. Contact Type

1. Was study data collected for this visit?

   0 ☐ No (Complete Part III, IV, and VI)

   1 ☐ Yes

   If yes, what type of contact is this?

   1 ☐ Clinic

   2 ☐ Home

   3 ☐ Phone

   4 ☐ Proxy (skip to Part III)

   5 ☐ Other (Please specify): ______________________

Part II. Health Information

2. Do you have a healthcare provider for your health needs after the end of the study?

   0 ☐ No (Provide participant with "Tips for Finding a Healthcare Provider and Insurance" document then SKIP to Question 3)

   1 ☐ Yes

   If Yes, confirm PCP Contact Information on the Participant Contact Form is current.

   Prior to this visit, has care for blood pressure treatment management been transitioned to the participant's health care provider?

   0 ☐ No

   1 ☐ Yes

3. Which of the following describes your current type of insurance coverage (mark all that apply)?

1 ☐ Medicare

2 ☐ Medicaid

3 ☐ VA

4 ☐ Affordable Care Act

5 ☐ Private/Other (including Medicare supplement, if applicable)

6 ☐ Uninsured (Provide participant with "Tips for Finding a Healthcare Provider and Insurance" document)

4. How old were you when you were first told by a health care provider that you have high blood pressure? (for women: while you were not pregnant)?

_____ Age (in years)

5. Prior to enrollment in SPRINT, had you ever been prescribed medications for high blood pressure? (for women: while you were not pregnant)?

0 ☐ No

1 ☐ Yes

If Yes, indicate the age when you began taking blood pressure medications.
_____ Age (in years)

6. Since you joined SPRINT on *date of randomization*, for health reasons, have you stayed in a nursing or rehabilitation facility one or more times?

0 ☐ No (skip to Part III)

1 ☐ Yes

If Yes, were you there for a total of

1 ☐ 100 days or less

2 ☐ More than 100 days

If you have stayed in a nursing or rehabilatition facility one or more times since joining SPRINT, how many times have you stayed in one? _____

Do you currently reside in either an assisted living facility or nursing home?

0 ☐ No

1 ☐ Yes (if yes, update Participant Contact Information Form)

Part III. Participant Materials Checklist

7. Check each item off as given/mailed to participant

1 ☐ Participant Bulletin

2 ☐ Certificate of Appreciation

3 ☐ SPRINT jar opener

4 ☐ SPRINT Medical Summary Report (participant copy)

5 ☐ SPRINT Medical Summary Report (health care provider copy)

6 ☐ Health Care Provider Letter

7 ☐ GAP Information (medication sheets and assistance applications, if applicable)

8 ☐ 3-month supply of study medication dispensed (if applicable)

If no materials were given/mailed to participant check here ☐

Part IV. End of Study Final Visit Summary

8. Has the participant signed the SPRINT Addendum to the Informed Consent?

0 ☐ No (If no, indicate reason for refusal below)

1 ☐ Participant not interested in future contact about extension or follow-up studies

2 ☐ Undecided/Needs additional time to review

3 ☐ Other - Please specify ____

1 ☐ Yes

(If yes, date signed ___/___/_____)

9. Has the participant signed an updated Medical Release document?

0 ☐ No

1 ☐ Yes

Part V. Completed Visits

10. Indicate the forms/data that were collected at this visit:

Note that forms highlighted in grey are not expected to be completed at the closeout visit for this participant.

	Collected? No	Collected? Yes
Events Ascertainment Form	☐	☐
Close-out Blood Pressure Management Form	☐	☐
BP Medication Log	☐	☐
Participant Contact Information Form	☐	☐
Close-out Lab Shipment Form (A or B)	☐	☐
MRI CBC Lab Shipment form (only subsample)	☐	☐
MRI Screening Form (only subsample)	☐	☐
Annual History and Physical Exam Form	☐	☐
My Health Form	☐	☐
Men's/Women's Health Form (only subsample)	☐	☐
Falls Self Efficacy Form (only subsample)	☐	☐
Morisky/Patient Satisfaction	☐	☐
ECG	☐	☐
Drug Dispensing Form	☐	☐

11. Were the MIND Screening or MIND Extended Battery forms included in the packet for this visit or did you manually print them out?

0 ☐ No (STOP)

1 ☐ Yes

If yes, did you complete any MIND testing?

0 ☐ No (complete MIND Cover Page(s) only)

1 ☐ Yes

If yes, indicate the tests that were collected at this visit:

1 ☐ MIND Screening Battery

2 ☐ MIND Extended Battery

Part VI. Missed Visits

12. Indicate below the reason the visit was missed

1 ☐ Participant cannot be located

2 ☐ Participant located but unable to make contact or collect data

3 ☐ Participant is hospitalized (document in source notes when, where and why participant was admtted. Complete an SAE form if not previously completed and gather materials for event adjudication.)

4 ☐ Participant died (complete an SAE form)

5 ☐ Scheduling conflict

6 ☐ Participant withdrew consent (Complete a Participant Status Log)

7 ☐ Other, please specify ____

APPENDIX 13 – Close-out Blood Pressure Management Form

SPRINT BP Management Form - Close Out

Participant Name:	________________________________	Date of Visit:	__ __ / __ __ / 20 __ __ (mm/dd/yyyy)
Participant ID:	___ ___ ___ ___ ___ ___ ___ ___	Form Completed by:	__ __ __ __
Visit: 12M	Site: ___ ___ ___	Date Entered:	__ __ / __ __ / 20 __ __ (mm/dd/yyyy)
		Data Entered by:	__ __ __ __

Part A. Current Blood Pressure (Cuff Size used at _____ was ______________________________)

1. ☐ Check here if measurement was not performed using the study automated device.

Seated Measurements

	Systolic	Diastolic	Heart Rate
1st	_____ mmHg	_____ mmHg	_____ bpm
2nd	_____ mmHg	_____ mmHg	_____ bpm
3rd	_____ mmHg	_____ mmHg	_____ bpm
Average	_____ mmHg	_____ mmHg	_____ bpm

2. ☐ Check here and **STOP** if participant is unable to stand.

Standing Measurement (@ 1 minute)

Systolic	Diastolic	Heart Rate
_____ mmHg	_____ mmHg	_____ bpm

3. Did participant experience dizziness or light headed feelings when standing for this exam?
   - ☐ No
   - ☐ Yes

APPENDIX 14 – Close-out Visit A Lab Form

SPRINT - Central Laboratory Specimen Collection Form
Closeout Visit "A"

PID: ___ ___ ___ ___ ___ ___ ___ ___ Site ID: ___ ___ ___ Visit Code: CLA

12345678 *999*

This form is used to accompany specimens drawn for a single subject and shipped to the Central Lab for analysis. Please refer to the Manual of Operations for detailed instruction. This form must be copied and the copy should be retained at the site. The original should be placed into a ziplock bag and then placed inside the orange FedEx Clinical Pak for shipment.

Ship on the day of specimen collection (do not ship on days which precede federal holidays) to:

SPRINT Central Laboratory
Advanced Research and Diagnostic Laboratory
1200 Washington Ave S
Suite 175
Minneapolis, MN 55415

Please place the appropriate pre-printed participant's Laboratory ID sticker in the box to the right:

LABEL ➔ ➔ ➔

Blood Collection

Date: __ __ / __ __ / 20 __ __ Time: __ __ : __ __ AM / PM Initials: ___________

Please check all tubes included in the shipment. Use the checkboxes for each tube to indicate draws.

☐ Tube #1 - CHEM (7.5ml red-top w/ gel separator)

☐ Tube #2 - Urine Alb/Creat (10ml screw-cap vial)

Urine Collection

Date: __ __ / __ __ / 20 __ __ Time: __ __ : __ __ AM / PM Initials: ___________

Specimen Collection/Processing Comments:

APPENDIX 15 – Close-out Visit B Lab Form

SPRINT - Central Laboratory Specimen Collection Form - Closeout Visit "B"

PID: ___ ___ ___ ___ ___ ___ ___ ___	Site ID: ___ ___ ___	Visit Code: CLB
12345678	*999*	

This form is used to accompany specimens drawn for a single subject and shipped to the Central Lab for analysis. Please refer to the Manual of Operations for detailed instruction. This form must be copied and the copy should be retained at the site. The original should be placed into a ziplock bag and then placed inside the orange FedEx Clinical Pak for shipment.

Ship on the day of specimen collection (do not ship on days which precede federal holidays) to:

SPRINT Central Laboratory
Advanced Research and Diagnostic Laboratory
1200 Washington Ave S
Suite 175
Minneapolis, MN 55415

Please place the appropriate pre-printed participant's Laboratory ID sticker in the box to the right:<br><br>**LABEL** ➜ ➜ ➜	

Blood Collection

Date: __ __ / __ __ / 20 __ __ Time: __ __ : __ __ AM / PM Initials: ___________

Has it been > 8 hours since you last ate/drank anything (other than water), including candy and chewing gum? ☐ No ☐ Yes

Please check all tubes included in the shipment. Use the checkboxes for each tube to indicate draws.

☐ Tube #1 - GLU/CHEM/Lipid (7.5ml red-top w/ gel separator)

☐ Tube #2 - Serum Storage (7.5ml red-top w/ gel separator)

☐ Tube #4 - Plasma Storage (8ml EDTA purple-top)

☐ Tube #5 - Plasma Storage (8ml EDTA purple-top)

☐ Urine Alb/Creat/ Storage (10ml screw-cap vial)

Urine Collection

Date: __ __ / __ __ / 20 __ __ Time: __ __ : __ __ AM / PM Initials: ___________

Specimen Collection/Processing Comments:

APPENDIX 16 – MRI CBC Lab Form

SPRINT - Central Laboratory Specimen Collection Form
MRI CBC

PID: ___ ___ ___ ___ ___ ___ ___ ___	Site ID: ___ ___ ___	Visit Code: CBC
12345678	*999*	

This form is used to accompany specimens drawn for a single subject and shipped to the Central Lab for analysis. Please refer to the Manual of Operations for detailed instruction. This form must be copied and the copy should be retained at the site. The original should be placed into a ziplock bag and then placed inside the orange FedEx Clinical Pak for shipment.

Ship on the day of specimen collection (do not ship on days which precede federal holidays) to:

SPRINT Central Laboratory
Advanced Research and Diagnostic Laboratory
1200 Washington Ave S
Suite 175
Minneapolis, MN 55415

Please place the appropriate pre-printed participant's Laboratory ID sticker in the box to the right:

LABEL ➡ ➡ ➡

Blood Collection

Date: __ __ / __ __ / 20 __ __ Time: __ __ : __ __ AM / PM Initials: ___________

Please check all tubes included in the shipment. Use the checkboxes for each tube to indicate draws.

☐ Tube #1 - CBC (4mL purple top)

Specimen Collection/Processing Comments:

APPENDIX 17 – Tips for Finding a Healthcare Provider or Insurance

HELPFUL HINTS ABOUT FINDING A HEALTH CARE PROVIDER OR INSURANCE

There are several resources you can use to find a health care provider:

- Discuss options of an internal physician referral within the SPRINT clinic's health care system.
- Talk to friends, neighbors, or co-workers about their health care providers.
- Identify a few doctors in a physician's guide who are conveniently located. A few example website are listed below.
- Search the Internet for providers and provider groups located near you.
    - Google: "Doctors search *name of state*"
    - Ask.Com: "Doctors *name of state*"
    - Web MD Physician Directory: doctor.webmd.com
    - Plan Finder website: finder.healthcare.gov
    - Healthgrades.com
    - Health Resources and Services Administration: findahealthcenter.hrsa.gov
    - National Association of Free and Charitable Clinics: nafcclinics.org
    - Medicare.gov: www.medicare.gov/physiciancompare/
    - Veterans Affairs (VA): va.gov/healthbenefits/apply
- If you have a health insurance plan, you usually can call a toll-free plan number for advice on finding a health care provider, and insurance providers often have a website with a feature for searching online for health care providers within the network.

Other questions to consider before selecting a health care provider are:

- What are the health care provider's qualifications?
- Is the health care provider's office conveniently located near your home or work place?
- What is the health care provider's availability – such as are they accepting new patients and how long does it take to get an appointment?
- What are the office hours and policies regarding urgent care appointments?
- Does the health care provider take your insurance, and how are co-payments or other financial matters handled?
- Are there additional services such as access via email or Internet to ask questions or to request appointments or prescription refills?

If you don't currently have health care insurance, open enrollment for 2016 coverage through the Affordable Care Act is November 1, 2015 through January 31, 2016. Some helpful websites are:

www.healthcare.gov
www.getcoveredamerica.org

The Medicare website (www.medicare.gov) is helpful to determine if you are eligible and to help calculate your premium. Even if you aren't 65, you may still be eligible.

If you are a veteran and meet the basic eligibility requirements, the VA encourages you to apply today by completing and submitting "VA Form 10-10EZ, Application for Health Benefits" online at www.1010ez.med.va.gov/.

For more detailed information about Medicare, the Affordable Care Act and the VA health benefits, please speak with your SPRINT clinic and use the SPRINT public web site (www.sprinttrial.org.) to review and download information that may be helpful to you regarding your health care.

APPENDIX 18 – Letter to Healthcare Provider

<<Insert Date Here>>

Dear <<Insert HCP Name Here or if Not Known ‘Health Care Provider’>>

We are pleased to inform you that your patient, << Insert Participant Name Here>> has completed participation in the Systolic Blood Pressure Intervention Trial (SPRINT). More than 9,300 people participated in this National Institutes of Health sponsored nationwide trial, which tested whether treating systolic blood pressure to a goal of <120 mmHg would reduce the risk of heart and kidney disease, stroke, or dementia more than the current systolic blood pressure goal of 140 mmHg.

Study participants were randomized to intensive blood pressure control (systolic blood pressure <120 mmHg) or standard blood pressure control (systolic blood pressure < 140 mmHg). The composite primary outcome was non-fatal myocardial infarction, acute coronary syndrome, heart failure, non-fatal stroke, or cardiovascular death. ***The group assigned to the intensive systolic blood pressure goal of <120 mm Hg (compared to the group assigned to the 140 mm Hg goal) showed a 30% overall reduction in the risk of the events in the composite endpoint, as well as an overall lower risk of death.*** For further information about the results of the SPRINT trial, please visit the study website, www.sprinttrial.org.

For your information, your patient’s group assignment, blood pressure history, and other clinical parameters throughout the study can be found in the attached summary. His/her current antihypertensive medication regimen is also listed in this document.

<<Insert Participant’s Name here>> has been advised to call your office for an appointment to resume management of hypertension with you. Thank you for supporting your patient’s participation in this important study. If you have any questions regarding the care of this patient, please do not hesitate to contact our office at <<Insert Clinic Contact Phone Number here>>.

Sincerely,

<<Insert Site PI Name Here>>

<<Insert Site Contact Information Here>>

The SPRINT Team

APPENDIX 19 – Study Medical Summary

SPRINT STUDY MEDICAL SUMMARY REPORT **Study ID: 10000550**

Mary Jane Watson
Principal Investigator: **Dr.**______________________________
Randomization Visit Date: **10/31/2010** Close-out Visit Date: __________________

Clinical Center: **Coordinating Center Test Site**
(phone:____________________)
Randomized to: **Intensive Blood Pressure**

Notes:

Antihypertensive medications:

Date	Medication
10/08/2015	atenolol[25MG] spironalactone[25MG] amlodipine[2.5MG]

Adverse events reported during study:

Event	Date	Code
Init Hosp	08/02/2011	10002388 - Angina unstable
Init Hosp	08/05/2011	10008479 - Chest pain
Init Hosp	11/03/2011	10031161 - Osteoarthritis
Init Hosp	05/09/2012	10050953 - Lower gastrointestinal haemorrhage
Init Hosp	05/18/2012	10013573 - Dizziness
Init Hosp	02/24/2013	10008479 - Chest pain
ER	03/22/2013	10016173 - Fall
Init Hosp	05/22/2014	10072286 - Narcotic bowel syndrome

Systolic blood pressure over the course of the SPRINT study

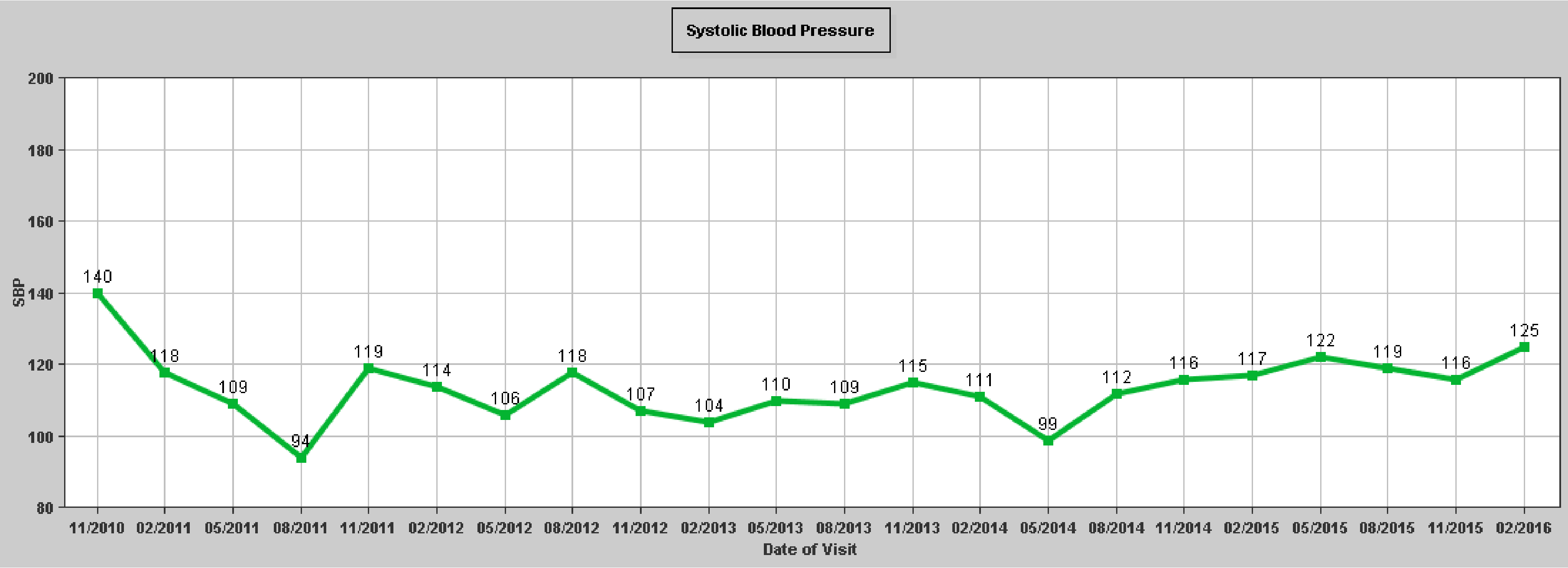

Diastolic blood pressure over the course of the SPRINT study

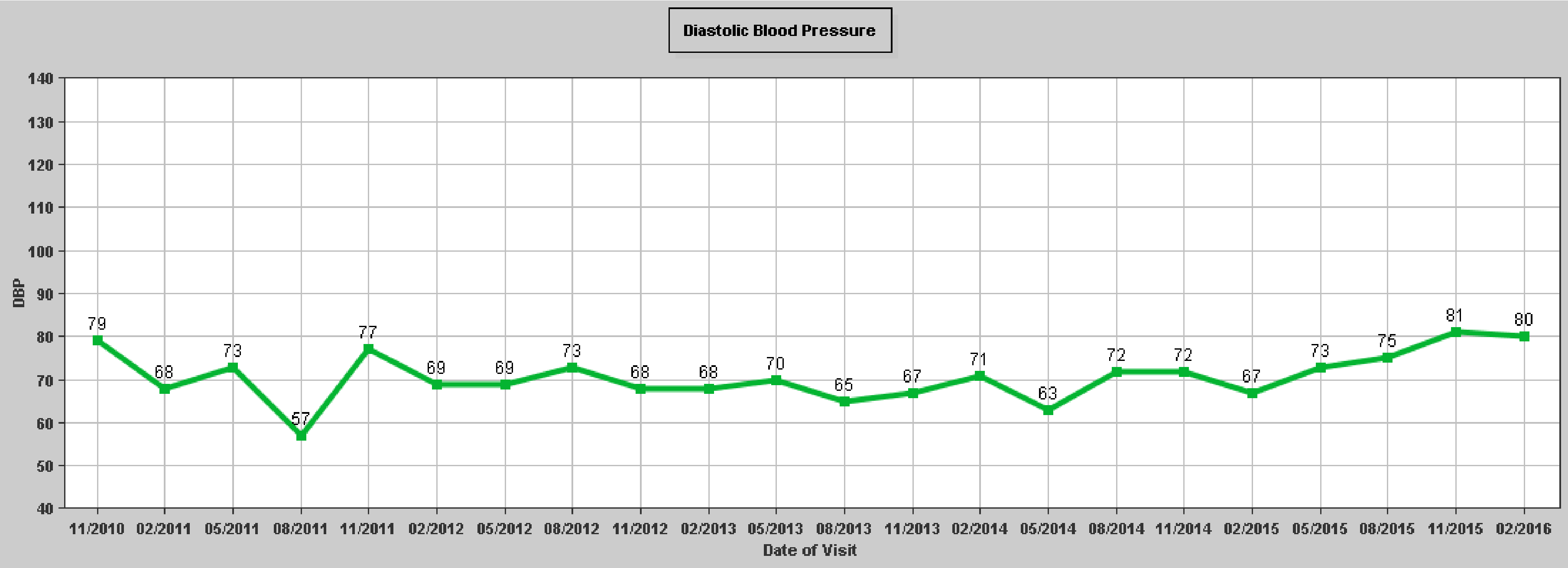

Heart Rate over the course of the SPRINT study

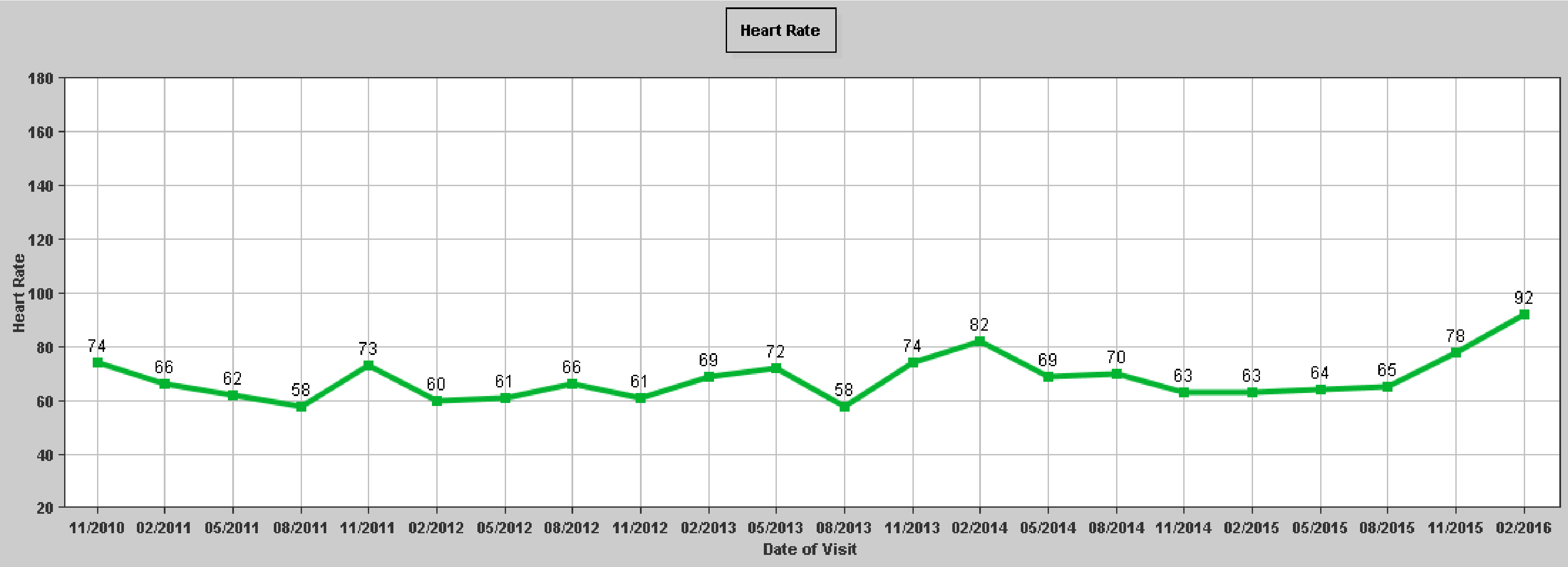

Selected measures over the course of the SPRINT study:

Date	Fasting	K (mmol/L) [3.3-5.1]	BUN (mg/dL) [6-23]	Serum Cr (mg/dL) [0.40-1.20]	eGFR (ml/min/1.73 m2)	Sodium (mmol/L) [133-145]	Chloride (mmol/L) [96-108]	BiCarb (mmol/L) [22-29]	Urine Cr (mg/dL) [Male 40-278 Female 29-226]	Chol (mg/dL) [0-200]	HDL (mg/dL) [Male >40 Female >50]	LDL (mg/dL) [0-129]	Trig (mg/dL) [<150]	Alb/Cr Ratio (mg/g Cr) [<30]	Urine Microalb (mg/L)	Glucose (mg/dL) [60-99]
12/27/2010		4.6	42	1.30	39.56	143	105	27	50	146	54	78	70	14.00	7	87
01/27/2011	Yes	4.7	40	1.27	40.63	141	104	28								
03/28/2011		4.4	40	1.25	41.37	142	106	23								
06/28/2011		5.0	40	1.33	38.48	136	104	23	24					8.33	2	
01/04/2012	No	4.3	51	1.45	34.79	143	104	29	24	152	49	85	91	8.33	2	
06/26/2012		4.1	33	1.39	36.48	136	99	25								
01/03/2013	Yes	4.6	39	1.51	33.11	141	102	28	64	128	45	66	85	14.06	9	101
06/26/2013		4.4	46	1.31	38.97	142	103	26								

APPENDIX 20 – Certificate of Appreciation

Certificate of Appreciation

The Sponsors and Collaborators of

SPRINT
Systolic Blood Pressure Intervention Trial

Gratefully acknowledge the outstanding contribution of

On this day,

Paul K. Whelton, M.D., Steering Committee Chairman	Clinical Site Principal Investigator
Lawrence J. Fine, M.D., DrPH, NHLBI Project Officer	Clinical Site Coordinator

APPENDIX 21 – Participant Bulletin

SPRINT
Systolic Blood Pressure Intervention Trial

THANK YOU, SPRINT VOLUNTEERS!

Thank you for your dedication and commitment to the SPRINT Trial. We appreciate the time and effort you gave for your clinic visits, phone calls, medication changes, blood draws, and answering the many questions you were asked during the study.

As part of SPRINT, you were one of 9,361 volunteers dedicated to testing the effects of intensive control of systolic blood pressure on your risk of having a heart attack or a stroke. It is because of your hard work that we were able to successfully answer this important question – **we could not have done this without you!**

The final visits for SPRINT volunteers are underway and will end by the end of April 2016. We are committed to helping you through this transition from blood pressure care provided by SPRINT to that of your health care provider. This bulletin provides answers to some questions you may have; your SPRINT clinic staff is ready to help. Feel free to share your concerns and questions with them.

Thank you again for your dedication to SPRINT. You should be very proud of the part you played in exploring treatment plans for controlling blood pressure. The SPRINT findings will likely have a significant impact on treatment approaches used in the future.

What About the Study Results?

• • •

In September you received a letter telling you that the study found that treatment to the systolic blood pressure goal of 120 mm Hg (compared to the 140 mm Hg goal) reduced the risk of major complications or death due to heart problems, as well as the overall risk of death.

We are collecting final data and analyzing study results, and your SPRINT clinic will let you know when the SPRINT results are published.

What Now? Treating Your Blood Pressure after SPRINT

Even though SPRINT is ending, your blood pressure still needs to be treated. Your SPRINT clinic staff will work with your health care provider to ensure a smooth transfer of your care.

<u>What will my BP goal be after SPRINT?</u>

All SPRINT participants currently have a systolic blood pressure goal less than 140 mm Hg (or less than 120 mm Hg). Now that you have finished the study, it is up to you and your health care provider to determine your blood pressure goal. Your goal will depend on a number of factors, including if you have any other health conditions and availability of the medications. Be sure to discuss this with your health care provider.

<u>What if my primary health care provider is not my SPRINT doctor?</u>

Many SPRINT participants will be returning to a previous provider or moving to a new one. To help with this transition, a letter for your health care provider is included in this SPRINT Participant Kit.

<u>What about my SPRINT study records?</u>

Included in this SPRINT Participant Kit are two copies of your Medical Summary Report that summarizes your study history and treatment, including information about medicines. One copy is for you to keep for your records. With your permission, your SPRINT staff can send one copy of this report to your current or new healthcare provider, along with a letter describing the SPRINT study. To be able to send these on to your health care provider, you will be asked to sign a medical record release. If a medical event related to your SPRINT medications occurs after your final visit, SPRINT may need more information about that event. If a medical release is needed, your SPRINT staff will contact you and ask you to complete one.

<u>May I enroll in another study?</u>

Yes, other research studies may be available in your area. Your clinic staff may be able to help you locate one to participate in if you are interested. Your ability to enroll in another study will depend on the requirements for the study, and you should tell the new study staff that you have just finished SPRINT. Even if you enroll in another study, you still may need to find a provider for your medical care.

What about Medications?

You will receive a 3-month supply of your current SPRINT medications at your last SPRINT visit. (However, if you were not taking SPRINT medications, you will not receive any medications.) Please note that you will not get any more medications from SPRINT after this 3-month supply. If you do not have a healthcare provider now, it is important that you find one soon! Your SPRINT clinic can assist you if you need help finding a provider.

Additionally, the SPRINT Participant Kit includes an information sheet for each medication you received. This sheet provides instructions for use and lists possible medication side effects.

Are there programs that can help me get free or low-cost blood pressure medicines?

Yes. Information on assistance programs can be found in your SPRINT Participant Kit. This includes information on assistance programs through drug companies or retail stores.

What about an Extension?

Will there be an extension or follow-up to SPRINT?

At this time it is not known whether there will be an extension. For example, an observational extension to SPRINT is being considered, but it has not been finalized. Observational studies, which are common after large clinical trials like SPRINT finish, do not give you treatments; instead you are contacted on a regular basis to see how you are doing. Observational extension studies give researchers a chance to gather more information on long-term effects of the study treatments.

Your Medical Care Is Important!

Be a partner with your health care provider in your medical care!

Share with others everything you learned in SPRINT!

• • •

If you don't have a health care provider, find one now!

Your SPRINT clinic can help you with your search, just ask!

If an extension study begins, your SPRINT clinic staff will ask you if you would like to learn more about it. At that time, you will be given all available information about the study and will be asked to sign a consent form if you are interested in participating.

In addition, we are working on plans for additional follow-up studies that could include a variety of topics such as blood pressure treatment, memory testing, or other research related to healthy aging for which you or your family could be eligible. Because this research is in the planning phase, at this time we are simply asking that you sign a consent form that allows us to contact you in the future to ask if you are interested in any SPRINT extension or follow-up study.

If there is an extension, will I be provided with medications?

If the study is an observational extension, we only collect health information. People who volunteer to participate in this kind of study will be asked to answer health questions either in person or by phone, but no medications will be provided.

Materials in this Kit

In addition to this bulletin, your SPRINT Participant Kit also contains the following items:

Thank-You Gift

A certificate of appreciation and a SPRINT jar opener.

Medical History Report

A report including recent history of your participation in SPRINT. Two copies will be provided, one for your records and one to share with your health care provider, if you wish.

Provider Letter

If your health care provider is not your SPRINT doctor, a letter for your provider about your participation in SPRINT.

Medication Information Sheets

Instructions for taking your medications, including a list of possible side effects, information on assistance programs, and available applications for those programs.

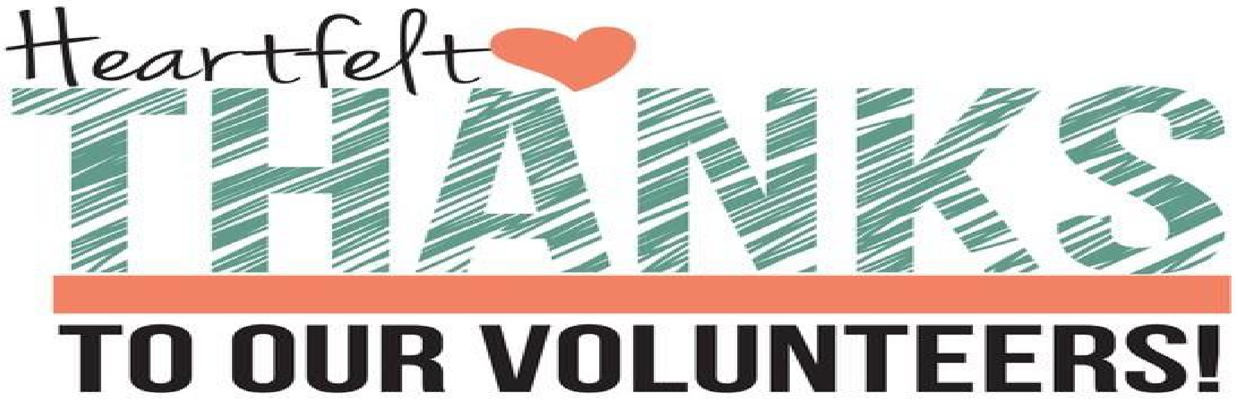

Participant Spotlight: SPRINT Participant Mike Korologos

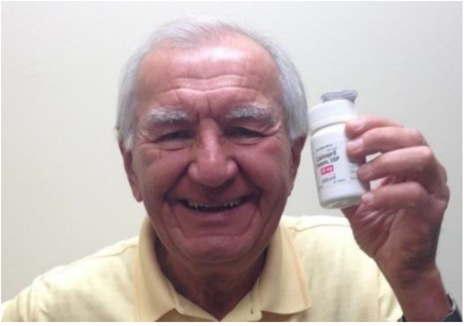

Mike is a native of Salt Lake City, Utah who graduated from the University of Utah as a journalism & political science graduate. Mike is a retired Salt Lake Tribune sports writer & columnist and newsroom executive and a public relations consultant who was the former Press Chief for the Organizing Committee of the 2002 Olympic Winter Games in Salt Lake City. Mike now enjoys golfing, skiing, traveling, hiking and photography.

What Led You To Your Decision To Join SPRINT?

I'm a strong advocate of research of almost any kind so I was excited to be a participant in a national study on heart disease in hopes that in some small way I would be helping an important cause.

What Do You Do To Keep Healthy?

I have never smoked and I do moderate exercise. I supplement my stretching & light weight lifting/pulling with cardio exercise by pedaling a stationary bike and walking on a treadmill vigorously for at least an hour 3-5 times a week. I am careful with my food intake and keep close tabs on my weight so it doesn't get too far on the upside. I shun desserts (most of the time!) and try hard to avoid snacking on such foods as cookies, potato chips, candy, etc. I satisfy my snack cravings by nibbling on veggies like carrots, cauliflower, and celery which I keep in chilled water in the refrigerator.

What Would You Say To Another Person Your Age To Inspire Them To Maintain Or Even Improve Their Health?

Stay active mentally as well as physically in order to experience a good quality of life. By being healthy you can do more things that bring you pleasure like golfing, hiking, camping, or whatever your interest. Such pleasurable activities build on each other as they are stimulating and challenging. They can also inspire you to get up and move which in turn gives you something to look forward to that adds a little excitement and spice to your life that can make you feel better mentally and physically.

How Do You Keep Such A Positive Attitude?

I talk to myself. When I feel down or sorry for myself, I say: "OK, so you don't like how you feel. Well, you're the only one who can change that. Get on with it and change your feelings. Get over it." Don't waste the day with negative vibes and with self-pity, but enjoy it and soak it in. As a one-time sports writer, I heard a lot of coaches admonish their players to play better so on my down days and moments I give myself a talk that a coach might give to his team.

APPENDIX 22 – Drug Transfer Form (Clinic to Clinic)

DATE: ______________________ Page___ of ____

DRUG TRANSFER NOTICE
SPRINT (P213)

Fax or E-Mail this immediately to:

Norbert Archibeque
Norbert.archibeque@va.gov
Pharmaceutical Project Manager
SPRINT Drug Distribution Center
VA Cooperative Studies Program Clinical Research Pharmacy Coordinating Center (151-I)
2401 Centre Avenue SE, Albuquerque, NM 87106-4180
505-248-3205

The following bottles have been shipped **From:** **SITE No.: __________**

To: **SITE No.: __________**

DRUG: ______________________________

BOTTLE NUMBERS: ___________
____
____
____
____

DRUG: ______________________________

BOTTLE NUMBERS: ___________
____
____
____
____

Person Completing this Form at Original Site: ______________________________________

Date Drug Transferred to New Site: ______________________________________

UPON RECEIPT OF THE STUDY DRUG AT NEW SITE:

Person Accepting Drug: ______________________________________

Date Drug Received: ______________________________________

* Reproduce for additional copies.
