# Supplementary material for: Association of Intensive Blood Pressure Control and Living Arrangement on Cardiovascular Outcomes by Race: Post Hoc Analysis of SPRINT Randomized Clinical Trial
Source: JAMA Netw Open. 2022 Mar 14;5(3):e222037. doi: 10.1001/jamanetworkopen.2022.2037 (PMC9608340; doi:10.1001/jamanetworkopen.2022.2037)
Supplement: Supplement 2. — eAppendix. Transportability Approach to Extend SPRINT Findings to the External Target Populations eFigure 1. Trends in Systolic Blood Pressure and Number of Antihypertensive Medications According to the Living Arrangement Status and Treatment Assignment Among Black Individuals and Non-Black Individuals eFigure 2. Association of Intensive Blood Pressure Control With Cardiovascular Outcomes, All-Cause Mortality, and Composite Of Cardiovascular Outcomes And All-Cause Mortality According to Living Arrangement Among Black Individuals and Non-Black Individuals Adjusting For Baseline Characteristics eFigure 3. Association of Intensive Blood Pressure Control (vs Standard Control) With the Secondary Outcomes Among the Hypothetical Population by Varying the Distribution of Black Individuals and People Living Alone eTable 1. Questionnaires About Race and Living Arrangement in SPRINT eTable 2. Distribution of Incident Serious Adverse Events According to the Living Arrangement Status and Treatment Assignment Among Black Individuals and Non-Black Individuals [file jamanetwopen-e222037-s002.pdf]

Supplemental Online Content

Inoue K, Watson KE, Kondo N, et al. Association of intensive blood pressure control and living arrangement on cardiovascular outcomes by race: post hoc analysis of SPRINT randomized control trial. *JAMA Netw Open*. 2022;5(3):e222037.
doi:10.1001/jamanetworkopen.2022.2037

eAppendix. Transportability Approach to Extend SPRINT Findings to the External Target Populations
eFigure 1. Trends in Systolic Blood Pressure and Number of Antihypertensive Medications According to the Living Arrangement Status and Treatment Assignment Among Black Individuals and Non-Black Individuals
eFigure 2. Association of Intensive Blood Pressure Control With Cardiovascular Outcomes, All-Cause Mortality, and Composite of Cardiovascular Outcomes And All-Cause Mortality According to Living Arrangement Among Black Individuals and Non-Black Individuals Adjusting For Baseline Characteristics
eFigure 3. Association of Intensive Blood Pressure Control (vs Standard Control) With the Secondary Outcomes Among the Hypothetical Population by Varying the Distribution of Black Individuals and People Living Alone
eTable 1. Questionnaires About Race and Living Arrangement in SPRINT
eTable 2. Distribution of Incident Serious Adverse Events According to the Living Arrangement Status and Treatment Assignment Among Black Individuals and Non-Black Individuals

This supplemental material has been provided by the authors to give readers additional information about their work.

© 2022 Inoue K et al. *JAMA Network Open*.

eAppendix. Transportability Approach to Extend SPRINT Findings to the External Target Populations

1. Concept of transportability

It is often challenging for researchers and practitioners to make meaningful inferences about the external validity of RCT results. When the study population is a random sample of the target population, we can simply generalize the findings to the target population. However, almost all research in the real world does not have such a perfect setting, and the researchers and readers need to consider whether and to what extent the findings are transportable or generalizable to the population of their interests. For example, in RCTs, participants are likely to have a high risk of the outcome of interest (to obtain sufficient statistical power) and have health-seeking behaviors. Even when the trial sample is a random subset of the target population, we also need to consider extending the results to that same population at a different time point if the distribution of the individual's characteristics varies over time. This is particularly important for the analysis focusing on living arrangement status given the rapidly increasing number of older adults living alone in the US over the last several decades.

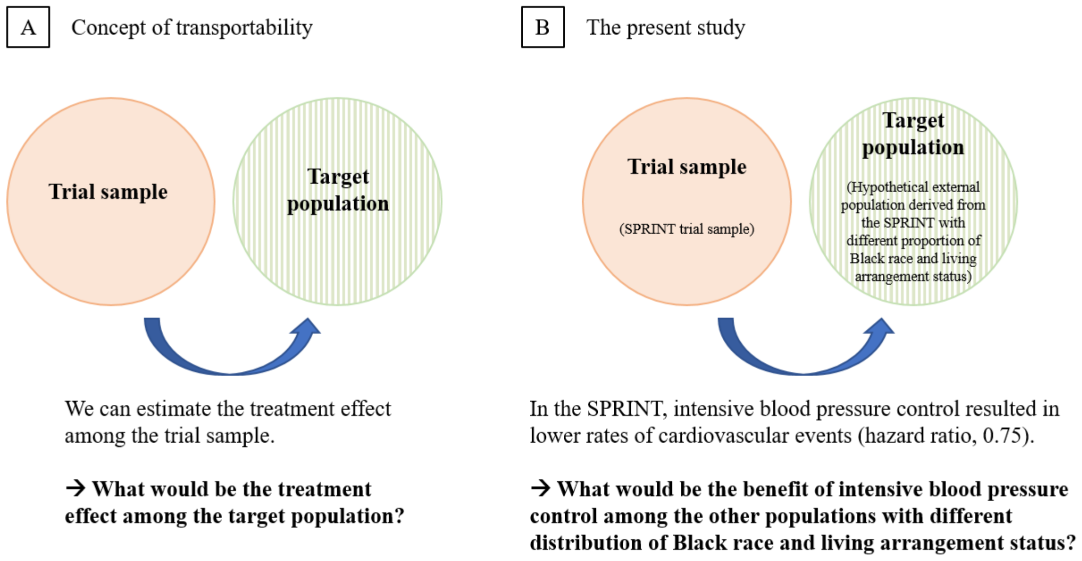

© 2022 Inoue K et al. *JAMA Network Open.*

Transportability is a statistical approach to extend the RCT results to the target population of interest by comparing the distribution of baseline characteristics that modify the effect of the intervention under consideration (i.e. intensive blood pressure control in the present study).[1–5] It is important to define the target population to make meaningful inferences about the external validity of study findings.[6,7] Once the target population is well-defined, the distribution of participants' characteristics and the details of intervention need to be compared between the target population and study sample.

2. Causal framework

Within the counterfactual framework,[8] the average treatment effect can be calculated by taking a contrast of the average potential outcome under two different interventions; i.e. $E[Y_{X=1}]$ and $E[Y_{X=0}]$ where X denotes the treatment of interest (1, treated; 0, untreated), Y denotes the outcome of interest, and $Y_{X=a}$ denotes the potential outcome if the exposure had taken value $X = a$. Therefore, the average treatment effect (in the absolute difference scale) among the study sample can be written as $E[Y_{X=1} | S=1]−E[Y_{X=0} | S=1]$, and that among the target population can be written as $E[Y_{X=1} | S=0]−E[Y_{X=0} | S=0]$ where $S=1$ and $S=0$ denote the study sample and the target population, respectively.

Example causal diagram in randomized controlled trials

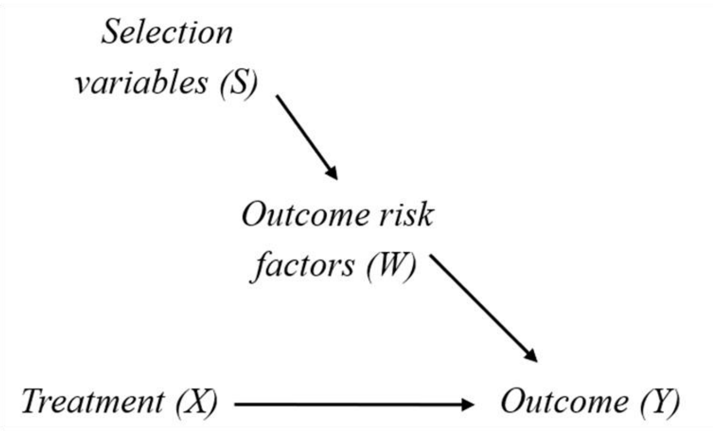

Notation: treatment, X (1, treated; 0, untreated); outcome, Y; outcome risk factors or confounders, W; an indicator of the study sample, S (1, study sample; 0, target population).

© 2022 Inoue K et al. *JAMA Network Open*.

Assumptions necessary to conduct transportability analysis include:[1,2]

(i) Conditional exchangeability over study participation (so-called "S-admissibility"): the participants enrolled in SPRINT are exchangeable with those in the hypothetical target population conditional on covariates

(ii) Conditional exchangeability over intervention in the study participant population: the participants in the intensive blood pressure control group are exchangeable with the participants in the standard blood pressure control group. This assumption is expected to hold by randomization in SPRINT.

(iii) Positivity of RCT participation and intervention assignment: the probability of being in SPRINT is not zero in any stratum defined by covariates, and the probability of being in the intensive blood pressure control group is not zero.

(iv) Consistency: the potential outcome under a specified intervention (intensive blood pressure control) for any individual who received that intervention is equal to the individual's observed outcome.

(v) No interference: one participant's blood pressure control does not influence other participants' cardiovascular event outcomes.

(vi) No measurement error: all variables in SPRINT are correctly measured.

(vii) Correct model specification: the used to determine whether the participant is in SPRINT or the hypothetical target population are correctly specified.

3. Estimation

In this study, we employed the following five steps (the inverse-odds weighting approach[2,5]) to estimate the average treatment effects across hypothetical target populations with different

© 2022 Inoue K et al. *JAMA Network Open.*

distributions of Black individuals and people living alone that were derived from the original SPRINT trial. The distribution of other covariates among those hypothetical target populations remained the same as in the original trial. Because we simulated hypothetical target populations, the above-mentioned assumptions were considered to hold.

Step 1. Create a copy of the original SPRINT data but with a different distribution of Black race and living arrangement status (i.e., randomly assign these variables based on their proportions among the hypothetical target population of interest).

Step 2. Calculate the inverse odds of sampling weights using the following equation:

$$\left[\frac{\text{Probability of being in the target population given Black race and living arrangement status}}{\text{Probability of being in the SPRINT trial given Black race and living arrangement status}}\right]$$

$$\times \left[\frac{\text{Probability of being in the SPRINT trial}}{\text{Probability of being in the target population}}\right]$$

Step 3. Assign the weights calculated (in Step 2) to individuals in the original SPRINT data which has follow-up information on the outcomes. Assign 0 to individuals in the copied data (i.e., hypothetical target population).

Step 4. Employ Cox proportional hazard models along with the assigned weights (in Step 3) to estimate the average treatment effects of intensive blood pressure control on cardiovascular outcomes among hypothetical target populations (i.e., emulate the hypothetical target population from the original SPRINT participants using the inverse odds of sampling weights),

Step 5. Calculate the 95% confidence interval by 5000 bootstrapped samples.

Notice that the calculated 95% confidence interval is affected by the sample size of the target population (n=9,342 in our study i.e., the total number of participants enrolled in the original SPRINT), and thus need to be carefully interpreted. In addition, because the SPRINT included

© 2022 Inoue K et al. *JAMA Network Open*.

participants with high CVD risk and without diabetes, this approach does not allow us to transport the results to population with low CVD risk or those with diabetes. Further details in graphical presentation and mathematical expressions of transportability formula can be found in prior literature.[1–5]

References:

1. Bareinboim E, Pearl J. A General Algorithm for Deciding Transportability of Experimental Results. *Journal of Causal Inference*. 2013;1(1). doi:10.1515/jci-2012-0004
2. Inoue K, Hsu W, Arah OA, Prosper AE, Aberle DR, Bui AAT. Generalizability and Transportability of the National Lung Screening Trial data: Extending trial results to different populations. *Cancer Epidemiol Biomarkers Prev*. Published online September 20, 2021:cebp.0585.2021. doi:10.1158/1055-9965.EPI-21-0585
3. Lesko CR, Buchanan AL, Westreich D, Edwards JK, Hudgens MG, Cole SR. Generalizing Study Results: A Potential Outcomes Perspective. *Epidemiology*. 2017;28(4):553-561. doi:10.1097/EDE.0000000000000664
4. Dahabreh IJ, Robertson SE, Steingrimsson JA, Stuart EA, Hernán MA. Extending inferences from a randomized trial to a new target population. *Statistics in Medicine*. 2020;39(14):1999-2014. doi:10.1002/sim.8426
5. Westreich D, Edwards JK, Lesko CR, Stuart E, Cole SR. Transportability of Trial Results Using Inverse Odds of Sampling Weights. *Am J Epidemiol*. 2017;186(8):1010-1014. doi:10.1093/aje/kwx164
6. Rothwell PM. External validity of randomised controlled trials: “To whom do the results of this trial apply?” *The Lancet*. 2005;365(9453):82-93. doi:10.1016/S0140-6736(04)17670-8

© 2022 Inoue K et al. *JAMA Network Open.*

7. Rothman KJ, Gallacher JEJ, Hatch EE. Why representativeness should be avoided. *Int J Epidemiol*. 2013;42(4):1012-1014. doi:10.1093/ije/dys223

8. Pearl J. *Causality*. Cambridge University Press; 2009.

© 2022 Inoue K et al. *JAMA Network Open.*

eFigure 1. Trends in Systolic Blood Pressure and Number of Antihypertensive Medications According to the Living Arrangement Status and Treatment Assignment Among Black Individuals and Non-Black Individuals

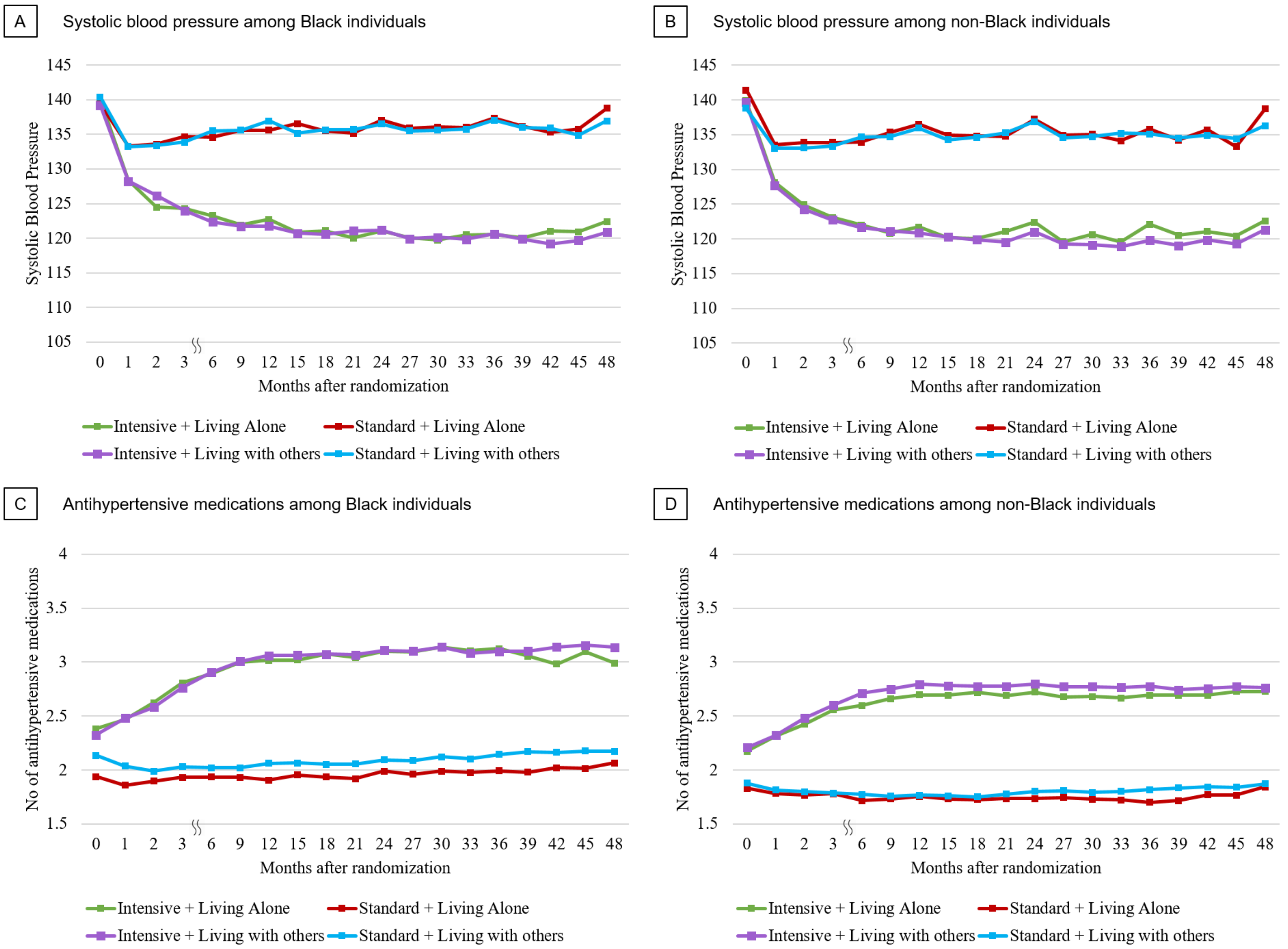

© 2022 Inoue K et al. *JAMA Network Open.*

eFigure 2. Association of Intensive Blood Pressure Control With Cardiovascular Outcomes, All-Cause Mortality, and Composite Of Cardiovascular Outcomes And All-Cause Mortality According to Living Arrangement Among Black Individuals and Non-Black Individuals Adjusting For Baseline Characteristics

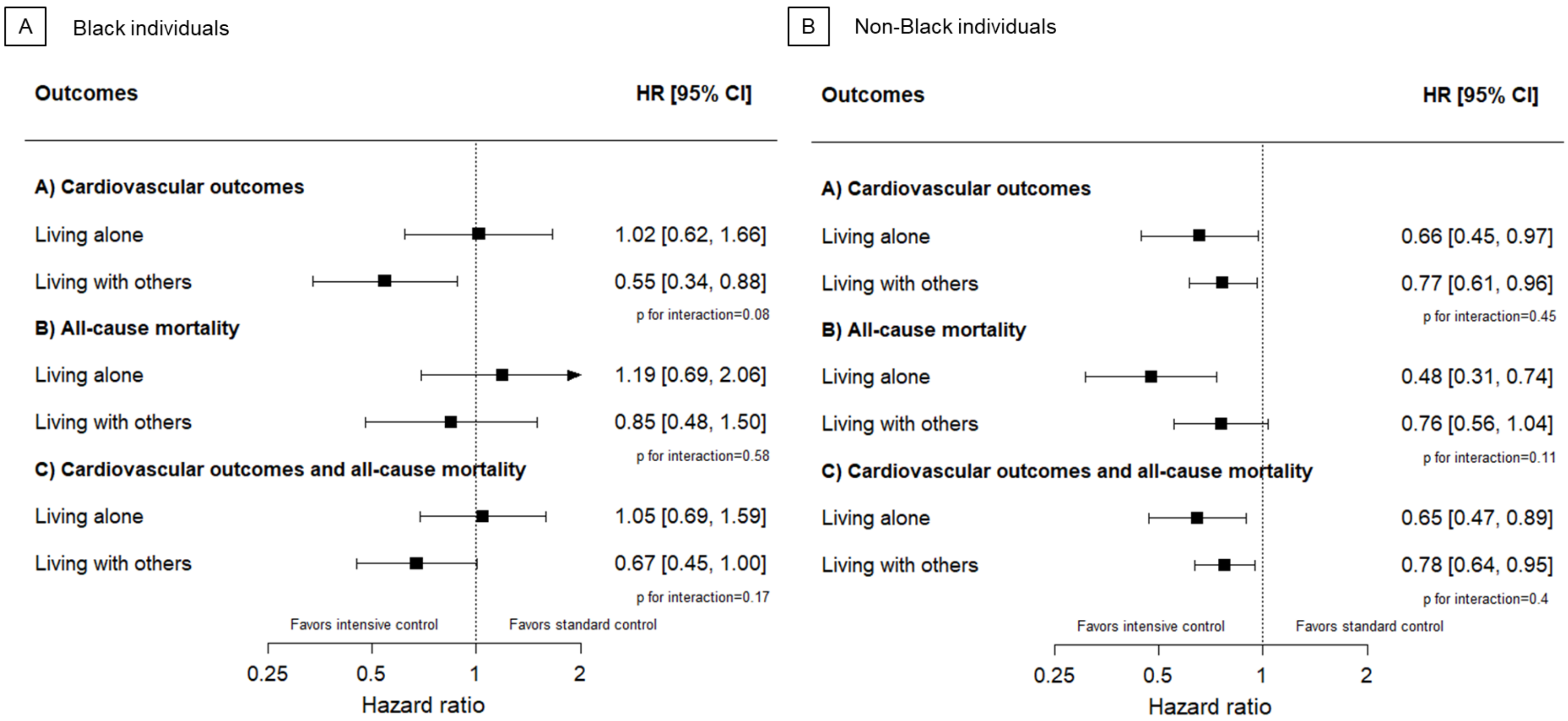

Adjusted for sex, age, education status, insurance status, smoking status, alcohol intake, systolic blood pressure, prior history of clinical cardiovascular disease, prior history of subclinical cardiovascular disease, statin use, aspirin use, number of antihypertensive medications, and 10-y Framingham cardiovascular disease risk score.

© 2022 Inoue K et al. *JAMA Network Open*.

eFigure 3. Association of Intensive Blood Pressure Control (vs Standard Control) With the Secondary Outcomes Among the Hypothetical Population by Varying the Distribution of Black Individuals and People Living Alone

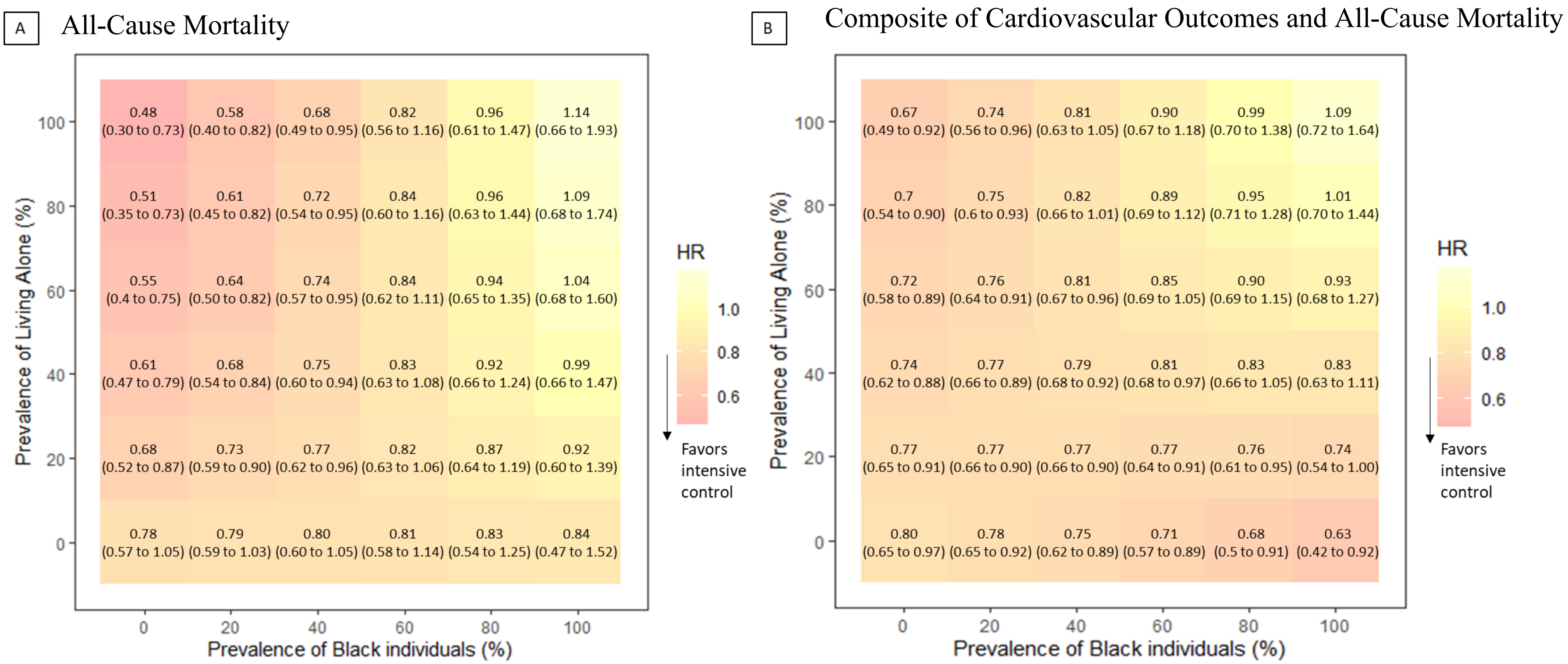

The 95% confidence intervals for each estimate were calculated by 5000 bootstrapped samples.

© 2022 Inoue K et al. *JAMA Network Open.*

eTable 1. Questionnaires About Race and Living Arrangement in SPRINT

<u>Race:</u> Information on race was provided as "White", "Black", "Hispanic", or "Other" in the SPRINT-Primary Outcome Paper data based on the questionnaires below.

- *Is participant of Spanish, Hispanic or Latino Origin?*
  Responses: No, Yes (Puerto Rican), Yes (Cuban), Yes (Mexican, Mexican American, Chicano), or Yes (other)

- *What is participant's race/ethnicity?*
  Responses: White/Caucasian, Black/African American, American Indian/Alaska Native, Native Hawaiian/Pacific islander, Asian, or Other

<u>Living arrangement:</u>

- *Do you live with one or more other adults?*
  Response: Yes, or No

© 2022 Inoue K et al. *JAMA Network Open.*

eTable 2. Distribution of Incident Serious Adverse Events According to the Living Arrangement Status and Treatment Assignment Among Black Individuals and Non-Black Individuals

Variables	Black individuals living alone (N=1001)		Black individuals living with others (N=1792)		Non-Black individuals living alone (N=1713)		Non-Black individuals living with others (N=4836)	
	**Intensive treatment (N=493)**	**Standard treatment (N=508)**	**Intensive treatment (N=881)**	**Standard treatment (N=911)**	**Intensive treatment (N=860)**	**Standard treatment (N=853)**	**Intensive treatment (N=2435)**	**Standard treatment (N=2401)**
The number (%) of participants who experienced the occurrence of serious adverse events	186 (37.7)	183 (36.0)	294 (33.4)	326 (35.8)	343 (39.9)	354 (41.5)	969 (39.8)	872 (36.3)
	HR (95%CI) = 1.09 (0.89 to 1.33)		HR (95%CI) = 0.91 (0.78 to 1.06)		HR (95%CI) = 0.94 (0.81 to 1.10)		HR (95%CI) = 1.11 (1.02 to 1.22)	
	p-for-interaction = 0.17				p-for-interaction = 0.06			
Conditions of interest for treatment-related serious adverse events, No (%)								
Hypotension	13 (2.6)	8 (1.6)	15 (1.7)	13 (1.4)	23 (2.7)	18 (2.1)	59 (2.4)	27 (1.1)
Syncope	9 (1.8)	9 (1.8)	18 (2.0)	16 (1.8)	18 (2.1)	18 (2.1)	62 (2.6)	37 (1.5)
Bradycardia	11 (2.2)	9 (1.8)	9 (1.0)	5 (0.6)	12 (1.4)	23 (2.7)	55 (2.3)	36 (1.5)
Electrolyte abnormality	14 (2.8)	15 (3.0)	25 (2.8)	22 (2.4)	28 (3.3)	29 (3.4)	77 (3.2)	41 (1.7)
Injurious fall	9 (1.8)	8 (1.6)	9 (1.0)	8 (0.9)	37 (4.3)	41 (4.8)	50 (2.1)	53 (2.2)
Acute kidney injury or acute renal failure	29 (5.9)	16 (3.2)	46 (5.2)	25 (2.7)	34 (4.0)	28 (3.3)	84 (3.5)	48 (2.0)

© 2022 Inoue K et al. *JAMA Network Open.*
