# Supplementary material for: Association of Intensive Blood Pressure Control and Living Arrangement on Cardiovascular Outcomes by Race: Post Hoc Analysis of SPRINT Randomized Clinical Trial
Source: JAMA Netw Open. 2022 Mar 14;5(3):e222037. doi: 10.1001/jamanetworkopen.2022.2037 (PMC9608340; doi:10.1001/jamanetworkopen.2022.2037)
Supplement: Supplement 3. — Data Sharing Statement [file jamanetwopen-e222037-s003.pdf]

Data Sharing Statement

Inoue. Association of Intensive Blood Pressure Control and Living Arrangement on Cardiovascular Outcomes by Race. *JAMA Netw Open*. Published March 14, 2022. doi:10.1001/jamanetworkopen.2022.2037

Data
Data available: No

Additional Information
Explanation for why data not available: This is a secondary analysis of SPRINT randomized controlled trial, and all data are already available at https://biolincc.nhlbi.nih.gov/studies/sprint/. There is no data collected particularly for our study.
